# Genome-wide RNA interference analysis of renal carcinoma survival regulators identifies MCT4 as a Warburg effect metabolic target

**DOI:** 10.1002/path.4006

**Published:** 2012-04-18

**Authors:** Marco Gerlinger, Claudio R Santos, Bradley Spencer-Dene, Pierre Martinez, David Endesfelder, Rebecca A Burrell, Marcus Vetter, Ming Jiang, Rebecca E Saunders, Gavin Kelly, Karl Dykema, Nathalie Rioux-Leclercq, Gordon Stamp, Jean Jacques Patard, James Larkin, Michael Howell, Charles Swanton

**Affiliations:** 1 Translational Cancer Therapeutics Laboratory, Cancer Research UK London Research Institute, Lincoln's Inn FieldsLondon, WC2A 3LY, UK; 2 Barts Cancer Institute, Queen Mary University of LondonCharterhouse Square, London, EC1M 6BQ, UK; 3 Experimental Histopathology Laboratory, Cancer Research UK London Research InstituteLincoln's Inn Fields, London, WC2A 3LY, UK; 4 Royal Marsden Hospital, Department of Medical Oncology, Renal UnitFulham Road London, UK; 5 High Throughput Screening Laboratory, Cancer Research UK London Research InstituteLincoln's Inn Fields, London, WC2A 3LY, UK; 6 Bioinformatics & Biostatistics Service, Cancer Research UK London Research InstituteLincoln's Inn Fields, London, WC2A 3LY, UK; 7 Lab of Computational Biology, Van Andel Research InstituteGrand Rapids, Michigan, USA; 8 Department of Pathology, Rennes University HospitalRennes, France; 9 Department of Urology, Bicêtre Hospital, Paris XI UniversityParis, France; 10 University College London Cancer InstituteHuntley Street, London, UK

**Keywords:** renal cell carcinoma, lactate transport, MCT4, SLC16A3, cancer metabolism, Warburg effect, survival regulator, therapeutic target

## Abstract

Clear cell renal cell carcinoma (ccRCC) is the most common pathological subtype of kidney cancer. Here, we integrated an unbiased genome-wide RNA interference screen for ccRCC survival regulators with an analysis of recurrently overexpressed genes in ccRCC to identify new therapeutic targets in this disease. One of the most potent survival regulators, the monocarboxylate transporter MCT4 (SLC16A3), impaired ccRCC viability in all eight ccRCC lines tested and was the seventh most overexpressed gene in a meta-analysis of five ccRCC expression datasets. MCT4 silencing impaired secretion of lactate generated through glycolysis and induced cell cycle arrest and apoptosis. Silencing MCT4 resulted in intracellular acidosis, and reduction in intracellular ATP production together with partial reversion of the Warburg effect in ccRCC cell lines. Intra-tumoural heterogeneity in the intensity of MCT4 protein expression was observed in primary ccRCCs. MCT4 protein expression analysis based on the highest intensity of expression in primary ccRCCs was associated with poorer relapse-free survival, whereas modal intensity correlated with Fuhrman nuclear grade. Consistent with the potential selection of subclones enriched for MCT4 expression during disease progression, MCT4 expression was greater at sites of metastatic disease. These data suggest that MCT4 may serve as a novel metabolic target to reverse the Warburg effect and limit disease progression in ccRCC.

Copyright © 2012 Pathological Society of Great Britain and Ireland. Published by John Wiley & Sons, Ltd.

## Introduction

Clear cell carcinoma (ccRCC) is the commonest subtype of renal cell carcinoma, accounting for 80% of cases. These tumours are highly resistant to cytotoxic chemotherapy and until recently, systemic treatment options for advanced ccRCC were limited to cytokine-based therapies, such as interleukin-2 and interferon-α. Recently, anti-angiogenic drugs and mTOR inhibitors, all targeting the HIF–VEGF axis which is activated in up to 91% of ccRCCs through loss of the VHL tumour suppressor gene 1, have been shown to be effective in metastatic ccRCC 2–5. Although these drugs increase overall survival to more than 2 years 6, resistance invariably occurs, making the identification of new molecular targets a major clinical need to improve outcomes in patients with metastatic ccRCC.

In this study, we combined a genome-wide siRNA screen in a ccRCC cell line with analysis of a tumour versus normal tissue mRNA expression dataset to identify genes essential for, and strongly overexpressed in, ccRCC. We show that the lactate transporter MCT4/SLC16A3 is necessary for the survival of ccRCC cell lines and highly overexpressed in ccRCCs. MCT4 is required for lactate secretion, pH homoeostasis, and maintenance of the Warburg effect. The high level of MCT4 expression, particularly in metastatic and aggressive ccRCCs, together with a highly restricted expression pattern in normal tissues suggests the utility of MCT4 as a potential new drug target.

## Materials and methods

### Genome-wide siRNA screen

RCC4 cells were transfected in 384 well plates and cell numbers determined with an Acumen Explorer (TTP Labtech, Melbourn, UK) after 4 days. A robust Z-score was calculated and smoothed for edge effects. Follow-up experiments were performed in 96-well plates and read similarly. Caspase 3/7 activity was determined with the Apo-ONE kit (Promega, Madison, WI, USA) and normalized with cell number.

### mRNA expression analysis

For the identification of siRNA screen hits overexpressed in ccRCC, expression data from GEO (GSE14994) were used. The meta-analysis of genes overexpressed in ccRCC and in metastatic versus primary tumours was performed and visualized on Oncomine™ (Compendia Bioscience, Ann Arbor, MI, USA). Expression of MCT1 and MCT4 in cancer cell lines was from the Cancer Genome Project cell line mRNA expression dataset (http://www.sanger.ac.uk/genetics/CGP).

### Cell cycle profiling/sub-G1 analysis

Cells were harvested and fixed in 70% ethanol, washed twice in phosphate–citrate buffer, and treated with RNase. DNA was stained with propidium iodide (50 µg/ml) and analysis was performed on a BD-LSRII flow cytometer. Cell populations with sub-G1 content were defined as apoptotic and cell cycle phases calculated using a Watson pragmatic model.

### Metabolic assays

Lactate secretion after 2 h of incubation in pyruvate-free medium with 11 mm glucose and 2 mm glutamine was measured with a BioVision kit and normalized to cell mass measured with a sulphorodamine B assay (Sigma, St Louis, MO, USA).

Intracellular lactate concentrations were determined in equal numbers of cells washed with ice-cold PBS (pH 5), trypsinized, and then lysed with distilled water.

ATP levels were measured in equal numbers of viable cells using the ENLITEN® ATP Assay System (Promega).

Glycolysis and oxygen consumption rates were estimated by measuring extracellular acidification and oxygen consumption rates in a Seahorse XF96 (Seahorse Bioscience, North Billerica, MA,USA) extracellular flux analyser 3 days after siRNA transfection. Cells were assayed in XF medium with 2 g/l glucose.

Intracellular pH: cells were loaded with 10 µm SNARF-4F (Molecular Probes, Eugene, OR, USA) and analysed in a BD-LSRII flow cytometer. The ratio between the 660 nm and the 585 nm emission was calculated after excitation with a yellow laser (561 nm) and a standard curve was built as described 7.

### MCT4 expression analysis in ccRCC specimens

Clinical samples immunostained for MCT4 expression were scored independently by two observers blinded to the associated clinical data as follows: 1 = no or minimal expression; 2 = weak staining; 3 = intermediate staining; 4 = strong staining. Highest and modal MCT4 expression was recorded for each sample. Relapse-free and overall survival was estimated using the Kaplan–Meier method from the time of surgery to relapse or from diagnosis of metastatic disease to death from any cause, respectively. Statistical significance was calculated by the log-rank test where appropriate. Follow-up was calculated for patients alive at the last follow-up.

### Statistical analysis

Student's *t*-test was used to compare the means of independent samples.

### Supplementary materials and methods

Supplementary materials and methods may be found in the Supporting information.

## Results

**Figure 1:**
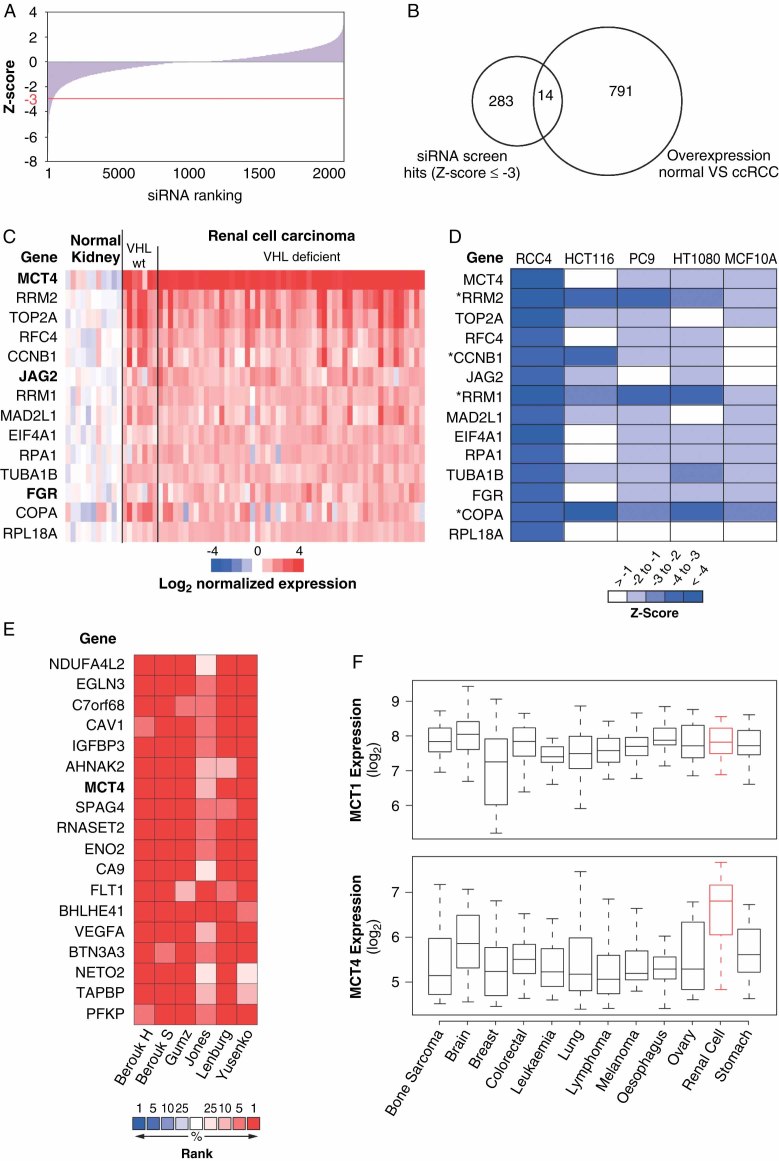
(A) Genome-wide siRNA screen to identify genes required for proliferation and/or survival of RCC4 cells. Z-scores of cell numbers after 4 days of transfection are displayed. The red line indicates the cut-off (Z-score ≤− 3) for selection of hits. (B) Overlap between the 297 essential genes in RCC4 cells (Z-score ≤− 3) and the 805 genes overexpressed in clear cell renal carcinoma compared with normal kidney tissue. Expression data from ref 8. (C) Expression levels of the 14 genes identified in B in normal kidney and in ccRCC tumours, either wt or deficient for VHL. Values are log_2_ normalized to the median expression in the normal kidney samples. (D) Effect of silencing the 14 genes identified in B on proliferation of RCC4 and four non-ccRCC cell lines in genome-wide siRNA screens displayed as a heat map of the Z-scores. *Genes whose silencing is detrimental in non-ccRCC cell lines. (E) *MCT4* is the seventh most overexpressed gene in ccRCC based on a meta-analysis of five different ccRCC expression datasets available in Oncomine 8, 23, 43–45. Genes are ordered based on their median expression rank across the datasets. Berouk H and S refer to hereditary and sporadic ccRCCs from the Beroukhim dataset. (F) Comparison of *MCT1* and *MCT4* expression in ccRCC versus non-ccRCC cell lines. Normalized log_2_ expression is displayed as boxplots. The whiskers extend to the most extreme data point that is no more than 1.5 times the interquartile range from the box

A genome-wide siRNA screen targeting over 21 000 genes was performed in the VHL-deficient RCC4 cell line to identify essential genes for proliferation or survival of ccRCC cells. Cell numbers were measured 4 days after siRNA transfection and data were normalized and transformed into a Z-score indicating how many standard deviations the cell numbers of an individual experiment were above or below the median. Two hundred and ninety-seven genes had a Z-score of − 3 or below, indicating a statistically significant reduction in cell number (Figure 1A and Supporting information, Supplementary Table 1). In order to focus our analysis on genes that may be important in ccRCC biology, we investigated which of those were significantly overexpressed in 59 ccRCC tumour specimens in comparison to 11 normal kidney samples in a published mRNA expression dataset 8. Fourteen of the 297 genes identified in the screen were among the 805 genes that were significantly overexpressed (≥ two-fold overexpression, FDR = 1%) in tumour samples (Figures 1B and 1C).

Unpublished data from similar genome-wide siRNA screens previously performed in our High-Throughput Screening Laboratory in four non-ccRCC cell lines showed that ten out of the 14 genes had no significant anti-proliferative effects when silenced, suggesting that they may be ccRCC-specific survival regulators.

One of the genes identified through this approach was that encoding the Notch ligand JAG2. A literature search revealed that targeting Notch signalling with γ-secretase inhibitors attenuates the growth of ccRCC cell lines *in vitro* and *in vivo* 9. Silencing of JAG2 reduced the cell number by 50–90% in a panel of eight ccRCC cell lines but not in the immortalized embryonic kidney cell line HEK293 (Supporting information, Supplementary Figure 1A); these were efficiently killed by the silencing of *PLK1* and *UBB* (Supporting information, Supplementary Figure 1B), validating our integrative functional genomics approach for therapeutic target identification.

Of the 14 overexpressed genes, *SLC16A3/MCT4* was the most highly and consistently overexpressed in both VHL-wild type and VHL-deficient ccRCCs (Figure 1C). A meta-analysis of five ccRCC expression datasets revealed that *MCT4* is the seventh most strongly overexpressed gene in this tumour type (Figure 1E). Expression analysis of a panel of 541 cancer cell lines comprising 12 tumour types revealed higher expression of *MCT4* in ccRCC relative to cell lines from other tumour entities. In contrast, expression levels of the related monocarboxylate transporter *SLC16A1/MCT1* were similar in all tumour types (Figure 1F).

Standard deconvolution techniques 10 were applied to validate the pool of four *MCT4* siRNAs used in the screen. The four individual siRNAs reduced *MCT4* mRNA levels and impaired proliferation to a similar degree to the pooled siRNAs (Supporting information, Supplementary Figure 2A). Comparable results were obtained with different *MCT4* siRNA sequences from another manufacturer (Supporting information, Supplementary Figure 2B). The reproducible correlation between the degree of mRNA knockdown and the anti-proliferative effect of all siRNAs indicates that *MCT4* silencing, rather than off-target effects attributable to individual siRNAs, led to the anti-proliferative effect.

**Figure 2:**
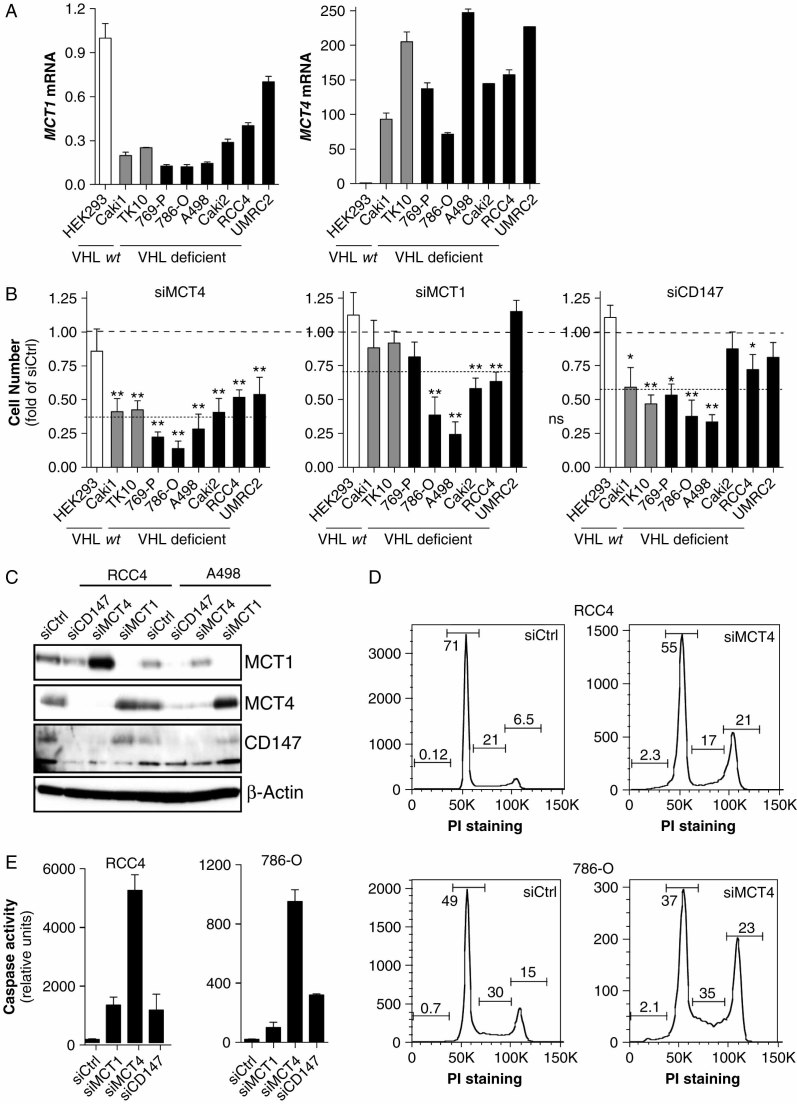
(A) Q-PCR expression analysis of *MCT1* and *MCT4* in eight ccRCC cell lines and in the embryonic kidney cell line HEK293. Expression was normalized with *B*2*M* levels and represented as fold of expression in HEK293. Graphs show mean ± SD (*n* = 3). (B) Effect of *MCT1, MCT4*, and *CD147* silencing in a panel of eight ccRCC cell lines and in HEK293. Four days after transfection with siRNA, cell numbers were determined and normalized to non-targeting siRNAs. Graphs show mean ± SD (*n* = 6). *p < 0.01; **p < 0.001. (C) Expression of MCT4 and CD147 is co-dependent. RCC4 and A498 were transfected with siRNA,lysed after 3 days and used for western blot analysis. (D) *MCT4* silencing induces G2/M cell cycle arrest in RCC4 and 786-O. RCC4 and A498 were transfected with siRNA, stained with PI after 3 days, and analysed by FACS. Number represent the percentage of cells in sub-G1 and phases of the cell cycle. (E) *MCT4* silencing induces apoptosis in RCC4 and 786-O. Cells were transfected with siRNA and caspase 3/7 activity and cell numbers were determined after 4 days. Graphs show mean ± SD of caspase activity normalized to cell number in the same well (*n* = 3)

MCT4 is one of four monocarboxylate transporters (MCT1–4) that shuttle pyruvate or lactate and protons across the plasma membrane in a gradient-dependent manner 11. Most cancers display an increase in glucose usage and lactate secretion even in the presence of oxygen, a feature known as aerobic glycolysis or the Warburg effect (WE) 12, 13. Based on substrate affinities and transport kinetics, MCT4 is assumed to be the main transporter responsible for the secretion of lactate out of highly glycolytic cells 14, 15. *MCT1* and *MCT4* were expressed in all ccRCC cell lines, whereas HEK293 expressed only *MCT1* (Figure 2A). *SLC16A7/MCT2* and *SCL16A8/MCT3* were not detectable by qPCR (data not shown).

We further investigated the dependencies of ccRCC cells on *MCT1* and *MCT4* expression. Silencing *MCT4* significantly reduced proliferation in eight ccRCC cell lines tested, with no obvious distinction in sensitivity based on VHL status (Figure 2B, mean reduction 63%). Silencing of *MCT1* with a validated siRNA pool (Supporting information, Supplementary Figure 2C) in the same cell line panel significantly reduced the cell number in only four ccRCC cell lines (Figure 2B, mean reduction 30%). The embryonic kidney cell line HEK293 was insensitive to *MCT4* and *MCT1* silencing. Neither silencing of *MCT4* nor that of *MCT1* significantly affected the mRNA levels of the other transporter (Supporting information, Supplementary Figure 2D).

MCT1 and MCT4 form complexes with the ancillary protein CD147, which is required for protein expression and activity of both transporters 16–18. *CD147* silencing has been described as a strategy to reduce the expression of both MCT1 and MCT4 16. Accordingly, MCT1 and MCT4 protein levels were decreased by *CD147* silencing (Figure 2C), which significantly impaired proliferation in six out of eight ccRCC cell lines (Figure 2B, mean reduction 42%). CD147 expression is lost after silencing *MCT4* but not *MCT1* (Figure 2C). Taken together, these data suggest that only the MCT4/CD147 complex is essential for proliferation and survival of ccRCC cells.

We next sought to investigate the mechanisms contributing to the reduction in cell number after *MCT4* silencing. Cell cycle profiling of ccRCC cell lines 4 days after *MCT4* silencing showed a G2/M-phase arrest and an increase in the apoptotic sub-G1 fraction compared with control transfected cells (Figure 2D). Apoptosis induction was confirmed by an increase in caspase 3/7 activity (Figure 2E). Thus, *MCT4* silencing results in both cytotoxic and cytostatic effects.

**Figure 3:**
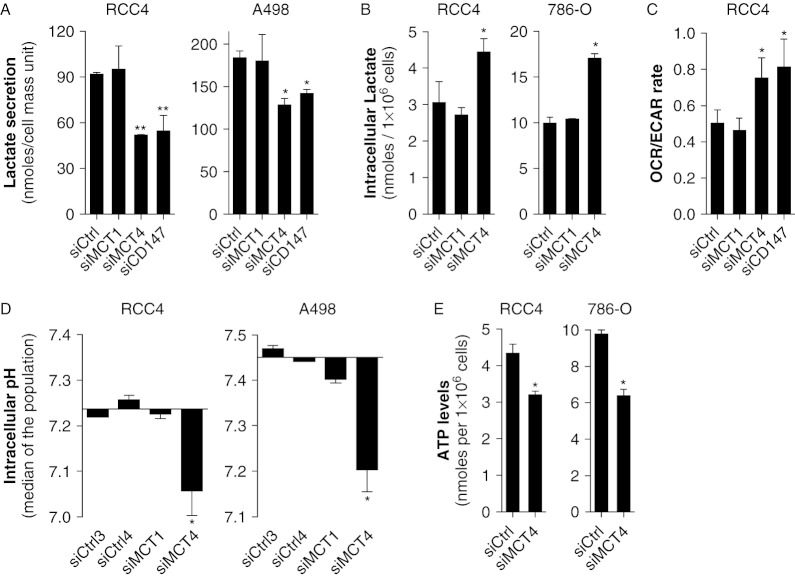
(A) *MCT4* and *CD147* silencing reduces lactate secretion in RCC4 and in A498. Following 2 days of transfection, cells were incubated in fresh medium for 2 h. Lactate secreted into the medium was quantified and normalized to cell mass content (siCtrl = 1 cell mass unit). Graph shows mean ± SD (*n* = 3). *p < 0.05; **p < 0.001. (B) *MCT4* silencing leads to intracellular accumulation of lactate in RCC4 and 786-O. Two days after transfection, an identical number of cells were lysed in water and lactate was quantified. Graphs show mean ± SD (*n* = 3). *p < 0.05. (C) Silencing of *MCT4* impairs glycolysis in RCC4. Three days after transfection, extracellular acidification (ECAR) and oxygen consumption (OCR) rates were determined using a Seahorse XF96; the ratio between ECAR and OCR was calculated for each well. Graphs show mean ± SD (*n* = 6). *p < 0.001. (D) *MCT4* silencing induces intracellular acidification in RCC4 and A498. Cells were loaded with SNARF-4F 2 days after transfection and analysed by FACS. The 660/585 nm emission ratio was determined after excitation with a yellow laser. A standard curve generated with cells incubated at pH 6, 7, and 8 was used to calculate the intracellular pH of each sample. Graphs show mean ± SD (*n* = 3). *p < 0.05 versus both siCtrls. (E) *MCT4* silencing reduces ATP levels in RCC4 and 786-O. ATP levels were measured 2 days after siRNA transfection in identical cell numbers. Graphs show mean ± SD (*n* = 3). *p < 0.01

To confirm that the essential function of MCT4 in ccRCC model systems relates to its role in lactate secretion and in maintaining glycolysis, we investigated the metabolic consequences of MCT4 knockdown. Silencing of *MCT4* and *CD147* reduced lactate secretion in ccRCC cell lines, whereas *MCT1* silencing had no effect (Figure 3A). Lactate accumulated intracellularly following *MCT4* repression (Figure 3B), confirming that this transporter is important for lactate secretion in ccRCC cells. Measurements of the extracellular acidification and the oxygen consumption rates after silencing of *MCT4* or *CD147* revealed a reduction of glycolysis relative to oxygen consumption, indicating that MCT4/CD147 loss renders cells less glycolytic (Figure 3C).

MCTs contribute to intracellular pH regulation by co-transporting lactate together with a proton 19. Silencing of *MCT4*, but not *MCT1*, decreased intracellular pH in two ccRCC cell lines by 0.2 and 0.25 units, respectively (Figure 3D), indicating that MCT4 is important to maintain pH homeostasis. *MCT4* silencing also decreased cellular ATP levels (Figure 3E), which may be a consequence of the inhibition of glycolysis by intracellular acidification 20.

**Figure 4:**
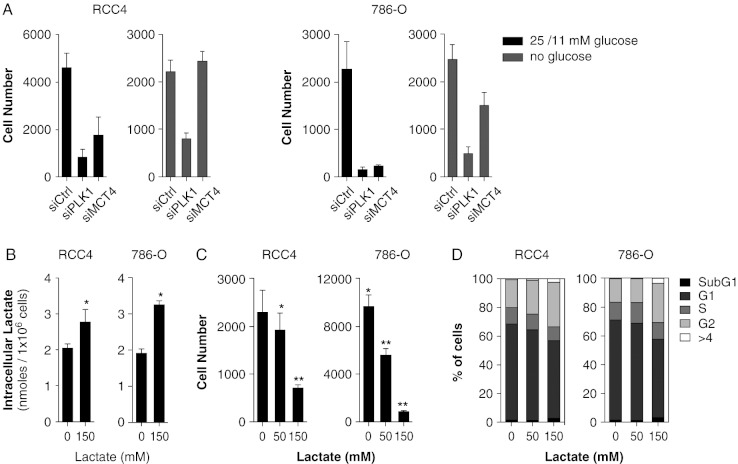
(A) Glucose depletion reduces sensitivity of RCC4 and 786-O to *MCT4* silencing. Cells were transfected as indicated and incubated either in glucose-free or in medium with 25 (RCC4) or 11 (786-O) mm glucose. Four days after transfection, cell number was determined. Graphs show mean ± SD (*n* = 6). (B) Addition of lactate to the medium induces accumulation of intracellular lactate in RCC4 and 786-O. Two days after incubation in the presence or absence of 150 mm lactate, an identical number of cells were lysed in water and lactate was quantified. Graphs show mean ± SD (*n* = 3). *p < 0.01. (C) Addition of lactate to the medium reduces proliferation of RCC4 and 786-O. Cells were grown in the presence of the indicated concentrations of lactate. After 4 days, cell number was determined. Graphs show mean ± SD (*n* = 12). *p < 0.05; **p < 0.001. (D) Addition of lactate to the medium induces G2 arrest in RCC4 and 786-O. Cells were grown in the presence of the indicated concentrations of lactate, DAPI-stained, and analysed on an Acumen Explorer eX3

To further demonstrate that ccRCCs require MCT4 to excrete glucose-derived lactate, we investigated the effect of silencing MCT4 in cells growing in glucose-free medium. ccRCC cell lines proliferated less in the absence of glucose, and silencing of *MCT4* under these conditions had a blunted effect on proliferation compared with glucose-replete conditions (Figure 4A), while suppression of *PLK1*, a gene essential for mitosis 21, was equally detrimental in both conditions. ccRCC cells were also grown in the presence of 150 mm exogenous lactate. Since MCT4 is a passive co-transporter that shuttles lactate and protons in the direction of transmembrane gradients 11, this led to the accumulation of lactate intracellularly (Figure 4B). Incubation in these conditions reduced proliferation (Figure 4C) and led to the accumulation of cells in G2/M (Figure 4D), mimicking the effect of *MCT4* silencing. Taken together, these observations indicate that ccRCC cells depend on MCT4 expression to eliminate lactate produced by glycolysis.

**Figure 5:**
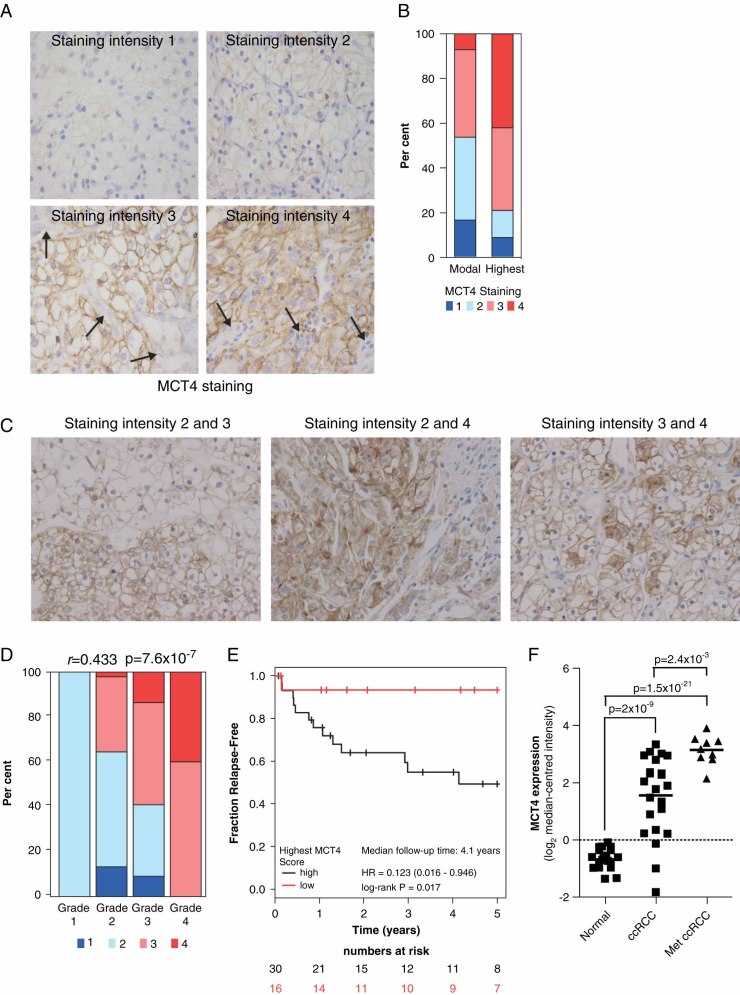
(A) ccRCC FFPE specimens demonstrating representative MCT4 staining intensities (original magnification 20×). Arrows indicate stromal cells. (B) Distribution of modal and highest MCT4 staining intensities in 127 primary ccRCC tumours. (C) ccRCC FFPE specimens demonstrating intra-tumoural heterogeneity in MCT4 staining intensity (original magnification 20×). (D) Correlation between modal MCT4 staining intensity and Fuhrman tumour grade. Pearson correlation coefficient (*r*) was calculated (*n* = 127). (E) Relapse-free survival (RFS) by highest observed MCT4 staining in tumour specimens of patients treated surgically for early-stage ccRCC (low = MCT4 staining intensity 1 or 2; high = MCT4 staining intensity 3 or 4). (F) *MCT4* mRNA expression in primary ccRCC versus metastatic ccRCC versus normal kidney specimens. Expression data from ref 23

MCT4 protein expression was assessed by immunostaining 127 surgical primary ccRCC specimens. MCT4 staining intensity was scored on a scale from 1 (lowest expression) to 4 (highest expression; for details see the Materials and methods section). Examples of staining intensities are shown in Figure 5A. MCT4 expression was detectable in the majority of ccRCC samples (Figure 5B), consistent with mRNA expression data (Figure 1C). Whereas stromal expression of MCT4 has been shown in breast cancer 22, in ccRCCs the stromal compartment was predominantly MCT4-negative (arrows in Figure 5A). The cancer cells in many specimens displayed heterogeneous MCT4 staining (Figure 5C) and both the modal and the highest staining intensities were recorded (Figure 5B).

Modal MCT4 expression was highly correlated with Fuhrman nuclear grade (Figure 5D, *p* < 0.001). Relapse-free survival (RFS) analysis in 46 patients with early-stage disease treated with potentially curative surgery and for whom follow-up data were available showed a significantly worse RFS in patients with tumours showing MCT4 staining intensities of 3 or 4 (Figure 5E, *p* = 0.017). The difference in RFS was only apparent if tumours were categorized based on the highest observed MCT4 expression within the samples but not when categorized based on the modal staining intensity. This may indicate that a subset of ccRCC cells with high MCT4 expression has an increased ability to disseminate from a heterogeneous primary tumour. This was further supported by significantly higher *MCT4* mRNA expression levels in metastatic ccRCC lesions compared with primary tumours (Figure 5F), consistent with the colonization of metastatic sites by subclones with high MCT4 expression. Neither modal nor highest MCT4 expression in the primary tumour correlated with overall survival in 50 patients with metastatic disease (Supporting information, Supplementary Figure 3).

To evaluate potential toxicities of MCT4 targeting approaches, we immunostained several normal tissues for MCT4; high expression was found only in skeletal muscle, bone marrow, and Brunner's glands of the duodenum (Supporting information, Supplementary Figure 4). Taken together, these data suggest the potential of MCT4 targeting to limit ccRCC growth.

## Discussion

We integrated whole-genome siRNA screening, mRNA expression analysis, and metabolic assays to identify the monocarboxylate transporter MCT4 as a potential novel therapeutic target in ccRCC. *MCT4* was the seventh most overexpressed gene in ccRCC and its overexpression was independent of VHL status. MCT4 abrogation reduced lactate secretion, intracellular pH and ATP levels, and led to G2/M arrest and apoptosis in ccRCC cell lines. High-level expression of MCT4 in primary tumours correlated with poor RFS, and metastatic lesions displayed higher MCT4 protein expression than primary tumours. Taken together, these data suggest the potential of MCT4 to serve as a novel therapeutic target in ccRCC. Importantly, the discovery of an MCT1/MCT2 isoform-specific inhibitor 24, 25 suggests that isoform-specific targeting of MCT4 may also be possible.

MCT4's main function is the secretion of lactate and protons from highly glycolytic cells 15. High rates of glucose uptake and lactate secretion are features of the WE that contribute to support ATP production and provide intermediates for biosynthesis in many tumours 12, 13, 26. Inactivation of the VHL tumour suppressor gene leads to the stabilization of hypoxia-inducible factor 1 alpha (HIF1α), which induces the WE and up-regulates the direct HIF1α target MCT4 27. Aerobic glycolysis can also be induced by oncogene activation, such as *MYC* amplification, or loss of certain tumour suppressors such as PTEN 28, both of which are known to occur in ccRCC 29, 30. This may explain our observations that *MCT4* expression is independent of VHL status in ccRCC.

*MCT4* knockdown, but not silencing of the related MCT1 transporter, impaired proliferation of all the ccRCC cell lines tested, indicating that ccRCCs depend strongly on MCT4 rather than MCT1 for survival. We have demonstrated through a series of metabolic assays that *MCT4* knockdown impairs the cell's ability to secrete the weak acid lactate, which consequently leads to a reduction in intracellular pH. A similar decrease in pH has been shown to increase caspase activation by two- to three-fold 31, which is consistent with the induction of apoptosis that we observed after *MCT4* silencing. The reduction in ATP levels is also a potential consequence of this alteration in the acid–base balance 20. Accumulation of intracellular lactate may also inhibit its conversion from pyruvate by lactate dehydrogenase 32, the main reaction regenerating NAD^+^ in highly glycolytic cells 33. Silencing of *MCT4* also led to a G2/M cell cycle arrest, indicating that there may be additional links between metabolism and cell cycle progression apart from those described recently 34.

*MCT4* silencing led to a concomitant loss in the expression of CD147, an ancillary protein necessary for MCT activity 35 that is required for *in vivo* growth of colon carcinoma 16 and pancreatic 36 cancer cells. Although CD147 interacts with other proteins, it has been shown that its major pro-tumour role is to control MCT1/4 activity 16. This is in agreement with our observation that high concentrations of lactate in the medium, which led to the accumulation of intracellular lactate, reduced cell viability and impaired cell cycle progression, phenocopying the consequences of *MCT4* silencing. Conversely, glucose withdrawal, which reduces lactate production, attenuated the effect of *MCT4* knockdown. Thus, it is likely that the accumulation of intracellular lactate is responsible for the impact on cell viability after *MCT4* silencing, rather than loss of another function attributable to CD147.

Silencing or pharmacological inhibition of MCT1 in colon cancer cells has been shown to be effective only in the absence of MCT4 expression, indicating functional redundancy of MCT1 and MCT4 16. In contrast, our results highlight an essential and non-redundant function of MCT4 in ccRCC. Higher expression of MCT4 in ccRCC cell lines compared with several other tumour types may be indicative of a higher dependence on aerobic glycolysis and thus on the MCT4 isoform, optimized for lactate secretion. A decisive advantage of an MCT4 isoform-specific inhibitor for ccRCC therapeutic use would be the highly restricted expression in normal body tissues in comparison to the ubiquitously expressed MCT1 11. High-level expression of MCT4 in muscle tissue could be a potential hurdle to therapeutic targeting.

The presence of ccRCC cells with high-level expression of MCT4 in tumour specimens correlated with relapse-free survival after surgery for early-stage cancers. This association with RFS was only observed when tumours were scored for the highest MCT4 expression rather than modal MCT4 expression, suggesting that intra-tumour heterogeneity may impact on phenotypic differences in metastatic potential. This is consistent with reports showing that high MCT4 expression and lactate secretion increase cell motility and invasive potential in *in vitro* models of breast 17 and lung 37 cancer, and that MCT4 causes extracellular acidification in xenografts 38, which may contribute to matrix degradation and invasion (reviewed in ref 39).

Recent studies demonstrated that aerobic glycolysis in tumour cells can be targeted through inhibition of the glucose transporter GLUT1 40, LDH-A 41 or PFKFB3 42. Our study shows that the inhibition of lactate elimination through MCT4 targeting may be a novel strategy to target tumour-specific metabolic alterations such as the WE. The development of inhibitors specific to the MCT4 isoform would be a prerequisite to examine this hypothesis in patients with ccRCC. Such developments might add a novel therapeutic class of agents, which target tumour metabolism, for the treatment of kidney cancer.

## SUPPORTING INFORMATION ON THE INTERNET

The following supporting information may be found in the online version of this article.

**Supplementary materials and methods**

**Table S1.** Results of a genome-wide siRNA screen for survival regulators in the RCC4 cell line.

**Figure S1.** (A) A panel of renal cell carcinoma cell lines is highly sensitive to silencing of JAG2. (B) Silencing of PLK1 and UBB efficiently reduce cell numbers in HEK293 4 days after transfection.

**Figure S2.** (A) Effect of MCT4 silencing on cell number correlates well with silencing efficiency. (B) MCT4 silencing phenotype is also observed using different siRNA sequences obtained from another manufacturer. (C) The effect of silencing MCT1 on cell number is not significant despite efficient silencing. (D) MCT1 and MCT4 silencing is specific.

**Figure S3.** Overall survival (OS) by highest and modal MCT4 staining intensity in tumour specimens of patients with metastatic disease treated with a palliative nephrectomy.

**Figure S4.** MCT4 expression in normal tissues (20×).

## References

[1] Linehan WM, Srinivasan R, Schmidt LS (2010). The genetic basis of kidney cancer: a metabolic disease. Nature Rev Urol.

[2] Motzer RJ, Hutson TE, Tomczak P (2007). Sunitinib versus interferon alfa in metastatic renal-cell carcinoma. N Engl J Med.

[3] Escudier B, Eisen T, Stadler WM (2007). Sorafenib in advanced clear-cell renal-cell carcinoma. N Engl J Med.

[4] Hudes G, Carducci M, Tomczak P (2007). Temsirolimus, interferon alfa, or both for advanced renal-cell carcinoma. N Engl J Med.

[5] Motzer RJ, Escudier B, Oudard S (2008). Efficacy of everolimus in advanced renal cell carcinoma: a double-blind, randomised, placebo-controlled phase III trial. Lancet.

[6] Escudier B, Goupil MG, Massard C (2009). Sequential therapy in renal cell carcinoma. Cancer.

[7] van Erp PE, Jansen MJ, de Jongh GJ (1991). Ratiometric measurement of intracellular pH in cultured human keratinocytes using carboxy-SNARF-1 and flow cytometry. Cytometry.

[8] Beroukhim R, Brunet JP, Di Napoli A (2009). Patterns of gene expression and copy-number alterations in von-Hippel Lindau disease-associated and sporadic clear cell carcinoma of the kidney. Cancer Res.

[9] Sjolund J, Johansson M, Manna S (2008). Suppression of renal cell carcinoma growth by inhibition of Notch signaling *in vitro* and *in vivo*. J Clin Invest.

[10] Echeverri CJ, Beachy PA, Baum B (2006). Minimizing the risk of reporting false positives in large-scale RNAi screens. Nature Methods.

[11] Halestrap AP, Meredith D (2004). The *SLC16* gene family—from monocarboxylate transporters (MCTs) to aromatic amino acid transporters and beyond. Pflugers Arch.

[12] Vander Heiden MG, Cantley LC, Thompson CB (2009). Understanding the Warburg effect: the metabolic requirements of cell proliferation. Science.

[13] Hsu PP, Sabatini DM (2008). Cancer cell metabolism: Warburg and beyond. Cell.

[14] Manning Fox JE, Meredith D, Halestrap AP (2000). Characterisation of human monocarboxylate transporter 4 substantiates its role in lactic acid efflux from skeletal muscle. J Physiol.

[15] Dimmer KS, Friedrich B, Lang F (2000). The low-affinity monocarboxylate transporter MCT4 is adapted to the export of lactate in highly glycolytic cells. Biochem J.

[16] Le Floch R, Chiche J, Marchiq I (2011). CD147 subunit of lactate/H^+^ symporters MCT1 and hypoxia-inducible MCT4 is critical for energetics and growth of glycolytic tumors. Proc Natl Acad Sci U S A.

[17] Gallagher SM, Castorino JJ, Wang D (2007). Monocarboxylate transporter 4 regulates maturation and trafficking of CD147 to the plasma membrane in the metastatic breast cancer cell line MDA-MB-231. Cancer Res.

[18] Kirk P, Wilson MC, Heddle C (2000). CD147 is tightly associated with lactate transporters MCT1 and MCT4 and facilitates their cell surface expression. EMBO J.

[19] Parks SK, Chiche J, Pouyssegur J (2011). pH control mechanisms of tumor survival and growth. J Cell Physiol.

[20] Halperin ML, Connors HP, Relman AS (1969). Factors that control the effect of pH on glycolysis in leukocytes. J Biol Chem.

[21] Sumara I, Gimenez-Abian JF, Gerlich D (2004). Roles of polo-like kinase 1 in the assembly of functional mitotic spindles. Curr Biol.

[22] Whitaker-Menezes D, Martinez-Outschoorn UE, Lin Z (2011). Evidence for a stromal–epithelial ‘lactate shuttle’ in human tumors: MCT4 is a marker of oxidative stress in cancer-associated fibroblasts. Cell Cycle.

[23] Jones J, Otu H, Spentzos D (2005). Gene signatures of progression and metastasis in renal cell cancer. Clin Cancer Res.

[24] Ovens MJ, Manoharan C, Wilson MC (2010). The inhibition of monocarboxylate transporter 2 (MCT2) by AR-C155858 is modulated by the associated ancillary protein. Biochem J.

[25] Ovens MJ, Davies AJ, Wilson MC (2010). AR-C155858 is a potent inhibitor of monocarboxylate transporters MCT1 and MCT2 that binds to an intracellular site involving transmembrane helices 7–10. Biochem J.

[26] Shlomi T, Benyamini T, Gottlieb E (2011). Genome-scale metabolic modeling elucidates the role of proliferative adaptation in causing the Warburg effect. PLoS Comput Biol.

[27] Ullah MS, Davies AJ, Halestrap AP (2006). The plasma membrane lactate transporter MCT4, but not MCT1, is up-regulated by hypoxia through a HIF-1α-dependent mechanism. J Biol Chem.

[28] Cairns RA, Harris IS, Mak TW (2011). Regulation of cancer cell metabolism. Nature Rev Cancer.

[29] Nickerson ML, Jaeger E, Shi Y (2008). Improved identification of von Hippel–Lindau gene alterations in clear cell renal tumors. Clin Cancer Res.

[30] Cairns P, Evron E, Okami K (1998). Point mutation and homozygous deletion of PTEN/MMAC1 in primary bladder cancers. Oncogene.

[31] Matsuyama S, Llopis J, Deveraux QL (2000). Changes in intramitochondrial and cytosolic pH: early events that modulate caspase activation during apoptosis. Nature Cell Biol.

[32] Karlsson J, Hulten B, Sjodin B (1974). Substrate activation and product inhibition of LDH activity in human skeletal muscle. Acta Physiol Scand.

[33] Locasale JW, Cantley LC (2011). Metabolic flux and the regulation of mammalian cell growth. Cell Metab.

[34] Almeida A, Bolanos JP, Moncada S (2009). E3 ubiquitin ligase APC/C-Cdh1 accounts for the Warburg effect by linking glycolysis to cell proliferation. Proc Natl Acad Sci U S A.

[35] Weidle UH, Scheuer W, Eggle D (2010). Cancer-related issues of CD147. Cancer Genomics Proteomics.

[36] Schneiderhan W, Scheler M, Holzmann KH (2009). CD147 silencing inhibits lactate transport and reduces malignant potential of pancreatic cancer cells in *in vivo* and *in vitro* models. Gut.

[37] Izumi H, Takahashi M, Uramoto H (2011). Monocarboxylate transporters 1 and 4 are involved in the invasion activity of human lung cancer cells. Cancer Sci.

[38] Chiche J, Le Fur Y, Vilmen C (2012). *In vivo* pH in metabolic-defective Ras-transformed fibroblast tumors: key role of the monocarboxylate transporter, MCT4, for inducing an alkaline intracellular pH. Int J Cancer.

[39] Webb BA, Chimenti M, Jacobson MP (2011). Dysregulated pH: a perfect storm for cancer progression. Nature Rev Cancer.

[40] Chan DA, Sutphin PD, Nguyen P (2011). Targeting GLUT1 and the Warburg effect in renal cell carcinoma by chemical synthetic lethality. Sci Transl Med.

[41] Le A, Cooper CR, Gouw AM (2010). Inhibition of lactate dehydrogenase A induces oxidative stress and inhibits tumor progression. Proc Natl Acad Sci U S A.

[42] Clem B, Telang S, Clem A (2008). Small-molecule inhibition of 6-phosphofructo-2-kinase activity suppresses glycolytic flux and tumor growth. Mol Cancer Ther.

[43] Gumz ML, Zou H, Kreinest PA (2007). Secreted frizzled-related protein 1 loss contributes to tumor phenotype of clear cell renal cell carcinoma. Clin Cancer Res.

[44] Lenburg ME, Liou LS, Gerry NP (2003). Previously unidentified changes in renal cell carcinoma gene expression identified by parametric analysis of microarray data. BMC Cancer.

[45] Yusenko MV, Kuiper RP, Boethe T (2009). High-resolution DNA copy number and gene expression analyses distinguish chromophobe renal cell carcinomas and renal oncocytomas. BMC Cancer.

[46] Gentleman RC, Carey VJ, Bates DM (2004). Bioconductor: open software development for computational biology and bioinformatics. Genome Biol.

[47] Smyth GK, Gentleman R, Carey V, Huber W (2005). Limma: linear models for microarray data. Bioinformatics and Computational Biology Solutions Using R and Bioconductor.

