# Supplementary material for: Genome-wide RNA interference analysis of renal carcinoma survival regulators identifies MCT4 as a Warburg effect metabolic target
Source: J Pathol. 2012 Apr 18;227(2):146–56. doi: 10.1002/path.4006 (PMC3504091; doi:10.1002/path.4006)
Supplement: Supplementary file 1 [file path0227-0146-SD6.pdf]

| GeneID | GeneName | Z-score | GeneID | GeneName | Z-score |
|---|---|---|---|---|---|
| NM_001034 | RRM2 | -12.16 | NM_021009 | UBC | -10.43 |
| NM_001033 | RRM1 | -10.25 | NM_018225 | SMU1 | -9.84 |
| XM_371107 | LOC388460 | -9.34 | XM_292836 | LOC342994 | -9.04 |
| NM_000978 | RPL23 | -8.88 | NM_015414 | RPL36 | -8.51 |
| NM_007209 | RPL35 | -8.49 | NM_003333 | UBA52 | -8.31 |
| NM_003973 | RPL14 | -8.16 | NM_080662 | PEX11G | -8.14 |
| XM_208281 | LOC285053 | -8.12 | NM_001997 | FAU | -8.08 |
| NM_032040 | CCDC8 | -8.06 | XM_209178 | LOC284393 | -7.97 |
| NM_004523 | KIF11 | -7.52 | XM_370872 | LOC388132 | -7.47 |
| XM_371757 | LOC389305 | -7.42 | XM_375543 | LOC400652 | -7.35 |
| NM_012433 | SF3B1 | -7.29 | NM_178431 | LCE3A | -7.13 |
| NM_002786 | PSMA1 | -7.08 | NM_014814 | P44S10 | -7.08 |
| XM_371781 | LOC389342 | -7 | XM_371853 | LOC389435 | -6.76 |
| XM_371115 | LOC388474 | -6.76 | NM_001005 | RPS3 | -6.71 |
| NM_005030 | PLK1 | -6.7 | XM_039702 | LOC145767 | -6.66 |
| XM_034594 | KIAA1604 | -6.59 | NM_005877 | SF3A1 | -6.57 |
| NM_002952 | RPS2 | -6.55 | XM_087499 | LOC152663 | -6.53 |
| NM_002875 | RAD51 | -6.45 | XM_371160 | LOC388532 | -6.45 |
| NM_005441 | CHAF1B | -6.43 | NM_003103 | SON | -6.42 |
| XM_373633 | LOC388134 | -6.4 | NM_018140 | FLJ10565 | -6.36 |
| XM_371023 | LOC388344 | -6.34 | XM_370713 | LOC387907 | -6.31 |
| NM_002696 | POLR2G | -6.22 | NM_032704 | TUBA6 | -6.18 |
| XM_371843 | LOC389425 | -6.18 | XM_056681 | LOC144581 | -6.17 |
| NM_001009 | RPS5 | -6.06 | NM_001024 | RPS21 | -6.04 |
| NM_004766 | COPB2 | -6.02 | NM_005805 | PSMD14 | -6.01 |
| NM_138484 | SGOL1 | -5.91 | NM_001237 | CCNA2 | -5.9 |
| XM_371330 | LOC388720 | -5.89 | NM_002295 | LAMR1 | -5.87 |
| NM_002812 | PSMD8 | -5.87 | NM_007104 | RPL10A | -5.87 |
| NM_001168 | BIRC5 | -5.76 | NM_020238 | INCENP | -5.76 |
| NM_006930 | SKP1A | -5.75 | XM_209704 | LOC285658 | -5.71 |
| NM_001416 | EIF4A1 | -5.64 | NM_000994 | RPL32 | -5.62 |
| NM_030931 | DEFB126 | -5.62 | NM_003170 | SUPT6H | -5.6 |
| XM_114317 | LOC200916 | -5.59 | XM_016713 | LOC146053 | -5.57 |
| XM_373343 | LOC392447 | -5.54 | XM_376154 | LOC401019 | -5.5 |
| NM_012177 | FBXO5 | -5.41 | NM_016633 | ERAF | -5.33 |
| NM_024011 | CDC2L2 | -5.3 | NM_012112 | TPX2 | -5.25 |
| NM_004094 | EIF2S1 | -5.23 | XM_029805 | LOC90193 | -5.22 |
| XM_371151 | LOC388519 | -5.2 | NM_015932 | C13ORF12 | -5.18 |
| XM_371470 | LOC388907 | -5.15 | XM_086494 | LOC149329 | -5.13 |
| NM_006013 | RPL10 | -5.11 | NM_173687 | FLJ37131 | -5.08 |
| XM_291428 | LOC343153 | -5.03 | NM_004856 | KIF23 | -5.02 |
| NM_000998 | RPL37A | -5.01 | NM_003755 | EIF3S4 | -5 |
| NM_018955 | UBB | -4.99 | NM_002140 | HNRPK | -4.91 |
| NM_014338 | PISD | -4.91 | XM_034640 | LOC158345 | -4.88 |
| XM_047083 | LOC92755 | -4.88 | NM_004207 | SLC16A3 | -4.87 |

| | | | | | |
|---|---|---|---|---|---|
| NM_016128 | COPG | -4.87 | NM_182583 | FLJ38374 | -4.84 |
| NM_006083 | IK | -4.82 | NM_014740 | DDX48 | -4.8 |
| NM_030763 | NSBP1 | -4.8 | NM_001274 | CHEK1 | -4.79 |
| NM_001259 | CDK6 | -4.76 | NM_001253 | CDC5L | -4.76 |
| NM_015077 | SARM1 | -4.75 | NM_145662 | SPANXA2 | -4.72 |
| NM_006072 | CCL26 | -4.71 | NM_005303 | GPR40 | -4.7 |
| NM_001708 | OPN1SW | -4.67 | NM_194295 | DKFZP434I1020 | -4.67 |
| NM_018518 | MCM10 | -4.66 | NM_012245 | SKIIP | -4.65 |
| NM_018685 | ANLN | -4.64 | NM_002787 | PSMA2 | -4.63 |
| XM_370727 | LOC387930 | -4.57 | NM_017431 | PRKAG3 | -4.56 |
| NM_001898 | CST1 | -4.56 | NM_001813 | CENPE | -4.55 |
| NM_002410 | MGAT5 | -4.53 | NM_015956 | MRPL4 | -4.53 |
| XM_379594 | LOC286149 | -4.53 | NM_032680 | MGC4266 | -4.52 |
| NM_138820 | MGC2198 | -4.51 | NM_006565 | CTCF | -4.5 |
| NM_006362 | NXF1 | -4.5 | NM_194303 | C10ORF39 | -4.48 |
| NM_003318 | TTK | -4.47 | NM_004958 | FRAP1 | -4.44 |
| NM_004247 | U5-116KD | -4.44 | NM_005008 | NHP2L1 | -4.41 |
| NM_006445 | PRPF8 | -4.4 | NM_004568 | SERPINB6 | -4.4 |
| NM_006325 | RAN | -4.37 | NM_016057 | COPZ1 | -4.37 |
| XM_165448 | LOC220717 | -4.36 | NM_153260 | FLJ36812 | -4.33 |
| NM_173678 | FLJ40722 | -4.32 | NM_005187 | CBFA2T3 | -4.29 |
| NM_001004353 | LOC441476 | -4.25 | NM_014502 | PRP19 | -4.24 |
| NM_032875 | FBXL20 | -4.22 | XM_377761 | LOC402100 | -4.21 |
| NM_003173 | SUV39H1 | -4.2 | NM_002945 | RPA1 | -4.19 |
| NM_018383 | WDR33 | -4.19 | NM_004965 | HMGN1 | -4.18 |
| NM_001211 | BUB1B | -4.17 | NM_005115 | MVP | -4.17 |
| NM_000969 | RPL5 | -4.16 | NM_014756 | CH-TOG | -4.16 |
| XM_058721 | LOC123722 | -4.16 | NM_002946 | RPA2 | -4.15 |
| XM_062912 | LOC122038 | -4.14 | NM_031287 | SF3B5 | -4.12 |
| NM_145273 | TREM4 | -4.11 | NM_001101 | ACTB | -4.1 |
| NM_052997 | ANKRD30A | -4.1 | XM_372048 | LOC389672 | -4.1 |
| XM_374437 | LOC392670 | -4.1 | NM_005387 | NUP98 | -4.09 |
| NM_018093 | FLJ10439 | -4.08 | NM_001067 | TOP2A | -4.07 |
| NM_003908 | EIF2S2 | -4.07 | NM_017777 | FLJ20345 | -4.07 |
| NM_194293 | CMYA1 | -4.07 | NM_006582 | GMEB1 | -4.06 |
| NM_000937 | POLR2A | -4.05 | NM_002811 | PSMD7 | -4.05 |
| XM_294473 | LOC346950 | -4.04 | NM_000975 | RPL11 | -4.03 |
| NM_012423 | RPL13A | -4.03 | NM_020127 | TUFT1 | -4.02 |
| NM_017584 | ALDRL6 | -4 | NM_000581 | GPX1 | -3.99 |
| NM_005262 | GFER | -3.99 | NM_198442 | FLJ45651 | -3.99 |
| NM_004763 | ITGB1BP1 | -3.98 | NM_021974 | POLR2F | -3.98 |
| XM_375912 | LOC400840 | -3.97 | NM_004122 | GHSR | -3.94 |
| NM_004315 | ASAH1 | -3.94 | NM_000788 | DCK | -3.93 |
| NM_032342 | C9ORF125 | -3.93 | NM_015925 | LISCH7 | -3.93 |
| NM_007165 | SF3A2 | -3.93 | NM_031217 | KIF18A | -3.91 |
| NM_003609 | HIRIP3 | -3.91 | NM_005489 | SH2D3C | -3.9 |

| | | | | | |
|---|---|---|---|---|---|
| NM_138347 | ZNF551 | -3.9 | NM_006107 | OA48-18 | -3.89 |
| NM_004502 | HOXB7 | -3.88 | NM_006246 | PPP2R5E | -3.87 |
| NM_018159 | NUDT11 | -3.87 | NM_025047 | FLJ22595 | -3.87 |
| XM_036729 | USP41 | -3.87 | XM_377760 | LOC402098 | -3.87 |
| NM_145699 | APOBEC3A | -3.86 | NM_198184 | OSTN | -3.85 |
| NM_003400 | XPO1 | -3.84 | XM_058073 | NUP205 | -3.82 |
| NM_019077 | UGT1A7 | -3.82 | XM_015717 | LOC149224 | -3.82 |
| XM_087089 | KIAA0007 | -3.81 | NM_000083 | CLCN1 | -3.8 |
| XM_012219 | LOC144483 | -3.8 | XM_379694 | LOC401577 | -3.8 |
| NM_003707 | RUVBL1 | -3.79 | NM_004643 | PABPN1 | -3.78 |
| NM_002954 | RPS27A | -3.78 | NM_004610 | TCP10 | -3.78 |
| NM_032306 | SPATA11 | -3.78 | NM_133459 | FLJ30681 | -3.78 |
| NM_018903 | PCDHA12 | -3.76 | NM_003466 | PAX8 | -3.76 |
| XM_375698 | LOC400745 | -3.76 | NM_019610 | KAT3 | -3.75 |
| NM_005471 | GNPDA1 | -3.74 | NM_002808 | PSMD2 | -3.72 |
| NM_000938 | POLR2B | -3.72 | NM_006493 | CLN5 | -3.72 |
| NM_005845 | ABCC4 | -3.72 | NM_133492 | ASAH3 | -3.72 |
| NM_000449 | RFX5 | -3.71 | NM_015360 | SKIV2L2 | -3.71 |
| NM_021980 | OPTN | -3.7 | NM_000972 | RPL7A | -3.7 |
| NM_006585 | CCT8 | -3.69 | XM_371644 | MUSTN1 | -3.69 |
| NM_182560 | FLJ25773 | -3.64 | NM_033405 | PRIC285 | -3.63 |
| NM_005151 | USP14 | -3.63 | NM_015560 | OPA1 | -3.63 |
| NM_002265 | KPNB1 | -3.63 | NM_004371 | COPA | -3.63 |
| NM_032023 | RASSF4 | -3.63 | NM_021192 | HOXD11 | -3.62 |
| NM_005650 | TCF20 | -3.62 | NM_001437 | ESR2 | -3.62 |
| NM_005131 | THOC1 | -3.61 | NM_000661 | RPL9 | -3.61 |
| NM_020382 | SET8 | -3.6 | XM_373313 | OR1J5 | -3.59 |
| NM_000973 | RPL8 | -3.59 | NM_015023 | WDTC1 | -3.59 |
| NM_004445 | EPHB6 | -3.58 | NM_006068 | TLR6 | -3.54 |
| NM_003465 | CHIT1 | -3.54 | NM_003380 | VIM | -3.54 |
| NM_005206 | CRK | -3.52 | NM_021126 | MPST | -3.52 |
| NM_000976 | RPL12 | -3.52 | NM_018079 | FLJ10379 | -3.52 |
| NM_018394 | FLJ11342 | -3.52 | XM_370865 | LOC388122 | -3.52 |
| XM_370910 | LOC388181 | -3.52 | NM_001007 | RPS4X | -3.5 |
| NM_017824 | 38412 | -3.5 | NM_001568 | EIF3S6 | -3.49 |
| NM_002594 | PCSK2 | -3.49 | NM_002066 | GML | -3.48 |
| NM_000997 | RPL37 | -3.48 | NM_004551 | NDUFS3 | -3.48 |
| NM_000967 | RPL3 | -3.48 | NM_004422 | DVL2 | -3.47 |
| NM_002382 | MAX | -3.46 | NM_001022 | RPS19 | -3.46 |
| NM_016204 | GDF2 | -3.44 | NM_198488 | FLJ46072 | -3.44 |
| NM_006469 | IVNS1ABP | -3.43 | NM_020382 | SET8 | -3.43 |
| NM_000986 | RPL24 | -3.43 | NM_014691 | AQR | -3.43 |
| NM_017842 | FLJ20489 | -3.43 | NM_032134 | DKFZP434P0316 | -3.43 |
| NM_002467 | MYC | -3.42 | NM_016041 | F-LANA | -3.42 |
| NM_002916 | RFC4 | -3.41 | NM_005954 | MT3 | -3.41 |
| NM_130807 | MOBKL2A | -3.41 | XM_378219 | LOC399737 | -3.41 |

| | | | | | |
|---|---|---|---|---|---|
| NM_012160 | FBXL4 | -3.39 | NM_004953 | EIF4G1 | -3.39 |
| NM_014886 | TINP1 | -3.38 | XM_373246 | LOC392208 | -3.38 |
| NM_001776 | ENTPD1 | -3.37 | NM_004076 | CRYBB3 | -3.37 |
| NM_152373 | MGC27466 | -3.37 | NM_014275 | MGAT4B | -3.36 |
| NM_016376 | ANKFY1 | -3.36 | XM_045911 | STXBP5L | -3.35 |
| NM_003920 | TIMELESS | -3.34 | NM_016476 | ANAPC11 | -3.34 |
| NM_018124 | RFWD3 | -3.34 | NM_016388 | TRIM | -3.33 |
| NM_007111 | TFDP1 | -3.33 | NM_004822 | NTN1 | -3.33 |
| NM_001415 | EIF2S3 | -3.33 | NM_001032 | RPS29 | -3.32 |
| NM_002807 | PSMD1 | -3.32 | NM_005066 | SFPQ | -3.31 |
| NM_016451 | COPB | -3.3 | NM_017768 | FLJ20331 | -3.29 |
| NM_024640 | FLJ23476 | -3.29 | NM_001005853 | OR6B2 | -3.29 |
| NM_002011 | FGFR4 | -3.28 | NM_147191 | MMP21 | -3.28 |
| NM_152832 | MTVR1 | -3.28 | NM_002358 | MAD2L1 | -3.28 |
| NM_001013 | RPS9 | -3.28 | NM_002987 | CCL17 | -3.27 |
| NM_013277 | RACGAP1 | -3.27 | NM_032444 | BTBD12 | -3.27 |
| XM_065899 | LOC130773 | -3.27 | NM_016633 | ERAF | -3.26 |
| XM_291729 | TAF3 | -3.26 | NM_002226 | JAG2 | -3.25 |
| NM_052865 | C20ORF72 | -3.25 | NM_152658 | THAP8 | -3.25 |
| NM_001686 | ATP5B | -3.23 | NM_002788 | PSMA3 | -3.23 |
| XM_378701 | LOC146795 | -3.23 | NM_198543 | MGC35434 | -3.23 |
| XM_376420 | LOC401206 | -3.23 | NM_015062 | PPRC1 | -3.22 |
| NM_144987 | U2AF1L3 | -3.22 | NM_000971 | RPL7 | -3.21 |
| NM_032786 | FLJ14451 | -3.21 | NM_194284 | CLDN23 | -3.21 |
| XM_371758 | LOC389308 | -3.21 | NM_002357 | MAD | -3.2 |
| NM_000493 | COL10A1 | -3.19 | XM_291204 | LOC340228 | -3.19 |
| NM_019059 | TOMM7 | -3.18 | NM_144974 | FLJ31846 | -3.18 |
| NM_006562 | LBX1 | -3.17 | NM_012452 | TNFRSF13B | -3.17 |
| NM_002940 | ABCE1 | -3.17 | NM_000977 | RPL13 | -3.17 |
| NM_153324 | DEFB123 | -3.16 | NM_001020 | RPS16 | -3.15 |
| XM_293352 | LOC347438 | -3.15 | NM_021620 | PRDM13 | -3.14 |
| NM_016218 | POLK | -3.14 | NM_020175 | LOC56931 | -3.14 |
| XM_291095 | LOC339970 | -3.14 | NM_013943 | CLIC4 | -3.13 |
| NM_014089 | NUPL1 | -3.13 | XM_057296 | LOC116064 | -3.13 |
| NM_001395 | DUSP9 | -3.12 | NM_001517 | GTF2H4 | -3.12 |
| NM_000993 | RPL31 | -3.12 | NM_003757 | EIF3S2 | -3.11 |
| NM_003752 | EIF3S8 | -3.11 | NM_005146 | SART1 | -3.11 |
| NM_032186 | KIAA1221 | -3.11 | NM_001001414 | LOC342897 | -3.11 |
| XM_373949 | LOC388885 | -3.11 | NM_006105 | RAPGEF3 | -3.1 |
| NM_016601 | KCNK9 | -3.1 | NM_016202 | ZNF580 | -3.1 |
| NM_020701 | KIAA1160 | -3.1 | NM_024790 | FLJ22490 | -3.1 |
| XM_041221 | LOC153027 | -3.1 | NM_173695 | FLJ36601 | -3.09 |
| XM_050625 | SFRP2 | -3.08 | NM_024943 | FLJ23235 | -3.08 |
| NM_203402 | LOC161247 | -3.07 | NM_001680 | FXYD2 | -3.06 |
| NM_003378 | VGF | -3.06 | NM_024859 | FLJ21687 | -3.06 |
| NM_139242 | MTFMT | -3.06 | XM_058332 | LOC118670 | -3.06 |

| | | | | | |
|---|---|---|---|---|---|
| NM_007262 | PARK7 | -3.04 | NM_014663 | JMJD2A | -3.04 |
| NM_012104 | BACE1 | -3.04 | NM_138780 | SYTL5 | -3.04 |
| NM_138451 | LOC115811 | -3.04 | NM_001511 | CXCL1 | -3.03 |
| NM_130467 | PAGE-5 | -3.03 | XM_379159 | LOC151484 | -3.03 |
| XM_378542 | LOC283761 | -3.03 | NM_007279 | U2AF2 | -3.02 |
| NM_014597 | ERBP | -3.02 | NM_080722 | ADAMTS14 | -3.01 |
| NM_007273 | REA | -3.01 | NM_020809 | ARHGAP20 | -3.01 |
| NM_006143 | GPR19 | -3 | NM_001005504 | OR4F21 | -3 |
| NM_018197 | ZFP64 | -2.99 | NM_001273 | CHD4 | -2.99 |
| NM_147180 | PPP3R2 | -2.99 | NM_015245 | ANKS1 | -2.99 |
| NM_014960 | KIAA1001 | -2.99 | NM_016047 | P14 | -2.99 |
| XM_104657 | LOC164714 | -2.99 | NM_005926 | MFAP1 | -2.98 |
| NM_015459 | DKFZP564J0863 | -2.98 | NM_018040 | GPATC2 | -2.98 |
| NM_025138 | C13ORF23 | -2.98 | NM_018406 | MUC4 | -2.97 |
| NM_033255 | EPSTI1 | -2.97 | NM_015443 | LOC284058 | -2.97 |
| NM_002796 | PSMB4 | -2.96 | NM_004708 | PDCD5 | -2.96 |
| NM_001088 | AANAT | -2.96 | NM_002694 | POLR2C | -2.96 |
| XM_035527 | FLJ10980 | -2.96 | XM_377926 | LOC402245 | -2.96 |
| NM_003870 | IQGAP1 | -2.95 | NM_000996 | RPL35A | -2.95 |
| NM_021915 | ZNF69 | -2.95 | NM_018942 | HMX1 | -2.94 |
| XM_371210 | TAS1R3 | -2.94 | NM_006150 | LMO6 | -2.93 |
| NM_024604 | FLJ21908 | -2.93 | XM_212162 | LOC286080 | -2.93 |
| NM_021953 | FOXM1 | -2.92 | NM_006666 | RUVBL2 | -2.92 |
| NM_002309 | LIF | -2.92 | NM_001634 | AMD1 | -2.92 |
| NM_152232 | TAS1R2 | -2.91 | NM_003872 | NRP2 | -2.91 |
| NM_003986 | BBOX1 | -2.91 | NM_014918 | CHSY1 | -2.91 |
| NM_031213 | C19ORF27 | -2.91 | NM_173645 | FLJ37357 | -2.91 |
| XM_375261 | LOC400452 | -2.91 | NM_005607 | PTK2 | -2.9 |
| NM_004631 | LRP8 | -2.9 | NM_207311 | LOC92558 | -2.9 |
| NM_181621 | KRTAP13-2 | -2.9 | NM_004490 | GRB14 | -2.89 |
| NM_000281 | PCBD | -2.89 | NM_001031 | RPS28 | -2.89 |
| NM_013453 | SPANXA1 | -2.89 | NM_020129 | LGALS14 | -2.89 |
| NM_000160 | GCGR | -2.88 | NM_004657 | SDPR | -2.88 |
| NM_000947 | PRIM2A | -2.88 | NM_001378 | DNCI2 | -2.88 |
| NM_000979 | RPL18 | -2.88 | NM_144976 | ZNF564 | -2.88 |
| NM_178499 | MGC39827 | -2.88 | XM_370684 | LOC387845 | -2.88 |
| NM_144667 | FLJ32894 | -2.87 | XM_375654 | FLJ35258 | -2.87 |
| NM_033387 | C9ORF59 | -2.87 | NM_014301 | ISCU | -2.86 |
| NM_145045 | MGC20983 | -2.86 | NM_004843 | IL27RA | -2.85 |
| NM_005582 | LY64 | -2.85 | NM_032758 | PHF5A | -2.85 |
| NM_201401 | FLJ26056 | -2.85 | NM_002798 | PSMB6 | -2.84 |
| NM_006082 | K-ALPHA-1 | -2.84 | XM_376365 | LOC116349 | -2.84 |
| NM_000274 | OAT | -2.83 | NM_024302 | MMP28 | -2.83 |
| NM_001523 | HAS1 | -2.83 | NM_017438 | C21ORF18 | -2.83 |
| NM_022819 | PLA2G2F | -2.83 | XM_371222 | LOC388595 | -2.83 |
| XM_374179 | LOC389422 | -2.83 | NM_013328 | PYCR2 | -2.82 |

| | | |
|---|---|---|
| NM_001001415 | ZNF429 | -2.82 |
| NM_002815 | PSMD11 | -2.81 |
| XM_378199 | FLJ10232 | -2.81 |
| NM_000987 | RPL26 | -2.8 |
| NM_152506 | C21ORF129 | -2.8 |
| NM_016290 | RAP80 | -2.79 |
| NM_018933 | PCDHB13 | -2.79 |
| NM_015412 | DKFZP434F2021 | -2.78 |
| NM_052946 | NOSTRIN | -2.78 |
| NM_003858 | CCNK | -2.77 |
| NM_022737 | LPPR2 | -2.77 |
| XM_374987 | LOC400055 | -2.77 |
| NM_017857 | SSH3 | -2.76 |
| NM_178449 | TIP39 | -2.76 |
| NM_020718 | USP31 | -2.75 |
| NM_001760 | CCND3 | -2.75 |
| NM_032837 | FLJ14775 | -2.75 |
| NM_014911 | AAK1 | -2.74 |
| XM_376720 | KIAA0543 | -2.74 |
| XM_085833 | LOC147646 | -2.74 |
| NM_005916 | MCM7 | -2.73 |
| NM_198468 | C6ORF167 | -2.73 |
| XM_372926 | LOC391370 | -2.73 |
| NM_004730 | ETF1 | -2.72 |
| NM_022090 | LOC63920 | -2.72 |
| NM_012115 | CASP8AP2 | -2.71 |
| NM_001003 | RPLP1 | -2.71 |
| XM_095122 | LOC168474 | -2.71 |
| NM_182702 | TESSP2 | -2.71 |
| XM_378693 | LOC400614 | -2.71 |
| NM_001005489 | OR5B17 | -2.71 |
| NM_003195 | TCEA2 | -2.7 |
| NM_000995 | RPL34 | -2.7 |
| XM_380042 | LOC402694 | -2.7 |
| NM_031966 | CCNB1 | -2.69 |
| NM_022074 | FLJ22794 | -2.69 |
| NM_052849 | MGC20481 | -2.69 |
| NM_138338 | POLR3H | -2.69 |
| NM_002561 | P2RX5 | -2.68 |
| NM_003091 | SNRPB | -2.67 |
| XM_051221 | SKIP | -2.67 |
| XM_379877 | LOC402556 | -2.67 |
| NM_003302 | TRIP6 | -2.66 |
| NM_032314 | MGC4767 | -2.66 |
| NM_005153 | USP10 | -2.65 |
| NM_016304 | C15ORF15 | -2.65 |
| NM_012152 | EDG7 | -2.81 |
| NM_016001 | CGI-48 | -2.81 |
| NM_001821 | CHML | -2.8 |
| NM_012426 | SF3B3 | -2.8 |
| NM_174977 | SEC14L4 | -2.8 |
| NM_016541 | GNG13 | -2.79 |
| NM_000990 | RPL27A | -2.78 |
| NM_032352 | BRMS1L | -2.78 |
| NM_006827 | TMP21 | -2.77 |
| NM_022567 | NYX | -2.77 |
| NM_006210 | PEG3 | -2.77 |
| NM_004358 | CDC25B | -2.76 |
| XM_291345 | KIAA0522 | -2.76 |
| XM_114152 | LOC200205 | -2.76 |
| NM_001255 | CDC20 | -2.75 |
| NM_002789 | PSMA4 | -2.75 |
| XM_039218 | LOC91561 | -2.75 |
| NM_006098 | GNB2L1 | -2.74 |
| XM_372884 | CECR2 | -2.74 |
| NM_152553 | IBRDC1 | -2.74 |
| NM_004335 | BST2 | -2.73 |
| XM_370995 | SNAI3 | -2.73 |
| NM_000945 | PPP3R1 | -2.72 |
| NM_000970 | RPL6 | -2.72 |
| NM_000359 | TGM1 | -2.71 |
| NM_001114 | ADCY7 | -2.71 |
| NM_173532 | FLJ35838 | -2.71 |
| NM_198822 | ATP5L2 | -2.71 |
| XM_370723 | LOC387922 | -2.71 |
| XM_377041 | LOC401618 | -2.71 |
| NM_004472 | FOXD1 | -2.7 |
| NM_002522 | NPTX1 | -2.7 |
| XM_290345 | LOC339799 | -2.7 |
| XM_376869 | LOC401525 | -2.7 |
| NM_022352 | CARD9 | -2.69 |
| XM_042936 | GRIP2 | -2.69 |
| NM_138457 | FOXP4 | -2.69 |
| NM_133259 | LRPPRC | -2.68 |
| NM_012223 | MYO1B | -2.68 |
| NM_024322 | MGC11266 | -2.67 |
| XM_372803 | LOC391130 | -2.67 |
| NM_002092 | GRSF1 | -2.66 |
| NM_007071 | HHLA3 | -2.66 |
| NM_005248 | FGR | -2.65 |
| NM_003547 | HIST1H4G | -2.65 |
| NM_004815 | PARG1 | -2.64 |

| | | | | | |
|---|---|---|---|---|---|
| NM_022752 | ZNF574 | -2.64 | NM_030786 | SYNCOILIN | -2.64 |
| XM_372039 | LOC389651 | -2.64 | XM_374339 | LOC389896 | -2.64 |
| NM_001005521 | C21ORF37 | -2.64 | NM_058229 | FBXO32 | -2.63 |
| NM_004270 | CRSP9 | -2.63 | NM_002795 | PSMB3 | -2.63 |
| NM_000968 | RPL4 | -2.63 | NM_183375 | ESSPL | -2.63 |
| NM_012321 | LSM4 | -2.62 | XM_378705 | LOC400620 | -2.62 |
| NM_006260 | DNAJC3 | -2.61 | XM_048104 | FLG | -2.61 |
| NM_006932 | SMTN | -2.61 | NM_003750 | EIF3S10 | -2.61 |
| NM_000985 | RPL17 | -2.61 | NM_000988 | RPL27 | -2.61 |
| NM_022086 | ELMO2 | -2.61 | NM_000224 | KRT18 | -2.6 |
| NM_024813 | FLJ13150 | -2.6 | NM_032595 | PPP1R9B | -2.59 |
| NM_000046 | ARSB | -2.59 | NM_001249 | ENTPD5 | -2.59 |
| NM_014293 | NPTXR | -2.59 | NM_007341 | SH3BGR | -2.59 |
| NM_014904 | RAB11-FIP2 | -2.59 | NM_018085 | IPO9 | -2.59 |
| NM_182532 | LOC199964 | -2.59 | NM_182554 | C10ORF53 | -2.59 |
| NM_024647 | NUP43 | -2.59 | NM_000569 | FCGR3A | -2.58 |
| NM_003457 | ZNF207 | -2.58 | NM_001003938 | HBM | -2.58 |
| NM_002810 | PSMD4 | -2.57 | NM_004772 | C5ORF13 | -2.57 |
| NM_014481 | APEX2 | -2.57 | NM_018988 | GFOD1 | -2.57 |
| NM_207438 | FLJ43808 | -2.57 | NM_002486 | NCBP1 | -2.56 |
| NM_007374 | SIX6 | -2.56 | NM_024292 | UBL5 | -2.56 |
| NM_000131 | F7 | -2.56 | NM_014014 | U5-200KD | -2.56 |
| NM_002816 | PSMD12 | -2.56 | NM_014143 | PDCD1LG1 | -2.56 |
| NM_032026 | TATDN1 | -2.56 | NM_058189 | C21ORF69 | -2.56 |
| NM_152518 | FLJ38359 | -2.56 | NM_178429 | LEP11 | -2.56 |
| XM_378412 | LOC400125 | -2.56 | NM_213655 | HSN2 | -2.56 |
| NM_007170 | TESK2 | -2.55 | NM_001507 | MLNR | -2.55 |
| NM_004941 | DHX8 | -2.55 | NM_013396 | USP25 | -2.55 |
| NM_007346 | OGFR | -2.55 | NM_153363 | MGC42415 | -2.55 |
| NM_007223 | GPR | -2.54 | XM_050278 | KIF26A | -2.54 |
| NM_001194 | HCN2 | -2.54 | NM_000989 | RPL30 | -2.54 |
| NM_020196 | XAB2 | -2.54 | NM_021177 | LSM2 | -2.54 |
| NM_032133 | MYCBPAP | -2.54 | NM_152324 | MGC35169 | -2.54 |
| NM_182535 | LOC200261 | -2.54 | XM_379987 | LOC402634 | -2.54 |
| NM_002082 | GRK6 | -2.53 | NM_016337 | EVL | -2.53 |
| NM_018663 | PXMP2 | -2.53 | NM_013304 | ZDHHC1 | -2.53 |
| NM_004090 | DUSP3 | -2.52 | NM_001287 | CLCN7 | -2.52 |
| NM_000136 | FANCC | -2.52 | NM_000980 | RPL18A | -2.52 |
| NM_145308 | LOC220070 | -2.52 | NM_003114 | SPAG1 | -2.51 |
| NM_007130 | ZNF41 | -2.51 | NM_144691 | CAPN12 | -2.51 |
| NM_181619 | KRTAP21-1 | -2.51 | NM_182972 | IRF2BP2 | -2.51 |
| NM_001043 | SLC6A2 | -2.5 | NM_020226 | PRDM8 | -2.5 |
| NM_001089 | ABCA3 | -2.5 | NM_000984 | RPL23A | -2.5 |
| NM_006598 | SLC12A7 | -2.5 | NM_014333 | IGSF4 | -2.5 |
| NM_053044 | HTRA3 | -2.5 | XM_209941 | C9ORF117 | -2.5 |
| XM_372749 | LOC390975 | -2.5 | XM_379855 | LOC402530 | -2.5 |

| | | | | | |
|---|---|---|---|---|---|
| NM_020370 | GPR84 | -2.49 | NM_030905 | OR2J2 | -2.49 |
| NM_003880 | WISP3 | -2.49 | NM_021955 | GNGT1 | -2.49 |
| NM_002809 | PSMD3 | -2.49 | NM_145253 | LOC124402 | -2.49 |
| NM_130776 | GAGED4 | -2.49 | NM_000981 | RPL19 | -2.48 |
| NM_014050 | MRPL42 | -2.48 | NM_014138 | TMEM29 | -2.48 |
| NM_017563 | IL17RD | -2.48 | NM_017509 | KLK15 | -2.48 |
| NM_022780 | FLJ13910 | -2.48 | NM_152358 | MGC33947 | -2.48 |
| NM_207353 | LOC286480 | -2.48 | XM_048462 | RUSC2 | -2.47 |
| NM_031894 | FTHL17 | -2.47 | NM_018469 | HT008 | -2.47 |
| XM_379073 | LOC386597 | -2.47 | XM_376554 | LOC401285 | -2.47 |
| NM_004597 | SNRPD2 | -2.46 | NM_020131 | C1ORF6 | -2.46 |
| NM_139126 | PPIL4 | -2.46 | NM_002111 | HD | -2.45 |
| NM_015934 | NOP5/NOP58 | -2.45 | NM_145172 | NYD-SP29 | -2.45 |
| NM_002091 | GRP | -2.44 | NM_018089 | FLJ10415 | -2.44 |
| NM_152586 | USP54 | -2.44 | XM_372160 | LOC389814 | -2.44 |
| NM_015112 | MAST2 | -2.43 | NM_021232 | PRODH2 | -2.43 |
| NM_000631 | NCF4 | -2.43 | NM_012465 | TLL2 | -2.43 |
| XM_029101 | KIAA0947 | -2.43 | NM_017793 | RPP25 | -2.43 |
| NM_024109 | MGC2654 | -2.43 | NM_032582 | USP32 | -2.43 |
| XM_374138 | LOC389329 | -2.43 | XM_371204 | LOC388574 | -2.43 |
| NM_005274 | GNG5 | -2.42 | NM_001608 | ACADL | -2.42 |
| NM_133639 | RHOV | -2.42 | NM_003818 | CDS2 | -2.42 |
| NM_153247 | SLC29A4 | -2.42 | NM_001716 | BLR1 | -2.41 |
| NM_003941 | WASL | -2.41 | NM_014569 | ZFP95 | -2.41 |
| NM_022462 | HIF3A | -2.41 | NM_021228 | SR-A1 | -2.41 |
| NM_144658 | DOCK11 | -2.41 | NM_152899 | IL4I1 | -2.41 |
| NM_000024 | ADRB2 | -2.4 | NM_000028 | AGL | -2.4 |
| XM_371573 | NXPH2 | -2.4 | NM_024097 | MGC955 | -2.4 |
| NM_024675 | FLJ21816 | -2.4 | NM_144631 | ZNF513 | -2.4 |
| XM_373004 | LOC391594 | -2.4 | NM_001619 | ADRBK1 | -2.39 |
| NM_000168 | GLI3 | -2.39 | NM_020979 | APS | -2.39 |
| NM_001002 | RPLP0 | -2.39 | NM_014347 | ZNF324 | -2.39 |
| NM_018082 | POLR3B | -2.39 | NM_022119 | PRSS22 | -2.39 |
| NM_181604 | KRTAP6-2 | -2.39 | XM_374279 | LOC389685 | -2.39 |
| XM_293449 | LOC389915 | -2.39 | NM_004759 | MAPKAPK2 | -2.38 |
| NM_001934 | DLX4 | -2.38 | NM_000999 | RPL38 | -2.38 |
| NM_004809 | STOML1 | -2.38 | NM_024804 | FLJ12606 | -2.38 |
| NM_025234 | REC14 | -2.38 | XM_376679 | FLJ25778 | -2.38 |
| NM_005105 | RBM8A | -2.37 | NM_024111 | MGC4504 | -2.37 |
| XM_378340 | LOC400004 | -2.37 | NM_001892 | CSNK1A1 | -2.36 |
| NM_007191 | WIF1 | -2.36 | NM_017655 | GIPC2 | -2.36 |
| NM_004254 | SLC22A8 | -2.36 | NM_004213 | SLC28A1 | -2.36 |
| NM_005822 | DSCR1L1 | -2.36 | NM_147128 | ZNRF2 | -2.36 |
| XM_291671 | RBM20 | -2.36 | XM_371936 | LOC389538 | -2.36 |
| NM_001004053 | ACTBL1 | -2.36 | NM_213606 | LOC387700 | -2.36 |
| NM_001005501 | OR4K2 | -2.36 | NM_017886 | FLJ20574 | -2.35 |

| | | |
|---|---|---|
| NM_000609 | CXCL12 | -2.35 |
| NM_006065 | SIRPB1 | -2.35 |
| NM_031273 | TEX13B | -2.35 |
| XM_375000 | KIAA1853 | -2.35 |
| NM_004547 | NDUFB4 | -2.35 |
| NM_015442 | CNOT10 | -2.34 |
| NM_022452 | FBS1 | -2.34 |
| NM_000141 | FGFR2 | -2.33 |
| NM_004516 | ILF3 | -2.33 |
| NM_002395 | ME1 | -2.33 |
| NM_002676 | PMM1 | -2.33 |
| NM_080723 | VMP | -2.33 |
| XM_291974 | LOC338750 | -2.33 |
| XM_371677 | LOC389173 | -2.33 |
| NM_006230 | POLD2 | -2.32 |
| NM_182556 | LOC283130 | -2.32 |
| XM_372562 | LOC390564 | -2.32 |
| NM_002068 | GNA15 | -2.31 |
| NM_024123 | LY6G6E | -2.31 |
| NM_182487 | OLFML2A | -2.31 |
| NM_006335 | TIMM17A | -2.3 |
| NM_018193 | FLJ10719 | -2.3 |
| NM_001001716 | NFKBIB | -2.29 |
| NM_130786 | A1BG | -2.29 |
| XM_376830 | KIAA0258 | -2.29 |
| NM_020414 | DDX24 | -2.29 |
| XM_172230 | LOC257039 | -2.29 |
| NM_018249 | CDK5RAP2 | -2.28 |
| NM_003840 | TNFRSF10D | -2.28 |
| NM_001683 | ATP2B2 | -2.28 |
| NM_023013 | LOC65121 | -2.28 |
| NM_152748 | FLJ31340 | -2.28 |
| NM_020761 | Raptor | -2.27 |
| NM_016480 | PAIP2 | -2.27 |
| XM_377818 | LOC402148 | -2.27 |
| NM_007294 | BRCA1 | -2.26 |
| NM_016284 | KIAA1007 | -2.26 |
| NM_203458 | N2N | -2.26 |
| XM_379839 | LOC402508 | -2.26 |
| NM_032511 | C6ORF168 | -2.25 |
| NM_018953 | HOXC5 | -2.24 |
| NM_017668 | NDE1 | -2.24 |
| NM_032370 | MGC15716 | -2.24 |
| XM_116936 | LOC196541 | -2.24 |
| NM_000428 | LTBP2 | -2.23 |
| NM_002481 | PPP1R12B | -2.23 |
| NM_001206 | BTEB1 | -2.35 |
| NM_020210 | SEMA4B | -2.35 |
| NM_031954 | KCTD10 | -2.35 |
| XM_379717 | LOC401607 | -2.35 |
| NM_001498 | GCLC | -2.34 |
| NM_019057 | FLJ10404 | -2.34 |
| NM_031452 | C6ORF119 | -2.34 |
| XM_290796 | KIAA1361 | -2.33 |
| NM_001488 | TADA2L | -2.33 |
| NM_000948 | PRL | -2.33 |
| NM_000982 | RPL21 | -2.33 |
| NM_139278 | LGI3 | -2.33 |
| XM_372768 | LOC391012 | -2.33 |
| NM_004299 | ABCB7 | -2.32 |
| XM_059729 | IRAK1BP1 | -2.32 |
| XM_372535 | LOC390508 | -2.32 |
| NM_013314 | BLNK | -2.31 |
| NM_003751 | EIF3S9 | -2.31 |
| NM_153236 | HIAN7 | -2.31 |
| XM_372448 | LOC390284 | -2.31 |
| NM_021149 | COTL1 | -2.3 |
| NM_021135 | RPS6KA2 | -2.29 |
| NM_012108 | BRDG1 | -2.29 |
| NM_005672 | PSCA | -2.29 |
| NM_016016 | CGI-69 | -2.29 |
| NM_152737 | MGC33993 | -2.29 |
| NM_003301 | TRHR | -2.28 |
| NM_015040 | PIP5K3 | -2.28 |
| NM_003353 | UCN | -2.28 |
| NM_018097 | FLJ10460 | -2.28 |
| NM_145309 | LOC220074 | -2.28 |
| NM_007160 | OR2H3 | -2.27 |
| NM_007080 | LSM6 | -2.27 |
| NM_153350 | FBXL16 | -2.27 |
| NM_001280 | CIRBP | -2.26 |
| NM_003945 | ATP6V0E | -2.26 |
| NM_014286 | FREQ | -2.26 |
| XM_373099 | LOC391847 | -2.26 |
| NM_005765 | ATP6AP2 | -2.25 |
| NM_175892 | FLJ37266 | -2.25 |
| NM_016213 | TRIP4 | -2.24 |
| NM_014482 | BMP10 | -2.24 |
| NM_032761 | MGC16075 | -2.24 |
| NM_003508 | FZD9 | -2.23 |
| NM_006086 | TUBB4 | -2.23 |
| NM_182710 | HTATIP | -2.23 |

| | | | | | |
|---|---|---|---|---|---|
| NM_000803 | FOLR2 | -2.23 | NM_002793 | PSMB1 | -2.23 |
| NM_017929 | PEX26 | -2.23 | NM_024721 | ZFH4 | -2.23 |
| NM_031934 | RAB34 | -2.23 | NM_178125 | TRIM50A | -2.23 |
| XM_378517 | MGC15885 | -2.23 | XM_375308 | LOC400509 | -2.23 |
| NM_000024 | ADRB2 | -2.22 | NM_006773 | DDX18 | -2.22 |
| NM_021002 | IFNA6 | -2.22 | NM_006076 | HRBL | -2.22 |
| NM_197964 | HSPC268 | -2.22 | NM_198504 | PAQR9 | -2.22 |
| XM_371778 | LOC389341 | -2.22 | NM_005823 | MSLN | -2.21 |
| NM_004127 | GPS1 | -2.21 | NM_000854 | GSTT2 | -2.21 |
| NM_002033 | FUT4 | -2.21 | NM_022551 | RPS18 | -2.21 |
| NM_032379 | SYTL2 | -2.21 | NM_052864 | T2BP | -2.21 |
| NM_144711 | MGC22679 | -2.21 | NM_002044 | GALK2 | -2.2 |
| NM_021074 | NDUFV2 | -2.2 | NM_014444 | 76P | -2.2 |
| NM_021943 | TEX27 | -2.2 | NM_153603 | COG7 | -2.2 |
| XM_380012 | LOC402670 | -2.2 | NM_012127 | CIZ1 | -2.19 |
| NM_030936 | RNF32 | -2.19 | NM_020070 | IGLL1 | -2.19 |
| NM_016593 | CYP39A1 | -2.19 | XM_016113 | LOC158104 | -2.19 |
| XM_377885 | LOC402208 | -2.19 | NM_004817 | TJP2 | -2.18 |
| NM_000576 | IL1B | -2.18 | NM_016536 | ZNF571 | -2.18 |
| NM_015980 | HMP19 | -2.18 | NM_022072 | FLJ22609 | -2.18 |
| NM_198563 | MGC52022 | -2.18 | XM_378553 | LOC400475 | -2.18 |
| NM_017986 | FLJ10060 | -2.17 | NM_003608 | GPR65 | -2.17 |
| NM_012369 | OR2F1 | -2.17 | NM_020137 | GRIPAP1 | -2.17 |
| NM_005244 | EYA2 | -2.17 | NM_015904 | EIF5B | -2.17 |
| NM_020904 | PLEKHA4 | -2.17 | XM_378390 | LOC144678 | -2.17 |
| XM_373539 | LOC387871 | -2.17 | NM_016059 | PPIL1 | -2.16 |
| NM_004435 | ENDOG | -2.16 | NM_006444 | SMC2L1 | -2.16 |
| NM_015004 | EXOSC7 | -2.16 | NM_002139 | RBMX | -2.16 |
| NM_016472 | C14ORF129 | -2.16 | NM_025072 | PTGES2 | -2.16 |
| NM_194282 | DKFZP686L1814 | -2.16 | NM_207472 | FLJ46020 | -2.16 |
| XM_292029 | LOC341371 | -2.16 | XM_379317 | LOC401175 | -2.16 |
| XM_378239 | LOC399789 | -2.16 | NM_012146 | DUX1 | -2.15 |
| NM_002466 | MYBL2 | -2.15 | NM_021198 | CTDSP1 | -2.15 |
| NM_020799 | AMSH-LP | -2.15 | NM_004691 | ATP6V0D1 | -2.15 |
| NM_015864 | C6ORF32 | -2.15 | NM_014023 | WDR37 | -2.15 |
| NM_206909 | EFA6R | -2.15 | NM_002689 | POLA2 | -2.15 |
| NM_013234 | EIF3K | -2.15 | NM_016132 | MYEF2 | -2.15 |
| NM_030782 | CRR9 | -2.15 | XM_293886 | LOC345576 | -2.15 |
| NM_001002036 | ASTL | -2.15 | NM_002982 | CCL2 | -2.14 |
| NM_018314 | UEV3 | -2.14 | NM_152291 | MUC7 | -2.14 |
| NM_005088 | DXYS155E | -2.14 | NM_012121 | CDC42EP4 | -2.14 |
| NM_024048 | MGC3020 | -2.14 | NM_024085 | FLJ22169 | -2.14 |
| NM_173525 | MGC34805 | -2.14 | NM_153032 | FLJ32065 | -2.14 |
| NM_173586 | MGC34821 | -2.14 | NM_181538 | GJE1 | -2.14 |
| NM_003088 | FSCN1 | -2.13 | NM_001011 | RPS7 | -2.13 |
| NM_016652 | CRNKL1 | -2.13 | NM_032478 | MRPL38 | -2.13 |

| | | | | | |
|---|---|---|---|---|---|
| NM_032892 | MGC14161 | -2.13 | NM_173605 | KCNRG | -2.13 |
| NM_002766 | PRPSAP1 | -2.12 | NM_006662 | SRCAP | -2.12 |
| XM_071866 | CDR2 | -2.12 | NM_012408 | PRKCBP1 | -2.12 |
| NM_002805 | PSMC5 | -2.12 | NM_016605 | C5ORF6 | -2.12 |
| NM_032303 | C9ORF99 | -2.12 | NM_138796 | LOC128153 | -2.12 |
| XM_292957 | LOC344165 | -2.12 | NM_000124 | ERCC6 | -2.11 |
| NM_014737 | RASSF2 | -2.11 | NM_007000 | UPK1A | -2.11 |
| NM_007256 | SLCO2B1 | -2.11 | NM_015138 | KIAA0252 | -2.11 |
| NM_012269 | HYAL4 | -2.11 | NM_018101 | CDCA8 | -2.11 |
| NM_020927 | KIAA1576 | -2.11 | NM_020905 | RDH14 | -2.11 |
| NM_024610 | HSPBAP1 | -2.11 | NM_032634 | PIGO | -2.11 |
| XM_043492 | KIAA1728 | -2.11 | XM_379632 | LOC158376 | -2.11 |
| XM_372092 | LOC389727 | -2.11 | XM_374338 | LOC389893 | -2.11 |
| NM_005480 | TROAP | -2.1 | NM_004404 | NEDD5 | -2.1 |
| NM_006427 | SIVA | -2.1 | NM_000836 | GRIN2D | -2.1 |
| NM_000272 | NPHP1 | -2.1 | NM_005195 | CEBPD | -2.1 |
| NM_002631 | PGD | -2.1 | NM_002806 | PSMC6 | -2.1 |
| NM_031920 | ARG99 | -2.1 | XM_086894 | LOC150297 | -2.1 |
| NM_207329 | MYADML | -2.1 | NM_173562 | C6ORF69 | -2.1 |
| NM_199285 | MGC70924 | -2.1 | NM_020529 | NFKBIA | -2.09 |
| NM_003498 | SNN | -2.09 | NM_001695 | ATP6V1C1 | -2.09 |
| NM_004053 | BYSL | -2.09 | NM_033102 | PROSTEIN | -2.09 |
| XM_378825 | LOC400730 | -2.09 | NM_003559 | PIP5K2B | -2.08 |
| NM_003913 | PRPF4B | -2.08 | NM_003011 | SET | -2.08 |
| NM_004165 | RRAD | -2.08 | NM_001353 | AKR1C1 | -2.08 |
| NM_002669 | PLRG1 | -2.08 | NM_014517 | UBP1 | -2.08 |
| NM_015235 | CSTF2T | -2.08 | NM_024688 | C10ORF68 | -2.08 |
| NM_182756 | SPY1 | -2.08 | NM_001619 | ADRBK1 | -2.07 |
| NM_007186 | CEP2 | -2.07 | NM_020672 | S100A14 | -2.07 |
| NM_001902 | CTH | -2.07 | NM_004623 | TTC4 | -2.07 |
| NM_007272 | CTRC | -2.07 | NM_024954 | UBTD1 | -2.07 |
| NM_080616 | C20ORF112 | -2.07 | NM_182517 | MGC52423 | -2.07 |
| XM_211090 | LOC283587 | -2.07 | XM_375670 | LOC400726 | -2.07 |
| NM_000686 | AGTR2 | -2.06 | NM_004417 | DUSP1 | -2.06 |
| NM_003933 | BAIAP3 | -2.06 | NM_006802 | SF3A3 | -2.06 |
| NM_012470 | TNPO3 | -2.06 | NM_014402 | QP-C | -2.06 |
| NM_199265 | THSD3 | -2.06 | XM_375298 | KIAA1987 | -2.06 |
| NM_207392 | UNQ467 | -2.06 | XM_373809 | LOC388553 | -2.06 |
| NM_212557 | UNQ689 | -2.06 | NM_006549 | CAMKK2 | -2.05 |
| NM_013276 | CARKL | -2.05 | NM_003950 | F2RL3 | -2.05 |
| NM_005227 | EFNA4 | -2.05 | NM_005594 | NACA | -2.05 |
| NM_001029 | RPS26 | -2.05 | XM_371175 | ZNF229 | -2.05 |
| NM_018409 | DKFZP761O0113 | -2.05 | NM_022465 | ZNFN1A4 | -2.05 |
| NM_024631 | FLJ23342 | -2.05 | NM_033182 | FBXO44 | -2.05 |
| NM_173593 | B4GALNAC-T3 | -2.05 | NM_181617 | KRTAP21-2 | -2.05 |
| NM_178839 | LRRTM1 | -2.05 | NM_198698 | KRTAP12-4 | -2.05 |

| | | | | | |
|---|---|---|---|---|---|
| XM_378044 | LOC402354 | -2.05 | XM_378608 | LOC400541 | -2.05 |
| NM_153809 | TAF1L | -2.04 | XM_166527 | KIAA0415 | -2.04 |
| NM_022753 | FLJ12903 | -2.04 | XM_290822 | LOC284367 | -2.04 |
| NM_005334 | HCFC1 | -2.03 | NM_004366 | CLCN2 | -2.03 |
| NM_014270 | SLC7A9 | -2.03 | XM_371877 | KIAA0960 | -2.03 |
| NM_004595 | SMS | -2.03 | NM_001004 | RPLP2 | -2.03 |
| NM_007169 | PEMT | -2.03 | XM_034872 | 39692 | -2.03 |
| NM_207317 | FLJ32921 | -2.03 | XM_040149 | LOC151273 | -2.03 |
| XM_292468 | LOC342293 | -2.03 | NM_001001411 | LOC163223 | -2.03 |
| NM_000324 | RHAG | -2.02 | NM_005918 | MDH2 | -2.02 |
| NM_006938 | SNRPD1 | -2.02 | NM_014965 | OIP106 | -2.02 |
| NM_018684 | HCA127 | -2.02 | NM_022130 | GOLPH3 | -2.02 |
| NM_138454 | LOC115861 | -2.02 | NM_152721 | DOK5L | -2.02 |
| NM_017567 | NAGK | -2.01 | NM_023947 | MGC3234 | -2.01 |
| NM_006231 | POLE | -2.01 | NM_006421 | BIG1 | -2.01 |
| NM_002991 | CCL24 | -2.01 | NM_005209 | CRYBA2 | -2.01 |
| NM_001649 | APXL | -2.01 | NM_001015 | RPS11 | -2.01 |
| NM_005786 | SDCCAG33 | -2.01 | NM_078628 | MSL3L1 | -2.01 |
| NM_007086 | WDHD1 | -2.01 | NM_017894 | ZFP29 | -2.01 |
| NM_018126 | TMEM33 | -2.01 | NM_018373 | SYNJ2BP | -2.01 |
| NM_024829 | FLJ22662 | -2.01 | NM_183374 | CYP26C1 | -2.01 |
| NM_198995 | C18ORF34 | -2.01 | XM_291200 | LOC340221 | -2.01 |
| NM_003503 | CDC7 | -2 | NM_004814 | HPRP8BP | -2 |
| NM_138477 | CDAN1 | -2 | NM_024877 | FLJ13265 | -2 |
| NM_032014 | MRPS24 | -2 | NM_030652 | EGFL8 | -2 |
| XM_047462 | SPIRE2 | -2 | NM_178556 | FLJ36180 | -2 |
| NM_194313 | C9ORF48 | -2 | NM_207394 | FLJ45949 | -2 |
| XM_376416 | LOC401204 | -2 | XM_372050 | LOC389674 | -2 |
| NM_145059 | FUK | -1.99 | NM_005922 | MAP3K4 | -1.99 |
| NM_005458 | GPR51 | -1.99 | NM_031283 | TCF7L1 | -1.99 |
| NM_000187 | HGD | -1.99 | NM_139057 | ADAMTS17 | -1.99 |
| NM_015247 | CYLD | -1.99 | NM_002389 | MCP | -1.99 |
| NM_005733 | KIF20A | -1.99 | NM_005124 | NUP153 | -1.99 |
| NM_001112 | ADARB1 | -1.99 | NM_016519 | AMBN | -1.99 |
| NM_018115 | SDAD1 | -1.99 | NM_022149 | MAGEF1 | -1.99 |
| NM_197954 | CLECSF12 | -1.99 | NM_032639 | FAPP2 | -1.99 |
| NM_031909 | C1QTNF4 | -1.99 | NM_152633 | FLJ34064 | -1.99 |
| NM_032958 | POLR2J2 | -1.99 | XM_084845 | LOC144383 | -1.99 |
| XM_166203 | LOC219537 | -1.99 | NM_004420 | DUSP8 | -1.98 |
| NM_004196 | CDKL1 | -1.98 | NM_000180 | GUCY2D | -1.98 |
| NM_031272 | TEX14 | -1.98 | NM_153839 | GPR111 | -1.98 |
| NM_004450 | ERH | -1.98 | NM_005177 | ATP6V0A1 | -1.98 |
| NM_006503 | PSMC4 | -1.98 | XM_370737 | FLJ10357 | -1.98 |
| NM_018929 | PCDHGC5 | -1.98 | NM_030816 | DKFZP566D1346 | -1.98 |
| NM_032425 | KIAA1822 | -1.98 | NM_032916 | MGC16279 | -1.98 |
| XM_166432 | LOC221442 | -1.98 | XM_379123 | LOC400999 | -1.98 |

| | | | | | |
|---|---|---|---|---|---|
| XM_379736 | LOC401628 | -1.98 | NM_016522 | HNT | -1.97 |
| NM_001070 | TUBG1 | -1.97 | NM_023076 | FLJ23360 | -1.97 |
| NM_000637 | GSR | -1.97 | NM_001949 | E2F3 | -1.97 |
| XM_291222 | DKFZP586J0619 | -1.97 | NM_024843 | CYBRD1 | -1.97 |
| NM_022740 | HIPK2 | -1.96 | NM_006429 | CCT7 | -1.96 |
| NM_000307 | POU3F4 | -1.96 | NM_006892 | DNMT3B | -1.96 |
| NM_198904 | GABRG2 | -1.96 | NM_005586 | MDFI | -1.96 |
| NM_138289 | ACTRT1 | -1.96 | NM_006466 | POLR3F | -1.96 |
| NM_019606 | FLJ20257 | -1.96 | NM_024763 | FLJ23129 | -1.96 |
| NM_198452 | PNCK | -1.96 | NM_138328 | RHBDL4 | -1.96 |
| NM_000709 | BCKDHA | -1.95 | NM_003364 | UPP1 | -1.95 |
| NM_002189 | IL15RA | -1.95 | NM_002420 | TRPM1 | -1.95 |
| NM_031450 | P5326 | -1.95 | NM_006423 | RABAC1 | -1.95 |
| NM_005431 | XRCC2 | -1.95 | NM_016034 | MRPS2 | -1.95 |
| NM_020407 | RHBG | -1.95 | NM_032890 | DISP1 | -1.95 |
| NM_005163 | AKT1 | -1.94 | NM_004496 | FOXA1 | -1.94 |
| NM_012067 | AKR7A3 | -1.94 | NM_001336 | CTSZ | -1.94 |
| NM_001969 | EIF5 | -1.94 | NM_006000 | TUBA1 | -1.94 |
| NM_003977 | AIP | -1.94 | NM_005068 | SIM1 | -1.94 |
| NM_004543 | NEB | -1.94 | NM_033375 | MYO1C | -1.94 |
| NM_001017 | RPS13 | -1.94 | NM_006829 | C10ORF116 | -1.94 |
| NM_015169 | RRS1 | -1.94 | NM_013346 | SNX12 | -1.94 |
| NM_013368 | SERTAD3 | -1.94 | NM_016175 | LOC51149 | -1.94 |
| NM_022762 | FLJ22318 | -1.94 | XM_375495 | AATK | -1.93 |
| NM_002764 | PRPS1 | -1.93 | NM_004361 | CDH7 | -1.93 |
| NM_138473 | SP1 | -1.93 | NM_002974 | SERPINB4 | -1.93 |
| NM_005721 | ACTR3 | -1.93 | NM_013285 | HUMAUANTIG | -1.93 |
| NM_016312 | WBP11 | -1.93 | NM_018254 | RCOR3 | -1.93 |
| NM_020444 | KIAA1191 | -1.93 | NM_032862 | TIGD5 | -1.93 |
| XM_291142 | LOC115548 | -1.93 | NM_138812 | LOC143241 | -1.93 |
| NM_053041 | COMMD7 | -1.93 | XM_376924 | C9ORF62 | -1.93 |
| NM_152999 | STEAP2 | -1.93 | NM_207350 | MGC72104 | -1.93 |
| NM_207346 | LOC283989 | -1.93 | XM_371314 | LOC388699 | -1.93 |
| XM_379336 | LOC401188 | -1.93 | NM_052853 | ADCK2 | -1.92 |
| NM_002714 | PPP1R10 | -1.92 | NM_145754 | KIFC2 | -1.92 |
| NM_178014 | OK/SW-CL.56 | -1.92 | NM_000508 | FGA | -1.92 |
| NM_014303 | PES1 | -1.92 | XM_372823 | OR2L2 | -1.92 |
| NM_145047 | NOR1 | -1.92 | XM_376722 | LOC155036 | -1.92 |
| XM_377725 | LOC402066 | -1.92 | NM_005307 | GRK4 | -1.91 |
| NM_138697 | TAS1R1 | -1.91 | NM_003338 | UBE2D1 | -1.91 |
| NM_004429 | EFNB1 | -1.91 | NM_000340 | SLC2A2 | -1.91 |
| NM_001655 | ARCN1 | -1.91 | NM_007198 | PROSC | -1.91 |
| NM_017827 | SARS2 | -1.91 | NM_194276 | FLJ20209 | -1.91 |
| NM_174931 | FLJ38348 | -1.91 | NM_181724 | LOC338773 | -1.91 |
| XM_294534 | LOC347169 | -1.91 | XM_379206 | LOC401081 | -1.91 |
| NM_005837 | POP7 | -1.9 | NM_000462 | UBE3A | -1.9 |

| | | | | | |
|---|---|---|---|---|---|
| NM_004229 | CRSP2 | -1.9 | NM_001547 | IFIT2 | -1.9 |
| NM_014656 | KIAA0040 | -1.9 | XM_043272 | JMJD3 | -1.9 |
| NM_014548 | TMOD2 | -1.9 | NM_024565 | FLJ14166 | -1.9 |
| NM_032765 | TRIM52 | -1.9 | NM_138452 | DHRS1 | -1.9 |
| XM_377919 | LOC402237 | -1.9 | NM_012082 | ZFPM2 | -1.89 |
| NM_004118 | FKHL18 | -1.89 | NM_005313 | GRP58 | -1.89 |
| NM_004100 | EYA4 | -1.89 | NM_001447 | FAT2 | -1.89 |
| NM_002886 | RAP2B | -1.89 | NM_021058 | HIST1H2BJ | -1.89 |
| NM_007033 | RER1 | -1.89 | NM_014178 | STXBP6 | -1.89 |
| NM_020313 | LOC57019 | -1.89 | NM_021635 | PBOV1 | -1.89 |
| NM_024633 | C14ORF139 | -1.89 | NM_031422 | CHST9 | -1.89 |
| NM_032868 | FLJ14981 | -1.89 | NM_152410 | PACRG | -1.89 |
| NM_152483 | FLJ25328 | -1.89 | NM_182700 | SP8 | -1.89 |
| XM_373367 | LOC392531 | -1.89 | NM_001003894 | CDY1B | -1.89 |
| NM_004897 | MINPP1 | -1.88 | NM_005429 | VEGFC | -1.88 |
| NM_025198 | LOC80298 | -1.88 | NM_006733 | FSHPRH1 | -1.88 |
| NM_001019 | RPS15A | -1.88 | NM_014669 | NUP93 | -1.88 |
| NM_006831 | HEAB | -1.88 | NM_030967 | KRTAP1-1 | -1.88 |
| NM_033448 | KRT6IRS | -1.88 | NM_182548 | MGC33835 | -1.88 |
| NM_173689 | CRB2 | -1.88 | XM_372128 | LOC389772 | -1.88 |
| XM_380138 | LOC402586 | -1.88 | XM_372494 | LOC390414 | -1.88 |
| NM_000215 | JAK3 | -1.87 | NM_001756 | SERPINA6 | -1.87 |
| NM_000312 | PROC | -1.87 | NM_006112 | PPIE | -1.87 |
| NM_022575 | VPS16 | -1.87 | NM_023942 | MGC3036 | -1.87 |
| NM_015645 | C1QTNF5 | -1.87 | NM_181775 | DKFZP434G0625 | -1.87 |
| NM_152420 | C9ORF41 | -1.87 | XM_116971 | LOC196993 | -1.87 |
| NM_145719 | TIGD3 | -1.87 | XM_294592 | LOC347273 | -1.87 |
| NM_004125 | GNG10 | -1.86 | NM_024075 | LENG5 | -1.86 |
| NM_004043 | ASMT | -1.86 | NM_000381 | MID1 | -1.86 |
| NM_000640 | IL13RA2 | -1.86 | AF345651 | COAS3 | -1.86 |
| J00146 | DHFRP1 | -1.86 | NM_005952 | MT1X | -1.86 |
| NM_031492 | MGC10871 | -1.86 | NM_178465 | TSPYL3 | -1.86 |
| XM_018399 | LOC144983 | -1.86 | NM_153021 | PLB1 | -1.86 |
| XM_371593 | LOC389075 | -1.86 | XM_370973 | LOC388255 | -1.86 |
| NM_001105 | ACVR1 | -1.85 | NM_018120 | FLJ10511 | -1.85 |
| NM_005347 | HSPA5 | -1.85 | NM_003183 | ADAM17 | -1.85 |
| NM_003969 | UBE2M | -1.85 | NM_003334 | UBE1 | -1.85 |
| NM_003744 | NUMB | -1.85 | NM_003827 | NAPA | -1.85 |
| NM_006366 | CAP2 | -1.85 | NM_013292 | HUMMLC2B | -1.85 |
| NM_030824 | ZNF442 | -1.85 | NM_032906 | MGC14156 | -1.85 |
| NM_001153 | ANXA4 | -1.84 | NM_002343 | LTF | -1.84 |
| NM_005911 | MAT2A | -1.84 | XM_375853 | FLJ10359 | -1.84 |
| NM_017945 | SLC35A5 | -1.84 | NM_147192 | DMBX1 | -1.84 |
| XM_295309 | LOC343052 | -1.84 | XM_374386 | LOC392617 | -1.84 |
| XM_371089 | LOC388438 | -1.84 | NM_004954 | MARK2 | -1.83 |
| NM_018030 | OSBPL1A | -1.83 | NM_003000 | SDHB | -1.83 |

| | | | | | |
|---|---|---|---|---|---|
| NM_020311 | CMKOR1 | -1.83 | NM_004369 | COL6A3 | -1.83 |
| NM_013364 | PNMA3 | -1.83 | NM_017556 | FBLP-1 | -1.83 |
| NM_022574 | PERQ1 | -1.83 | NM_199262 | SP6 | -1.83 |
| NM_139074 | DEFB127 | -1.83 | NM_198859 | PRICKLE2 | -1.83 |
| NM_012346 | NUP62 | -1.83 | XM_374014 | LOC389050 | -1.83 |
| XM_379531 | LOC401434 | -1.83 | NM_004223 | UBE2L6 | -1.82 |
| NM_000722 | CACNA2D1 | -1.82 | NM_003848 | SUCLG2 | -1.82 |
| NM_006620 | HBS1L | -1.82 | NM_013236 | E46L | -1.82 |
| NM_016573 | GMIP | -1.82 | NM_024329 | EFHD2 | -1.82 |
| XM_291019 | FLJ13305 | -1.82 | NM_032266 | C2ORF16 | -1.82 |
| NM_024996 | EFG1 | -1.82 | XM_371478 | LOC388927 | -1.82 |
| XM_371068 | LOC388401 | -1.82 | NM_006177 | NRL | -1.81 |
| NM_002480 | PPP1R12A | -1.81 | NM_001188 | BAK1 | -1.81 |
| NM_053056 | CCND1 | -1.81 | NM_175870 | LOC90925 | -1.81 |
| NM_004500 | HNRPC | -1.81 | NM_012256 | ZNF212 | -1.81 |
| NM_001120 | TETRAN | -1.81 | NM_018433 | JMJD1A | -1.81 |
| NM_022764 | FLJ12998 | -1.81 | NM_198444 | UNQ9366 | -1.81 |
| NM_153688 | ZFP1 | -1.81 | NM_145177 | DHRSX | -1.81 |
| NM_182557 | BCL9L | -1.81 | XM_088072 | LOC154907 | -1.81 |
| XM_378301 | LOC399924 | -1.81 | XM_378309 | LOC399951 | -1.81 |
| XM_372574 | LOC390595 | -1.81 | XM_375491 | LOC400622 | -1.81 |
| NM_001320 | CSNK2B | -1.8 | NM_004424 | E4F1 | -1.8 |
| NM_012331 | MSRA | -1.8 | NM_000673 | ADH7 | -1.8 |
| NM_001006 | RPS3A | -1.8 | NM_015308 | FNBP4 | -1.8 |
| NM_152316 | FLJ38968 | -1.8 | NM_194281 | C18ORF37 | -1.8 |
| XM_296817 | LOC342346 | -1.8 | XM_371736 | LOC389286 | -1.8 |
| NM_000934 | SERPINF2 | -1.79 | NM_003168 | SUPT4H1 | -1.79 |
| NM_001515 | GTF2H2 | -1.79 | NM_001316 | CSE1L | -1.79 |
| NM_004277 | SLC25A27 | -1.79 | NM_004305 | BIN1 | -1.79 |
| XM_374989 | KIAA0373 | -1.79 | NM_012126 | CHST5 | -1.79 |
| NM_024100 | WDR18 | -1.79 | NM_025239 | PDCD1LG2 | -1.79 |
| NM_032927 | MGC13159 | -1.79 | NM_144601 | CKLFSF3 | -1.79 |
| NM_173488 | SLCO6A1 | -1.79 | NM_152704 | FLJ25477 | -1.79 |
| XM_378832 | LOC284661 | -1.79 | NM_152619 | MGC45428 | -1.78 |
| NM_032682 | FOXP1 | -1.78 | NM_001335 | CTSW | -1.78 |
| NM_001354 | AKR1C2 | -1.78 | NM_001757 | CBR1 | -1.78 |
| NM_002197 | ACO1 | -1.78 | NM_005321 | HIST1H1E | -1.78 |
| NM_001412 | EIF1AX | -1.78 | NM_016167 | NOL7 | -1.78 |
| NM_016077 | BIT1 | -1.78 | NM_024772 | ZMYM1 | -1.78 |
| NM_031478 | DKFZP434I2117 | -1.78 | NM_152902 | MGC3794 | -1.78 |
| XM_173015 | LOC256483 | -1.78 | NM_003557 | PIP5K1A | -1.77 |
| NM_003323 | TULP2 | -1.77 | NM_003329 | TXN | -1.77 |
| NM_145914 | ZNF38 | -1.77 | NM_015531 | DKFZP586P0123 | -1.77 |
| NM_024076 | KCTD15 | -1.77 | NM_080862 | SSB4 | -1.77 |
| NM_153266 | MGC33486 | -1.77 | XM_063202 | LOC122585 | -1.77 |
| XM_374162 | LOC389370 | -1.77 | XM_375418 | LOC400581 | -1.77 |

| | | |
|---|---|---|
| NM_025233 | COASY | -1.76 |
| NM_020365 | EIF2B3 | -1.76 |
| NM_017532 | HSAJ2425 | -1.76 |
| NM_002947 | RPA3 | -1.76 |
| XM_113678 | NUP160 | -1.76 |
| NM_021925 | FLJ21820 | -1.76 |
| NM_138774 | C19ORF22 | -1.76 |
| NM_173806 | MGC50721 | -1.76 |
| NM_012302 | LPHN2 | -1.75 |
| NM_016229 | CYB5R2 | -1.75 |
| NM_005358 | LMO7 | -1.75 |
| NM_005576 | LOXL1 | -1.75 |
| NM_018302 | FLJ11017 | -1.75 |
| XM_375243 | KIAA1920 | -1.75 |
| XM_371108 | LOC388462 | -1.75 |
| NM_003224 | ARFRP1 | -1.74 |
| NM_021785 | RAI2 | -1.74 |
| NM_002362 | MAGEA4 | -1.74 |
| NM_005617 | RPS14 | -1.74 |
| NM_005882 | MAEA | -1.74 |
| NM_007367 | RALY | -1.74 |
| NM_145794 | DONSON | -1.74 |
| NM_024571 | C16ORF33 | -1.74 |
| NM_152391 | C2ORF22 | -1.74 |
| XM_294017 | SLC35D3 | -1.74 |
| NM_207492 | FLJ44477 | -1.74 |
| NM_001005478 | GGNBP1 | -1.74 |
| NM_007254 | PNKP | -1.73 |
| NM_173500 | TTBK2 | -1.73 |
| NM_005980 | S100P | -1.73 |
| NM_000197 | HSD17B3 | -1.73 |
| NM_004394 | DAP | -1.73 |
| NM_002494 | NDUFC1 | -1.73 |
| NM_003293 | TPS1 | -1.73 |
| NM_004704 | RNU3IP2 | -1.73 |
| NM_014033 | DKFZP586A0522 | -1.73 |
| NM_178167 | ZNF598 | -1.73 |
| XM_173105 | LOC256283 | -1.73 |
| NM_005297 | GPR24 | -1.72 |
| NM_000625 | NOS2A | -1.72 |
| NM_001851 | COL9A1 | -1.72 |
| NM_022834 | WARP | -1.72 |
| NM_052969 | RPL39L | -1.72 |
| XM_113967 | LOC201475 | -1.72 |
| XM_379461 | LOC401291 | -1.72 |
| NM_001968 | EIF4E | -1.71 |
| XM_376652 | DLX6 | -1.76 |
| NM_000286 | PEX12 | -1.76 |
| NM_004318 | ASPH | -1.76 |
| NM_004647 | DPF1 | -1.76 |
| NM_016190 | C1ORF10 | -1.76 |
| NM_032261 | C21ORF56 | -1.76 |
| NM_153685 | DKFZP547D2210 | -1.76 |
| NM_002646 | PIK3C2B | -1.75 |
| NM_006006 | ZBTB16 | -1.75 |
| NM_003567 | BCAR3 | -1.75 |
| NM_000366 | TPM1 | -1.75 |
| XM_044461 | KIAA1102 | -1.75 |
| NM_025152 | C14ORF127 | -1.75 |
| XM_371501 | MGC22014 | -1.75 |
| NM_000869 | HTR3A | -1.74 |
| NM_018494 | LRDD | -1.74 |
| NM_005185 | CALML3 | -1.74 |
| NM_005371 | METTL1 | -1.74 |
| NM_003136 | SRP54 | -1.74 |
| NM_015161 | ARL6IP | -1.74 |
| XM_049237 | KIAA0841 | -1.74 |
| NM_016604 | JMJD1B | -1.74 |
| NM_030629 | CMIP | -1.74 |
| NM_173824 | MGC26717 | -1.74 |
| NM_182600 | LOC286359 | -1.74 |
| XM_379372 | LOC401216 | -1.74 |
| NM_012096 | APPL | -1.73 |
| NM_006219 | PIK3CB | -1.73 |
| NM_030876 | OR5V1 | -1.73 |
| NM_006372 | SYNCRIP | -1.73 |
| NM_021801 | MMP26 | -1.73 |
| NM_024298 | LENG4 | -1.73 |
| NM_001026 | RPS24 | -1.73 |
| NM_014640 | TTLL4 | -1.73 |
| NM_199450 | ZNF365 | -1.73 |
| NM_016368 | ISYNA1 | -1.73 |
| NM_198690 | KRTAP18-9 | -1.73 |
| XM_377527 | LOC401911 | -1.73 |
| NM_012325 | MAPRE1 | -1.72 |
| NM_003298 | NR2C2 | -1.72 |
| NM_020839 | KIAA1449 | -1.72 |
| NM_032805 | ZNF206 | -1.72 |
| XM_058956 | PCP2 | -1.72 |
| XM_293416 | LOC347549 | -1.72 |
| NM_021634 | LGR7 | -1.71 |
| NM_012320 | LYPLA3 | -1.71 |

| | | | | | |
|---|---|---|---|---|---|
| NM_021797 | CHIA | -1.71 | NM_014570 | ARFGAP3 | -1.71 |
| NM_018947 | CYCS | -1.71 | NM_001402 | EEF1A1 | -1.71 |
| NM_003530 | HIST1H3D | -1.71 | NM_003847 | PEX11A | -1.71 |
| NM_006758 | U2AF1 | -1.71 | NM_012151 | F8A | -1.71 |
| NM_015261 | KIAA0056 | -1.71 | XM_290820 | FLJ10211 | -1.71 |
| NM_020889 | PHF12 | -1.71 | NM_032561 | C22ORF23 | -1.71 |
| NM_152331 | PTE2B | -1.71 | NM_145315 | LACE1 | -1.71 |
| NM_152783 | MGC25181 | -1.71 | NM_198851 | LOC348645 | -1.71 |
| XM_086287 | LOC148713 | -1.71 | XM_379036 | LOC400890 | -1.71 |
| NM_001204 | BMPR2 | -1.7 | NM_005283 | XCR1 | -1.7 |
| NM_006988 | ADAMTS1 | -1.7 | NM_004301 | BAF53A | -1.7 |
| NM_006528 | TFPI2 | -1.7 | NM_007053 | CD160 | -1.7 |
| NM_006435 | IFITM2 | -1.7 | NM_005897 | IPP | -1.7 |
| NM_005000 | NDUFA5 | -1.7 | NM_004553 | NDUFS6 | -1.7 |
| NM_001016 | RPS12 | -1.7 | NM_181453 | GCC2 | -1.7 |
| NM_014388 | MGC29875 | -1.7 | NM_013259 | NP25 | -1.7 |
| NM_018321 | BRIX | -1.7 | NM_145117 | NAV2 | -1.7 |
| NM_173680 | MGC33584 | -1.7 | XM_374526 | LOC392793 | -1.7 |
| XM_370763 | LOC387991 | -1.7 | NM_173655 | DKFZp434C1418 | -1.69 |
| NM_000865 | HTR1E | -1.69 | NM_005148 | UNC119 | -1.69 |
| NM_002993 | CXCL6 | -1.69 | NM_002013 | FKBP3 | -1.69 |
| NM_012304 | FBXL7 | -1.69 | NM_012179 | FBXO7 | -1.69 |
| NM_000166 | GJB1 | -1.69 | NM_031423 | CDCA1 | -1.69 |
| NM_014299 | BRD4 | -1.69 | NM_015582 | DKFZP564B147 | -1.69 |
| NM_022360 | FAM12B | -1.69 | NM_194286 | LOC144747 | -1.69 |
| NM_207476 | DKFZP547B139 | -1.69 | XM_210365 | LOC284288 | -1.69 |
| XM_374095 | LOC389242 | -1.69 | XM_378392 | LOC400087 | -1.69 |
| NM_001721 | BMX | -1.68 | NM_178510 | ANKK1 | -1.68 |
| NM_014586 | HUNK | -1.68 | NM_002613 | PDPK1 | -1.68 |
| NM_004783 | TAO1 | -1.68 | NM_004852 | ONECUT2 | -1.68 |
| NM_000532 | PCCB | -1.68 | NM_032782 | HAVCR2 | -1.68 |
| NM_144633 | KCNH8 | -1.68 | NM_013436 | NCKAP1 | -1.68 |
| NM_006924 | SFRS1 | -1.68 | NM_001084 | PLOD3 | -1.68 |
| NM_005723 | TM4SF9 | -1.68 | NM_014420 | DKK4 | -1.68 |
| NM_015849 | ELA2B | -1.68 | NM_017966 | FLJ20847 | -1.68 |
| NM_019895 | C3ORF4 | -1.68 | NM_030914 | C9ORF74 | -1.68 |
| NM_033427 | CTTNBP2 | -1.68 | NM_033504 | BCLP | -1.68 |
| NM_054114 | TAGAP | -1.68 | XM_085231 | LOC145783 | -1.68 |
| XM_371359 | LOC388763 | -1.68 | XM_372354 | LOC390059 | -1.68 |
| NM_002220 | ITPKA | -1.67 | NM_001053 | SSTR5 | -1.67 |
| NM_012148 | DUX3 | -1.67 | NM_004463 | FGD1 | -1.67 |
| NM_014213 | HOXD9 | -1.67 | NM_002445 | MSR1 | -1.67 |
| NM_022555 | HLA-DRB3 | -1.67 | NM_000800 | FGF1 | -1.67 |
| NM_030657 | LIM2 | -1.67 | NM_003125 | SPRR1B | -1.67 |
| NM_018679 | TCP11 | -1.67 | NM_005708 | GPC6 | -1.67 |
| XM_375105 | KIAA0329 | -1.67 | NM_007192 | SUPT16H | -1.67 |

| | | | | | |
|---|---|---|---|---|---|
| NM_032593 | HINT2 | -1.67 | NM_138358 | LOC90580 | -1.67 |
| XM_370927 | RNF151 | -1.67 | XM_378454 | LOC283547 | -1.67 |
| XM_292596 | LOC342541 | -1.67 | XM_379409 | LOC401256 | -1.67 |
| XM_374292 | LOC389707 | -1.67 | XM_377500 | LOC401895 | -1.67 |
| NM_138964 | GPR73 | -1.66 | NM_004559 | NSEP1 | -1.66 |
| NM_003813 | ADAM21 | -1.66 | NM_000125 | ESR1 | -1.66 |
| NM_004039 | ANXA2 | -1.66 | NM_002951 | RPN2 | -1.66 |
| NM_012113 | CA14 | -1.66 | NM_015387 | PREI3 | -1.66 |
| NM_016486 | LOC51249 | -1.66 | NM_017752 | FLJ20298 | -1.66 |
| NM_020182 | TMEPAI | -1.66 | NM_032204 | ASC1P100 | -1.66 |
| NM_052861 | MGC21675 | -1.66 | NM_174913 | C14ORF21 | -1.66 |
| XM_114447 | KIAA1999 | -1.66 | NM_173640 | R-SPONDIN | -1.66 |
| XM_371663 | LOC389147 | -1.66 | XM_378741 | LOC400644 | -1.66 |
| NM_000679 | ADRA1B | -1.65 | NM_006255 | PRKCH | -1.65 |
| NM_003942 | RPS6KA4 | -1.65 | NM_080823 | SRMS | -1.65 |
| NM_005955 | MTF1 | -1.65 | NM_175744 | RHOC | -1.65 |
| NM_017533 | MYH4 | -1.65 | NM_003093 | SNRPC | -1.65 |
| NM_013310 | C2ORF27 | -1.65 | NM_014574 | STRN3 | -1.65 |
| NM_016463 | CXXC5 | -1.65 | NM_016275 | SELT | -1.65 |
| NM_024526 | EPS8L3 | -1.65 | XM_039908 | LOC91664 | -1.65 |
| XM_088951 | OLFM3 | -1.65 | NM_144583 | ATP6V1C2 | -1.65 |
| XM_069595 | LOC135896 | -1.65 | XM_294328 | LOC346588 | -1.65 |
| XM_376412 | LOC401202 | -1.65 | XM_373578 | LOC387950 | -1.65 |
| NM_001001960 | OR5W2 | -1.65 | NM_003578 | SOAT2 | -1.64 |
| NM_004651 | USP11 | -1.64 | NM_001376 | DNCH1 | -1.64 |
| NM_001306 | CLDN3 | -1.64 | NM_006855 | KDELR3 | -1.64 |
| NM_005466 | MED6 | -1.64 | NM_003304 | TRPC1 | -1.64 |
| NM_004966 | HNRPF | -1.64 | NM_001006608 | STGC3 | -1.64 |
| NM_016161 | ALPHA4GNT | -1.64 | NM_002790 | PSMA5 | -1.64 |
| NM_002791 | PSMA6 | -1.64 | NM_001023 | RPS20 | -1.64 |
| NM_013286 | HUMAGCGB | -1.64 | NM_024113 | MGC4707 | -1.64 |
| NM_005684 | GPR52 | -1.63 | NM_014360 | NKX2-8 | -1.63 |
| NM_015401 | HDAC7A | -1.63 | NM_016239 | MYO15A | -1.63 |
| NM_022080 | NAPB | -1.63 | NM_013339 | ALG6 | -1.63 |
| NM_005383 | NEU2 | -1.63 | NM_003731 | SSNA1 | -1.63 |
| XM_032397 | DKFZP564I122 | -1.63 | NM_016046 | EXOSC1 | -1.63 |
| NM_016632 | LOC51326 | -1.63 | NM_032725 | MGC13125 | -1.63 |
| NM_207313 | LOC124842 | -1.63 | XM_378675 | LOC147093 | -1.63 |
| XM_371604 | FLJ37034 | -1.63 | XM_379321 | LOC340107 | -1.63 |
| NM_199181 | FLJ44670 | -1.63 | XM_209227 | LOC391087 | -1.63 |
| XM_372588 | LOC390633 | -1.63 | NM_001800 | CDKN2D | -1.62 |
| NM_001337 | CX3CR1 | -1.62 | NM_001617 | ADD2 | -1.62 |
| NM_006216 | SERPINE2 | -1.62 | NM_007081 | RABL2B | -1.62 |
| NM_001936 | DPP6 | -1.62 | XM_039627 | CNTN3 | -1.62 |
| NM_015969 | MRPS17 | -1.62 | NM_018073 | RNF137 | -1.62 |
| NM_018176 | LGI2 | -1.62 | NM_024953 | FLJ13089 | -1.62 |

| | | | | | |
|---|---|---|---|---|---|
| XM_208927 | FLJ36208 | -1.62 | XM_290401 | LOC340318 | -1.62 |
| NM_182704 | SELV | -1.62 | NM_206966 | MGC23985 | -1.62 |
| XM_208261 | LOC284890 | -1.62 | XM_375814 | LOC400790 | -1.62 |
| NM_005577 | LPA | -1.61 | NM_002848 | PTPRO | -1.61 |
| NM_000073 | CD3G | -1.61 | NM_000091 | COL4A3 | -1.61 |
| NM_021146 | CDT6 | -1.61 | NM_001358 | DHX15 | -1.61 |
| NM_014042 | DKFZP564M082 | -1.61 | NM_021154 | PSAT1 | -1.61 |
| NM_018218 | USP40 | -1.61 | NM_020933 | ZNF317 | -1.61 |
| NM_033053 | DMRTC1 | -1.61 | NM_024515 | MGC4645 | -1.61 |
| XM_375783 | KIAA1245 | -1.61 | XM_376303 | LOC285484 | -1.61 |
| NM_175834 | KRT6L | -1.61 | XM_371245 | LOC388625 | -1.61 |
| NM_002745 | MAPK1 | -1.6 | NM_012378 | OR8B8 | -1.6 |
| NM_020167 | NMUR2 | -1.6 | NM_001675 | ATF4 | -1.6 |
| NM_004805 | POLR2D | -1.6 | NM_002000 | FCAR | -1.6 |
| NM_004042 | ARSF | -1.6 | NM_000792 | DIO1 | -1.6 |
| NM_003480 | MFAP5 | -1.6 | NM_003493 | HIST3H3 | -1.6 |
| NM_003665 | FCN3 | -1.6 | NM_005155 | PPT2 | -1.6 |
| NM_015613 | LRRC21 | -1.6 | NM_018286 | FLJ10970 | -1.6 |
| NM_032048 | EMILIN2 | -1.6 | NM_207459 | FLJ35767 | -1.6 |
| XM_066243 | LOC128939 | -1.6 | XM_113596 | LOC196752 | -1.6 |
| XM_294261 | LOC346545 | -1.6 | XM_378472 | LOC400236 | -1.6 |
| XM_373543 | LOC387876 | -1.6 | NM_005883 | APC2 | -1.59 |
| NM_005243 | EWSR1 | -1.59 | NM_021209 | CARD12 | -1.59 |
| NM_004991 | MDS1 | -1.59 | NM_016373 | WWOX | -1.59 |
| NM_001903 | CTNNA1 | -1.59 | NM_001214 | C16ORF3 | -1.59 |
| NM_002282 | KRTHB3 | -1.59 | NM_005499 | UBA2 | -1.59 |
| NM_007069 | HRASLS3 | -1.59 | NM_015641 | TES | -1.59 |
| NM_017758 | FLJ20308 | -1.59 | NM_021227 | DC2 | -1.59 |
| XM_380171 | IRX1 | -1.59 | NM_030581 | WDR59 | -1.59 |
| NM_032518 | COL25A1 | -1.59 | NM_148178 | C9ORF23 | -1.59 |
| XM_084000 | MCART2 | -1.59 | XM_378955 | LOC400829 | -1.59 |
| XM_371645 | LOC389127 | -1.59 | XM_373252 | LOC392221 | -1.59 |
| NM_001001710 | LOC401565 | -1.59 | NM_004262 | HAT | -1.58 |
| NM_000964 | RARA | -1.58 | NM_005760 | CEBPZ | -1.58 |
| NM_024736 | GSDMDC1 | -1.58 | NM_080792 | PTPNS1 | -1.58 |
| NM_014839 | LPPR4 | -1.58 | NM_017599 | VEZATIN | -1.58 |
| NM_018379 | FLJ11280 | -1.58 | NM_020209 | SHD | -1.58 |
| NM_175872 | FLJ38451 | -1.58 | NM_152909 | ZNF548 | -1.58 |
| NM_207102 | FBXW12 | -1.58 | NM_004698 | PRPF3 | -1.58 |
| XM_373985 | LOC388948 | -1.58 | XM_377675 | LOC402016 | -1.58 |
| NM_030946 | OR5U1 | -1.58 | NM_003742 | ABCB11 | -1.57 |
| NM_003169 | SUPT5H | -1.57 | XM_495987 | GNG2 | -1.57 |
| NM_020448 | DJ462O23.2 | -1.57 | NM_152397 | MGC39725 | -1.57 |
| NM_152502 | FLJ32154 | -1.57 | XM_378201 | LOC282980 | -1.57 |
| NM_153248 | MGC14276 | -1.57 | XM_372816 | LOC391169 | -1.57 |
| XM_378648 | LOC400572 | -1.57 | XM_373922 | LOC388807 | -1.57 |

| | | | | | |
|---|---|---|---|---|---|
| NM_054105 | OR6C2 | -1.57 | NM_002980 | SCTR | -1.56 |
| NM_018899 | PCDHAC2 | -1.56 | NM_000554 | CRX | -1.56 |
| NM_022468 | MMP25 | -1.56 | NM_000547 | TPO | -1.56 |
| NM_003248 | THBS4 | -1.56 | NM_001774 | CD37 | -1.56 |
| NM_018455 | BM039 | -1.56 | NM_012091 | ADAT1 | -1.56 |
| NM_001014 | RPS10 | -1.56 | NM_017585 | SLC2A6 | -1.56 |
| NM_017417 | GALNT8 | -1.56 | NM_022101 | FLJ22965 | -1.56 |
| NM_024598 | FLJ13154 | -1.56 | NM_144707 | PROM2 | -1.56 |
| NM_178549 | MGC42493 | -1.56 | NM_198582 | FLJ43374 | -1.56 |
| XM_373546 | LOC387884 | -1.56 | NM_002627 | PFKP | -1.55 |
| NM_000018 | ACADVL | -1.55 | NM_000694 | ALDH3B1 | -1.55 |
| NM_003236 | TGFA | -1.55 | NM_007124 | UTRN | -1.55 |
| NM_012092 | ICOS | -1.55 | NM_006863 | LILRA1 | -1.55 |
| NM_018644 | B3GAT1 | -1.55 | NM_016305 | SS18L2 | -1.55 |
| NM_015885 | PCF11 | -1.55 | NM_031299 | CDCA3 | -1.55 |
| NM_031440 | TMEM7 | -1.55 | NM_032562 | PLA2G12B | -1.55 |
| NM_130441 | CLECSF7 | -1.55 | XM_372950 | LOC391426 | -1.55 |
| NM_001260 | CDK8 | -1.54 | NM_017425 | SPA17 | -1.54 |
| NM_006599 | NFAT5 | -1.54 | NM_002622 | PFDN1 | -1.54 |
| NM_004737 | LARGE | -1.54 | NM_031886 | KCNA7 | -1.54 |
| NM_018410 | DKFZP762E1312 | -1.54 | NM_001436 | FBL | -1.54 |
| NM_003080 | SMPD2 | -1.54 | XM_375651 | KIAA1115 | -1.54 |
| NM_015962 | C14ORF111 | -1.54 | NM_018589 | C14ORF116 | -1.54 |
| NM_018918 | PCDHGA5 | -1.54 | NM_020854 | KIAA1468 | -1.54 |
| NM_138460 | CKLFSF5 | -1.54 | NM_153044 | FLJ35801 | -1.54 |
| NM_178552 | MGC35206 | -1.54 | XM_091886 | LOC162962 | -1.54 |
| XM_294692 | LOC338749 | -1.54 | XM_374836 | LOC399815 | -1.54 |
| NM_033180 | OR51B2 | -1.53 | NM_004634 | BRPF1 | -1.53 |
| NM_005496 | SMC4L1 | -1.53 | NM_000199 | SGSH | -1.53 |
| NM_014697 | CAPON | -1.53 | NM_006919 | SERPINB3 | -1.53 |
| NM_016085 | C2ORF28 | -1.53 | NM_006101 | KNTC2 | -1.53 |
| NM_021916 | ZNF70 | -1.53 | NM_145238 | ZNF31 | -1.53 |
| NM_014366 | NS | -1.53 | NM_018230 | NUP133 | -1.53 |
| NM_021165 | KIAA1747 | -1.53 | NM_032588 | RNF28 | -1.53 |
| NM_080839 | GGTL4 | -1.53 | NM_058172 | ANTXR2 | -1.53 |
| NM_080871 | ASB10 | -1.53 | NM_207323 | DKFZP667M2411 | -1.53 |
| XM_371923 | LOC389523 | -1.53 | XM_370649 | LOC387805 | -1.53 |
| XM_370883 | LOC388148 | -1.53 | NM_001184 | ATR | -1.52 |
| NM_014975 | SAST | -1.52 | NM_005928 | MFGE8 | -1.52 |
| NM_006640 | MSF | -1.52 | NM_012461 | TINF2 | -1.52 |
| NM_003167 | SULT2A1 | -1.52 | NM_005364 | MAGEA8 | -1.52 |
| NM_018956 | C9ORF9 | -1.52 | NM_024793 | KIAA0643 | -1.52 |
| NM_015534 | ZZZ3 | -1.52 | NM_016613 | DKFZP434L142 | -1.52 |
| NM_018260 | FLJ10891 | -1.52 | NM_018023 | FLJ10201 | -1.52 |
| NM_052942 | GBP5 | -1.52 | NM_020841 | OSBPL8 | -1.52 |
| NM_138373 | MYADM | -1.52 | NM_153338 | FLJ90165 | -1.52 |

| | | | | | |
|---|---|---|---|---|---|
| NM_080431 | ARPM2 | -1.52 | NM_139282 | OTEX | -1.52 |
| NM_152749 | MGC33190 | -1.52 | NM_173694 | ATP11C | -1.52 |
| NM_181726 | LRP2BP | -1.52 | NM_178177 | NMNAT3 | -1.52 |
| NM_003511 | HIST1H2AL | -1.52 | XM_495886 | KIAA1991 | -1.52 |
| XM_371490 | LOC388943 | -1.52 | NM_000875 | IGF1R | -1.51 |
| NM_006496 | GNAI3 | -1.51 | NM_019839 | LTB4R2 | -1.51 |
| NM_004571 | PKNOX1 | -1.51 | NM_005597 | NFIC | -1.51 |
| NM_138340 | ABHD3 | -1.51 | NM_000196 | HSD11B2 | -1.51 |
| NM_031282 | IRTA1 | -1.51 | NM_052850 | GADD45GIP1 | -1.51 |
| NM_024700 | SNIP1 | -1.51 | NM_003849 | SUCLG1 | -1.51 |
| NM_019043 | APBB1IP | -1.51 | NM_144570 | C16ORF34 | -1.51 |
| NM_138369 | LOC91272 | -1.51 | XM_055481 | KIAA1915 | -1.51 |
| NM_033419 | PERLD1 | -1.51 | NM_152590 | FLJ36004 | -1.51 |
| XM_059396 | LOC130063 | -1.51 | XM_294019 | LOC345930 | -1.51 |
| XM_372640 | LOC390734 | -1.51 | NM_014791 | MELK | -1.5 |
| NM_002082 | GRK6 | -1.5 | NM_001571 | IRF3 | -1.5 |
| NM_020699 | P66BETA | -1.5 | NM_004861 | GAL3ST1 | -1.5 |
| NM_000257 | MYH7 | -1.5 | NM_002333 | LRP3 | -1.5 |
| NM_000121 | EPOR | -1.5 | NM_016112 | PKD2L1 | -1.5 |
| NM_021127 | PMAIP1 | -1.5 | NM_002455 | MTX1 | -1.5 |
| NM_006408 | AGR2 | -1.5 | NM_003314 | TTC1 | -1.5 |
| NM_004846 | EIF4EL3 | -1.5 | NM_015224 | RAP140 | -1.5 |
| NM_014026 | DCPS | -1.5 | XM_372927 | TTC7A | -1.5 |
| XM_372083 | KIAA1161 | -1.5 | NM_024032 | MGC3130 | -1.5 |
| NM_017647 | FTSJ3 | -1.5 | NM_145014 | FLJ32915 | -1.5 |
| NM_182582 | MGC34919 | -1.5 | XM_371492 | LOC388949 | -1.5 |
| XM_371715 | LOC389240 | -1.5 | NM_002756 | MAP2K3 | -1.49 |
| NM_006625 | FUSIP1 | -1.49 | NM_017534 | MYH2 | -1.49 |
| NM_014223 | NFYC | -1.49 | NM_000588 | IL3 | -1.49 |
| NM_001999 | FBN2 | -1.49 | NM_012111 | AHSA1 | -1.49 |
| NM_004748 | CPR8 | -1.49 | NM_001025 | RPS23 | -1.49 |
| NM_016481 | C9ORF156 | -1.49 | NM_018114 | FLJ10496 | -1.49 |
| NM_024742 | FLJ13063 | -1.49 | NM_152338 | ZG16 | -1.49 |
| NM_058166 | TRIM6 | -1.49 | NM_138701 | C7ORF11 | -1.49 |
| XM_211287 | LOC283999 | -1.49 | NM_198083 | DHRS4L2 | -1.49 |
| NM_001001963 | OR2L8 | -1.49 | NM_016340 | RAPGEF6 | -1.48 |
| NM_004188 | GFI1B | -1.48 | NM_002316 | LMX1B | -1.48 |
| NM_000402 | G6PD | -1.48 | NM_001379 | DNMT1 | -1.48 |
| NM_012475 | USP21 | -1.48 | NM_021057 | IFNA7 | -1.48 |
| NM_016240 | SCARA3 | -1.48 | NM_001832 | CLPS | -1.48 |
| NM_005545 | ISLR | -1.48 | NM_003275 | TMOD1 | -1.48 |
| NM_004765 | BCL7C | -1.48 | NM_006824 | EBNA1BP2 | -1.48 |
| NM_015681 | EPPB9 | -1.48 | NM_017426 | NUP54 | -1.48 |
| NM_018032 | LUC7L | -1.48 | NM_025132 | PWDMP | -1.48 |
| NM_022492 | FLJ12788 | -1.48 | NM_024297 | MGC2941 | -1.48 |
| NM_024573 | C6ORF211 | -1.48 | NM_032318 | HIATL2 | -1.48 |

| | | | | | |
|---|---|---|---|---|---|
| XM_058426 | FLJ00012 | -1.48 | XM_378712 | LOC146713 | -1.48 |
| XM_097886 | LOC150223 | -1.48 | NM_139177 | SLC39A11 | -1.48 |
| NM_207390 | FLJ45910 | -1.48 | XM_380069 | LOC402714 | -1.48 |
| XM_370714 | LOC387908 | -1.48 | XM_373690 | LOC388282 | -1.48 |
| NM_022158 | FN3K | -1.47 | NM_002767 | PRPSAP2 | -1.47 |
| NM_000323 | RET | -1.47 | NM_001482 | GATM | -1.47 |
| NM_003879 | CFLAR | -1.47 | NM_001202 | BMP4 | -1.47 |
| NM_000343 | SLC5A1 | -1.47 | NM_000097 | CPOX | -1.47 |
| NM_017526 | OBRGRP | -1.47 | NM_012409 | PRND | -1.47 |
| NM_021014 | SSX3 | -1.47 | NM_007030 | P25 | -1.47 |
| NM_007252 | POU6F2 | -1.47 | NM_015139 | SLC35D1 | -1.47 |
| NM_015556 | SIPA1L1 | -1.47 | NM_018250 | FLJ10871 | -1.47 |
| NM_020204 | LHX9 | -1.47 | NM_030907 | MGC10731 | -1.47 |
| NM_033258 | GNG8 | -1.47 | NM_020461 | TUBGCP6 | -1.47 |
| NM_194250 | LOC91752 | -1.47 | NM_138572 | TBN | -1.47 |
| XM_210022 | GARNL1 | -1.47 | NM_207482 | FLJ44048 | -1.47 |
| XM_371672 | LOC389168 | -1.47 | NM_001717 | BNC1 | -1.46 |
| NM_004824 | CDYL | -1.46 | NM_003935 | TOP3B | -1.46 |
| NM_002431 | MNAT1 | -1.46 | NM_002121 | HLA-DPB1 | -1.46 |
| NM_001566 | INPP4A | -1.46 | NM_002256 | KISS1 | -1.46 |
| NM_001356 | DDX3X | -1.46 | NM_002692 | POLE2 | -1.46 |
| NM_003574 | VAPA | -1.46 | NM_003782 | B3GALT4 | -1.46 |
| NM_018146 | FLJ10581 | -1.46 | NM_024513 | FYCO1 | -1.46 |
| NM_032301 | FBXW9 | -1.46 | NM_022776 | OSBPL11 | -1.46 |
| XM_085433 | FLJ25404 | -1.46 | NM_138288 | C14ORF147 | -1.46 |
| XM_166659 | OTUD1 | -1.46 | XM_166747 | LOC219797 | -1.46 |
| XM_370716 | LOC387912 | -1.46 | XM_374354 | LOC390550 | -1.46 |
| NM_001006655 | DKFZP564J102 | -1.46 | NM_015178 | RHOBTB2 | -1.45 |
| NM_006917 | RXRG | -1.45 | NM_000044 | AR | -1.45 |
| NM_002273 | KRT8 | -1.45 | NM_000397 | CYBB | -1.45 |
| NM_032667 | BSCL2 | -1.45 | NM_032489 | ACRBP | -1.45 |
| NM_006979 | SLC39A7 | -1.45 | NM_005737 | ARL7 | -1.45 |
| NM_012451 | SYNGR4 | -1.45 | NM_207514 | FLJ20186 | -1.45 |
| NM_018196 | TMLHE | -1.45 | NM_024164 | TPSB2 | -1.45 |
| NM_153204 | C21ORF90 | -1.45 | NM_178841 | RNF166 | -1.45 |
| NM_145276 | ZNF563 | -1.45 | NM_153263 | ZNF549 | -1.45 |
| XM_372759 | LOC390998 | -1.45 | XM_378908 | LOC400793 | -1.45 |
| XM_373354 | LOC392487 | -1.45 | NM_018216 | PANK4 | -1.44 |
| NM_000839 | GRM2 | -1.44 | NM_172373 | ELF1 | -1.44 |
| NM_001959 | EEF1B2 | -1.44 | NM_000961 | PTGIS | -1.44 |
| NM_006313 | USP15 | -1.44 | NM_004977 | KCNC3 | -1.44 |
| NM_006121 | KRT1 | -1.44 | NM_001246 | ENTPD2 | -1.44 |
| NM_004116 | FKBP1B | -1.44 | NM_002557 | OVGP1 | -1.44 |
| NM_018102 | ZNF334 | -1.44 | NM_173490 | LOC134285 | -1.44 |
| NM_153345 | FLJ90586 | -1.44 | NM_152680 | FLJ32028 | -1.44 |
| NM_178034 | PLA2G4D | -1.44 | XM_210860 | LOC283034 | -1.44 |

| | | | | | |
|---|---|---|---|---|---|
| XM_376558 | LOC401293 | -1.44 | XM_379605 | LOC401477 | -1.44 |
| NM_023067 | FOXL2 | -1.43 | NM_014140 | SMARCAL1 | -1.43 |
| NM_006241 | PPP1R2 | -1.43 | NM_012163 | FBXL9 | -1.43 |
| NM_001963 | EGF | -1.43 | NM_003650 | CST7 | -1.43 |
| NM_001238 | CCNE1 | -1.43 | NM_004746 | DLGAP1 | -1.43 |
| NM_014890 | DOC1 | -1.43 | NM_000285 | PEPD | -1.43 |
| NM_058222 | TECTB | -1.43 | XM_042841 | RAFTLIN | -1.43 |
| XM_370754 | THTPA | -1.43 | NM_032522 | MGC2629 | -1.43 |
| NM_173847 | SPACA3 | -1.43 | NM_152466 | FLJ25168 | -1.43 |
| NM_152427 | CFLP1 | -1.43 | NM_173796 | MGC24125 | -1.43 |
| NM_175889 | FLJ37045 | -1.43 | XM_379355 | LOC340074 | -1.43 |
| XM_371326 | LOC388714 | -1.43 | NM_002110 | HCK | -1.42 |
| NM_006711 | RNPS1 | -1.42 | NM_007312 | HYAL1 | -1.42 |
| NM_003124 | SPR | -1.42 | NM_003337 | UBE2B | -1.42 |
| NM_020244 | CHPT1 | -1.42 | NM_032166 | TREX1 | -1.42 |
| NM_002428 | MMP15 | -1.42 | NM_003738 | PTCH2 | -1.42 |
| NM_004881 | TP53I3 | -1.42 | NM_002043 | GABRR2 | -1.42 |
| NM_000587 | C7 | -1.42 | NM_001724 | BPGM | -1.42 |
| NM_006474 | T1A-2 | -1.42 | NM_017590 | ROXAN | -1.42 |
| NM_014471 | SPINK4 | -1.42 | NM_015721 | GEMIN4 | -1.42 |
| NM_025002 | C6ORF208 | -1.42 | XM_034262 | KIAA1727 | -1.42 |
| NM_033119 | NKD1 | -1.42 | NM_018100 | EFHC1 | -1.42 |
| XM_036218 | ZNF506 | -1.42 | NM_203411 | LOC92162 | -1.42 |
| NM_058230 | ZNF354B | -1.42 | NM_080870 | DPCR1 | -1.42 |
| XM_378667 | LOC147004 | -1.42 | NM_177965 | LOC157657 | -1.42 |
| NM_139246 | C9ORF97 | -1.42 | NM_145062 | C6ORF113 | -1.42 |
| NM_152723 | FLJ38159 | -1.42 | NM_177550 | SLC13A5 | -1.42 |
| NM_199182 | IMAA | -1.42 | XM_290866 | LOC339377 | -1.42 |
| XM_292624 | LOC342600 | -1.42 | XM_059672 | LOC133874 | -1.42 |
| XM_374016 | LOC389055 | -1.42 | XM_379339 | LOC401199 | -1.42 |
| XM_378316 | LOC399959 | -1.42 | NM_004857 | AKAP5 | -1.41 |
| NM_002446 | MAP3K10 | -1.41 | NM_018971 | GPR27 | -1.41 |
| NM_024507 | KREMEN2 | -1.41 | XM_166103 | DNA2L | -1.41 |
| NM_003521 | HIST1H2BM | -1.41 | NM_003766 | BECN1 | -1.41 |
| NM_174902 | LOC143458 | -1.41 | NM_153705 | MGC33424 | -1.41 |
| NM_021649 | TIRP | -1.41 | NM_206921 | C6ORF204 | -1.41 |
| XM_063481 | LOC123103 | -1.41 | XM_291857 | LOC341098 | -1.41 |
| XM_376269 | LOC401089 | -1.41 | XM_372632 | LOC390714 | -1.41 |
| NM_025164 | KIAA0999 | -1.4 | NM_000683 | ADRA2C | -1.4 |
| NM_153002 | GPR156 | -1.4 | NM_005293 | GPR20 | -1.4 |
| NM_001087 | AAMP | -1.4 | NM_016223 | PACSIN3 | -1.4 |
| NM_004139 | LBP | -1.4 | NM_006058 | TNIP1 | -1.4 |
| NM_006024 | TAX1BP1 | -1.4 | NM_016427 | TCEB3B | -1.4 |
| NM_013243 | SCG3 | -1.4 | NM_001719 | BMP7 | -1.4 |
| NM_002336 | LRP6 | -1.4 | NM_152703 | C7orf6 | -1.4 |
| NM_002188 | IL13 | -1.4 | NM_005132 | REC8L1 | -1.4 |

| | | | | | |
|---|---|---|---|---|---|
| NM_005218 | DEFB1 | -1.4 | NM_018714 | COG1 | -1.4 |
| NM_014830 | ZBTB39 | -1.4 | NM_019850 | NGEF | -1.4 |
| NM_015871 | ZNF593 | -1.4 | NM_015893 | PRH | -1.4 |
| NM_020426 | LOC57151 | -1.4 | NM_025019 | TUBA4 | -1.4 |
| NM_025084 | FLJ22795 | -1.4 | NM_030964 | SPRY4 | -1.4 |
| NM_033404 | KNDC1 | -1.4 | NM_145044 | ZNF501 | -1.4 |
| NM_182525 | FLJ32770 | -1.4 | NM_199280 | LOC165186 | -1.4 |
| NM_153231 | ZNF550 | -1.4 | XM_290463 | FAM22A | -1.4 |
| XM_378544 | LOC283682 | -1.4 | XM_209563 | LOC285311 | -1.4 |
| XM_293412 | LOC347544 | -1.4 | XM_290547 | LOC338734 | -1.4 |
| XM_208563 | LOC283202 | -1.4 | XM_379184 | LOC401059 | -1.4 |
| XM_373616 | LOC388073 | -1.4 | NM_001004336 | FLJ90757 | -1.4 |
| NM_016507 | CRK7 | -1.39 | NM_003610 | RAE1 | -1.39 |
| NM_004869 | VPS4B | -1.39 | NM_014267 | SMAP | -1.39 |
| NM_018025 | GPATC1 | -1.39 | NM_018665 | DDX43 | -1.39 |
| NM_022346 | HCAP-G | -1.39 | NM_014801 | FLJ11383 | -1.39 |
| NM_138412 | RDH13 | -1.39 | XM_166420 | PHACTR1 | -1.39 |
| NM_198501 | FLJ42461 | -1.39 | XM_063315 | LOC122748 | -1.39 |
| XM_170840 | LOC256223 | -1.39 | NM_003113 | SP100 | -1.38 |
| NM_001422 | ELF5 | -1.38 | NM_003470 | USP7 | -1.38 |
| NM_017912 | HERC6 | -1.38 | NM_002262 | KLRD1 | -1.38 |
| NM_000688 | ALAS1 | -1.38 | NM_001302 | CORT | -1.38 |
| NM_005322 | HIST1H1B | -1.38 | NM_015909 | NAG | -1.38 |
| NM_020817 | KIAA1407 | -1.38 | NM_025205 | EG1 | -1.38 |
| NM_024933 | FLJ12056 | -1.38 | NM_032531 | KIRREL3 | -1.38 |
| NM_138342 | LOC89944 | -1.38 | NM_205543 | ALS2CR16 | -1.38 |
| NM_152421 | MGC20262 | -1.38 | NM_153030 | FLJ31958 | -1.38 |
| NM_152598 | FLJ35757 | -1.38 | NM_199129 | KUA | -1.38 |
| NM_001001711 | DDI1 | -1.38 | NM_052841 | STK22C | -1.37 |
| NM_000910 | NPY2R | -1.37 | NM_016084 | RASD1 | -1.37 |
| NM_001941 | DSC3 | -1.37 | NM_005531 | IFI16 | -1.37 |
| NM_013261 | PPARGC1A | -1.37 | NM_006191 | PA2G4 | -1.37 |
| NM_004001 | FCGR2B | -1.37 | NM_001148 | ANK2 | -1.37 |
| NM_001000 | RPL39 | -1.37 | NM_003527 | HIST1H2BO | -1.37 |
| NM_017742 | ZCCHC2 | -1.37 | NM_017673 | C1ORF26 | -1.37 |
| NM_018393 | FLJ11336 | -1.37 | NM_021944 | C14ORF93 | -1.37 |
| NM_024741 | ZNF408 | -1.37 | NM_024845 | FLJ14154 | -1.37 |
| XM_376094 | LOC90499 | -1.37 | NM_052970 | HSPA12B | -1.37 |
| NM_144612 | LOXHD1 | -1.37 | NM_144703 | C20ORF40 | -1.37 |
| NM_198572 | MGC61633 | -1.37 | NM_207447 | UNQ9370 | -1.37 |
| XM_209753 | LOC285770 | -1.37 | XM_376320 | LOC401136 | -1.37 |
| NM_001005483 | OR4K5 | -1.37 | NM_021629 | GNB4 | -1.36 |
| NM_054032 | MRGX4 | -1.36 | NM_001049 | SSTR1 | -1.36 |
| NM_015450 | POT1 | -1.36 | NM_002874 | RAD23B | -1.36 |
| NM_054113 | CIB3 | -1.36 | NM_152490 | MGC39558 | -1.36 |
| NM_004397 | DDX6 | -1.36 | NM_005543 | INSL3 | -1.36 |

| | | | | | |
|---|---|---|---|---|---|
| NM_002173 | IFNA16 | -1.36 | NM_003862 | FGF18 | -1.36 |
| NM_000396 | CTSK | -1.36 | NM_015991 | C1QA | -1.36 |
| NM_021123 | GAGE7 | -1.36 | NM_005898 | M11S1 | -1.36 |
| NM_006760 | UPK2 | -1.36 | NM_002904 | RDBP | -1.36 |
| NM_006032 | CPNE6 | -1.36 | NM_018203 | FLJ10748 | -1.36 |
| NM_033225 | CSMD1 | -1.36 | NM_032705 | MGC14801 | -1.36 |
| NM_033510 | DISP2 | -1.36 | NM_144674 | FLJ32871 | -1.36 |
| XM_088551 | KIAA2026 | -1.36 | NM_181774 | SLC36A3 | -1.36 |
| NM_182613 | FLJ33915 | -1.36 | XM_378861 | LOC400752 | -1.36 |
| NM_002106 | H2AFZ | -1.35 | NM_032580 | HES7 | -1.35 |
| NM_006048 | UBE4B | -1.35 | NM_000746 | CHRNA7 | -1.35 |
| NM_172369 | C1QG | -1.35 | NM_002318 | LOXL2 | -1.35 |
| NM_003546 | HIST1H4L | -1.35 | NM_004902 | RNPC2 | -1.35 |
| NM_152892 | DKFZP434K1815 | -1.35 | XM_373033 | LOC391701 | -1.35 |
| NM_001700 | AZU1 | -1.34 | NM_006575 | MAP4K5 | -1.34 |
| NM_001837 | CCR3 | -1.34 | NM_006637 | OR5I1 | -1.34 |
| NM_014921 | LPHN1 | -1.34 | NM_006265 | RAD21 | -1.34 |
| NM_002914 | RFC2 | -1.34 | NM_022171 | TCTA | -1.34 |
| NM_002413 | MGST2 | -1.34 | NM_015466 | PTPN23 | -1.34 |
| NM_000808 | GABRA3 | -1.34 | NM_007121 | NR1H2 | -1.34 |
| NM_004269 | CRSP8 | -1.34 | NM_001887 | CRYBB1 | -1.34 |
| NM_005850 | SF3B4 | -1.34 | NM_007062 | PWP1 | -1.34 |
| NM_014019 | HSPC009 | -1.34 | NM_016039 | C14ORF166 | -1.34 |
| NM_020117 | LARS | -1.34 | NM_018018 | SLC38A4 | -1.34 |
| NM_022138 | SMOC2 | -1.34 | NM_024805 | C18ORF22 | -1.34 |
| NM_033515 | ARHGAP18 | -1.34 | NM_174976 | ZDHHC22 | -1.34 |
| XM_043500 | LOC92196 | -1.34 | XM_371268 | LOC388649 | -1.34 |
| XM_379161 | LOC401037 | -1.34 | XM_374276 | LOC389676 | -1.34 |
| XM_373605 | LOC388010 | -1.34 | XM_373372 | LOC392546 | -1.34 |
| NM_007199 | IRAK3 | -1.33 | NM_021733 | TSKS | -1.33 |
| NM_019041 | MTRF1L | -1.33 | NM_003365 | UQCRC1 | -1.33 |
| NM_003477 | PDHX | -1.33 | NM_002639 | SERPINB5 | -1.33 |
| NM_002123 | HLA-DQB1 | -1.33 | NM_004494 | HDGF | -1.33 |
| NM_001330 | CTF1 | -1.33 | NM_016610 | TLR8 | -1.33 |
| NM_145061 | C13ORF3 | -1.33 | NM_006442 | DRAP1 | -1.33 |
| NM_002161 | IARS | -1.33 | NM_021012 | KCNJ12 | -1.33 |
| NM_003142 | SSB | -1.33 | NM_199328 | CLDN8 | -1.33 |
| NM_004294 | MTRF1 | -1.33 | NM_004816 | C9ORF61 | -1.33 |
| NM_007372 | DDX42 | -1.33 | NM_015201 | BOP1 | -1.33 |
| NM_017773 | LAX | -1.33 | NM_017839 | FLJ20481 | -1.33 |
| NM_018273 | FLJ10922 | -1.33 | NM_024706 | FLJ13479 | -1.33 |
| NM_025240 | B7H3 | -1.33 | XM_056298 | KIAA1889 | -1.33 |
| XM_371015 | USP43 | -1.33 | NM_152329 | PPIL5 | -1.33 |
| NM_144704 | FLJ30473 | -1.33 | XM_088118 | FAM10A7 | -1.33 |
| NM_182588 | DKFZP686P0288 | -1.33 | NM_207416 | FLJ44082 | -1.33 |
| NM_003525 | HIST1H2BI | -1.33 | XM_086616 | LOC149643 | -1.33 |

| | | | | | |
|---|---|---|---|---|---|
| XM_211837 | LOC285307 | -1.33 | XM_086637 | LOC149709 | -1.33 |
| XM_379177 | LOC401053 | -1.33 | XM_372521 | LOC390466 | -1.33 |
| XM_373800 | LOC388528 | -1.33 | NM_001005519 | OR6C68 | -1.33 |
| NM_025197 | CDK5RAP3 | -1.32 | NM_001838 | CCR7 | -1.32 |
| NM_014278 | APG-1 | -1.32 | NM_006761 | YWHAE | -1.32 |
| NM_004959 | NR5A1 | -1.32 | NM_004603 | STX1A | -1.32 |
| NM_052951 | DNTTIP1 | -1.32 | NM_018010 | ESRRBL1 | -1.32 |
| NM_003630 | PEX3 | -1.32 | NM_006170 | NOL1 | -1.32 |
| NM_004888 | ATP6V1G1 | -1.32 | XM_042833 | ZNF609 | -1.32 |
| NM_022109 | CDW92 | -1.32 | NM_014394 | GHITM | -1.32 |
| NM_024603 | FLJ11588 | -1.32 | NM_030628 | KIAA1698 | -1.32 |
| NM_033128 | SCIN | -1.32 | NM_207312 | LOC112714 | -1.32 |
| NM_144614 | MBD3L2 | -1.32 | XM_085634 | LOC146909 | -1.32 |
| NM_178483 | C20ORF79 | -1.32 | XM_094794 | DACT2 | -1.32 |
| NM_144977 | FAM31B | -1.32 | XM_167709 | C10ORF38 | -1.32 |
| XM_373030 | LOC285556 | -1.32 | NM_152286 | C9ORF111 | -1.32 |
| XM_210501 | LOC286528 | -1.32 | XM_372625 | LOC390704 | -1.32 |
| NM_006209 | ENPP2 | -1.31 | NM_005388 | PDCL | -1.31 |
| NM_002873 | RAD17 | -1.31 | NM_006897 | HOXC9 | -1.31 |
| NM_177400 | NKX6-2 | -1.31 | NM_000458 | TCF2 | -1.31 |
| NM_015570 | AUTS2 | -1.31 | NM_000106 | CYP2D6 | -1.31 |
| NM_003719 | PDE8B | -1.31 | NM_001663 | ARF6 | -1.31 |
| NM_019887 | DIABLO | -1.31 | NM_001098 | ACO2 | -1.31 |
| NM_001863 | COX6B1 | -1.31 | NM_000767 | CYP2B6 | -1.31 |
| NM_014429 | MORC | -1.31 | NM_198795 | TDRD1 | -1.31 |
| XM_049952 | FLJ23529 | -1.31 | XM_095991 | C9ORF81 | -1.31 |
| NM_032664 | FUT10 | -1.31 | NM_138456 | MGC20410 | -1.31 |
| NM_152339 | MGC26885 | -1.31 | NM_182534 | FLJ23703 | -1.31 |
| XM_379371 | LOC285626 | -1.31 | XM_172860 | LOC255649 | -1.31 |
| XM_371643 | LOC389124 | -1.31 | XM_372952 | LOC391427 | -1.31 |
| XM_372654 | LOC390760 | -1.31 | XM_372202 | LOC389844 | -1.31 |
| NM_005184 | CALM3 | -1.3 | NM_006875 | PIM2 | -1.3 |
| NM_018980 | TAS2R5 | -1.3 | NM_003411 | ZFY | -1.3 |
| NM_000383 | AIRE | -1.3 | NM_020385 | XPMC2H | -1.3 |
| NM_001692 | ATP6V1B1 | -1.3 | NM_000660 | TGFB1 | -1.3 |
| NM_021625 | TRPV4 | -1.3 | NM_006837 | COPS5 | -1.3 |
| NM_001759 | CCND2 | -1.3 | NM_031412 | GABARAPL1 | -1.3 |
| NM_033084 | FANCD2 | -1.3 | NM_001371 | DNAH8 | -1.3 |
| XM_372195 | AGRN | -1.3 | NM_013280 | FLRT1 | -1.3 |
| NM_014604 | TAX1BP3 | -1.3 | NM_018075 | FLJ10375 | -1.3 |
| NM_017871 | FLJ20542 | -1.3 | NM_031443 | CCM2 | -1.3 |
| NM_031453 | C10ORF45 | -1.3 | NM_178494 | FLJ40125 | -1.3 |
| NM_152434 | CWF19L2 | -1.3 | NM_176890 | TAS2R50 | -1.3 |
| XM_039721 | LOC91632 | -1.3 | XM_087182 | LOC151363 | -1.3 |
| XM_372035 | LOC389643 | -1.3 | XM_373572 | LOC387941 | -1.3 |
| NM_018638 | EKI1 | -1.29 | NM_024800 | NEK11 | -1.29 |

| | | | | | |
|---|---|---|---|---|---|
| NM_005647 | TBL1X | -1.29 | NM_005025 | SERPINI1 | -1.29 |
| NM_004505 | USP6 | -1.29 | NM_013371 | IL19 | -1.29 |
| NM_004831 | CRSP7 | -1.29 | NM_002555 | SLC22A18 | -1.29 |
| NM_006606 | RBBP9 | -1.29 | NM_005335 | HCLS1 | -1.29 |
| NM_001012 | RPS8 | -1.29 | NM_014749 | KIAA0586 | -1.29 |
| NM_014447 | ARFIP1 | -1.29 | NM_021208 | C9ORF27 | -1.29 |
| XM_371286 | MGC45731 | -1.29 | NM_018593 | SLC16A10 | -1.29 |
| NM_145033 | C21ORF100 | -1.29 | NM_207358 | LOC339789 | -1.29 |
| NM_207391 | FLJ45744 | -1.29 | NM_199339 | LOC374768 | -1.29 |
| NM_203348 | MGC50722 | -1.29 | XM_292021 | LOC341346 | -1.29 |
| XM_373746 | LOC388416 | -1.29 | NM_005787 | ALG3 | -1.28 |
| NM_002388 | MCM3 | -1.28 | NM_014369 | PTPN18 | -1.28 |
| NM_015532 | GRINL1A | -1.28 | NM_005547 | IVL | -1.28 |
| NM_014041 | SPC12 | -1.28 | NM_016380 | LOC51212 | -1.28 |
| NM_016497 | MRPL51 | -1.28 | NM_020144 | PAPOLB | -1.28 |
| NM_024567 | FLJ21616 | -1.28 | NM_030891 | LRRC3 | -1.28 |
| NM_207345 | UNQ9341 | -1.28 | NM_182591 | FLJ37673 | -1.28 |
| NM_178174 | TREML1 | -1.28 | NM_017437 | CPSF2 | -1.28 |
| XM_063308 | LOC122740 | -1.28 | XM_059923 | LOC137829 | -1.28 |
| XM_211174 | LOC283710 | -1.28 | XM_091809 | LOC147468 | -1.28 |
| XM_089863 | LOC143502 | -1.28 | XM_377594 | LOC401954 | -1.28 |
| XM_372778 | LOC391035 | -1.28 | XM_379797 | LOC402469 | -1.28 |
| XM_379998 | LOC402644 | -1.28 | XM_372030 | LOC389633 | -1.28 |
| XM_378358 | LOC400041 | -1.28 | NM_001004136 | OR2T2 | -1.28 |
| NM_005881 | BCKDK | -1.27 | NM_021027 | UGT1A9 | -1.27 |
| NM_002213 | ITGB5 | -1.27 | XM_291055 | KIAA1268 | -1.27 |
| NM_000135 | FANCA | -1.27 | NM_176820 | NALP9 | -1.27 |
| NM_005212 | CSN3 | -1.27 | NM_002804 | PSMC3 | -1.27 |
| NM_005715 | UST | -1.27 | NM_006638 | RPP40 | -1.27 |
| NM_014316 | CARHSP1 | -1.27 | NM_018981 | DNAJC10 | -1.27 |
| XM_027307 | PLEKHG1 | -1.27 | NM_022160 | DMRTA1 | -1.27 |
| NM_052948 | SNX26 | -1.27 | NM_138983 | OLIG1 | -1.27 |
| NM_213597 | LOC124751 | -1.27 | NM_153329 | MGC10204 | -1.27 |
| NM_080825 | C20ORF144 | -1.27 | XM_370651 | FLJ32810 | -1.27 |
| NM_201286 | USP51 | -1.27 | NM_153699 | GSTA5 | -1.27 |
| NM_145299 | LOC200383 | -1.27 | NM_152750 | FLJ23834 | -1.27 |
| NM_205852 | UNQ5782 | -1.27 | NM_178349 | LCE1B | -1.27 |
| XM_373073 | LOC391761 | -1.27 | XM_378766 | LOC400669 | -1.27 |
| NM_004148 | NINJ1 | -1.26 | NM_014521 | SH3BP4 | -1.26 |
| NM_003012 | SFRP1 | -1.26 | NM_003653 | COPS3 | -1.26 |
| NM_033377 | CGB1 | -1.26 | NM_018265 | FLJ10901 | -1.26 |
| NM_002101 | GYPC | -1.26 | NM_021967 | SERF1A | -1.26 |
| NM_014232 | VAMP2 | -1.26 | NM_004718 | COX7A2L | -1.26 |
| NM_012469 | C20ORF14 | -1.26 | NM_018106 | ZDHHC4 | -1.26 |
| NM_018135 | MRPS18A | -1.26 | NM_018142 | FLJ10569 | -1.26 |
| NM_017707 | UPLC1 | -1.26 | NM_018397 | CHDH | -1.26 |

| | | | | | |
|---|---|---|---|---|---|
| NM_017751 | FLJ20297 | -1.26 | NM_080668 | CDCA5 | -1.26 |
| NM_052956 | BUCS1 | -1.26 | XM_059776 | FKBP1C | -1.26 |
| NM_144775 | SMCR8 | -1.26 | NM_139176 | NALP7 | -1.26 |
| NM_153453 | VGLL2 | -1.26 | NM_198853 | TRIM50C | -1.26 |
| XM_114129 | LOC200159 | -1.26 | XM_293868 | LOC345537 | -1.26 |
| XM_378865 | LOC400756 | -1.26 | XM_379267 | LOC401134 | -1.26 |
| XM_379892 | LOC402567 | -1.26 | NM_002418 | MLN | -1.25 |
| NM_000070 | CAPN3 | -1.25 | NM_001614 | ACTG1 | -1.25 |
| NM_003139 | SRPR | -1.25 | NM_003239 | TGFB3 | -1.25 |
| NM_000501 | ELN | -1.25 | NM_001853 | COL9A3 | -1.25 |
| NM_014515 | CNOT2 | -1.25 | NM_005986 | SOX1 | -1.25 |
| NM_006945 | SPRR2B | -1.25 | NM_014648 | DZIP3 | -1.25 |
| NM_012254 | SLC27A5 | -1.25 | NM_016338 | IPO11 | -1.25 |
| NM_025265 | MGC2776 | -1.25 | NM_025193 | HSD3B7 | -1.25 |
| NM_032556 | IL1F10 | -1.25 | XM_064856 | LOC125893 | -1.25 |
| NM_080834 | C20ORF152 | -1.25 | NM_177986 | DSG4 | -1.25 |
| NM_139250 | CTAG1A | -1.25 | NM_152756 | AVO3 | -1.25 |
| XM_378985 | LOC284751 | -1.25 | XM_058611 | LOC150928 | -1.25 |
| XM_291643 | LOC339562 | -1.25 | XM_116396 | LOC202227 | -1.25 |
| XM_379840 | LOC402509 | -1.25 | XM_371176 | LOC388550 | -1.25 |
| NM_001005480 | OR2A2 | -1.25 | NM_000916 | OXTR | -1.24 |
| NM_016358 | IRX4 | -1.24 | NM_000856 | GUCY1A3 | -1.24 |
| NM_003701 | TNFSF11 | -1.24 | NM_175852 | DKFZP451J0118 | -1.24 |
| NM_003185 | TAF4 | -1.24 | NM_002726 | PREP | -1.24 |
| NM_004882 | CIR | -1.24 | NM_012101 | TRIM29 | -1.24 |
| NM_015652 | C10ORF12 | -1.24 | NM_017943 | FBXO34 | -1.24 |
| NM_021216 | ZNF71 | -1.24 | NM_024600 | FLJ20898 | -1.24 |
| NM_032550 | KIAA1914 | -1.24 | NM_207330 | LOC152519 | -1.24 |
| NM_173543 | FLJ32844 | -1.24 | NM_173815 | FLJ37464 | -1.24 |
| XM_294854 | LOC339209 | -1.24 | XM_293976 | LOC340120 | -1.24 |
| XM_376549 | LOC401280 | -1.24 | XM_372019 | LOC389618 | -1.24 |
| XM_377947 | LOC402281 | -1.24 | XM_373477 | LOC387715 | -1.24 |
| NM_004333 | BRAF | -1.23 | NM_004126 | GNG11 | -1.23 |
| NM_001834 | CLTB | -1.23 | NM_012090 | MACF1 | -1.23 |
| NM_144770 | RBM11 | -1.23 | NM_138693 | KLF14 | -1.23 |
| NM_030767 | AKNA | -1.23 | NM_015446 | ELYS | -1.23 |
| NM_003749 | IRS2 | -1.23 | NM_001671 | ASGR1 | -1.23 |
| NM_000183 | HADHB | -1.23 | XM_166125 | PALD | -1.23 |
| NM_003186 | TAGLN | -1.23 | NM_007363 | NONO | -1.23 |
| NM_003249 | THOP1 | -1.23 | NM_014146 | WBSCR5 | -1.23 |
| NM_006077 | CBARA1 | -1.23 | NM_015037 | KIAA0913 | -1.23 |
| NM_021025 | TLX3 | -1.23 | NM_017911 | C22ORF8 | -1.23 |
| NM_021189 | IGSF4B | -1.23 | NM_022450 | RHBDF1 | -1.23 |
| NM_144722 | FLJ23577 | -1.23 | NM_032817 | FLJ14641 | -1.23 |
| NM_138334 | SBBI54 | -1.23 | NM_153254 | FLJ36119 | -1.23 |
| NM_152776 | MGC40579 | -1.23 | XM_370777 | LOC374569 | -1.23 |

| | | | | | |
|---|---|---|---|---|---|
| NM_198699 | KRTAP18-12 | -1.23 | XM_291991 | LOC387870 | -1.23 |
| XM_059166 | LOC127602 | -1.23 | XM_070619 | LOC137814 | -1.23 |
| XM_376464 | LOC401233 | -1.23 | XM_377837 | LOC402170 | -1.23 |
| XM_373538 | LOC387869 | -1.23 | NM_033118 | MYLK2 | -1.22 |
| NM_005972 | PPYR1 | -1.22 | NM_022098 | LOC63929 | -1.22 |
| NM_004761 | RGL2 | -1.22 | NM_000201 | ICAM1 | -1.22 |
| NM_022054 | KCNK13 | -1.22 | NM_018298 | MCOLN3 | -1.22 |
| NM_005024 | SERPINB10 | -1.22 | NM_002817 | PSMD13 | -1.22 |
| XM_038520 | KIAA0542 | -1.22 | NM_006413 | RPP30 | -1.22 |
| NM_015132 | SNX13 | -1.22 | XM_375762 | NTNG1 | -1.22 |
| XM_027045 | CUTL2 | -1.22 | NM_016014 | C9ORF77 | -1.22 |
| NM_020317 | NPD014 | -1.22 | NM_024889 | C10ORF81 | -1.22 |
| NM_033111 | LOC88523 | -1.22 | NM_153214 | FLJ37440 | -1.22 |
| NM_152488 | FLJ32833 | -1.22 | NM_133473 | ZNF431 | -1.22 |
| NM_173667 | FLJ37543 | -1.22 | XM_292943 | NAP5 | -1.22 |
| XM_379507 | LOC401385 | -1.22 | XM_379934 | LOC402600 | -1.22 |
| NM_001001964 | OR2T11 | -1.22 | NM_139021 | ERK8 | -1.21 |
| NM_015129 | 38961 | -1.21 | NM_032638 | GATA2 | -1.21 |
| NM_018127 | ELAC2 | -1.21 | NM_017990 | PDPR | -1.21 |
| NM_176880 | TRA16 | -1.21 | NM_021485 | RPS6KB2 | -1.21 |
| NM_016315 | GULP1 | -1.21 | NM_005550 | KIFC3 | -1.21 |
| NM_000186 | HF1 | -1.21 | NM_012420 | IFIT5 | -1.21 |
| NM_002367 | MAGEB4 | -1.21 | NM_015462 | DKFZP586L0724 | -1.21 |
| XM_059095 | FNBP2 | -1.21 | NM_012473 | TXN2 | -1.21 |
| NM_015603 | CCDC9 | -1.21 | NM_016052 | CGI-115 | -1.21 |
| NM_016397 | TH1L | -1.21 | NM_022457 | RFWD2 | -1.21 |
| NM_080603 | ZSWIM1 | -1.21 | NM_174959 | LOC136306 | -1.21 |
| NM_145282 | LOC153328 | -1.21 | NM_181847 | AMIGO2 | -1.21 |
| NM_003510 | HIST1H2AK | -1.21 | XM_170659 | LOC254439 | -1.21 |
| XM_375816 | LOC400792 | -1.21 | XM_378799 | LOC400691 | -1.21 |
| XM_373779 | LOC388480 | -1.21 | NM_003685 | KHSRP | -1.2 |
| NM_016269 | LEF1 | -1.2 | NM_006291 | TNFAIP2 | -1.2 |
| NM_006597 | HSPA8 | -1.2 | NM_025090 | USP36 | -1.2 |
| NM_006462 | C20ORF18 | -1.2 | NM_002835 | PTPN12 | -1.2 |
| NM_003269 | NR2E1 | -1.2 | NM_003790 | TNFRSF25 | -1.2 |
| NM_005450 | NOG | -1.2 | NM_006887 | ZFP36L2 | -1.2 |
| NM_182549 | HLA-DQB2 | -1.2 | NM_002887 | RARS | -1.2 |
| NM_014666 | ENTH | -1.2 | XM_084529 | KIAA0298 | -1.2 |
| NM_006090 | CEPT1 | -1.2 | NM_006817 | C12ORF8 | -1.2 |
| NM_014315 | KLHDC2 | -1.2 | NM_014028 | OSTM1 | -1.2 |
| NM_018161 | NADSYN1 | -1.2 | NM_018923 | PCDHGB2 | -1.2 |
| NM_020135 | WRNIP1 | -1.2 | NM_020951 | ZNF529 | -1.2 |
| NM_031919 | CSDUFD1 | -1.2 | NM_015242 | CENTD2 | -1.2 |
| NM_133450 | KIAA1977 | -1.2 | NM_152418 | FLJ35775 | -1.2 |
| XM_293911 | ANKRD31 | -1.2 | NM_181624 | KRTAP23-1 | -1.2 |
| NM_198533 | SCDR10 | -1.2 | XM_373821 | LOC388586 | -1.2 |

| | | | | | |
|---|---|---|---|---|---|
| XM_372114 | LOC389757 | -1.2 | XM_374646 | LOC392979 | -1.2 |
| XM_374920 | LOC399940 | -1.2 | XM_375007 | LOC400093 | -1.2 |
| XM_372559 | LOC390558 | -1.2 | XM_370833 | LOC388076 | -1.2 |
| XM_378639 | LOC400567 | -1.2 | NM_004197 | STK19 | -1.19 |
| NM_005451 | PDLIM7 | -1.19 | NM_004427 | PHC2 | -1.19 |
| NM_003146 | SSRP1 | -1.19 | NM_002500 | NEUROD1 | -1.19 |
| NM_018584 | CAMKIINALPHA | -1.19 | NM_004574 | PNUTL2 | -1.19 |
| NM_013269 | LLT1 | -1.19 | NM_012226 | P2RX2 | -1.19 |
| NM_006920 | SCN1A | -1.19 | NM_007051 | FAF1 | -1.19 |
| NM_000517 | HBA2 | -1.19 | NM_014486 | AD7C-NTP | -1.19 |
| NM_004175 | SNRPD3 | -1.19 | NM_006672 | SLC22A7 | -1.19 |
| NM_015554 | GLCE | -1.19 | NM_012143 | TFIP11 | -1.19 |
| NM_012257 | HBP1 | -1.19 | NM_016396 | HSPC129 | -1.19 |
| NM_020750 | XPO5 | -1.19 | NM_032431 | HRD1 | -1.19 |
| NM_138392 | LOC92799 | -1.19 | NM_178540 | MGC48915 | -1.19 |
| XM_168073 | LOC221344 | -1.19 | XM_379097 | LOC400957 | -1.19 |
| XM_374013 | LOC389048 | -1.19 | XM_372468 | LOC390338 | -1.19 |
| NM_001005471 | OR2T6 | -1.19 | NM_001001790 | C9ORF105 | -1.19 |
| NM_018401 | STK32B | -1.18 | NM_002469 | MYF6 | -1.18 |
| NM_002254 | KIF3C | -1.18 | NM_173043 | IL18BP | -1.18 |
| NM_002373 | MAP1A | -1.18 | NM_005086 | SSPN | -1.18 |
| NM_017588 | WDR5 | -1.18 | NM_006189 | OMP | -1.18 |
| NM_005381 | NCL | -1.18 | NM_001021 | RPS17 | -1.18 |
| NM_182795 | NPM2 | -1.18 | NM_006663 | RAI | -1.18 |
| NM_014474 | SMPDL3B | -1.18 | NM_016102 | TRIM17 | -1.18 |
| NM_014582 | OBP2A | -1.18 | NM_032946 | NXF5 | -1.18 |
| NM_020861 | ZBTB2 | -1.18 | NM_024787 | RNF122 | -1.18 |
| XM_031009 | LOC90342 | -1.18 | XM_371079 | FBF1 | -1.18 |
| XM_375558 | KIAA1881 | -1.18 | NM_138789 | LOC120379 | -1.18 |
| XM_067585 | LOC131873 | -1.18 | NM_175733 | LOC143425 | -1.18 |
| NM_206893 | MS4A10 | -1.18 | NM_198565 | UNQ3030 | -1.18 |
| XM_379479 | LOC401317 | -1.18 | XM_380143 | LOC402592 | -1.18 |
| XM_379692 | LOC401574 | -1.18 | XM_380129 | LOC402564 | -1.18 |
| NM_004203 | PKMYT1 | -1.17 | NM_003668 | MAPKAPK5 | -1.17 |
| NM_003953 | MPZL1 | -1.17 | NM_006465 | ARID3B | -1.17 |
| NM_023918 | TAS2R8 | -1.17 | NM_000597 | IGFBP2 | -1.17 |
| NM_002588 | PCDHGC3 | -1.17 | NM_000296 | PKD1 | -1.17 |
| NM_001451 | FOXF1 | -1.17 | NM_002452 | NUDT1 | -1.17 |
| NM_004120 | GBP2 | -1.17 | NM_003925 | MBD4 | -1.17 |
| NM_000456 | SUOX | -1.17 | NM_000255 | MUT | -1.17 |
| NM_022739 | SMURF2 | -1.17 | NM_002659 | PLAUR | -1.17 |
| NM_001318 | CSHL1 | -1.17 | NM_015507 | EGFL6 | -1.17 |
| NM_002820 | PTHLH | -1.17 | NM_014580 | SLC2A8 | -1.17 |
| NM_003970 | MYOM2 | -1.17 | XM_376589 | KIAA0241 | -1.17 |
| NM_130781 | RAB24 | -1.17 | NM_016095 | PFS2 | -1.17 |
| NM_018946 | NANS | -1.17 | XM_035299 | ZSWIM6 | -1.17 |

| | | |
|---|---|---|
| NM_022127 | SLC28A3 | -1.17 |
| NM_032578 | MYPN | -1.17 |
| NM_153606 | FLJ32796 | -1.17 |
| NM_173623 | FLJ35808 | -1.17 |
| NM_174979 | C14ORF65 | -1.17 |
| XM_374302 | LOC389782 | -1.17 |
| NM_000208 | INSR | -1.16 |
| NM_007344 | TTF1 | -1.16 |
| NM_001406 | EFNB3 | -1.16 |
| NM_003625 | PPFIA2 | -1.16 |
| NM_004268 | CRSP6 | -1.16 |
| NM_032513 | SLC30A2 | -1.16 |
| NM_015227 | POFUT2 | -1.16 |
| NM_016040 | CGI-100 | -1.16 |
| NM_022763 | FNDC3B | -1.16 |
| NM_022827 | FLJ21347 | -1.16 |
| NM_182482 | BAGE2 | -1.16 |
| NM_173633 | FLJ90805 | -1.16 |
| XM_292357 | LOC342096 | -1.16 |
| NM_001005226 | OR2B3 | -1.16 |
| NM_001824 | CKM | -1.15 |
| NM_022755 | C9ORF12 | -1.15 |
| NM_005250 | FOXL1 | -1.15 |
| NM_003899 | ARHGEF7 | -1.15 |
| NM_004515 | ILF2 | -1.15 |
| NM_032045 | KREMEN1 | -1.15 |
| NM_004460 | FAP | -1.15 |
| NM_003956 | CH25H | -1.15 |
| NM_006614 | CHL1 | -1.15 |
| XM_113763 | C14ORF125 | -1.15 |
| NM_015691 | KIAA1280 | -1.15 |
| XM_373827 | FLJ20719 | -1.15 |
| NM_031206 | FLJ12525 | -1.15 |
| NM_032878 | MGC15677 | -1.15 |
| NM_022456 | RAB3IP | -1.15 |
| NM_182604 | FLJ36131 | -1.15 |
| NM_198693 | KRTAP18-2 | -1.15 |
| NM_000399 | EGR2 | -1.14 |
| NM_003259 | ICAM5 | -1.14 |
| NM_004476 | FOLH1 | -1.14 |
| NM_006109 | SKB1 | -1.14 |
| NM_001714 | BICD1 | -1.14 |
| NM_003126 | SPTA1 | -1.14 |
| NM_014298 | QPRT | -1.14 |
| NM_016080 | CGI-150 | -1.14 |
| NM_145204 | SENP8 | -1.14 |
| NM_024321 | MGC10433 | -1.17 |
| NM_144706 | C2ORF15 | -1.17 |
| XM_378314 | LOC283152 | -1.17 |
| NM_173627 | FLJ35220 | -1.17 |
| XM_061427 | LOC119358 | -1.17 |
| NM_003583 | DYRK2 | -1.16 |
| NM_001420 | ELAVL3 | -1.16 |
| NM_003450 | ZNF174 | -1.16 |
| NM_000936 | PNLIP | -1.16 |
| NM_003746 | DNCL1 | -1.16 |
| NM_005555 | KRT6B | -1.16 |
| NM_006798 | UGT2A1 | -1.16 |
| NM_015293 | SYNE1 | -1.16 |
| NM_022461 | AZ2 | -1.16 |
| NM_022475 | HHIP | -1.16 |
| XM_044212 | KIAA1862 | -1.16 |
| NM_199242 | UNC13D | -1.16 |
| XM_379432 | LOC285733 | -1.16 |
| XM_376658 | LOC401393 | -1.16 |
| XM_372205 | LOC389850 | -1.16 |
| NM_006658 | C7ORF16 | -1.15 |
| NM_002074 | GNB1 | -1.15 |
| NM_015859 | GTF2A1 | -1.15 |
| NM_006205 | PDE6H | -1.15 |
| NM_014619 | GRIK4 | -1.15 |
| NM_000725 | CACNB3 | -1.15 |
| NM_002948 | RPL15 | -1.15 |
| NM_014744 | TBC1D5 | -1.15 |
| NM_012477 | WBP1 | -1.15 |
| NM_015456 | COBRA1 | -1.15 |
| XM_290818 | SPIRE1 | -1.15 |
| NM_021220 | ZNF339 | -1.15 |
| NM_032753 | MGC15631 | -1.15 |
| NM_144995 | DHX57 | -1.15 |
| NM_182526 | FLJ33387 | -1.15 |
| XM_293599 | TMPRSS7 | -1.15 |
| XM_374730 | LOC392702 | -1.15 |
| NM_004488 | GP5 | -1.14 |
| NM_003946 | NOL3 | -1.14 |
| NM_004581 | RABGGTA | -1.14 |
| NM_002661 | PLCG2 | -1.14 |
| NM_022147 | IFRG28 | -1.14 |
| NM_015296 | DOCK9 | -1.14 |
| XM_046390 | ZNF473 | -1.14 |
| NM_182791 | FLJ32855 | -1.14 |
| XM_065166 | KIAA1957 | -1.14 |

| | | |
|---|---|---|
| NM_175921 | LOC285636 | -1.14 |
| NM_178555 | FLJ25770 | -1.14 |
| NM_207429 | FLJ45803 | -1.14 |
| XM_374586 | LOC392843 | -1.14 |
| NM_001001436 | LOC388272 | -1.14 |
| NM_000930 | PLAT | -1.13 |
| NM_031483 | ITCH | -1.13 |
| NM_002264 | KPNA1 | -1.13 |
| NM_021078 | GCN5L2 | -1.13 |
| NM_005745 | BCAP31 | -1.13 |
| NM_015158 | ANKRD15 | -1.13 |
| NM_014596 | ZNRD1 | -1.13 |
| NM_019617 | GKN1 | -1.13 |
| NM_030953 | TIGD6 | -1.13 |
| XM_375629 | DKFZP434J0226 | -1.13 |
| NM_153689 | FLJ38973 | -1.13 |
| NM_203481 | MGC70870 | -1.13 |
| XM_374625 | LOC392943 | -1.13 |
| NM_001004737 | OR5K2 | -1.13 |
| NM_003617 | RGS5 | -1.12 |
| NM_030752 | TCP1 | -1.12 |
| NM_024607 | PPP1R3B | -1.12 |
| NM_000734 | CD3Z | -1.12 |
| NM_000119 | EPB42 | -1.12 |
| NM_017977 | AIM1L | -1.12 |
| NM_006969 | ZNF28 | -1.12 |
| NM_016407 | C20ORF43 | -1.12 |
| NM_018144 | SEC61A2 | -1.12 |
| NM_017876 | RNF126 | -1.12 |
| NM_020404 | CD164L1 | -1.12 |
| NM_022497 | MRPS25 | -1.12 |
| NM_198150 | DKFZP313G1735 | -1.12 |
| XM_293937 | LOC345711 | -1.12 |
| XM_380098 | LOC402475 | -1.12 |
| NM_018208 | FLJ10761 | -1.11 |
| NM_000707 | AVPR1B | -1.11 |
| NM_005569 | LIMK2 | -1.11 |
| NM_006564 | CXCR6 | -1.11 |
| NM_176888 | TAS2R48 | -1.11 |
| NM_018898 | PCDHAC1 | -1.11 |
| NM_000248 | MITF | -1.11 |
| NM_014746 | RNF144 | -1.11 |
| NM_002085 | GPX4 | -1.11 |
| NM_030663 | MCSP | -1.11 |
| NM_014742 | TM9SF4 | -1.11 |
| NM_007230 | MAN1B1 | -1.11 |
| NM_173697 | FLJ33610 | -1.14 |
| NM_207393 | UNQ483 | -1.14 |
| XM_377786 | LOC402120 | -1.14 |
| XM_373713 | LOC388334 | -1.14 |
| NM_022170 | WBSCR1 | -1.13 |
| NM_139025 | ADAMTS13 | -1.13 |
| NM_019009 | TOLLIP | -1.13 |
| NM_005736 | ACTR1A | -1.13 |
| NM_003984 | SLC13A2 | -1.13 |
| NM_207304 | MBNL2 | -1.13 |
| NM_015945 | SLC35C2 | -1.13 |
| NM_016559 | PEX5R | -1.13 |
| NM_031953 | SNX25 | -1.13 |
| NM_024628 | SLC12A8 | -1.13 |
| NM_198445 | FLJ45909 | -1.13 |
| XM_290615 | DKFZP762F0713 | -1.13 |
| XM_374226 | LOC389533 | -1.13 |
| XM_379696 | LOC401579 | -1.13 |
| NM_012247 | SEPHS1 | -1.12 |
| NM_001541 | HSPB2 | -1.12 |
| NM_000963 | PTGS2 | -1.12 |
| NM_001501 | GNRH2 | -1.12 |
| NM_001147 | ANGPT2 | -1.12 |
| NM_004493 | HADH2 | -1.12 |
| NM_005033 | EXOSC9 | -1.12 |
| NM_172020 | POM121 | -1.12 |
| NM_018983 | NOLA1 | -1.12 |
| NM_018178 | GPP34R | -1.12 |
| NM_018326 | HIMAP4 | -1.12 |
| NM_020453 | ATP10D | -1.12 |
| NM_025091 | CXYORF2 | -1.12 |
| NM_207366 | FLJ44060 | -1.12 |
| XM_294688 | LOC338731 | -1.12 |
| XM_379899 | LOC402572 | -1.12 |
| NM_016082 | CDK5RAP1 | -1.11 |
| NM_004444 | EPHB4 | -1.11 |
| NM_002880 | RAF1 | -1.11 |
| NM_020377 | CYSLTR2 | -1.11 |
| XM_371933 | PPP1R9A | -1.11 |
| NM_003802 | MYH13 | -1.11 |
| NM_003408 | ZFP37 | -1.11 |
| NM_000518 | HBB | -1.11 |
| NM_000783 | CYP26A1 | -1.11 |
| NM_006054 | RTN3 | -1.11 |
| NM_006876 | B3GNT6 | -1.11 |
| NM_178225 | FBXW5 | -1.11 |

| | | | | | |
|---|---|---|---|---|---|
| NM_019008 | FLJ20232 | -1.11 | NM_017733 | FLJ20265 | -1.11 |
| NM_018155 | FLJ10618 | -1.11 | NM_021173 | POLD4 | -1.11 |
| NM_023937 | MRPL34 | -1.11 | NM_032325 | MGC11102 | -1.11 |
| XM_059468 | MDH1B | -1.11 | XM_370944 | LOC146177 | -1.11 |
| NM_174914 | LOC167127 | -1.11 | NM_130901 | C15ORF16 | -1.11 |
| NM_078483 | SLC36A1 | -1.11 | XM_374020 | LOC375295 | -1.11 |
| NM_194249 | DND1 | -1.11 | XM_069842 | LOC136321 | -1.11 |
| XM_377566 | LOC401940 | -1.11 | XM_374101 | LOC389250 | -1.11 |
| XM_374270 | LOC389659 | -1.11 | XM_372543 | LOC390530 | -1.11 |
| NM_004714 | DYRK1B | -1.1 | NM_002024 | FMR1 | -1.1 |
| NM_005641 | TAF6 | -1.1 | NM_014943 | ZHX2 | -1.1 |
| NM_002912 | REV3L | -1.1 | NM_000140 | FECH | -1.1 |
| NM_014871 | USP52 | -1.1 | NM_145251 | STYX | -1.1 |
| NM_000564 | IL5RA | -1.1 | NM_006211 | PENK | -1.1 |
| NM_005761 | PLXNC1 | -1.1 | NM_003355 | UCP2 | -1.1 |
| NM_001664 | RHOA | -1.1 | NM_020350 | AGTRAP | -1.1 |
| NM_000067 | CA2 | -1.1 | NM_000331 | SAA1 | -1.1 |
| NM_006552 | SCGB1D1 | -1.1 | NM_002080 | GOT2 | -1.1 |
| NM_004793 | PRSS15 | -1.1 | NM_199131 | VAX1 | -1.1 |
| NM_015213 | RAB6IP1 | -1.1 | NM_018070 | SSBP3 | -1.1 |
| NM_022006 | FXYD7 | -1.1 | NM_018264 | FLJ10900 | -1.1 |
| NM_017879 | FLJ20557 | -1.1 | NM_020169 | LXN | -1.1 |
| NM_019100 | DMAP1 | -1.1 | NM_020214 | LOC56965 | -1.1 |
| NM_020644 | C11ORF15 | -1.1 | NM_020710 | KIAA1185 | -1.1 |
| NM_021188 | ZNF410 | -1.1 | NM_024882 | C6ORF155 | -1.1 |
| NM_152603 | ZNF567 | -1.1 | NM_181536 | PKD1L3 | -1.1 |
| XM_028217 | LOC90024 | -1.1 | XM_378883 | LOC400768 | -1.1 |
| XM_371535 | LOC389002 | -1.1 | XM_379818 | LOC402488 | -1.1 |
| NM_001004348 | FLJ16171 | -1.1 | NM_000686 | AGTR2 | -1.09 |
| NM_001300 | COPEB | -1.09 | NM_003923 | FOXH1 | -1.09 |
| NM_001266 | CES1 | -1.09 | NM_003442 | ZNF143 | -1.09 |
| NM_176783 | PSME1 | -1.09 | NM_001155 | ANXA6 | -1.09 |
| NM_005583 | LYL1 | -1.09 | NM_006341 | MAD2L2 | -1.09 |
| NM_005278 | GPM6B | -1.09 | NM_012084 | GLUD2 | -1.09 |
| NM_006057 | B3GALT5 | -1.09 | NM_014913 | KIAA0863 | -1.09 |
| XM_038288 | ZCCHC11 | -1.09 | NM_007364 | P24B | -1.09 |
| NM_080730 | HOM-TES-103 | -1.09 | XM_058628 | C14ORF109 | -1.09 |
| NM_018722 | KCNQ1DN | -1.09 | NM_178817 | C21ORF61 | -1.09 |
| NM_021825 | MDS025 | -1.09 | NM_032577 | LOC84664 | -1.09 |
| NM_032788 | ZNF514 | -1.09 | NM_138391 | C1ORF37 | -1.09 |
| NM_080745 | RNF36 | -1.09 | XM_086996 | LOC150763 | -1.09 |
| NM_178569 | CEI | -1.09 | NM_153268 | FLJ31579 | -1.09 |
| NM_182757 | IBRDC2 | -1.09 | NM_207449 | FLJ44674 | -1.09 |
| NM_004981 | KCNJ4 | -1.09 | XM_116036 | LOC200959 | -1.09 |
| XM_208319 | LOC285412 | -1.09 | XM_377882 | LOC402205 | -1.09 |
| XM_378411 | LOC400123 | -1.09 | NM_001001667 | OR6V1 | -1.09 |

| | | |
|---|---|---|
| NM_005241 | EVI1 | -1.08 |
| NM_006480 | RGS14 | -1.08 |
| NM_012182 | FOXB1 | -1.08 |
| NM_003196 | TCEA3 | -1.08 |
| NM_139018 | NKIR | -1.08 |
| NM_000214 | JAG1 | -1.08 |
| NM_000370 | TTPA | -1.08 |
| NM_005510 | DOM3Z | -1.08 |
| NM_006814 | PSMF1 | -1.08 |
| NM_016300 | ARPP-21 | -1.08 |
| NM_012137 | DDAH1 | -1.08 |
| NM_024627 | FLJ21125 | -1.08 |
| NM_145234 | CHRDL1 | -1.08 |
| NM_033420 | C19ORF6 | -1.08 |
| NM_144690 | ZNF582 | -1.08 |
| NM_175616 | FIS | -1.08 |
| XM_208731 | C14ORF68 | -1.08 |
| NM_178545 | LOC339456 | -1.08 |
| NM_198686 | RAB15 | -1.08 |
| XM_292796 | LOC342931 | -1.08 |
| XM_377970 | LOC402295 | -1.08 |
| XM_380120 | LOC402537 | -1.08 |
| XM_372668 | LOC390790 | -1.08 |
| NM_002968 | SALL1 | -1.07 |
| NM_016086 | MK-STYX | -1.07 |
| NM_003786 | ABCC3 | -1.07 |
| NM_001696 | ATP6V1E1 | -1.07 |
| NM_033133 | CNP | -1.07 |
| NM_020783 | SYT4 | -1.07 |
| NM_014385 | SIGLEC7 | -1.07 |
| NM_017622 | FLJ20014 | -1.07 |
| NM_024731 | C16ORF44 | -1.07 |
| NM_032140 | DKFZP434A1319 | -1.07 |
| XM_059929 | LOC137886 | -1.07 |
| NM_145034 | LOC163590 | -1.07 |
| NM_198469 | C9ORF18 | -1.07 |
| NM_207646 | LOC400696 | -1.07 |
| NM_003684 | MKNK1 | -1.06 |
| NM_001427 | EN2 | -1.06 |
| NM_003418 | ZNF9 | -1.06 |
| NM_022552 | DNMT3A | -1.06 |
| NM_006676 | USP20 | -1.06 |
| NM_002894 | RBBP8 | -1.06 |
| NM_003549 | HYAL3 | -1.06 |
| XM_371417 | KIAA0179 | -1.06 |
| NM_018091 | ELP3 | -1.06 |
| NM_000054 | AVPR2 | -1.08 |
| NM_004697 | PRPF4 | -1.08 |
| NM_002200 | IRF5 | -1.08 |
| NM_003907 | EIF2B5 | -1.08 |
| NM_002619 | PF4 | -1.08 |
| NM_000900 | MGP | -1.08 |
| NM_000830 | GRIK1 | -1.08 |
| NM_002442 | MSI1 | -1.08 |
| NM_005829 | AP3S2 | -1.08 |
| XM_375375 | KIAA0431 | -1.08 |
| NM_012459 | TIMM8B | -1.08 |
| NM_024989 | PGAP1 | -1.08 |
| NM_080659 | MGC14839 | -1.08 |
| NM_198451 | FOXR2 | -1.08 |
| NM_173527 | FLJ38964 | -1.08 |
| NM_173358 | SSX7 | -1.08 |
| NM_182573 | FLJ30469 | -1.08 |
| NM_182603 | FLJ37874 | -1.08 |
| NM_023002 | HAPLN4 | -1.08 |
| XM_372789 | LOC391088 | -1.08 |
| XM_378259 | LOC399832 | -1.08 |
| XM_370895 | LOC388163 | -1.08 |
| NM_001001343 | MGC27121 | -1.08 |
| NM_021958 | HLX1 | -1.07 |
| NM_000832 | GRIN1 | -1.07 |
| NM_031892 | SH3KBP1 | -1.07 |
| XM_290552 | CNGA4 | -1.07 |
| NM_002151 | HPN | -1.07 |
| NM_014932 | NLGN1 | -1.07 |
| NM_031890 | CECR6 | -1.07 |
| NM_020650 | RCN3 | -1.07 |
| NM_025215 | PUS1 | -1.07 |
| NM_032175 | FLJ12787 | -1.07 |
| XM_379260 | LOC152742 | -1.07 |
| NM_145016 | BXMAS2-10 | -1.07 |
| NM_153003 | OFCC1 | -1.07 |
| XM_372769 | LOC391013 | -1.07 |
| NM_004724 | ZW10 | -1.06 |
| NM_005596 | NFIB | -1.06 |
| NM_017436 | A4GALT | -1.06 |
| NM_000601 | HGF | -1.06 |
| NM_003584 | DUSP11 | -1.06 |
| NM_173714 | MTND6 | -1.06 |
| NM_014708 | KNTC1 | -1.06 |
| NM_016127 | MGC8721 | -1.06 |
| NM_017787 | C10ORF26 | -1.06 |

| | | | | | |
|---|---|---|---|---|---|
| NM_021224 | ZNF462 | -1.06 | NM_032620 | GTPBP3 | -1.06 |
| NM_178450 | 37681 | -1.06 | NM_172347 | KCNG4 | -1.06 |
| NM_152573 | RASEF | -1.06 | NM_198467 | FLJ42526 | -1.06 |
| NM_016456 | TMEM9 | -1.06 | NM_199461 | NANOS1 | -1.06 |
| XM_115925 | LOC203604 | -1.06 | XM_374189 | LOC389441 | -1.06 |
| NM_001001330 | C10ORF74 | -1.06 | NM_002498 | NEK3 | -1.05 |
| NM_003893 | LDB1 | -1.05 | NM_004926 | ZFP36L1 | -1.05 |
| NM_001097 | ACR | -1.05 | NM_005658 | TRAF1 | -1.05 |
| NM_000928 | PLA2G1B | -1.05 | NM_004884 | PUNC | -1.05 |
| NM_001092 | ABR | -1.05 | NM_002867 | RAB3B | -1.05 |
| NM_000828 | GRIA3 | -1.05 | NM_005516 | HLA-E | -1.05 |
| NM_000833 | GRIN2A | -1.05 | NM_001652 | AQP6 | -1.05 |
| NM_182615 | MGC40069 | -1.05 | NM_005966 | NAB1 | -1.05 |
| NM_005147 | DNAJA3 | -1.05 | NM_016090 | RBM7 | -1.05 |
| NM_015176 | FBXO28 | -1.05 | NM_015912 | LOC51059 | -1.05 |
| NM_018049 | PLEKHJ1 | -1.05 | NM_020684 | NPD007 | -1.05 |
| NM_024786 | ZDHHC11 | -1.05 | NM_024836 | FLJ22301 | -1.05 |
| NM_025029 | FLJ14346 | -1.05 | NM_031459 | SESN2 | -1.05 |
| NM_032740 | MGC5391 | -1.05 | NM_033083 | EAF1 | -1.05 |
| NM_198440 | C22ORF14 | -1.05 | NM_205767 | QIL1 | -1.05 |
| XM_376718 | FLJ45737 | -1.05 | NM_173635 | FLJ40235 | -1.05 |
| NM_207376 | LOC387882 | -1.05 | XM_293971 | LOC345778 | -1.05 |
| XM_373038 | TRIM61 | -1.05 | NM_003427 | ZNF76 | -1.04 |
| NM_002148 | HOXD10 | -1.04 | NM_002454 | MTRR | -1.04 |
| NM_004994 | MMP9 | -1.04 | NM_002504 | NFX1 | -1.04 |
| NM_005188 | CBL | -1.04 | NM_021006 | CCL3L1 | -1.04 |
| NM_005120 | TNRC11 | -1.04 | NM_001298 | CNGA3 | -1.04 |
| NM_014208 | DSPP | -1.04 | NM_021922 | FANCE | -1.04 |
| NM_000326 | RLBP1 | -1.04 | NM_002792 | PSMA7 | -1.04 |
| NM_130777 | GAGED3 | -1.04 | NM_006594 | AP4B1 | -1.04 |
| NM_006781 | C6ORF10 | -1.04 | NM_018204 | CKAP2 | -1.04 |
| NM_016535 | ZNF581 | -1.04 | NM_032186 | KIAA1221 | -1.04 |
| NM_058187 | C21ORF63 | -1.04 | NM_032859 | C13ORF6 | -1.04 |
| NM_033495 | KLHL13 | -1.04 | NM_194278 | C14ORF43 | -1.04 |
| NM_130830 | LRRC15 | -1.04 | NM_182533 | FLJ31031 | -1.04 |
| NM_182572 | ZSCAN1 | -1.04 | NM_199134 | LOC283970 | -1.04 |
| XM_063084 | LOC122335 | -1.04 | NM_178546 | LOC339483 | -1.04 |
| XM_294540 | LOC340485 | -1.04 | XM_293396 | LOC347512 | -1.04 |
| XM_377928 | LOC402247 | -1.04 | XM_379648 | LOC401539 | -1.04 |
| XM_374296 | LOC389771 | -1.04 | XM_376909 | LOC401551 | -1.04 |
| XM_370537 | LOC387643 | -1.04 | XM_373748 | LOC388418 | -1.04 |
| XM_378750 | LOC400653 | -1.04 | NM_006254 | PRKCD | -1.03 |
| NM_004606 | TAF1 | -1.03 | NM_006280 | SSR4 | -1.03 |
| NM_002049 | GATA1 | -1.03 | NM_002142 | HOXA9 | -1.03 |
| NM_014589 | PLA2G2E | -1.03 | NM_012167 | FBXO11 | -1.03 |
| NM_152306 | UHRF2 | -1.03 | NM_001611 | ACP5 | -1.03 |

| | | | | | |
|---|---|---|---|---|---|
| NM_014443 | IL17B | -1.03 | NM_002042 | GABRR1 | -1.03 |
| NM_006981 | NR4A3 | -1.03 | NM_000421 | KRT10 | -1.03 |
| NM_003571 | BFSP2 | -1.03 | NM_018451 | CENPJ | -1.03 |
| NM_002288 | LAIR2 | -1.03 | NM_020432 | PHTF2 | -1.03 |
| NM_005493 | RANBP9 | -1.03 | NM_006701 | TXNL4 | -1.03 |
| XM_051091 | KIAA1040 | -1.03 | NM_014149 | HSPC049 | -1.03 |
| NM_015860 | HUMRTVLH3 | -1.03 | NM_017638 | FLJ20045 | -1.03 |
| NM_017652 | ZNF586 | -1.03 | NM_020962 | NOPE | -1.03 |
| NM_024820 | KIAA1608 | -1.03 | NM_022165 | LIN7B | -1.03 |
| NM_024540 | MRPL24 | -1.03 | NM_024781 | C18ORF14 | -1.03 |
| NM_031439 | SOX7 | -1.03 | NM_080663 | MGC16943 | -1.03 |
| NM_153343 | ENPP6 | -1.03 | NM_207331 | LOC153561 | -1.03 |
| NM_153357 | SLC16A11 | -1.03 | XM_090294 | FLJ38508 | -1.03 |
| NM_182587 | C2ORF21 | -1.03 | NM_198568 | GJB7 | -1.03 |
| NM_182632 | SLC6A18 | -1.03 | NM_001005241 | OR4N4 | -1.03 |
| NM_207118 | C6ORF175 | -1.03 | XM_290734 | LOC339162 | -1.03 |
| XM_067904 | LOC132556 | -1.03 | XM_371638 | LOC389117 | -1.03 |
| XM_374004 | LOC389025 | -1.03 | XM_376822 | LOC401497 | -1.03 |
| NM_001002847 | DERPC | -1.03 | NM_006182 | DDR2 | -1.02 |
| NM_003851 | CREG | -1.02 | NM_021961 | TEAD1 | -1.02 |
| NM_005504 | BCAT1 | -1.02 | NM_000155 | GALT | -1.02 |
| NM_007101 | SARDH | -1.02 | NM_020159 | SMARCAD1 | -1.02 |
| NM_007027 | TOPBP1 | -1.02 | NM_004655 | AXIN2 | -1.02 |
| NM_007039 | PTPN21 | -1.02 | NM_014448 | ARHGEF16 | -1.02 |
| NM_005717 | ARPC5 | -1.02 | NM_138296 | PTCRA | -1.02 |
| NM_021021 | SNTB1 | -1.02 | NM_007006 | CPSF5 | -1.02 |
| NM_170678 | ITGB1BP3 | -1.02 | NM_017982 | FLJ10052 | -1.02 |
| NM_018245 | OGDHL | -1.02 | NM_018270 | C20ORF20 | -1.02 |
| NM_031418 | TMEM16C | -1.02 | NM_024807 | TREML2 | -1.02 |
| NM_030576 | MGC10986 | -1.02 | NM_024864 | FLJ22578 | -1.02 |
| NM_031446 | C18ORF21 | -1.02 | NM_032290 | ANKRD32 | -1.02 |
| NM_052859 | RFT1 | -1.02 | NM_152340 | FLJ39075 | -1.02 |
| NM_144588 | ZFYVE27 | -1.02 | XM_088142 | C7ORF32 | -1.02 |
| NM_015447 | CAMSAP1 | -1.02 | XM_095568 | DKFZP762C1112 | -1.02 |
| NM_153450 | LCMR1 | -1.02 | NM_145720 | TIGD4 | -1.02 |
| NM_147129 | FLJ36525 | -1.02 | NM_198534 | FLJ35784 | -1.02 |
| XM_293821 | LOC345456 | -1.02 | XM_371305 | LOC388688 | -1.02 |
| XM_374003 | LOC389024 | -1.02 | XM_379146 | LOC401021 | -1.02 |
| XM_371402 | LOC388799 | -1.02 | XM_372227 | LOC389866 | -1.02 |
| NM_020990 | CKMT1 | -1.01 | NM_000020 | ACVRL1 | -1.01 |
| NM_033141 | MAP3K9 | -1.01 | NM_002461 | MVD | -1.01 |
| NM_003177 | SYK | -1.01 | NM_023005 | BAZ1B | -1.01 |
| NM_013391 | DMGDH | -1.01 | NM_002424 | MMP8 | -1.01 |
| NM_002547 | OPHN1 | -1.01 | NM_014948 | UBCE7IP5 | -1.01 |
| NM_139283 | TA-PP2C | -1.01 | NM_003473 | STAM | -1.01 |
| NM_006070 | TFG | -1.01 | NM_004525 | LRP2 | -1.01 |

| | | | | | |
|---|---|---|---|---|---|
| NM_005675 | DGCR6 | -1.01 | NM_001133 | AFM | -1.01 |
| NM_001865 | COX7A2 | -1.01 | NM_005669 | C5orf18 | -1.01 |
| NM_014676 | PUM1 | -1.01 | XM_088459 | KIAA0310 | -1.01 |
| NM_014147 | HSPC047 | -1.01 | NM_016120 | RNF12 | -1.01 |
| NM_016625 | MGC12197 | -1.01 | NM_020789 | IGSF9 | -1.01 |
| NM_020805 | KLHL14 | -1.01 | NM_022902 | SLC30A5 | -1.01 |
| XM_372124 | ZCCHC6 | -1.01 | NM_025232 | C8ORF20 | -1.01 |
| NM_030793 | FBXO38 | -1.01 | NM_031472 | MGC11134 | -1.01 |
| NM_032569 | N-PAC | -1.01 | XM_166529 | GLCCI1 | -1.01 |
| NM_153446 | GALGT2 | -1.01 | NM_152357 | ZNF440 | -1.01 |
| NM_080827 | WFDC6 | -1.01 | NM_144677 | MGAT5B | -1.01 |
| NM_207374 | UNQ6469 | -1.01 | NM_199338 | FLJ35171 | -1.01 |
| XM_096885 | LOC145853 | -1.01 | XM_212319 | LOC286441 | -1.01 |
| XM_372775 | LOC391025 | -1.01 | XM_377884 | LOC402207 | -1.01 |
| XM_379406 | LOC401254 | -1.01 | NM_001699 | AXL | -1 |
| NM_152835 | LOC149420 | -1 | NM_005301 | GPR35 | -1 |
| NM_004356 | CD81 | -1 | NM_004680 | CDY1 | -1 |
| NM_012383 | OSTF1 | -1 | NM_002178 | IGFBP6 | -1 |
| NM_002196 | INSM1 | -1 | NM_005230 | ELK3 | -1 |
| NM_001610 | ACP2 | -1 | NM_005932 | MIPEP | -1 |
| NM_004253 | PLAA | -1 | NM_005276 | GPD1 | -1 |
| NM_138793 | ENTPD8 | -1 | X53943 | HSSUCCDH | -1 |
| NM_001801 | CDO1 | -1 | NM_152736 | ZNF187 | -1 |
| NM_014231 | VAMP1 | -1 | NM_005871 | SMNDC1 | -1 |
| NM_007236 | CHP | -1 | NM_014907 | FRMPD1 | -1 |
| XM_166213 | DTX4 | -1 | NM_016458 | LOC51236 | -1 |
| NM_016484 | LOC51248 | -1 | NM_016546 | C1RL | -1 |
| NM_014603 | HUMPPA | -1 | NM_017660 | P66ALPHA | -1 |
| NM_018322 | C6ORF64 | -1 | NM_024334 | MGC3222 | -1 |
| NM_032486 | MGC3248 | -1 | NM_032332 | MGC4238 | -1 |
| XM_370567 | KIAA1975 | -1 | NM_194302 | DKFZP434O0527 | -1 |
| NM_207504 | FLJ46365 | -1 | XM_063871 | LOC123855 | -1 |
| XM_379108 | LOC400969 | -1 | XM_379458 | LOC401287 | -1 |
| XM_377832 | LOC402166 | -1 | XM_373553 | LOC387895 | -1 |
| XM_378682 | LOC400602 | -1 | XM_373772 | LOC388459 | -1 |
| XM_379879 | LOC402558 | -1 | NM_006301 | MAP3K12 | -0.99 |
| NM_012360 | OR1F1 | -0.99 | NM_004134 | HSPA9B | -0.99 |
| NM_018902 | PCDHA11 | -0.99 | NM_006709 | BAT8 | -0.99 |
| NM_000353 | TAT | -0.99 | NM_022337 | RAB38 | -0.99 |
| NM_006239 | PPEF2 | -0.99 | NM_002562 | P2RX7 | -0.99 |
| NM_021072 | HCN1 | -0.99 | NM_006430 | CCT4 | -0.99 |
| NM_006329 | FBLN5 | -0.99 | NM_005892 | FMNL1 | -0.99 |
| NM_005015 | OXA1L | -0.99 | NM_006298 | ZNF192 | -0.99 |
| NM_014279 | OLFM1 | -0.99 | NM_020470 | YIF1 | -0.99 |
| NM_024979 | MCF2L | -0.99 | NM_018210 | FLJ10769 | -0.99 |
| NM_020180 | BRUNOL4 | -0.99 | NM_030807 | SLC2A11 | -0.99 |

| | | | | | |
|---|---|---|---|---|---|
| NM_024026 | MRP63 | -0.99 | NM_024595 | FLJ12666 | -0.99 |
| NM_024102 | MEP50 | -0.99 | NM_031449 | DKFZP761I2123 | -0.99 |
| NM_032272 | MAF1 | -0.99 | XM_352953 | MGC10646 | -0.99 |
| NM_144605 | FLJ25410 | -0.99 | NM_152312 | GYLTL1B | -0.99 |
| NM_207015 | LOC254827 | -0.99 | NM_207342 | MGC87042 | -0.99 |
| NM_198553 | FLJ30851 | -0.99 | NM_182616 | MGC61550 | -0.99 |
| NM_207469 | UNQ827 | -0.99 | XM_377830 | DUB1A | -0.99 |
| XM_085138 | LOC145414 | -0.99 | XM_373840 | LOC388623 | -0.99 |
| XM_374893 | LOC399904 | -0.99 | NM_001495 | GFRA2 | -0.98 |
| NM_016457 | PRKD2 | -0.98 | NM_002626 | PFKL | -0.98 |
| NM_000674 | ADORA1 | -0.98 | NM_005294 | GPR21 | -0.98 |
| NM_002589 | PCDH7 | -0.98 | NM_003461 | ZYX | -0.98 |
| NM_172208 | TAPBP | -0.98 | NM_003869 | CES2 | -0.98 |
| NM_004408 | DNM1 | -0.98 | NM_005361 | MAGEA2 | -0.98 |
| NM_007161 | LST1 | -0.98 | XM_038604 | UNC13A | -0.98 |
| NM_020393 | PGLYRP4 | -0.98 | NM_032138 | KBTBD7 | -0.98 |
| NM_020856 | ZNF537 | -0.98 | XM_041126 | KIAA1486 | -0.98 |
| NM_023933 | MGC2494 | -0.98 | NM_025069 | FLJ14299 | -0.98 |
| NM_032744 | C6ORF105 | -0.98 | XM_373433 | LOC90379 | -0.98 |
| NM_173471 | SLC25A26 | -0.98 | NM_138808 | LOC132200 | -0.98 |
| NM_173597 | FLJ37587 | -0.98 | NM_173822 | MGC39518 | -0.98 |
| NM_207470 | FLJ45832 | -0.98 | XM_374094 | LOC389237 | -0.98 |
| XM_371798 | LOC389366 | -0.98 | XM_379962 | LOC402620 | -0.98 |
| XM_372310 | LOC389953 | -0.98 | XM_373362 | LOC392517 | -0.98 |
| NM_001004303 | LOC199920 | -0.98 | NM_015133 | MAPK8IP3 | -0.97 |
| NM_022128 | RBKS | -0.97 | NM_005417 | SRC | -0.97 |
| NM_002023 | FMOD | -0.97 | NM_005595 | NFIA | -0.97 |
| NM_014237 | ADAM18 | -0.97 | NM_005343 | HRAS | -0.97 |
| NM_003560 | PLA2G6 | -0.97 | NM_006290 | TNFAIP3 | -0.97 |
| NM_005983 | SKP2 | -0.97 | NM_006678 | CMRF35 | -0.97 |
| NM_003712 | PPAP2C | -0.97 | NM_005426 | TP53BP2 | -0.97 |
| NM_004004 | GJB2 | -0.97 | NM_003504 | CDC45L | -0.97 |
| NM_020465 | NDRG4 | -0.97 | NM_001697 | ATP5O | -0.97 |
| NM_014905 | GLS | -0.97 | NM_014623 | MEA | -0.97 |
| NM_018055 | NODAL | -0.97 | NM_006007 | ZNF216 | -0.97 |
| NM_006634 | VAMP5 | -0.97 | NM_012149 | DUX5 | -0.97 |
| NM_016570 | PTX1 | -0.97 | NM_016201 | AMOTL2 | -0.97 |
| NM_016464 | HSPC196 | -0.97 | NM_017916 | FLJ20643 | -0.97 |
| NM_017730 | FLJ20259 | -0.97 | NM_020642 | C11ORF17 | -0.97 |
| NM_022368 | PJA1 | -0.97 | NM_032169 | FLJ12592 | -0.97 |
| NM_031465 | MGC13204 | -0.97 | NM_032770 | MGC16291 | -0.97 |
| NM_032795 | FLJ14494 | -0.97 | NM_032718 | MGC11332 | -0.97 |
| NM_080926 | LOC96610 | -0.97 | NM_173466 | DKFZP434P055 | -0.97 |
| NM_080389 | DEFB104 | -0.97 | NM_213602 | CD33L3 | -0.97 |
| NM_178558 | FLJ90430 | -0.97 | XM_290848 | LOC339344 | -0.97 |
| XM_372647 | LOC348180 | -0.97 | XM_298151 | LOC344595 | -0.97 |

| | | | | | |
|---|---|---|---|---|---|
| XM_378835 | LOC400733 | -0.97 | XM_372028 | LOC389630 | -0.97 |
| XM_374275 | LOC389671 | -0.97 | XM_373693 | LOC388295 | -0.97 |
| NM_001135 | AGC1 | -0.96 | NM_006237 | POU4F1 | -0.96 |
| NM_007122 | USF1 | -0.96 | NM_004740 | TIAF1 | -0.96 |
| NM_014520 | MYBBP1A | -0.96 | NM_002842 | PTPRH | -0.96 |
| NM_152511 | DUSP18 | -0.96 | NM_001644 | APOBEC1 | -0.96 |
| NM_003119 | SPG7 | -0.96 | NM_002290 | LAMA4 | -0.96 |
| NM_004334 | BST1 | -0.96 | NM_005689 | ABCB6 | -0.96 |
| NM_003130 | SRI | -0.96 | NM_016196 | RBM19 | -0.96 |
| NM_006645 | STARD10 | -0.96 | NM_014926 | SLITRK3 | -0.96 |
| NM_014335 | CRI1 | -0.96 | NM_017753 | PRG-3 | -0.96 |
| NM_017878 | HRASLS2 | -0.96 | NM_020121 | UGCGL2 | -0.96 |
| NM_018428 | HCA66 | -0.96 | NM_145814 | CACNG6 | -0.96 |
| NM_024654 | FLJ23323 | -0.96 | NM_031211 | LAT1-3TM | -0.96 |
| NM_032921 | MGC15875 | -0.96 | NM_053045 | MGC14327 | -0.96 |
| NM_080869 | WFDC12 | -0.96 | NM_152538 | IGSF11 | -0.96 |
| NM_145293 | LOC196549 | -0.96 | NM_203301 | FBXO33 | -0.96 |
| NM_198482 | LOC284948 | -0.96 | NM_173669 | FLJ34047 | -0.96 |
| XM_379650 | LOC340515 | -0.96 | NM_181688 | KRTAP10-10 | -0.96 |
| NM_201589 | MAFA | -0.96 | XM_291625 | LOC374947 | -0.96 |
| XM_379180 | LOC401056 | -0.96 | XM_374090 | LOC389232 | -0.96 |
| XM_370668 | LOC387825 | -0.96 | XM_373497 | LOC387763 | -0.96 |
| NM_001001786 | BRCC2 | -0.96 | NM_152534 | FLJ32685 | -0.95 |
| NM_004443 | EPHB3 | -0.95 | NM_002720 | PPP4C | -0.95 |
| NM_019106 | 37865 | -0.95 | NM_006163 | NFE2 | -0.95 |
| NM_000480 | AMPD3 | -0.95 | NM_000819 | GART | -0.95 |
| NM_032673 | NSPC1 | -0.95 | NM_001178 | ARNTL | -0.95 |
| NM_178813 | AKAP28 | -0.95 | NM_016952 | CDON | -0.95 |
| NM_001914 | CYB5 | -0.95 | NM_005863 | NET1 | -0.95 |
| NM_014230 | SRP68 | -0.95 | NM_014328 | RUSC1 | -0.95 |
| NM_013237 | PX19 | -0.95 | NM_019029 | CPVL | -0.95 |
| NM_017904 | FLJ20619 | -0.95 | NM_020170 | LOC56926 | -0.95 |
| NM_018671 | SMAP-1 | -0.95 | NM_020428 | CTL2 | -0.95 |
| XM_168030 | ZNF319 | -0.95 | NM_024541 | C10ORF76 | -0.95 |
| NM_025207 | PP591 | -0.95 | NM_032279 | DKFZP761I1011 | -0.95 |
| NM_032509 | LOC84549 | -0.95 | XM_376590 | LOC89231 | -0.95 |
| NM_033284 | TBL1Y | -0.95 | NM_033626 | JM11 | -0.95 |
| NM_052952 | DIRC1 | -0.95 | NM_144681 | FLJ32734 | -0.95 |
| NM_153354 | MGC33214 | -0.95 | NM_145008 | FLJ30213 | -0.95 |
| XM_378964 | LOC339593 | -0.95 | NM_207380 | FLJ43339 | -0.95 |
| NM_207439 | FLJ46358 | -0.95 | XM_063123 | LOC122423 | -0.95 |
| XM_059956 | LOC138255 | -0.95 | XM_376619 | LOC401357 | -0.95 |
| XM_373749 | LOC388420 | -0.95 | NM_000162 | GCK | -0.94 |
| NM_004972 | JAK2 | -0.94 | NM_181504 | PIK3R1 | -0.94 |
| NM_003726 | SCAP1 | -0.94 | NM_000516 | GNAS | -0.94 |
| NM_023915 | GPR87 | -0.94 | NM_005959 | MTNR1B | -0.94 |

| | | |
|---|---|---|
| NM_003651 | CSDA | -0.94 |
| NM_019892 | INPP5E | -0.94 |
| NM_005484 | ADPRTL2 | -0.94 |
| NM_003852 | TIF1 | -0.94 |
| NM_000261 | MYOC | -0.94 |
| NM_019841 | TRPV5 | -0.94 |
| NM_000440 | PDE6A | -0.94 |
| NM_005511 | MLANA | -0.94 |
| NM_005971 | FXYD3 | -0.94 |
| NM_032727 | INA | -0.94 |
| NM_014822 | SEC24D | -0.94 |
| NM_005791 | MPHOSPH10 | -0.94 |
| NM_015929 | LIPT1 | -0.94 |
| NM_018039 | JMJD2D | -0.94 |
| NM_020193 | C11ORF30 | -0.94 |
| NM_033066 | MPP4 | -0.94 |
| NM_024798 | SNX22 | -0.94 |
| NM_144587 | C10ORF87 | -0.94 |
| NM_080678 | NCE2 | -0.94 |
| XM_378973 | LOC284801 | -0.94 |
| XM_210906 | LOC283166 | -0.94 |
| XM_375929 | LOC400856 | -0.94 |
| NM_004842 | AKAP7 | -0.93 |
| NM_004360 | CDH1 | -0.93 |
| NM_007182 | RASSF1 | -0.93 |
| NM_001040 | SHBG | -0.93 |
| NM_003077 | SMARCD2 | -0.93 |
| NM_000193 | SHH | -0.93 |
| NM_002434 | MPG | -0.93 |
| NM_006686 | ACTL7B | -0.93 |
| NM_000066 | C8B | -0.93 |
| NM_005266 | GJA5 | -0.93 |
| NM_017732 | PH-4 | -0.93 |
| NM_054023 | SCGB3A2 | -0.93 |
| NM_004202 | TMSB4Y | -0.93 |
| NM_021004 | DHRS4 | -0.93 |
| NM_014281 | SIAHBP1 | -0.93 |
| NM_015630 | EPC2 | -0.93 |
| NM_014106 | FLJ20582 | -0.93 |
| NM_024756 | MMRN2 | -0.93 |
| NM_032329 | ING5 | -0.93 |
| NM_032366 | MGC13114 | -0.93 |
| NM_138344 | C14ORF152 | -0.93 |
| NM_178824 | FLJ33620 | -0.93 |
| NM_176816 | KENAE | -0.93 |
| XM_211197 | LOC283780 | -0.93 |
| NM_004739 | MTA2 | -0.94 |
| NM_006202 | PDE4A | -0.94 |
| NM_016937 | POLA | -0.94 |
| NM_002709 | PPP1CB | -0.94 |
| NM_003396 | WNT9B | -0.94 |
| NM_001363 | DKC1 | -0.94 |
| NM_012191 | NAT6 | -0.94 |
| NM_002137 | HNRPA2B1 | -0.94 |
| NM_003456 | ZNF205 | -0.94 |
| NM_004807 | HS6ST1 | -0.94 |
| NM_005788 | HRMT1L3 | -0.94 |
| NM_006825 | CKAP4 | -0.94 |
| NM_016446 | C9ORF127 | -0.94 |
| XM_290854 | GPR108 | -0.94 |
| NM_020800 | KIAA1374 | -0.94 |
| NM_022365 | DNAJC1 | -0.94 |
| NM_025243 | SLC19A3 | -0.94 |
| NM_177998 | OTOP1 | -0.94 |
| NM_080617 | CBLNL1 | -0.94 |
| NM_198570 | UNQ739 | -0.94 |
| XM_373802 | LOC388530 | -0.94 |
| NM_001005282 | OR5M8 | -0.94 |
| NM_001956 | EDN2 | -0.93 |
| NM_018014 | BCL11A | -0.93 |
| NM_002778 | PSAP | -0.93 |
| NM_006168 | NKX6-1 | -0.93 |
| NM_000034 | ALDOA | -0.93 |
| NM_002047 | GARS | -0.93 |
| NM_170744 | UNC5B | -0.93 |
| NM_000593 | TAP1 | -0.93 |
| NM_001343 | DAB2 | -0.93 |
| NM_022344 | NJMU-R1 | -0.93 |
| NM_005697 | SCAMP2 | -0.93 |
| XM_084514 | HSPCAL3 | -0.93 |
| NM_006865 | LILRA3 | -0.93 |
| NM_007359 | CASC3 | -0.93 |
| NM_012387 | PADI4 | -0.93 |
| NM_019037 | EXOSC4 | -0.93 |
| NM_020782 | KLHDC5 | -0.93 |
| NM_032151 | DCOHM | -0.93 |
| NM_032351 | MRPL45 | -0.93 |
| NM_138413 | C10ORF65 | -0.93 |
| NM_033197 | C20ORF114 | -0.93 |
| NM_144980 | C6ORF118 | -0.93 |
| XM_210062 | RAC4 | -0.93 |
| XM_294219 | LOC346355 | -0.93 |

| | | | | | |
|---|---|---|---|---|---|
| XM_373053 | LOC391733 | -0.93 | XM_377957 | LOC402287 | -0.93 |
| XM_378590 | LOC400533 | -0.93 | XM_379699 | LOC401583 | -0.93 |
| XM_380127 | LOC402560 | -0.93 | NM_006307 | SRPX | -0.92 |
| NM_004169 | SHMT1 | -0.92 | NM_002463 | MX2 | -0.92 |
| NM_000437 | PAFAH2 | -0.92 | NM_174907 | PPP4R2 | -0.92 |
| NM_006440 | TXNRD2 | -0.92 | NM_005409 | CXCL11 | -0.92 |
| NM_018558 | GABRQ | -0.92 | NM_001650 | AQP4 | -0.92 |
| NM_004504 | HRB | -0.92 | NM_152295 | TARS | -0.92 |
| NM_005867 | DSCR4 | -0.92 | NM_015370 | HS747E2A | -0.92 |
| NM_017570 | OPLAH | -0.92 | NM_016422 | RNF141 | -0.92 |
| NM_016125 | LOC51136 | -0.92 | NM_017716 | MS4A12 | -0.92 |
| NM_020211 | RGMA | -0.92 | NM_138400 | C7ORF3 | -0.92 |
| NM_031438 | NUDT12 | -0.92 | NM_033103 | RHPN2 | -0.92 |
| NM_152360 | ZNF573 | -0.92 | NM_174896 | MGC24133 | -0.92 |
| NM_144668 | MGC33630 | -0.92 | NM_145178 | ATOH7 | -0.92 |
| XM_375077 | C14ORF25 | -0.92 | NM_194272 | RBPMS2 | -0.92 |
| XM_374017 | LOC389059 | -0.92 | XM_378353 | LOC400030 | -0.92 |
| NM_001005490 | OR6C74 | -0.92 | NM_182707 | PSG8 | -0.92 |
| NM_022081 | HPS4 | -0.91 | NM_005778 | RBM5 | -0.91 |
| NM_002653 | PITX1 | -0.91 | NM_014564 | LHX3 | -0.91 |
| NM_005587 | MEF2A | -0.91 | NM_000712 | BLVRA | -0.91 |
| NM_005078 | TLE3 | -0.91 | NM_002238 | KCNH1 | -0.91 |
| NM_020535 | KIR2DL5 | -0.91 | NM_031933 | WNT8A | -0.91 |
| NM_139017 | IL31RA | -0.91 | NM_001848 | COL6A1 | -0.91 |
| NM_002826 | QSCN6 | -0.91 | NM_017744 | ST7L | -0.91 |
| NM_001803 | CDW52 | -0.91 | NM_002215 | ITIH1 | -0.91 |
| NM_031284 | ADP-GK | -0.91 | NM_005827 | SLC35B1 | -0.91 |
| NM_014829 | DDX46 | -0.91 | NM_014991 | WDFY3 | -0.91 |
| NM_014157 | HSPC065 | -0.91 | NM_022727 | HTF9C | -0.91 |
| NM_015917 | LOC51064 | -0.91 | NM_018991 | DKFZP434A0131 | -0.91 |
| NM_017765 | PQLC2 | -0.91 | NM_018111 | FLJ10490 | -0.91 |
| NM_021800 | DNAJC12 | -0.91 | NM_021732 | AVPI1 | -0.91 |
| NM_020863 | ZNF406 | -0.91 | NM_022772 | EPS8L2 | -0.91 |
| NM_022116 | FIGNL1 | -0.91 | NM_024728 | C7ORF10 | -0.91 |
| NM_030939 | C6ORF62 | -0.91 | NM_031455 | CCDC3 | -0.91 |
| NM_207310 | DKFZP434E2321 | -0.91 | NM_144632 | FLJ30294 | -0.91 |
| NM_152393 | KBTBD5 | -0.91 | NM_144637 | ZDHHC19 | -0.91 |
| NM_182752 | LOC127262 | -0.91 | XM_095168 | EZI | -0.91 |
| NM_152678 | FLJ34969 | -0.91 | NM_182540 | LOC203522 | -0.91 |
| NM_178865 | TDE2L | -0.91 | NM_207410 | UNQ9356 | -0.91 |
| NM_206922 | TLP | -0.91 | XM_291767 | LOC340900 | -0.91 |
| XM_374026 | LOC389081 | -0.91 | XM_372781 | LOC391047 | -0.91 |
| XM_370585 | LOC387721 | -0.91 | XM_371418 | LOC388830 | -0.91 |
| NM_001001795 | MGC70857 | -0.91 | NM_002069 | GNAI1 | -0.9 |
| NM_003834 | RGS11 | -0.9 | NM_005304 | GPR41 | -0.9 |
| NM_015517 | MIZF | -0.9 | NM_003944 | SELENBP1 | -0.9 |

| | | | | | |
|---|---|---|---|---|---|
| NM_022037 | TIA1 | -0.9 | NM_002697 | POU2F1 | -0.9 |
| NM_000282 | PCCA | -0.9 | NM_057175 | TBDN100 | -0.9 |
| NM_000228 | LAMB3 | -0.9 | NM_004862 | LITAF | -0.9 |
| NM_017869 | BANP | -0.9 | NM_006443 | C6ORF108 | -0.9 |
| NM_003798 | CTNNAL1 | -0.9 | BC025985 | IGHG4 | -0.9 |
| NM_138443 | CCDC5 | -0.9 | NM_052843 | OBSCN | -0.9 |
| NM_000232 | SGCB | -0.9 | NM_012234 | RYBP | -0.9 |
| NM_015646 | RAP1B | -0.9 | NM_004068 | AP2M1 | -0.9 |
| NM_006526 | ZNF217 | -0.9 | NM_007007 | CPSF6 | -0.9 |
| NM_015193 | ARC | -0.9 | NM_015079 | KIAA1055 | -0.9 |
| NM_022047 | DEF6 | -0.9 | NM_019078 | UGT1A5 | -0.9 |
| NM_016327 | UPB1 | -0.9 | NM_017686 | GDAP2 | -0.9 |
| NM_018476 | BEX1 | -0.9 | NM_020194 | GL004 | -0.9 |
| NM_030811 | MRPS26 | -0.9 | NM_130782 | RGS18 | -0.9 |
| NM_024589 | FLJ22386 | -0.9 | NM_033060 | KRTAP4-10 | -0.9 |
| XM_372075 | C9ORF123 | -0.9 | NM_138804 | LOC130951 | -0.9 |
| NM_080610 | CST9L | -0.9 | NM_152366 | MGC33338 | -0.9 |
| XM_059954 | C9ORF57 | -0.9 | XM_378860 | LOC149478 | -0.9 |
| XM_376829 | LOC158381 | -0.9 | NM_198458 | ZNF497 | -0.9 |
| NM_152694 | ZCCHC5 | -0.9 | NM_153375 | PLAC2 | -0.9 |
| XM_209104 | LOC284293 | -0.9 | XM_371791 | LOC389352 | -0.9 |
| XM_379664 | LOC401549 | -0.9 | XM_373765 | LOC388439 | -0.9 |
| XM_378760 | LOC400662 | -0.9 | NM_001004351 | MGC57359 | -0.9 |
| XM_373954 | LOC388893 | -0.9 | NM_004419 | DUSP5 | -0.89 |
| NM_020423 | PACE-1 | -0.89 | NM_014381 | MLH3 | -0.89 |
| NM_006271 | S100A1 | -0.89 | NM_006261 | PROP1 | -0.89 |
| NM_002541 | OGDH | -0.89 | NM_004260 | RECQL4 | -0.89 |
| NM_003105 | SORL1 | -0.89 | NM_021081 | GHRH | -0.89 |
| NM_003599 | SUPT3H | -0.89 | NM_000423 | KRT2A | -0.89 |
| NM_001042 | SLC2A4 | -0.89 | NM_015362 | DERP6 | -0.89 |
| NM_005085 | NUP214 | -0.89 | NM_004802 | OTOF | -0.89 |
| NM_001606 | ABCA2 | -0.89 | NM_003313 | TSTA3 | -0.89 |
| NM_006574 | CSPG5 | -0.89 | NM_018957 | SH3BP1 | -0.89 |
| NM_015363 | ZIM2 | -0.89 | NM_030758 | OSBP2 | -0.89 |
| NM_014342 | MTCH2 | -0.89 | NM_018134 | FLJ10547 | -0.89 |
| NM_018182 | FLJ10700 | -0.89 | NM_019602 | BTNL2 | -0.89 |
| NM_021933 | FLJ12438 | -0.89 | NM_031950 | KSP37 | -0.89 |
| XM_088525 | C9ORF28 | -0.89 | NM_138416 | LOC112937 | -0.89 |
| NM_052945 | TNFRSF13C | -0.89 | NM_138439 | LOC114984 | -0.89 |
| XM_379204 | LOC152225 | -0.89 | NM_144994 | ANKRD23 | -0.89 |
| NM_152786 | C9ORF43 | -0.89 | XM_372924 | LOC391362 | -0.89 |
| XM_379182 | LOC401058 | -0.89 | XM_373865 | LOC388690 | -0.89 |
| XM_371837 | LOC389416 | -0.89 | XM_377129 | LOC401647 | -0.89 |
| XM_373555 | LOC387897 | -0.89 | XM_370894 | LOC388160 | -0.89 |
| NM_022445 | TPK1 | -0.88 | NM_000316 | PTHR1 | -0.88 |
| NM_005483 | CHAF1A | -0.88 | NM_006813 | PNRC1 | -0.88 |

| | | | | | |
|---|---|---|---|---|---|
| NM_170607 | TCFL4 | -0.88 | NM_003399 | XPNPEP2 | -0.88 |
| NM_000062 | SERPING1 | -0.88 | NM_174941 | M160 | -0.88 |
| NM_006116 | MAP3K7IP1 | -0.88 | NM_000494 | COL17A1 | -0.88 |
| NM_021952 | ELAVL4 | -0.88 | NM_019054 | FAM35A | -0.88 |
| NM_006198 | PCP4 | -0.88 | NM_002403 | MFAP2 | -0.88 |
| NM_021029 | RPL36A | -0.88 | NM_006782 | ZFPL1 | -0.88 |
| NM_003569 | STX7 | -0.88 | NM_018691 | C5ORF3 | -0.88 |
| NM_007275 | TUSC2 | -0.88 | NM_014016 | SACM1L | -0.88 |
| NM_015609 | DKFZP566C0424 | -0.88 | NM_014407 | KCNMB3 | -0.88 |
| NM_032295 | SLC37A3 | -0.88 | XM_042234 | LOC92017 | -0.88 |
| XM_038576 | LOC91461 | -0.88 | NM_138440 | LOC114990 | -0.88 |
| XM_085375 | ZFP90 | -0.88 | XM_085383 | LOC146206 | -0.88 |
| NM_144996 | DKFZP761H079 | -0.88 | NM_173561 | UNC5CL | -0.88 |
| NM_178525 | MGC33407 | -0.88 | NM_003241 | TGM4 | -0.88 |
| XM_058857 | LOC124871 | -0.88 | XM_210876 | LOC283065 | -0.88 |
| XM_376160 | LOC401026 | -0.88 | XM_370924 | LOC388199 | -0.88 |
| NM_018084 | KIAA1212 | -0.88 | NM_032844 | MASTL | -0.87 |
| NM_148963 | GPRC6A | -0.87 | NM_018179 | ATF7IP | -0.87 |
| NM_001186 | BACH1 | -0.87 | NM_003489 | NRIP1 | -0.87 |
| NM_005920 | MEF2D | -0.87 | NM_019857 | CTPS2 | -0.87 |
| NM_000413 | HSD17B1 | -0.87 | NM_016021 | UBE2J1 | -0.87 |
| NM_003628 | PKP4 | -0.87 | NM_031903 | MRPL32 | -0.87 |
| NM_004475 | FLOT2 | -0.87 | NM_006178 | NSF | -0.87 |
| NM_004876 | ZNF254 | -0.87 | NM_006710 | COPS8 | -0.87 |
| NM_015324 | KIAA0409 | -0.87 | NM_018226 | RNPEPL1 | -0.87 |
| NM_017809 | NXF2 | -0.87 | NM_020657 | ZNF304 | -0.87 |
| NM_024614 | FLJ13197 | -0.87 | NM_024824 | FLJ11806 | -0.87 |
| NM_031957 | KRTAP1-5 | -0.87 | NM_182481 | BAGE3 | -0.87 |
| NM_181710 | ZNRF4 | -0.87 | NM_144724 | MRVLDC2 | -0.87 |
| NM_153233 | FLJ36445 | -0.87 | NM_175922 | MGC35308 | -0.87 |
| XM_293923 | LOC345645 | -0.87 | XM_293875 | LOC345557 | -0.87 |
| XM_291977 | LOC338751 | -0.87 | XM_379595 | LOC401471 | -0.87 |
| XM_371028 | LOC388353 | -0.87 | XM_370930 | LOC388205 | -0.87 |
| NM_006422 | AKAP3 | -0.86 | NM_002740 | PRKCI | -0.86 |
| NM_007284 | PTK9L | -0.86 | NM_030784 | GPR63 | -0.86 |
| NM_021619 | PRDM12 | -0.86 | NM_014468 | VENTX2 | -0.86 |
| NM_014244 | ADAMTS2 | -0.86 | NM_000169 | GLA | -0.86 |
| NM_000240 | MAOA | -0.86 | NM_003234 | TFRC | -0.86 |
| NM_003336 | UBE2A | -0.86 | NM_006240 | PPEF1 | -0.86 |
| NM_004829 | NCR1 | -0.86 | NM_005310 | GRB7 | -0.86 |
| NM_014432 | IL20RA | -0.86 | NM_017442 | TLR9 | -0.86 |
| NM_003846 | PEX11B | -0.86 | NM_001044 | SLC6A3 | -0.86 |
| NM_005678 | SNURF | -0.86 | NM_001867 | COX7C | -0.86 |
| NM_004990 | MARS | -0.86 | NM_016108 | AIG1 | -0.86 |
| NM_006958 | ZNF16 | -0.86 | NM_006348 | COG5 | -0.86 |
| NM_006629 | ZNF271 | -0.86 | NM_015659 | DKFZP564M182 | -0.86 |

| | | | | | |
|---|---|---|---|---|---|
| NM_014359 | OPTC | -0.86 | NM_015936 | CGI-04 | -0.86 |
| NM_020437 | LOC57168 | -0.86 | NM_022473 | ZFP106 | -0.86 |
| NM_033086 | FGD3 | -0.86 | NM_152543 | FLJ25371 | -0.86 |
| NM_145026 | SPATS1 | -0.86 | NM_173656 | ZNF619 | -0.86 |
| XM_375316 | LOC283887 | -0.86 | XM_380174 | LOC285192 | -0.86 |
| NM_177403 | MGC9726 | -0.86 | NM_198992 | SYT10 | -0.86 |
| NM_207384 | UNQ5810 | -0.86 | NM_207389 | FLJ44861 | -0.86 |
| XM_017966 | LOC152905 | -0.86 | XM_371847 | LOC389429 | -0.86 |
| XM_379508 | LOC401386 | -0.86 | XM_372090 | LOC389725 | -0.86 |
| XM_377231 | LOC401704 | -0.86 | XM_375373 | LOC400547 | -0.86 |
| XM_375282 | LOC400479 | -0.86 | NM_004560 | ROR2 | -0.85 |
| NM_053006 | STK22B | -0.85 | NM_024980 | GPR157 | -0.85 |
| NM_014268 | MAPRE2 | -0.85 | NM_002359 | MAFG | -0.85 |
| NM_004457 | ACSL3 | -0.85 | NM_030949 | PPP1R14C | -0.85 |
| NM_012464 | TLL1 | -0.85 | NM_000805 | GAS | -0.85 |
| NM_003573 | LTBP4 | -0.85 | NM_001854 | COL11A1 | -0.85 |
| NM_018844 | BCAP29 | -0.85 | NM_001793 | CDH3 | -0.85 |
| NM_002462 | MX1 | -0.85 | NM_012227 | PGPL | -0.85 |
| NM_005803 | FLOT1 | -0.85 | NM_006460 | HIS1 | -0.85 |
| XM_375042 | DKFZP434B061 | -0.85 | NM_016045 | C20ORF45 | -0.85 |
| NM_016070 | MRPS23 | -0.85 | NM_016126 | LOC51668 | -0.85 |
| NM_017656 | ZNF562 | -0.85 | NM_032292 | FLJ20203 | -0.85 |
| NM_017633 | C6ORF37 | -0.85 | NM_020199 | C5ORF15 | -0.85 |
| NM_022484 | FLJ13576 | -0.85 | NM_032127 | DKFZP566M1046 | -0.85 |
| NM_144604 | LOC124245 | -0.85 | NM_152408 | FLJ35779 | -0.85 |
| NM_207327 | LOC150383 | -0.85 | NM_152504 | FLJ25067 | -0.85 |
| NM_153702 | MGC10084 | -0.85 | NM_174961 | SSX8 | -0.85 |
| NM_181610 | KRTAP19-4 | -0.85 | NM_182620 | FAM33A | -0.85 |
| XM_086402 | LOC149018 | -0.85 | XM_293529 | LOC344657 | -0.85 |
| XM_372138 | LOC389786 | -0.85 | XM_374765 | LOC399712 | -0.85 |
| NM_001002925 | OR5AP2 | -0.85 | NM_004570 | PIK3C2G | -0.84 |
| NM_002860 | PYCS | -0.84 | NM_005855 | RAMP1 | -0.84 |
| NM_152526 | ALS2CR19 | -0.84 | NM_003528 | HIST2H2BE | -0.84 |
| NM_020418 | PCBP4 | -0.84 | NM_002160 | TNC | -0.84 |
| NM_001478 | GALGT | -0.84 | NM_198834 | ACACA | -0.84 |
| NM_006357 | UBE2E3 | -0.84 | NM_080677 | DLC2 | -0.84 |
| NM_014784 | ARHGEF11 | -0.84 | NM_002353 | TACSTD2 | -0.84 |
| NM_002618 | PEX13 | -0.84 | NM_005718 | ARPC4 | -0.84 |
| NM_006807 | CBX1 | -0.84 | NM_000883 | IMPDH1 | -0.84 |
| NM_019842 | KCNQ5 | -0.84 | NM_006463 | STAMBP | -0.84 |
| NM_007362 | NCBP2 | -0.84 | NM_012118 | CCRN4L | -0.84 |
| NM_020847 | TNRC6 | -0.84 | NM_016607 | ARMCX3 | -0.84 |
| NM_017637 | BNC2 | -0.84 | NM_017539 | DNAH3 | -0.84 |
| NM_020243 | TOMM22 | -0.84 | NM_032479 | MRPL36 | -0.84 |
| NM_023943 | MGC3040 | -0.84 | NM_024764 | C14ORF161 | -0.84 |
| NM_025267 | MGC2744 | -0.84 | NM_031904 | FKSG44 | -0.84 |

| | | | | | |
|---|---|---|---|---|---|
| NM_030819 | MGC11335 | -0.84 | NM_033342 | TRIM7 | -0.84 |
| NM_080655 | MGC17337 | -0.84 | NM_152279 | ZNF585B | -0.84 |
| NM_170698 | DJ222E13.2 | -0.84 | NM_152470 | C18ORF23 | -0.84 |
| NM_153611 | MGC20446 | -0.84 | XM_371662 | LOC255330 | -0.84 |
| NM_175903 | LOC284033 | -0.84 | XM_211509 | LOC284527 | -0.84 |
| XM_115897 | LOC203510 | -0.84 | XM_372940 | LOC391403 | -0.84 |
| XM_373001 | LOC391566 | -0.84 | XM_379402 | LOC401242 | -0.84 |
| XM_374432 | LOC392666 | -0.84 | XM_379608 | LOC401480 | -0.84 |
| XM_378993 | LOC400852 | -0.84 | NM_016291 | IHPK2 | -0.83 |
| NM_031220 | PITPNM3 | -0.83 | NM_005793 | NME6 | -0.83 |
| NM_004736 | XPR1 | -0.83 | NM_016734 | PAX5 | -0.83 |
| NM_006473 | TAF6L | -0.83 | NM_032797 | AMID | -0.83 |
| NM_002569 | FURIN | -0.83 | NM_003312 | TST | -0.83 |
| NM_002717 | PPP2R2A | -0.83 | NM_002891 | RASGRF1 | -0.83 |
| NM_013409 | FST | -0.83 | NM_002175 | IFNA21 | -0.83 |
| NM_000040 | APOC3 | -0.83 | NM_014456 | PDCD4 | -0.83 |
| NM_016333 | SRRM2 | -0.83 | NM_001629 | ALOX5AP | -0.83 |
| NM_001431 | EPB41L2 | -0.83 | NM_021013 | KRTHA4 | -0.83 |
| NM_006228 | PNOC | -0.83 | NM_015196 | KIAA0922 | -0.83 |
| NM_015633 | FGFR1OP2 | -0.83 | NM_020528 | PCBP3 | -0.83 |
| XM_044921 | KIAA1442 | -0.83 | NM_172231 | SF4 | -0.83 |
| NM_021946 | FLJ11362 | -0.83 | NM_022104 | C20ORF67 | -0.83 |
| NM_024638 | QTRTD1 | -0.83 | NM_025212 | CXXC4 | -0.83 |
| NM_145753 | PHLDB2 | -0.83 | NM_052832 | SLC26A7 | -0.83 |
| NM_130808 | CPNE4 | -0.83 | NM_138479 | LOC148898 | -0.83 |
| NM_152548 | FLJ25333 | -0.83 | NM_174951 | FAM9A | -0.83 |
| XM_088735 | H2BFWT | -0.83 | NM_138967 | SCAMP5 | -0.83 |
| NM_145027 | C6ORF102 | -0.83 | XM_170658 | DKFZP762C186 | -0.83 |
| NM_198480 | ZNF615 | -0.83 | NM_177967 | PHGDHL1 | -0.83 |
| XM_375700 | LOC400746 | -0.83 | XM_371817 | LOC389385 | -0.83 |
| XM_373585 | LOC387977 | -0.83 | NM_001005236 | OR1L1 | -0.83 |
| NM_002748 | MAPK6 | -0.82 | NM_002685 | EXOSC10 | -0.82 |
| NM_005424 | TIE | -0.82 | NM_002922 | RGS1 | -0.82 |
| NM_005527 | HSPA1L | -0.82 | NM_006106 | YAP1 | -0.82 |
| NM_006963 | ZNF22 | -0.82 | NM_003791 | MBTPS1 | -0.82 |
| NM_005040 | PRCP | -0.82 | NM_001875 | CPS1 | -0.82 |
| NM_005536 | IMPA1 | -0.82 | NM_020777 | SORCS2 | -0.82 |
| NM_000594 | TNF | -0.82 | NM_017767 | SLC39A4 | -0.82 |
| NM_139054 | ADAMTS18 | -0.82 | NM_014632 | MICAL2 | -0.82 |
| NM_000454 | SOD1 | -0.82 | NM_005328 | HAS2 | -0.82 |
| NM_000793 | DIO2 | -0.82 | NM_007353 | GNA12 | -0.82 |
| NM_002079 | GOT1 | -0.82 | NM_024409 | NPPC | -0.82 |
| NM_006993 | NPM3 | -0.82 | NM_015947 | ASCC1 | -0.82 |
| NM_017580 | ZRANB1 | -0.82 | NM_017919 | STX17 | -0.82 |
| NM_018027 | FRMD4 | -0.82 | NM_030963 | RNF146 | -0.82 |
| NM_032256 | DKFZP434K2435 | -0.82 | NM_032564 | DGAT2 | -0.82 |

| | | | | | |
|---|---|---|---|---|---|
| NM_033085 | FATE | -0.82 | NM_175736 | FMNL3 | -0.82 |
| NM_199133 | LOC134145 | -0.82 | NM_018083 | ZNF358 | -0.82 |
| NM_152496 | FLJ31434 | -0.82 | NM_207582 | HERV-FRD | -0.82 |
| NM_152647 | FLJ32800 | -0.82 | NM_153836 | CREG2 | -0.82 |
| NM_194310 | LOC284837 | -0.82 | NM_199243 | GPR150 | -0.82 |
| NM_178428 | LCE2A | -0.82 | XM_208835 | LOC283767 | -0.82 |
| XM_373349 | LOC392467 | -0.82 | NM_001004330 | FLJ46688 | -0.82 |
| NM_001001696 | FLJ44006 | -0.82 | NM_003467 | CXCR4 | -0.81 |
| NM_198074 | OR2C3 | -0.81 | NM_001554 | CYR61 | -0.81 |
| NM_004658 | RASAL1 | -0.81 | NM_000769 | CYP2C19 | -0.81 |
| NM_005977 | RNF6 | -0.81 | NM_001201 | BMP3 | -0.81 |
| NM_000757 | CSF1 | -0.81 | NM_002687 | PNN | -0.81 |
| NM_004415 | DSP | -0.81 | NM_004770 | KCNB2 | -0.81 |
| NM_000898 | MAOB | -0.81 | NM_006788 | RALBP1 | -0.81 |
| NM_021614 | KCNN2 | -0.81 | NM_021128 | POLR2L | -0.81 |
| NM_003649 | DDO | -0.81 | NM_006458 | TRIM3 | -0.81 |
| XM_045308 | PHF19 | -0.81 | NM_019072 | SGTB | -0.81 |
| NM_018348 | FLJ11171 | -0.81 | NM_020654 | SENP7 | -0.81 |
| NM_020354 | ENTPD7 | -0.81 | NM_021823 | MDS018 | -0.81 |
| NM_022361 | POPDC3 | -0.81 | NM_022895 | FLJ12448 | -0.81 |
| NM_022340 | ZFYVE20 | -0.81 | NM_025004 | FLJ13215 | -0.81 |
| XM_290811 | KIAA1713 | -0.81 | NM_030674 | SLC38A1 | -0.81 |
| NM_032488 | LOC84518 | -0.81 | NM_032499 | HH114 | -0.81 |
| NM_052910 | SLITRK1 | -0.81 | NM_181706 | LOC120526 | -0.81 |
| XM_378175 | LOC124446 | -0.81 | NM_145650 | MUC15 | -0.81 |
| NM_152551 | C6ORF151 | -0.81 | NM_152550 | SH3RF2 | -0.81 |
| NM_152697 | MGC34032 | -0.81 | NM_153612 | HS3ST5 | -0.81 |
| XM_379086 | LOC285045 | -0.81 | XM_290831 | LOC339321 | -0.81 |
| XM_497067 | OR7E31P | -0.81 | XM_373885 | LOC388735 | -0.81 |
| XM_173132 | LOC255620 | -0.81 | XM_088367 | LOC157708 | -0.81 |
| XM_379072 | LOC400939 | -0.81 | XM_374254 | LOC389636 | -0.81 |
| XM_378742 | LOC400645 | -0.81 | NM_001005922 | KRTAP5-1 | -0.81 |
| NM_002565 | P2RY4 | -0.8 | NM_002551 | OR3A2 | -0.8 |
| NM_003777 | DNAH11 | -0.8 | NM_030665 | RAI1 | -0.8 |
| NM_002473 | MYH9 | -0.8 | NM_052884 | SIGLEC11 | -0.8 |
| NM_001385 | DPYS | -0.8 | NM_006044 | HDAC6 | -0.8 |
| NM_014336 | AIPL1 | -0.8 | NM_000565 | IL6R | -0.8 |
| NM_003728 | UNC5C | -0.8 | NM_013246 | CLC | -0.8 |
| NM_001302 | CORT | -0.8 | NM_003673 | TCAP | -0.8 |
| NM_006020 | ALKBH | -0.8 | NM_002048 | GAS1 | -0.8 |
| NM_001817 | CEACAM4 | -0.8 | NM_002799 | PSMB7 | -0.8 |
| NM_003271 | TM4SF7 | -0.8 | NM_006703 | NUDT3 | -0.8 |
| NM_015440 | FTHFSDC1 | -0.8 | NM_014017 | MAPBPIP | -0.8 |
| NM_015662 | SLB | -0.8 | NM_016429 | COPZ2 | -0.8 |
| NM_013334 | GMPPB | -0.8 | NM_020358 | RNF18 | -0.8 |
| NM_020390 | EIF5A2 | -0.8 | XM_114432 | ZNF608 | -0.8 |

| | | | | | |
|---|---|---|---|---|---|
| NM_024712 | ELMO3 | -0.8 | NM_024577 | KIAA1985 | -0.8 |
| NM_025137 | FLJ21439 | -0.8 | NM_025264 | THUMPD2 | -0.8 |
| NM_025163 | SMP3 | -0.8 | NM_032141 | DKFZP434K1421 | -0.8 |
| NM_030944 | C15ORF5 | -0.8 | NM_032118 | FLJ12953 | -0.8 |
| NM_032899 | BJ-TSA-9 | -0.8 | NM_138408 | C6ORF51 | -0.8 |
| NM_033036 | GAL3ST3 | -0.8 | NM_138419 | DUFD1 | -0.8 |
| NM_172193 | KLHDC1 | -0.8 | XM_088331 | LOC157570 | -0.8 |
| NM_205548 | UNQ9217 | -0.8 | NM_198276 | TMEM17 | -0.8 |
| NM_182598 | FLJ36980 | -0.8 | XM_370948 | LOC388228 | -0.8 |
| NM_207406 | FLJ43965 | -0.8 | NM_019110 | ZNF307 | -0.8 |
| XM_372258 | LOC389899 | -0.8 | XM_371431 | LOC388854 | -0.8 |
| NM_005192 | CDKN3 | -0.79 | NM_005734 | HIPK3 | -0.79 |
| NM_006904 | PRKDC | -0.79 | NM_032781 | PTPN5 | -0.79 |
| NM_006852 | TLK2 | -0.79 | NM_005507 | CFL1 | -0.79 |
| NM_005780 | LHFP | -0.79 | XM_371380 | S100A13 | -0.79 |
| NM_021212 | ZF | -0.79 | NM_005551 | KLK2 | -0.79 |
| NM_014936 | ENPP4 | -0.79 | NM_005943 | MOCS1 | -0.79 |
| NM_000512 | GALNS | -0.79 | NM_001141 | ALOX15B | -0.79 |
| NM_002116 | HLA-A | -0.79 | NM_000206 | IL2RG | -0.79 |
| NM_016113 | TRPV2 | -0.79 | NM_002203 | ITGA2 | -0.79 |
| NM_002356 | MARCKS | -0.79 | NM_013385 | PSCD4 | -0.79 |
| NM_001543 | NDST1 | -0.79 | NM_000846 | GSTA2 | -0.79 |
| NM_021016 | PSG3 | -0.79 | NM_006743 | RBM3 | -0.79 |
| NM_003490 | SYN3 | -0.79 | NM_006771 | KRTHA8 | -0.79 |
| NM_006118 | HAX1 | -0.79 | NM_020997 | LEFTB | -0.79 |
| NM_014467 | SRPUL | -0.79 | NM_013293 | TRA2A | -0.79 |
| NM_017434 | DUOX1 | -0.79 | NM_019082 | DDX56 | -0.79 |
| NM_020395 | LOC57117 | -0.79 | NM_198945 | ALS2CR17 | -0.79 |
| NM_024045 | DDX50 | -0.79 | XM_370878 | KIAA2002 | -0.79 |
| NM_025103 | CCDC2 | -0.79 | NM_032188 | MYST1 | -0.79 |
| NM_138461 | LOC116211 | -0.79 | NM_177478 | MTF | -0.79 |
| NM_144645 | MGC26744 | -0.79 | XM_375033 | LOC144776 | -0.79 |
| NM_173507 | FLJ37118 | -0.79 | XM_016548 | CDY | -0.79 |
| XM_175125 | DKFZP434P0216 | -0.79 | NM_182567 | FLJ34690 | -0.79 |
| NM_201628 | FLJ43806 | -0.79 | XM_293401 | LOC347527 | -0.79 |
| XM_373854 | LOC388652 | -0.79 | XM_374173 | LOC389409 | -0.79 |
| XM_379483 | LOC401321 | -0.79 | XM_377835 | LOC402168 | -0.79 |
| XM_374266 | LOC389650 | -0.79 | XM_372319 | LOC389983 | -0.79 |
| XM_370582 | LOC387718 | -0.79 | NM_007174 | CIT | -0.78 |
| NM_021120 | DLG3 | -0.78 | NM_007264 | ADMR | -0.78 |
| NM_003390 | WEE1 | -0.78 | NM_006374 | STK25 | -0.78 |
| NM_012352 | OR1A2 | -0.78 | NM_032951 | WBSCR14 | -0.78 |
| NM_001179 | ART3 | -0.78 | NM_000946 | PRIM1 | -0.78 |
| NM_000203 | IDUA | -0.78 | NM_006034 | TP53I11 | -0.78 |
| NM_032738 | FREB | -0.78 | NM_004979 | KCND1 | -0.78 |
| NM_001251 | CD68 | -0.78 | NM_012394 | PFDN2 | -0.78 |

| | | | | | |
|---|---|---|---|---|---|
| NM_004507 | HUS1 | -0.78 | NM_003104 | SORD | -0.78 |
| NM_002396 | ME2 | -0.78 | NM_001018 | RPS15 | -0.78 |
| NM_004245 | TGM5 | -0.78 | NM_006820 | C1ORF29 | -0.78 |
| NM_007280 | OIP5 | -0.78 | NM_012392 | PEF | -0.78 |
| NM_152692 | C1GALT2 | -0.78 | NM_016623 | BM-009 | -0.78 |
| NM_019018 | FLJ11127 | -0.78 | NM_016488 | PPHLN1 | -0.78 |
| NM_017559 | DKFZP434H2215 | -0.78 | NM_019605 | SERTAD4 | -0.78 |
| NM_020748 | KIAA1287 | -0.78 | NM_024643 | C14ORF140 | -0.78 |
| NM_024841 | FLJ14213 | -0.78 | NM_032262 | DKFZP434N035 | -0.78 |
| NM_133367 | C6ORF33 | -0.78 | XM_371605 | LOC151174 | -0.78 |
| NM_198275 | LOC196264 | -0.78 | NM_145030 | MGC22793 | -0.78 |
| NM_174925 | LOC205251 | -0.78 | NM_032436 | C13ORF8 | -0.78 |
| XM_209073 | LOC284207 | -0.78 | NM_182625 | FLJ40869 | -0.78 |
| XM_295865 | LOC340895 | -0.78 | XM_294960 | LOC339453 | -0.78 |
| XM_293828 | LOC345462 | -0.78 | XM_374086 | LOC389224 | -0.78 |
| XM_377836 | LOC402169 | -0.78 | XM_373908 | LOC388789 | -0.78 |
| NM_002731 | PRKACB | -0.77 | NM_000679 | ADRA1B | -0.77 |
| NM_003745 | SOCS1 | -0.77 | NM_005300 | GPR34 | -0.77 |
| NM_019599 | TAS2R1 | -0.77 | NM_005186 | CAPN1 | -0.77 |
| NM_003132 | SRM | -0.77 | NM_004637 | RAB7 | -0.77 |
| NM_013282 | UHRF1 | -0.77 | NM_017514 | PLXNA3 | -0.77 |
| NM_002845 | PTPRM | -0.77 | NM_006628 | ARPP-19 | -0.77 |
| NM_020655 | JPH3 | -0.77 | NM_005236 | ERCC4 | -0.77 |
| NM_014922 | NALP1 | -0.77 | NM_001688 | ATP5F1 | -0.77 |
| NM_005663 | WHSC2 | -0.77 | NM_004939 | DDX1 | -0.77 |
| NM_006901 | MYO9A | -0.77 | NM_003446 | ZNF157 | -0.77 |
| XM_045581 | AZI1 | -0.77 | NM_012265 | C22ORF3 | -0.77 |
| XM_376310 | ZCCHC4 | -0.77 | NM_016101 | CGI-37 | -0.77 |
| NM_016023 | CGI-77 | -0.77 | NM_016258 | YTHDF2 | -0.77 |
| NM_017838 | NOLA2 | -0.77 | NM_018335 | C14ORF131 | -0.77 |
| NM_020379 | MAN1C1 | -0.77 | NM_024715 | C5ORF14 | -0.77 |
| NM_024725 | FLJ23518 | -0.77 | NM_024650 | FLJ22531 | -0.77 |
| NM_030568 | C6ORF148 | -0.77 | NM_031962 | KRTAP9-3 | -0.77 |
| NM_053280 | ODF3 | -0.77 | NM_207319 | FLJ32867 | -0.77 |
| NM_144718 | LOC152185 | -0.77 | NM_181713 | UBXD4 | -0.77 |
| NM_145294 | LOC197336 | -0.77 | NM_145011 | ZNF25 | -0.77 |
| NM_153256 | C10ORF47 | -0.77 | NM_172370 | DAOA | -0.77 |
| NM_198924 | TRIM50B | -0.77 | NM_005866 | OPRS1 | -0.77 |
| XM_062269 | LOC120793 | -0.77 | XM_372840 | LOC391209 | -0.77 |
| XM_370845 | LOC388097 | -0.77 | NM_001002758 | PRY2 | -0.77 |
| NM_004942 | DEFB4 | -0.76 | NM_002548 | OR1D2 | -0.76 |
| NM_014430 | CIDEB | -0.76 | NM_176821 | NALP10 | -0.76 |
| NM_002603 | PDE7A | -0.76 | NM_004111 | FEN1 | -0.76 |
| NM_005213 | CSTA | -0.76 | NM_020438 | DOLPP1 | -0.76 |
| NM_006274 | CCL19 | -0.76 | NM_020389 | TRPC7 | -0.76 |
| NM_003639 | IKBKG | -0.76 | NM_018834 | MATR3 | -0.76 |

| | | | | | |
|---|---|---|---|---|---|
| NM_001985 | ETFB | -0.76 | NM_014252 | SLC25A15 | -0.76 |
| NM_004750 | CRLF1 | -0.76 | NM_001317 | CSH1 | -0.76 |
| NM_004701 | CCNB2 | -0.76 | NM_080612 | GAB3 | -0.76 |
| NM_024735 | FBXO31 | -0.76 | NM_001001655 | MGC90512 | -0.76 |
| NM_001267 | CHAD | -0.76 | NM_002216 | ITIH2 | -0.76 |
| NM_006161 | NEUROG1 | -0.76 | NM_004278 | PIGL | -0.76 |
| NM_007063 | TBC1D8 | -0.76 | NM_006867 | RBPMS | -0.76 |
| NM_020368 | SAS10 | -0.76 | NM_024920 | FLJ14281 | -0.76 |
| NM_032316 | NICN1 | -0.76 | XM_040592 | ZNF469 | -0.76 |
| NM_032597 | NYD-SP21 | -0.76 | NM_033100 | PCDH21 | -0.76 |
| NM_152665 | FLJ40873 | -0.76 | NM_182833 | LOC220032 | -0.76 |
| NM_145006 | SUSD3 | -0.76 | NM_152722 | FLJ25530 | -0.76 |
| NM_173662 | LOC285533 | -0.76 | NM_173621 | FLJ34790 | -0.76 |
| NM_181722 | LOC285908 | -0.76 | NM_182489 | LOC346673 | -0.76 |
| NM_178019 | CATSPER3 | -0.76 | XM_379642 | LOC401528 | -0.76 |
| XM_379635 | LOC401506 | -0.76 | NM_172207 | CAMKK1 | -0.75 |
| NM_019884 | GSK3A | -0.75 | NM_002072 | GNAQ | -0.75 |
| NM_181885 | GPR100 | -0.75 | NM_176883 | TAS2R41 | -0.75 |
| NM_005435 | ARHGEF5 | -0.75 | NM_002656 | PLAGL1 | -0.75 |
| NM_001352 | DBP | -0.75 | NM_024865 | NANOG | -0.75 |
| NM_005685 | GTF2IRD1 | -0.75 | NM_003927 | MBD2 | -0.75 |
| NM_003367 | USF2 | -0.75 | NM_005485 | ADPRTL3 | -0.75 |
| NM_000859 | HMGCR | -0.75 | NM_139318 | KCNH5 | -0.75 |
| NM_004228 | PSCD2 | -0.75 | NM_006846 | SPINK5 | -0.75 |
| NM_005581 | LU | -0.75 | NM_000417 | IL2RA | -0.75 |
| NM_002438 | MRC1 | -0.75 | NM_006019 | TCIRG1 | -0.75 |
| NM_000352 | ABCC8 | -0.75 | NM_015434 | DKFZP434B168 | -0.75 |
| NM_006317 | BASP1 | -0.75 | NM_145237 | LOC94431 | -0.75 |
| NM_001313 | CRMP1 | -0.75 | NM_024014 | HOXA6 | -0.75 |
| NM_006513 | SARS | -0.75 | NM_003740 | KCNK5 | -0.75 |
| NM_014661 | KIAA0140 | -0.75 | XM_375729 | C1ORF34 | -0.75 |
| NM_015380 | CGI-51 | -0.75 | NM_014145 | C20ORF30 | -0.75 |
| NM_016369 | CLDN18 | -0.75 | NM_021260 | ZFYVE1 | -0.75 |
| NM_017429 | BCMO1 | -0.75 | NM_017631 | FLJ20035 | -0.75 |
| NM_018327 | C20ORF38 | -0.75 | NM_194071 | CREB3L2 | -0.75 |
| NM_023074 | FLJ12644 | -0.75 | NM_025241 | UBXD1 | -0.75 |
| NM_025257 | C6ORF29 | -0.75 | NM_024832 | RIN3 | -0.75 |
| NM_025108 | FLJ13909 | -0.75 | NM_031482 | APG10L | -0.75 |
| NM_032599 | NYD-SP18 | -0.75 | NM_052873 | MGC16028 | -0.75 |
| XM_373731 | KRTAP4-9 | -0.75 | NM_030631 | SLC25A21 | -0.75 |
| NM_152319 | MGC35033 | -0.75 | NM_182485 | CPEB2 | -0.75 |
| NM_173519 | MGC34646 | -0.75 | NM_178508 | MGC57858 | -0.75 |
| NM_173588 | FLJ37794 | -0.75 | XM_171032 | LOC255812 | -0.75 |
| NM_207400 | FLJ39739 | -0.75 | XM_371139 | FLJ14959 | -0.75 |
| NM_178566 | ZDHHC21 | -0.75 | NM_207428 | FLJ45212 | -0.75 |
| XM_059267 | LOC128710 | -0.75 | XM_210826 | LOC286404 | -0.75 |

| | | | | | |
|---|---|---|---|---|---|
| XM_167275 | LOC222901 | -0.75 | XM_294634 | LOC340268 | -0.75 |
| XM_376795 | LOC401478 | -0.75 | XM_370707 | LOC387890 | -0.75 |
| XM_378434 | LOC400161 | -0.75 | XM_373368 | LOC392533 | -0.75 |
| NM_001002906 | XKRY2 | -0.75 | NM_001005566 | OR5B2 | -0.75 |
| NM_003948 | CDKL2 | -0.74 | NM_004154 | P2RY6 | -0.74 |
| NM_006219 | PIK3CB | -0.74 | XM_378929 | KIAA0663 | -0.74 |
| NM_005709 | USH1C | -0.74 | NM_006079 | CITED2 | -0.74 |
| NM_017420 | SIX4 | -0.74 | NM_002877 | RAD51L1 | -0.74 |
| NM_006502 | POLH | -0.74 | NM_003855 | IL18R1 | -0.74 |
| NM_002560 | P2RX4 | -0.74 | XM_377014 | ARHGEF9 | -0.74 |
| NM_013230 | CD24 | -0.74 | NM_001207 | BTF3 | -0.74 |
| NM_002230 | JUP | -0.74 | NM_012282 | KCNE1L | -0.74 |
| NM_015894 | STMN3 | -0.74 | NM_002633 | PGM1 | -0.74 |
| XM_035572 | C4ORF9 | -0.74 | NM_005445 | CSPG6 | -0.74 |
| XM_376328 | FAM13A1 | -0.74 | NM_006859 | LIAS | -0.74 |
| NM_014480 | ZNF544 | -0.74 | XM_039676 | KIAA1240 | -0.74 |
| NM_018139 | C14ORF104 | -0.74 | NM_018418 | SPATA7 | -0.74 |
| NM_019107 | C19ORF10 | -0.74 | NM_020708 | SLC12A5 | -0.74 |
| NM_022156 | PP3111 | -0.74 | NM_024740 | DIBD1 | -0.74 |
| NM_024664 | FLJ11838 | -0.74 | XM_371740 | ELOVL7 | -0.74 |
| NM_152283 | ZFP62 | -0.74 | NM_022083 | C1ORF24 | -0.74 |
| NM_194251 | GPR151 | -0.74 | NM_080830 | CST11 | -0.74 |
| NM_152556 | FLJ31818 | -0.74 | XM_087384 | LOC152098 | -0.74 |
| NM_152580 | FLJ32742 | -0.74 | NM_153371 | LNX2 | -0.74 |
| NM_173589 | FLJ35709 | -0.74 | XM_370613 | LOC387755 | -0.74 |
| XM_373562 | LOC387917 | -0.74 | XM_373636 | LOC388140 | -0.74 |
| XM_372663 | LOC390777 | -0.74 | NM_001001691 | FLJ44790 | -0.74 |
| NM_214710 | PRSSL1 | -0.74 | NM_002654 | PKM2 | -0.73 |
| NM_000798 | DRD5 | -0.73 | NM_002944 | ROS1 | -0.73 |
| NM_012474 | UMPK | -0.73 | NM_002926 | RGS12 | -0.73 |
| NM_001940 | DRPLA | -0.73 | NM_019619 | PARD3 | -0.73 |
| NM_006383 | CIB2 | -0.73 | NM_021151 | CROT | -0.73 |
| NM_005657 | TP53BP1 | -0.73 | NM_000234 | LIG1 | -0.73 |
| NM_021129 | PP | -0.73 | NM_003947 | KALRN | -0.73 |
| NM_002342 | LTBR | -0.73 | NM_000319 | PEX5 | -0.73 |
| NM_007123 | USH2A | -0.73 | NM_004632 | DAP3 | -0.73 |
| NM_000611 | CD59 | -0.73 | NM_004233 | CD83 | -0.73 |
| NM_006894 | FMO3 | -0.73 | NM_024430 | PSTPIP2 | -0.73 |
| NM_001030 | RPS27 | -0.73 | NM_015488 | MR-1 | -0.73 |
| NM_005017 | PCYT1A | -0.73 | NM_014279 | OLFM1 | -0.73 |
| NM_148921 | EPN2 | -0.73 | NM_012205 | HAAO | -0.73 |
| NM_006588 | SULT1C2 | -0.73 | NM_015640 | PAI-RBP1 | -0.73 |
| NM_012388 | PLDN | -0.73 | NM_017510 | HSGP25L2G | -0.73 |
| NM_197958 | FLJ11196 | -0.73 | XM_375087 | PLEKHH1 | -0.73 |
| XM_038567 | MTA3 | -0.73 | NM_024812 | BAALC | -0.73 |
| NM_032271 | TRAF7 | -0.73 | NM_057161 | KLHDC3 | -0.73 |

| | | | | | |
|---|---|---|---|---|---|
| NM_138290 | RPIB9 | -0.73 | NM_015243 | COH1 | -0.73 |
| XM_370769 | LOC161394 | -0.73 | NM_153244 | C10ORF111 | -0.73 |
| NM_153184 | IGSF4D | -0.73 | NM_203424 | LOC389123 | -0.73 |
| XM_294997 | LOC339541 | -0.73 | XM_042178 | LOC152015 | -0.73 |
| XM_294666 | LOC338616 | -0.73 | XM_374025 | LOC389077 | -0.73 |
| XM_372979 | LOC391498 | -0.73 | XM_372838 | LOC391205 | -0.73 |
| XM_379668 | LOC401552 | -0.73 | XM_378625 | LOC400553 | -0.73 |
| XM_371026 | LOC388351 | -0.73 | XM_375634 | LOC400709 | -0.73 |
| XM_379023 | LOC400876 | -0.73 | XM_042066 | MAP3K1 | -0.72 |
| NM_031432 | UCK1 | -0.72 | NM_000845 | GRM8 | -0.72 |
| NM_023920 | TAS2R13 | -0.72 | NM_006569 | CGREF1 | -0.72 |
| NM_053281 | DACH2 | -0.72 | NM_006479 | PIR51 | -0.72 |
| NM_003406 | YWHAZ | -0.72 | NM_033029 | LMLN | -0.72 |
| NM_004582 | RABGGTB | -0.72 | NM_001842 | CNTFR | -0.72 |
| NM_170743 | IL28RA | -0.72 | NM_004242 | HMGN3 | -0.72 |
| NM_002208 | ITGAE | -0.72 | NM_000744 | CHRNA4 | -0.72 |
| NM_001847 | COL4A6 | -0.72 | NM_019644 | ANKRD7 | -0.72 |
| NM_002233 | KCNA4 | -0.72 | NM_023929 | ZBTB10 | -0.72 |
| NM_004988 | MAGEA1 | -0.72 | NM_003441 | ZNF141 | -0.72 |
| NM_003296 | CRISP2 | -0.72 | NM_004712 | HGS | -0.72 |
| NM_014690 | KIAA0773 | -0.72 | NM_006803 | AP3M2 | -0.72 |
| NM_013325 | APG4B | -0.72 | NM_170601 | CSE-C | -0.72 |
| NM_017516 | RAB39 | -0.72 | NM_018421 | TBC1D2 | -0.72 |
| NM_019091 | PLEKHA3 | -0.72 | NM_023926 | FLJ12895 | -0.72 |
| NM_024814 | CBLL1 | -0.72 | NM_030645 | KIAA1720 | -0.72 |
| NM_025109 | MYOHD1 | -0.72 | NM_032233 | C14ORF154 | -0.72 |
| NM_198443 | UNQ2446 | -0.72 | NM_144611 | MGC32124 | -0.72 |
| NM_178858 | SFXN2 | -0.72 | NM_174899 | FBXO36 | -0.72 |
| NM_080748 | C20ORF52 | -0.72 | XM_088566 | KIAA1958 | -0.72 |
| NM_174921 | LOC201895 | -0.72 | NM_173800 | FLJ90650 | -0.72 |
| NM_198281 | LOC285513 | -0.72 | NM_182585 | DKFZP451M2119 | -0.72 |
| XM_376212 | LOC339862 | -0.72 | NM_014788 | TRIM14 | -0.72 |
| XM_376902 | LOC401548 | -0.72 | XM_374817 | LOC399788 | -0.72 |
| XM_374326 | LOC389858 | -0.72 | NM_015533 | DKFZP586B1621 | -0.71 |
| NM_032409 | PINK1 | -0.71 | NM_000025 | ADRB3 | -0.71 |
| NM_002440 | MSH4 | -0.71 | XM_290809 | TAF4B | -0.71 |
| NM_012482 | ZNF281 | -0.71 | NM_000545 | TCF1 | -0.71 |
| NM_000690 | ALDH2 | -0.71 | NM_003753 | EIF3S7 | -0.71 |
| NM_016216 | DBR1 | -0.71 | NM_000237 | LPL | -0.71 |
| NM_002838 | PTPRC | -0.71 | NM_006990 | WASF2 | -0.71 |
| NM_012098 | ANGPTL2 | -0.71 | NM_015848 | HUMCYT2A | -0.71 |
| NM_170707 | LMNA | -0.71 | NM_025134 | CHD9 | -0.71 |
| NM_000134 | FABP2 | -0.71 | NM_020967 | NCOA5 | -0.71 |
| NM_005843 | STAM2 | -0.71 | NM_006005 | WFS1 | -0.71 |
| NM_002245 | KCNK1 | -0.71 | NM_003119 | SPG7 | -0.71 |
| NM_004600 | SSA2 | -0.71 | NM_015919 | ZNF226 | -0.71 |

| | | | | | |
|---|---|---|---|---|---|
| NM_006586 | TNRC5 | -0.71 | NM_014043 | DKFZP564O123 | -0.71 |
| NM_018561 | USP49 | -0.71 | NM_013356 | SLC16A8 | -0.71 |
| NM_016436 | C20ORF104 | -0.71 | NM_017858 | FLJ20516 | -0.71 |
| NM_020378 | KLP1 | -0.71 | NM_019843 | EIF4ENIF1 | -0.71 |
| NM_017519 | ARID1B | -0.71 | NM_022168 | MDA5 | -0.71 |
| NM_024068 | MGC2731 | -0.71 | XM_376680 | KIAA1718 | -0.71 |
| NM_025236 | RNF39 | -0.71 | NM_032168 | FLJ12519 | -0.71 |
| NM_033439 | C9ORF26 | -0.71 | NM_080654 | NY-REN-41 | -0.71 |
| NM_020896 | OSBPL5 | -0.71 | NM_052868 | IGSF8 | -0.71 |
| NM_139169 | TRUB1 | -0.71 | XM_097265 | LOC147670 | -0.71 |
| NM_153269 | C20ORF96 | -0.71 | XM_087928 | LOC154449 | -0.71 |
| NM_145301 | LOC201158 | -0.71 | XM_378951 | LOC284628 | -0.71 |
| NM_207349 | LOC284739 | -0.71 | NM_207356 | LOC339448 | -0.71 |
| XM_379459 | LOC401289 | -0.71 | XM_372726 | LOC390927 | -0.71 |
| XM_373715 | LOC388340 | -0.71 | NM_003647 | DGKE | -0.7 |
| NM_002020 | FLT4 | -0.7 | NM_016440 | VRK3 | -0.7 |
| NM_019012 | PEPP2 | -0.7 | NM_000332 | SCA1 | -0.7 |
| NM_004837 | GGPS1 | -0.7 | NM_000401 | EXT2 | -0.7 |
| NM_002372 | MAN2A1 | -0.7 | NM_000884 | IMPDH2 | -0.7 |
| NM_000954 | PTGDS | -0.7 | NM_001110 | ADAM10 | -0.7 |
| NM_007102 | GUCA2B | -0.7 | NM_030912 | TRIM8 | -0.7 |
| NM_003145 | SSR2 | -0.7 | NM_022342 | KIF9 | -0.7 |
| NM_004633 | IL1R2 | -0.7 | NM_006181 | NTN2L | -0.7 |
| NM_003003 | SEC14L1 | -0.7 | NM_000035 | ALDOB | -0.7 |
| NM_000336 | SCNN1B | -0.7 | NM_004063 | CDH17 | -0.7 |
| NM_152945 | DRB1 | -0.7 | NM_024830 | FLJ12443 | -0.7 |
| NM_005317 | GZMM | -0.7 | NM_004616 | TM4SF3 | -0.7 |
| NM_003905 | APPBP1 | -0.7 | NM_014719 | KIAA0738 | -0.7 |
| NM_006351 | TIMM44 | -0.7 | NM_015346 | ZFYVE26 | -0.7 |
| NM_018125 | FLJ10521 | -0.7 | NM_021219 | JAM2 | -0.7 |
| NM_021174 | DBC-1 | -0.7 | XM_046581 | ZSWIM5 | -0.7 |
| NM_025044 | BICC1 | -0.7 | NM_031466 | T1 | -0.7 |
| NM_031490 | LONP | -0.7 | NM_033413 | MGC16309 | -0.7 |
| NM_183075 | CYP2U1 | -0.7 | NM_138431 | LOC113655 | -0.7 |
| NM_033396 | TNKS1BP1 | -0.7 | NM_052854 | CREB3L1 | -0.7 |
| NM_138436 | LOC114926 | -0.7 | NM_013329 | C21ORF66 | -0.7 |
| NM_080764 | SUHW2 | -0.7 | NM_133468 | BMPER | -0.7 |
| XM_209640 | LOC285501 | -0.7 | XM_290838 | LOC339324 | -0.7 |
| NM_181787 | LOC286148 | -0.7 | XM_295097 | LOC339902 | -0.7 |
| XM_376318 | LOC401132 | -0.7 | XM_379817 | LOC402487 | -0.7 |
| XM_373315 | LOC392390 | -0.7 | XM_378350 | LOC400027 | -0.7 |
| XM_373909 | LOC388790 | -0.7 | NM_004517 | ILK | -0.69 |
| NM_002376 | MARK3 | -0.69 | NM_001703 | BAI2 | -0.69 |
| NM_005470 | ABI1 | -0.69 | NM_000529 | MC2R | -0.69 |
| NM_015508 | TIPARP | -0.69 | NM_000892 | KLKB1 | -0.69 |
| NM_022051 | EGLN1 | -0.69 | NM_001145 | ANG | -0.69 |

| | | | | | |
|---|---|---|---|---|---|
| NM_000448 | RAG1 | -0.69 | NM_012419 | RGS17 | -0.69 |
| NM_000689 | ALDH1A1 | -0.69 | NM_003842 | TNFRSF10B | -0.69 |
| NM_022835 | PLEKHG2 | -0.69 | NM_018147 | FAIM | -0.69 |
| NM_003392 | WNT5A | -0.69 | NM_001859 | SLC31A1 | -0.69 |
| NM_004373 | COX6A1 | -0.69 | NM_001891 | CSN2 | -0.69 |
| NM_000777 | CYP3A5 | -0.69 | NM_001533 | HNRPL | -0.69 |
| NM_006148 | LASP1 | -0.69 | NM_173710 | MTND3 | -0.69 |
| NM_080658 | ACY-3 | -0.69 | NM_006030 | CACNA2D2 | -0.69 |
| NM_012291 | ESPL1 | -0.69 | NM_014686 | KIAA0355 | -0.69 |
| XM_375812 | KIAA0907 | -0.69 | NM_015317 | PUM2 | -0.69 |
| NM_015461 | ZNF521 | -0.69 | NM_012458 | TIMM13 | -0.69 |
| XM_373313 | OR1J5 | -0.69 | NM_016518 | PIPOX | -0.69 |
| NM_019052 | C6ORF18 | -0.69 | NM_016098 | BRP44L | -0.69 |
| NM_018301 | FLJ11016 | -0.69 | NM_017546 | C2ORF29 | -0.69 |
| NM_020706 | SFRS15 | -0.69 | NM_020890 | KIAA1524 | -0.69 |
| NM_024660 | FLJ22573 | -0.69 | NM_033407 | DOCK7 | -0.69 |
| NM_138459 | C6ORF68 | -0.69 | NM_144600 | FLJ31153 | -0.69 |
| NM_153377 | LRIG3 | -0.69 | XM_060970 | PRRXL1 | -0.69 |
| NM_175054 | HIST4H4 | -0.69 | NM_183239 | GSTO2 | -0.69 |
| NM_153211 | C18ORF17 | -0.69 | NM_153456 | HS6ST3 | -0.69 |
| XM_379384 | LOC285766 | -0.69 | XM_378196 | FLJ36112 | -0.69 |
| XM_378207 | LOC338588 | -0.69 | XM_293092 | GPR148 | -0.69 |
| NM_005995 | TBX10 | -0.69 | NM_003516 | HIST2H2AA | -0.69 |
| NM_203306 | MGC39606 | -0.69 | XM_371488 | LOC388939 | -0.69 |
| XM_374167 | LOC389390 | -0.69 | XM_376586 | LOC401314 | -0.69 |
| XM_378280 | LOC399876 | -0.69 | XM_373533 | LOC387859 | -0.69 |
| XM_378805 | LOC400701 | -0.69 | XM_370934 | LOC388210 | -0.69 |
| NM_001004340 | LOC440607 | -0.69 | XM_376001 | LOC400921 | -0.69 |
| NM_001002910 | CYP2D7P1 | -0.69 | NM_020965 | MAGI-3 | -0.68 |
| XM_060316 | OR2T1 | -0.68 | NM_005054 | RANBP2L1 | -0.68 |
| NM_005432 | XRCC3 | -0.68 | NM_014247 | RAPGEF2 | -0.68 |
| NM_004416 | DTX1 | -0.68 | XM_291314 | FBXO10 | -0.68 |
| NM_012174 | FBXW8 | -0.68 | NM_006612 | KIF1C | -0.68 |
| NM_003800 | RNGTT | -0.68 | NM_012275 | IL1F5 | -0.68 |
| NM_001244 | TNFSF8 | -0.68 | NM_006409 | ARPC1A | -0.68 |
| NM_000515 | GH1 | -0.68 | NM_002398 | MEIS1 | -0.68 |
| NM_004668 | MGAM | -0.68 | NM_052925 | LENG8 | -0.68 |
| NM_000200 | HTN3 | -0.68 | NM_005509 | DMXL1 | -0.68 |
| NM_005275 | GNL1 | -0.68 | NM_002652 | PIP | -0.68 |
| NM_005949 | MT1F | -0.68 | NM_018477 | ACTR10 | -0.68 |
| NM_021026 | RFPL1 | -0.68 | NM_003407 | ZFP36 | -0.68 |
| NM_007159 | SLMAP | -0.68 | NM_004234 | ZNF235 | -0.68 |
| NM_014699 | KIAA0296 | -0.68 | NM_014735 | PHF16 | -0.68 |
| NM_006385 | ZNF211 | -0.68 | NM_014950 | ZBTB1 | -0.68 |
| NM_025154 | UNC84A | -0.68 | NM_014320 | HEBP2 | -0.68 |
| NM_012322 | LSM5 | -0.68 | NM_015569 | DNM3 | -0.68 |

| | | |
|---|---|---|
| NM_013343 | LOH3CR2A | -0.68 |
| NM_018231 | FLJ10815 | -0.68 |
| NM_020964 | KIAA1632 | -0.68 |
| NM_025129 | FLJ22688 | -0.68 |
| NM_031306 | DKFZP564B1023 | -0.68 |
| XM_027074 | L3MBTL3 | -0.68 |
| XM_030559 | PARD6B | -0.68 |
| NM_052872 | IL17F | -0.68 |
| NM_172003 | CBWD2 | -0.68 |
| NM_032627 | SSBP4 | -0.68 |
| NM_173852 | KRTCAP2 | -0.68 |
| XM_371388 | DKFZP434D177-LIKE | -0.68 |
| NM_198687 | KRTAP18-4 | -0.68 |
| XM_211995 | LOC285721 | -0.68 |
| XM_378462 | LOC400221 | -0.68 |
| XM_371118 | LOC388478 | -0.68 |
| NM_002849 | PTPRR | -0.67 |
| NM_003600 | STK6 | -0.67 |
| NM_004624 | VIPR1 | -0.67 |
| NM_000505 | F12 | -0.67 |
| NM_004946 | DOCK2 | -0.67 |
| XM_171149 | MGC26484 | -0.67 |
| NM_000570 | FCGR3B | -0.67 |
| NM_005719 | ARPC3 | -0.67 |
| NM_005345 | HSPA1A | -0.67 |
| NM_006115 | PRAME | -0.67 |
| NM_005258 | GCHFR | -0.67 |
| NM_001010 | RPS6 | -0.67 |
| NM_003966 | SEMA5A | -0.67 |
| NM_005710 | PQBP1 | -0.67 |
| NM_015271 | TRIM2 | -0.67 |
| XM_048235 | HYPM | -0.67 |
| NM_018711 | DKFZP761H039 | -0.67 |
| NM_019852 | METTL3 | -0.67 |
| NM_022748 | TENS1 | -0.67 |
| NM_024810 | FLJ23018 | -0.67 |
| NM_030805 | LMAN2L | -0.67 |
| NM_032415 | CARD11 | -0.67 |
| NM_033129 | SCRT2 | -0.67 |
| XM_060020 | LOC139231 | -0.67 |
| NM_152528 | FLJ36175 | -0.67 |
| NM_145653 | TCEB3C | -0.67 |
| NM_020690 | MASK-BP3 | -0.67 |
| XM_032059 | LOC90485 | -0.67 |
| XM_377958 | LOC402289 | -0.67 |
| XM_373472 | LOC387709 | -0.67 |
| NM_019039 | DDX4 | -0.68 |
| NM_018996 | KIAA1582 | -0.68 |
| NM_024091 | MGC5297 | -0.68 |
| NM_032831 | C7ORF19 | -0.68 |
| NM_031421 | DKFZP434H0115 | -0.68 |
| NM_199249 | MGC13170 | -0.68 |
| NM_032926 | MGC15737 | -0.68 |
| NM_152400 | FLJ39370 | -0.68 |
| NM_153223 | FLJ36090 | -0.68 |
| NM_145029 | C6ORF136 | -0.68 |
| NM_181843 | NUDT8 | -0.68 |
| XM_370925 | LOC283951 | -0.68 |
| XM_115715 | LOC200493 | -0.68 |
| XM_373587 | LOC387981 | -0.68 |
| XM_378317 | LOC399961 | -0.68 |
| NM_020341 | PAK7 | -0.67 |
| NM_001741 | CALCA | -0.67 |
| NM_080865 | GPR62 | -0.67 |
| NM_001607 | ACAA1 | -0.67 |
| NM_021204 | MASA | -0.67 |
| NM_006242 | PPP1R3D | -0.67 |
| NM_000080 | CHRNE | -0.67 |
| NM_004757 | SCYE1 | -0.67 |
| XM_371332 | KIF21B | -0.67 |
| NM_014177 | HSPC154 | -0.67 |
| NM_003564 | TAGLN2 | -0.67 |
| NM_001937 | DPT | -0.67 |
| NM_003724 | JRK | -0.67 |
| NM_005467 | NAALAD2 | -0.67 |
| NM_012099 | ASE-1 | -0.67 |
| NM_015432 | PLEKHG4 | -0.67 |
| NM_017688 | BSPRY | -0.67 |
| NM_017964 | SLC30A6 | -0.67 |
| NM_020714 | ZNF490 | -0.67 |
| NM_024052 | C17ORF39 | -0.67 |
| NM_025260 | C6ORF25 | -0.67 |
| NM_031298 | MGC2963 | -0.67 |
| NM_018287 | ARHGAP12 | -0.67 |
| XM_071793 | C14ORF28 | -0.67 |
| NM_152473 | FLJ32214 | -0.67 |
| NM_014069 | PSORS1C2 | -0.67 |
| NM_207381 | FLJ41287 | -0.67 |
| NM_207464 | FLJ40008 | -0.67 |
| XM_171489 | LOC256144 | -0.67 |
| XM_379541 | LOC401441 | -0.67 |
| XM_378236 | LOC399786 | -0.67 |

| | | | | | |
|---|---|---|---|---|---|
| XM_372565 | LOC390569 | -0.67 | XM_375150 | LOC400301 | -0.67 |
| XM_371413 | LOC388822 | -0.67 | NM_001005327 | OR6K3 | -0.67 |
| NM_212551 | SB145 | -0.67 | NM_002982 | CCL2 | -0.66 |
| NM_001786 | CDC2 | -0.66 | XM_291304 | C9ORF96 | -0.66 |
| NM_002611 | PDK2 | -0.66 | NM_000289 | PFKM | -0.66 |
| NM_014006 | SMG1 | -0.66 | NM_002723 | PRB4 | -0.66 |
| NM_000960 | PTGIR | -0.66 | NM_016943 | TAS2R3 | -0.66 |
| NM_001794 | CDH4 | -0.66 | NM_004052 | BNIP3 | -0.66 |
| NM_022759 | FLJ21865 | -0.66 | NM_017775 | FLJ20343 | -0.66 |
| NM_006505 | PVR | -0.66 | NM_033389 | SSH2 | -0.66 |
| NM_002009 | FGF7 | -0.66 | NM_033655 | CNTNAP3 | -0.66 |
| NM_000258 | MYL3 | -0.66 | NM_004386 | CSPG3 | -0.66 |
| NM_004137 | KCNMB1 | -0.66 | NM_002349 | LY75 | -0.66 |
| NM_021048 | MAGEA10 | -0.66 | NM_002862 | PYGB | -0.66 |
| XM_033113 | KIAA0789 | -0.66 | NM_014787 | DNAJC6 | -0.66 |
| XM_033371 | C14ORF120 | -0.66 | XM_376189 | DKFZP586K1520 | -0.66 |
| NM_016032 | ZDHHC9 | -0.66 | NM_018116 | FLJ10504 | -0.66 |
| NM_018137 | HRMT1L6 | -0.66 | NM_018912 | PCDHGA1 | -0.66 |
| NM_022493 | NARFL | -0.66 | NM_024795 | FLJ22800 | -0.66 |
| NM_030924 | BGR | -0.66 | XM_373381 | H2AFB | -0.66 |
| NM_032135 | C14ORF155 | -0.66 | NM_032809 | C9ORF54 | -0.66 |
| NM_032498 | PEPP-2 | -0.66 | NM_033124 | NYD-SP28 | -0.66 |
| NM_138361 | LRSAM1 | -0.66 | NM_052882 | ZIM3 | -0.66 |
| NM_052871 | MGC4677 | -0.66 | NM_053055 | CTMP | -0.66 |
| NM_173482 | FLJ40365 | -0.66 | NM_144652 | FLJ25409 | -0.66 |
| NM_152423 | FLJ33516 | -0.66 | NM_173495 | FLJ30296 | -0.66 |
| NM_152549 | MGC39633 | -0.66 | NM_182530 | FLJ25056 | -0.66 |
| XM_166443 | TDRD6 | -0.66 | NM_145010 | C10ORF63 | -0.66 |
| NM_145702 | TIGD1 | -0.66 | NM_145023 | CCDC7 | -0.66 |
| NM_144999 | MGC20806 | -0.66 | NM_203373 | FBXL22 | -0.66 |
| NM_198498 | MGC50104 | -0.66 | NM_207367 | FLJ42291 | -0.66 |
| NM_207414 | FLJ43860 | -0.66 | NM_207478 | FLJ44385 | -0.66 |
| XM_085967 | LOC147942 | -0.66 | XM_092019 | LOC163131 | -0.66 |
| XM_210613 | LOC285563 | -0.66 | XM_294209 | LOC340260 | -0.66 |
| XM_378776 | LOC400675 | -0.66 | XM_380013 | LOC402671 | -0.66 |
| XM_380020 | LOC402676 | -0.66 | NM_004967 | IBSP | -0.65 |
| NM_005936 | MLLT4 | -0.65 | NM_004426 | PHC1 | -0.65 |
| NM_015944 | CGI-14 | -0.65 | NM_014294 | TRAM1 | -0.65 |
| NM_003594 | TTF2 | -0.65 | NM_001139 | ALOX12B | -0.65 |
| NM_021235 | EPS15L1 | -0.65 | NM_004198 | CHRNA6 | -0.65 |
| NM_000433 | NCF2 | -0.65 | NM_001446 | FABP7 | -0.65 |
| NM_020452 | ATP8B2 | -0.65 | NM_004929 | CALB1 | -0.65 |
| NM_013279 | C11ORF9 | -0.65 | NM_001961 | EEF2 | -0.65 |
| NM_006277 | ITSN2 | -0.65 | NM_001095 | ACCN2 | -0.65 |
| XM_047025 | OATL1 | -0.65 | NM_004776 | B4GALT5 | -0.65 |
| NM_014725 | STARD8 | -0.65 | NM_005776 | CNIH | -0.65 |

| | | | | | |
|---|---|---|---|---|---|
| NM_012289 | KEAP1 | -0.65 | NM_007243 | NRM | -0.65 |
| NM_015157 | PHLDB1 | -0.65 | NM_012198 | GCA | -0.65 |
| NM_012135 | D6S2654E | -0.65 | NM_015950 | MRPL2 | -0.65 |
| NM_016114 | ASB1 | -0.65 | NM_016209 | LOC51693 | -0.65 |
| NM_017626 | DNAJB12 | -0.65 | NM_017863 | FLJ20527 | -0.65 |
| NM_021156 | DJ971N18.2 | -0.65 | NM_018688 | BIN3 | -0.65 |
| XM_027330 | RBM25 | -0.65 | XM_051699 | KIAA1344 | -0.65 |
| NM_024699 | FLJ14007 | -0.65 | NM_032932 | RAB11-FIP4 | -0.65 |
| NM_033426 | KIAA1737 | -0.65 | NM_213599 | TMEM16E | -0.65 |
| NM_173596 | SLC39A5 | -0.65 | XM_371311 | FLJ36032 | -0.65 |
| XM_378855 | LOC339442 | -0.65 | XM_211339 | LOC284120 | -0.65 |
| XM_097278 | LOC147710 | -0.65 | XM_374945 | LOC399968 | -0.65 |
| XM_372584 | LOC390616 | -0.65 | NM_001001794 | MGC33692 | -0.65 |
| XM_039796 | TNIK | -0.64 | NM_014566 | OR1D5 | -0.64 |
| NM_032971 | PCDH11Y | -0.64 | NM_006169 | NNMT | -0.64 |
| NM_006225 | PLCD1 | -0.64 | NM_000213 | ITGB4 | -0.64 |
| NM_016523 | KLRF1 | -0.64 | NM_000575 | IL1A | -0.64 |
| NM_003822 | NR5A2 | -0.64 | NM_012281 | KCND2 | -0.64 |
| NM_030766 | BCL2L14 | -0.64 | NM_001586 | CXORF2 | -0.64 |
| NM_000848 | GSTM2 | -0.64 | NM_002112 | HDC | -0.64 |
| NM_004538 | NAP1L3 | -0.64 | NM_003059 | SLC22A4 | -0.64 |
| NM_023068 | SN | -0.64 | NM_021796 | PLAC1 | -0.64 |
| XM_375181 | KIAA1018 | -0.64 | NM_015966 | SDBCAG84 | -0.64 |
| NM_017746 | TEX10 | -0.64 | NM_018913 | PCDHGA10 | -0.64 |
| NM_020808 | SIPA1L2 | -0.64 | NM_022469 | FLJ21195 | -0.64 |
| NM_025180 | FLJ13386 | -0.64 | XM_370692 | LOC121006 | -0.64 |
| NM_182507 | LOC144501 | -0.64 | NM_173496 | MPP7 | -0.64 |
| NM_178827 | FLJ35834 | -0.64 | NM_198274 | SMYD1 | -0.64 |
| NM_130899 | MGC26988 | -0.64 | NM_152640 | DCP1B | -0.64 |
| NM_178862 | SIMP | -0.64 | NM_173811 | FLJ32675 | -0.64 |
| XM_371187 | MGC45922 | -0.64 | XM_379054 | LOC339674 | -0.64 |
| XM_371215 | LOC388585 | -0.64 | XM_114355 | LOC201651 | -0.64 |
| XM_295034 | LOC339693 | -0.64 | XM_371820 | LOC389389 | -0.64 |
| NM_024110 | CARD14 | -0.63 | NM_002984 | CCL4 | -0.63 |
| NM_002625 | PFKFB1 | -0.63 | NM_005302 | GPR37 | -0.63 |
| NM_003552 | OR1D4 | -0.63 | NM_001557 | IL8RB | -0.63 |
| NM_000242 | MBL2 | -0.63 | NM_004349 | CBFA2T1 | -0.63 |
| NM_005315 | GSCL | -0.63 | NM_002505 | NFYA | -0.63 |
| NM_021923 | FGFRL1 | -0.63 | NM_002451 | MTAP | -0.63 |
| NM_000348 | SRD5A2 | -0.63 | NM_181745 | GPR120 | -0.63 |
| NM_003604 | IRS4 | -0.63 | NM_000618 | IGF1 | -0.63 |
| NM_000068 | CACNA1A | -0.63 | NM_005925 | MEP1B | -0.63 |
| NM_025136 | OPA3 | -0.63 | NM_000023 | SGCA | -0.63 |
| NM_004338 | C18ORF1 | -0.63 | NM_001307 | CLDN7 | -0.63 |
| NM_020056 | HLA-DQA2 | -0.63 | NM_002456 | MUC1 | -0.63 |
| NM_145035 | ADMP | -0.63 | NM_013249 | ZNF214 | -0.63 |

| | | | | | |
|---|---|---|---|---|---|
| NM_003531 | HIST1H3C | -0.63 | NM_006300 | ZNF230 | -0.63 |
| NM_003951 | SLC25A14 | -0.63 | NM_004790 | SLC22A6 | -0.63 |
| NM_005772 | RCL1 | -0.63 | NM_015203 | KIAA0460 | -0.63 |
| NM_015511 | C20ORF4 | -0.63 | NM_148571 | MRPL27 | -0.63 |
| NM_016122 | NY-REN-58 | -0.63 | NM_016065 | MRPS16 | -0.63 |
| NM_018973 | DPM3 | -0.63 | NM_018434 | RNF130 | -0.63 |
| NM_018530 | GSDML | -0.63 | NM_018917 | PCDHGA4 | -0.63 |
| XM_291085 | CDGAP | -0.63 | NM_022354 | SPATA1 | -0.63 |
| NM_022369 | FLJ12541 | -0.63 | NM_022749 | RAI16 | -0.63 |
| NM_022750 | ZC3HDC1 | -0.63 | NM_022751 | C18ORF11 | -0.63 |
| NM_022115 | PRDM15 | -0.63 | NM_024535 | CORO7 | -0.63 |
| NM_032413 | NMES1 | -0.63 | XM_055095 | KIAA1906 | -0.63 |
| NM_178812 | LYRIC | -0.63 | NM_139072 | DNER | -0.63 |
| NM_080742 | B3GAT2 | -0.63 | NM_130768 | C7ORF7 | -0.63 |
| NM_152445 | C14ORF44 | -0.63 | NM_178470 | WDR40B | -0.63 |
| NM_133498 | SPACA4 | -0.63 | XM_114685 | C9ORF21 | -0.63 |
| NM_153289 | DEFB119 | -0.63 | NM_198279 | CXORF23 | -0.63 |
| NM_145865 | FLJ38819 | -0.63 | NM_198474 | UNQ564 | -0.63 |
| NM_147156 | TMEM23 | -0.63 | NM_152775 | MGC33607 | -0.63 |
| XM_292160 | MGC75495 | -0.63 | NM_181539 | KRT25B | -0.63 |
| XM_372145 | LOC389792 | -0.63 | XM_060580 | LOC127623 | -0.63 |
| XM_007651 | LOC116166 | -0.63 | XM_292512 | LOC342371 | -0.63 |
| XM_372996 | LOC391555 | -0.63 | XM_371350 | LOC388753 | -0.63 |
| XM_374682 | LOC393045 | -0.63 | XM_378746 | LOC400649 | -0.63 |
| NM_005372 | MOS | -0.62 | NM_000443 | ABCB4 | -0.62 |
| NM_001164 | APBB1 | -0.62 | NM_006734 | HIVEP2 | -0.62 |
| NM_016580 | PCDH12 | -0.62 | NM_016162 | ING4 | -0.62 |
| NM_002485 | NBS1 | -0.62 | NM_000346 | SOX9 | -0.62 |
| NM_005100 | AKAP12 | -0.62 | NM_005099 | ADAMTS4 | -0.62 |
| NM_001229 | CASP9 | -0.62 | NM_012210 | TRIM32 | -0.62 |
| NM_153837 | GPR114 | -0.62 | NM_058238 | WNT7B | -0.62 |
| NM_001037 | SCN1B | -0.62 | NM_003568 | ANXA9 | -0.62 |
| NM_001085 | SERPINA3 | -0.62 | NM_002433 | MOG | -0.62 |
| NM_006949 | STXBP2 | -0.62 | NM_007354 | GR6 | -0.62 |
| NM_016096 | LOC51123 | -0.62 | NM_016591 | C2GNT3 | -0.62 |
| NM_016355 | DDX47 | -0.62 | NM_017717 | MUCDHL | -0.62 |
| NM_018992 | KCTD5 | -0.62 | NM_018439 | IMPACT | -0.62 |
| NM_020442 | VARS2L | -0.62 | NM_023077 | FLJ12439 | -0.62 |
| XM_291223 | MYO1G | -0.62 | NM_024784 | ZBTB3 | -0.62 |
| XM_375569 | DKFZP434I1610 | -0.62 | NM_145231 | C14ORF143 | -0.62 |
| NM_033513 | C19ORF20 | -0.62 | NM_152274 | MGC29729 | -0.62 |
| XM_371259 | DKFZP547I048 | -0.62 | XM_379205 | LOC151658 | -0.62 |
| NM_194290 | LOC153684 | -0.62 | NM_152609 | FLJ32001 | -0.62 |
| NM_182538 | MGC29671 | -0.62 | XM_173173 | AOF1 | -0.62 |
| NM_152753 | SCUBE3 | -0.62 | NM_145179 | C21ORF93 | -0.62 |
| XM_376419 | LOC285638 | -0.62 | XM_291144 | LOC340089 | -0.62 |

| | | | | | |
|---|---|---|---|---|---|
| XM_376101 | LOC400987 | -0.62 | XM_377877 | LOC402200 | -0.62 |
| XM_380108 | LOC402506 | -0.62 | XM_374835 | LOC399814 | -0.62 |
| XM_373571 | LOC387940 | -0.62 | NM_005211 | CSF1R | -0.61 |
| NM_002736 | PRKAR2B | -0.61 | NM_006212 | PFKFB2 | -0.61 |
| NM_003242 | TGFBR2 | -0.61 | NM_003331 | TYK2 | -0.61 |
| NM_019888 | MC3R | -0.61 | NM_000539 | RHO | -0.61 |
| NM_004705 | PRKRIR | -0.61 | NM_001965 | EGR4 | -0.61 |
| NM_006792 | MORF4 | -0.61 | NM_003410 | ZFX | -0.61 |
| NM_021966 | TCL1A | -0.61 | XM_042698 | USP22 | -0.61 |
| NM_002564 | P2RY2 | -0.61 | NM_006579 | EBP | -0.61 |
| NM_000944 | PPP3CA | -0.61 | NM_000590 | IL9 | -0.61 |
| NM_001035 | RYR2 | -0.61 | NM_002381 | MATN3 | -0.61 |
| NM_007285 | GABARAPL2 | -0.61 | NM_004487 | GOLGB1 | -0.61 |
| NM_020947 | KIAA1609 | -0.61 | NM_005357 | LIPE | -0.61 |
| NM_000239 | LYZ | -0.61 | NM_001828 | CLC | -0.61 |
| NM_001622 | AHSG | -0.61 | NM_001001 | RPL36AL | -0.61 |
| NM_007077 | AP4S1 | -0.61 | NM_006647 | NOXA1 | -0.61 |
| NM_006651 | CPLX1 | -0.61 | NM_015571 | SENP6 | -0.61 |
| NM_015470 | GAF1 | -0.61 | NM_012329 | MMD | -0.61 |
| NM_014344 | FJX1 | -0.61 | NM_032217 | ANKRD17 | -0.61 |
| XM_212241 | DKFZP434M131 | -0.61 | NM_016583 | PLUNC | -0.61 |
| NM_018838 | DAP13 | -0.61 | NM_024692 | FLJ21069 | -0.61 |
| NM_030938 | VMP1 | -0.61 | NM_032280 | ZCCHC9 | -0.61 |
| NM_032322 | RNF135 | -0.61 | XM_371484 | KIAA1724 | -0.61 |
| NM_152282 | FLJ23751 | -0.61 | NM_177531 | PKHD1L1 | -0.61 |
| NM_139015 | SPPL3 | -0.61 | NM_080604 | TJP4 | -0.61 |
| NM_130469 | JDP2 | -0.61 | NM_152359 | CPT1C | -0.61 |
| XM_378251 | LOC143188 | -0.61 | XM_379133 | LOC151121 | -0.61 |
| NM_152501 | IFIX | -0.61 | NM_130769 | GPHA2 | -0.61 |
| NM_153226 | TMEM20 | -0.61 | NM_152614 | MGC35154 | -0.61 |
| NM_152725 | SLC39A12 | -0.61 | NM_173643 | DKFZP547G0215 | -0.61 |
| NM_173661 | FLJ35424 | -0.61 | NM_207355 | POTE15 | -0.61 |
| NM_207387 | FLJ35696 | -0.61 | NM_207423 | FLJ45983 | -0.61 |
| XM_374037 | LOC389105 | -0.61 | XM_371678 | LOC389174 | -0.61 |
| XM_373489 | LOC387746 | -0.61 | XM_373650 | LOC388180 | -0.61 |
| XM_370893 | LOC388159 | -0.61 | NM_001001917 | OR56A1 | -0.61 |
| XM_378155 | LOC402434 | -0.61 | NM_152572 | C9ORF98 | -0.6 |
| NM_004431 | EPHA2 | -0.6 | NM_004440 | EPHA7 | -0.6 |
| NM_004742 | BAIAP1 | -0.6 | NM_005392 | PHF2 | -0.6 |
| NM_004785 | SLC9A3R2 | -0.6 | NM_004097 | EMX1 | -0.6 |
| NM_012183 | FOXD3 | -0.6 | NM_006164 | NFE2L2 | -0.6 |
| NM_002919 | RFX3 | -0.6 | NM_006713 | PC4 | -0.6 |
| NM_006706 | TCERG1 | -0.6 | NM_000030 | AGXT | -0.6 |
| NM_001673 | ASNS | -0.6 | NM_016230 | NCB5OR | -0.6 |
| XM_376059 | SERTAD2 | -0.6 | NM_022110 | FKBPL | -0.6 |
| NM_000100 | CSTB | -0.6 | NM_002712 | PPP1R7 | -0.6 |

| | | | | | |
|---|---|---|---|---|---|
| NM_002067 | GNA11 | -0.6 | NM_003363 | USP4 | -0.6 |
| NM_002507 | NGFR | -0.6 | NM_004725 | BUB3 | -0.6 |
| NM_006870 | DSTN | -0.6 | NM_002823 | PTMA | -0.6 |
| NM_005203 | COL13A1 | -0.6 | NM_004481 | GALNT2 | -0.6 |
| NM_014458 | AB026190 | -0.6 | NM_024866 | ADM2 | -0.6 |
| NM_003116 | SPAG4 | -0.6 | NM_005114 | HS3ST1 | -0.6 |
| NM_015039 | NMNAT2 | -0.6 | NM_007360 | KLRK1 | -0.6 |
| NM_020765 | RBAF600 | -0.6 | NM_015642 | ZNF288 | -0.6 |
| NM_015644 | TTLL3 | -0.6 | NM_020637 | FGF22 | -0.6 |
| XM_089081 | DNB5 | -0.6 | NM_016930 | STX18 | -0.6 |
| NM_017512 | HSRTSBETA | -0.6 | XM_046531 | KIAA1614 | -0.6 |
| NM_021627 | SENP2 | -0.6 | NM_024029 | MGC3262 | -0.6 |
| NM_024746 | FLJ13840 | -0.6 | NM_024112 | C9ORF16 | -0.6 |
| NM_024940 | DOCK5 | -0.6 | XM_030378 | ZNF527 | -0.6 |
| NM_080737 | SYTL4 | -0.6 | NM_198147 | LOC116236 | -0.6 |
| NM_133370 | YT521 | -0.6 | NM_178867 | SFXN4 | -0.6 |
| NM_144616 | FLJ32416 | -0.6 | NM_152449 | FLJ33008 | -0.6 |
| NM_178232 | HAPLN3 | -0.6 | NM_144705 | MGC27019 | -0.6 |
| NM_153232 | CRI2 | -0.6 | NM_144982 | MGC23401 | -0.6 |
| XM_113743 | TMEM16F | -0.6 | XM_371588 | LOC389069 | -0.6 |
| XM_379637 | LOC401513 | -0.6 | XM_380155 | LOC402626 | -0.6 |
| XM_374269 | LOC389657 | -0.6 | XM_378043 | LOC402353 | -0.6 |
| XM_370776 | LOC388015 | -0.6 | XM_372343 | LOC390031 | -0.6 |
| XM_370868 | LOC388125 | -0.6 | NM_001004343 | LOC440738 | -0.6 |
| XM_374379 | LOC392583 | -0.6 | NM_001005324 | OR10V1 | -0.6 |
| NM_213607 | LOC388389 | -0.6 | NM_001296 | CCBP2 | -0.59 |
| NM_000740 | CHRM3 | -0.59 | XM_291437 | OR1C1 | -0.59 |
| NM_000233 | LHCGR | -0.59 | NM_001809 | CENPA | -0.59 |
| NM_004726 | REPS2 | -0.59 | NM_004143 | CITED1 | -0.59 |
| NM_013258 | ASC | -0.59 | NM_006162 | NFATC1 | -0.59 |
| NM_005413 | SIX3 | -0.59 | NM_015143 | METAP1 | -0.59 |
| NM_001126 | ADSS | -0.59 | NM_013378 | VPREB3 | -0.59 |
| NM_002083 | GPX2 | -0.59 | NM_021201 | MS4A7 | -0.59 |
| NM_030640 | DUSP16 | -0.59 | NM_000639 | TNFSF6 | -0.59 |
| NM_032414 | PROK1 | -0.59 | NM_000737 | CGB | -0.59 |
| NM_005446 | P2RXL1 | -0.59 | NM_013401 | RAB3IL1 | -0.59 |
| NM_001115 | ADCY8 | -0.59 | NM_002375 | MAP4 | -0.59 |
| NM_019624 | ABCB9 | -0.59 | NM_003090 | SNRPA1 | -0.59 |
| NM_013309 | SLC30A4 | -0.59 | NM_004212 | SLC28A2 | -0.59 |
| NM_015275 | KIAA1033 | -0.59 | NM_014175 | MRPL15 | -0.59 |
| NM_016097 | HSPC039 | -0.59 | NM_016606 | C5ORF19 | -0.59 |
| NM_016357 | EPLIN | -0.59 | NM_022050 | SCAND2 | -0.59 |
| XM_377955 | ANKIB1 | -0.59 | NM_020433 | JPH2 | -0.59 |
| NM_021254 | C21ORF59 | -0.59 | NM_024059 | MGC5356 | -0.59 |
| NM_080864 | RLN3 | -0.59 | NM_032646 | TTYH2 | -0.59 |
| NM_198988 | LENG9 | -0.59 | NM_181776 | SLC36A2 | -0.59 |

| | | | | | |
|---|---|---|---|---|---|
| NM_145306 | C10ORF35 | -0.59 | NM_201566 | SLC16A13 | -0.59 |
| NM_173673 | FLJ34503 | -0.59 | NM_198540 | B3GALT7 | -0.59 |
| NM_207405 | FLJ46481 | -0.59 | NM_207510 | FLJ45224 | -0.59 |
| XM_170597 | LOC256374 | -0.59 | XM_375174 | LOC400340 | -0.59 |
| XM_378321 | LOC399972 | -0.59 | XM_373686 | LOC388277 | -0.59 |
| NM_004877 | GMFG | -0.58 | NM_000221 | KHK | -0.58 |
| NM_005864 | EFS | -0.58 | NM_014325 | CORO1C | -0.58 |
| NM_006227 | PLTP | -0.58 | NM_006991 | ZNF197 | -0.58 |
| NM_005541 | INPP5D | -0.58 | NM_000341 | SLC3A1 | -0.58 |
| NM_022648 | TNS | -0.58 | NM_004841 | RASAL2 | -0.58 |
| NM_002975 | SCGF | -0.58 | NM_004464 | FGF5 | -0.58 |
| NM_019010 | KRT20 | -0.58 | NM_000126 | ETFA | -0.58 |
| NM_004900 | APOBEC3B | -0.58 | NM_018136 | ASPM | -0.58 |
| NM_033378 | CGB2 | -0.58 | NM_005860 | FSTL3 | -0.58 |
| NM_015008 | KIAA0779 | -0.58 | NM_001172 | ARG2 | -0.58 |
| NM_004501 | HNRPU | -0.58 | NM_002319 | LRCH4 | -0.58 |
| NM_003486 | SLC7A5 | -0.58 | NM_016831 | PER3 | -0.58 |
| NM_003794 | SNX4 | -0.58 | XM_376203 | TATDN2 | -0.58 |
| XM_043493 | SV2C | -0.58 | NM_015605 | DKFZP566K0524 | -0.58 |
| NM_014419 | DKKL1-PENDING | -0.58 | NM_015687 | FILIP1 | -0.58 |
| NM_016521 | LOC51270 | -0.58 | NM_021616 | TRIM34 | -0.58 |
| NM_016056 | CGI-119 | -0.58 | NM_019096 | GTPBP2 | -0.58 |
| NM_017888 | FLJ20581 | -0.58 | NM_018252 | FLJ10874 | -0.58 |
| NM_020192 | C7ORF36 | -0.58 | NM_020755 | TDE2 | -0.58 |
| NM_022903 | FLJ12800 | -0.58 | NM_024089 | KDELC1 | -0.58 |
| NM_024094 | MGC5528 | -0.58 | NM_030569 | ITIH5 | -0.58 |
| NM_032793 | FLJ14490 | -0.58 | NM_032319 | C2ORF7 | -0.58 |
| NM_032631 | HDGF2 | -0.58 | NM_138395 | METRS | -0.58 |
| NM_053017 | ART5 | -0.58 | NM_021059 | HIST2H3C | -0.58 |
| NM_152428 | FRMPD2 | -0.58 | NM_175607 | CNTN4 | -0.58 |
| NM_178356 | LCE4A | -0.58 | NM_144992 | MGC26733 | -0.58 |
| NM_207351 | FLJ33674 | -0.58 | NM_178433 | LCE3B | -0.58 |
| NM_198532 | FLJ45778 | -0.58 | XM_293157 | LOC343705 | -0.58 |
| XM_372763 | LOC391003 | -0.58 | XM_379096 | LOC400955 | -0.58 |
| XM_379391 | LOC401230 | -0.58 | XM_372108 | LOC389748 | -0.58 |
| XM_373556 | LOC387898 | -0.58 | XM_375663 | LOC400720 | -0.58 |
| NM_001002255 | SUMO4 | -0.58 | NM_001736 | C5R1 | -0.57 |
| NM_145015 | MRGPRF | -0.57 | NM_013345 | GPR132 | -0.57 |
| NM_001326 | CSTF3 | -0.57 | NM_015460 | MYRIP | -0.57 |
| NM_005967 | NAB2 | -0.57 | NM_145285 | NKX2-3 | -0.57 |
| NM_001869 | CPA2 | -0.57 | NM_020661 | AICDA | -0.57 |
| NM_004117 | FKBP5 | -0.57 | NM_012116 | CBLC | -0.57 |
| NM_000260 | MYO7A | -0.57 | NM_001492 | GDF1 | -0.57 |
| NM_002989 | CCL21 | -0.57 | NM_013437 | LRP12 | -0.57 |
| NM_005910 | MAPT | -0.57 | NM_080657 | CIG5 | -0.57 |
| NM_003833 | MATN4 | -0.57 | NM_006799 | PRSS21 | -0.57 |

| | | | | | |
|---|---|---|---|---|---|
| NM_012447 | STAG3 | -0.57 | NM_015714 | G0S2 | -0.57 |
| NM_021035 | KIAA1404 | -0.57 | NM_021045 | ZNF248 | -0.57 |
| NM_020961 | KIAA1627 | -0.57 | NM_020857 | VPS18 | -0.57 |
| NM_025140 | FLJ22471 | -0.57 | NM_033199 | UCN2 | -0.57 |
| NM_183058 | LYZL2 | -0.57 | NM_152405 | JMY | -0.57 |
| NM_173485 | C20ORF17 | -0.57 | XM_085367 | FLJ40162 | -0.57 |
| NM_173826 | DKFZP313N0621 | -0.57 | XM_211108 | LOC283584 | -0.57 |
| XM_292122 | LOC338829 | -0.57 | XM_379175 | LOC401052 | -0.57 |
| XM_373042 | LOC391722 | -0.57 | XM_372345 | LOC390033 | -0.57 |
| XM_378617 | LOC400548 | -0.57 | XM_372586 | LOC390625 | -0.57 |
| NM_002651 | PIK4CB | -0.56 | NM_000388 | CASR | -0.56 |
| NM_020149 | MEIS2 | -0.56 | NM_005326 | HAGH | -0.56 |
| NM_000190 | HMBS | -0.56 | NM_005622 | SAH | -0.56 |
| NM_020383 | XPNPEP1 | -0.56 | NM_003895 | SYNJ1 | -0.56 |
| NM_005339 | HIP2 | -0.56 | NM_022039 | SHFM3 | -0.56 |
| NM_020731 | AHRR | -0.56 | NM_004984 | KIF5A | -0.56 |
| NM_005704 | PTPRU | -0.56 | NM_000743 | CHRNA3 | -0.56 |
| NM_000042 | APOH | -0.56 | NM_015711 | GLTSCR1 | -0.56 |
| XM_376776 | TOX | -0.56 | NM_006596 | POLQ | -0.56 |
| NM_006369 | MUF1 | -0.56 | XM_051081 | TBC1D12 | -0.56 |
| NM_015448 | DPCD | -0.56 | NM_016491 | MRPL37 | -0.56 |
| NM_013382 | POMT2 | -0.56 | NM_016619 | PLAC8 | -0.56 |
| NM_017645 | FAM29A | -0.56 | NM_018152 | C20ORF12 | -0.56 |
| NM_017671 | C20ORF42 | -0.56 | NM_018257 | C20ORF36 | -0.56 |
| NM_018715 | TD-60 | -0.56 | NM_019894 | TMPRSS4 | -0.56 |
| NM_020653 | ZNF287 | -0.56 | NM_020813 | ZNF471 | -0.56 |
| NM_033052 | DMRTC2 | -0.56 | NM_024099 | MGC2477 | -0.56 |
| NM_031307 | FKSG32 | -0.56 | NM_030962 | CMT4B2 | -0.56 |
| NM_032920 | C21ORF124 | -0.56 | NM_138569 | C6ORF142 | -0.56 |
| NM_144586 | MGC29643 | -0.56 | NM_139162 | SMCR7 | -0.56 |
| NM_080674 | C20ORF86 | -0.56 | XM_379636 | C9ORF122 | -0.56 |
| NM_152778 | MGC33302 | -0.56 | XM_375099 | LOC283585 | -0.56 |
| NM_173628 | FLJ40457 | -0.56 | NM_181647 | LOC285398 | -0.56 |
| XM_377701 | LOC284861 | -0.56 | NM_198478 | NKPD1 | -0.56 |
| XM_371085 | LOC339229 | -0.56 | NM_182596 | FLJ25037 | -0.56 |
| NM_001001479 | SLC35E4 | -0.56 | NM_181684 | KRTAP12-2 | -0.56 |
| XM_066069 | LOC128629 | -0.56 | XM_295126 | LOC339997 | -0.56 |
| XM_208373 | LOC285984 | -0.56 | XM_372760 | LOC390999 | -0.56 |
| XM_374057 | LOC389161 | -0.56 | XM_379552 | LOC401449 | -0.56 |
| XM_379634 | LOC401504 | -0.56 | XM_380099 | LOC402476 | -0.56 |
| XM_378052 | LOC402357 | -0.56 | XM_371081 | LOC388424 | -0.56 |
| XM_372214 | LOC389857 | -0.56 | NM_006067 | NOC4 | -0.56 |
| NM_152700 | MGC26597 | -0.55 | NM_002030 | FPRL2 | -0.55 |
| NM_000733 | CD3E | -0.55 | NM_003906 | MCM3AP | -0.55 |
| NM_002628 | PFN2 | -0.55 | NM_016089 | ZNF589 | -0.55 |
| NM_015994 | ATP6V1D | -0.55 | NM_013366 | ANAPC2 | -0.55 |

| | | | | | |
|---|---|---|---|---|---|
| NM_021223 | MYL7 | -0.55 | NM_018223 | CHFR | -0.55 |
| NM_003591 | CUL2 | -0.55 | NM_004244 | CD163 | -0.55 |
| NM_002207 | ITGA9 | -0.55 | NM_003808 | TNFSF13 | -0.55 |
| NM_002276 | KRT19 | -0.55 | NM_015719 | COL5A3 | -0.55 |
| NM_005639 | SYT1 | -0.55 | NM_001638 | APOF | -0.55 |
| NM_001805 | CEBPE | -0.55 | NM_005141 | FGB | -0.55 |
| NM_016946 | F11R | -0.55 | NM_000766 | CYP2A13 | -0.55 |
| NM_004955 | SLC29A1 | -0.55 | NM_006501 | MOBP | -0.55 |
| NM_152707 | SLC25A16 | -0.55 | NM_014738 | KIAA0195 | -0.55 |
| XM_041363 | PDZRN3 | -0.55 | XM_114303 | GRSP1 | -0.55 |
| NM_016466 | MGC41816 | -0.55 | NM_016630 | SPG21 | -0.55 |
| NM_019066 | MAGEL2 | -0.55 | NM_018990 | CXORF9 | -0.55 |
| NM_020319 | ANKMY2 | -0.55 | NM_033520 | C19ORF33 | -0.55 |
| NM_024612 | DHX40 | -0.55 | NM_138778 | C9ORF112 | -0.55 |
| NM_144594 | FLJ32942 | -0.55 | NM_138798 | LOC129531 | -0.55 |
| NM_139170 | LOC146562 | -0.55 | NM_173566 | MGC50372 | -0.55 |
| NM_175906 | MGC33608 | -0.55 | XM_291419 | GPR153 | -0.55 |
| NM_177987 | TUBB8-PENDING | -0.55 | NM_199244 | FOXD4B | -0.55 |
| XM_113912 | LOC201140 | -0.55 | XM_373866 | LOC388692 | -0.55 |
| XM_379068 | LOC400937 | -0.55 | XM_374052 | LOC389150 | -0.55 |
| XM_371818 | LOC389386 | -0.55 | XM_374142 | LOC389333 | -0.55 |
| XM_379657 | LOC401544 | -0.55 | XM_370577 | LOC387712 | -0.55 |
| XM_378331 | LOC399986 | -0.55 | XM_373630 | LOC388126 | -0.55 |
| XM_370691 | LOC387856 | -0.55 | XM_377076 | LOC401625 | -0.55 |
| NM_013410 | AK3 | -0.54 | NM_014216 | ITPK1 | -0.54 |
| NM_031417 | MARK4 | -0.54 | NM_002591 | PCK1 | -0.54 |
| NM_021643 | TRIB2 | -0.54 | NM_000633 | BCL2 | -0.54 |
| NM_005618 | DLL1 | -0.54 | NM_139058 | ARX | -0.54 |
| NM_005390 | PDHA2 | -0.54 | NM_003129 | SQLE | -0.54 |
| NM_000478 | ALPL | -0.54 | NM_018486 | HDAC8 | -0.54 |
| NM_001375 | DNASE2 | -0.54 | NM_006195 | PBX3 | -0.54 |
| NM_000878 | IL2RB | -0.54 | NM_003611 | OFD1 | -0.54 |
| NM_001041 | SI | -0.54 | NM_004541 | NDUFA1 | -0.54 |
| NM_006940 | SOX5 | -0.54 | NM_003916 | AP1S2 | -0.54 |
| NM_005686 | SOX13 | -0.54 | NM_004728 | DDX21 | -0.54 |
| NM_005726 | TSFM | -0.54 | NM_006328 | RBM14 | -0.54 |
| NM_007213 | PRAF2 | -0.54 | NM_014010 | ASTN2 | -0.54 |
| NM_015117 | ZC3HDC3 | -0.54 | XM_047610 | KIAA1086 | -0.54 |
| NM_013403 | STRN4 | -0.54 | NM_032472 | PPIL3 | -0.54 |
| NM_018282 | PSPC1 | -0.54 | NM_018296 | FLJ11004 | -0.54 |
| NM_031271 | TEX15 | -0.54 | NM_020124 | IFNK | -0.54 |
| NM_022747 | C14ORF136 | -0.54 | NM_024875 | SYNPO2L | -0.54 |
| NM_030792 | PP1665 | -0.54 | NM_080593 | HIST1H2BK | -0.54 |
| NM_033188 | KRTAP4-5 | -0.54 | NM_145241 | WDR31 | -0.54 |
| NM_176813 | BCMP11 | -0.54 | NM_152589 | FLJ35821 | -0.54 |
| NM_152754 | SEMA3D | -0.54 | XM_378525 | LOC283731 | -0.54 |

| | | |
|---|---|---|
| NM_198993 | STAC2 | -0.54 |
| XM_114158 | LOC200226 | -0.54 |
| XM_373449 | LOC387638 | -0.54 |
| NM_001005493 | OR6C6 | -0.54 |
| NM_005465 | AKT3 | -0.53 |
| NM_182493 | LOC91807 | -0.53 |
| NM_000309 | PPOX | -0.53 |
| NM_024022 | TMPRSS3 | -0.53 |
| NM_005690 | DNM1L | -0.53 |
| NM_005018 | PDCD1 | -0.53 |
| NM_030761 | WNT4 | -0.53 |
| NM_145640 | APOL3 | -0.53 |
| NM_005488 | TOM1 | -0.53 |
| NM_003768 | PEA15 | -0.53 |
| NM_030916 | PVRL4 | -0.53 |
| NM_002763 | PROX1 | -0.53 |
| NM_000716 | C4BPB | -0.53 |
| NM_003092 | SNRPB2 | -0.53 |
| NM_004823 | KCNK6 | -0.53 |
| NM_014733 | ZFYVE16 | -0.53 |
| NM_019044 | FLJ10996 | -0.53 |
| NM_017985 | C9ORF68 | -0.53 |
| NM_018288 | PHF10 | -0.53 |
| NM_020246 | SLC12A9 | -0.53 |
| NM_020314 | MGC16824 | -0.53 |
| NM_033440 | ELA2A | -0.53 |
| NM_024884 | C14ORF160 | -0.53 |
| NM_025217 | ULBP2 | -0.53 |
| NM_030915 | LBH | -0.53 |
| NM_032869 | CML66 | -0.53 |
| NM_052880 | MGC17330 | -0.53 |
| NM_144608 | FLJ32384 | -0.53 |
| XM_376535 | C6orf190 | -0.53 |
| NM_080675 | SPAG4L | -0.53 |
| NM_152594 | SPRED1 | -0.53 |
| XM_293312 | LOC347376 | -0.53 |
| XM_293360 | LOC340571 | -0.53 |
| XM_377946 | LOC402280 | -0.53 |
| XM_379958 | LOC402619 | -0.53 |
| XM_374767 | LOC399716 | -0.53 |
| NM_001348 | DAPK3 | -0.52 |
| NM_016308 | UMP-CMPK | -0.52 |
| NM_178168 | OR10A5 | -0.52 |
| NM_018429 | BDP1 | -0.52 |
| NM_002128 | HMGB1 | -0.52 |
| NM_005224 | ARID3A | -0.52 |
| XM_064883 | LOC125962 | -0.54 |
| XM_371592 | LOC389073 | -0.54 |
| NM_001001679 | FLJ41423 | -0.54 |
| NM_000680 | ADRA1A | -0.53 |
| NM_002596 | PCTK3 | -0.53 |
| NM_004885 | GPR74 | -0.53 |
| NM_003475 | C11ORF13 | -0.53 |
| NM_000787 | DBH | -0.53 |
| NM_003972 | BTAF1 | -0.53 |
| NM_019618 | IL1F9 | -0.53 |
| NM_000407 | GP1BB | -0.53 |
| NM_003297 | NR2C1 | -0.53 |
| NM_001641 | APEX1 | -0.53 |
| NM_006769 | LMO4 | -0.53 |
| NM_006832 | PLEKHC1 | -0.53 |
| NM_005112 | WDR1 | -0.53 |
| NM_004395 | DBN1 | -0.53 |
| NM_007153 | ZNF208 | -0.53 |
| XM_375770 | LRIG2 | -0.53 |
| NM_006042 | HS3ST3A1 | -0.53 |
| NM_019002 | ETAA16 | -0.53 |
| NM_018363 | C10ORF59 | -0.53 |
| NM_018414 | SIAT7A | -0.53 |
| NM_020466 | DJ122O8.2 | -0.53 |
| XM_371184 | KIAA1183 | -0.53 |
| NM_024580 | FLJ13119 | -0.53 |
| NM_025059 | C6ORF97 | -0.53 |
| NM_031300 | MXD3 | -0.53 |
| NM_032813 | FLJ14624 | -0.53 |
| NM_145239 | LOC112476 | -0.53 |
| NM_053282 | EAT2 | -0.53 |
| NM_152375 | FLJ38753 | -0.53 |
| NM_152906 | DKFZP761P1121 | -0.53 |
| NM_152542 | DKFZP761G058 | -0.53 |
| NM_145657 | GSH1 | -0.53 |
| XM_096376 | LOC143034 | -0.53 |
| XM_379163 | LOC401038 | -0.53 |
| XM_376386 | LOC401190 | -0.53 |
| XM_378305 | LOC399933 | -0.53 |
| NM_001005285 | OR2AT4 | -0.53 |
| NM_004409 | DMPK | -0.52 |
| NM_018490 | GPR48 | -0.52 |
| NM_004147 | DRG1 | -0.52 |
| NM_006546 | IMP-1 | -0.52 |
| NM_003017 | SFRS3 | -0.52 |
| NM_007222 | ZHX1 | -0.52 |

| | | | | | |
|---|---|---|---|---|---|
| NM_014236 | GNPAT | -0.52 | NM_006745 | SC4MOL | -0.52 |
| NM_001873 | CPE | -0.52 | NM_005020 | PDE1C | -0.52 |
| NM_001493 | GDI1 | -0.52 | NM_005091 | PGLYRP1 | -0.52 |
| NM_002162 | ICAM3 | -0.52 | NM_004773 | TRIP3 | -0.52 |
| NM_003250 | THRA | -0.52 | NM_058242 | KRT6C | -0.52 |
| NM_014567 | BCAR1 | -0.52 | NM_057176 | BSND | -0.52 |
| NM_001923 | DDB1 | -0.52 | NM_003875 | GMPS | -0.52 |
| XM_084530 | KIAA0033 | -0.52 | NM_000426 | LAMA2 | -0.52 |
| NM_000904 | NQO2 | -0.52 | NM_002664 | PLEK | -0.52 |
| NM_002727 | PRG1 | -0.52 | NM_001729 | BTC | -0.52 |
| NM_001682 | ATP2B1 | -0.52 | NM_021949 | ATP2B3 | -0.52 |
| NM_002065 | GLUL | -0.52 | NM_005849 | IGSF6 | -0.52 |
| NM_014453 | BC-2 | -0.52 | NM_016053 | CGI-116 | -0.52 |
| NM_016067 | MRPS18C | -0.52 | NM_018999 | KIAA1128 | -0.52 |
| NM_022099 | C20ORF51 | -0.52 | NM_022777 | RABL5 | -0.52 |
| NM_024296 | MGC1203 | -0.52 | NM_177477 | LYNX1 | -0.52 |
| NM_024809 | FLJ12975 | -0.52 | NM_032372 | MGC16186 | -0.52 |
| NM_052943 | MGC16491 | -0.52 | NM_181441 | MRPL55 | -0.52 |
| NM_080833 | C20ORF151 | -0.52 | NM_080574 | C20ORF70 | -0.52 |
| NM_144693 | ZNF558 | -0.52 | NM_152533 | MGC34728 | -0.52 |
| NM_175853 | LOC150759 | -0.52 | XM_117294 | LOC200933 | -0.52 |
| NM_173084 | TRIM59 | -0.52 | XM_094066 | LOC152877 | -0.52 |
| XM_208200 | LOC284387 | -0.52 | XM_372995 | LOC391553 | -0.52 |
| XM_372802 | LOC391123 | -0.52 | XM_371754 | LOC389301 | -0.52 |
| XM_376607 | LOC401335 | -0.52 | XM_376716 | LOC401429 | -0.52 |
| XM_375305 | LOC400506 | -0.52 | XM_373927 | LOC388815 | -0.52 |
| NM_001005495 | OR2T3 | -0.52 | NM_001896 | CSNK2A2 | -0.51 |
| NM_005565 | LCP2 | -0.51 | NM_002041 | GABPB2 | -0.51 |
| NM_002286 | LAG3 | -0.51 | NM_006498 | LGALS2 | -0.51 |
| NM_000059 | BRCA2 | -0.51 | NM_016135 | ETV7 | -0.51 |
| NM_130465 | FBXO23 | -0.51 | NM_021138 | TRAF2 | -0.51 |
| NM_007155 | ZP3 | -0.51 | NM_021005 | NR2F2 | -0.51 |
| NM_020632 | ATP6V0A4 | -0.51 | NM_004830 | CRSP3 | -0.51 |
| NM_001737 | C9 | -0.51 | NM_014694 | KIAA0605 | -0.51 |
| NM_014857 | HHL | -0.51 | NM_002271 | KPNB3 | -0.51 |
| NM_000337 | SGCD | -0.51 | NM_173702 | MTATP6 | -0.51 |
| NM_207517 | ADAMTSL3 | -0.51 | NM_015315 | LARP | -0.51 |
| NM_002568 | PABPC1 | -0.51 | NM_013306 | SNX15 | -0.51 |
| NM_016319 | COPS7A | -0.51 | NM_016332 | SEPX1 | -0.51 |
| NM_018061 | FLJ10330 | -0.51 | NM_018133 | FLJ10546 | -0.51 |
| NM_020971 | SPTBN4 | -0.51 | NM_024769 | ASAM | -0.51 |
| XM_046437 | C20ORF50 | -0.51 | NM_030930 | UNC93B1 | -0.51 |
| NM_032036 | FAM14A | -0.51 | NM_032126 | DKFZP564J047 | -0.51 |
| NM_032317 | WBSCR18 | -0.51 | NM_032649 | CNDP1 | -0.51 |
| NM_033211 | LOC90355 | -0.51 | NM_025077 | TOE1 | -0.51 |
| XM_040910 | C14ORF73 | -0.51 | NM_177963 | SYT12 | -0.51 |

| | | | | | |
|---|---|---|---|---|---|
| XM_372882 | LOC128954 | -0.51 | NM_080872 | UNC5D | -0.51 |
| NM_194319 | ZNF542 | -0.51 | XM_376821 | C9ORF14 | -0.51 |
| XM_376874 | C9ORF71 | -0.51 | XM_170842 | FLJ40244 | -0.51 |
| NM_173590 | FLJ36102 | -0.51 | NM_198489 | DLNB14 | -0.51 |
| NM_194312 | LOC339768 | -0.51 | NM_175924 | MGC50831 | -0.51 |
| NM_198181 | LOC374676 | -0.51 | XM_098164 | LOC152122 | -0.51 |
| XM_294093 | LOC346171 | -0.51 | XM_069621 | LOC135946 | -0.51 |
| XM_294070 | LOC346085 | -0.51 | XM_298045 | LOC347475 | -0.51 |
| XM_379094 | LOC400953 | -0.51 | XM_371481 | LOC388931 | -0.51 |
| XM_379587 | LOC401464 | -0.51 | XM_378389 | LOC400084 | -0.51 |
| NM_001004329 | FLJ16139 | -0.51 | NM_004567 | PFKFB4 | -0.5 |
| NM_001390 | DTNA | -0.5 | NM_006732 | FOSB | -0.5 |
| NM_012484 | HMMR | -0.5 | NM_003413 | ZIC3 | -0.5 |
| NM_016265 | ZNF325 | -0.5 | NM_021020 | LZTS1 | -0.5 |
| NM_003121 | SPIB | -0.5 | NM_020299 | AKR1B10 | -0.5 |
| NM_001748 | CAPN2 | -0.5 | NM_002392 | MDM2 | -0.5 |
| NM_015376 | RASGRP3 | -0.5 | NM_002667 | PLN | -0.5 |
| NM_005608 | PTPRCAP | -0.5 | NM_014203 | AP2A1 | -0.5 |
| NM_005205 | COX6A2 | -0.5 | NM_022914 | 24432 | -0.5 |
| NM_007135 | ZNF79 | -0.5 | NM_003435 | ZNF134 | -0.5 |
| NM_005416 | SPRR3 | -0.5 | NM_004636 | SEMA3B | -0.5 |
| NM_006688 | C1QL1 | -0.5 | NM_007266 | XAB1 | -0.5 |
| NM_152260 | C18B11 | -0.5 | NM_015931 | LOC51066 | -0.5 |
| NM_017445 | H2BFS | -0.5 | NM_024662 | FLJ10774 | -0.5 |
| XM_087386 | HEG | -0.5 | NM_022347 | IFRG15 | -0.5 |
| NM_023070 | LOC65243 | -0.5 | NM_030968 | C1QTNF1 | -0.5 |
| NM_080863 | ASB16 | -0.5 | NM_171998 | RAB39B | -0.5 |
| NM_052968 | APOA5 | -0.5 | NM_152387 | FLJ31322 | -0.5 |
| XM_066058 | C20ORF174 | -0.5 | NM_152441 | FBXL14 | -0.5 |
| XM_371225 | LOC284729 | -0.5 | NM_207493 | FLJ44896 | -0.5 |
| XM_295598 | LOC343484 | -0.5 | XM_059578 | LOC132203 | -0.5 |
| XM_211251 | LOC283902 | -0.5 | XM_293160 | LOC343702 | -0.5 |
| XM_291663 | LOC340654 | -0.5 | XM_291270 | LOC340393 | -0.5 |
| XM_376819 | LOC401494 | -0.5 | XM_373487 | LOC387742 | -0.5 |
| XM_370876 | LOC388137 | -0.5 | XM_370829 | LOC388069 | -0.5 |
| XM_378793 | LOC400684 | -0.5 | NM_001005329 | OR51A4 | -0.5 |
| NM_152696 | HIPK1 | -0.49 | NM_006051 | APBB3 | -0.49 |
| XM_093895 | KIAA0882 | -0.49 | NM_003005 | SELP | -0.49 |
| NM_000903 | NQO1 | -0.49 | NM_005738 | ARL4A | -0.49 |
| NM_007082 | RABL2A | -0.49 | NM_015071 | ARHGAP26 | -0.49 |
| NM_012162 | FBXL6 | -0.49 | NM_033223 | GABRG3 | -0.49 |
| NM_001761 | CCNF | -0.49 | NM_020348 | CNNM1 | -0.49 |
| NM_005748 | YAF2 | -0.49 | NM_015160 | PMPCA | -0.49 |
| NM_001500 | GMDS | -0.49 | NM_018672 | ABCA5 | -0.49 |
| NM_003692 | TMEFF1 | -0.49 | XM_375660 | ZNF264 | -0.49 |
| NM_005835 | SLC17A2 | -0.49 | NM_014802 | KIAA0528 | -0.49 |

| | | | | | |
|---|---|---|---|---|---|
| NM_015055 | SWAP70 | -0.49 | NM_015653 | C22ORF11 | -0.49 |
| NM_016029 | DHRS7 | -0.49 | NM_017815 | C14ORF94 | -0.49 |
| NM_018405 | HSA272196 | -0.49 | NM_018275 | FLJ10925 | -0.49 |
| NM_022055 | KCNK12 | -0.49 | NM_024857 | FLJ12735 | -0.49 |
| NM_145169 | C6ORF83 | -0.49 | NM_152346 | MGC34680 | -0.49 |
| NM_145807 | LOC126147 | -0.49 | NM_133262 | ATP6V1G3 | -0.49 |
| NM_153011 | FLJ30594 | -0.49 | NM_145279 | MOBKL2C | -0.49 |
| NM_153029 | FLJ31821 | -0.49 | NM_173801 | FLJ36198 | -0.49 |
| NM_173807 | MGC33370 | -0.49 | NM_173568 | FLJ36335 | -0.49 |
| XM_378886 | LOC284475 | -0.49 | XM_033704 | DKFZP434C184 | -0.49 |
| XM_208213 | LOC284541 | -0.49 | XM_208313 | LOC285321 | -0.49 |
| XM_376618 | LOC401356 | -0.49 | XM_376814 | LOC401487 | -0.49 |
| XM_374803 | LOC399763 | -0.49 | XM_378273 | LOC399867 | -0.49 |
| XM_379006 | LOC400865 | -0.49 | NM_001005286 | OR6F1 | -0.49 |
| NM_006343 | MERTK | -0.48 | NM_173598 | KSR2 | -0.48 |
| NM_178564 | LOC340371 | -0.48 | NM_005282 | GPR4 | -0.48 |
| NM_023922 | TAS2R14 | -0.48 | NM_014391 | ANKRD1 | -0.48 |
| NM_174980 | VN1R3 | -0.48 | NM_005029 | PITX3 | -0.48 |
| NM_003204 | NFE2L1 | -0.48 | NM_003739 | AKR1C3 | -0.48 |
| NM_000120 | EPHX1 | -0.48 | NM_005440 | ARHN | -0.48 |
| NM_018727 | TRPV1 | -0.48 | NM_002840 | PTPRF | -0.48 |
| NM_000526 | KRT14 | -0.48 | NM_014078 | MRPL13 | -0.48 |
| NM_030582 | COL18A1 | -0.48 | NM_005513 | GTF2E1 | -0.48 |
| NM_006996 | SLC19A2 | -0.48 | NM_001046 | SLC12A2 | -0.48 |
| NM_006997 | TACC2 | -0.48 | NM_001636 | SLC25A6 | -0.48 |
| NM_001740 | CALB2 | -0.48 | NM_001476 | GAGE6 | -0.48 |
| NM_175617 | MT1E | -0.48 | NM_030673 | SEC13L1 | -0.48 |
| NM_003094 | SNRPE | -0.48 | NM_021018 | HIST1H3F | -0.48 |
| NM_014731 | PROSAPIP1 | -0.48 | NM_014608 | CYFIP1 | -0.48 |
| XM_371179 | FBXO46 | -0.48 | NM_014392 | D4S234E | -0.48 |
| NM_017424 | CECR1 | -0.48 | NM_017600 | DKFZP434M0331 | -0.48 |
| NM_018279 | TMEM19 | -0.48 | NM_020232 | TNFSF5IP1 | -0.48 |
| NM_021218 | C9ORF80 | -0.48 | XM_048128 | KIAA1596 | -0.48 |
| XM_371848 | C6ORF115 | -0.48 | NM_021928 | FLJ22649 | -0.48 |
| NM_021630 | PDLIM2 | -0.48 | NM_022137 | SMOC1 | -0.48 |
| NM_024687 | FLJ23049 | -0.48 | NM_025058 | TRIM46 | -0.48 |
| NM_033017 | TRIM4 | -0.48 | NM_138381 | MGC15763 | -0.48 |
| NM_152341 | FLJ30002 | -0.48 | NM_144625 | FLJ32978 | -0.48 |
| NM_178819 | DKFZP586M1819 | -0.48 | NM_152463 | EME1 | -0.48 |
| NM_080873 | ASB11 | -0.48 | NM_130900 | RAET1L | -0.48 |
| NM_172005 | WFDC13 | -0.48 | NM_020865 | DHX36 | -0.48 |
| NM_145022 | C10ORF25 | -0.48 | NM_153267 | MAMDC2 | -0.48 |
| NM_199227 | MAP1D | -0.48 | XM_379069 | LOC339822 | -0.48 |
| NM_182610 | SAMD7 | -0.48 | XM_209695 | LOC285643 | -0.48 |
| XM_209423 | LOC284964 | -0.48 | XM_209554 | LOC285299 | -0.48 |
| XM_378852 | LOC400748 | -0.48 | XM_374103 | LOC389252 | -0.48 |

| | | | | | |
|---|---|---|---|---|---|
| XM_379905 | LOC402576 | -0.48 | XM_374589 | LOC392848 | -0.48 |
| XM_375378 | LOC400549 | -0.48 | XM_372876 | LOC391269 | -0.48 |
| NM_001001888 | VCX-C | -0.48 | NM_016231 | NLK | -0.47 |
| NM_005163 | AKT1 | -0.47 | NM_005242 | F2RL1 | -0.47 |
| NM_001407 | CELSR3 | -0.47 | NM_001747 | CAPG | -0.47 |
| NM_004216 | DEDD | -0.47 | NM_003675 | PRPF18 | -0.47 |
| NM_018935 | PCDHB15 | -0.47 | NM_002961 | S100A4 | -0.47 |
| NM_000693 | ALDH1A3 | -0.47 | NM_002130 | HMGCS1 | -0.47 |
| NM_003761 | VAMP8 | -0.47 | NM_000536 | RAG2 | -0.47 |
| NM_017414 | USP18 | -0.47 | NM_020774 | MIB1 | -0.47 |
| NM_002182 | IL1RAP | -0.47 | NM_002634 | PHB | -0.47 |
| NM_005183 | CACNA1F | -0.47 | NM_144585 | SLC22A12 | -0.47 |
| NM_030932 | DIAPH3 | -0.47 | NM_000562 | C8A | -0.47 |
| NM_004403 | DFNA5 | -0.47 | NM_001322 | CST2 | -0.47 |
| NM_144646 | IGJ | -0.47 | NM_000786 | CYP51A1 | -0.47 |
| NM_004153 | ORC1L | -0.47 | NM_004549 | NDUFC2 | -0.47 |
| NM_007147 | ZNF175 | -0.47 | NM_003492 | CXORF12 | -0.47 |
| NM_006987 | RPH3AL | -0.47 | NM_005500 | SAE1 | -0.47 |
| NM_014767 | SPOCK2 | -0.47 | NM_015694 | KIAA1285 | -0.47 |
| NM_017819 | RG9MTD1 | -0.47 | NM_018509 | PRO1855 | -0.47 |
| NM_018453 | C14ORF11 | -0.47 | NM_019117 | KLHL4 | -0.47 |
| XM_371084 | KIAA1447 | -0.47 | XM_291015 | KIDINS220 | -0.47 |
| NM_021815 | SLC5A7 | -0.47 | NM_021186 | ZP4 | -0.47 |
| NM_020875 | FRAS1 | -0.47 | NM_032907 | BMSC-UBP | -0.47 |
| NM_138446 | C7ORF30 | -0.47 | NM_173473 | C10ORF104 | -0.47 |
| NM_153634 | CPNE8 | -0.47 | NM_020828 | ZFP28 | -0.47 |
| NM_144710 | 40422 | -0.47 | NM_203406 | LOC153364 | -0.47 |
| NM_175884 | FLJ36031 | -0.47 | NM_178842 | LASS3 | -0.47 |
| NM_173559 | FLJ25791 | -0.47 | NM_173646 | FLJ39660 | -0.47 |
| XM_292819 | NANOS3 | -0.47 | NM_183059 | C1ORF36 | -0.47 |
| NM_182605 | FLJ40448 | -0.47 | NM_205841 | MGC21394 | -0.47 |
| XM_211816 | LOC285248 | -0.47 | XM_295007 | LOC339583 | -0.47 |
| XM_209076 | LOC284232 | -0.47 | XM_071013 | LOC138652 | -0.47 |
| XM_379408 | LOC401255 | -0.47 | XM_372445 | LOC390278 | -0.47 |
| XM_371165 | LOC388538 | -0.47 | NM_004274 | AKAP6 | -0.46 |
| NM_032571 | EMR3 | -0.46 | NM_004072 | CMKLR1 | -0.46 |
| NM_012202 | GNG3 | -0.46 | NM_002649 | PIK3CG | -0.46 |
| NM_033282 | OPN4 | -0.46 | NM_022111 | CLSPN | -0.46 |
| NM_001731 | BTG1 | -0.46 | NM_032584 | ZNF347 | -0.46 |
| NM_018419 | SOX18 | -0.46 | NM_004176 | SREBF1 | -0.46 |
| NM_005518 | HMGCS2 | -0.46 | NM_002421 | MMP1 | -0.46 |
| NM_004082 | DCTN1 | -0.46 | NM_002847 | PTPRN2 | -0.46 |
| NM_003743 | NCOA1 | -0.46 | NM_000335 | SCN5A | -0.46 |
| NM_031885 | BBS2 | -0.46 | NM_019084 | CCNJ | -0.46 |
| NM_005542 | INSIG1 | -0.46 | NM_005705 | PHEMX | -0.46 |
| NM_145208 | MBD3L1 | -0.46 | NM_052877 | MED8 | -0.46 |

| | | | | | |
|---|---|---|---|---|---|
| NM_014109 | ATAD2 | -0.46 | NM_004059 | CCBL1 | -0.46 |
| NM_001998 | FBLN2 | -0.46 | NM_004084 | DEFA1 | -0.46 |
| XM_031401 | EGFL3 | -0.46 | NM_005560 | LAMA5 | -0.46 |
| NM_003501 | ACOX3 | -0.46 | NM_002643 | PIGF | -0.46 |
| NM_031246 | PSG2 | -0.46 | NM_003291 | TPP2 | -0.46 |
| NM_005638 | SYBL1 | -0.46 | NM_014519 | ZNF232 | -0.46 |
| NM_004660 | DDX3Y | -0.46 | NM_006590 | USP39 | -0.46 |
| NM_006387 | CHERP | -0.46 | NM_006405 | TM9SF1 | -0.46 |
| NM_006780 | SMA3 | -0.46 | NM_014893 | NLGN4Y | -0.46 |
| NM_014934 | DZIP1 | -0.46 | NM_012342 | BAMBI | -0.46 |
| NM_013252 | CLECSF5 | -0.46 | NM_019598 | KLK12 | -0.46 |
| NM_018105 | THAP1 | -0.46 | NM_020231 | MDS010 | -0.46 |
| NM_152233 | SNX6 | -0.46 | NM_024041 | SCNM1 | -0.46 |
| NM_024834 | C10ORF119 | -0.46 | NM_024899 | C18ORF9 | -0.46 |
| NM_052959 | PANX3 | -0.46 | NM_152509 | FLJ31568 | -0.46 |
| NM_144984 | C10ORF72 | -0.46 | NM_153036 | C6ORF78 | -0.46 |
| XM_379099 | LOC339807 | -0.46 | NM_020063 | BARHL2 | -0.46 |
| XM_372110 | LOC375719 | -0.46 | XM_085236 | LOC145788 | -0.46 |
| XM_065278 | LOC129521 | -0.46 | XM_292784 | LOC339351 | -0.46 |
| XM_374460 | LOC155081 | -0.46 | XM_371796 | LOC389364 | -0.46 |
| XM_379848 | LOC402521 | -0.46 | XM_373524 | LOC387833 | -0.46 |
| NM_000682 | ADRA2B | -0.45 | NM_018485 | GPR77 | -0.45 |
| NM_016235 | GPRC5B | -0.45 | NM_000513 | OPN1MW | -0.45 |
| NM_005292 | GPR18 | -0.45 | NM_172027 | ABTB1 | -0.45 |
| NM_176810 | NALP13 | -0.45 | NM_019105 | TNXB | -0.45 |
| NM_021240 | DMRT3 | -0.45 | NM_001154 | ANXA5 | -0.45 |
| NM_002655 | PLAG1 | -0.45 | NM_002076 | GNS | -0.45 |
| NM_003295 | TPT1 | -0.45 | NM_004347 | CASP5 | -0.45 |
| NM_013438 | UBQLN1 | -0.45 | NM_000762 | CYP2A6 | -0.45 |
| XM_040265 | KIAA0217 | -0.45 | NM_013367 | ANAPC4 | -0.45 |
| NM_003637 | ITGA10 | -0.45 | NM_001119 | ADD1 | -0.45 |
| NM_000387 | SLC25A20 | -0.45 | NM_006316 | MYCNOS | -0.45 |
| NM_153207 | AEBP2 | -0.45 | NM_003284 | TNP1 | -0.45 |
| XM_375024 | UTP14C | -0.45 | NM_181581 | PP35 | -0.45 |
| NM_014553 | TFCP2L1 | -0.45 | NM_017983 | FLJ10055 | -0.45 |
| NM_018713 | ZNT8 | -0.45 | NM_018475 | TPARL | -0.45 |
| NM_152278 | MGC23947 | -0.45 | NM_022661 | SPANXC | -0.45 |
| NM_024044 | MGC5178 | -0.45 | NM_024519 | FLJ13725 | -0.45 |
| NM_025083 | FLJ21128 | -0.45 | NM_031291 | DKFZP434N1235 | -0.45 |
| NM_032029 | FKSG87 | -0.45 | NM_032307 | C9ORF64 | -0.45 |
| NM_032592 | PHACS | -0.45 | NM_033212 | MGC10992 | -0.45 |
| NM_053052 | IMAGE3451454 | -0.45 | NM_145249 | FAM14B | -0.45 |
| NM_178466 | C20ORF71 | -0.45 | NM_153215 | FLJ38608 | -0.45 |
| NM_138797 | LOC129138 | -0.45 | NM_130898 | CREB3L4 | -0.45 |
| NM_145174 | DNAJB7 | -0.45 | NM_144721 | THAP6 | -0.45 |
| NM_182558 | FLJ33810 | -0.45 | XM_171224 | CARM1L | -0.45 |

| | | | | | |
|---|---|---|---|---|---|
| NM_207357 | LOC339524 | -0.45 | XM_096676 | LOC144762 | -0.45 |
| XM_373977 | LOC388934 | -0.45 | XM_373101 | LOC391849 | -0.45 |
| XM_374136 | LOC389325 | -0.45 | XM_379904 | LOC402575 | -0.45 |
| XM_379803 | LOC402477 | -0.45 | XM_373588 | LOC387983 | -0.45 |
| XM_370958 | LOC388237 | -0.45 | XM_373351 | LOC392473 | -0.45 |
| NM_001005469 | OR5B3 | -0.45 | NM_025224 | BTBD4 | -0.45 |
| NM_005261 | GEM | -0.44 | NM_018900 | PCDHA1 | -0.44 |
| NM_003469 | SCG2 | -0.44 | NM_019558 | HOXD8 | -0.44 |
| NM_000310 | PPT1 | -0.44 | NM_006266 | RALGDS | -0.44 |
| NM_015382 | HECTD1 | -0.44 | NM_000873 | ICAM2 | -0.44 |
| NM_001181 | ASGR2 | -0.44 | NM_006533 | MIA | -0.44 |
| NM_001432 | EREG | -0.44 | NM_005245 | FAT | -0.44 |
| NM_005031 | FXYD1 | -0.44 | NM_003657 | BCAS1 | -0.44 |
| NM_003801 | GPAA1 | -0.44 | NM_025092 | FLJ22635 | -0.44 |
| NM_016250 | NDRG2 | -0.44 | NM_139247 | ADCY4 | -0.44 |
| NM_003211 | TDG | -0.44 | NM_006992 | B7 | -0.44 |
| NM_006774 | INMT | -0.44 | NM_014912 | CPEB3 | -0.44 |
| XM_371542 | RW1 | -0.44 | NM_014357 | LCE2B | -0.44 |
| NM_016496 | LOC51257 | -0.44 | NM_015916 | FAM26B | -0.44 |
| NM_016391 | HSPC111 | -0.44 | NM_018053 | FLJ10307 | -0.44 |
| NM_020336 | KIAA1219 | -0.44 | NM_018689 | KIAA1199 | -0.44 |
| XM_034717 | KIAA0493 | -0.44 | XM_290941 | PRNPIP | -0.44 |
| NM_024539 | RNF128 | -0.44 | NM_024873 | TNIP3 | -0.44 |
| NM_024900 | PHF17 | -0.44 | NM_032726 | PLCD4 | -0.44 |
| XM_290629 | C14ORF78 | -0.44 | NM_015163 | TRIM9 | -0.44 |
| NM_144634 | MGC26768 | -0.44 | NM_145651 | RYD5 | -0.44 |
| XM_376677 | LOC155006 | -0.44 | NM_144964 | RG9MTD3 | -0.44 |
| NM_153228 | FLJ38335 | -0.44 | XM_375646 | ZNF525 | -0.44 |
| XM_374915 | LOC283219 | -0.44 | NM_173573 | MGC35138 | -0.44 |
| XM_379691 | LOC284600 | -0.44 | XM_209655 | LOC285544 | -0.44 |
| XM_010658 | LOC159184 | -0.44 | XM_171165 | LOC253289 | -0.44 |
| XM_293334 | LOC340554 | -0.44 | XM_376560 | LOC401295 | -0.44 |
| XM_377941 | LOC402269 | -0.44 | XM_378449 | LOC400201 | -0.44 |
| NM_001001394 | HCG3 | -0.44 | XM_378103 | LOC402388 | -0.44 |
| XM_371466 | LOC388900 | -0.44 | NM_015716 | MINK | -0.43 |
| NM_030952 | SNARK | -0.43 | NM_018096 | FLJ10458 | -0.43 |
| NM_018841 | GNG12 | -0.43 | NM_014499 | P2RY10 | -0.43 |
| NM_003967 | PNR | -0.43 | NM_001157 | ANXA11 | -0.43 |
| NM_181711 | GRASP | -0.43 | NM_003075 | SMARCC2 | -0.43 |
| NM_005421 | TAL2 | -0.43 | NM_000306 | POU1F1 | -0.43 |
| NM_005354 | JUND | -0.43 | NM_001872 | CPB2 | -0.43 |
| NM_004744 | LRAT | -0.43 | NM_003348 | UBE2N | -0.43 |
| NM_000229 | LCAT | -0.43 | NM_007068 | DMC1 | -0.43 |
| NM_005223 | DNASE1 | -0.43 | NM_003460 | ZP2 | -0.43 |
| NM_005219 | DIAPH1 | -0.43 | NM_002468 | MYD88 | -0.43 |
| NM_014999 | RAB21 | -0.43 | NM_031296 | RAB33B | -0.43 |

| | | |
|---|---|---|
| NM_020247 | CABC1 | -0.43 |
| NM_004986 | KTN1 | -0.43 |
| NM_004764 | PIWIL1 | -0.43 |
| NM_001289 | CLIC2 | -0.43 |
| NM_006913 | RNF5 | -0.43 |
| NM_021998 | ZNF6 | -0.43 |
| NM_015136 | STAB1 | -0.43 |
| NM_014313 | SMP1 | -0.43 |
| NM_012385 | P8 | -0.43 |
| NM_016068 | TTC11 | -0.43 |
| NM_016462 | TMEM14C | -0.43 |
| NM_018381 | FLJ11286 | -0.43 |
| XM_106386 | KIAA1345 | -0.43 |
| NM_024306 | FA2H | -0.43 |
| NM_030580 | ZNF34 | -0.43 |
| XM_373675 | LOC90835 | -0.43 |
| XM_292778 | LRRC4B | -0.43 |
| XM_377720 | LOC150356 | -0.43 |
| NM_152578 | FMR1NB | -0.43 |
| NM_145316 | C6ORF128 | -0.43 |
| NM_207408 | FLJ27505 | -0.43 |
| XM_379318 | LOC401176 | -0.43 |
| NM_206899 | OR10P1 | -0.43 |
| NM_002649 | PIK3CG | -0.42 |
| NM_014341 | MTCH1 | -0.42 |
| NM_014176 | HSPC150 | -0.42 |
| NM_018177 | N4BP2 | -0.42 |
| NM_016186 | SERPINA10 | -0.42 |
| NM_021229 | NTN4 | -0.42 |
| NM_003780 | B4GALT2 | -0.42 |
| NM_014318 | 37347 | -0.42 |
| NM_018717 | MAML3 | -0.42 |
| NM_018347 | C20ORF29 | -0.42 |
| NM_024520 | FLJ22555 | -0.42 |
| NM_025146 | MAK3 | -0.42 |
| XM_376254 | DKFZP667G2110 | -0.42 |
| NM_152547 | BTNL9 | -0.42 |
| NM_152716 | FLJ36874 | -0.42 |
| XM_211529 | LOC284591 | -0.42 |
| NM_178544 | ZNF546 | -0.42 |
| XM_170950 | LOC254897 | -0.42 |
| XM_379853 | LOC402527 | -0.42 |
| XM_372563 | LOC390566 | -0.42 |
| XM_370909 | LOC388177 | -0.42 |
| NM_004105 | EFEMP1 | -0.41 |
| NM_014598 | SOCS7 | -0.41 |
| NM_017623 | CNNM3 | -0.43 |
| NM_005601 | NKG7 | -0.43 |
| NM_018343 | RIOK2 | -0.43 |
| NM_173713 | MTND5 | -0.43 |
| NM_002761 | PRM1 | -0.43 |
| NM_004872 | C1ORF8 | -0.43 |
| XM_039733 | KIAA0953 | -0.43 |
| NM_012427 | KLK5 | -0.43 |
| NM_015610 | DKFZP434J154 | -0.43 |
| NM_018968 | SNTG2 | -0.43 |
| NM_017654 | FLJ20073 | -0.43 |
| NM_020143 | LOC56902 | -0.43 |
| NM_022144 | TNMD | -0.43 |
| XM_376331 | KIAA1680 | -0.43 |
| NM_024893 | C20ORF39 | -0.43 |
| NM_152343 | FLJ25414 | -0.43 |
| XM_375695 | LOC126917 | -0.43 |
| NM_152531 | FLJ35155 | -0.43 |
| NM_138329 | NALP6 | -0.43 |
| XM_292260 | SLC35F4 | -0.43 |
| NM_207498 | FLJ43093 | -0.43 |
| XM_379897 | LOC402571 | -0.43 |
| NM_033115 | MGC16169 | -0.42 |
| NM_080817 | GPR82 | -0.42 |
| NM_006311 | NCOR1 | -0.42 |
| NM_012455 | TIC | -0.42 |
| NM_005940 | MMP11 | -0.42 |
| NM_000566 | FCGR1A | -0.42 |
| NM_003226 | TFF3 | -0.42 |
| NM_014568 | GALNT5 | -0.42 |
| NM_017714 | C20ORF13 | -0.42 |
| NM_018047 | RBM22 | -0.42 |
| NM_022917 | NOL6 | -0.42 |
| NM_024818 | UBE1DC1 | -0.42 |
| NM_022073 | EGLN3 | -0.42 |
| XM_085234 | UNC13C | -0.42 |
| NM_152628 | MGC39715 | -0.42 |
| NM_148675 | DSCR9 | -0.42 |
| NM_174947 | C19ORF30 | -0.42 |
| NM_198994 | TGM6 | -0.42 |
| XM_373290 | LOC392314 | -0.42 |
| XM_378202 | LOC399707 | -0.42 |
| XM_370904 | LOC388173 | -0.42 |
| XM_373750 | LOC388421 | -0.42 |
| NM_002417 | MKI67 | -0.41 |
| NM_005522 | HOXA1 | -0.41 |

| | | | | | |
|---|---|---|---|---|---|
| NM_000695 | ALDH3B2 | -0.41 | NM_002641 | PIGA | -0.41 |
| NM_016020 | TFB1M | -0.41 | NM_002900 | RBP3 | -0.41 |
| NM_000503 | EYA1 | -0.41 | NM_002335 | LRP5 | -0.41 |
| NM_002850 | PTPRS | -0.41 | NM_000729 | CCK | -0.41 |
| NM_004093 | EFNB2 | -0.41 | NM_005247 | FGF3 | -0.41 |
| NM_006530 | GAS41 | -0.41 | NM_006744 | RBP4 | -0.41 |
| NM_153700 | STRC | -0.41 | NM_003975 | SH2D2A | -0.41 |
| XM_371214 | PLCL4 | -0.41 | NM_006114 | TOMM40 | -0.41 |
| NM_015419 | DKFZP564I1922 | -0.41 | NM_017565 | FAM20A | -0.41 |
| NM_018240 | KIRREL | -0.41 | NM_018283 | NUDT15 | -0.41 |
| NM_018026 | PACS1 | -0.41 | NM_021935 | PROK2 | -0.41 |
| NM_024759 | FLJ13955 | -0.41 | NM_024895 | PDZK7 | -0.41 |
| NM_032228 | MLSTD2 | -0.41 | NM_173465 | COL23A1 | -0.41 |
| NM_145039 | MGC16385 | -0.41 | NM_033416 | IMP4 | -0.41 |
| NM_017954 | CADPS2 | -0.41 | NM_170694 | SERHL | -0.41 |
| NM_022754 | SFXN1 | -0.41 | NM_080607 | C20ORF102 | -0.41 |
| NM_144649 | FLJ33069 | -0.41 | NM_174906 | MGC39724 | -0.41 |
| NM_152889 | CHST13 | -0.41 | XM_088683 | DGAT2L4 | -0.41 |
| NM_130810 | DYX1C1 | -0.41 | XM_375027 | OR7E156P | -0.41 |
| XM_379195 | LOC285401 | -0.41 | NM_178175 | LHFPL1 | -0.41 |
| NM_181615 | KRTAP20-1 | -0.41 | XM_379098 | LOC339803 | -0.41 |
| NM_199005 | ZNF322B | -0.41 | NM_198526 | DKFZP547K1113 | -0.41 |
| NM_207465 | FLJ45337 | -0.41 | XM_166966 | LOC220429 | -0.41 |
| NM_203425 | LOC388407 | -0.41 | XM_209728 | LOC285703 | -0.41 |
| XM_113228 | LOC196266 | -0.41 | XM_372707 | LOC390880 | -0.41 |
| XM_379887 | LOC402565 | -0.41 | NM_001365 | DLG4 | -0.4 |
| NM_153445 | OR5P3 | -0.4 | NM_001254 | CDC6 | -0.4 |
| NM_005872 | BCAS2 | -0.4 | NM_005806 | OLIG2 | -0.4 |
| NM_003118 | SPARC | -0.4 | NM_006951 | TAF5 | -0.4 |
| NM_004231 | ATP6V1F | -0.4 | NM_001359 | DECR1 | -0.4 |
| NM_003676 | DEGS | -0.4 | NM_000932 | PLCB3 | -0.4 |
| NM_000057 | BLM | -0.4 | NM_021978 | ST14 | -0.4 |
| NM_013433 | TNPO2 | -0.4 | NM_000102 | CYP17A1 | -0.4 |
| NM_004526 | MCM2 | -0.4 | NM_000110 | DPYD | -0.4 |
| NM_002716 | PPP2R1B | -0.4 | NM_006384 | CIB1 | -0.4 |
| NM_001331 | CTNND1 | -0.4 | NM_004689 | MTA1 | -0.4 |
| NM_000574 | DAF | -0.4 | NM_003561 | PLA2G10 | -0.4 |
| NM_004339 | PTTG1IP | -0.4 | NM_001822 | CHN1 | -0.4 |
| NM_000780 | CYP7A1 | -0.4 | XM_208352 | ADH5B | -0.4 |
| NM_004630 | SF1 | -0.4 | NM_007173 | SPUVE | -0.4 |
| NM_015103 | PLXND1 | -0.4 | NM_014933 | SEC31L1 | -0.4 |
| NM_015187 | KIAA0746 | -0.4 | NM_001477 | GAGE7B | -0.4 |
| NM_019062 | FLJ20225 | -0.4 | NM_016538 | SIRT7 | -0.4 |
| NM_138284 | IL17D | -0.4 | NM_018337 | ZNF444 | -0.4 |
| NM_032309 | CHCHD5 | -0.4 | NM_032514 | MAP1LC3A | -0.4 |
| XM_372194 | MGC13275 | -0.4 | XM_027658 | FNDC1 | -0.4 |

| | | | | | |
|---|---|---|---|---|---|
| NM_033059 | KRTAP4-14 | -0.4 | NM_145244 | DDIT4L | -0.4 |
| NM_152308 | MGC24665 | -0.4 | NM_152284 | SHAX3 | -0.4 |
| NM_052857 | MGC20398 | -0.4 | XM_058513 | LRRK2 | -0.4 |
| NM_152332 | MTAC2D1 | -0.4 | NM_177549 | LOC149830 | -0.4 |
| NM_130902 | COX7B2 | -0.4 | XM_166376 | KIAA1949 | -0.4 |
| NM_145291 | ZNF509 | -0.4 | NM_152587 | MGC33948 | -0.4 |
| NM_206996 | PF6 | -0.4 | NM_207348 | LOC284723 | -0.4 |
| XM_291326 | KIAA2022 | -0.4 | XM_374319 | LOC339457 | -0.4 |
| NM_198502 | FLJ43826 | -0.4 | XM_208443 | LOC286526 | -0.4 |
| NM_198510 | UNQ6369 | -0.4 | XM_372199 | FLJ16518 | -0.4 |
| NM_207445 | FLJ39531 | -0.4 | XM_016093 | LOC143243 | -0.4 |
| XM_374117 | LOC389275 | -0.4 | XM_379518 | LOC401400 | -0.4 |
| XM_374751 | LOC392790 | -0.4 | XM_380011 | LOC402669 | -0.4 |
| XM_375108 | LOC400258 | -0.4 | XM_377122 | LOC401646 | -0.4 |
| XM_372378 | LOC390121 | -0.4 | XM_378807 | LOC400704 | -0.4 |
| XM_373721 | LOC388358 | -0.4 | XM_378573 | LOC400512 | -0.4 |
| NM_033516 | NYD-SP25 | -0.39 | NM_004297 | GNA14 | -0.39 |
| NM_005272 | GNAT2 | -0.39 | NM_003902 | FUBP1 | -0.39 |
| NM_004357 | CD151 | -0.39 | NM_004430 | EGR3 | -0.39 |
| NM_031944 | MIXL1 | -0.39 | NM_001290 | LDB2 | -0.39 |
| NM_014272 | ADAMTS7 | -0.39 | NM_004295 | TRAF4 | -0.39 |
| NM_001100 | ACTA1 | -0.39 | NM_152422 | PTPDC1 | -0.39 |
| NM_001709 | BDNF | -0.39 | NM_005563 | STMN1 | -0.39 |
| NM_005118 | TNFSF15 | -0.39 | NM_000966 | RARG | -0.39 |
| NM_000578 | SLC11A1 | -0.39 | NM_015665 | AAAS | -0.39 |
| NM_003658 | BARX2 | -0.39 | NM_017955 | CDCA4 | -0.39 |
| NM_001233 | CAV2 | -0.39 | NM_016221 | DCTN4 | -0.39 |
| NM_002157 | HSPE1 | -0.39 | NM_017440 | MDM1 | -0.39 |
| NM_022045 | MTBP | -0.39 | NM_030808 | NDEL1 | -0.39 |
| NM_004055 | CAPN5 | -0.39 | NM_004138 | KRTHA3A | -0.39 |
| NM_019109 | ALG1 | -0.39 | NM_002802 | PSMC1 | -0.39 |
| NM_003058 | SLC22A2 | -0.39 | NM_004182 | UXT | -0.39 |
| NM_014872 | ZBTB5 | -0.39 | NM_212481 | ARID5A | -0.39 |
| NM_015229 | KIAA0664 | -0.39 | NM_014007 | ZNF297B | -0.39 |
| NM_012105 | BACE2 | -0.39 | NM_016037 | CGI-94 | -0.39 |
| NM_018473 | THEM2 | -0.39 | NM_019557 | LOC56181 | -0.39 |
| NM_020064 | BARHL1 | -0.39 | NM_023924 | BRD9 | -0.39 |
| NM_024639 | ZNF322A | -0.39 | NM_024557 | RIC3 | -0.39 |
| NM_025008 | FLJ13544 | -0.39 | NM_025196 | GRPEL1 | -0.39 |
| NM_032433 | ZNF333 | -0.39 | NM_032621 | BEX2 | -0.39 |
| NM_032864 | FLJ14936 | -0.39 | NM_144569 | FLJ25348 | -0.39 |
| NM_130896 | WFDC8 | -0.39 | NM_014914 | CENTG2 | -0.39 |
| NM_138399 | LOC93109 | -0.39 | XM_058661 | C14ORF35 | -0.39 |
| NM_130809 | LOC133619 | -0.39 | NM_138467 | LOC127253 | -0.39 |
| NM_001001660 | LOC144363 | -0.39 | NM_152515 | FLJ40629 | -0.39 |
| NM_152585 | MGC33094 | -0.39 | NM_173563 | C6ORF146 | -0.39 |

| | | |
|---|---|---|
| NM_182569 | FLJ37451 | -0.39 |
| NM_182570 | FLJ25715 | -0.39 |
| NM_207375 | UNQ5792 | -0.39 |
| NM_207499 | FLJ41841 | -0.39 |
| XM_209489 | LOC285141 | -0.39 |
| XM_377886 | LOC402209 | -0.39 |
| XM_372583 | LOC390613 | -0.39 |
| XM_375639 | LOC400714 | -0.39 |
| XM_371111 | LOC388469 | -0.39 |
| NM_213720 | C22ORF16 | -0.39 |
| NM_001894 | CSNK1E | -0.38 |
| NM_006716 | ASK | -0.38 |
| NM_000294 | PHKG2 | -0.38 |
| NM_003328 | TXK | -0.38 |
| NM_012134 | LMOD1 | -0.38 |
| NM_001440 | EXTL3 | -0.38 |
| NM_003330 | TXNRD1 | -0.38 |
| NM_006197 | PCM1 | -0.38 |
| NM_000114 | EDN3 | -0.38 |
| NM_012404 | ANP32D | -0.38 |
| NM_012235 | SCAP | -0.38 |
| NM_001967 | EIF4A2 | -0.38 |
| NM_003513 | HIST1H2AB | -0.38 |
| NM_015044 | GGA2 | -0.38 |
| NM_014590 | ERVWE1 | -0.38 |
| NM_017885 | HCFC1R1 | -0.38 |
| NM_018368 | C6ORF209 | -0.38 |
| NM_019556 | MOSPD1 | -0.38 |
| NM_022897 | RANBP17 | -0.38 |
| NM_052940 | MGC8974 | -0.38 |
| NM_139161 | CRB3 | -0.38 |
| NM_175614 | NDUFA11 | -0.38 |
| NM_175887 | LOC222171 | -0.38 |
| NM_198486 | RPL7L1 | -0.38 |
| XM_208847 | LOC283804 | -0.38 |
| XM_291016 | LOC339804 | -0.38 |
| XM_167152 | LOC222701 | -0.38 |
| XM_376334 | LOC401147 | -0.38 |
| XM_380095 | LOC402472 | -0.38 |
| XM_375928 | LOC400855 | -0.38 |
| NM_000733 | CD3E | -0.37 |
| NM_002730 | PRKACA | -0.37 |
| NM_153838 | GPR115 | -0.37 |
| NM_002210 | ITGAV | -0.37 |
| NM_003112 | SP4 | -0.37 |
| NM_004035 | ACOX1 | -0.37 |
| NM_173608 | C14ORF80 | -0.39 |
| NM_178172 | LOC338328 | -0.39 |
| NM_207340 | LOC254359 | -0.39 |
| XM_085722 | LOC147166 | -0.39 |
| XM_071093 | LOC138799 | -0.39 |
| XM_374177 | LOC389415 | -0.39 |
| XM_372676 | LOC390801 | -0.39 |
| XM_372679 | LOC390812 | -0.39 |
| NM_001005325 | OR6M1 | -0.39 |
| XM_380026 | LOC402682 | -0.39 |
| NM_005876 | APEG1 | -0.38 |
| NM_002744 | PRKCZ | -0.38 |
| NM_004612 | TGFBR1 | -0.38 |
| NM_004392 | DACH1 | -0.38 |
| XM_052620 | MAN2B2 | -0.38 |
| NM_002526 | NT5E | -0.38 |
| NM_006310 | NPEPPS | -0.38 |
| NM_022151 | MOAP1 | -0.38 |
| NM_175068 | K6IRS3 | -0.38 |
| NM_002973 | SCA2 | -0.38 |
| NM_006096 | NDRG1 | -0.38 |
| NM_006305 | ANP32A | -0.38 |
| NM_003708 | RODH-4 | -0.38 |
| NM_016609 | SLC22A17 | -0.38 |
| NM_016403 | HSPC148 | -0.38 |
| NM_018157 | HSYN | -0.38 |
| NM_020139 | DHRS6 | -0.38 |
| NM_020820 | PREX1 | -0.38 |
| NM_152261 | MGC17943 | -0.38 |
| NM_152733 | BTBD9 | -0.38 |
| NM_144607 | FLJ32499 | -0.38 |
| NM_152372 | FLJ35961 | -0.38 |
| XM_374976 | LOC283331 | -0.38 |
| NM_182626 | FLJ25102 | -0.38 |
| XM_295062 | LOC339782 | -0.38 |
| XM_097792 | LOC150051 | -0.38 |
| XM_379075 | LOC400942 | -0.38 |
| XM_379424 | LOC401268 | -0.38 |
| XM_374261 | LOC389642 | -0.38 |
| NM_001005500 | OR4M1 | -0.38 |
| NM_004672 | MAP3K6 | -0.37 |
| NM_005592 | MUSK | -0.37 |
| NM_007158 | D1S155E | -0.37 |
| NM_006361 | HOXB13 | -0.37 |
| NM_130767 | CACH-1 | -0.37 |
| NM_001303 | COX10 | -0.37 |

| | | | | | |
|---|---|---|---|---|---|
| NM_004314 | ART1 | -0.37 | NM_004131 | GZMB | -0.37 |
| NM_006304 | SHFM1 | -0.37 | NM_000053 | ATP7B | -0.37 |
| NM_020242 | KNSL7 | -0.37 | NM_016224 | SNX9 | -0.37 |
| NM_002184 | IL6ST | -0.37 | NM_020996 | FGF6 | -0.37 |
| NM_000525 | KCNJ11 | -0.37 | NM_024649 | BBS1 | -0.37 |
| NM_025237 | SOST | -0.37 | NM_012305 | AP2A2 | -0.37 |
| NM_001129 | AEBP1 | -0.37 | NM_001450 | FHL2 | -0.37 |
| NM_004177 | STX3A | -0.37 | NM_003563 | SPOP | -0.37 |
| NM_004279 | PMPCB | -0.37 | NM_003770 | KRTHA7 | -0.37 |
| NM_003781 | B3GALT3 | -0.37 | NM_006554 | MTX2 | -0.37 |
| NM_015043 | KIAA0676 | -0.37 | XM_051197 | KIAA1005 | -0.37 |
| NM_015692 | CPAMD8 | -0.37 | NM_015955 | C2ORF4 | -0.37 |
| NM_015891 | CDC40 | -0.37 | NM_015378 | VPS13D | -0.37 |
| NM_020447 | C15ORF17 | -0.37 | NM_022067 | C14ORF133 | -0.37 |
| NM_024509 | LRFN3 | -0.37 | NM_024545 | SAP130 | -0.37 |
| NM_032752 | ZNF496 | -0.37 | NM_032918 | RERG | -0.37 |
| NM_058163 | DT1P1A10 | -0.37 | NM_144570 | C16ORF34 | -0.37 |
| NM_030649 | CENTB5 | -0.37 | NM_133493 | CD109 | -0.37 |
| NM_182758 | FLJ38736 | -0.37 | XM_374078 | LOC285548 | -0.37 |
| XM_372257 | UBE2NL | -0.37 | XM_379100 | LOC400958 | -0.37 |
| XM_379190 | LOC401062 | -0.37 | XM_373298 | LOC392347 | -0.37 |
| XM_372348 | LOC390037 | -0.37 | NM_001004304 | LOC283337 | -0.37 |
| XM_378620 | LOC400550 | -0.37 | XM_378957 | LOC400832 | -0.37 |
| NM_024046 | MGC8407 | -0.36 | NM_021970 | MAP2K1IP1 | -0.36 |
| XM_292627 | OR4D1 | -0.36 | NM_138465 | GLI4 | -0.36 |
| NM_005193 | CDX4 | -0.36 | NM_134442 | CREB1 | -0.36 |
| NM_001268 | CHC1L | -0.36 | NM_018975 | TERF2IP | -0.36 |
| XM_034274 | MYBL1 | -0.36 | NM_004376 | COX15 | -0.36 |
| NM_001917 | DAO | -0.36 | NM_006731 | FCMD | -0.36 |
| NM_016594 | FKBP11 | -0.36 | NM_000940 | PON3 | -0.36 |
| NM_001922 | DCT | -0.36 | NM_015884 | MBTPS2 | -0.36 |
| NM_033506 | FBXO24 | -0.36 | NM_004687 | MTMR4 | -0.36 |
| NM_012284 | KCNH3 | -0.36 | NM_017416 | IL1RAPL2 | -0.36 |
| NM_001065 | TNFRSF1A | -0.36 | NM_001880 | ATF2 | -0.36 |
| NM_014750 | DLG7 | -0.36 | NM_013260 | HCNGP | -0.36 |
| NM_004878 | PTGES | -0.36 | NM_020485 | RHCE | -0.36 |
| NM_173711 | MTND4 | -0.36 | NM_002935 | RNASE3 | -0.36 |
| NM_006674 | HCP5 | -0.36 | NM_007259 | VPS45A | -0.36 |
| XM_044580 | KIAA1024 | -0.36 | NM_015449 | NICE-3 | -0.36 |
| NM_015667 | C9ORF36 | -0.36 | NM_016022 | APH-1A | -0.36 |
| NM_015852 | H-PLK | -0.36 | NM_015937 | PIGT | -0.36 |
| XM_052597 | USP53 | -0.36 | NM_018993 | RIN2 | -0.36 |
| XM_032997 | MICAL3 | -0.36 | NM_020796 | SEMA6A | -0.36 |
| NM_020801 | ARRDC3 | -0.36 | NM_022453 | RNF25 | -0.36 |
| NM_024106 | ZNF426 | -0.36 | NM_025231 | ZNF435 | -0.36 |
| AK023591 | C9ORF13 | -0.36 | NM_173472 | MGC40179 | -0.36 |

| | | | | | |
|---|---|---|---|---|---|
| NM_152348 | FLJ33817 | -0.36 | NM_144613 | COXVIB2 | -0.36 |
| NM_172069 | KIAA2028 | -0.36 | NM_144618 | MGC29891 | -0.36 |
| NM_203394 | E2F7 | -0.36 | XM_378388 | LOC144742 | -0.36 |
| XM_066946 | LOC139886 | -0.36 | NM_201453 | LOC220869 | -0.36 |
| NM_152675 | FLJ23754 | -0.36 | NM_176886 | TAS2R45 | -0.36 |
| XM_290948 | LOC343071 | -0.36 | NM_207467 | FLJ35530 | -0.36 |
| XM_293633 | LOC344892 | -0.36 | XM_373981 | LOC388942 | -0.36 |
| XM_378200 | LOC399706 | -0.36 | XM_373545 | LOC387883 | -0.36 |
| XM_372472 | LOC390349 | -0.36 | XM_372556 | LOC390551 | -0.36 |
| XM_372190 | LOC389832 | -0.36 | NM_052947 | HAK | -0.35 |
| NM_000706 | AVPR1A | -0.35 | NM_175886 | PRPS1L1 | -0.35 |
| NM_014226 | RAGE | -0.35 | NM_018931 | PCDHB11 | -0.35 |
| NM_021570 | BARX1 | -0.35 | NM_021830 | PEO1 | -0.35 |
| NM_019034 | RHOF | -0.35 | NM_003369 | UVRAG | -0.35 |
| NM_004771 | MMP20 | -0.35 | NM_015320 | ARHGEF4 | -0.35 |
| NM_001429 | EP300 | -0.35 | NM_019111 | HLA-DRA | -0.35 |
| NM_003391 | WNT2 | -0.35 | NM_001628 | AKR1B1 | -0.35 |
| NM_004621 | TRPC6 | -0.35 | NM_012301 | AIP1 | -0.35 |
| NM_000560 | CD53 | -0.35 | NM_004956 | ETV1 | -0.35 |
| XM_039515 | G2 | -0.35 | NM_000818 | GAD2 | -0.35 |
| NM_006784 | WDR3 | -0.35 | NM_004927 | MRPL49 | -0.35 |
| NM_003361 | UMOD | -0.35 | NM_004271 | LY86 | -0.35 |
| NM_005730 | CTDSP2 | -0.35 | NM_006047 | RBM12 | -0.35 |
| NM_006795 | EHD1 | -0.35 | NM_014445 | SERP1 | -0.35 |
| NM_012384 | GMEB2 | -0.35 | NM_144991 | C21ORF29 | -0.35 |
| NM_019015 | CSGLCA-T | -0.35 | NM_017881 | C9ORF95 | -0.35 |
| NM_018471 | LEREPO4 | -0.35 | NM_020844 | KIAA1456 | -0.35 |
| NM_021806 | FAM3A | -0.35 | XM_352894 | DNCH2 | -0.35 |
| NM_024637 | GAL3ST4 | -0.35 | NM_025230 | WDR23 | -0.35 |
| NM_031301 | PSFL | -0.35 | XM_086186 | FLJ13815 | -0.35 |
| NM_032033 | FKSG43 | -0.35 | NM_032291 | DKFZP761D221 | -0.35 |
| NM_032811 | TBRG1 | -0.35 | NM_032840 | FLJ14800 | -0.35 |
| NM_152266 | MGC32020 | -0.35 | NM_153342 | FLJ90024 | -0.35 |
| XM_378546 | LOC145820 | -0.35 | XM_167149 | C6ORF194 | -0.35 |
| NM_173583 | FLJ33790 | -0.35 | NM_178539 | TAFA2 | -0.35 |
| NM_199346 | PFN4 | -0.35 | XM_374082 | LOC389212 | -0.35 |
| XM_374218 | LOC389506 | -0.35 | XM_374133 | LOC389318 | -0.35 |
| XM_376555 | LOC401286 | -0.35 | XM_376866 | LOC401524 | -0.35 |
| XM_372429 | LOC390245 | -0.35 | XM_377476 | LOC401884 | -0.35 |
| XM_375284 | LOC400481 | -0.35 | XM_371423 | LOC388846 | -0.35 |
| NM_002741 | PRKCL1 | -0.34 | NM_006293 | TYRO3 | -0.34 |
| NM_005288 | GPR12 | -0.34 | NM_004779 | CNOT8 | -0.34 |
| NM_014368 | LHX6 | -0.34 | NM_005359 | SMAD4 | -0.34 |
| NM_138430 | ADPRHL1 | -0.34 | NM_000784 | CYP27A1 | -0.34 |
| NM_033453 | ITPA | -0.34 | NM_030957 | ADAMTS10 | -0.34 |
| NM_016316 | REV1L | -0.34 | NM_000052 | ATP7A | -0.34 |

| | | |
|---|---|---|
| NM_012158 | FBXL3A | -0.34 |
| NM_001835 | CLTCL1 | -0.34 |
| NM_147130 | NCR3 | -0.34 |
| NM_000813 | GABRB2 | -0.34 |
| NM_005497 | GJA7 | -0.34 |
| NM_000349 | STAR | -0.34 |
| NM_000726 | CACNB4 | -0.34 |
| NM_018960 | GNMT | -0.34 |
| NM_021070 | LTBP3 | -0.34 |
| NM_016038 | SBDS | -0.34 |
| NM_004539 | NARS | -0.34 |
| NM_006985 | NPIP | -0.34 |
| NM_005826 | HNRPR | -0.34 |
| NM_016383 | HOM-TES-85 | -0.34 |
| XM_379021 | C21ORF30 | -0.34 |
| NM_018119 | POLR3E | -0.34 |
| NM_019118 | RP4-622L5 | -0.34 |
| XM_371398 | MYH7B | -0.34 |
| XM_352951 | MGC11349 | -0.34 |
| NM_032174 | FLJ12770 | -0.34 |
| XM_378183 | MGC5457 | -0.34 |
| NM_173499 | MGC44294 | -0.34 |
| NM_138574 | PWWP1 | -0.34 |
| NM_182553 | MGC50896 | -0.34 |
| XM_378431 | LOC283483 | -0.34 |
| XM_377506 | EMR4 | -0.34 |
| XM_170754 | LOC256394 | -0.34 |
| XM_298233 | LOC344709 | -0.34 |
| XM_377881 | LOC402204 | -0.34 |
| XM_376809 | LOC401485 | -0.34 |
| NM_012377 | OR7C2 | -0.33 |
| NM_177551 | GPR109A | -0.33 |
| NM_003919 | SGCE | -0.33 |
| NM_004503 | HOXC6 | -0.33 |
| NM_002599 | PDE2A | -0.33 |
| NM_001749 | CAPNS1 | -0.33 |
| NM_006221 | PIN1 | -0.33 |
| NM_006793 | PRDX3 | -0.33 |
| NM_003268 | TLR5 | -0.33 |
| NM_001171 | ABCC6 | -0.33 |
| NM_012141 | DDX26 | -0.33 |
| NM_032213 | RBM29 | -0.33 |
| NM_005411 | SFTPA1 | -0.33 |
| NM_004378 | CRABP1 | -0.33 |
| XM_372810 | PLXNA2 | -0.33 |
| NM_030754 | SAA2 | -0.33 |
| NM_001480 | GALR1 | -0.34 |
| NM_014906 | PPM1E | -0.34 |
| NM_002170 | IFNA8 | -0.34 |
| NM_002508 | NID | -0.34 |
| NM_012129 | CLDN12 | -0.34 |
| NM_019590 | KIAA1217 | -0.34 |
| NM_014361 | CNTN5 | -0.34 |
| NM_006478 | GAS2L1 | -0.34 |
| NM_006215 | SERPINA4 | -0.34 |
| NM_002893 | RBBP7 | -0.34 |
| NM_003176 | SYCP1 | -0.34 |
| NM_014790 | KIAA0555 | -0.34 |
| NM_007015 | LECT1 | -0.34 |
| NM_016091 | EIF3S6IP | -0.34 |
| NM_018145 | FLJ10579 | -0.34 |
| NM_018052 | FLJ10305 | -0.34 |
| NM_021640 | C12ORF10 | -0.34 |
| NM_021930 | RINT-1 | -0.34 |
| NM_024921 | POF1B | -0.34 |
| NM_032423 | ZNF528 | -0.34 |
| NM_032340 | C6ORF125 | -0.34 |
| NM_203374 | LOC147808 | -0.34 |
| NM_178126 | LOC162427 | -0.34 |
| NM_175735 | LYG2 | -0.34 |
| NM_182589 | HTR3E | -0.34 |
| XM_290936 | MGC35030 | -0.34 |
| XM_293121 | LOC343629 | -0.34 |
| XM_172874 | LOC253724 | -0.34 |
| XM_376648 | LOC401387 | -0.34 |
| NM_006213 | PHKG1 | -0.33 |
| NM_013308 | H963 | -0.33 |
| NM_033297 | NALP12 | -0.33 |
| NM_005438 | FOSL1 | -0.33 |
| NM_012406 | PRDM4 | -0.33 |
| NM_002153 | HSD17B2 | -0.33 |
| NM_002911 | RENT1 | -0.33 |
| NM_002425 | MMP10 | -0.33 |
| NM_002981 | CCL1 | -0.33 |
| NM_002059 | GH2 | -0.33 |
| NM_000065 | C6 | -0.33 |
| XM_496318 | DMWD | -0.33 |
| NM_002269 | KPNA5 | -0.33 |
| NM_004037 | AMPD2 | -0.33 |
| NM_001890 | CSN1S1 | -0.33 |
| NM_017419 | ACCN5 | -0.33 |
| NM_007152 | ZNF195 | -0.33 |

| | | | | | |
|---|---|---|---|---|---|
| NM_006295 | VARS2 | -0.33 | NM_006685 | PROL3 | -0.33 |
| NM_015400 | DKFZP586N0721 | -0.33 | NM_016492 | RANGNRF | -0.33 |
| NM_015974 | CRYL1 | -0.33 | NM_016013 | NDUFAF1 | -0.33 |
| NM_016028 | SUV420H1 | -0.33 | NM_016642 | SPTBN5 | -0.33 |
| NM_018424 | EPB41L4B | -0.33 | NM_017909 | C6ORF96 | -0.33 |
| NM_017680 | ASPN | -0.33 | NM_017706 | FLJ20195 | -0.33 |
| NM_017797 | BTBD2 | -0.33 | NM_019593 | KIAA1434 | -0.33 |
| NM_020457 | THAP11 | -0.33 | NM_024775 | GEMIN6 | -0.33 |
| XM_370575 | FBXL15 | -0.33 | NM_030579 | CYB5-M | -0.33 |
| NM_025076 | UXS1 | -0.33 | NM_030927 | DC-TM4F2 | -0.33 |
| NM_032710 | MGC13053 | -0.33 | NM_153750 | C21ORF81 | -0.33 |
| NM_145345 | SOC | -0.33 | NM_058164 | OLFM2 | -0.33 |
| NM_052863 | SCGB3A1 | -0.33 | NM_133456 | APXL2 | -0.33 |
| NM_144683 | MGC23280 | -0.33 | NM_024944 | CHODL | -0.33 |
| NM_152717 | MGC35295 | -0.33 | NM_198472 | C10ORF125 | -0.33 |
| NM_173082 | SHPRH | -0.33 | NM_173829 | FLJ36754 | -0.33 |
| XM_374860 | TP53I5 | -0.33 | NM_205855 | UNQ1940 | -0.33 |
| NM_005128 | C21ORF5 | -0.33 | XM_117408 | LOC202546 | -0.33 |
| XM_209920 | LOC286157 | -0.33 | XM_371504 | LOC388965 | -0.33 |
| XM_372328 | LOC390006 | -0.33 | XM_371018 | LOC388337 | -0.33 |
| NM_001004355 | FLJ31132 | -0.33 | NM_054111 | IHPK3 | -0.32 |
| NM_014916 | LMTK2 | -0.32 | NM_001295 | CCR1 | -0.32 |
| NM_000132 | F8 | -0.32 | NM_000439 | PCSK1 | -0.32 |
| NM_007260 | LYPLA2 | -0.32 | NM_000375 | UROS | -0.32 |
| NM_133638 | ADAMTS19 | -0.32 | NM_015941 | ATP6V1H | -0.32 |
| NM_001526 | HCRTR2 | -0.32 | NM_002985 | CCL5 | -0.32 |
| NM_000632 | ITGAM | -0.32 | NM_000806 | GABRA1 | -0.32 |
| NM_016187 | BIN2 | -0.32 | NM_004113 | FGF12 | -0.32 |
| NM_003036 | SKI | -0.32 | NM_023009 | MLP | -0.32 |
| NM_015480 | PVRL3 | -0.32 | NM_139166 | STARS | -0.32 |
| NM_004363 | CEACAM5 | -0.32 | NM_004132 | HABP2 | -0.32 |
| NM_002794 | PSMB2 | -0.32 | NM_001028 | RPS25 | -0.32 |
| NM_003086 | SNAPC4 | -0.32 | NM_003710 | SPINT1 | -0.32 |
| NM_003693 | SCARF1 | -0.32 | XM_031102 | WDR22 | -0.32 |
| NM_032635 | NIFIE14 | -0.32 | NM_006617 | NES | -0.32 |
| NM_007229 | PACSIN2 | -0.32 | NM_203459 | KIAA1078 | -0.32 |
| NM_014961 | RIPX | -0.32 | NM_013370 | OKL38 | -0.32 |
| NM_015958 | CGI-30 | -0.32 | NM_017898 | FLJ20605 | -0.32 |
| NM_017615 | C10ORF86 | -0.32 | NM_018169 | FLJ10652 | -0.32 |
| NM_017640 | LRRC16 | -0.32 | NM_018304 | FLJ11029 | -0.32 |
| NM_020203 | MEPE | -0.32 | XM_370738 | CHD8 | -0.32 |
| NM_021259 | TMEM8 | -0.32 | NM_022358 | KCNK15 | -0.32 |
| NM_022485 | FLJ22405 | -0.32 | NM_032015 | RNF26 | -0.32 |
| NM_030639 | KIAA1701 | -0.32 | NM_138444 | KCTD12 | -0.32 |
| XM_037817 | FLJ31033 | -0.32 | NM_152267 | FLJ38628 | -0.32 |
| NM_174897 | BPIL3 | -0.32 | NM_153019 | TMPRSS6 | -0.32 |

| | | | | | |
|---|---|---|---|---|---|
| NM_207347 | C18ORF30 | -0.32 | XM_379280 | LOC285422 | -0.32 |
| NM_174978 | C14ORF39 | -0.32 | NM_007137 | ZNF81 | -0.32 |
| XM_063336 | LOC122867 | -0.32 | XM_292824 | LOC342969 | -0.32 |
| XM_372822 | LOC391191 | -0.32 | XM_371621 | LOC389099 | -0.32 |
| XM_374021 | LOC389067 | -0.32 | XM_375410 | LOC400574 | -0.32 |
| XM_373906 | LOC388786 | -0.32 | NM_001004311 | FIGLA | -0.32 |
| XM_373964 | LOC388910 | -0.32 | XM_032491 | IKBKB | -0.31 |
| NM_002138 | HNRPD | -0.31 | NM_005221 | DLX5 | -0.31 |
| NM_0055633 | SOS1 | -0.31 | NM_014009 | FOXP3 | -0.31 |
| NM_000665 | ACHE | -0.31 | NM_001428 | ENO1 | -0.31 |
| NM_002719 | PPP2R5C | -0.31 | NM_016509 | CLEC2 | -0.31 |
| NM_014288 | ITGB3BP | -0.31 | NM_003476 | CSRP3 | -0.31 |
| XM_371755 | RGNEF | -0.31 | NM_003595 | TPST2 | -0.31 |
| NM_003612 | SEMA7A | -0.31 | XM_496278 | ZNF516 | -0.31 |
| NM_006358 | SLC25A17 | -0.31 | NM_032102 | SRP46 | -0.31 |
| NM_007187 | WBP4 | -0.31 | NM_014182 | ORMDL2 | -0.31 |
| NM_013271 | PCSK1N | -0.31 | NM_015993 | TM4SF11 | -0.31 |
| NM_016222 | DDX41 | -0.31 | NM_019045 | DKFZP686L20145 | -0.31 |
| NM_019058 | DDIT4 | -0.31 | NM_017681 | FLJ20130 | -0.31 |
| NM_018035 | FLJ10241 | -0.31 | NM_018121 | C10ORF6 | -0.31 |
| NM_020179 | FN5 | -0.31 | NM_020441 | CORO1B | -0.31 |
| XM_290768 | C17ORF27 | -0.31 | NM_022916 | VPS33A | -0.31 |
| NM_032115 | KCNK16 | -0.31 | NM_032391 | PRAC | -0.31 |
| NM_178122 | LOC90529 | -0.31 | NM_138362 | LOC90736 | -0.31 |
| NM_053016 | PALM2 | -0.31 | NM_138784 | LOC116123 | -0.31 |
| NM_024514 | CYP2R1 | -0.31 | NM_130847 | AMOTL1 | -0.31 |
| NM_181645 | FLJ25393 | -0.31 | NM_153370 | PI16 | -0.31 |
| XM_166184 | SLC37A2 | -0.31 | NM_182539 | MGC33600 | -0.31 |
| NM_153331 | KCTD6 | -0.31 | NM_173668 | C5ORF17 | -0.31 |
| NM_213605 | ZNF517 | -0.31 | XM_376665 | LOC286009 | -0.31 |
| NM_206886 | NY-SAR-41 | -0.31 | NM_001001852 | PIM3 | -0.31 |
| XM_211009 | LOC283389 | -0.31 | XM_166776 | LOC219869 | -0.31 |
| XM_378840 | LOC400740 | -0.31 | XM_374064 | LOC389182 | -0.31 |
| XM_379638 | LOC401518 | -0.31 | XM_372452 | LOC390299 | -0.31 |
| NM_005356 | LCK | -0.3 | NM_020400 | GPR92 | -0.3 |
| NM_002531 | NTSR1 | -0.3 | NM_016280 | CES4 | -0.3 |
| NM_006593 | TBR1 | -0.3 | NM_004390 | CTSH | -0.3 |
| NM_014762 | DHCR24 | -0.3 | NM_000181 | GUSB | -0.3 |
| NM_002768 | PCOLN3 | -0.3 | NM_000414 | HSD17B4 | -0.3 |
| NM_002708 | PPP1CA | -0.3 | NM_002830 | PTPN4 | -0.3 |
| NM_016941 | DLL3 | -0.3 | NM_031889 | ENAM | -0.3 |
| NM_012448 | STAT5B | -0.3 | NM_003062 | SLIT3 | -0.3 |
| NM_021098 | CACNA1H | -0.3 | NM_018645 | HES6 | -0.3 |
| BC050399 | RFNG | -0.3 | NM_004585 | RARRES3 | -0.3 |
| NM_015696 | GPX7 | -0.3 | NM_021983 | HLA-DRB4 | -0.3 |
| NM_012332 | ACATE2 | -0.3 | NM_173511 | ALS2CR13 | -0.3 |

| | | | | | |
|---|---|---|---|---|---|
| NM_003491 | ARD1 | -0.3 | NM_003679 | KMO | -0.3 |
| NM_003890 | FCGBP | -0.3 | NM_014859 | KIAA0672 | -0.3 |
| NM_014874 | MFN2 | -0.3 | NM_007180 | TREH | -0.3 |
| XM_044632 | KIAA0556 | -0.3 | XM_290944 | PLEKHM2 | -0.3 |
| NM_015321 | MECT1 | -0.3 | NM_013262 | MYLIP | -0.3 |
| XM_375337 | FBXL19 | -0.3 | NM_017953 | FLJ20729 | -0.3 |
| NM_018042 | FLJ10260 | -0.3 | NM_017609 | C10ORF92 | -0.3 |
| NM_018088 | FLJ10408 | -0.3 | NM_017958 | PLEKHB2 | -0.3 |
| NM_018306 | FLJ11036 | -0.3 | NM_021225 | PROL1 | -0.3 |
| XM_030665 | KIAA1229 | -0.3 | NM_022767 | FLJ12484 | -0.3 |
| NM_024028 | MGC3265 | -0.3 | NM_025125 | C10ORF57 | -0.3 |
| NM_024910 | FLJ12700 | -0.3 | NM_032900 | ARHGAP19 | -0.3 |
| NM_080861 | SSB3 | -0.3 | NM_173463 | DKFZP761B107 | -0.3 |
| NM_052933 | TSGA13 | -0.3 | NM_138788 | LOC120224 | -0.3 |
| NM_153016 | FLJ30672 | -0.3 | NM_173542 | LOC196463 | -0.3 |
| NM_153451 | ORAOV1 | -0.3 | NM_172368 | G30 | -0.3 |
| XM_211291 | LOC283994 | -0.3 | XM_371505 | LOC388968 | -0.3 |
| NM_207388 | FLJ31222 | -0.3 | NM_206894 | MGC62100 | -0.3 |
| NM_198573 | UNQ470 | -0.3 | XM_292958 | LOC344167 | -0.3 |
| XM_096883 | LOC145846 | -0.3 | XM_085200 | LOC145660 | -0.3 |
| XM_379136 | LOC401006 | -0.3 | XM_374093 | LOC389236 | -0.3 |
| XM_372118 | FLJ43859 | -0.3 | XM_370570 | LOC387702 | -0.3 |
| NM_213726 | INCA1 | -0.3 | NM_001929 | DGUOK | -0.29 |
| NM_020185 | DUSP22 | -0.29 | NM_002734 | PRKAR1A | -0.29 |
| NM_025247 | MGC5601 | -0.29 | NM_021972 | SPHK1 | -0.29 |
| NM_002983 | CCL3 | -0.29 | NM_013940 | OR10H1 | -0.29 |
| NM_004948 | DSC1 | -0.29 | NM_019069 | WDR5B | -0.29 |
| NM_007276 | CBX3 | -0.29 | NM_006559 | KHDRBS1 | -0.29 |
| NM_003412 | ZIC1 | -0.29 | NM_005996 | TBX3 | -0.29 |
| NM_002905 | RDH5 | -0.29 | NM_003033 | SIAT4A | -0.29 |
| NM_018319 | TDP1 | -0.29 | NM_003227 | TFR2 | -0.29 |
| NM_052995 | USH3A | -0.29 | NM_006789 | APOBEC2 | -0.29 |
| NM_002581 | PAPPA | -0.29 | NM_000465 | BARD1 | -0.29 |
| NM_012283 | KCNG2 | -0.29 | NM_024529 | HRPT2 | -0.29 |
| NM_005851 | DOC-1R | -0.29 | NM_015855 | WIT-1 | -0.29 |
| NM_005973 | PRCC | -0.29 | NM_002857 | PEX19 | -0.29 |
| NM_000262 | NAGA | -0.29 | NM_003971 | SPAG9 | -0.29 |
| XM_043613 | GRID1 | -0.29 | NM_024007 | EBF | -0.29 |
| NM_001502 | GP2 | -0.29 | NM_002695 | POLR2E | -0.29 |
| NM_004548 | NDUFB10 | -0.29 | NM_032270 | AD158 | -0.29 |
| NM_006955 | ZNF11B | -0.29 | NM_017413 | APLN | -0.29 |
| NM_014647 | LKAP | -0.29 | NM_004275 | TRFP | -0.29 |
| NM_006631 | ZNF266 | -0.29 | NM_015305 | KIAA0759 | -0.29 |
| NM_015469 | NIPSNAP3A | -0.29 | NM_014174 | THY28 | -0.29 |
| NM_015971 | MRPS7 | -0.29 | NM_016649 | C20ORF6 | -0.29 |
| XM_291128 | RBM27 | -0.29 | NM_016467 | LOC51240 | -0.29 |

| | | | | | |
|---|---|---|---|---|---|
| NM_017737 | TOCA1 | -0.29 | NM_017679 | BCAS3 | -0.29 |
| NM_018387 | STRBP | -0.29 | XM_049078 | KIAA1239 | -0.29 |
| NM_021934 | FLJ11773 | -0.29 | NM_023930 | KCTD14 | -0.29 |
| NM_022826 | 39142 | -0.29 | NM_031959 | KRTAP3-2 | -0.29 |
| NM_138966 | NETO1 | -0.29 | NM_031288 | HMGA1L4 | -0.29 |
| NM_033104 | STN2 | -0.29 | NM_152337 | FLJ32702 | -0.29 |
| NM_175053 | K6IRS4 | -0.29 | NM_033207 | TMEM10 | -0.29 |
| NM_033198 | PIGS | -0.29 | NM_173487 | LOC132321 | -0.29 |
| NM_173849 | GSC | -0.29 | XM_051271 | FAM10A6 | -0.29 |
| NM_153773 | C21ORF99 | -0.29 | NM_153234 | C5ORF11 | -0.29 |
| NM_152715 | MGC10233 | -0.29 | XM_114973 | LOC203806 | -0.29 |
| NM_170746 | C11ORF31 | -0.29 | NM_181616 | KRTAP20-2 | -0.29 |
| NM_207411 | UNQ2754 | -0.29 | XM_172917 | LOC256453 | -0.29 |
| XM_212013 | LOC285777 | -0.29 | XM_373848 | LOC388641 | -0.29 |
| XM_378897 | LOC400781 | -0.29 | XM_374301 | LOC389778 | -0.29 |
| XM_373444 | LOC387630 | -0.29 | XM_378706 | LOC400621 | -0.29 |
| XM_380022 | LOC402679 | -0.29 | NM_001006116 | C21ORF49 | -0.29 |
| NM_001006117 | RBMY1J | -0.29 | NM_004327 | BCR | -0.28 |
| NM_017506 | OR7A5 | -0.28 | NM_022788 | P2RY12 | -0.28 |
| NM_003555 | OR1G1 | -0.28 | NM_012373 | OR3A3 | -0.28 |
| NM_000101 | CYBA | -0.28 | NM_005963 | MYH1 | -0.28 |
| NM_016307 | PRRX2 | -0.28 | NM_012391 | SPDEF | -0.28 |
| NM_021251 | CAPN10 | -0.28 | NM_004716 | PCSK7 | -0.28 |
| NM_024420 | PLA2G4A | -0.28 | NM_016238 | ANAPC7 | -0.28 |
| NM_022076 | DUSP21 | -0.28 | NM_021976 | RXRB | -0.28 |
| NM_006179 | NTF5 | -0.28 | NM_139075 | TPCN2 | -0.28 |
| NM_003666 | BLZF1 | -0.28 | NM_004930 | CAPZB | -0.28 |
| NM_016511 | CLEC1 | -0.28 | NM_004484 | GPC3 | -0.28 |
| NM_000122 | ERCC3 | -0.28 | NM_002168 | IDH2 | -0.28 |
| NM_016234 | ACSL5 | -0.28 | NM_006953 | UPK3A | -0.28 |
| NM_007084 | SOX21 | -0.28 | XM_038920 | N4BP3 | -0.28 |
| NM_015374 | UNC84B | -0.28 | NM_152257 | KIAA0889 | -0.28 |
| NM_015424 | CHRDL2 | -0.28 | NM_016955 | SLA/LP | -0.28 |
| NM_016058 | CGI-121 | -0.28 | NM_019053 | SEC15L1 | -0.28 |
| NM_053004 | GNB1L | -0.28 | NM_017676 | FLJ20125 | -0.28 |
| NM_018390 | FLJ11323 | -0.28 | NM_018074 | FLJ10374 | -0.28 |
| NM_020177 | FEM1C | -0.28 | NM_018698 | NXT2 | -0.28 |
| NM_020659 | TTYH1 | -0.28 | XM_371279 | KIAA1163 | -0.28 |
| NM_021626 | SCPEP1 | -0.28 | NM_021812 | BPESC1 | -0.28 |
| NM_020749 | MTUS1 | -0.28 | NM_022063 | C10ORF84 | -0.28 |
| NM_022913 | DKFZP761C169 | -0.28 | NM_022828 | YTHDC2 | -0.28 |
| NM_024027 | COLEC11 | -0.28 | NM_024560 | FLJ21963 | -0.28 |
| NM_032138 | KBTBD7 | -0.28 | NM_194327 | GALIG | -0.28 |
| NM_030954 | DKFZP564A022 | -0.28 | NM_031468 | CALN1 | -0.28 |
| NM_138418 | MGC15416 | -0.28 | NM_018718 | TSGA14 | -0.28 |
| XM_043624 | DKFZP434E1822 | -0.28 | XM_043863 | DKFZP434H2226 | -0.28 |

| | | |
|---|---|---|
| NM_031908 | C1QTNF2 | -0.28 |
| XM_376284 | LOC93556 | -0.28 |
| XM_376372 | LOC134121 | -0.28 |
| NM_145205 | LOC127540 | -0.28 |
| NM_144682 | FLJ31952 | -0.28 |
| NM_153454 | C21ORF86 | -0.28 |
| NM_198080 | LOC253827 | -0.28 |
| XM_378971 | LOC284798 | -0.28 |
| NM_198580 | SLC27A1 | -0.28 |
| NM_207462 | FLJ45684 | -0.28 |
| XM_295166 | LOC340148 | -0.28 |
| XM_374653 | LOC392993 | -0.28 |
| XM_378215 | LOC399726 | -0.28 |
| XM_372272 | LOC389905 | -0.28 |
| NM_000154 | GALK1 | -0.27 |
| NM_005904 | SMAD7 | -0.27 |
| NM_017872 | ICF45 | -0.27 |
| NM_001050 | SSTR2 | -0.27 |
| NM_004671 | PIAS2 | -0.27 |
| NM_006777 | ZBTB33 | -0.27 |
| NM_012231 | PRDM2 | -0.27 |
| NM_004346 | CASP3 | -0.27 |
| NM_001047 | SRD5A1 | -0.27 |
| NM_022349 | MS4A6A | -0.27 |
| NM_004185 | WNT2B | -0.27 |
| NM_001138 | AGRP | -0.27 |
| NM_014665 | LRRC14 | -0.27 |
| NM_178460 | PTPNS1L2 | -0.27 |
| NM_004944 | DNASE1L3 | -0.27 |
| NM_002642 | PIGC | -0.27 |
| NM_004858 | SLC4A8 | -0.27 |
| NM_006331 | C2F | -0.27 |
| NM_007281 | SCRG1 | -0.27 |
| NM_014018 | MRPS28 | -0.27 |
| NM_019073 | SPATA6 | -0.27 |
| NM_017833 | C21ORF55 | -0.27 |
| NM_018212 | ENAH | -0.27 |
| XM_051862 | LOC58489 | -0.27 |
| NM_030641 | APOL6 | -0.27 |
| NM_030958 | SLCO5A1 | -0.27 |
| XM_030892 | ZNF616 | -0.27 |
| NM_174889 | LOC91942 | -0.27 |
| NM_052971 | LEAP-2 | -0.27 |
| NM_080831 | DEFB129 | -0.27 |
| NM_153239 | KIAA1924 | -0.27 |
| XM_291007 | LOC339766 | -0.27 |
| XM_378250 | LOC92482 | -0.28 |
| XM_058720 | KIAA1971 | -0.28 |
| NM_144617 | HSPB6 | -0.28 |
| NM_145265 | LOC133957 | -0.28 |
| NM_153290 | FAM10A4 | -0.28 |
| NM_173584 | MGC45840 | -0.28 |
| NM_152757 | FLJ30313 | -0.28 |
| NM_181489 | ZNF445 | -0.28 |
| NM_207451 | FLJ45121 | -0.28 |
| XM_068121 | LOC132969 | -0.28 |
| XM_371331 | LOC388722 | -0.28 |
| XM_374315 | LOC389815 | -0.28 |
| NM_001005235 | OR1L4 | -0.28 |
| XM_379881 | LOC402559 | -0.28 |
| NM_014215 | INSRR | -0.27 |
| NM_014720 | SLK | -0.27 |
| NM_152779 | MGC26856 | -0.27 |
| NM_022105 | DATF1 | -0.27 |
| NM_005937 | MLLT6 | -0.27 |
| NM_002167 | ID3 | -0.27 |
| NM_003709 | KLF7 | -0.27 |
| NM_001535 | HRMT1L1 | -0.27 |
| NM_006398 | UBD | -0.27 |
| NM_005505 | SCARB1 | -0.27 |
| NM_002206 | ITGA7 | -0.27 |
| NM_001521 | GTF3C2 | -0.27 |
| NM_014389 | PELP1 | -0.27 |
| NM_021117 | CRY2 | -0.27 |
| NM_004524 | LLGL2 | -0.27 |
| NM_006749 | SLC20A2 | -0.27 |
| NM_005844 | HCG9 | -0.27 |
| NM_007034 | DNAJB4 | -0.27 |
| NM_015425 | POLR1A | -0.27 |
| NM_014188 | HSPC182 | -0.27 |
| NM_016226 | VPS29 | -0.27 |
| NM_018353 | C14ORF106 | -0.27 |
| NM_018836 | SHREW1 | -0.27 |
| NM_024602 | FLJ21156 | -0.27 |
| NM_025099 | FLJ22170 | -0.27 |
| XM_290496 | DIXDC1 | -0.27 |
| XM_036115 | ZC3HDC5 | -0.27 |
| NM_133371 | MYOZ3 | -0.27 |
| NM_145248 | LOC122258 | -0.27 |
| NM_052909 | KIAA1909 | -0.27 |
| NM_153023 | SPATA13 | -0.27 |
| NM_178130 | TXNDC6 | -0.27 |

| | | | | | |
|---|---|---|---|---|---|
| XM_208658 | LOC283398 | -0.27 | XM_291697 | LOC340745 | -0.27 |
| XM_166720 | LOC220998 | -0.27 | XM_379106 | LOC400964 | -0.27 |
| XM_373043 | LOC391723 | -0.27 | XM_374268 | LOC389656 | -0.27 |
| XM_374428 | LOC392661 | -0.27 | XM_375609 | LOC400693 | -0.27 |
| XM_375560 | LOC400673 | -0.27 | NM_001001915 | OR2G2 | -0.27 |
| NM_001006608 | STGC3 | -0.27 | NM_001826 | CKS1B | -0.26 |
| NM_018653 | GPRC5C | -0.26 | NM_030643 | APOL4 | -0.26 |
| NM_004096 | EIF4EBP2 | -0.26 | NM_004385 | CSPG2 | -0.26 |
| NM_006561 | CUGBP2 | -0.26 | NM_003379 | VIL2 | -0.26 |
| NM_006747 | SIPA1 | -0.26 | NM_005253 | FOSL2 | -0.26 |
| NM_002729 | HHEX | -0.26 | NM_006437 | ADPRTL1 | -0.26 |
| NM_020361 | CPA6 | -0.26 | NM_001905 | CTPS | -0.26 |
| NM_003748 | ALDH4A1 | -0.26 | NM_003793 | CTSF | -0.26 |
| NM_021044 | DHH | -0.26 | NM_001125 | ADPRH | -0.26 |
| NM_004181 | UCHL1 | -0.26 | NM_012072 | C1QR1 | -0.26 |
| NM_004123 | GIP | -0.26 | NM_019113 | FGF21 | -0.26 |
| NM_005142 | GIF | -0.26 | NM_004978 | KCNC4 | -0.26 |
| NM_005178 | BCL3 | -0.26 | NM_004702 | CCNE2 | -0.26 |
| NM_032818 | C9ORF100 | -0.26 | NM_003471 | KCNAB1 | -0.26 |
| NM_005779 | LHFPL2 | -0.26 | NM_005905 | SMAD9 | -0.26 |
| NM_016180 | MATP | -0.26 | NM_012364 | OR1Q1 | -0.26 |
| NM_003955 | SOCS3 | -0.26 | NM_003401 | XRCC4 | -0.26 |
| NM_005395 | PMS2L3 | -0.26 | NM_005988 | SPRR2A | -0.26 |
| NM_017503 | SURF2 | -0.26 | NM_004699 | DXS9928E | -0.26 |
| NM_014262 | LEPREL2 | -0.26 | XM_290517 | KIAA0404 | -0.26 |
| XM_039385 | KIAA1093 | -0.26 | NM_015343 | DULLARD | -0.26 |
| NM_015388 | C6ORF109 | -0.26 | NM_014065 | HT001 | -0.26 |
| NM_013240 | C21ORF127 | -0.26 | NM_018045 | FLJ10276 | -0.26 |
| NM_017921 | NPL4 | -0.26 | XM_038291 | FLJ13456 | -0.26 |
| NM_024578 | FLJ22709 | -0.26 | NM_032784 | THSD2 | -0.26 |
| NM_032448 | KIAA1838 | -0.26 | NM_138402 | LOC93349 | -0.26 |
| NM_145168 | HSPC105 | -0.26 | NM_152314 | MGC34830 | -0.26 |
| NM_174900 | ZFP42 | -0.26 | XM_086826 | LOC150368 | -0.26 |
| NM_138336 | LOC150678 | -0.26 | XM_371263 | MCOLN2 | -0.26 |
| NM_173616 | FLJ35894 | -0.26 | NM_198850 | LOC284345 | -0.26 |
| NM_198493 | FLJ45235 | -0.26 | XM_291485 | LOC343263 | -0.26 |
| XM_211853 | LOC285344 | -0.26 | XM_016532 | LOC220793 | -0.26 |
| XM_098450 | LOC153959 | -0.26 | XM_209910 | LOC286129 | -0.26 |
| XM_372992 | LOC391542 | -0.26 | XM_374070 | LOC389188 | -0.26 |
| XM_372486 | LOC390377 | -0.26 | XM_373672 | LOC388235 | -0.26 |
| XM_373395 | LOC392582 | -0.26 | NM_002513 | NME3 | -0.25 |
| NM_001792 | CDH2 | -0.25 | NM_014424 | HSPB7 | -0.25 |
| NM_013347 | HSU24186 | -0.25 | NM_006608 | PHTF1 | -0.25 |
| NM_006965 | ZNF24 | -0.25 | NM_003429 | ZNF85 | -0.25 |
| NM_015636 | EIF2B4 | -0.25 | NM_000056 | BCKDHB | -0.25 |
| NM_000128 | F11 | -0.25 | NM_014815 | THRAP4 | -0.25 |

| | | | | | |
|---|---|---|---|---|---|
| NM_000351 | STS | -0.25 | NM_016178 | OAZ3 | -0.25 |
| NM_006074 | TRIM22 | -0.25 | NM_007317 | KIF22 | -0.25 |
| NM_006653 | FRS3 | -0.25 | NM_005817 | M6PRBP1 | -0.25 |
| NM_006854 | KDELR2 | -0.25 | NM_007036 | ESM1 | -0.25 |
| NM_005731 | ARPC2 | -0.25 | NM_016370 | RAB9B | -0.25 |
| NM_000384 | APOB | -0.25 | NM_001131 | CRISP1 | -0.25 |
| NM_139320 | CHRFAM7A | -0.25 | NM_000195 | HPS1 | -0.25 |
| NM_015525 | IBTK | -0.25 | NM_002825 | PTN | -0.25 |
| NM_001158 | AOC2 | -0.25 | NM_001745 | CAMLG | -0.25 |
| NM_002159 | HTN1 | -0.25 | NM_004466 | GPC5 | -0.25 |
| NM_002997 | SDC1 | -0.25 | NM_006026 | H1FX | -0.25 |
| NM_005096 | ZNF261 | -0.25 | XM_376232 | VPRBP | -0.25 |
| NM_007072 | HHLA2 | -0.25 | NM_006467 | POLR3G | -0.25 |
| NM_015014 | KIAA0117 | -0.25 | XM_291159 | DKFZP434F011 | -0.25 |
| NM_017421 | COQ3 | -0.25 | NM_017739 | FLJ20277 | -0.25 |
| XM_378191 | PRO2964 | -0.25 | NM_020205 | ZA20D1 | -0.25 |
| XM_371352 | FMN2 | -0.25 | NM_022898 | BCL11B | -0.25 |
| NM_031939 | MRO | -0.25 | NM_032214 | SLA2 | -0.25 |
| NM_032549 | IMMP2L | -0.25 | NM_032136 | DKFZP434L1717 | -0.25 |
| NM_080665 | MGC19604 | -0.25 | NM_138363 | LOC90799 | -0.25 |
| NM_080656 | MGC13017 | -0.25 | NM_145165 | CHURC1 | -0.25 |
| NM_144599 | SPG6 | -0.25 | NM_172000 | HE9 | -0.25 |
| NM_152478 | ZNF583 | -0.25 | XM_379117 | LOC150568 | -0.25 |
| NM_152581 | MOSPD2 | -0.25 | NM_181646 | FLJ32110 | -0.25 |
| NM_145115 | ZNF498 | -0.25 | XM_291816 | OTOG | -0.25 |
| XM_293398 | RAB41 | -0.25 | NM_207422 | FLJ44635 | -0.25 |
| XM_291645 | LOC343563 | -0.25 | XM_172995 | LOC255809 | -0.25 |
| XM_373822 | LOC388587 | -0.25 | XM_371816 | LOC389384 | -0.25 |
| XM_380112 | LOC402515 | -0.25 | XM_373266 | LOC392275 | -0.25 |
| XM_373540 | LOC387872 | -0.25 | XM_375031 | LOC400145 | -0.25 |
| XM_371192 | LOC388561 | -0.25 | XM_371424 | LOC388847 | -0.25 |
| NM_025195 | TRIB1 | -0.24 | NM_006137 | CD7 | -0.24 |
| NM_005391 | PDK3 | -0.24 | NM_004783 | TAO1 | -0.24 |
| NM_054021 | GPR101 | -0.24 | NM_153444 | OR5P2 | -0.24 |
| NM_001797 | CDH11 | -0.24 | NM_001279 | CIDEA | -0.24 |
| NM_022161 | BIRC7 | -0.24 | NM_007355 | HSPCB | -0.24 |
| NM_000893 | KNG1 | -0.24 | NM_015897 | PIAS4 | -0.24 |
| NM_015983 | LOC51619 | -0.24 | NM_024080 | TRPM8 | -0.24 |
| NM_003320 | TUB | -0.24 | NM_004792 | PPIG | -0.24 |
| NM_032833 | PPP1R15B | -0.24 | NM_007066 | PKIG | -0.24 |
| NM_080744 | SRCRB4D | -0.24 | NM_031907 | USP26 | -0.24 |
| NM_014489 | FRAG1 | -0.24 | NM_006264 | PTPN13 | -0.24 |
| NM_006273 | CCL7 | -0.24 | NM_001781 | CD69 | -0.24 |
| NM_000109 | DMD | -0.24 | NM_001205 | BNIP1 | -0.24 |
| NM_016426 | GTSE1 | -0.24 | NM_138387 | G6PC3 | -0.24 |
| NM_005338 | HIP1 | -0.24 | NM_002520 | NPM1 | -0.24 |

| | | | | | |
|---|---|---|---|---|---|
| NM_006715 | MAN2C1 | -0.24 | NM_007100 | ATP5I | -0.24 |
| NM_005503 | APBA2 | -0.24 | NM_002705 | PPL | -0.24 |
| NM_005049 | PWP2H | -0.24 | NM_004286 | GTPBP1 | -0.24 |
| NM_014171 | CRIPT | -0.24 | NM_004855 | PIGB | -0.24 |
| NM_015575 | TNRC15 | -0.24 | NM_013305 | SIAT8E | -0.24 |
| NM_016301 | MGC14560 | -0.24 | NM_017847 | C1ORF27 | -0.24 |
| NM_017861 | FLJ20522 | -0.24 | NM_020665 | TMEM27 | -0.24 |
| XM_039698 | KIAA1432 | -0.24 | NM_022103 | FLJ14011 | -0.24 |
| NM_022787 | NMNAT1 | -0.24 | NM_022486 | SUSD1 | -0.24 |
| NM_024092 | MGC5508 | -0.24 | NM_024648 | FLJ22222 | -0.24 |
| NM_024570 | FLJ11712 | -0.24 | NM_025061 | FLJ23420 | -0.24 |
| NM_025079 | FLJ23231 | -0.24 | XM_371706 | KIAA1109 | -0.24 |
| NM_032032 | FKSG42 | -0.24 | NM_032790 | FLJ14466 | -0.24 |
| NM_033120 | NKD2 | -0.24 | NM_152326 | ANKRD9 | -0.24 |
| NM_182504 | WBSCR28 | -0.24 | NM_153703 | PODN | -0.24 |
| NM_203495 | COMMD6 | -0.24 | NM_138969 | RDH-E2 | -0.24 |
| NM_145292 | GALNT15 | -0.24 | NM_198461 | FLJ45273 | -0.24 |
| XM_114456 | LOC202181 | -0.24 | XM_291464 | LOC343221 | -0.24 |
| XM_060537 | LOC127550 | -0.24 | XM_209097 | LOC284269 | -0.24 |
| XM_113971 | APM-1 | -0.24 | XM_372827 | LOC391196 | -0.24 |
| XM_375688 | LOC400736 | -0.24 | XM_373843 | LOC388628 | -0.24 |
| XM_372973 | LOC391491 | -0.24 | XM_371622 | LOC389101 | -0.24 |
| XM_377889 | LOC402211 | -0.24 | XM_379417 | LOC401264 | -0.24 |
| XM_371182 | LOC388554 | -0.24 | XM_372261 | LOC389900 | -0.24 |
| NM_005235 | ERBB4 | -0.23 | NM_005233 | EPHA3 | -0.23 |
| NM_000707 | AVPR1B | -0.23 | NM_000843 | GRM6 | -0.23 |
| NM_000890 | KCNJ5 | -0.23 | NM_001388 | DRG2 | -0.23 |
| NM_014718 | CLSTN3 | -0.23 | NM_000021 | PSEN1 | -0.23 |
| NM_002881 | RALB | -0.23 | NM_000412 | HRG | -0.23 |
| NM_006297 | XRCC1 | -0.23 | NM_004309 | ARHGDIA | -0.23 |
| NM_006602 | TCFL5 | -0.23 | NM_003420 | ZNF35 | -0.23 |
| NM_001545 | ICT1 | -0.23 | NM_139073 | SPATA3 | -0.23 |
| XM_291141 | KIAA0303 | -0.23 | NM_004184 | WARS | -0.23 |
| NM_005552 | KNS2 | -0.23 | NM_003479 | PTP4A2 | -0.23 |
| NM_001768 | CD8A | -0.23 | NM_002943 | RORA | -0.23 |
| NM_005554 | KRT6A | -0.23 | NM_001856 | COL16A1 | -0.23 |
| NM_000094 | COL7A1 | -0.23 | NM_005900 | SMAD1 | -0.23 |
| NM_015627 | ARH | -0.23 | NM_005370 | RAB8A | -0.23 |
| NM_005097 | LGI1 | -0.23 | NM_014485 | PGDS | -0.23 |
| NM_017705 | MPRG | -0.23 | XM_039548 | SMYD5 | -0.23 |
| NM_004168 | SDHA | -0.23 | NM_021727 | FADS3 | -0.23 |
| NM_007168 | ABCA8 | -0.23 | NM_014222 | NDUFA8 | -0.23 |
| NM_015256 | ACSL6 | -0.23 | NM_002638 | PI3 | -0.23 |
| NM_002950 | RPN1 | -0.23 | NM_170750 | PSMD10 | -0.23 |
| NM_006507 | REG1B | -0.23 | NM_006754 | SYPL | -0.23 |
| NM_014747 | RIMS3 | -0.23 | NM_005771 | DHRS9 | -0.23 |

| | | | | | |
|---|---|---|---|---|---|
| NM_015018 | KIAA1117 | -0.23 | NM_015072 | KIAA0998 | -0.23 |
| NM_032109 | OTP | -0.23 | NM_015604 | WDR21 | -0.23 |
| NM_016490 | LOC51252 | -0.23 | NM_015889 | PCQAP | -0.23 |
| NM_016578 | HBXAP | -0.23 | XM_371258 | KIAA1579 | -0.23 |
| NM_018697 | LANCL2 | -0.23 | NM_018926 | PCDHGB6 | -0.23 |
| NM_020682 | CYT19 | -0.23 | NM_020775 | KIAA1324 | -0.23 |
| NM_030613 | FLJ21628 | -0.23 | NM_024491 | BITE | -0.23 |
| NM_031463 | LOC83693 | -0.23 | NM_031218 | ZNF505 | -0.23 |
| NM_080653 | ATP6V1E2 | -0.23 | XM_290732 | RNF157 | -0.23 |
| NM_152335 | FLJ38190 | -0.23 | NM_139164 | STARD4 | -0.23 |
| XM_059061 | LOC126661 | -0.23 | NM_153449 | SLC2A14 | -0.23 |
| NM_178844 | NOD3 | -0.23 | NM_145018 | FLJ25416 | -0.23 |
| NM_198492 | LSECTIN | -0.23 | XM_379573 | LOC286135 | -0.23 |
| NM_199351 | C1ORF32 | -0.23 | NM_198524 | TEX9 | -0.23 |
| NM_203309 | MGC48595 | -0.23 | XM_094074 | LOC166752 | -0.23 |
| XM_379181 | LOC401057 | -0.23 | XM_379274 | LOC401151 | -0.23 |
| XM_379325 | LOC401180 | -0.23 | XM_377976 | LOC402301 | -0.23 |
| XM_373638 | LOC388142 | -0.23 | XM_373378 | LOC392559 | -0.23 |
| NM_000616 | CD4 | -0.22 | NM_004156 | PPP2CB | -0.22 |
| NM_004755 | RPS6KA5 | -0.22 | NM_053278 | TRAR5 | -0.22 |
| NM_005281 | GPR3 | -0.22 | NM_004062 | CDH16 | -0.22 |
| NM_004970 | IGFALS | -0.22 | NM_005053 | RAD23A | -0.22 |
| NM_004904 | CREB5 | -0.22 | NM_000321 | RB1 | -0.22 |
| NM_004555 | NFATC3 | -0.22 | NM_016006 | ABHD5 | -0.22 |
| NM_006877 | GMPR | -0.22 | NM_000521 | HEXB | -0.22 |
| NM_014578 | RHOD | -0.22 | NM_002534 | OAS1 | -0.22 |
| NM_003368 | USP1 | -0.22 | NM_013956 | NRG1 | -0.22 |
| NM_003463 | PTP4A1 | -0.22 | NM_001463 | FRZB | -0.22 |
| NM_000418 | IL4R | -0.22 | NM_014207 | CD5 | -0.22 |
| NM_002209 | ITGAL | -0.22 | NM_030759 | NRBF2 | -0.22 |
| NM_002607 | PDGFA | -0.22 | NM_014624 | S100A6 | -0.22 |
| NM_001852 | COL9A2 | -0.22 | NM_020989 | CRYGC | -0.22 |
| NM_003039 | SLC2A5 | -0.22 | NM_006029 | PNMA1 | -0.22 |
| NM_005645 | TAF13 | -0.22 | NM_005065 | SEL1L | -0.22 |
| NM_001676 | ATP12A | -0.22 | NM_014064 | AD-003 | -0.22 |
| NM_005600 | NIT1 | -0.22 | NM_002899 | RBP1 | -0.22 |
| NM_021977 | SLC22A3 | -0.22 | NM_004683 | RGN | -0.22 |
| NM_006038 | SPATA2 | -0.22 | NM_006064 | RRAGB | -0.22 |
| NM_006691 | XLKD1 | -0.22 | XM_039169 | KIAA1276 | -0.22 |
| XM_294533 | OR1J4 | -0.22 | NM_014186 | COMMD9 | -0.22 |
| NM_016153 | LW-1 | -0.22 | NM_017820 | FLJ20433 | -0.22 |
| NM_017786 | FLJ20366 | -0.22 | NM_018360 | CXORF15 | -0.22 |
| NM_020646 | ASCL3 | -0.22 | NM_020824 | ARHGAP21 | -0.22 |
| XM_168578 | MUC3B | -0.22 | NM_024635 | MAK10 | -0.22 |
| NM_024311 | ET | -0.22 | NM_032807 | FBXO18 | -0.22 |
| NM_145233 | ZNF625 | -0.22 | NM_033410 | MGC13138 | -0.22 |

| | | |
|---|---|---|
| NM_033213 | MGC12466 | -0.22 |
| NM_033547 | MGC16733 | -0.22 |
| NM_173505 | ANKRD29 | -0.22 |
| XM_374912 | XRRA1 | -0.22 |
| XM_379164 | LOC151171 | -0.22 |
| NM_138337 | MICL | -0.22 |
| NM_175063 | LOC284361 | -0.22 |
| XM_087490 | LOC152586 | -0.22 |
| XM_293380 | LOC347454 | -0.22 |
| XM_372815 | LOC391165 | -0.22 |
| XM_371647 | LOC389129 | -0.22 |
| XM_374295 | LOC389767 | -0.22 |
| XM_373463 | LOC387686 | -0.22 |
| NM_212552 | LOC388962 | -0.22 |
| NM_004073 | PLK3 | -0.21 |
| NM_002019 | FLT1 | -0.21 |
| NM_000905 | NPY | -0.21 |
| NM_033108 | HSFY1 | -0.21 |
| NM_004914 | RAB36 | -0.21 |
| NM_022113 | KIF13A | -0.21 |
| NM_003974 | DOK2 | -0.21 |
| NM_001645 | APOC1 | -0.21 |
| NM_014764 | DAZAP2 | -0.21 |
| NM_002824 | PTMS | -0.21 |
| NM_020978 | AMY2B | -0.21 |
| NM_018063 | HELLS | -0.21 |
| NM_002126 | HLF | -0.21 |
| NM_014057 | OGN | -0.21 |
| NM_004789 | LHX2 | -0.21 |
| NM_004853 | STX8 | -0.21 |
| NM_013448 | BAZ1A | -0.21 |
| XM_087353 | KIAA0794 | -0.21 |
| NM_013253 | DKK3 | -0.21 |
| NM_018024 | FLJ10204 | -0.21 |
| NM_019855 | CABP5 | -0.21 |
| NM_021200 | PLEKHB1 | -0.21 |
| NM_032849 | FLJ14834 | -0.21 |
| NM_052860 | ZNF300 | -0.21 |
| NM_177452 | TRAPPC6B | -0.21 |
| NM_144642 | SYNPR | -0.21 |
| NM_174909 | MGC23909 | -0.21 |
| XM_378777 | LOC284385 | -0.21 |
| NM_182634 | FLJ36166 | -0.21 |
| NM_207494 | FLJ40092 | -0.21 |
| XM_068229 | LOC133185 | -0.21 |
| XM_374062 | LOC389172 | -0.21 |
| NM_152333 | SLC25A29 | -0.22 |
| NM_173489 | FLJ40243 | -0.22 |
| NM_153040 | FLJ32831 | -0.22 |
| NM_194288 | LOC146712 | -0.22 |
| NM_173352 | K5B | -0.22 |
| XM_370696 | FLJ34236 | -0.22 |
| NM_207431 | FLJ45950 | -0.22 |
| XM_210642 | LOC285697 | -0.22 |
| XM_295195 | LOC340267 | -0.22 |
| XM_377644 | LOC401993 | -0.22 |
| XM_374752 | LOC392791 | -0.22 |
| XM_379850 | LOC402524 | -0.22 |
| NM_001005497 | OR6C75 | -0.22 |
| NM_001203 | BMPR1B | -0.21 |
| NM_006314 | CNKSR1 | -0.21 |
| NM_003137 | SRPK1 | -0.21 |
| NM_012138 | AATF | -0.21 |
| NM_000404 | GLB1 | -0.21 |
| NM_033341 | BIRC8 | -0.21 |
| NM_003853 | IL18RAP | -0.21 |
| NM_003153 | STAT6 | -0.21 |
| NM_020360 | PLSCR3 | -0.21 |
| NM_000509 | FGG | -0.21 |
| NM_022743 | SMYD3 | -0.21 |
| NM_020474 | GALNT1 | -0.21 |
| NM_012314 | KIR2DS4 | -0.21 |
| NM_002540 | ODF2 | -0.21 |
| NM_006309 | LRRFIP2 | -0.21 |
| XM_376578 | PHF14 | -0.21 |
| XM_375685 | KLHL21 | -0.21 |
| NM_015381 | TAFA5 | -0.21 |
| NM_015677 | SH3YL1 | -0.21 |
| NM_016010 | CGI-62 | -0.21 |
| NM_017969 | FLJ10006 | -0.21 |
| NM_021187 | CYP4F11 | -0.21 |
| XM_048362 | KIAA1543 | -0.21 |
| NM_138423 | H63 | -0.21 |
| NM_173469 | LOC92912 | -0.21 |
| NM_139244 | STXBP5 | -0.21 |
| NM_153031 | FLJ32063 | -0.21 |
| NM_175908 | LOC284296 | -0.21 |
| XM_379230 | LOC339894 | -0.21 |
| NM_207417 | FLJ46082 | -0.21 |
| XM_060887 | LOC128192 | -0.21 |
| XM_067369 | LOC131405 | -0.21 |
| XM_376756 | LOC401454 | -0.21 |

| | | | | | |
|---|---|---|---|---|---|
| XM_378090 | LOC402382 | -0.21 | XM_373557 | LOC387901 | -0.21 |
| XM_378365 | LOC400050 | -0.21 | XM_377537 | LOC401922 | -0.21 |
| XM_373744 | LOC388414 | -0.21 | XM_372648 | LOC390748 | -0.21 |
| XM_379030 | LOC400880 | -0.21 | XM_372268 | LOC389904 | -0.21 |
| NM_007313 | ABL1 | -0.2 | NM_004767 | GPR37L1 | -0.2 |
| NM_006759 | UGP2 | -0.2 | NM_033179 | OR51B4 | -0.2 |
| NM_004825 | CDY2 | -0.2 | NM_014292 | CBX6 | -0.2 |
| NM_018938 | PCDHB4 | -0.2 | NM_032335 | PHF6 | -0.2 |
| NM_002970 | SAT | -0.2 | NM_016525 | UBAP1 | -0.2 |
| NM_004249 | RAB28 | -0.2 | NM_001918 | DBT | -0.2 |
| NM_015277 | NEDD4L | -0.2 | NM_004667 | HERC2 | -0.2 |
| NM_033272 | KCNH7 | -0.2 | NM_016363 | GP6 | -0.2 |
| NM_005472 | KCNE3 | -0.2 | NM_007074 | CORO1A | -0.2 |
| NM_004170 | SLC1A1 | -0.2 | NM_015710 | GLTSCR2 | -0.2 |
| NM_004883 | NRG2 | -0.2 | NM_012425 | RSU1 | -0.2 |
| NM_006069 | MRVI1 | -0.2 | NM_002582 | PARN | -0.2 |
| NM_024411 | PDYN | -0.2 | NM_021095 | SLC5A6 | -0.2 |
| NM_005460 | SNCAIP | -0.2 | XM_379798 | KIAA0087 | -0.2 |
| NM_006805 | HNRPA0 | -0.2 | NM_007277 | SEC6L1 | -0.2 |
| NM_015170 | SULF1 | -0.2 | NM_012382 | OSRF | -0.2 |
| NM_015523 | DKFZP566E144 | -0.2 | NM_015361 | R3HDM | -0.2 |
| NM_020349 | ANKRD2 | -0.2 | NM_016581 | SITPEC | -0.2 |
| NM_016475 | C14ORF100 | -0.2 | NM_017723 | FLJ20245 | -0.2 |
| NM_018128 | FLJ10534 | -0.2 | NM_018318 | FLJ11088 | -0.2 |
| NM_020871 | LRCH2 | -0.2 | NM_025203 | FLJ21945 | -0.2 |
| NM_024924 | FLJ12985 | -0.2 | NM_030796 | DKFZP564K0822 | -0.2 |
| XM_372239 | LOC92129 | -0.2 | XM_085175 | TTC7B | -0.2 |
| XM_379559 | LOC157278 | -0.2 | NM_173828 | C5ORF16 | -0.2 |
| NM_173833 | MGC45780 | -0.2 | XM_379592 | LOC286144 | -0.2 |
| NM_198999 | PRES | -0.2 | XM_167908 | LOC221140 | -0.2 |
| XM_376043 | LOC400948 | -0.2 | XM_376600 | LOC401330 | -0.2 |
| XM_374735 | LOC392730 | -0.2 | NM_002822 | PTK9 | -0.19 |
| NM_000684 | ADRB1 | -0.19 | NM_006888 | CALM1 | -0.19 |
| NM_080836 | STK35 | -0.19 | NM_004248 | GPR10 | -0.19 |
| NM_147199 | MRGX1 | -0.19 | NM_021170 | HES4 | -0.19 |
| NM_022768 | RBM15 | -0.19 | NM_001755 | CBFB | -0.19 |
| NM_002918 | RFX1 | -0.19 | NM_001958 | EEF1A2 | -0.19 |
| NM_000320 | QDPR | -0.19 | XM_046751 | PPFIA4 | -0.19 |
| NM_003070 | SMARCA2 | -0.19 | NM_022139 | GFRA4 | -0.19 |
| NM_004864 | GDF15 | -0.19 | NM_003841 | TNFRSF10C | -0.19 |
| NM_139136 | KCNC2 | -0.19 | NM_004326 | BCL9 | -0.19 |
| NM_000099 | CST3 | -0.19 | NM_002231 | KAI1 | -0.19 |
| XM_037493 | SHANK3 | -0.19 | NM_005528 | DNAJC4 | -0.19 |
| NM_001931 | DLAT | -0.19 | XM_035037 | LRP4 | -0.19 |
| NM_016533 | NINJ2 | -0.19 | NM_003134 | SRP14 | -0.19 |
| NM_001062 | TCN1 | -0.19 | NM_004679 | VCY | -0.19 |

| | | | | | |
|---|---|---|---|---|---|
| NM_015080 | NRXN2 | -0.19 | NM_004818 | DDX23 | -0.19 |
| NM_014667 | VGLL4 | -0.19 | NM_006052 | DSCR3 | -0.19 |
| XM_375377 | KIAA0513 | -0.19 | NM_006373 | VAT1 | -0.19 |
| NM_007078 | LDB3 | -0.19 | NM_006704 | SUGT1 | -0.19 |
| NM_014284 | NCDN | -0.19 | NM_013446 | MKRN1 | -0.19 |
| XM_290511 | ZDHHC5 | -0.19 | NM_014070 | C6ORF15 | -0.19 |
| NM_016331 | ANC_2H01 | -0.19 | NM_016061 | CGI-127 | -0.19 |
| NM_018364 | FLJ11220 | -0.19 | XM_371614 | FLJ10707 | -0.19 |
| NM_018553 | HSA277841 | -0.19 | NM_018277 | C21ORF77 | -0.19 |
| NM_031277 | RNF17 | -0.19 | NM_022978 | SERF1B | -0.19 |
| NM_021211 | LOC58486 | -0.19 | NM_021938 | BRUNOL5 | -0.19 |
| NM_022371 | TOR3A | -0.19 | NM_024054 | C7ORF25 | -0.19 |
| NM_024871 | FLJ12748 | -0.19 | NM_025219 | DNAJC5 | -0.19 |
| NM_053279 | C8ORF13 | -0.19 | NM_015059 | TLN2 | -0.19 |
| NM_052941 | GBP4 | -0.19 | NM_173353 | TPH2 | -0.19 |
| XM_050564 | MGC20533 | -0.19 | NM_145272 | LOC146853 | -0.19 |
| NM_152599 | FLJ35773 | -0.19 | XM_379198 | LOC285286 | -0.19 |
| NM_175920 | FLJ39485 | -0.19 | NM_182577 | C19ORF19 | -0.19 |
| NM_203302 | MGC70863 | -0.19 | NM_207468 | FLJ43505 | -0.19 |
| XM_291548 | LOC343406 | -0.19 | XM_290780 | LOC339237 | -0.19 |
| XM_372780 | LOC391045 | -0.19 | XM_374002 | LOC389023 | -0.19 |
| XM_371480 | LOC388929 | -0.19 | XM_372919 | LOC391356 | -0.19 |
| XM_374152 | LOC389353 | -0.19 | XM_379625 | LOC401495 | -0.19 |
| XM_351854 | LOC389797 | -0.19 | XM_373466 | LOC387690 | -0.19 |
| XM_372497 | LOC390426 | -0.19 | XM_372682 | LOC390816 | -0.19 |
| NM_001004331 | MGC88374 | -0.19 | NM_001005517 | OR5K4 | -0.19 |
| NM_022833 | C9ORF88 | -0.19 | NM_014011 | SOCS5 | -0.18 |
| NM_003505 | FZD1 | -0.18 | NM_015690 | STK36 | -0.18 |
| NM_178129 | P2RY8 | -0.18 | NM_032777 | GPR124 | -0.18 |
| NM_022304 | HRH2 | -0.18 | NM_014504 | RABGEF1 | -0.18 |
| NM_014048 | MRTF-B | -0.18 | NM_003016 | SFRS2 | -0.18 |
| NM_003616 | SIP1 | -0.18 | NM_003898 | SYNJ2 | -0.18 |
| NM_024960 | PANK2 | -0.18 | NM_000602 | SERPINE1 | -0.18 |
| XM_047871 | DXS1283E | -0.18 | NM_000250 | MPO | -0.18 |
| NM_006519 | TCTEL1 | -0.18 | NM_003502 | AXIN1 | -0.18 |
| BC065228 | PSPHL | -0.18 | NM_003930 | SCAP2 | -0.18 |
| NM_003921 | BCL10 | -0.18 | NM_002308 | LGALS9 | -0.18 |
| NM_005529 | HSPG2 | -0.18 | NM_007278 | GABARAP | -0.18 |
| NM_005502 | ABCA1 | -0.18 | NM_032282 | DKFZP547D155 | -0.18 |
| NM_020396 | BCL2L10 | -0.18 | NM_001001395 | LMO3 | -0.18 |
| NM_021999 | ITM2B | -0.18 | NM_004447 | EPS8 | -0.18 |
| NM_006545 | TUSC4 | -0.18 | XM_039393 | PLXNA4 | -0.18 |
| NM_003579 | RAD54L | -0.18 | NM_000724 | CACNB2 | -0.18 |
| NM_004915 | ABCG1 | -0.18 | NM_015599 | PGM3 | -0.18 |
| NM_014049 | ACAD9 | -0.18 | NM_006714 | SMPDL3A | -0.18 |
| NM_147200 | C6ORF4 | -0.18 | NM_015379 | BRI3 | -0.18 |

| | | | | | |
|---|---|---|---|---|---|
| NM_015527 | DKFZP434P1750 | -0.18 | NM_012200 | B3GAT3 | -0.18 |
| NM_015703 | CGI-96 | -0.18 | NM_012131 | CLDN17 | -0.18 |
| NM_016042 | EXOSC3 | -0.18 | NM_016352 | CPA4 | -0.18 |
| NM_016105 | FKBP7 | -0.18 | NM_016199 | LSM7 | -0.18 |
| NM_017583 | TRIM44 | -0.18 | XM_376664 | KIAA1218 | -0.18 |
| NM_145214 | TRIM11 | -0.18 | NM_030806 | C1ORF21 | -0.18 |
| NM_054028 | AMAC | -0.18 | NM_032346 | MGC13096 | -0.18 |
| NM_053046 | EGLN2 | -0.18 | NM_052878 | IMAGE:4215339 | -0.18 |
| NM_178123 | SESTD1 | -0.18 | NM_138777 | MRRF | -0.18 |
| NM_052964 | MIST | -0.18 | NM_152325 | MGC40178 | -0.18 |
| XM_058879 | LOC124976 | -0.18 | NM_145257 | LOC126731 | -0.18 |
| NM_173502 | FLJ90661 | -0.18 | NM_173508 | SLC35F3 | -0.18 |
| NM_153365 | FLJ90013 | -0.18 | NM_173802 | MGC50559 | -0.18 |
| NM_198484 | ZNF621 | -0.18 | NM_182975 | FLJ20403 | -0.18 |
| NM_182574 | FLJ36070 | -0.18 | XM_295017 | C21ORF54 | -0.18 |
| NM_173690 | C9ORF126 | -0.18 | NM_198546 | LOC374955 | -0.18 |
| NM_198857 | FLJ43855 | -0.18 | NM_207433 | FLJ44874 | -0.18 |
| NM_003520 | HIST1H2BN | -0.18 | XM_210411 | LOC285101 | -0.18 |
| XM_208320 | LOC285442 | -0.18 | NM_001005209 | MGC99813 | -0.18 |
| XM_295261 | LOC340511 | -0.18 | XM_373858 | LOC388667 | -0.18 |
| XM_373979 | LOC388938 | -0.18 | XM_379276 | LOC401155 | -0.18 |
| XM_373036 | LOC391707 | -0.18 | XM_376587 | LOC401316 | -0.18 |
| XM_373468 | LOC387697 | -0.18 | XM_373446 | LOC387632 | -0.18 |
| XM_373763 | LOC388436 | -0.18 | XM_373930 | LOC388820 | -0.18 |
| NM_001004473 | OR10K1 | -0.18 | NM_005913 | MC5R | -0.17 |
| NM_138445 | GPR146 | -0.17 | NM_004664 | LIN7A | -0.17 |
| NM_006527 | SLBP | -0.17 | NM_007109 | TCF19 | -0.17 |
| NM_002657 | PLAGL2 | -0.17 | NM_021804 | ACE2 | -0.17 |
| NM_003758 | EIF3S1 | -0.17 | NM_002600 | PDE4B | -0.17 |
| NM_001072 | UGT1A6 | -0.17 | NM_005575 | LNPEP | -0.17 |
| NM_002574 | PRDX1 | -0.17 | NM_001380 | DOCK1 | -0.17 |
| NM_000104 | CYP1B1 | -0.17 | NM_003940 | USP13 | -0.17 |
| NM_022781 | RNF38 | -0.17 | NM_003939 | BTRC | -0.17 |
| NM_001099 | ACPP | -0.17 | NM_003580 | NSMAF | -0.17 |
| NM_005749 | TOB1 | -0.17 | NM_001174 | ARHGAP6 | -0.17 |
| NM_001102 | ACTN1 | -0.17 | NM_000093 | COL5A1 | -0.17 |
| NM_004389 | CTNNA2 | -0.17 | NM_012261 | C20ORF103 | -0.17 |
| NM_000519 | HBD | -0.17 | NM_207517 | ADAMTSL3 | -0.17 |
| NM_006767 | LZTR1 | -0.17 | NM_000379 | XDH | -0.17 |
| NM_005490 | SH2D3A | -0.17 | NM_006514 | SCN10A | -0.17 |
| NM_012313 | KIR2DS3 | -0.17 | NM_002261 | KLRC3 | -0.17 |
| NM_002525 | NRD1 | -0.17 | NM_005935 | MLLT2 | -0.17 |
| NM_002864 | PZP | -0.17 | NM_201563 | FCGR2C | -0.17 |
| NM_014677 | RIMS2 | -0.17 | NM_014805 | EPM2AIP1 | -0.17 |
| XM_047357 | KIAA0342 | -0.17 | NM_014753 | BMS1L | -0.17 |
| NM_015027 | KIAA0251 | -0.17 | NM_015684 | ATP5S | -0.17 |

| | | | | | |
|---|---|---|---|---|---|
| NM_019003 | SPIN2 | -0.17 | NM_017772 | C6ORF197 | -0.17 |
| NM_020153 | FLJ21827 | -0.17 | XM_035497 | KIAA1602 | -0.17 |
| NM_145664 | SPANXB2 | -0.17 | NM_024069 | MGC2749 | -0.17 |
| NM_024719 | GRTP1 | -0.17 | NM_024116 | MGC5306 | -0.17 |
| XM_378723 | FLJ22659 | -0.17 | NM_032207 | FLJ21742 | -0.17 |
| NM_032358 | MGC13183 | -0.17 | NM_032732 | IL17RC | -0.17 |
| NM_080860 | TSGA2 | -0.17 | NM_033345 | RGS8 | -0.17 |
| NM_052889 | COP | -0.17 | NM_080605 | B3GALT6 | -0.17 |
| XM_086095 | LOC148203 | -0.17 | NM_144727 | CRYGN | -0.17 |
| NM_152495 | FLJ38993 | -0.17 | NM_144725 | FLJ25439 | -0.17 |
| NM_198946 | LCN6 | -0.17 | NM_015338 | ASXL1 | -0.17 |
| NM_152608 | FLJ35382 | -0.17 | NM_173548 | ZNF584 | -0.17 |
| NM_152706 | MGC26647 | -0.17 | NM_170685 | TAC4 | -0.17 |
| XM_375713 | LOC284551 | -0.17 | XM_211843 | LOC285326 | -0.17 |
| XM_169434 | LOC219414 | -0.17 | XM_290743 | LOC339184 | -0.17 |
| XM_379189 | LOC401061 | -0.17 | XM_371561 | LOC389039 | -0.17 |
| XM_372004 | LOC389602 | -0.17 | XM_374121 | LOC389290 | -0.17 |
| XM_374236 | LOC389562 | -0.17 | XM_379526 | LOC401410 | -0.17 |
| XM_374401 | LOC392631 | -0.17 | XM_373469 | LOC387701 | -0.17 |
| XM_374325 | LOC389846 | -0.17 | NM_138316 | PANK1 | -0.16 |
| NM_000840 | GRM3 | -0.16 | NM_020649 | CBX8 | -0.16 |
| NM_017409 | HOXC10 | -0.16 | NM_005169 | PHOX2A | -0.16 |
| NM_003924 | PHOX2B | -0.16 | NM_005612 | REST | -0.16 |
| NM_004498 | ONECUT1 | -0.16 | NM_019102 | HOXA5 | -0.16 |
| NM_024794 | ABHD9 | -0.16 | NM_006294 | UQCRB | -0.16 |
| NM_002528 | NTHL1 | -0.16 | NM_000178 | GSS | -0.16 |
| NM_002602 | PDE6G | -0.16 | NM_003652 | CPZ | -0.16 |
| NM_001136 | AGER | -0.16 | NM_000915 | OXT | -0.16 |
| NM_032902 | PPP1R16A | -0.16 | NM_001789 | CDC25A | -0.16 |
| NM_000600 | IL6 | -0.16 | NM_000595 | LTA | -0.16 |
| NM_014166 | VDRIP | -0.16 | NM_001643 | APOA2 | -0.16 |
| NM_001658 | ARF1 | -0.16 | NM_024596 | FLJ12847 | -0.16 |
| NM_014422 | PIB5PA | -0.16 | NM_006439 | MAB21L2 | -0.16 |
| NM_080822 | OVCA2 | -0.16 | NM_004074 | COX8A | -0.16 |
| NM_001312 | CRIP2 | -0.16 | NM_021010 | DEFA5 | -0.16 |
| NM_006934 | SLC6A9 | -0.16 | NM_001077 | UGT2B17 | -0.16 |
| NM_003526 | HIST1H2BC | -0.16 | NM_006100 | SIAT10 | -0.16 |
| NM_006425 | SLU7 | -0.16 | NM_007356 | LAMB4 | -0.16 |
| NM_014001 | GGA3 | -0.16 | NM_015024 | XPO7 | -0.16 |
| NM_014614 | PSME4 | -0.16 | XM_291064 | KIAA0540 | -0.16 |
| NM_015496 | DKFZP434I116 | -0.16 | NM_021115 | SEZ6L | -0.16 |
| XM_044196 | DKFZP434C212 | -0.16 | NM_014460 | PIPPIN | -0.16 |
| NM_014170 | HSPC135 | -0.16 | NM_013319 | TERE1 | -0.16 |
| NM_152745 | NXPH1 | -0.16 | NM_018920 | PCDHGA7 | -0.16 |
| NM_020125 | SLAMF8 | -0.16 | NM_018330 | KIAA1598 | -0.16 |
| NM_024789 | C10ORF77 | -0.16 | NM_024682 | TBC1D17 | -0.16 |

| | | | | | |
|---|---|---|---|---|---|
| NM_033309 | MGC4655 | -0.16 | XM_056434 | TTC6 | -0.16 |
| NM_052900 | CSMD3 | -0.16 | NM_152355 | ZNF441 | -0.16 |
| NM_194317 | MGC52057 | -0.16 | NM_138802 | LOC130617 | -0.16 |
| XM_059074 | LOC126755 | -0.16 | NM_152450 | MGC26690 | -0.16 |
| XM_375359 | BEAN | -0.16 | NM_138813 | ATP8B3 | -0.16 |
| NM_173513 | MGC43122 | -0.16 | NM_182536 | GDDR | -0.16 |
| NM_153255 | MCMDC1 | -0.16 | NM_152764 | MGC35212 | -0.16 |
| XM_374257 | LOC286114 | -0.16 | NM_005127 | CLECSF2 | -0.16 |
| NM_207435 | FLJ40142 | -0.16 | XM_292724 | LOC342934 | -0.16 |
| XM_211988 | LOC285711 | -0.16 | XM_084445 | LOC143158 | -0.16 |
| XM_372757 | LOC390992 | -0.16 | XM_374006 | LOC389028 | -0.16 |
| XM_373773 | LOC388461 | -0.16 | XM_375307 | LOC400508 | -0.16 |
| XM_372639 | LOC390732 | -0.16 | XM_371411 | LOC388819 | -0.16 |
| NM_002005 | FES | -0.15 | NM_020666 | CLK4 | -0.15 |
| NM_022150 | C7ORF9 | -0.15 | NM_001506 | GPR32 | -0.15 |
| NM_002383 | MAZ | -0.15 | NM_012311 | KIN | -0.15 |
| NM_016157 | TRO | -0.15 | NM_007128 | VPREB1 | -0.15 |
| NM_000541 | SAG | -0.15 | NM_001989 | EVX1 | -0.15 |
| NM_001497 | B4GALT1 | -0.15 | NM_000537 | REN | -0.15 |
| NM_007332 | TRPA1 | -0.15 | NM_005857 | ZMPSTE24 | -0.15 |
| NM_005349 | RBPSUH | -0.15 | NM_001001549 | GRB10 | -0.15 |
| NM_000827 | GRIA1 | -0.15 | NM_002088 | GRIK5 | -0.15 |
| NM_000212 | ITGB3 | -0.15 | NM_002305 | LGALS1 | -0.15 |
| NM_000385 | AQP1 | -0.15 | NM_021007 | SCN2A2 | -0.15 |
| NM_004976 | KCNC1 | -0.15 | NM_012471 | TRPC5 | -0.15 |
| NM_015595 | SGEF | -0.15 | NM_033043 | CGB5 | -0.15 |
| NM_002064 | GLRX | -0.15 | NM_000889 | ITGB7 | -0.15 |
| NM_024599 | RHBDL6 | -0.15 | NM_023934 | HCBP6 | -0.15 |
| NM_033415 | MGC19595 | -0.15 | NM_018403 | HSA275986 | -0.15 |
| NM_000447 | PSEN2 | -0.15 | NM_012432 | SETDB1 | -0.15 |
| NM_000662 | NAT1 | -0.15 | NM_001687 | ATP5D | -0.15 |
| NM_005005 | NDUFB9 | -0.15 | NM_006403 | NEDD9 | -0.15 |
| NM_033224 | PURB | -0.15 | NM_007370 | RFC5 | -0.15 |
| NM_003063 | SLN | -0.15 | NM_004644 | AP3B2 | -0.15 |
| NM_145911 | ZNF23 | -0.15 | NM_021100 | NFS1 | -0.15 |
| XM_376764 | PNMA2 | -0.15 | NM_015463 | C2ORF32 | -0.15 |
| XM_291253 | KIAA0146 | -0.15 | NM_016449 | LOC51233 | -0.15 |
| NM_018191 | RCBTB1 | -0.15 | NM_022356 | LEPRE1 | -0.15 |
| NM_022494 | ZDHHC6 | -0.15 | NM_022841 | FLJ12994 | -0.15 |
| NM_024844 | PCNT1 | -0.15 | NM_032139 | ANKRD27 | -0.15 |
| NM_032412 | ORF1-FL49 | -0.15 | NM_032492 | GL009 | -0.15 |
| NM_152350 | MGC40157 | -0.15 | XM_370871 | LOC145837 | -0.15 |
| NM_178496 | LOC151963 | -0.15 | NM_207332 | LOC157697 | -0.15 |
| NM_173523 | MAGEB6 | -0.15 | XM_376474 | LOC166994 | -0.15 |
| NM_138294 | PATE | -0.15 | NM_182830 | MAMDC1 | -0.15 |
| NM_194300 | LOC223075 | -0.15 | NM_182904 | P4HA3 | -0.15 |

| | | | | | |
|---|---|---|---|---|---|
| NM_152768 | FLJ25378 | -0.15 | NM_153757 | NAP1L5 | -0.15 |
| XM_379119 | LOC285000 | -0.15 | NM_175910 | ZNF493 | -0.15 |
| XM_371632 | FLJ36157 | -0.15 | NM_182973 | TMPRSS9 | -0.15 |
| XM_291321 | LOC340527 | -0.15 | XM_209913 | LOC286140 | -0.15 |
| XM_371500 | LOC388960 | -0.15 | XM_377831 | LOC402165 | -0.15 |
| XM_380047 | LOC402697 | -0.15 | XM_373495 | LOC387761 | -0.15 |
| XM_372522 | LOC390468 | -0.15 | XM_378735 | LOC400642 | -0.15 |
| XM_371134 | LOC388503 | -0.15 | NM_032630 | CINP | -0.14 |
| NM_001220 | CAMK2B | -0.14 | NM_015985 | ANGPT4 | -0.14 |
| NM_031916 | ASP | -0.14 | NM_006475 | POSTN | -0.14 |
| NM_172250 | MMAA | -0.14 | NM_000442 | PECAM1 | -0.14 |
| NM_001973 | ELK4 | -0.14 | NM_016270 | KLF2 | -0.14 |
| NM_003111 | SP3 | -0.14 | NM_003722 | TP73L | -0.14 |
| NM_021809 | TGIF2 | -0.14 | NM_014172 | PHPT1 | -0.14 |
| NM_015256 | ACSL6 | -0.14 | NM_001225 | CASP4 | -0.14 |
| NM_012161 | FBXL5 | -0.14 | NM_003015 | SFRP5 | -0.14 |
| NM_000614 | CNTF | -0.14 | NM_000742 | CHRNA2 | -0.14 |
| NM_004452 | ESRRB | -0.14 | NM_000435 | NOTCH3 | -0.14 |
| NM_018098 | ECT2 | -0.14 | NM_000090 | COL3A1 | -0.14 |
| NM_000891 | KCNJ2 | -0.14 | NM_000719 | CACNA1C | -0.14 |
| NM_000720 | CACNA1D | -0.14 | NM_012434 | SLC17A5 | -0.14 |
| NM_024492 | LPAL2 | -0.14 | NM_018896 | CACNA1G | -0.14 |
| NM_001951 | E2F5 | -0.14 | NM_012211 | ITGA11 | -0.14 |
| NM_020142 | LOC56901 | -0.14 | NM_005532 | IFI27 | -0.14 |
| NM_012333 | MYCBP | -0.14 | NM_005380 | NBL1 | -0.14 |
| NM_181782 | NCOA7 | -0.14 | NM_002243 | KCNJ15 | -0.14 |
| NM_002544 | OMG | -0.14 | NM_002371 | MAL | -0.14 |
| NM_003545 | HIST1H4E | -0.14 | XM_085596 | ZNF18 | -0.14 |
| NM_003622 | PPFIBP1 | -0.14 | XM_378182 | CHD1L | -0.14 |
| NM_014652 | IPO13 | -0.14 | NM_005847 | SLC23A1 | -0.14 |
| NM_006424 | SLC34A2 | -0.14 | XM_034904 | KIAA0912 | -0.14 |
| NM_015094 | HIC2 | -0.14 | XM_041018 | KIAA0367 | -0.14 |
| NM_014966 | DHX30 | -0.14 | NM_014038 | BZW2 | -0.14 |
| NM_016176 | CAB45 | -0.14 | NM_019027 | FLJ20273 | -0.14 |
| NM_017634 | KCTD9 | -0.14 | NM_017653 | FLJ20071 | -0.14 |
| NM_018385 | FLJ11301 | -0.14 | NM_018480 | HT007 | -0.14 |
| NM_018655 | LENEP | -0.14 | NM_020223 | FAM20C | -0.14 |
| NM_022742 | DKFZP434G156 | -0.14 | NM_022905 | FLJ12572 | -0.14 |
| NM_032647 | MGC10561 | -0.14 | NM_032681 | TRIM51 | -0.14 |
| XM_058964 | LOC89887 | -0.14 | NM_033122 | NYD-SP26 | -0.14 |
| NM_138787 | LOC119710 | -0.14 | NM_152394 | MGC39662 | -0.14 |
| NM_152439 | VMD2L3 | -0.14 | NM_144670 | FLJ25179 | -0.14 |
| NM_152600 | ZNF579 | -0.14 | NM_152652 | ZNF553 | -0.14 |
| NM_152729 | NT5C2L1 | -0.14 | NM_153045 | C9ORF91 | -0.14 |
| NM_153691 | FLJ90036 | -0.14 | XM_372528 | FLJ36749 | -0.14 |
| XM_379690 | FAM41C | -0.14 | NM_198082 | LOC284001 | -0.14 |

| | | | | | |
|---|---|---|---|---|---|
| NM_172367 | TUSC5 | -0.14 | NM_198153 | TLT4 | -0.14 |
| NM_198494 | FLJ16030 | -0.14 | XM_379700 | LOC286442 | -0.14 |
| XM_292012 | LOC341315 | -0.14 | XM_088680 | LOC158813 | -0.14 |
| XM_371555 | LOC389031 | -0.14 | XM_373890 | LOC388746 | -0.14 |
| XM_372125 | LOC389768 | -0.14 | XM_376852 | LOC401516 | -0.14 |
| XM_370634 | LOC387781 | -0.14 | XM_372651 | LOC390754 | -0.14 |
| NM_001001657 | OR2Y1 | -0.14 | XM_373952 | LOC388889 | -0.14 |
| NM_006738 | AKAP13 | -0.13 | NM_002732 | PRKACG | -0.13 |
| NM_005028 | PIP5K2A | -0.13 | NM_006826 | YWHAQ | -0.13 |
| NM_004983 | KCNJ9 | -0.13 | NM_006135 | CAPZA1 | -0.13 |
| NM_000599 | IGFBP5 | -0.13 | NM_153686 | MLR1 | -0.13 |
| NM_002097 | GTF3A | -0.13 | NM_003325 | HIRA | -0.13 |
| NM_006621 | AHCYL1 | -0.13 | NM_003206 | TCF21 | -0.13 |
| NM_005418 | ST5 | -0.13 | NM_014508 | APOBEC3C | -0.13 |
| NM_002177 | IFNW1 | -0.13 | NM_003889 | NR1I2 | -0.13 |
| NM_001988 | EVPL | -0.13 | NM_005419 | STAT2 | -0.13 |
| NM_000218 | KCNQ1 | -0.13 | NM_002704 | PPBP | -0.13 |
| NM_021960 | MCL1 | -0.13 | NM_003632 | CNTNAP1 | -0.13 |
| NM_004804 | CIAO1 | -0.13 | NM_013324 | CISH | -0.13 |
| NM_001763 | CD1A | -0.13 | BC069763 | C2orf12 | -0.13 |
| NM_031474 | NRIP2 | -0.13 | NM_002152 | HRC | -0.13 |
| NM_001531 | MR1 | -0.13 | NM_002143 | HPCA | -0.13 |
| NM_002361 | MAG | -0.13 | NM_000607 | ORM1 | -0.13 |
| NM_002491 | NDUFB3 | -0.13 | NM_014229 | SLC6A11 | -0.13 |
| NM_013359 | ZNF221 | -0.13 | NM_022719 | DGCR14 | -0.13 |
| NM_021015 | SSX5 | -0.13 | NM_004676 | PRY | -0.13 |
| NM_006418 | GW112 | -0.13 | NM_024330 | SLC27A3 | -0.13 |
| NM_006668 | CYP46A1 | -0.13 | XM_375471 | KIAA0924 | -0.13 |
| NM_013264 | DDX25 | -0.13 | NM_013442 | STOML2 | -0.13 |
| NM_016347 | CML2 | -0.13 | NM_017874 | C20ORF27 | -0.13 |
| NM_018188 | ATAD3A | -0.13 | NM_018569 | PRO0971 | -0.13 |
| NM_018463 | MDS028 | -0.13 | NM_020353 | PLSCR4 | -0.13 |
| XM_375557 | C19ORF29 | -0.13 | NM_021932 | RIC-8 | -0.13 |
| NM_024658 | IPO4 | -0.13 | NM_024681 | FLJ12242 | -0.13 |
| NM_032880 | MGC15730 | -0.13 | XM_071712 | LOC120376 | -0.13 |
| NM_145250 | C14ORF6 | -0.13 | NM_133452 | RAVER1 | -0.13 |
| NM_152367 | FLJ38716 | -0.13 | NM_145048 | MGC29898 | -0.13 |
| NM_144662 | MGC26605 | -0.13 | XM_378558 | LOC146443 | -0.13 |
| NM_152552 | SAMD3 | -0.13 | NM_144981 | FLJ25059 | -0.13 |
| NM_152758 | YTHDF3 | -0.13 | NM_182559 | MGC57341 | -0.13 |
| NM_173657 | FLJ31139 | -0.13 | XM_378858 | LOC339539 | -0.13 |
| NM_207354 | LOC338692 | -0.13 | XM_375606 | DKFZP779O175 | -0.13 |
| NM_207503 | FLJ42280 | -0.13 | XM_291577 | LOC339521 | -0.13 |
| XM_088686 | LOC158825 | -0.13 | XM_294387 | LOC340359 | -0.13 |
| XM_373838 | LOC388617 | -0.13 | XM_379849 | LOC402522 | -0.13 |
| XM_371157 | LOC388526 | -0.13 | NM_018238 | FLJ10842 | -0.12 |

| | | | | | |
|---|---|---|---|---|---|
| NM_000476 | AK1 | -0.12 | NM_004566 | PFKFB3 | -0.12 |
| NM_003258 | TK1 | -0.12 | NM_022096 | ANKRD5 | -0.12 |
| NM_173856 | VN1R2 | -0.12 | NM_005461 | MAFB | -0.12 |
| NM_016544 | RBJ | -0.12 | NM_002163 | ICSBP1 | -0.12 |
| NM_002539 | ODC1 | -0.12 | XM_290331 | GGT2 | -0.12 |
| NM_025104 | DRF1 | -0.12 | NM_005915 | MCM6 | -0.12 |
| NM_003117 | SPAM1 | -0.12 | NM_012180 | FBXO8 | -0.12 |
| NM_004964 | HDAC1 | -0.12 | NM_005861 | STUB1 | -0.12 |
| NM_012276 | ILT7 | -0.12 | NM_005408 | CCL13 | -0.12 |
| NM_006664 | CCL27 | -0.12 | NM_007179 | INSL6 | -0.12 |
| NM_080387 | CLECSF8 | -0.12 | NM_001489 | NR6A1 | -0.12 |
| NM_004114 | FGF13 | -0.12 | NM_003235 | TG | -0.12 |
| NM_014191 | SCN8A | -0.12 | NM_004749 | TBRG4 | -0.12 |
| NM_003074 | SMARCC1 | -0.12 | NM_001487 | BLOC1S1 | -0.12 |
| NM_006031 | PCNT2 | -0.12 | NM_002902 | RCN2 | -0.12 |
| NM_005632 | SOLH | -0.12 | NM_021160 | BAT5 | -0.12 |
| NM_005747 | ELA3A | -0.12 | NM_014849 | SV2A | -0.12 |
| NM_021267 | LASS1 | -0.12 | NM_006558 | KHDRBS3 | -0.12 |
| XM_376903 | KIAA0674 | -0.12 | NM_015352 | POFUT1 | -0.12 |
| NM_016354 | SLCO4A1 | -0.12 | NM_013290 | HUMGT198A | -0.12 |
| NM_013335 | GMPPA | -0.12 | NM_017922 | PRPF39 | -0.12 |
| NM_017639 | CDHJ | -0.12 | NM_017729 | EPS8L1 | -0.12 |
| NM_018248 | FLJ10858 | -0.12 | NM_022840 | METTL4 | -0.12 |
| NM_024988 | FLJ12355 | -0.12 | NM_030570 | UPK3B | -0.12 |
| NM_024869 | FLJ14050 | -0.12 | NM_030633 | KIAA1712 | -0.12 |
| NM_025201 | PP9099 | -0.12 | NM_032222 | FLJ22374 | -0.12 |
| NM_032226 | ZCCHC7 | -0.12 | NM_201517 | H2AFV | -0.12 |
| NM_138385 | LOC92305 | -0.12 | NM_194326 | MGC52010 | -0.12 |
| XM_351317 | LEMD1 | -0.12 | NM_152322 | BTBD11 | -0.12 |
| NM_178229 | IQGAP3 | -0.12 | NM_145050 | MGC27434 | -0.12 |
| NM_080621 | C20ORF136 | -0.12 | NM_144712 | SLC23A3 | -0.12 |
| XM_370785 | C14ORF81 | -0.12 | NM_138330 | TIZ | -0.12 |
| NM_145064 | STAC3 | -0.12 | XM_379684 | LOC286238 | -0.12 |
| XM_292740 | LOC342892 | -0.12 | NM_198180 | P518 | -0.12 |
| NM_207386 | FLJ45455 | -0.12 | XM_292197 | LOC341651 | -0.12 |
| XM_099034 | LOC159170 | -0.12 | XM_208554 | LOC283226 | -0.12 |
| XM_373831 | LOC388604 | -0.12 | XM_379489 | LOC401345 | -0.12 |
| XM_371770 | LOC389322 | -0.12 | XM_376643 | LOC401383 | -0.12 |
| XM_374371 | LOC392177 | -0.12 | XM_373238 | LOC392188 | -0.12 |
| XM_372498 | LOC390427 | -0.12 | XM_378381 | LOC400070 | -0.12 |
| XM_373682 | LOC388270 | -0.12 | XM_371177 | LOC388551 | -0.12 |
| XM_373666 | LOC388214 | -0.12 | XM_373670 | LOC388227 | -0.12 |
| NM_001001412 | FAM26C | -0.12 | NM_001005280 | OR10A7 | -0.12 |
| XM_373970 | LOC388917 | -0.12 | NM_000292 | PHKA2 | -0.11 |
| NM_153840 | GPR110 | -0.11 | NM_000584 | IL8 | -0.11 |
| NM_012418 | FSCN2 | -0.11 | NM_020227 | PRDM9 | -0.11 |

| | | | | | |
|---|---|---|---|---|---|
| NM_016260 | ZNFN1A2 | -0.11 | NM_007011 | ABHD2 | -0.11 |
| NM_004413 | DPEP1 | -0.11 | NM_145740 | GSTA1 | -0.11 |
| NM_000015 | NAT2 | -0.11 | NM_001665 | RHOG | -0.11 |
| NM_004666 | VNN1 | -0.11 | NM_005706 | TSSC4 | -0.11 |
| NM_014080 | DUOX2 | -0.11 | NM_000504 | F10 | -0.11 |
| NM_000029 | AGT | -0.11 | NM_005067 | SIAH2 | -0.11 |
| NM_181790 | GPR142 | -0.11 | NM_003393 | WNT8B | -0.11 |
| NM_003247 | THBS2 | -0.11 | NM_006931 | SLC2A3 | -0.11 |
| NM_033364 | AAT1 | -0.11 | NM_004996 | ABCC1 | -0.11 |
| NM_000718 | CACNA1B | -0.11 | NM_014944 | CLSTN1 | -0.11 |
| NM_002017 | FLI1 | -0.11 | NM_006547 | IMP-3 | -0.11 |
| NM_015069 | ZNF423 | -0.11 | NM_006819 | STIP1 | -0.11 |
| NM_017460 | CYP3A4 | -0.11 | NM_002370 | MAGOH | -0.11 |
| NM_004164 | RBP2 | -0.11 | NM_002972 | SBF1 | -0.11 |
| NM_003009 | SEPW1 | -0.11 | NM_003452 | ZNF189 | -0.11 |
| NM_004199 | P4HA2 | -0.11 | NM_006364 | SEC23A | -0.11 |
| NM_006635 | ZNF272 | -0.11 | NM_181873 | CRA | -0.11 |
| NM_007046 | EMILIN1 | -0.11 | NM_012307 | EPB41L3 | -0.11 |
| NM_015515 | KRT23 | -0.11 | NM_015600 | C20ORF22 | -0.11 |
| NM_014435 | ASAHL | -0.11 | NM_016075 | C13ORF9 | -0.11 |
| NM_016575 | TU12B1-TY | -0.11 | NM_018259 | TTC17 | -0.11 |
| NM_017865 | FLJ20531 | -0.11 | NM_018396 | METTL2 | -0.11 |
| XM_047995 | ODZ2 | -0.11 | NM_020773 | TBC1D14 | -0.11 |
| NM_024082 | TMG3 | -0.11 | NM_024516 | MGC4606 | -0.11 |
| NM_024098 | MGC2574 | -0.11 | NM_024544 | FLJ12875 | -0.11 |
| XM_379403 | C6ORF12 | -0.11 | NM_024901 | FLJ22457 | -0.11 |
| NM_032484 | LGP1 | -0.11 | NM_032327 | ZDHHC16 | -0.11 |
| NM_032866 | FLJ14957 | -0.11 | XM_372210 | PPP1R3F | -0.11 |
| NM_033557 | LOC90522 | -0.11 | NM_138785 | C6ORF72 | -0.11 |
| NM_144675 | MGC18079 | -0.11 | NM_138471 | LOC144097 | -0.11 |
| NM_207334 | FLJ44952 | -0.11 | XM_114087 | KIAA1836 | -0.11 |
| NM_174924 | LOC204474 | -0.11 | NM_147197 | WFDC11 | -0.11 |
| NM_203376 | LOC388730 | -0.11 | NM_173604 | FLJ25694 | -0.11 |
| XM_375313 | MGC9515 | -0.11 | NM_199163 | FLJ25222 | -0.11 |
| NM_212553 | LOC391622 | -0.11 | NM_207461 | FLJ44881 | -0.11 |
| XM_059037 | LOC126374 | -0.11 | XM_097729 | LOC149704 | -0.11 |
| XM_298053 | LOC347487 | -0.11 | XM_379079 | LOC400946 | -0.11 |
| XM_372954 | LOC391429 | -0.11 | XM_371771 | LOC389323 | -0.11 |
| XM_379299 | LOC401165 | -0.11 | XM_380007 | LOC402663 | -0.11 |
| XM_374904 | LOC399921 | -0.11 | XM_370965 | LOC388242 | -0.11 |
| XM_373788 | LOC388494 | -0.11 | NM_001001703 | FLJ46010 | -0.11 |
| XM_373962 | LOC388903 | -0.11 | NM_153220 | MGC35440 | -0.11 |
| NM_022784 | FLJ12476 | -0.1 | NM_005781 | ACK1 | -0.1 |
| NM_014572 | LATS2 | -0.1 | NM_013233 | STK39 | -0.1 |
| NM_002075 | GNB3 | -0.1 | NM_019858 | GRCA | -0.1 |
| NM_012351 | OR10J1 | -0.1 | NM_003382 | VIPR2 | -0.1 |

| | | | | | |
|---|---|---|---|---|---|
| NM_004365 | CETN3 | -0.1 | NM_006822 | RAB40B | -0.1 |
| NM_003735 | PCDHGA12 | -0.1 | NM_005701 | RNUT1 | -0.1 |
| NM_003443 | ZNF151 | -0.1 | NM_000488 | SERPINC1 | -0.1 |
| NM_181872 | DMRT2 | -0.1 | NM_003792 | EDF1 | -0.1 |
| NM_004239 | TRIP11 | -0.1 | NM_000161 | GCH1 | -0.1 |
| NM_005026 | PIK3CD | -0.1 | NM_000147 | FUCA1 | -0.1 |
| NM_003001 | SDHC | -0.1 | NM_006009 | TUBA3 | -0.1 |
| NM_002014 | FKBP4 | -0.1 | NM_020410 | ATP13A | -0.1 |
| NM_002931 | RING1 | -0.1 | NM_005811 | GDF11 | -0.1 |
| NM_005623 | CCL8 | -0.1 | NM_000591 | CD14 | -0.1 |
| NM_001627 | ALCAM | -0.1 | NM_002272 | KRT4 | -0.1 |
| NM_153334 | SCARF2 | -0.1 | NM_145818 | COG4 | -0.1 |
| NM_018190 | BBS7 | -0.1 | NM_153478 | CSAG1 | -0.1 |
| NM_016412 | IGF2AS | -0.1 | NM_003978 | PSTPIP1 | -0.1 |
| NR_001447 | MT1L | -0.1 | NM_024611 | NARG2 | -0.1 |
| NM_002977 | SCN9A | -0.1 | NM_002888 | RARRES1 | -0.1 |
| NM_003149 | STAC | -0.1 | NM_017818 | WDR8 | -0.1 |
| NM_001693 | ATP6V1B2 | -0.1 | NM_014206 | C11ORF10 | -0.1 |
| NM_000704 | ATP4A | -0.1 | NM_001503 | GPLD1 | -0.1 |
| NM_021728 | OTX2 | -0.1 | NM_005947 | MT1B | -0.1 |
| NM_017651 | AHI1 | -0.1 | NM_002780 | PSG4 | -0.1 |
| NM_002909 | REG1A | -0.1 | NM_003543 | HIST1H4H | -0.1 |
| NM_153029 | N4BP1 | -0.1 | XM_371036 | KIAA0100 | -0.1 |
| NM_014706 | SART3 | -0.1 | NM_006049 | SNAPC5 | -0.1 |
| NM_006438 | COLEC10 | -0.1 | NM_025176 | KIAA0980 | -0.1 |
| XM_038664 | KIAA0564 | -0.1 | NM_016643 | LOC51333 | -0.1 |
| XM_042685 | KIAA1414 | -0.1 | XM_030729 | DKFZP434I1117 | -0.1 |
| XM_371082 | FLJ20753 | -0.1 | NM_017837 | FLJ20477 | -0.1 |
| NM_017849 | FLJ20507 | -0.1 | NM_019594 | LRRC8 | -0.1 |
| NM_024065 | PDCL3 | -0.1 | NM_024723 | FLJ23471 | -0.1 |
| NM_024956 | FLJ23375 | -0.1 | XM_046861 | BA526D8.4 | -0.1 |
| NM_080596 | HIST1H2AH | -0.1 | NM_145254 | LOC124491 | -0.1 |
| NM_213596 | FOXN4 | -0.1 | NM_203298 | CHCHD1 | -0.1 |
| NM_194279 | HBLD1 | -0.1 | NM_178859 | OSTBETA | -0.1 |
| NM_145271 | LOC146542 | -0.1 | XM_379437 | LOC153910 | -0.1 |
| NM_203408 | LOC158724 | -0.1 | NM_178498 | MGC52019 | -0.1 |
| NM_174927 | SPAS1 | -0.1 | NM_173552 | MGC33365 | -0.1 |
| NM_178816 | CASC2 | -0.1 | XM_375443 | LOC284100 | -0.1 |
| NM_181609 | KRTAP19-3 | -0.1 | NM_181840 | TRIK | -0.1 |
| NM_207172 | GPR154 | -0.1 | NM_182827 | FKBP9L | -0.1 |
| NM_001004342 | TRIM67 | -0.1 | XM_173120 | LOC254938 | -0.1 |
| XM_292664 | LOC342659 | -0.1 | XM_209196 | LOC284422 | -0.1 |
| XM_066484 | LOC139116 | -0.1 | XM_373824 | LOC388589 | -0.1 |
| XM_379077 | LOC400944 | -0.1 | XM_373600 | LOC388002 | -0.1 |
| NM_016248 | AKAP11 | -0.09 | NM_017662 | TRPM6 | -0.09 |
| NM_005433 | YES1 | -0.09 | NM_006794 | GPR75 | -0.09 |

| | | |
|---|---|---|
| NM_013936 | OR12D2 | -0.09 |
| NM_017422 | CALML5 | -0.09 |
| NM_000247 | MICA | -0.09 |
| NM_004316 | ASCL1 | -0.09 |
| NM_012481 | ZNFN1A3 | -0.09 |
| NM_016576 | GMPR2 | -0.09 |
| NM_003288 | TPD52L2 | -0.09 |
| NM_001889 | CRYZ | -0.09 |
| NM_022089 | HSA9947 | -0.09 |
| XM_097508 | LGR6 | -0.09 |
| NM_021132 | PPP3CB | -0.09 |
| NM_130770 | HTR3C | -0.09 |
| NM_052866 | ADAMTSL1 | -0.09 |
| NM_005876 | APEG1 | -0.09 |
| NM_005573 | LMNB1 | -0.09 |
| NM_005584 | MAB21L1 | -0.09 |
| NM_003027 | SH3GL3 | -0.09 |
| XM_372058 | PVT1 | -0.09 |
| NM_004968 | ICA1 | -0.09 |
| NM_005337 | HEM1 | -0.09 |
| NM_005042 | PRH2 | -0.09 |
| NM_003046 | SLC7A2 | -0.09 |
| NM_007113 | THH | -0.09 |
| NM_003444 | ZNF154 | -0.09 |
| NM_004694 | SLC16A6 | -0.09 |
| NM_004713 | SDCCAG1 | -0.09 |
| XM_376518 | C6ORF84 | -0.09 |
| NM_015278 | SASH1 | -0.09 |
| NM_015602 | LAP1B | -0.09 |
| NM_013400 | REPIN1 | -0.09 |
| NM_018967 | SNTG1 | -0.09 |
| NM_015590 | FLJ20249 | -0.09 |
| NM_031415 | MLZE | -0.09 |
| NM_021242 | MIG12 | -0.09 |
| XM_291090 | RNF150 | -0.09 |
| NM_024762 | ZNF552 | -0.09 |
| NM_030817 | DKFZP434F0318 | -0.09 |
| NM_032836 | FLJ14768 | -0.09 |
| NM_032377 | MGC4549 | -0.09 |
| XM_054983 | ZNF618 | -0.09 |
| NM_058169 | LOH12CR1 | -0.09 |
| NM_130773 | CNTNAP5 | -0.09 |
| XM_378550 | LOC145757 | -0.09 |
| XM_032542 | FLJ41352 | -0.09 |
| NM_182529 | THAP5 | -0.09 |
| NM_173591 | FLJ90579 | -0.09 |
| NM_012368 | OR2C1 | -0.09 |
| NM_014056 | HIG1 | -0.09 |
| NM_000295 | SERPINA1 | -0.09 |
| NM_006896 | HOXA7 | -0.09 |
| NM_016066 | GLRX2 | -0.09 |
| NM_004455 | EXTL1 | -0.09 |
| NM_030648 | SET7 | -0.09 |
| NM_000444 | PHEX | -0.09 |
| NM_007218 | RNF139 | -0.09 |
| NM_022455 | NSD1 | -0.09 |
| NM_017581 | CHRNA9 | -0.09 |
| NM_001632 | ALPP | -0.09 |
| NM_006433 | GNLY | -0.09 |
| NM_006353 | HMGN4 | -0.09 |
| NM_016424 | LUC7A | -0.09 |
| NM_017569 | P38IP | -0.09 |
| NM_005414 | SKIL | -0.09 |
| NM_001215 | CA6 | -0.09 |
| NM_005216 | DDOST | -0.09 |
| NM_001532 | SLC29A2 | -0.09 |
| NM_004545 | NDUFB1 | -0.09 |
| NM_002978 | SCNN1D | -0.09 |
| NM_004645 | COIL | -0.09 |
| NM_003575 | ZNF282 | -0.09 |
| NM_003795 | SNX3 | -0.09 |
| NM_014771 | RNF40 | -0.09 |
| XM_166270 | KIAA0774 | -0.09 |
| NM_015478 | L3MBTL | -0.09 |
| NM_138768 | MYEOV | -0.09 |
| NM_016297 | PCYOX1 | -0.09 |
| NM_017868 | TTC12 | -0.09 |
| NM_017530 | LOC55565 | -0.09 |
| NM_020163 | LOC56920 | -0.09 |
| XM_172801 | KIAA1210 | -0.09 |
| NM_024051 | C7ORF24 | -0.09 |
| NM_024782 | FLJ12610 | -0.09 |
| NM_031955 | NYD-SP12 | -0.09 |
| NM_032526 | NT5C1A | -0.09 |
| NM_145243 | MPRP-1 | -0.09 |
| NM_173477 | USH1G | -0.09 |
| XM_069035 | C6ORF213 | -0.09 |
| XM_378680 | LOC147080 | -0.09 |
| NM_153353 | MGC27085 | -0.09 |
| NM_152583 | MGC40053 | -0.09 |
| NM_152699 | SENP5 | -0.09 |
| NM_203392 | LOC284123 | -0.09 |

| | | | | | |
|---|---|---|---|---|---|
| NM_133466 | ZNF545 | -0.09 | XM_376566 | LOC285924 | -0.09 |
| NM_203282 | ZNF539 | -0.09 | NM_078471 | MYO18A | -0.09 |
| NM_207581 | LOC405753 | -0.09 | XM_208204 | LOC284441 | -0.09 |
| XM_373056 | LOC391742 | -0.09 | XM_374290 | LOC389703 | -0.09 |
| XM_370898 | LOC388166 | -0.09 | XM_371019 | LOC388339 | -0.09 |
| XM_377027 | LOC401605 | -0.09 | XM_377073 | LOC401624 | -0.09 |
| NM_001001938 | C9ORF47 | -0.09 | NM_005255 | GAK | -0.08 |
| NM_030768 | ILKAP | -0.08 | NM_002530 | NTRK3 | -0.08 |
| NM_000431 | MVK | -0.08 | NM_005161 | AGTRL1 | -0.08 |
| NM_014496 | RPS6KA6 | -0.08 | NM_015292 | MBC2 | -0.08 |
| NM_130806 | LGR8 | -0.08 | NM_002921 | RGR | -0.08 |
| NM_014004 | PCDHGA8 | -0.08 | NM_006499 | LGALS8 | -0.08 |
| NM_005312 | RAPGEF1 | -0.08 | NM_001938 | DR1 | -0.08 |
| NM_002479 | MYOG | -0.08 | NM_002460 | IRF4 | -0.08 |
| NM_177398 | LMX1A | -0.08 | NM_017825 | ADPRHL2 | -0.08 |
| NM_001648 | KLK3 | -0.08 | NM_000434 | NEU1 | -0.08 |
| NM_020663 | RHOJ | -0.08 | NM_000373 | UMPS | -0.08 |
| NM_005373 | MPL | -0.08 | NM_002119 | HLA-DOA | -0.08 |
| NM_001192 | TNFRSF17 | -0.08 | NM_000173 | GP1BA | -0.08 |
| NM_000419 | ITGA2B | -0.08 | NM_000745 | CHRNA5 | -0.08 |
| NM_000089 | COL1A2 | -0.08 | NM_144606 | BHD | -0.08 |
| NM_052818 | CG018 | -0.08 | NM_002994 | CXCL5 | -0.08 |
| NM_017589 | BTG4 | -0.08 | NM_001240 | CCNT1 | -0.08 |
| NM_006021 | DLEU2 | -0.08 | NM_000420 | KEL | -0.08 |
| XM_049619 | PRDM6 | -0.08 | NM_004136 | IREB2 | -0.08 |
| NM_002218 | ITIH4 | -0.08 | NM_003022 | SH3BGRL | -0.08 |
| NM_007131 | ZNF75 | -0.08 | XM_371150 | ZNF90 | -0.08 |
| NM_003128 | SPTBN1 | -0.08 | NM_007148 | ZNF179 | -0.08 |
| NM_003613 | CILP | -0.08 | NM_003938 | AP3D1 | -0.08 |
| NM_004784 | NDST3 | -0.08 | NM_014811 | KIAA0649 | -0.08 |
| NM_006869 | CENTA1 | -0.08 | NM_015265 | SATB2 | -0.08 |
| NM_022044 | SDF2L1 | -0.08 | NM_012192 | FXC1 | -0.08 |
| NM_014047 | HSPC023 | -0.08 | NM_014464 | TINAG | -0.08 |
| NM_014506 | TOR1B | -0.08 | NM_014549 | DKFZP434P211 | -0.08 |
| NM_016423 | ZNF219 | -0.08 | NM_016947 | C6ORF48 | -0.08 |
| NM_144564 | SLC39A3 | -0.08 | NM_016116 | ASB4 | -0.08 |
| NM_019001 | 37135 | -0.08 | NM_017907 | FLJ20625 | -0.08 |
| NM_016143 | NSFL1C | -0.08 | XM_166523 | TTYH3 | -0.08 |
| NM_025155 | FLJ11848 | -0.08 | NM_030772 | GJA10 | -0.08 |
| NM_031304 | MGC4293 | -0.08 | NM_031469 | SH3BGRL2 | -0.08 |
| NM_032123 | KIRREL2 | -0.08 | NM_032608 | MYO18B | -0.08 |
| NM_032347 | ZNF397 | -0.08 | NM_032510 | PARD6G | -0.08 |
| NM_032854 | FLJ14871 | -0.08 | NM_032380 | EFG2 | -0.08 |
| NM_033130 | SIGLEC10 | -0.08 | XM_054951 | GALNT13 | -0.08 |
| NM_053283 | DCD | -0.08 | NM_178814 | AP1S3 | -0.08 |
| NM_153216 | FLJ25680 | -0.08 | NM_145049 | MGC10067 | -0.08 |

| | | | | | |
|---|---|---|---|---|---|
| NM_152369 | MGC45474 | -0.08 | NM_199511 | URB | -0.08 |
| NM_145912 | NFAM1 | -0.08 | NM_144702 | FLJ32884 | -0.08 |
| NM_145307 | PLEKHK1 | -0.08 | NM_152738 | MGC40222 | -0.08 |
| NM_018325 | C9ORF72 | -0.08 | NM_152784 | MGC39581 | -0.08 |
| NM_182607 | MGC44287 | -0.08 | NM_178315 | FLJ39582 | -0.08 |
| NM_198512 | FLJ25989 | -0.08 | NM_015153 | PHF3 | -0.08 |
| NM_020467 | LOC57228 | -0.08 | NM_207424 | FLJ40536 | -0.08 |
| XM_059132 | LOC127309 | -0.08 | XM_290660 | LOC339003 | -0.08 |
| XM_293943 | LOC340096 | -0.08 | XM_293342 | LOC347424 | -0.08 |
| XM_209889 | LOC286076 | -0.08 | XM_378925 | LOC400800 | -0.08 |
| XM_377880 | LOC402203 | -0.08 | XM_379838 | LOC402505 | -0.08 |
| XM_374787 | LOC399747 | -0.08 | XM_373451 | LOC387647 | -0.08 |
| XM_370665 | LOC387820 | -0.08 | XM_378743 | LOC400647 | -0.08 |
| NM_001004334 | GPR158L1 | -0.08 | XM_378156 | LOC402436 | -0.08 |
| NM_172194 | OR4Q3 | -0.08 | NM_206967 | MGC17624 | -0.08 |
| NM_004064 | CDKN1B | -0.07 | NM_018440 | PAG | -0.07 |
| NM_007118 | TRIO | -0.07 | NM_000634 | IL8RA | -0.07 |
| NM_002959 | SORT1 | -0.07 | NM_006192 | PAX1 | -0.07 |
| NM_004741 | NOLC1 | -0.07 | NM_004474 | FOXD2 | -0.07 |
| NM_002040 | GABPA | -0.07 | NM_021163 | RBAK | -0.07 |
| NM_000538 | RFXAP | -0.07 | NM_018488 | TBX4 | -0.07 |
| NM_002629 | PGAM1 | -0.07 | NM_007171 | POMT1 | -0.07 |
| NM_004550 | NDUFS2 | -0.07 | NM_004615 | TM4SF2 | -0.07 |
| NM_002839 | PTPRD | -0.07 | NM_017636 | TRPM4 | -0.07 |
| NM_000605 | IFNA2 | -0.07 | NM_005691 | ABCC9 | -0.07 |
| NM_002007 | FGF4 | -0.07 | NM_001066 | TNFRSF1B | -0.07 |
| NM_002252 | KCNS3 | -0.07 | NM_004323 | BAG1 | -0.07 |
| NM_032124 | DKFZP564D1378 | -0.07 | NM_152622 | FLJ35954 | -0.07 |
| NM_021973 | HAND2 | -0.07 | NM_002165 | ID1 | -0.07 |
| NM_014891 | PDAP1 | -0.07 | NM_145040 | PRKCDBP | -0.07 |
| NM_006861 | RAB35 | -0.07 | NM_003082 | SNAPC1 | -0.07 |
| NM_005634 | SOX3 | -0.07 | NM_000850 | GSTM4 | -0.07 |
| NM_004130 | GYG | -0.07 | NM_001467 | SLC37A4 | -0.07 |
| NM_004121 | GGTLA1 | -0.07 | NM_002797 | PSMB5 | -0.07 |
| NM_032360 | ACBD6 | -0.07 | NM_005664 | MKRN3 | -0.07 |
| NM_005674 | ZNF239 | -0.07 | NM_003536 | HIST1H3H | -0.07 |
| NM_005423 | TFF2 | -0.07 | NM_004639 | BAT3 | -0.07 |
| NM_030664 | PTER | -0.07 | NM_014793 | LCMT2 | -0.07 |
| NM_014835 | OSBPL2 | -0.07 | NM_014772 | KIAA0427 | -0.07 |
| NM_002510 | GPNMB | -0.07 | XM_496278 | ZNF516 | -0.07 |
| NM_006808 | SEC61B | -0.07 | XM_291291 | DDHD2 | -0.07 |
| NM_015525 | IBTK | -0.07 | NM_014055 | CDV-1 | -0.07 |
| NM_014412 | SIP | -0.07 | NM_016215 | EGFL7 | -0.07 |
| NM_017738 | C9ORF39 | -0.07 | NM_017658 | BTBD5 | -0.07 |
| NM_017875 | FLJ20551 | -0.07 | NM_020374 | C12ORF4 | -0.07 |
| NM_019896 | POLE4 | -0.07 | NM_015455 | KIAA1194 | -0.07 |

| | | |
|---|---|---|
| XM_036299 | KIAA1522 | -0.07 |
| NM_022736 | FLJ14153 | -0.07 |
| NM_024518 | ULBP3 | -0.07 |
| NM_032154 | PCGF6 | -0.07 |
| XM_376656 | MYH16 | -0.07 |
| NM_032257 | ZMYND12 | -0.07 |
| NM_032783 | FLJ14431 | -0.07 |
| NM_033089 | C20ORF99 | -0.07 |
| XM_370870 | MGC4809 | -0.07 |
| XM_291105 | MGC21874 | -0.07 |
| NM_153833 | H1FOO | -0.07 |
| NM_173498 | DKFZP434C0631 | -0.07 |
| NM_181780 | BTLA | -0.07 |
| NM_152702 | C9ORF94 | -0.07 |
| NM_145060 | C18ORF24 | -0.07 |
| NM_173810 | MGC29649 | -0.07 |
| XM_171410 | DKFZP667B0210 | -0.07 |
| NM_173825 | RABL3 | -0.07 |
| NM_182584 | FLJ33706 | -0.07 |
| NM_205864 | CTAG3 | -0.07 |
| NM_182595 | DKFZP564N2472 | -0.07 |
| NM_003760 | EIF4G3 | -0.07 |
| NM_207452 | FLJ45200 | -0.07 |
| XM_295059 | LOC339771 | -0.07 |
| XM_087141 | LOC151256 | -0.07 |
| XM_374071 | LOC389189 | -0.07 |
| XM_377109 | LOC401638 | -0.07 |
| XM_378747 | LOC400651 | -0.07 |
| NM_006256 | PRKCL2 | -0.06 |
| NM_007369 | GPR161 | -0.06 |
| NM_005631 | SMO | -0.06 |
| NM_004322 | BAD | -0.06 |
| NM_018906 | PCDHA3 | -0.06 |
| NM_006094 | DLC1 | -0.06 |
| NM_000159 | GCDH | -0.06 |
| NM_005992 | TBX1 | -0.06 |
| NM_018961 | UBASH3A | -0.06 |
| NM_007037 | ADAMTS8 | -0.06 |
| NM_000410 | HFE | -0.06 |
| NM_014452 | TNFRSF21 | -0.06 |
| NM_006858 | IL1RL1LG | -0.06 |
| NM_005191 | CD80 | -0.06 |
| NM_002060 | GJA4 | -0.06 |
| NM_002614 | PDZK1 | -0.06 |
| NM_012110 | CHIC2 | -0.06 |
| NM_024817 | FLJ13710 | -0.06 |
| NM_022373 | FLJ22313 | -0.07 |
| NM_022769 | TORC3 | -0.07 |
| NM_030922 | NIPA2 | -0.07 |
| NM_031429 | RTBDN | -0.07 |
| NM_032041 | NCALD | -0.07 |
| NM_032572 | RNASE7 | -0.07 |
| NM_033546 | MRLC2 | -0.07 |
| NM_138300 | PYGO2 | -0.07 |
| NM_145236 | B3GNT7 | -0.07 |
| NM_145267 | C6ORF57 | -0.07 |
| NM_152442 | RAD9B | -0.07 |
| NM_153219 | ZNF524 | -0.07 |
| NM_138279 | BNIPL | -0.07 |
| NM_145063 | C6ORF130 | -0.07 |
| NM_175076 | C8ORF15 | -0.07 |
| NM_175062 | RASGEF1C | -0.07 |
| NM_173813 | FLJ34154 | -0.07 |
| XM_378514 | LOC283663 | -0.07 |
| XM_378606 | LOC283867 | -0.07 |
| XM_295155 | LOC340094 | -0.07 |
| NM_181612 | KRTAP19-6 | -0.07 |
| NM_207443 | FLJ45244 | -0.07 |
| XM_293042 | LOC344423 | -0.07 |
| XM_117257 | LOC200624 | -0.07 |
| XM_351723 | LOC375593 | -0.07 |
| XM_376305 | LOC401121 | -0.07 |
| XM_373506 | LOC387784 | -0.07 |
| NM_153273 | IHPK1 | -0.06 |
| NM_145185 | MAP2K7 | -0.06 |
| NM_006174 | NPY5R | -0.06 |
| NM_176882 | TAS2R40 | -0.06 |
| NM_012323 | MAFF | -0.06 |
| NM_004292 | RIN1 | -0.06 |
| NM_057179 | TWIST2 | -0.06 |
| NM_000363 | TNNI3 | -0.06 |
| NM_000372 | TYR | -0.06 |
| NM_005084 | PLA2G7 | -0.06 |
| NM_005355 | KIF25 | -0.06 |
| NM_004758 | BZRAP1 | -0.06 |
| NM_145068 | TRPV3 | -0.06 |
| NM_014978 | SORCS3 | -0.06 |
| NM_000812 | GABRB1 | -0.06 |
| NM_003060 | SLC22A5 | -0.06 |
| NM_033027 | AXUD1 | -0.06 |
| NM_013263 | BRD7 | -0.06 |
| NM_002281 | KRTHB1 | -0.06 |

| | | |
|---|---|---|
| NM_006152 | LRMP | -0.06 |
| NM_006283 | TACC1 | -0.06 |
| NM_183357 | ADCY5 | -0.06 |
| NM_004398 | DDX10 | -0.06 |
| NM_024501 | HOXD1 | -0.06 |
| NM_002408 | MGAT2 | -0.06 |
| NM_020683 | AD026 | -0.06 |
| NM_003164 | STX5A | -0.06 |
| NM_004923 | MTL5 | -0.06 |
| NM_005740 | DNAL4 | -0.06 |
| NM_014864 | FAM20B | -0.06 |
| NM_012467 | TPSG1 | -0.06 |
| NM_012417 | PITPNC1 | -0.06 |
| NM_016073 | HDGFRP3 | -0.06 |
| NM_058186 | FAM3B | -0.06 |
| NM_017794 | KIAA1797 | -0.06 |
| NM_018090 | FLJ10420 | -0.06 |
| NM_020181 | C14ORF162 | -0.06 |
| NM_022746 | FLJ22390 | -0.06 |
| NM_024323 | MGC11271 | -0.06 |
| NM_024942 | C10ORF88 | -0.06 |
| NM_024909 | C6ORF134 | -0.06 |
| NM_080666 | C14ORF150 | -0.06 |
| NM_133636 | HEL308 | -0.06 |
| XM_043739 | LOC92235 | -0.06 |
| NM_182510 | FLJ32252 | -0.06 |
| NM_198078 | C21ORF121 | -0.06 |
| NM_172366 | FBXO16 | -0.06 |
| NM_145288 | ZNF342 | -0.06 |
| NM_198471 | FLJ46061 | -0.06 |
| NM_182760 | SUMF1 | -0.06 |
| NM_178538 | LOC338799 | -0.06 |
| NM_207486 | FLJ44076 | -0.06 |
| XM_291075 | LOC339926 | -0.06 |
| XM_087761 | LOC153770 | -0.06 |
| XM_377558 | LOC401937 | -0.06 |
| XM_376314 | LOC401125 | -0.06 |
| XM_378512 | LOC400369 | -0.06 |
| XM_378646 | LOC400571 | -0.06 |
| NM_001004054 | POTE2 | -0.06 |
| XM_031612 | EIF2AK4 | -0.05 |
| NM_001974 | EMR1 | -0.05 |
| NM_005795 | CALCRL | -0.05 |
| NM_000139 | MS4A2 | -0.05 |
| NM_003835 | RGS9 | -0.05 |
| NM_032390 | MKI67IP | -0.05 |
| NM_002690 | POLB | -0.06 |
| NM_021000 | PTTG3 | -0.06 |
| NM_004057 | CALB3 | -0.06 |
| NR_001435 | HLA-DPB2 | -0.06 |
| NM_006196 | PCBP1 | -0.06 |
| NM_002668 | PLP2 | -0.06 |
| NM_003274 | TMEM1 | -0.06 |
| NM_003631 | PARG | -0.06 |
| XM_113947 | KIAA0565 | -0.06 |
| NM_014848 | SV2B | -0.06 |
| XM_031553 | SR140 | -0.06 |
| NM_015439 | C6ORF80 | -0.06 |
| NM_014498 | GOLPH4 | -0.06 |
| NM_017550 | KIAA1193 | -0.06 |
| NM_016207 | CPSF3 | -0.06 |
| NM_018130 | FLJ10539 | -0.06 |
| NM_018676 | THSD1 | -0.06 |
| NM_021185 | C19ORF15 | -0.06 |
| NM_023112 | OTUB2 | -0.06 |
| NM_033467 | MMEL2 | -0.06 |
| NM_025218 | ULBP1 | -0.06 |
| NM_032331 | MGC2408 | -0.06 |
| XM_032304 | LOC90525 | -0.06 |
| NM_148897 | SDR-O | -0.06 |
| NM_153035 | FLJ32112 | -0.06 |
| NM_144664 | MGC33371 | -0.06 |
| NM_138814 | LOC150379 | -0.06 |
| NM_133462 | TTC14 | -0.06 |
| NM_145000 | FLJ25422 | -0.06 |
| XM_375449 | LOC284106 | -0.06 |
| NM_181725 | FLJ12760 | -0.06 |
| NM_198947 | CANP | -0.06 |
| XM_209252 | LOC284546 | -0.06 |
| XM_292723 | LOC342933 | -0.06 |
| XM_209918 | LOC286151 | -0.06 |
| XM_376281 | LOC401097 | -0.06 |
| XM_373559 | LOC387905 | -0.06 |
| XM_378325 | LOC399978 | -0.06 |
| NM_001002907 | OR8K1 | -0.06 |
| NM_003646 | DGKZ | -0.05 |
| NM_006909 | RASGRF2 | -0.05 |
| NM_001735 | C5 | -0.05 |
| NM_144733 | GPR73L1 | -0.05 |
| NM_018949 | GPR14 | -0.05 |
| NM_001231 | CASQ1 | -0.05 |
| NM_000251 | MSH2 | -0.05 |

| | | | | | |
|---|---|---|---|---|---|
| NM_014239 | EIF2B2 | -0.05 | NM_006885 | ATBF1 | -0.05 |
| NM_005144 | HR | -0.05 | NM_000098 | CPT2 | -0.05 |
| NM_172238 | TFAP2BL1 | -0.05 | NM_001190 | BCAT2 | -0.05 |
| NM_018044 | WBSCR20A | -0.05 | NM_002775 | PRSS11 | -0.05 |
| NM_004401 | DFFA | -0.05 | NM_004145 | MYO9B | -0.05 |
| NM_002885 | RAP1GA1 | -0.05 | NM_001615 | ACTG2 | -0.05 |
| NM_015906 | TRIM33 | -0.05 | NM_004652 | USP9X | -0.05 |
| NM_012125 | CHRM5 | -0.05 | NM_002355 | M6PR | -0.05 |
| NM_002779 | PSD | -0.05 | NM_015319 | TENC1 | -0.05 |
| NM_016562 | TLR7 | -0.05 | NM_005430 | WNT1 | -0.05 |
| NM_014862 | ARNT2 | -0.05 | NM_000475 | NR0B1 | -0.05 |
| NM_005654 | NR2F1 | -0.05 | NM_002293 | LAMC1 | -0.05 |
| NM_003154 | STATH | -0.05 | NM_000092 | COL4A4 | -0.05 |
| NM_003797 | EED | -0.05 | NM_020952 | TRPM3 | -0.05 |
| NM_080476 | CDC91L1 | -0.05 | NM_019061 | PIP3AP | -0.05 |
| NM_017784 | OSBPL10 | -0.05 | NM_006695 | RPIP8 | -0.05 |
| NM_003061 | SLIT1 | -0.05 | NM_001423 | EMP1 | -0.05 |
| NM_005896 | IDH1 | -0.05 | NM_004396 | DDX5 | -0.05 |
| NM_021063 | HIST1H2BD | -0.05 | NM_152713 | ITM1 | -0.05 |
| NM_001926 | DEFA6 | -0.05 | NM_012335 | MYO1F | -0.05 |
| NM_004533 | MYBPC2 | -0.05 | NM_020132 | AGPAT3 | -0.05 |
| NM_006905 | PSG1 | -0.05 | NM_003087 | SNCG | -0.05 |
| NM_005410 | SEPP1 | -0.05 | NM_001005478 | GGNBP1 | -0.05 |
| NM_012080 | FAM16AX | -0.05 | XM_375247 | ZNF592 | -0.05 |
| NM_014824 | FCHSD2 | -0.05 | NM_014730 | KIAA0152 | -0.05 |
| XM_291106 | KIAA0232 | -0.05 | NM_014752 | KIAA0102 | -0.05 |
| NM_007231 | SLC6A14 | -0.05 | NM_015464 | SOSTDC1 | -0.05 |
| NM_012263 | TTLL1 | -0.05 | NM_015364 | LY96 | -0.05 |
| NM_013301 | HSU79303 | -0.05 | NM_016503 | MRPL30 | -0.05 |
| NM_017941 | HLC-8 | -0.05 | XM_372231 | FLJ20105 | -0.05 |
| NM_017840 | MRPL16 | -0.05 | NM_017691 | FLJ20156 | -0.05 |
| NM_017851 | FLJ20509 | -0.05 | NM_018666 | SAGE1 | -0.05 |
| NM_018491 | LOC55871 | -0.05 | NM_020409 | MRPL47 | -0.05 |
| NM_020422 | LOC57146 | -0.05 | NM_018728 | MYO5C | -0.05 |
| NM_018847 | KIAA1354 | -0.05 | NM_020340 | KIAA1244 | -0.05 |
| NM_031895 | CACNG8 | -0.05 | NM_021217 | ZNF77 | -0.05 |
| NM_022758 | C6ORF106 | -0.05 | NM_033067 | DMRTB1 | -0.05 |
| NM_022120 | OXCT2 | -0.05 | NM_024034 | GDAP1L1 | -0.05 |
| NM_025244 | TSGA10 | -0.05 | NM_025189 | ZNF430 | -0.05 |
| NM_025041 | FLJ22173 | -0.05 | NM_032038 | SPINL | -0.05 |
| NM_032044 | REG4 | -0.05 | NM_031479 | INHBE | -0.05 |
| NM_032596 | C9ORF24 | -0.05 | NM_207308 | LOC91181 | -0.05 |
| XM_370767 | C14ORF46 | -0.05 | NM_130784 | C10ORF94 | -0.05 |
| NM_144578 | C14ORF32 | -0.05 | NM_145262 | GLYCTK | -0.05 |
| XM_379196 | LOC151877 | -0.05 | NM_139174 | LOC161931 | -0.05 |
| NM_130772 | S100Z | -0.05 | NM_173535 | CLECSF13 | -0.05 |

| | | | | | |
|---|---|---|---|---|---|
| NM_024852 | EIF2C3 | -0.05 | XM_113625 | LOC195977 | -0.05 |
| NM_139284 | LGI4 | -0.05 | XM_093024 | LOC169981 | -0.05 |
| NM_152644 | FAM24B | -0.05 | XM_113871 | LOC197350 | -0.05 |
| NM_174918 | LOC199675 | -0.05 | NM_145659 | IL27 | -0.05 |
| NM_198151 | LOC253012 | -0.05 | NM_147195 | FLJ35740 | -0.05 |
| NM_175854 | PAN3 | -0.05 | NM_173611 | FLJ38426 | -0.05 |
| XM_209413 | LOC284912 | -0.05 | XM_295270 | LOC340581 | -0.05 |
| NM_003522 | HIST1H2BF | -0.05 | NM_203454 | MGC26594 | -0.05 |
| XM_376622 | LOC401361 | -0.05 | XM_373094 | LOC391817 | -0.05 |
| XM_377956 | LOC402286 | -0.05 | XM_374260 | LOC389641 | -0.05 |
| XM_373478 | LOC387717 | -0.05 | XM_373583 | LOC387974 | -0.05 |
| XM_372424 | LOC390233 | -0.05 | XM_372307 | LOC389950 | -0.05 |
| XM_370718 | LOC387914 | -0.05 | XM_378783 | LOC400680 | -0.05 |
| NM_001004326 | FLJ16331 | -0.05 | NM_001001872 | C14ORF37 | -0.05 |
| XM_372224 | LOC389860 | -0.05 | NM_172089 | TNFSF12-TNFSF13 | -0.05 |
| NM_002314 | LIMK1 | -0.04 | NM_016557 | CCRL1 | -0.04 |
| NM_000957 | PTGER3 | -0.04 | NM_014565 | OR1A1 | -0.04 |
| NM_002411 | SCGB2A2 | -0.04 | NM_152860 | SP7 | -0.04 |
| NM_002015 | FOXO1A | -0.04 | NM_024503 | HIVEP3 | -0.04 |
| NM_000063 | C2 | -0.04 | NM_015989 | CSAD | -0.04 |
| NM_001912 | CTSL | -0.04 | NM_022060 | ABHD4 | -0.04 |
| NM_006395 | APG7L | -0.04 | NM_000182 | HADHA | -0.04 |
| NM_005908 | MANBA | -0.04 | NM_002021 | FMO1 | -0.04 |
| NM_001698 | AUH | -0.04 | NM_004879 | EI24 | -0.04 |
| XM_030577 | ATP9A | -0.04 | NM_003909 | CPNE3 | -0.04 |
| NM_007368 | RASA3 | -0.04 | NM_017778 | WHSC1L1 | -0.04 |
| NM_207111 | TRIAD3 | -0.04 | NM_003807 | TNFSF14 | -0.04 |
| NM_001525 | HCRTR1 | -0.04 | NM_018646 | TRPV6 | -0.04 |
| NM_002673 | PLXNB1 | -0.04 | NM_000810 | GABRA5 | -0.04 |
| NM_005123 | NR1H4 | -0.04 | NM_001646 | APOC4 | -0.04 |
| NM_004425 | ECM1 | -0.04 | NM_001640 | APEH | -0.04 |
| NM_020980 | AQP9 | -0.04 | NM_002267 | KPNA3 | -0.04 |
| NM_000668 | ADH1B | -0.04 | NM_003914 | CCNA1 | -0.04 |
| NM_003772 | JRKL | -0.04 | NM_007250 | KLF8 | -0.04 |
| NM_001995 | ACSL1 | -0.04 | NM_000466 | PEX1 | -0.04 |
| NM_018256 | WDR12 | -0.04 | NM_004906 | WTAP | -0.04 |
| NM_004047 | ATP6V0B | -0.04 | NM_001424 | EMP2 | -0.04 |
| NM_002084 | GPX3 | -0.04 | NM_002018 | FLII | -0.04 |
| NM_002278 | KRTHA2 | -0.04 | NM_006188 | OCM | -0.04 |
| NM_024410 | ODF1 | -0.04 | NM_002722 | PPY | -0.04 |
| NM_018308 | ACOXL | -0.04 | NM_003040 | SLC4A2 | -0.04 |
| NM_024105 | ALG12 | -0.04 | NM_002901 | RCN1 | -0.04 |
| NM_003115 | UAP1 | -0.04 | NM_003294 | TPSB1 | -0.04 |
| NM_004604 | STX4A | -0.04 | NM_007269 | STXBP3 | -0.04 |
| NM_004870 | MPDU1 | -0.04 | NM_003717 | NPFF | -0.04 |
| NM_014761 | KIAA0174 | -0.04 | NM_007075 | WDRX1 | -0.04 |

| | | | | | |
|---|---|---|---|---|---|
| NM_006843 | SDS | -0.04 | NM_007238 | PXMP4 | -0.04 |
| NM_006578 | GNB5 | -0.04 | NM_015323 | KIAA0776 | -0.04 |
| NM_015540 | RPAP1 | -0.04 | NM_014062 | NOB1P | -0.04 |
| NM_015654 | DKFZP564C103 | -0.04 | NM_015660 | GIMAP2 | -0.04 |
| NM_016931 | NOX4 | -0.04 | NM_016141 | DNCLI1 | -0.04 |
| NM_053039 | UGT2B28 | -0.04 | NM_019067 | FLJ10613 | -0.04 |
| NM_017759 | FLJ20309 | -0.04 | NM_018081 | FLJ10385 | -0.04 |
| NM_017665 | ZCCHC10 | -0.04 | NM_017895 | DDX27 | -0.04 |
| NM_020161 | DKFZP547H025 | -0.04 | NM_021061 | ZNF647 | -0.04 |
| NM_020713 | KIAA1196 | -0.04 | NM_022459 | XPO4 | -0.04 |
| NM_022785 | FLJ23588 | -0.04 | NM_023931 | MGC2474 | -0.04 |
| NM_031898 | TEKT3 | -0.04 | NM_022900 | CAS1 | -0.04 |
| NM_024040 | CUEDC2 | -0.04 | NM_024666 | FLJ11506 | -0.04 |
| NM_024588 | FLJ23584 | -0.04 | NM_024825 | FLJ23447 | -0.04 |
| NM_031424 | C20ORF55 | -0.04 | NM_198179 | GPR103 | -0.04 |
| NM_032801 | JAM3 | -0.04 | NM_032116 | KATNAL1 | -0.04 |
| NM_032772 | ZNF503 | -0.04 | XM_045086 | KIAA1764 | -0.04 |
| NM_033468 | ZNF257 | -0.04 | NM_152264 | SLC39A13 | -0.04 |
| NM_053002 | TRALPUSH | -0.04 | NM_080753 | WFDC10A | -0.04 |
| NM_144661 | C10ORF82 | -0.04 | NM_152480 | C19ORF23 | -0.04 |
| NM_152791 | ZNF555 | -0.04 | NM_173517 | VKORC1L1 | -0.04 |
| XM_097977 | LOC150946 | -0.04 | NM_145296 | IGSF4C | -0.04 |
| XM_290922 | MGC27277 | -0.04 | NM_145300 | LOC200420 | -0.04 |
| NM_145111 | DKFZP727G131 | -0.04 | NM_173551 | SAMD6 | -0.04 |
| NM_172240 | TUWD12 | -0.04 | XM_378238 | LOC283050 | -0.04 |
| NM_173570 | ZDHHC23 | -0.04 | XM_173083 | LOC255025 | -0.04 |
| NM_194309 | C21ORF125 | -0.04 | NM_182566 | LOC284013 | -0.04 |
| XM_379430 | LOC285758 | -0.04 | NM_207362 | MGC42367 | -0.04 |
| NM_198564 | FLJ44290 | -0.04 | NM_198079 | FLJ40113 | -0.04 |
| XM_371237 | CATSPER4 | -0.04 | NM_203308 | MGC34774 | -0.04 |
| NM_207436 | FLJ42957 | -0.04 | XM_293034 | LOC344405 | -0.04 |
| XM_171154 | LOC253883 | -0.04 | XM_378894 | LOC400778 | -0.04 |
| XM_374115 | LOC389273 | -0.04 | XM_379273 | LOC401149 | -0.04 |
| XM_371851 | LOC389432 | -0.04 | XM_374185 | LOC389437 | -0.04 |
| XM_379482 | LOC401320 | -0.04 | XM_377834 | LOC402167 | -0.04 |
| XM_372416 | LOC390205 | -0.04 | XM_378299 | LOC399919 | -0.04 |
| XM_372441 | LOC390271 | -0.04 | XM_378757 | LOC400659 | -0.04 |
| XM_371397 | LOC388795 | -0.04 | XM_377071 | LOC401622 | -0.04 |
| NM_001005516 | OR5K3 | -0.04 | NM_004677 | XKRY | -0.04 |
| NM_032910 | C21ORF119 | -0.04 | NM_000142 | FGFR3 | -0.03 |
| XM_290516 | HSMDPKIN | -0.03 | NM_002576 | PAK1 | -0.03 |
| NM_002595 | PCTK2 | -0.03 | NM_003690 | PRKRA | -0.03 |
| NM_002419 | MAP3K11 | -0.03 | NM_002054 | GCG | -0.03 |
| NM_004382 | CRHR1 | -0.03 | NM_000322 | RDS | -0.03 |
| NM_000871 | HTR6 | -0.03 | NM_016944 | TAS2R4 | -0.03 |
| NM_006352 | ZNF238 | -0.03 | NM_001642 | APLP2 | -0.03 |

| | | | | | |
|---|---|---|---|---|---|
| NM_021193 | HOXD12 | -0.03 | NM_002698 | POU2F2 | -0.03 |
| NM_001454 | FOXJ1 | -0.03 | NM_016531 | KLF3 | -0.03 |
| NM_003030 | SHOX2 | -0.03 | NM_002624 | PFDN5 | -0.03 |
| NM_002636 | PHF1 | -0.03 | NM_024527 | ABHD8 | -0.03 |
| NM_201252 | AFAR3 | -0.03 | NM_000398 | DIA1 | -0.03 |
| NM_017902 | HIF1AN | -0.03 | NM_020300 | MGST1 | -0.03 |
| NM_000817 | GAD1 | -0.03 | NM_000045 | ARG1 | -0.03 |
| NM_004826 | ECEL1 | -0.03 | NM_005420 | SULT1E1 | -0.03 |
| NM_002870 | RAB13 | -0.03 | NM_000544 | TAP2 | -0.03 |
| NM_004905 | PRDX6 | -0.03 | NM_017670 | OTUB1 | -0.03 |
| NM_021102 | SPINT2 | -0.03 | NM_012144 | DNAI1 | -0.03 |
| NM_004238 | TRIP12 | -0.03 | NM_004619 | TRAF5 | -0.03 |
| NM_145021 | MIR | -0.03 | NM_018643 | TREM1 | -0.03 |
| NM_002190 | IL17 | -0.03 | NM_002996 | CX3CL1 | -0.03 |
| NM_000894 | LHB | -0.03 | NM_006172 | NPPA | -0.03 |
| NM_001668 | ARNT | -0.03 | NM_005234 | NR2F6 | -0.03 |
| NM_006186 | NR4A2 | -0.03 | NM_006059 | LAMC3 | -0.03 |
| NM_004706 | ARHGEF1 | -0.03 | NM_024009 | GJB3 | -0.03 |
| NM_001788 | CDC10 | -0.03 | NM_000371 | TTR | -0.03 |
| NM_002871 | RABIF | -0.03 | NM_006698 | BLCAP | -0.03 |
| NM_001344 | DAD1 | -0.03 | NM_020188 | DC13 | -0.03 |
| NM_006708 | GLO1 | -0.03 | NM_002129 | HMGB2 | -0.03 |
| NM_004456 | EZH2 | -0.03 | NM_024316 | LENG1 | -0.03 |
| NM_183003 | COX7A3 | -0.03 | NM_005530 | IDH3A | -0.03 |
| NM_004220 | ZNF213 | -0.03 | NM_004605 | SULT2B1 | -0.03 |
| NM_021030 | ZNF14 | -0.03 | NM_003959 | HIP1R | -0.03 |
| NM_012262 | HS2ST1 | -0.03 | NM_003829 | MPDZ | -0.03 |
| NM_005116 | SLC23A2 | -0.03 | NM_172201 | KCNE2 | -0.03 |
| NM_005482 | PIGK | -0.03 | NM_005724 | TM4SF8 | -0.03 |
| XM_032719 | CARM1 | -0.03 | NM_016348 | C5ORF4 | -0.03 |
| NM_006551 | SCGB1D2 | -0.03 | NM_015115 | KIAA0276 | -0.03 |
| NM_015141 | KIAA0089 | -0.03 | XM_030524 | SLC9A8 | -0.03 |
| NM_207303 | ATRNL1 | -0.03 | NM_019844 | SLCO1B3 | -0.03 |
| NM_205859 | OR2K2 | -0.03 | NM_016500 | CXORF26 | -0.03 |
| NM_014593 | CXXC1 | -0.03 | NM_015911 | LOC51058 | -0.03 |
| NM_019021 | FLJ20010 | -0.03 | NM_017446 | MRPL39 | -0.03 |
| NM_016485 | C6ORF55 | -0.03 | NM_018374 | FLJ11273 | -0.03 |
| NM_019095 | C20ORF155 | -0.03 | NM_017549 | UCC1 | -0.03 |
| NM_017760 | MTB | -0.03 | NM_017785 | FLJ20364 | -0.03 |
| NM_018151 | RIF1 | -0.03 | NM_018029 | FLJ10213 | -0.03 |
| NM_017619 | FLJ25070 | -0.03 | NM_018462 | C3ORF10 | -0.03 |
| NM_022908 | FLJ12442 | -0.03 | NM_022745 | ATPAF1 | -0.03 |
| NM_024063 | SPATA5L1 | -0.03 | NM_030762 | BHLHB3 | -0.03 |
| NM_024676 | FLJ22938 | -0.03 | NM_025150 | FLJ12528 | -0.03 |
| NM_024967 | ZNF556 | -0.03 | NM_031212 | SLC25A28 | -0.03 |
| NM_031286 | SH3BGRL3 | -0.03 | NM_032348 | MGC3047 | -0.03 |

| | | | | | |
|---|---|---|---|---|---|
| NM_080861 | SSB3 | -0.03 | NM_033411 | RWDD2 | -0.03 |
| NM_080667 | MGC15407 | -0.03 | NM_033386 | MIRAB13 | -0.03 |
| NM_144567 | LOC90806 | -0.03 | NM_145042 | MGC16703 | -0.03 |
| NM_033191 | KRTAP9-4 | -0.03 | NM_145037 | MGC15606 | -0.03 |
| NM_052892 | PKD1L2 | -0.03 | NM_052961 | SLC26A8 | -0.03 |
| NM_144593 | RHEBL1 | -0.03 | NM_182970 | RIMS4 | -0.03 |
| NM_080874 | ASB5 | -0.03 | NM_024087 | ASB9 | -0.03 |
| NM_173795 | FLJ32096 | -0.03 | NM_174912 | FLJ31204 | -0.03 |
| NM_174901 | FAM9C | -0.03 | NM_181712 | LOC163782 | -0.03 |
| NM_152630 | MGC26999 | -0.03 | XM_376307 | DKFZP667E0512 | -0.03 |
| NM_152689 | MGC9712 | -0.03 | NM_182537 | HTR3D | -0.03 |
| NM_174928 | LOC221143 | -0.03 | XM_378822 | LOC254099 | -0.03 |
| NM_172140 | IL29 | -0.03 | NM_173587 | RCOR2 | -0.03 |
| NM_176885 | TAS2R44 | -0.03 | NM_176889 | TAS2R49 | -0.03 |
| NM_178863 | KCTD13 | -0.03 | NM_153488 | MAGEA2B | -0.03 |
| NM_207401 | FLJ45717 | -0.03 | NM_198476 | FLJ41131 | -0.03 |
| XM_378589 | LOC283914 | -0.03 | XM_376111 | LOC285189 | -0.03 |
| XM_375500 | MGC16597 | -0.03 | NM_175064 | WBSCR19 | -0.03 |
| NM_198514 | NHLRC2 | -0.03 | XM_114047 | LOC199882 | -0.03 |
| NM_138699 | LOC93622 | -0.03 | NM_207463 | FLJ46230 | -0.03 |
| XM_067448 | LOC131572 | -0.03 | XM_063630 | LOC123397 | -0.03 |
| XM_060952 | LOC128367 | -0.03 | XM_291704 | LOC340765 | -0.03 |
| XM_378880 | LOC400764 | -0.03 | XM_372785 | LOC391062 | -0.03 |
| XM_371674 | LOC389170 | -0.03 | XM_379263 | LOC401126 | -0.03 |
| XM_377945 | LOC402279 | -0.03 | XM_380057 | LOC402709 | -0.03 |
| XM_373311 | LOC392382 | -0.03 | XM_373509 | LOC387789 | -0.03 |
| XM_373518 | LOC387821 | -0.03 | XM_370631 | LOC387778 | -0.03 |
| XM_373569 | LOC387937 | -0.03 | XM_372412 | LOC390197 | -0.03 |
| XM_377446 | LOC401861 | -0.03 | XM_371016 | LOC388335 | -0.03 |
| XM_371145 | LOC388514 | -0.03 | NM_005665 | EVI5 | -0.03 |
| NM_144610 | FLJ25006 | -0.02 | NM_000907 | NPR2 | -0.02 |
| NM_020168 | PAK6 | -0.02 | NM_002738 | PRKCB1 | -0.02 |
| NM_025144 | LAK | -0.02 | NM_031480 | RIOK1 | -0.02 |
| NM_030901 | OR7A17 | -0.02 | NM_000524 | HTR1A | -0.02 |
| NM_002924 | RGS7 | -0.02 | NM_004778 | GPR44 | -0.02 |
| NM_002239 | KCNJ3 | -0.02 | NM_021153 | CDH19 | -0.02 |
| NM_003633 | ENC1 | -0.02 | NM_005041 | PRF1 | -0.02 |
| NM_012334 | MYO10 | -0.02 | NM_002999 | SDC4 | -0.02 |
| NM_003174 | SVIL | -0.02 | NM_014795 | ZFHX1B | -0.02 |
| NM_018416 | FOXJ2 | -0.02 | NM_001537 | HSBP1 | -0.02 |
| NM_012258 | HEY1 | -0.02 | NM_005680 | TAF1B | -0.02 |
| NM_003214 | TEAD3 | -0.02 | NM_000481 | AMT | -0.02 |
| NM_007114 | TMF1 | -0.02 | NM_000170 | GLDC | -0.02 |
| NM_000922 | PDE3B | -0.02 | NM_017813 | FLJ20421 | -0.02 |
| NM_016640 | MRPS30 | -0.02 | NM_004536 | BIRC1 | -0.02 |
| NM_006659 | TUBGCP2 | -0.02 | NM_005019 | PDE1A | -0.02 |

| | | | | | |
|---|---|---|---|---|---|
| NM_031208 | DKFZP566J2046 | -0.02 | NM_015984 | UCHL5 | -0.02 |
| NM_005180 | BMI1 | -0.02 | NM_174916 | UBR1 | -0.02 |
| NM_000949 | PRLR | -0.02 | NM_014885 | ANAPC10 | -0.02 |
| NM_000163 | GHR | -0.02 | NM_005259 | GDF8 | -0.02 |
| NM_002172 | IFNA14 | -0.02 | NM_002195 | INSL4 | -0.02 |
| NM_177959 | DOK5 | -0.02 | NM_005059 | RLN2 | -0.02 |
| NM_004107 | FCGRT | -0.02 | NM_012156 | EPB41L1 | -0.02 |
| NM_002275 | KRT15 | -0.02 | NM_002234 | KCNA5 | -0.02 |
| NM_001850 | COL8A1 | -0.02 | NM_152520 | ZNF533 | -0.02 |
| NM_000583 | GC | -0.02 | NM_005143 | HP | -0.02 |
| NM_014154 | HSPC056 | -0.02 | NM_032483 | HTPAP | -0.02 |
| NM_001722 | POLR3D | -0.02 | NM_018556 | SIRPB2 | -0.02 |
| NM_002966 | S100A10 | -0.02 | NM_005052 | RAC3 | -0.02 |
| NM_012236 | SCMH1 | -0.02 | NM_004069 | AP2S1 | -0.02 |
| NM_004045 | ATOX1 | -0.02 | NM_002223 | ITPR2 | -0.02 |
| NM_002277 | KRTHA1 | -0.02 | NM_004561 | OVOL1 | -0.02 |
| NM_002489 | NDUFA4 | -0.02 | NM_002640 | SERPINB8 | -0.02 |
| NM_000983 | RPL22 | -0.02 | NM_002854 | PVALB | -0.02 |
| NM_003359 | UGDH | -0.02 | NM_003455 | ZNF202 | -0.02 |
| NM_021140 | UTX | -0.02 | NM_003725 | RODH | -0.02 |
| XM_032901 | KIAA0226 | -0.02 | XM_375397 | KIAA0753 | -0.02 |
| NM_014820 | TOMM70A | -0.02 | NM_006994 | BTN3A3 | -0.02 |
| NM_014884 | SFRS14 | -0.02 | NM_005785 | RNF41 | -0.02 |
| NM_006337 | MCRS1 | -0.02 | NM_005819 | STX6 | -0.02 |
| NM_006815 | RNP24 | -0.02 | NM_006397 | RNASEH2A | -0.02 |
| NM_007083 | NUDT6 | -0.02 | NM_006841 | SLC38A3 | -0.02 |
| NM_006700 | FLN29 | -0.02 | NM_015589 | SAMD4 | -0.02 |
| NM_014952 | BAHD1 | -0.02 | XM_028253 | C19ORF7 | -0.02 |
| NM_014319 | MAN1 | -0.02 | NM_032012 | C9ORF5 | -0.02 |
| NM_016454 | LOC51234 | -0.02 | NM_016009 | SH3GLB1 | -0.02 |
| NM_016185 | HN1 | -0.02 | NM_016256 | NAGPA | -0.02 |
| NM_021155 | CD209 | -0.02 | NM_016353 | ZDHHC2 | -0.02 |
| NM_017891 | FLJ20584 | -0.02 | NM_017899 | TSC | -0.02 |
| NM_017928 | FLJ20694 | -0.02 | NM_017697 | FLJ20171 | -0.02 |
| NM_017853 | DLP | -0.02 | NM_017855 | APIN | -0.02 |
| NM_017856 | FLJ20514 | -0.02 | NM_018362 | LIN7C | -0.02 |
| NM_018272 | CASC1 | -0.02 | NM_018708 | FEM1A | -0.02 |
| NM_018430 | TSNAXIP1 | -0.02 | NM_018006 | TRMT1 | -0.02 |
| NM_018467 | MDS032 | -0.02 | NM_020420 | DAZ4 | -0.02 |
| NM_020696 | KIAA1143 | -0.02 | XM_371953 | KIAA1466 | -0.02 |
| NM_021807 | SEC8L1 | -0.02 | NM_020868 | DPP10 | -0.02 |
| NM_021826 | FLJ13149 | -0.02 | NM_022912 | C2ORF23 | -0.02 |
| NM_024620 | FLJ12586 | -0.02 | NM_024636 | TNFAIP9 | -0.02 |
| NM_024522 | FLJ12650 | -0.02 | NM_024523 | GCC1 | -0.02 |
| NM_199052 | C20ORF7 | -0.02 | NM_024683 | FLJ22729 | -0.02 |
| NM_024590 | FLJ23548 | -0.02 | NM_024697 | FLJ22419 | -0.02 |

| | | | | | |
|---|---|---|---|---|---|
| NM_024958 | C20ORF98 | -0.02 | NM_031302 | LOC83468 | -0.02 |
| NM_032148 | SLC41A2 | -0.02 | NM_031935 | FIBL-6 | -0.02 |
| NM_031949 | TTLL2 | -0.02 | NM_032025 | EIF2A | -0.02 |
| NM_032250 | ANKRD20A | -0.02 | NM_032255 | ZNF541 | -0.02 |
| NM_032410 | HOOK3 | -0.02 | NM_032308 | MGC4189 | -0.02 |
| NM_032313 | C4ORF14 | -0.02 | NM_032842 | FLJ14803 | -0.02 |
| NM_032382 | COG8 | -0.02 | NM_032563 | LCE3D | -0.02 |
| NM_032933 | MGC11386 | -0.02 | NM_130446 | KLHL6 | -0.02 |
| NM_138357 | C10ORF42 | -0.02 | NM_153333 | MGC45400 | -0.02 |
| NM_138346 | MGC33867 | -0.02 | NM_052896 | CSMD2 | -0.02 |
| NM_052899 | KIAA1893 | -0.02 | NM_052923 | ZNF452 | -0.02 |
| NM_138441 | C6ORF150 | -0.02 | NM_080927 | DCBLD2 | -0.02 |
| NM_138335 | GNPDA2 | -0.02 | NM_144621 | ZBTB8 | -0.02 |
| NM_152417 | FLJ32370 | -0.02 | XM_085689 | KCTD11 | -0.02 |
| NM_178477 | C20ORF179 | -0.02 | NM_080552 | VIAAT | -0.02 |
| NM_032552 | DAB2IP | -0.02 | NM_144713 | FLJ32954 | -0.02 |
| NM_145286 | STOML3 | -0.02 | NM_144978 | FLJ32745 | -0.02 |
| NM_152728 | C18ORF20 | -0.02 | NM_173799 | FLJ39873 | -0.02 |
| NM_152709 | C10ORF24 | -0.02 | NM_152710 | C10ORF27 | -0.02 |
| NM_153325 | DEFB125 | -0.02 | NM_182551 | UNQ1849 | -0.02 |
| NM_032158 | WBSCR20C | -0.02 | XM_211858 | LOC285359 | -0.02 |
| NM_182594 | ZNF454 | -0.02 | XM_379074 | LOC339788 | -0.02 |
| XM_292765 | ZNF404 | -0.02 | NM_178557 | FLJ37478 | -0.02 |
| NM_197965 | SOAT | -0.02 | NM_201547 | C4ORF11 | -0.02 |
| NM_181727 | SPATA12 | -0.02 | NM_178233 | OTOP3 | -0.02 |
| NM_207368 | LOC348262 | -0.02 | NM_182627 | MGC64882 | -0.02 |
| NM_201402 | DUB3 | -0.02 | NM_207466 | FLJ46489 | -0.02 |
| XM_117152 | LOC199897 | -0.02 | NM_207511 | FLJ36268 | -0.02 |
| NM_207512 | LOC401610 | -0.02 | NM_205857 | FBI4 | -0.02 |
| XM_294778 | LOC339025 | -0.02 | XM_068889 | LOC134505 | -0.02 |
| XM_061626 | LOC119694 | -0.02 | XM_294311 | LOC346521 | -0.02 |
| XM_166916 | LOC219958 | -0.02 | XM_379191 | LOC401065 | -0.02 |
| XM_374028 | LOC389087 | -0.02 | XM_373974 | LOC388924 | -0.02 |
| XM_376267 | LOC401087 | -0.02 | XM_376051 | LOC400952 | -0.02 |
| XM_371845 | LOC389427 | -0.02 | XM_372097 | LOC389734 | -0.02 |
| XM_380036 | LOC402689 | -0.02 | XM_379851 | LOC402525 | -0.02 |
| XM_374965 | LOC400010 | -0.02 | XM_372303 | LOC389936 | -0.02 |
| XM_377343 | LOC401778 | -0.02 | XM_374885 | LOC399898 | -0.02 |
| XM_378360 | LOC400043 | -0.02 | XM_378660 | LOC400584 | -0.02 |
| XM_377426 | LOC401845 | -0.02 | XM_377529 | LOC401915 | -0.02 |
| XM_371125 | LOC388491 | -0.02 | XM_373688 | LOC388279 | -0.02 |
| NM_001004335 | FLJ42842 | -0.02 | NM_001004349 | FLJ45422 | -0.02 |
| XM_379730 | LOC401621 | -0.02 | NM_001005512 | OR4A47 | -0.02 |
| NM_001277 | CHKA | -0.01 | NM_018423 | STYK1 | -0.01 |
| NM_031965 | GSG2 | -0.01 | NM_139158 | ALS2CR7 | -0.01 |
| NM_002969 | MAPK12 | -0.01 | XM_166086 | NRG3 | -0.01 |

| | | | | | |
|---|---|---|---|---|---|
| NM_002529 | NTRK1 | -0.01 | NM_005456 | MAPK8IP1 | -0.01 |
| NM_032017 | MGC4796 | -0.01 | NM_002610 | PDK1 | -0.01 |
| NM_005374 | MPP2 | -0.01 | NM_005044 | PRKX | -0.01 |
| NM_005965 | MYLK | -0.01 | NM_006609 | MAP3K2 | -0.01 |
| NM_002929 | GRK1 | -0.01 | NM_005012 | ROR1 | -0.01 |
| NM_004054 | C3AR1 | -0.01 | NM_004367 | CCR6 | -0.01 |
| NM_030906 | STK33 | -0.01 | NM_006056 | NMUR1 | -0.01 |
| NM_002928 | RGS16 | -0.01 | NM_020633 | VN1R1 | -0.01 |
| NM_080879 | RAB40A | -0.01 | NM_004973 | JARID2 | -0.01 |
| NM_003299 | TRA1 | -0.01 | NM_001208 | BTF3L1 | -0.01 |
| NM_000638 | VTN | -0.01 | NM_006276 | SFRS7 | -0.01 |
| NM_005442 | EOMES | -0.01 | NM_014727 | MLL4 | -0.01 |
| NM_005378 | MYCN | -0.01 | NM_022716 | PRRX1 | -0.01 |
| NM_005859 | PURA | -0.01 | NM_003998 | NFKB1 | -0.01 |
| NM_000451 | SHOX | -0.01 | NM_002509 | NKX2-2 | -0.01 |
| NM_004387 | NKX2-5 | -0.01 | NM_006339 | HMG20B | -0.01 |
| NM_003079 | SMARCE1 | -0.01 | NM_003131 | SRF | -0.01 |
| NM_003201 | TFAM | -0.01 | NM_001911 | CTSG | -0.01 |
| NM_003597 | TIEG2 | -0.01 | NM_000157 | GBA | -0.01 |
| NM_015013 | AOF2 | -0.01 | NM_001935 | DPP4 | -0.01 |
| NM_012203 | GRHPR | -0.01 | NM_000923 | PDE4C | -0.01 |
| NM_015922 | NSDHL | -0.01 | NM_000137 | FAH | -0.01 |
| NM_004110 | FDXR | -0.01 | NM_000150 | FUT6 | -0.01 |
| NM_016335 | PRODH | -0.01 | NM_014243 | ADAMTS3 | -0.01 |
| NM_003352 | SUMO1 | -0.01 | NM_003883 | HDAC3 | -0.01 |
| NM_000531 | OTC | -0.01 | NM_020216 | RNPEP | -0.01 |
| NM_005914 | MCM4 | -0.01 | NM_019555 | ARHGEF3 | -0.01 |
| NM_004799 | ZFYVE9 | -0.01 | NM_030666 | SERPINB1 | -0.01 |
| NM_006217 | SERPINI2 | -0.01 | NM_016263 | FZR1 | -0.01 |
| NM_178422 | MPRA | -0.01 | NM_000826 | GRIA2 | -0.01 |
| NM_014439 | IL1F7 | -0.01 | XM_167711 | ITGA8 | -0.01 |
| NM_000479 | AMH | -0.01 | NM_000641 | IL11 | -0.01 |
| NM_002908 | REL | -0.01 | NM_002855 | PVRL1 | -0.01 |
| NM_024408 | NOTCH2 | -0.01 | NM_002312 | LIG4 | -0.01 |
| NM_032737 | LMNB2 | -0.01 | NM_153212 | GJB4 | -0.01 |
| NM_001514 | GTF2B | -0.01 | NM_015250 | BICD2 | -0.01 |
| NM_000355 | TCN2 | -0.01 | NM_002249 | KCNN3 | -0.01 |
| NM_001660 | ARF4 | -0.01 | NM_014629 | ARHGEF10 | -0.01 |
| NM_024997 | ATF7IP2 | -0.01 | NM_006356 | ATP5H | -0.01 |
| NM_015526 | CLIPR-59 | -0.01 | NM_001920 | DCN | -0.01 |
| NM_014503 | DRIM | -0.01 | NM_024733 | FLJ14345 | -0.01 |
| NM_005828 | HAN11 | -0.01 | NM_002025 | FMR2 | -0.01 |
| NM_005479 | FRAT1 | -0.01 | NM_000158 | GBE1 | -0.01 |
| NM_014380 | NGFRAP1 | -0.01 | NM_014625 | NPHS2 | -0.01 |
| NM_005824 | LRRC17 | -0.01 | NM_014061 | MAGEH1 | -0.01 |
| NM_000430 | PAFAH1B1 | -0.01 | NM_006253 | PRKAB1 | -0.01 |

| | | | | | |
|---|---|---|---|---|---|
| NM_000542 | SFTPB | -0.01 | NM_003076 | SMARCD1 | -0.01 |
| NM_001276 | CHI3L1 | -0.01 | NM_001584 | C11ORF8 | -0.01 |
| NM_001739 | CA5A | -0.01 | NM_004341 | CAD | -0.01 |
| NM_005170 | ASCL2 | -0.01 | NM_152296 | ATP1A3 | -0.01 |
| NM_001751 | CARS | -0.01 | NM_001323 | CST6 | -0.01 |
| NM_001187 | BAGE | -0.01 | NM_006736 | DNAJB2 | -0.01 |
| NM_013289 | KIR3DL1 | -0.01 | NM_002363 | MAGEB1 | -0.01 |
| NM_014945 | ABLIM3 | -0.01 | NM_014551 | 384D8-2 | -0.01 |
| NM_016453 | NCKIPSD | -0.01 | NM_018723 | A2BP1 | -0.01 |
| NM_020380 | AF15Q14 | -0.01 | NM_020679 | AD023 | -0.01 |
| NM_006303 | JTV1 | -0.01 | NM_005656 | TMPRSS2 | -0.01 |
| NM_004751 | GCNT3 | -0.01 | NM_004727 | SLC24A1 | -0.01 |
| XM_291018 | USP34 | -0.01 | NM_005750 | C4ORF6 | -0.01 |
| NM_005783 | TXNDC9 | -0.01 | NM_006804 | STARD3 | -0.01 |
| NM_006632 | SLC17A3 | -0.01 | NM_006693 | CPSF4 | -0.01 |
| NM_006707 | BTNL3 | -0.01 | NM_007048 | BTN3A1 | -0.01 |
| XM_290502 | KIAA1030 | -0.01 | XM_042323 | CAMTA1 | -0.01 |
| XM_034086 | KIAA1107 | -0.01 | NM_015171 | XPO6 | -0.01 |
| NM_015184 | PLCL2 | -0.01 | NM_014305 | TGDS | -0.01 |
| NM_015550 | OSBPL3 | -0.01 | NM_012399 | PITPNB | -0.01 |
| NM_020344 | SLC24A2 | -0.01 | NM_015585 | C20ORF26 | -0.01 |
| NM_015668 | DKFZP434I092 | -0.01 | NM_017415 | KLHL3 | -0.01 |
| NM_015685 | SDCBP2 | -0.01 | NM_020727 | ZNF295 | -0.01 |
| NM_016441 | CRIM1 | -0.01 | NM_016184 | CLECSF6 | -0.01 |
| NM_015948 | SLC35B3 | -0.01 | NM_014575 | SCHIP1 | -0.01 |
| NM_016049 | C14ORF122 | -0.01 | NM_014592 | KCNIP1 | -0.01 |
| NM_016299 | HSPA14 | -0.01 | NM_139265 | EHD4 | -0.01 |
| NM_019030 | DHX29 | -0.01 | NM_058182 | C21ORF51 | -0.01 |
| NM_017910 | FLJ20628 | -0.01 | NM_018150 | C1orf164 | -0.01 |
| NM_018189 | DPPA4 | -0.01 | NM_018437 | HEMGN | -0.01 |
| NM_020140 | EB-1 | -0.01 | NM_020154 | C15ORF24 | -0.01 |
| NM_020116 | FSTL5 | -0.01 | NM_020762 | SRGAP1 | -0.01 |
| NM_021202 | TP53INP2 | -0.01 | NM_020899 | ZBTB4 | -0.01 |
| XM_052561 | KIAA1337 | -0.01 | NM_021213 | PCTP | -0.01 |
| NM_021947 | SRR | -0.01 | NM_022082 | C20ORF59 | -0.01 |
| NM_201280 | MUTED | -0.01 | NM_023014 | LOC65122 | -0.01 |
| NM_022106 | C20ORF177 | -0.01 | NM_023071 | SPATS2 | -0.01 |
| NM_022142 | ELSPBP1 | -0.01 | NM_024108 | MGC2650 | -0.01 |
| NM_024773 | FLJ13798 | -0.01 | XM_370863 | ATP8B4 | -0.01 |
| NM_024994 | FLJ12595 | -0.01 | NM_025042 | WBSCR23 | -0.01 |
| NM_024886 | C10ORF95 | -0.01 | NM_025054 | VCIP135 | -0.01 |
| NM_030795 | STMN4 | -0.01 | NM_030926 | ITM2C | -0.01 |
| NM_030929 | KAZALD1 | -0.01 | NM_031945 | OCSP | -0.01 |
| NM_032276 | DKFZP547E052 | -0.01 | NM_032339 | C17ORF37 | -0.01 |
| XM_378193 | MGC10981 | -0.01 | NM_153752 | C21ORF84 | -0.01 |
| NM_017528 | WBSCR22 | -0.01 | NM_147190 | LASS5 | -0.01 |

| | | |
|---|---|---|
| NM_053051 | LIP8 | -0.01 |
| NM_178454 | MGC54289 | -0.01 |
| NM_145258 | MGC22773 | -0.01 |
| NM_207321 | C10ORF129 | -0.01 |
| NM_145270 | LOC146325 | -0.01 |
| NM_181644 | DKFZP761N1114 | -0.01 |
| NM_153008 | FLJ30277 | -0.01 |
| NM_206858 | MGC87149 | -0.01 |
| NM_007139 | ZNF92 | -0.01 |
| XM_352903 | FLJ30092 | -0.01 |
| NM_139249 | MS4A6E | -0.01 |
| NM_032251 | FLJ37970 | -0.01 |
| NM_153252 | BRODL | -0.01 |
| NM_173610 | FLJ33768 | -0.01 |
| NM_178532 | LOC285671 | -0.01 |
| NM_198154 | LOC339168 | -0.01 |
| NM_181605 | KRTAP6-3 | -0.01 |
| XM_375029 | LOC338862 | -0.01 |
| NM_178135 | SCDR9 | -0.01 |
| NM_182703 | LOC348094 | -0.01 |
| NM_207483 | FLJ45964 | -0.01 |
| NM_207497 | FLJ43752 | -0.01 |
| NM_207444 | FLJ35695 | -0.01 |
| XM_086604 | LOC149620 | -0.01 |
| XM_058719 | LOC123688 | -0.01 |
| XM_209187 | LOC284417 | -0.01 |
| XM_059830 | LOC136242 | -0.01 |
| XM_166869 | LOC219429 | -0.01 |
| XM_379156 | LOC401032 | -0.01 |
| XM_371503 | LOC388963 | -0.01 |
| XM_372779 | LOC391040 | -0.01 |
| XM_371569 | LOC389049 | -0.01 |
| XM_374051 | LOC389149 | -0.01 |
| XM_373058 | LOC391746 | -0.01 |
| XM_374096 | LOC389243 | -0.01 |
| XM_377878 | LOC402201 | -0.01 |
| XM_380135 | LOC402578 | -0.01 |
| XM_373301 | LOC392352 | -0.01 |
| XM_372447 | LOC390282 | -0.01 |
| XM_378623 | LOC400552 | -0.01 |
| XM_370984 | LOC388284 | -0.01 |
| XM_375931 | LOC400858 | -0.01 |
| XM_379065 | LOC400933 | -0.01 |
| NM_001006118 | RBMY1E | -0.01 |
| NM_001827 | CKS2 | 0 |
| NM_015981 | CAMK2A | 0 |
| NM_198447 | FLJ42654 | -0.01 |
| NM_178456 | C20ORF85 | -0.01 |
| XM_060509 | S100A7L2 | -0.01 |
| NM_144673 | CKLFSF2 | -0.01 |
| NM_022343 | C9ORF19 | -0.01 |
| NM_152557 | FLJ31413 | -0.01 |
| XM_377742 | FBXO41 | -0.01 |
| NM_198457 | ZNF600 | -0.01 |
| NM_178502 | DTX3 | -0.01 |
| NM_173550 | C9ORF93 | -0.01 |
| NM_145043 | NEIL2 | -0.01 |
| NM_152774 | MGC42090 | -0.01 |
| NM_012232 | PTRF | -0.01 |
| NM_178530 | FLJ38379 | -0.01 |
| NM_178534 | FLJ37940 | -0.01 |
| XM_371952 | LOC340351 | -0.01 |
| NM_183378 | OVCH1 | -0.01 |
| XM_291638 | LOC343070 | -0.01 |
| NM_198924 | MGC45477 | -0.01 |
| NM_207481 | FLJ34870 | -0.01 |
| NM_201520 | FLJ40217 | -0.01 |
| NM_203412 | LOC164153 | -0.01 |
| NM_207513 | LOC404785 | -0.01 |
| XM_291584 | LOC343474 | -0.01 |
| XM_171855 | LOC255275 | -0.01 |
| XM_071099 | LOC138805 | -0.01 |
| XM_372812 | LOC391162 | -0.01 |
| XM_372784 | LOC391059 | -0.01 |
| XM_373817 | LOC388577 | -0.01 |
| XM_371253 | LOC388633 | -0.01 |
| XM_371641 | LOC389120 | -0.01 |
| XM_377755 | LOC402094 | -0.01 |
| XM_374170 | LOC389398 | -0.01 |
| XM_379484 | LOC401324 | -0.01 |
| XM_377934 | LOC402252 | -0.01 |
| XM_374108 | LOC389264 | -0.01 |
| XM_374281 | LOC389687 | -0.01 |
| XM_370557 | LOC387676 | -0.01 |
| XM_378538 | LOC400433 | -0.01 |
| XM_378703 | LOC400619 | -0.01 |
| XM_372643 | LOC390738 | -0.01 |
| XM_372200 | LOC389842 | -0.01 |
| NM_001003702 | FLJ43692 | -0.01 |
| NM_000061 | BTK | 0 |
| NM_145025 | C6ORF199 | 0 |
| NM_003648 | DGKD | 0 |

| | | |
|---|---|---|
| NM_152649 | FLJ34389 | 0 |
| NM_003674 | CDK10 | 0 |
| NM_003640 | IKBKAP | 0 |
| NM_003551 | NME5 | 0 |
| NM_012324 | MAPK8IP2 | 0 |
| NM_145203 | CSNK1A1L | 0 |
| NM_003157 | NEK4 | 0 |
| NM_000680 | ADRA1A | 0 |
| NM_004230 | EDG5 | 0 |
| NM_003844 | TNFRSF10A | 0 |
| NM_013257 | SGKL | 0 |
| NM_005108 | XYLB | 0 |
| NM_001470 | GABBR1 | 0 |
| NM_003957 | STK29 | 0 |
| NM_015234 | GPR116 | 0 |
| NM_016602 | GPR2 | 0 |
| NM_005299 | GPR31 | 0 |
| NM_001843 | CNTN1 | 0 |
| NM_007059 | KPTN | 0 |
| NM_005348 | HSPCA | 0 |
| NM_004768 | SFRS11 | 0 |
| NM_006872 | ALF | 0 |
| NM_000484 | APP | 0 |
| NM_005238 | ETS1 | 0 |
| NM_000325 | PITX2 | 0 |
| NM_002145 | HOXB2 | 0 |
| NM_005635 | SSX1 | 0 |
| NM_003403 | YY1 | 0 |
| NM_000666 | ACY1 | 0 |
| NM_018427 | RRN3 | 0 |
| NM_000770 | CYP2C8 | 0 |
| NM_020676 | ABHD6 | 0 |
| NM_000925 | PDHB | 0 |
| NM_023079 | FLJ13855 | 0 |
| NM_005133 | RCE1 | 0 |
| NM_007274 | BACH | 0 |
| NM_032043 | BRIP1 | 0 |
| NM_001271 | CHD2 | 0 |
| NM_001061 | TBXAS1 | 0 |
| NM_007112 | THBS3 | 0 |
| NM_012478 | WBP2 | 0 |
| NM_002053 | GBP1 | 0 |
| NM_004152 | OAZ1 | 0 |
| NM_005732 | RAD50 | 0 |
| NM_006267 | RANBP2 | 0 |
| NM_005063 | SCD | 0 |
| NM_003718 | CDC2L5 | 0 |
| NM_003160 | AURKC | 0 |
| NM_033116 | NEK9 | 0 |
| NM_006556 | PMVK | 0 |
| NM_020804 | PACSIN1 | 0 |
| NM_002497 | NEK2 | 0 |
| NM_013254 | TBK1 | 0 |
| NM_003263 | TLR1 | 0 |
| NM_001957 | EDNRA | 0 |
| XM_371262 | ELTD1 | 0 |
| NM_017859 | URKL1 | 0 |
| NM_003405 | YWHAH | 0 |
| NM_003857 | GALR2 | 0 |
| NM_052847 | GNG7 | 0 |
| NM_020455 | GPR126 | 0 |
| NM_007232 | HRH3 | 0 |
| NM_006028 | HTR3B | 0 |
| NM_001553 | IGFBP7 | 0 |
| NM_002098 | GUCA1B | 0 |
| NM_007127 | VIL1 | 0 |
| NM_005428 | VAV1 | 0 |
| NM_015975 | TAF9L | 0 |
| NM_018489 | ASH1L | 0 |
| NM_001986 | ETV4 | 0 |
| NM_003865 | HESX1 | 0 |
| NM_002146 | HOXB3 | 0 |
| NM_001609 | ACADSB | 0 |
| NM_001227 | CASP7 | 0 |
| NM_004661 | CDC23 | 0 |
| NM_007022 | CYB561D2 | 0 |
| NM_139214 | TGIF2LY | 0 |
| NM_022126 | LHPP | 0 |
| NM_002402 | MEST | 0 |
| NM_001536 | HRMT1L2 | 0 |
| NM_000050 | ASS | 0 |
| NM_003287 | TPD52L1 | 0 |
| NM_000543 | SMPD1 | 0 |
| XM_496819 | TBX18 | 0 |
| NM_000669 | ADH1C | 0 |
| NM_006288 | THY1 | 0 |
| NM_032780 | TMEM25 | 0 |
| NM_024827 | HDAC11 | 0 |
| NM_002853 | RAD1 | 0 |
| NM_014214 | IMPA2 | 0 |
| NM_002606 | PDE9A | 0 |
| NM_006515 | SETMAR | 0 |

| | | | | | |
|---|---|---|---|---|---|
| NM_005021 | ENPP3 | 0 | NM_015915 | SPG3A | 0 |
| NM_012444 | SPO11 | 0 | NM_000400 | ERCC2 | 0 |
| NM_005646 | TARBP1 | 0 | NM_020998 | MST1 | 0 |
| XM_033391 | PPP1R3E | 0 | NM_001069 | TUBB | 0 |
| NM_004840 | ARHGEF6 | 0 | NM_014889 | PITRM1 | 0 |
| NM_007038 | ADAMTS5 | 0 | NM_016339 | RAPGEFL1 | 0 |
| XM_087254 | ATP11B | 0 | NM_005746 | PBEF1 | 0 |
| NM_005006 | NDUFS1 | 0 | NM_006534 | NCOA3 | 0 |
| NM_001166 | BIRC2 | 0 | NM_005762 | TRIM28 | 0 |
| NM_004562 | PARK2 | 0 | NM_005879 | TRIP | 0 |
| NM_007014 | WWP2 | 0 | NM_012168 | FBXO2 | 0 |
| NM_012170 | FBXO22 | 0 | NM_020892 | DTX2 | 0 |
| NM_001732 | BTN1A1 | 0 | NM_016372 | TPRA40 | 0 |
| NM_016364 | DUSP13 | 0 | NM_000510 | FSHB | 0 |
| NM_003854 | IL1RL2 | 0 | NM_005260 | GDF9 | 0 |
| NM_032871 | TNFRSF19L | 0 | NM_001819 | CHGB | 0 |
| NM_014376 | CYFIP2 | 0 | NM_001657 | AREG | 0 |
| NM_002391 | MDK | 0 | NM_007117 | TRH | 0 |
| NM_003377 | VEGFB | 0 | NM_021783 | XEDAR | 0 |
| NM_001405 | EFNA2 | 0 | NM_013251 | TAC3 | 0 |
| NM_006119 | FGF8 | 0 | NM_000461 | THRB | 0 |
| NM_002246 | KCNK3 | 0 | NM_004700 | KCNQ4 | 0 |
| XM_029962 | KCNT1 | 0 | NM_005984 | SLC25A1 | 0 |
| NM_152388 | ALS2CR4 | 0 | NM_005135 | SLC12A6 | 0 |
| NM_015865 | SLC14A1 | 0 | NM_001086 | AADAC | 0 |
| NM_003500 | ACOX2 | 0 | NM_005168 | ARHE | 0 |
| NM_000927 | ABCB1 | 0 | NM_000671 | ADH5 | 0 |
| NM_001791 | CDC42 | 0 | NM_007061 | CDC42EP1 | 0 |
| NM_000781 | CYP11A1 | 0 | NM_032490 | C14ORF142 | 0 |
| NM_014501 | UBE2S | 0 | NM_004091 | E2F2 | 0 |
| NM_001950 | E2F4 | 0 | NM_024585 | FLJ22160 | 0 |
| NM_152579 | FLJ38564 | 0 | NM_004960 | FUS | 0 |
| NM_032827 | ATOH8 | 0 | NM_007035 | KERA | 0 |
| NM_004461 | FARSLA | 0 | NM_000422 | KRT17 | 0 |
| NM_006165 | NFRKB | 0 | NM_004813 | PEX16 | 0 |
| NM_145252 | LOC124220 | 0 | BC052374 | LOC253039 | 0 |
| NM_138770 | LOC90557 | 0 | NM_006222 | PIN1L | 0 |
| NM_006224 | PITPN | 0 | NM_012267 | HSPBP1 | 0 |
| NM_015541 | LRIG1 | 0 | NM_014308 | P101-PI3K | 0 |
| NM_004575 | POU4F2 | 0 | NM_022136 | SAMSN1 | 0 |
| NM_018262 | WDR10 | 0 | NM_005175 | ATP5G1 | 0 |
| NM_001283 | AP1S1 | 0 | NM_001585 | C22ORF1 | 0 |
| NM_005182 | CA7 | 0 | XM_114735 | CR1L | 0 |
| NM_001677 | ATP1B1 | 0 | NM_001327 | CTAG1B | 0 |
| XM_088636 | CYLC1 | 0 | NM_001971 | ELA1 | 0 |
| NM_004434 | EML1 | 0 | NM_000774 | CYP2F1 | 0 |

| | | | | | |
|---|---|---|---|---|---|
| NM_001555 | IGSF1 | 0 | NM_181501 | ITGA1 | 0 |
| NM_001472 | GAGE2 | 0 | NM_021065 | HIST1H2AD | 0 |
| NM_021062 | HIST1H2BB | 0 | NM_002259 | KLRC1 | 0 |
| NM_001383 | DPH2L1 | 0 | NM_001386 | DPYSL2 | 0 |
| NM_005895 | GOLGA3 | 0 | NM_002081 | GPC1 | 0 |
| NM_013402 | FADS1 | 0 | NM_004998 | MYO1E | 0 |
| NM_006250 | PRH1 | 0 | NM_005907 | MAN1A1 | 0 |
| NM_002380 | MATN2 | 0 | NM_080283 | ABCA9 | 0 |
| NM_005917 | MDH1 | 0 | NM_012089 | ABCB10 | 0 |
| NM_002453 | MTIF2 | 0 | NM_173712 | MTND4L | 0 |
| NM_006278 | SIAT4C | 0 | NM_002896 | RBM4 | 0 |
| NM_021136 | RTN1 | 0 | NM_003081 | SNAP25 | 0 |
| NM_006751 | SSFA2 | 0 | NM_006299 | ZNF193 | 0 |
| NM_003165 | STXBP1 | 0 | NM_014242 | ZNF237 | 0 |
| NM_003678 | C22ORF19 | 0 | NM_003683 | D21S2056E | 0 |
| NM_004781 | VAMP3 | 0 | NM_004907 | IER2 | 0 |
| NM_004200 | SYT7 | 0 | NM_004674 | ASH2L | 0 |
| NM_004252 | SLC9A3R1 | 0 | NM_004796 | NRXN3 | 0 |
| NM_004258 | IGSF2 | 0 | NM_005455 | ZNF265 | 0 |
| NM_004696 | SLC16A4 | 0 | NM_004267 | CHST2 | 0 |
| NM_004867 | ITM2A | 0 | NM_004273 | CHST3 | 0 |
| NM_003860 | BANF1 | 0 | NM_014783 | ARHGAP11A | 0 |
| NM_005839 | SRRM1 | 0 | NM_014688 | USP6NL | 0 |
| NM_021647 | KIAA0626 | 0 | NM_005492 | CST8 | 0 |
| NM_005495 | SLC17A4 | 0 | NM_014704 | KIAA0562 | 0 |
| NM_016134 | PGCP | 0 | NM_014854 | SLC35E2 | 0 |
| NM_006108 | SPON1 | 0 | NM_006324 | CFDP1 | 0 |
| NM_006338 | LRRN5 | 0 | NM_006605 | RFPL2 | 0 |
| NM_006401 | ANP32B | 0 | NM_006643 | SDCCAG3 | 0 |
| NM_006414 | RPP38 | 0 | NM_007211 | C12ORF2 | 0 |
| NM_006464 | TGOLN2 | 0 | NM_006555 | YKT6 | 0 |
| NM_014974 | KIAA0934 | 0 | XM_290758 | KIAA0553 | 0 |
| NM_007350 | PHLDA1 | 0 | NM_014892 | RBM16 | 0 |
| NM_015259 | ICOSL | 0 | NM_015050 | KIAA0082 | 0 |
| XM_045423 | KIAA0701 | 0 | NM_015288 | PHF15 | 0 |
| XM_290598 | DDN | 0 | NM_012242 | DKK1 | 0 |
| NM_012341 | GTPBP4 | 0 | NM_015371 | HS322B1A | 0 |
| NM_015373 | C22ORF2 | 0 | NM_015471 | DC8 | 0 |
| NM_015474 | SAMHD1 | 0 | NM_015332 | KIAA1068 | 0 |
| NM_015340 | LARS2 | 0 | NM_015901 | NUDT13 | 0 |
| NM_012239 | SIRT3 | 0 | NM_012264 | C22ORF5 | 0 |
| NM_014034 | ASF1A | 0 | NM_014331 | SLC7A11 | 0 |
| NM_015404 | DFNB31 | 0 | NM_015542 | UPF2 | 0 |
| NM_015557 | CHD5 | 0 | NM_014020 | LR8 | 0 |
| NM_018959 | DAZAP1 | 0 | NM_014437 | SLC39A1 | 0 |
| NM_015697 | CL640 | 0 | NM_015701 | C2ORF30 | 0 |

| | | | | | |
|---|---|---|---|---|---|
| NM_014167 | HSPC128 | 0 | NM_014168 | HSPC133 | 0 |
| NM_014398 | LAMP3 | 0 | NM_014410 | CLUL1 | 0 |
| NM_013270 | TSP50 | 0 | NM_015686 | TMEM28 | 0 |
| NM_016459 | PACAP | 0 | NM_015997 | CGI-41 | 0 |
| NM_016565 | E2IG2 | 0 | NM_016119 | PHF11 | 0 |
| NM_014581 | OBP2B | 0 | NM_019060 | NICE-1 | 0 |
| NM_016524 | LOC51760 | 0 | NM_017734 | PALMD | 0 |
| NM_017745 | BCOR | 0 | NM_018058 | CRTAC1 | 0 |
| NM_017644 | DRE1 | 0 | NM_017801 | CKLFSF6 | 0 |
| NM_017805 | RASIP1 | 0 | NM_017933 | FLJ20701 | 0 |
| NM_175748 | C14ORF130 | 0 | NM_017826 | FLJ20449 | 0 |
| NM_018181 | ZNF532 | 0 | NM_018365 | MNS1 | 0 |
| NM_018195 | FLJ10726 | 0 | NM_018378 | FBXL8 | 0 |
| NM_018205 | LRRC20 | 0 | NM_018214 | LRRC1 | 0 |
| NM_018227 | FLJ10808 | 0 | NM_017722 | FLJ20244 | 0 |
| NM_018235 | CNDP2 | 0 | NM_017736 | THUMPD1 | 0 |
| NM_018487 | HCA112 | 0 | NM_018338 | FLJ11142 | 0 |
| NM_018648 | NOLA3 | 0 | NM_018656 | SLC35E3 | 0 |
| NM_018700 | TRIM36 | 0 | NM_018295 | FLJ11000 | 0 |
| NM_017925 | C9ORF55 | 0 | NM_032560 | KIAA2010 | 0 |
| NM_017980 | LIMS2 | 0 | NM_017991 | FLJ10081 | 0 |
| NM_018280 | FLJ10945 | 0 | NM_020660 | CX36 | 0 |
| NM_020152 | C21ORF7 | 0 | NM_019885 | CYP26B1 | 0 |
| XM_371956 | KIAA1549 | 0 | NM_020826 | SYT13 | 0 |
| NM_020830 | WDFY1 | 0 | NM_021245 | MYOZ1 | 0 |
| NM_020954 | KIAA1618 | 0 | XM_046305 | KIAA1205 | 0 |
| NM_020746 | KIAA1271 | 0 | NM_020752 | GPR158 | 0 |
| NM_021184 | C6ORF47 | 0 | NM_021046 | UHSKERB | 0 |
| XM_033173 | PCDH19 | 0 | NM_021927 | FLJ13220 | 0 |
| NM_021931 | DHX35 | 0 | NM_031902 | MRPS5 | 0 |
| NM_022370 | ROBO3 | 0 | NM_022464 | SIL1 | 0 |
| NM_022821 | ELOVL1 | 0 | NM_024053 | C22ORF18 | 0 |
| NM_024339 | MGC2655 | 0 | NM_024755 | FLJ13213 | 0 |
| NM_024554 | PGBD5 | 0 | NM_024558 | C14ORF138 | 0 |
| NM_024574 | FLJ23191 | 0 | NM_024575 | FLJ23467 | 0 |
| NM_024788 | FLJ21062 | 0 | NM_024581 | C6ORF60 | 0 |
| NM_024587 | FLJ22353 | 0 | NM_024299 | C20ORF149 | 0 |
| NM_024939 | FLJ21918 | 0 | NM_024946 | NIP30 | 0 |
| XM_371114 | FHOD3 | 0 | NM_024969 | TAIP-2 | 0 |
| NM_024993 | LRRTM4 | 0 | NM_025158 | RUFY1 | 0 |
| NM_025165 | ELL3 | 0 | NM_025188 | TRIM45 | 0 |
| NM_173050 | SCUBE1 | 0 | NM_025228 | T3JAM | 0 |
| NM_025115 | FLJ23263 | 0 | NM_030797 | DKFZP566A1524 | 0 |
| NM_031294 | DKFZP586M1120 | 0 | NM_181519 | SYT15 | 0 |
| NM_031913 | CHR3SYT | 0 | NM_032177 | PHAX | 0 |
| NM_031444 | C22ORF13 | 0 | NM_030937 | CCNL2 | 0 |

| | | | | | |
|---|---|---|---|---|---|
| NM_031956 | NYD-SP14 | 0 | NM_032027 | BBP | 0 |
| NM_030981 | RAB1B | 0 | NM_032581 | DRCTNNB1A | 0 |
| NM_032482 | DOT1L | 0 | NM_032439 | PHYHIPL | 0 |
| NM_032320 | GMRP-1 | 0 | NM_032609 | COX4I2 | 0 |
| NM_032644 | MGC2452 | 0 | NM_032826 | SLC35B4 | 0 |
| NM_032853 | MUM1 | 0 | NM_032747 | USMG5 | 0 |
| NM_032889 | MGC11308 | 0 | NM_138568 | XTP7 | 0 |
| XM_031561 | FLJ14502 | 0 | NM_032936 | C7ORF35 | 0 |
| NM_033449 | FCHSD1 | 0 | NM_133373 | PLCD3 | 0 |
| NM_079834 | SCAMP4 | 0 | NM_138422 | LOC113179 | 0 |
| NM_058190 | C21ORF70 | 0 | NM_198920 | C6ORF157 | 0 |
| XM_371152 | ZNF486 | 0 | NM_138360 | LOC90668 | 0 |
| NM_052879 | LOC113251 | 0 | NM_033277 | LACRT | 0 |
| NM_033397 | KIAA1754 | 0 | NM_178121 | SBP1 | 0 |
| XM_372193 | KIAA1751 | 0 | NM_033061 | KRTAP4-7 | 0 |
| NM_052934 | SLC26A9 | 0 | NM_152287 | ZFP276 | 0 |
| NM_144579 | SFXN5 | 0 | NM_144597 | MGC29937 | 0 |
| NM_173480 | LOC126295 | 0 | NM_181643 | LOC128344 | 0 |
| NM_153690 | FLJ90022 | 0 | NM_152399 | FLJ30834 | 0 |
| NM_152413 | MGC33309 | 0 | NM_145263 | LOC132671 | 0 |
| NM_152437 | DKFZP761B128 | 0 | NM_152384 | BBS5 | 0 |
| NM_153706 | MGC33648 | 0 | NM_175874 | MGC47869 | 0 |
| NM_182508 | FLJ40919 | 0 | NM_152462 | TMEM21A | 0 |
| XM_096733 | C14ORF72 | 0 | NM_152444 | ZADH1 | 0 |
| NM_173078 | SLITRK4 | 0 | NM_178491 | R3HDML | 0 |
| XM_085831 | LOC147645 | 0 | NM_145053 | MGC20470 | 0 |
| NM_002475 | MLC1SA | 0 | NM_152459 | MGC45438 | 0 |
| NM_144692 | LOC148137 | 0 | NM_152908 | FLJ31196 | 0 |
| XM_351649 | OACT1 | 0 | NM_152510 | MGC26710 | 0 |
| NM_152492 | FLJ32825 | 0 | NM_152570 | FLJ31810 | 0 |
| NM_175882 | IMP5 | 0 | NM_138280 | CLYBL | 0 |
| NM_152601 | FLJ38281 | 0 | NM_152636 | FLJ33979 | 0 |
| NM_173531 | ZNF100 | 0 | NM_181701 | QSCN6L1 | 0 |
| NM_152632 | CXORF22 | 0 | NM_207338 | KLPH | 0 |
| XM_378240 | LOC170425 | 0 | NM_130759 | IMAP1 | 0 |
| NM_152660 | MGC34648 | 0 | NM_152681 | FLJ38482 | 0 |
| NM_152734 | C6ORF89 | 0 | NM_145303 | LOC202459 | 0 |
| XM_371401 | C20ORF106 | 0 | NM_174923 | MGC31967 | 0 |
| XM_371878 | FLJ14712 | 0 | XM_166508 | TWISTNB | 0 |
| XM_291062 | KIAA2018 | 0 | XM_378230 | C10ORF40 | 0 |
| NM_145065 | MGC35521 | 0 | NM_173607 | C14ORF24 | 0 |
| NM_173659 | MGC29784 | 0 | XM_209140 | LOC284323 | 0 |
| NM_198477 | UNQ473 | 0 | NM_174945 | ZNF575 | 0 |
| XM_379258 | LOC285547 | 0 | XM_379064 | LOC284933 | 0 |
| NM_206880 | OR2V2 | 0 | NM_173676 | PNPLA1 | 0 |
| XM_379324 | LOC340113 | 0 | NM_198495 | CTAGE4 | 0 |

| | | | | | |
|---|---|---|---|---|---|
| NM_198317 | LOC339451 | 0 | XM_378941 | LOC339535 | 0 |
| XM_379665 | LOC286333 | 0 | XM_379728 | LOC286411 | 0 |
| XM_293090 | SH3MD4 | 0 | XM_371248 | LOC374973 | 0 |
| XM_294357 | LOC346653 | 0 | NM_207379 | FLJ42486 | 0 |
| NM_207382 | FLJ43276 | 0 | NM_198567 | FLJ44216 | 0 |
| NM_182614 | MGC20579 | 0 | NM_199337 | LOC374395 | 0 |
| NM_198529 | FLJ46247 | 0 | XM_379520 | FLJ43663 | 0 |
| NM_182905 | DKFZP434K1323 | 0 | NM_199358 | FOXD4L3 | 0 |
| NM_198944 | OR7C1 | 0 | NM_018964 | SLC37A1 | 0 |
| XM_062890 | LOC121981 | 0 | XM_496404 | LOC440683 | 0 |
| NM_203453 | LOC403313 | 0 | XM_292225 | LOC338879 | 0 |
| XM_086937 | LOC150519 | 0 | XM_209604 | LOC285423 | 0 |
| XM_292384 | LOC342132 | 0 | XM_060956 | LOC128371 | 0 |
| XM_211447 | LOC284409 | 0 | XM_117266 | LOC200726 | 0 |
| XM_065722 | LOC130429 | 0 | XM_297205 | LOC342900 | 0 |
| XM_297816 | LOC343637 | 0 | XM_114415 | LOC201989 | 0 |
| XM_211408 | LOC284260 | 0 | XM_292813 | LOC342972 | 0 |
| XM_210856 | LOC283029 | 0 | XM_294723 | LOC338825 | 0 |
| XM_373896 | LOC388755 | 0 | XM_378901 | LOC400786 | 0 |
| XM_371511 | LOC388972 | 0 | XM_371306 | LOC388689 | 0 |
| XM_376099 | LOC400985 | 0 | XM_373878 | LOC388719 | 0 |
| XM_371684 | LOC389192 | 0 | XM_373893 | LOC388751 | 0 |
| XM_377577 | LOC401944 | 0 | XM_371665 | LOC389151 | 0 |
| XM_373076 | LOC391764 | 0 | XM_373090 | LOC391797 | 0 |
| XM_371726 | LOC389267 | 0 | XM_379506 | LOC401384 | 0 |
| XM_291247 | LOC389530 | 0 | XM_379512 | LOC401394 | 0 |
| XM_374249 | LOC389628 | 0 | XM_379603 | LOC401476 | 0 |
| XM_379786 | LOC402461 | 0 | XM_374272 | LOC389666 | 0 |
| XM_373253 | LOC392222 | 0 | XM_374705 | LOC393078 | 0 |
| XM_379847 | LOC402520 | 0 | XM_373471 | LOC387706 | 0 |
| XM_376846 | LOC401511 | 0 | XM_374283 | LOC389689 | 0 |
| XM_378398 | LOC400092 | 0 | XM_378416 | LOC400134 | 0 |
| XM_373566 | LOC387931 | 0 | XM_372490 | LOC390387 | 0 |
| XM_373660 | LOC388206 | 0 | XM_372723 | LOC390916 | 0 |
| XM_371197 | LOC388565 | 0 | XM_371200 | LOC388567 | 0 |
| XM_378700 | LOC400618 | 0 | XM_372702 | LOC390874 | 0 |
| XM_371142 | LOC388509 | 0 | XM_373921 | LOC388806 | 0 |
| XM_371024 | LOC388345 | 0 | XM_372869 | LOC391253 | 0 |
| XM_377002 | LOC401590 | 0 | XM_371395 | LOC388794 | 0 |
| NM_001001681 | FLJ45300 | 0 | XM_372262 | LOC389901 | 0 |
| XM_373340 | LOC392433 | 0 | NM_001001712 | LCN10 | 0 |
| NM_001001920 | OR4C15 | 0 | NM_001002035 | DEFB108 | 0 |
| XM_373366 | LOC392528 | 0 | XM_379046 | LOC400923 | 0 |
| XM_373957 | LOC388897 | 0 | NM_001005276 | OR2AE1 | 0 |
| XM_372891 | LOC391303 | 0 | XM_351948 | LOC389916 | 0 |
| NM_001003819 | TRIM6-TRIM34 | 0 | XM_379885 | LOC402562 | 0 |

| | | | | | |
|---|---|---|---|---|---|
| NM_213609 | FAM19A1 | 0 | XM_380134 | LOC402574 | 0 |
| NM_001006121 | RBMY1B | 0 | NM_001006603 | MG29 | 0 |
| NM_005198 | CHKB | 0.01 | NM_004302 | ACVR1B | 0.01 |
| NM_032237 | FLJ23356 | 0.01 | XM_291277 | DKFZP761P0423 | 0.01 |
| NM_001626 | AKT2 | 0.01 | NM_001946 | DUSP6 | 0.01 |
| NM_001262 | CDKN2C | 0.01 | NM_173575 | STK32C | 0.01 |
| NM_032471 | PKIB | 0.01 | NM_002746 | MAPK3 | 0.01 |
| NM_006252 | PRKAA2 | 0.01 | XM_046685 | KIAA1399 | 0.01 |
| NM_015254 | KIF13B | 0.01 | NM_003831 | RIOK3 | 0.01 |
| NM_005406 | ROCK1 | 0.01 | NM_003161 | RPS6KB1 | 0.01 |
| NM_006622 | PLK2 | 0.01 | NM_001742 | CALCR | 0.01 |
| NM_005624 | CCL25 | 0.01 | NM_015000 | STK38L | 0.01 |
| NM_002923 | RGS2 | 0.01 | NM_031936 | GPR61 | 0.01 |
| NM_004432 | ELAVL2 | 0.01 | NM_002898 | RBMS2 | 0.01 |
| NM_004240 | TRIP10 | 0.01 | NM_152235 | SFRS8 | 0.01 |
| NM_003428 | ZNF84 | 0.01 | NM_003189 | TAL1 | 0.01 |
| NM_004098 | EMX2 | 0.01 | NM_004514 | FOXK2 | 0.01 |
| NM_006312 | NCOR2 | 0.01 | NM_012259 | HEY2 | 0.01 |
| NM_014571 | HEYL | 0.01 | NM_005870 | SAP18 | 0.01 |
| NM_006735 | HOXA2 | 0.01 | NM_001226 | CASP6 | 0.01 |
| NM_006974 | ZNF33A | 0.01 | NM_000687 | AHCY | 0.01 |
| NM_003754 | EIF3S5 | 0.01 | NM_004044 | ATIC | 0.01 |
| NM_016245 | DHRS8 | 0.01 | NM_004208 | PDCD8 | 0.01 |
| NM_152667 | HDHD4 | 0.01 | NM_020202 | NIT2 | 0.01 |
| NM_005659 | UFD1L | 0.01 | NM_021794 | ADAM30 | 0.01 |
| NM_003340 | UBE2D3 | 0.01 | NM_022553 | VPS52 | 0.01 |
| NM_004163 | RAB27B | 0.01 | NM_002542 | OGG1 | 0.01 |
| NM_002878 | RAD51L3 | 0.01 | NM_000300 | PLA2G2A | 0.01 |
| NM_006420 | ARFGEF2 | 0.01 | NM_004583 | RAB5C | 0.01 |
| NM_012189 | CABYR | 0.01 | NM_000801 | FKBP1A | 0.01 |
| NM_000265 | NCF1 | 0.01 | NM_004656 | BAP1 | 0.01 |
| NM_020165 | RAD18 | 0.01 | NM_003922 | HERC1 | 0.01 |
| NM_017641 | KIF21A | 0.01 | NM_014225 | PPP2R1A | 0.01 |
| NM_002829 | PTPN3 | 0.01 | NM_021090 | MTMR3 | 0.01 |
| NM_002186 | IL9R | 0.01 | NM_005538 | INHBC | 0.01 |
| NM_003581 | NCK2 | 0.01 | NM_002131 | HMGA1 | 0.01 |
| NM_003006 | SELPLG | 0.01 | NM_005117 | FGF19 | 0.01 |
| NM_005126 | NR1D2 | 0.01 | NM_001723 | BPAG1 | 0.01 |
| NM_016824 | ADD3 | 0.01 | NM_006511 | RSC1A1 | 0.01 |
| NM_003049 | SLC10A1 | 0.01 | NM_015120 | ALMS1 | 0.01 |
| NM_020399 | GOPC | 0.01 | NM_005477 | HCN4 | 0.01 |
| NM_004913 | C16ORF7 | 0.01 | NM_002251 | KCNS1 | 0.01 |
| NM_001662 | ARF5 | 0.01 | NM_005891 | ACAT2 | 0.01 |
| NM_002202 | ISL1 | 0.01 | XM_496142 | ITGAD | 0.01 |
| NM_001976 | ENO3 | 0.01 | NM_002301 | LDHC | 0.01 |
| NM_014459 | PCDH17 | 0.01 | NM_001151 | SLC25A4 | 0.01 |

| | | | | | |
|---|---|---|---|---|---|
| NM_005609 | PYGM | 0.01 | NM_005833 | RAB9P40 | 0.01 |
| NM_001690 | ATP6V1A | 0.01 | NM_001694 | ATP6V0C | 0.01 |
| NM_015270 | ADCY6 | 0.01 | NM_001122 | ADFP | 0.01 |
| NM_005140 | CNGA2 | 0.01 | NM_001860 | SLC31A2 | 0.01 |
| NM_000727 | CACNG1 | 0.01 | NM_001884 | HAPLN1 | 0.01 |
| NM_170662 | CBLB | 0.01 | NM_001679 | ATP1B3 | 0.01 |
| NM_001247 | ENTPD6 | 0.01 | NM_004391 | CYP8B1 | 0.01 |
| NM_000820 | GAS6 | 0.01 | NM_019112 | ABCA7 | 0.01 |
| NM_004146 | NDUFB7 | 0.01 | NM_016228 | AADAT | 0.01 |
| NM_005071 | SLC1A6 | 0.01 | NM_002762 | PRM2 | 0.01 |
| NM_013360 | ZNF222 | 0.01 | NM_004179 | TPH1 | 0.01 |
| NM_004622 | TSN | 0.01 | NM_003535 | HIST1H3J | 0.01 |
| NM_003586 | DOC2A | 0.01 | NM_003910 | G10 | 0.01 |
| NM_003762 | VAMP4 | 0.01 | NM_004848 | C1ORF38 | 0.01 |
| XM_379800 | KIAA0644 | 0.01 | NM_014748 | SNX17 | 0.01 |
| NM_006327 | TIMM23 | 0.01 | NM_014865 | CNAP1 | 0.01 |
| XM_376060 | ARHGAP25 | 0.01 | NM_005815 | ZNF443 | 0.01 |
| NM_015029 | POP1 | 0.01 | NM_014142 | NUDT5 | 0.01 |
| XM_045792 | GCN1L1 | 0.01 | NM_006459 | C10ORF69 | 0.01 |
| NM_007010 | DDX52 | 0.01 | NM_006786 | UTS2 | 0.01 |
| NM_012306 | FAIM2 | 0.01 | NM_014909 | KIAA1036 | 0.01 |
| NM_015022 | PDZK3 | 0.01 | XM_093839 | KIAA0826 | 0.01 |
| NM_015151 | C21ORF106 | 0.01 | NM_015303 | KIAA0804 | 0.01 |
| NM_014310 | RASD2 | 0.01 | NM_015327 | EST1B | 0.01 |
| NM_012279 | ZNF346 | 0.01 | NM_015497 | DKFZP564G2022 | 0.01 |
| NM_012244 | SLC7A8 | 0.01 | NM_015421 | DKFZP564K2062 | 0.01 |
| NM_012295 | CABIN1 | 0.01 | XM_291986 | OR10A3 | 0.01 |
| NM_013245 | VPS4A | 0.01 | NM_014466 | TEKT2 | 0.01 |
| NM_015658 | DKFZP564C186 | 0.01 | NM_014169 | C14ORF123 | 0.01 |
| NM_014484 | MOCS3 | 0.01 | NM_013238 | DNAJD1 | 0.01 |
| NM_014425 | INVS | 0.01 | NM_030622 | CYP2S1 | 0.01 |
| NM_015965 | GRIM19 | 0.01 | NM_016950 | SPOCK3 | 0.01 |
| NM_015987 | HEBP1 | 0.01 | NM_016115 | ASB3 | 0.01 |
| NM_016063 | C6ORF74 | 0.01 | NM_016527 | HAO2 | 0.01 |
| NM_018986 | SH3TC1 | 0.01 | NM_017821 | RHBDL2 | 0.01 |
| NM_017822 | FLJ20436 | 0.01 | NM_017952 | FLJ20758 | 0.01 |
| NM_016506 | KBTBD4 | 0.01 | NM_018392 | FLJ11331 | 0.01 |
| NM_018246 | FLJ10853 | 0.01 | NM_020200 | PRTFDC1 | 0.01 |
| NM_020645 | NRIP3 | 0.01 | XM_050325 | KIAA1126 | 0.01 |
| NM_020795 | NLGN2 | 0.01 | NM_020866 | KLHL1 | 0.01 |
| XM_372133 | KIAA1529 | 0.01 | NM_021210 | TRAPPC1 | 0.01 |
| NM_022367 | SEMA4A | 0.01 | NM_023007 | FLJ12517 | 0.01 |
| NM_022756 | FLJ11730 | 0.01 | NM_022460 | FLJ14249 | 0.01 |
| NM_022118 | C13ORF10 | 0.01 | NM_022838 | FLJ12969 | 0.01 |
| NM_024006 | VKORC1 | 0.01 | NM_024295 | MGC3067 | 0.01 |
| NM_024677 | FLJ14001 | 0.01 | NM_024792 | CT120 | 0.01 |

| | | |
|---|---|---|
| NM_024796 | FLJ22639 | 0.01 |
| NM_024947 | PHC3 | 0.01 |
| NM_030820 | COL21A1 | 0.01 |
| NM_031430 | RILP | 0.01 |
| NM_031454 | SELO | 0.01 |
| NM_032034 | SLC4A11 | 0.01 |
| NM_032286 | MGC5309 | 0.01 |
| NM_032324 | MGC13186 | 0.01 |
| NM_032344 | MGC13045 | 0.01 |
| NM_031460 | KCNK17 | 0.01 |
| XM_029323 | LOC90133 | 0.01 |
| NM_052911 | EFO1 | 0.01 |
| NM_053284 | WFIKKN | 0.01 |
| NM_144595 | FLJ30046 | 0.01 |
| NM_145171 | GPB5 | 0.01 |
| NM_198446 | FLJ45459 | 0.01 |
| NM_080740 | SUHW1 | 0.01 |
| NM_138799 | LOC129642 | 0.01 |
| NM_203299 | MGC41945 | 0.01 |
| NM_145056 | MGC15476 | 0.01 |
| NM_144695 | FLJ32421 | 0.01 |
| XM_379114 | LOC150577 | 0.01 |
| NM_152525 | FLJ25351 | 0.01 |
| NM_145057 | CDC42EP5 | 0.01 |
| XM_113706 | DNAH10 | 0.01 |
| NM_175060 | C14ORF27 | 0.01 |
| NM_152727 | CPNE2 | 0.01 |
| NM_145020 | FLJ32743 | 0.01 |
| NM_152913 | DKFZP761L1417 | 0.01 |
| NM_173803 | FLJ39599 | 0.01 |
| NM_182701 | GPX6 | 0.01 |
| NM_181717 | HCG27 | 0.01 |
| NM_148896 | NPB | 0.01 |
| XM_375935 | LOC284825 | 0.01 |
| XM_379530 | LOC285972 | 0.01 |
| NM_178314 | FLJ39378 | 0.01 |
| NM_198549 | FLJ35093 | 0.01 |
| NM_182617 | LOC348158 | 0.01 |
| XM_372143 | C9ORF119 | 0.01 |
| NM_207479 | FLJ41046 | 0.01 |
| NM_207487 | FLJ46211 | 0.01 |
| NM_001001551 | C9ORF103 | 0.01 |
| NM_203448 | MGC21881 | 0.01 |
| NM_207647 | MGC45564 | 0.01 |
| XM_096864 | LOC145780 | 0.01 |
| XM_293680 | LOC345051 | 0.01 |
| NM_025126 | RNF34 | 0.01 |
| XM_376325 | FLJ13105 | 0.01 |
| NM_030925 | CAB39L | 0.01 |
| XM_371164 | NYD-SP11 | 0.01 |
| NM_030965 | SIAT7E | 0.01 |
| NM_030978 | ARPC5L | 0.01 |
| NM_032296 | DKFZP761A132 | 0.01 |
| NM_032824 | FLJ14681 | 0.01 |
| NM_032679 | ZNF577 | 0.01 |
| XM_371461 | KIAA1671 | 0.01 |
| XM_027668 | SLC25A25 | 0.01 |
| NM_031910 | C1QTNF6 | 0.01 |
| XM_062871 | FLJ40176 | 0.01 |
| NM_178860 | SEZ6 | 0.01 |
| NM_032796 | SYAP1 | 0.01 |
| XM_059051 | LOC126520 | 0.01 |
| NM_174898 | LOC129530 | 0.01 |
| NM_198449 | MGC71745 | 0.01 |
| NM_130848 | DCNP1 | 0.01 |
| NM_182520 | FLJ36561 | 0.01 |
| XM_166346 | C6ORF129 | 0.01 |
| NM_152995 | HOZFP | 0.01 |
| NM_145652 | WFDC5 | 0.01 |
| NM_152617 | FLJ35794 | 0.01 |
| NM_201400 | SB153 | 0.01 |
| NM_152677 | ZNF494 | 0.01 |
| NM_181336 | LEMD2 | 0.01 |
| NM_152751 | C10ORF30 | 0.01 |
| NM_198473 | FLJ46111 | 0.01 |
| NM_152770 | MGC35043 | 0.01 |
| NM_153270 | FLJ34960 | 0.01 |
| XM_371335 | LOC388726 | 0.01 |
| NM_198485 | FLJ41238 | 0.01 |
| NM_152616 | TRIM42 | 0.01 |
| NM_207403 | FLJ42986 | 0.01 |
| NM_198283 | EGFL11 | 0.01 |
| NM_207377 | UNQ9438 | 0.01 |
| NM_175078 | KRT1B | 0.01 |
| XM_373798 | LOC374890 | 0.01 |
| NM_205856 | LOC389852 | 0.01 |
| NM_207491 | MGC48628 | 0.01 |
| NM_203449 | FLJ20444 | 0.01 |
| NM_203307 | MGC35402 | 0.01 |
| XM_291543 | LOC343381 | 0.01 |
| XM_295058 | LOC339760 | 0.01 |
| XM_093644 | LOC152138 | 0.01 |

| | | |
|---|---|---|
| XM_059473 | LOC130839 | 0.01 |
| XM_084868 | LOC144448 | 0.01 |
| XM_087800 | LOC153918 | 0.01 |
| XM_210186 | LOC283162 | 0.01 |
| XM_378843 | LOC400743 | 0.01 |
| XM_376200 | LOC401047 | 0.01 |
| XM_376241 | LOC401070 | 0.01 |
| XM_377824 | LOC402157 | 0.01 |
| XM_374047 | LOC389143 | 0.01 |
| XM_376591 | LOC401322 | 0.01 |
| XM_376353 | LOC401160 | 0.01 |
| XM_379909 | LOC402580 | 0.01 |
| XM_372169 | LOC389822 | 0.01 |
| XM_374307 | LOC389801 | 0.01 |
| XM_373445 | LOC387631 | 0.01 |
| XM_378272 | LOC399866 | 0.01 |
| XM_373637 | LOC388141 | 0.01 |
| XM_370843 | LOC388092 | 0.01 |
| XM_375387 | LOC400566 | 0.01 |
| XM_375292 | LOC400492 | 0.01 |
| XM_372609 | LOC390669 | 0.01 |
| XM_378649 | LOC400573 | 0.01 |
| XM_372864 | LOC391241 | 0.01 |
| NM_001004339 | LOC440590 | 0.01 |
| XM_373398 | LOC392586 | 0.01 |
| NM_002512 | NME2 | 0.02 |
| NM_006257 | PRKCQ | 0.02 |
| NM_032028 | STK22D | 0.02 |
| NM_033050 | GPR91 | 0.02 |
| NM_018939 | PCDHB6 | 0.02 |
| NM_021145 | DMTF1 | 0.02 |
| NM_002448 | MSX1 | 0.02 |
| NM_003120 | SPI1 | 0.02 |
| NM_004899 | BRE | 0.02 |
| NM_005994 | TBX2 | 0.02 |
| NM_002012 | FHIT | 0.02 |
| NM_003286 | TOP1 | 0.02 |
| NM_000772 | CYP2C18 | 0.02 |
| NM_002693 | POLG | 0.02 |
| NM_001513 | GSTZ1 | 0.02 |
| NM_015313 | ARHGEF12 | 0.02 |
| NM_001770 | CD19 | 0.02 |
| NM_014387 | LAT | 0.02 |
| NM_003394 | WNT10B | 0.02 |
| NM_018110 | DOK4 | 0.02 |
| NM_012064 | MIP | 0.02 |
| XM_293026 | LOC344382 | 0.01 |
| XM_208356 | LOC285849 | 0.01 |
| XM_291334 | LOC340543 | 0.01 |
| XM_098512 | LOC154323 | 0.01 |
| XM_371497 | LOC388957 | 0.01 |
| XM_371534 | LOC389000 | 0.01 |
| XM_372984 | LOC391509 | 0.01 |
| XM_372916 | LOC391352 | 0.01 |
| XM_373037 | LOC391711 | 0.01 |
| XM_373028 | LOC391661 | 0.01 |
| XM_377879 | LOC402202 | 0.01 |
| XM_372112 | LOC389753 | 0.01 |
| XM_374801 | LOC399761 | 0.01 |
| XM_370759 | LOC387979 | 0.01 |
| XM_373599 | LOC388001 | 0.01 |
| XM_372504 | LOC390436 | 0.01 |
| XM_378684 | LOC400604 | 0.01 |
| XM_375344 | LOC400526 | 0.01 |
| XM_375632 | LOC400708 | 0.01 |
| XM_372727 | LOC390928 | 0.01 |
| XM_373770 | LOC388454 | 0.01 |
| XM_371405 | LOC388809 | 0.01 |
| XM_372233 | LOC389873 | 0.01 |
| XM_372889 | LOC391298 | 0.01 |
| NM_001799 | CDK7 | 0.02 |
| NM_032435 | KIAA1804 | 0.02 |
| NM_002758 | MAP2K6 | 0.02 |
| NM_006282 | STK4 | 0.02 |
| NM_005305 | GPR42 | 0.02 |
| NM_003882 | WISP1 | 0.02 |
| NM_007348 | ATF6 | 0.02 |
| NM_007237 | SP140 | 0.02 |
| NM_000016 | ACADM | 0.02 |
| NM_006284 | TAF10 | 0.02 |
| NM_005589 | ALDH6A1 | 0.02 |
| NM_005265 | GGT1 | 0.02 |
| NM_001216 | CA9 | 0.02 |
| NM_006623 | PHGDH | 0.02 |
| NM_004161 | RAB1A | 0.02 |
| NM_002675 | PML | 0.02 |
| NM_012175 | FBXO3 | 0.02 |
| NM_021230 | MLL3 | 0.02 |
| NM_000165 | GJA1 | 0.02 |
| NM_005037 | PPARG | 0.02 |
| NM_000807 | GABRA2 | 0.02 |
| NM_001103 | ACTN2 | 0.02 |

| | | | | | |
|---|---|---|---|---|---|
| NM_000495 | COL4A5 | 0.02 | NM_133467 | CITED4 | 0.02 |
| NM_000031 | ALAD | 0.02 | NM_001443 | FABP1 | 0.02 |
| NM_000356 | TCOF1 | 0.02 | NM_017882 | CLN6 | 0.02 |
| NM_018354 | C20ORF46 | 0.02 | NM_005853 | IRX5 | 0.02 |
| NM_003541 | HIST1H4K | 0.02 | NM_020435 | GJA12 | 0.02 |
| NM_175747 | OLIG3 | 0.02 | NM_138702 | MAGEC3 | 0.02 |
| NM_003981 | PRC1 | 0.02 | NM_000965 | RARB | 0.02 |
| NM_003073 | SMARCB1 | 0.02 | NM_005621 | S100A12 | 0.02 |
| NM_001785 | CDA | 0.02 | NM_001544 | ICAM4 | 0.02 |
| NM_005333 | HCCS | 0.02 | NM_033045 | KRTHB4 | 0.02 |
| NM_004537 | NAP1L1 | 0.02 | NM_007002 | ADRM1 | 0.02 |
| NM_080284 | ABCA6 | 0.02 | NM_003034 | SIAT8A | 0.02 |
| NM_032021 | AD031 | 0.02 | XM_072402 | ACY1L2 | 0.02 |
| NM_003433 | ZNF132 | 0.02 | NM_021088 | ZNF2 | 0.02 |
| NM_003180 | SYT5 | 0.02 | NM_006764 | IFRD2 | 0.02 |
| NM_004863 | SPTLC2 | 0.02 | NM_003669 | INE1 | 0.02 |
| NM_014807 | TMEM24 | 0.02 | NM_012445 | SPON2 | 0.02 |
| NM_016172 | UBADC1 | 0.02 | NM_013441 | DSCR1L2 | 0.02 |
| NM_006386 | DDX17 | 0.02 | NM_006821 | ZAP128 | 0.02 |
| NM_006853 | KLK11 | 0.02 | XM_372267 | ZNF275 | 0.02 |
| NM_012190 | FTHFD | 0.02 | NM_006866 | LILRA2 | 0.02 |
| NM_006665 | HPSE | 0.02 | NM_015281 | KIAA1043 | 0.02 |
| NM_012217 | TPSD1 | 0.02 | NM_007352 | ELA3B | 0.02 |
| NM_013281 | FLRT3 | 0.02 | NM_012124 | CHORDC1 | 0.02 |
| NM_014395 | DAPP1 | 0.02 | NM_014356 | C6ORF123 | 0.02 |
| NM_015960 | CUTC | 0.02 | NM_015972 | POLR1D | 0.02 |
| NM_153694 | SYCP3 | 0.02 | NM_013312 | HOOK2 | 0.02 |
| NM_016468 | C14ORF112 | 0.02 | NM_013337 | TIMM22 | 0.02 |
| NM_016026 | RDH11 | 0.02 | NM_016035 | COQ4 | 0.02 |
| NM_016093 | RPL26L1 | 0.02 | NM_013393 | FTSJ2 | 0.02 |
| NM_016603 | C5ORF5 | 0.02 | NM_016183 | C1ORF33 | 0.02 |
| NM_015976 | SNX7 | 0.02 | NM_017553 | KIAA1259 | 0.02 |
| NM_016438 | CLST11240 | 0.02 | NM_017521 | FEV | 0.02 |
| NM_017789 | SEMA4C | 0.02 | NM_018076 | ARMC4 | 0.02 |
| NM_017836 | SLC41A3 | 0.02 | NM_017850 | FLJ20508 | 0.02 |
| NM_017709 | FLJ20202 | 0.02 | NM_017807 | OSGEP | 0.02 |
| NM_024718 | C9ORF86 | 0.02 | NM_018466 | MDS031 | 0.02 |
| NM_020228 | PRDM10 | 0.02 | NM_020787 | ZNF624 | 0.02 |
| NM_020925 | KIAA1573 | 0.02 | XM_372716 | WIZ | 0.02 |
| NM_021633 | KLHL12 | 0.02 | NM_021183 | RAP2C | 0.02 |
| NM_017542 | POGK | 0.02 | NM_020895 | KIAA1533 | 0.02 |
| NM_033055 | HIAT1 | 0.02 | NM_021941 | C21ORF97 | 0.02 |
| NM_023938 | SARG | 0.02 | XM_378187 | MGC4473 | 0.02 |
| NM_025097 | FLJ21106 | 0.02 | NM_025113 | C13ORF18 | 0.02 |
| NM_030814 | C9ORF45 | 0.02 | NM_031434 | C7ORF21 | 0.02 |
| NM_032315 | MGC4399 | 0.02 | NM_152259 | MGC45866 | 0.02 |

| | | | | | |
|---|---|---|---|---|---|
| NM_033064 | ATCAY | 0.02 | NM_130468 | D4ST1 | 0.02 |
| NM_052999 | CKLFSF1 | 0.02 | NM_153754 | C21ORF88 | 0.02 |
| NM_080920 | GGTLA4 | 0.02 | XM_373760 | LOC92659 | 0.02 |
| NM_152379 | DKFZP547B1713 | 0.02 | NM_133264 | WIRE | 0.02 |
| NM_173506 | MGC42718 | 0.02 | NM_144676 | MGC23911 | 0.02 |
| NM_153026 | PRICKLE1 | 0.02 | XM_086188 | LOC148418 | 0.02 |
| NM_145277 | HFE2 | 0.02 | NM_182524 | ZNF595 | 0.02 |
| NM_175075 | INM01 | 0.02 | NM_153608 | MGC17986 | 0.02 |
| NM_153356 | MGC34741 | 0.02 | NM_182547 | HNLF | 0.02 |
| NM_175885 | MGC33846 | 0.02 | XM_171068 | LOC253017 | 0.02 |
| NM_152789 | MGC40405 | 0.02 | NM_148894 | FLJ33215 | 0.02 |
| NM_172131 | WFDC10B | 0.02 | NM_173602 | FLJ34278 | 0.02 |
| NM_173622 | FLJ36674 | 0.02 | NM_178520 | FLJ38792 | 0.02 |
| XM_370932 | FLJ39639 | 0.02 | NM_173650 | DNAJC5G | 0.02 |
| NM_198491 | FLJ44299 | 0.02 | NM_199051 | DBCCR1L | 0.02 |
| XM_376781 | SLC10A5 | 0.02 | NM_182612 | FLJ34283 | 0.02 |
| NM_198586 | NHLRC1 | 0.02 | NM_178837 | LOC352909 | 0.02 |
| NM_198542 | MGC4728 | 0.02 | XM_114090 | LOC200019 | 0.02 |
| XM_292832 | LOC342991 | 0.02 | XM_087208 | LOC151451 | 0.02 |
| XM_292810 | LOC342970 | 0.02 | XM_072554 | LOC138729 | 0.02 |
| XM_086876 | LOC150207 | 0.02 | XM_371343 | LOC388739 | 0.02 |
| XM_375687 | LOC400734 | 0.02 | XM_371261 | LOC388642 | 0.02 |
| XM_294450 | LOC346887 | 0.02 | XM_208043 | LOC283116 | 0.02 |
| XM_208060 | LOC283257 | 0.02 | XM_373989 | LOC388959 | 0.02 |
| XM_371506 | LOC388969 | 0.02 | XM_371623 | LOC389102 | 0.02 |
| XM_379320 | LOC401177 | 0.02 | XM_374155 | LOC389358 | 0.02 |
| XM_374159 | LOC389362 | 0.02 | XM_377875 | LOC402199 | 0.02 |
| XM_379584 | LOC401463 | 0.02 | XM_373308 | LOC392376 | 0.02 |
| XM_373602 | LOC388006 | 0.02 | XM_378363 | LOC400047 | 0.02 |
| XM_374355 | LOC390565 | 0.02 | XM_378621 | LOC400551 | 0.02 |
| XM_378582 | LOC400523 | 0.02 | XM_378650 | LOC400575 | 0.02 |
| NM_001004714 | OR4K13 | 0.02 | NM_001005205 | OR8J1 | 0.02 |
| NM_001005279 | OR6K2 | 0.02 | NM_001003665 | DKFZP761P211 | 0.02 |
| NM_001003750 | OR8I2 | 0.02 | NM_001565 | CXCL10 | 0.03 |
| NM_144685 | HIPK4 | 0.03 | NM_016586 | MBIP | 0.03 |
| NM_000245 | MET | 0.03 | NM_000710 | BDKRB1 | 0.03 |
| NM_005307 | GRK4 | 0.03 | NM_012344 | NTSR2 | 0.03 |
| NM_003702 | RGS20 | 0.03 | NM_005166 | APLP1 | 0.03 |
| NM_000174 | GP9 | 0.03 | NM_002623 | PFDN4 | 0.03 |
| NM_033288 | ZNF160 | 0.03 | NM_014323 | ZNF278 | 0.03 |
| NM_001452 | FOXF2 | 0.03 | NM_005962 | MXI1 | 0.03 |
| NM_002228 | JUN | 0.03 | NM_005360 | MAF | 0.03 |
| NM_021615 | CHST6 | 0.03 | NM_006025 | P11 | 0.03 |
| NM_002299 | LCT | 0.03 | NM_001984 | ESD | 0.03 |
| NM_003837 | FBP2 | 0.03 | NM_005865 | PRSS16 | 0.03 |
| NM_019076 | UGT1A8 | 0.03 | NM_003216 | TEF | 0.03 |

| | | | | | |
|---|---|---|---|---|---|
| NM_021822 | APOBEC3G | 0.03 | NM_000499 | CYP1A1 | 0.03 |
| NM_002422 | MMP3 | 0.03 | NM_004564 | PET112L | 0.03 |
| NM_024117 | MAPKAP1 | 0.03 | NM_005369 | MCF2 | 0.03 |
| NM_004210 | NEURL | 0.03 | NM_005055 | RAPSN | 0.03 |
| NM_006287 | TFPI | 0.03 | NM_002938 | RNF4 | 0.03 |
| NM_005852 | CHD3 | 0.03 | NM_000795 | DRD2 | 0.03 |
| AF174606 | SHFM3P1 | 0.03 | NM_003620 | PPM1D | 0.03 |
| NM_006080 | SEMA3A | 0.03 | NM_006748 | SLA | 0.03 |
| NM_003326 | TNFSF4 | 0.03 | NM_003037 | SLAMF1 | 0.03 |
| NM_001996 | FBLN1 | 0.03 | NM_001651 | AQP5 | 0.03 |
| NM_001149 | ANK3 | 0.03 | NM_012316 | KPNA6 | 0.03 |
| NM_016277 | RAB23 | 0.03 | NM_021734 | SLC25A19 | 0.03 |
| NM_003459 | SLC30A3 | 0.03 | NM_000111 | SLC26A3 | 0.03 |
| NM_004414 | DSCR1 | 0.03 | NM_005340 | HINT1 | 0.03 |
| NM_018972 | GDAP1 | 0.03 | NM_002621 | PFC | 0.03 |
| NM_002354 | TACSTD1 | 0.03 | XM_374831 | SH3MD1 | 0.03 |
| NM_006989 | RASA4 | 0.03 | NM_004038 | AMY1A | 0.03 |
| NM_001897 | CSPG4 | 0.03 | NM_005893 | CCIN | 0.03 |
| NM_001684 | ATP2B4 | 0.03 | NM_001509 | GPX5 | 0.03 |
| NM_024017 | HOXB9 | 0.03 | NM_002465 | MYBPC1 | 0.03 |
| NM_005367 | MAGEA12 | 0.03 | NM_002488 | NDUFA2 | 0.03 |
| NM_004542 | NDUFA3 | 0.03 | NM_002409 | MGAT3 | 0.03 |
| NM_015657 | ABCA12 | 0.03 | NM_003044 | SLC6A12 | 0.03 |
| NM_003007 | SEMG1 | 0.03 | NM_018412 | ST7 | 0.03 |
| NM_021114 | SPINK2 | 0.03 | NM_003321 | TUFM | 0.03 |
| NM_007003 | GAGEC1 | 0.03 | NM_004851 | NAPSA | 0.03 |
| XM_371227 | CROCC | 0.03 | NM_005498 | AP1M2 | 0.03 |
| NM_014751 | MTSS1 | 0.03 | XM_375837 | FAM34A | 0.03 |
| NM_006636 | MTHFD2 | 0.03 | NM_014599 | MAGED2 | 0.03 |
| NM_015116 | LRCH1 | 0.03 | XM_035825 | KIAA0143 | 0.03 |
| NM_032182 | KIAA0157 | 0.03 | XM_166573 | KIAA0895 | 0.03 |
| NM_015493 | ANKRD25 | 0.03 | XM_166254 | ODZ4 | 0.03 |
| NM_013272 | SLCO3A1 | 0.03 | NM_024684 | PTD015 | 0.03 |
| NM_015631 | C10ORF61 | 0.03 | XM_372774 | DJ159A19.3 | 0.03 |
| NM_014181 | HSPC159 | 0.03 | NM_173467 | MT | 0.03 |
| NM_013268 | LGALS13 | 0.03 | NM_014367 | E2IG5 | 0.03 |
| NM_014552 | TFCP2L2 | 0.03 | NM_016100 | NAT5 | 0.03 |
| NM_016582 | SLC15A3 | 0.03 | NM_015886 | PI15 | 0.03 |
| NM_181354 | OXR1 | 0.03 | NM_018015 | FLJ10178 | 0.03 |
| NM_018017 | C10ORF118 | 0.03 | NM_018194 | MART2 | 0.03 |
| NM_018307 | RHOT1 | 0.03 | NM_015492 | DKFZP434H132 | 0.03 |
| NM_020187 | DC12 | 0.03 | NM_020425 | C6ORF162 | 0.03 |
| XM_375085 | KIAA1393 | 0.03 | NM_020693 | DSCAML1 | 0.03 |
| XM_049351 | KIAA1600 | 0.03 | NM_020834 | KIAA1443 | 0.03 |
| XM_044178 | KIAA1211 | 0.03 | NM_021810 | CDH26 | 0.03 |
| NM_021814 | ELOVL5 | 0.03 | NM_022779 | DDX31 | 0.03 |

| | | |
|---|---|---|
| NM_022481 | CENTD3 | 0.03 |
| NM_022145 | FKSG14 | 0.03 |
| NM_024332 | CXORF53 | 0.03 |
| NM_024312 | MGC4170 | 0.03 |
| NM_024816 | RABEP2 | 0.03 |
| NM_024986 | FLJ12331 | 0.03 |
| NM_030917 | FIP1L1 | 0.03 |
| NM_030966 | KRTAP1-3 | 0.03 |
| NM_032367 | ZBED3 | 0.03 |
| NM_032883 | C20ORF100 | 0.03 |
| NM_052875 | MGC10485 | 0.03 |
| NM_139160 | LOC91614 | 0.03 |
| NM_080741 | NEU4 | 0.03 |
| NM_017873 | ASB6 | 0.03 |
| NM_175878 | MGC57211 | 0.03 |
| NM_144720 | MARLIN1 | 0.03 |
| XM_379651 | C9ORF44 | 0.03 |
| NM_173533 | TDRD5 | 0.03 |
| NM_152732 | C6ORF206 | 0.03 |
| NM_181784 | SPRED2 | 0.03 |
| XM_208524 | LOC283129 | 0.03 |
| NM_153376 | FLJ90575 | 0.03 |
| NM_173632 | FLJ38288 | 0.03 |
| XM_378374 | LOC338809 | 0.03 |
| NM_178173 | LOC339834 | 0.03 |
| NM_198552 | MGC15887 | 0.03 |
| XM_371809 | C6ORF205 | 0.03 |
| NM_207454 | FLJ44815 | 0.03 |
| XM_089384 | LOC164118 | 0.03 |
| XM_293332 | LOC340549 | 0.03 |
| XM_084852 | LOC144404 | 0.03 |
| XM_371269 | LOC388650 | 0.03 |
| XM_378841 | LOC400741 | 0.03 |
| XM_371639 | LOC389118 | 0.03 |
| XM_379243 | LOC401106 | 0.03 |
| XM_371666 | LOC389152 | 0.03 |
| XM_379398 | LOC401237 | 0.03 |
| XM_380034 | LOC402687 | 0.03 |
| XM_372330 | LOC390009 | 0.03 |
| XM_373558 | LOC387903 | 0.03 |
| XM_375434 | LOC400590 | 0.03 |
| XM_380128 | LOC402563 | 0.03 |
| NM_017593 | BMP2K | 0.04 |
| NM_000675 | ADORA2A | 0.04 |
| NM_020061 | OPN1LW | 0.04 |
| NM_006194 | PAX9 | 0.04 |

| | | |
|---|---|---|
| NM_022823 | FNDC4 | 0.03 |
| NM_024033 | MGC5242 | 0.03 |
| NM_024593 | FLJ11767 | 0.03 |
| NM_024811 | FLJ12529 | 0.03 |
| NM_153448 | ESX1L | 0.03 |
| NM_025082 | FLJ13111 | 0.03 |
| NM_031285 | PP1057 | 0.03 |
| NM_032573 | TSP-NY | 0.03 |
| NM_032566 | ECG2 | 0.03 |
| NM_033112 | C6ORF153 | 0.03 |
| NM_033438 | SLAMF9 | 0.03 |
| NM_177402 | SYT2 | 0.03 |
| XM_378419 | LOC144766 | 0.03 |
| NM_153221 | CILP2 | 0.03 |
| NM_144709 | FLJ32312 | 0.03 |
| NM_178840 | MGC24047 | 0.03 |
| NM_153330 | DNAJB8 | 0.03 |
| NM_152629 | ZNF515 | 0.03 |
| NM_173545 | C2ORF13 | 0.03 |
| NM_152763 | MGC26989 | 0.03 |
| NM_172239 | GOR | 0.03 |
| XM_378452 | LOC253970 | 0.03 |
| NM_176782 | MGC27169 | 0.03 |
| XM_210048 | LOC286436 | 0.03 |
| NM_198550 | FLJ36760 | 0.03 |
| XM_294370 | GNAT3 | 0.03 |
| NM_203349 | RALP | 0.03 |
| NM_207457 | FLJ46126 | 0.03 |
| XM_293405 | LOC340595 | 0.03 |
| XM_291208 | LOC340246 | 0.03 |
| XM_166926 | LOC219968 | 0.03 |
| XM_066690 | LOC139425 | 0.03 |
| XM_371479 | LOC388928 | 0.03 |
| XM_377797 | LOC402123 | 0.03 |
| XM_374029 | LOC389089 | 0.03 |
| XM_371797 | LOC389365 | 0.03 |
| XM_380089 | LOC402463 | 0.03 |
| XM_373243 | LOC392197 | 0.03 |
| XM_373453 | LOC387654 | 0.03 |
| XM_370939 | LOC388221 | 0.03 |
| NM_001002760 | BPY2B | 0.03 |
| NM_213656 | KA35 | 0.03 |
| NM_005232 | EPHA1 | 0.04 |
| NM_001462 | FPRL1 | 0.04 |
| NM_005998 | CCT3 | 0.04 |
| NM_018901 | PCDHA10 | 0.04 |

| | | | | | |
|---|---|---|---|---|---|
| NM_004364 | CEBPA | 0.04 | NM_004509 | SP110 | 0.04 |
| NM_001063 | TF | 0.04 | NM_017410 | HOXC13 | 0.04 |
| NM_002199 | IRF2 | 0.04 | NM_013432 | NFKBIL2 | 0.04 |
| NM_012114 | CASP14 | 0.04 | NM_001907 | CTRL | 0.04 |
| NM_004092 | ECHS1 | 0.04 | NM_006895 | HNMT | 0.04 |
| NM_004531 | MOCS2 | 0.04 | NM_000511 | FUT2 | 0.04 |
| NM_003251 | THRSP | 0.04 | NM_003968 | UBE1C | 0.04 |
| NM_003641 | IFITM1 | 0.04 | NM_003587 | DHX16 | 0.04 |
| NM_031915 | SETDB2 | 0.04 | NM_006999 | POLS | 0.04 |
| NM_003626 | PPFIA1 | 0.04 | NM_004458 | ACSL4 | 0.04 |
| NM_024670 | SUV39H2 | 0.04 | NM_000108 | DLD | 0.04 |
| NM_000627 | LTBP1 | 0.04 | NM_002890 | RASA1 | 0.04 |
| NM_002605 | PDE8A | 0.04 | NM_000624 | SERPINA5 | 0.04 |
| NM_007144 | RNF110 | 0.04 | NM_032663 | USP30 | 0.04 |
| NM_002055 | GFAP | 0.04 | NM_000486 | AQP2 | 0.04 |
| NM_004183 | VMD2 | 0.04 | NM_001134 | AFP | 0.04 |
| NM_022449 | RAB17 | 0.04 | NM_015986 | CRLF3 | 0.04 |
| NM_152277 | DC-UBP | 0.04 | NM_021631 | FKSG2 | 0.04 |
| NM_006204 | PDE6C | 0.04 | NM_002701 | POU5F1 | 0.04 |
| NM_000339 | SLC12A3 | 0.04 | NM_014426 | SNX5 | 0.04 |
| NM_006379 | SEMA3C | 0.04 | NM_021914 | CFL2 | 0.04 |
| NM_001299 | CNN1 | 0.04 | NM_001633 | AMBP | 0.04 |
| NM_004928 | C21ORF2 | 0.04 | NM_001219 | CALU | 0.04 |
| NM_000778 | CYP4A11 | 0.04 | NM_014618 | DBC1 | 0.04 |
| NM_002248 | KCNN1 | 0.04 | NM_002283 | KRTHB5 | 0.04 |
| NM_006233 | POLR2I | 0.04 | NM_021049 | MAGEA5 | 0.04 |
| NM_005692 | ABCF2 | 0.04 | NM_007188 | ABCB8 | 0.04 |
| NM_002663 | PLD2 | 0.04 | NM_002927 | RGS13 | 0.04 |
| NM_006011 | SIAT8B | 0.04 | NM_005452 | C6ORF11 | 0.04 |
| NM_003705 | SLC25A12 | 0.04 | NM_004917 | KLK4 | 0.04 |
| NM_003765 | STX10 | 0.04 | NM_014650 | ZNF432 | 0.04 |
| NM_014763 | MRPL19 | 0.04 | NM_005801 | SUI1 | 0.04 |
| NM_007184 | NISCH | 0.04 | NM_022042 | SLC26A1 | 0.04 |
| NM_006550 | FSBP | 0.04 | NM_015140 | KIAA0153 | 0.04 |
| XM_043118 | KIAA0286 | 0.04 | NM_012100 | DNPEP | 0.04 |
| NM_012071 | COMMD3 | 0.04 | NM_014291 | GCAT | 0.04 |
| NM_018094 | GSPT2 | 0.04 | XM_044334 | KIAA0318 | 0.04 |
| NM_014184 | HSPC163 | 0.04 | NM_017527 | LY6K | 0.04 |
| NM_018013 | FLJ10159 | 0.04 | NM_018154 | ASF1B | 0.04 |
| XM_371575 | FNBP3 | 0.04 | NM_018371 | CHGN | 0.04 |
| NM_018285 | C15ORF12 | 0.04 | NM_017987 | RUFY2 | 0.04 |
| NM_021729 | VPS11 | 0.04 | NM_018704 | DKFZP547A023 | 0.04 |
| XM_035601 | DLGAP3 | 0.04 | NM_015409 | EP400 | 0.04 |
| NM_021182 | HB-1 | 0.04 | NM_032476 | MRPS6 | 0.04 |
| NM_022092 | CHTF18 | 0.04 | NM_022783 | DEPDC6 | 0.04 |
| NM_023927 | NS3TP2 | 0.04 | NM_023932 | EGFL9 | 0.04 |

| | | | | | |
|---|---|---|---|---|---|
| NM_022489 | FLJ22056 | 0.04 | NM_024615 | FLJ21308 | 0.04 |
| NM_024749 | FLJ12505 | 0.04 | NM_018527 | FLJ22054 | 0.04 |
| NM_025128 | MUS81 | 0.04 | NM_025181 | SLC35F5 | 0.04 |
| NM_024915 | TFCP2L3 | 0.04 | NM_032137 | DKFZP434N1817 | 0.04 |
| NM_030821 | PLA2G12A | 0.04 | NM_030918 | SNX27 | 0.04 |
| NM_031924 | RSHL2 | 0.04 | NM_031937 | TBC1D10 | 0.04 |
| NM_031210 | C14ORF156 | 0.04 | NM_032265 | ZMYND15 | 0.04 |
| NM_032785 | FLJ14442 | 0.04 | NM_031961 | KRTAP9-2 | 0.04 |
| NM_033280 | LOC90701 | 0.04 | NM_152347 | FLJ40342 | 0.04 |
| NM_152412 | ZNF572 | 0.04 | NM_152383 | MGC42174 | 0.04 |
| XM_375404 | C17ORF38 | 0.04 | NM_012363 | OR1N1 | 0.04 |
| NM_080878 | ITLN2 | 0.04 | NM_144686 | TMC4 | 0.04 |
| NM_153812 | PHF13 | 0.04 | XM_371619 | FGD5 | 0.04 |
| NM_001001661 | LOC155054 | 0.04 | NM_152562 | CDCA2 | 0.04 |
| NM_133637 | DQX1 | 0.04 | NM_152610 | FLJ35728 | 0.04 |
| NM_203498 | FLJ23865 | 0.04 | NM_147194 | MGC35361 | 0.04 |
| NM_173565 | LOC222967 | 0.04 | XM_378211 | LOC254312 | 0.04 |
| XM_374069 | LOC254808 | 0.04 | NM_173579 | FLJ40224 | 0.04 |
| NM_152771 | MGC39696 | 0.04 | NM_173855 | LOC283385 | 0.04 |
| XM_379514 | LOC340340 | 0.04 | XM_373440 | CSS3 | 0.04 |
| XM_291322 | KIAA2001 | 0.04 | NM_198506 | FLJ44691 | 0.04 |
| NM_198521 | FLJ25323 | 0.04 | XM_379203 | LOC348801 | 0.04 |
| NM_198285 | LOC349136 | 0.04 | NM_207409 | UNQ3045 | 0.04 |
| XM_117224 | LOC200312 | 0.04 | XM_373883 | LOC388731 | 0.04 |
| XM_377579 | LOC401945 | 0.04 | XM_373837 | LOC388616 | 0.04 |
| XM_374023 | LOC389071 | 0.04 | XM_374130 | LOC389310 | 0.04 |
| XM_376342 | LOC401154 | 0.04 | XM_374088 | LOC389230 | 0.04 |
| XM_371741 | LOC389293 | 0.04 | XM_379327 | LOC401182 | 0.04 |
| XM_374099 | LOC389247 | 0.04 | XM_374734 | LOC392726 | 0.04 |
| XM_378456 | LOC400212 | 0.04 | XM_370597 | LOC387733 | 0.04 |
| XM_370542 | LOC387648 | 0.04 | XM_373632 | LOC388131 | 0.04 |
| XM_378523 | LOC400388 | 0.04 | XM_378545 | LOC400456 | 0.04 |
| XM_375272 | LOC400468 | 0.04 | XM_373653 | LOC388193 | 0.04 |
| NM_001001850 | MGC21382 | 0.04 | NM_001005214 | FLJ25811 | 0.04 |
| XM_372213 | USP27X | 0.04 | NM_001004064 | OR8J3 | 0.04 |
| NM_001005567 | OR51B5 | 0.04 | NM_022766 | CERK | 0.05 |
| NM_001825 | CKMT2 | 0.05 | NM_004071 | CLK1 | 0.05 |
| NM_013302 | EEF2K | 0.05 | NM_002755 | MAP2K1 | 0.05 |
| NM_152529 | GPR155 | 0.05 | NM_017719 | SNRK | 0.05 |
| NM_003565 | ULK1 | 0.05 | NM_004226 | STK17B | 0.05 |
| NM_175911 | MGC40047 | 0.05 | NM_139209 | GRK7 | 0.05 |
| NM_145242 | LOC115131 | 0.05 | NM_023919 | TAS2R7 | 0.05 |
| NM_001707 | BCL7B | 0.05 | NM_006834 | RAB32 | 0.05 |
| NM_014836 | RHOBTB1 | 0.05 | NM_000425 | L1CAM | 0.05 |
| NM_001804 | CDX1 | 0.05 | NM_133476 | ZNF384 | 0.05 |
| NM_022363 | LHX5 | 0.05 | NM_005149 | TBX19 | 0.05 |

| | | | | | |
|---|---|---|---|---|---|
| NM_152910 | DGKH | 0.05 | NM_025211 | GKAP1 | 0.05 |
| NM_000408 | GPD2 | 0.05 | NM_024119 | LGP2 | 0.05 |
| NM_020315 | PDXP | 0.05 | NM_005809 | PRDX2 | 0.05 |
| NM_016442 | ARTS-1 | 0.05 | NM_000175 | GPI | 0.05 |
| NM_001527 | HDAC2 | 0.05 | NM_021111 | RECK | 0.05 |
| NM_005798 | RFP2 | 0.05 | NM_182488 | USP12 | 0.05 |
| NM_003357 | SCGB1A1 | 0.05 | NM_139245 | PPM1L | 0.05 |
| NM_000610 | CD44 | 0.05 | NM_004898 | CLOCK | 0.05 |
| NM_003877 | SOCS2 | 0.05 | NM_014249 | NR2E3 | 0.05 |
| NM_002291 | LAMB1 | 0.05 | NM_001170 | AQP7 | 0.05 |
| NM_001666 | ARHGAP4 | 0.05 | NM_002476 | MYL4 | 0.05 |
| NM_005775 | SCAM-1 | 0.05 | NM_001104 | ACTN3 | 0.05 |
| NM_002095 | GTF2E2 | 0.05 | NM_002247 | KCNMA1 | 0.05 |
| NM_001234 | CAV3 | 0.05 | XM_370947 | ERN2 | 0.05 |
| NM_018068 | PIWIL2 | 0.05 | NM_016445 | PLEK2 | 0.05 |
| NM_006269 | RP1 | 0.05 | NM_003035 | SIL | 0.05 |
| NM_080867 | SOCS4 | 0.05 | NM_012416 | RANBP6 | 0.05 |
| NM_015490 | SEC31L2 | 0.05 | NM_001516 | GTF2H3 | 0.05 |
| NM_004477 | FRG1 | 0.05 | NM_018284 | GBP3 | 0.05 |
| NM_005558 | LAD1 | 0.05 | NM_024016 | HOXB8 | 0.05 |
| NM_004997 | MYBPH | 0.05 | NM_018436 | ALLC | 0.05 |
| NM_144650 | ADHFE1 | 0.05 | NM_002915 | RFC3 | 0.05 |
| NM_003057 | SLC22A1 | 0.05 | NM_203288 | RP9 | 0.05 |
| NM_003983 | SLC7A6 | 0.05 | NM_004711 | SYNGR1 | 0.05 |
| NM_014723 | SNPH | 0.05 | NM_014734 | KIAA0247 | 0.05 |
| NM_014314 | DDX58 | 0.05 | NM_015559 | SETBP1 | 0.05 |
| NM_020999 | NEUROG3 | 0.05 | NM_018141 | MRPS10 | 0.05 |
| NM_017998 | C9ORF40 | 0.05 | XM_371595 | DOCK10 | 0.05 |
| NM_018320 | RNF121 | 0.05 | NM_018346 | FLJ11164 | 0.05 |
| XM_033853 | ZNF630 | 0.05 | NM_020924 | ZBTB26 | 0.05 |
| NM_022059 | CXCL16 | 0.05 | NM_021937 | SELB | 0.05 |
| NM_022100 | MRPS14 | 0.05 | NM_022463 | NXN | 0.05 |
| NM_022154 | SLC39A8 | 0.05 | NM_030789 | HM13 | 0.05 |
| NM_053005 | HCCA2 | 0.05 | XM_375153 | DKFZP547L112 | 0.05 |
| NM_030972 | ZNF611 | 0.05 | NM_032310 | C9ORF89 | 0.05 |
| XM_040486 | KIAA1789 | 0.05 | NM_032600 | NYD-SP17 | 0.05 |
| NM_031922 | REPS1 | 0.05 | NM_144580 | MGC31963 | 0.05 |
| NM_153034 | ZNF488 | 0.05 | XM_059702 | FLJ36748 | 0.05 |
| NM_152424 | FLJ39827 | 0.05 | NM_152512 | FLJ25421 | 0.05 |
| NM_147193 | FLJ36155 | 0.05 | NM_152596 | MGC33637 | 0.05 |
| NM_207337 | LOC196394 | 0.05 | NM_173540 | FUT11 | 0.05 |
| NM_174917 | LOC197322 | 0.05 | NM_153368 | CX40.1 | 0.05 |
| NM_198465 | NRK | 0.05 | XM_375334 | LOC283901 | 0.05 |
| XM_208333 | MGC48637 | 0.05 | NM_173086 | KRT6E | 0.05 |
| NM_175739 | SERPINA9 | 0.05 | XM_352847 | LOC340529 | 0.05 |
| NM_182971 | COX8C | 0.05 | NM_203370 | LOC389119 | 0.05 |

| | | |
|---|---|---|
| NM_198695 | KRTAP18-8 | 0.05 |
| XM_060171 | LOC126772 | 0.05 |
| XM_065050 | LOC126370 | 0.05 |
| XM_373059 | LOC391747 | 0.05 |
| XM_372099 | LOC389737 | 0.05 |
| XM_373560 | LOC387910 | 0.05 |
| XM_373505 | LOC387783 | 0.05 |
| XM_373382 | LOC392563 | 0.05 |
| NM_173351 | OR6B3 | 0.05 |
| NM_001569 | IRAK1 | 0.06 |
| NM_006641 | CCR9 | 0.06 |
| NM_005682 | GPR56 | 0.06 |
| NM_012068 | ATF5 | 0.06 |
| NM_005251 | FOXC2 | 0.06 |
| NM_003199 | TCF4 | 0.06 |
| NM_020664 | DECR2 | 0.06 |
| NM_022132 | MCCC2 | 0.06 |
| NM_000156 | GAMT | 0.06 |
| NM_018417 | SAC | 0.06 |
| NM_015547 | THEA | 0.06 |
| NM_014254 | TMEM5 | 0.06 |
| NM_001983 | ERCC1 | 0.06 |
| NM_006087 | TUBB5 | 0.06 |
| NM_006154 | NEDD4 | 0.06 |
| NM_198402 | PTPLB | 0.06 |
| NM_004186 | SEMA3F | 0.06 |
| NM_002514 | NOV | 0.06 |
| NM_019851 | FGF20 | 0.06 |
| NM_003051 | SLC16A1 | 0.06 |
| NM_080626 | BRI3BP | 0.06 |
| NM_001975 | ENO2 | 0.06 |
| NM_003897 | IER3 | 0.06 |
| NM_002439 | MSH3 | 0.06 |
| NM_001448 | GPC4 | 0.06 |
| NM_178120 | DLX1 | 0.06 |
| NM_002630 | PGC | 0.06 |
| NM_005386 | NNAT | 0.06 |
| NM_019848 | SLC10A3 | 0.06 |
| NM_153683 | KL | 0.06 |
| NM_005830 | MRPS31 | 0.06 |
| NM_005494 | DNAJB6 | 0.06 |
| NM_014869 | KIAA0763 | 0.06 |
| NM_006416 | SLC35A1 | 0.06 |
| NM_015415 | DKFZP564B167 | 0.06 |
| NM_013363 | PCOLCE2 | 0.06 |
| NM_014408 | TRAPPC3 | 0.06 |
| NM_207490 | FLJ45721 | 0.05 |
| NM_207450 | FLJ27243 | 0.05 |
| XM_295257 | LOC340477 | 0.05 |
| XM_374202 | LOC401303 | 0.05 |
| XM_373550 | LOC387888 | 0.05 |
| XM_373570 | LOC387939 | 0.05 |
| XM_371117 | LOC388476 | 0.05 |
| XM_377713 | LOC402055 | 0.05 |
| NM_004442 | EPHB2 | 0.06 |
| NM_025194 | ITPKC | 0.06 |
| NM_019897 | OR2S2 | 0.06 |
| NM_004282 | BAG2 | 0.06 |
| NM_005652 | TERF2 | 0.06 |
| NM_016169 | SUFU | 0.06 |
| NM_014256 | B3GNT3 | 0.06 |
| NM_002201 | ISG20 | 0.06 |
| NM_006903 | PPA2 | 0.06 |
| NM_003282 | TNNI2 | 0.06 |
| NM_003360 | UGT8 | 0.06 |
| NM_022085 | TXNDC5 | 0.06 |
| NM_002872 | RAC2 | 0.06 |
| NM_004218 | RAB11B | 0.06 |
| NM_024524 | AFURS1 | 0.06 |
| NM_006140 | CSF2RA | 0.06 |
| NM_080876 | DUSP19 | 0.06 |
| NM_003839 | TNFRSF11A | 0.06 |
| NM_005585 | SMAD6 | 0.06 |
| NM_000037 | ANK1 | 0.06 |
| NM_000558 | HBA1 | 0.06 |
| NM_005106 | DLEC1 | 0.06 |
| NM_152653 | UBE2E2 | 0.06 |
| NM_000278 | PAX2 | 0.06 |
| NM_001829 | CLCN3 | 0.06 |
| NM_005548 | KARS | 0.06 |
| NM_004797 | ACDC | 0.06 |
| NM_006159 | NELL2 | 0.06 |
| NM_020186 | ACN9 | 0.06 |
| NM_003734 | AOC3 | 0.06 |
| NM_004729 | ZBED1 | 0.06 |
| XM_376647 | SEMA3E | 0.06 |
| XM_376540 | PHACTR2 | 0.06 |
| NM_007176 | C14ORF1 | 0.06 |
| NM_007242 | DDX19 | 0.06 |
| NM_015594 | DKFZP434O047 | 0.06 |
| NM_152414 | BHLHB5 | 0.06 |
| NM_014428 | TJP3 | 0.06 |

| | | |
|---|---|---|
| NM_015873 | VILL | 0.06 |
| NM_017748 | FLJ20291 | 0.06 |
| NM_017957 | EPN3 | 0.06 |
| NM_017860 | FLJ20519 | 0.06 |
| NM_018292 | QRSL1 | 0.06 |
| NM_020970 | KIAA1641 | 0.06 |
| NM_024325 | MGC10715 | 0.06 |
| NM_024309 | TNIP2 | 0.06 |
| NM_030770 | TMPRSS5 | 0.06 |
| NM_031917 | ANGPTL6 | 0.06 |
| NM_032042 | DKFZP564D172 | 0.06 |
| NM_133443 | GPT2 | 0.06 |
| NM_152362 | MGC17791 | 0.06 |
| NM_138807 | LOC132001 | 0.06 |
| NM_138963 | RPS4Y2 | 0.06 |
| NM_152527 | SLC16A14 | 0.06 |
| NM_153246 | MGC45491 | 0.06 |
| NM_182552 | MGC43690 | 0.06 |
| XM_113978 | LOC284352 | 0.06 |
| NM_183241 | LOC286257 | 0.06 |
| XM_379551 | LOC349196 | 0.06 |
| XM_211086 | LOC283553 | 0.06 |
| NM_206833 | CTXN1 | 0.06 |
| XM_114222 | LOC200373 | 0.06 |
| XM_295091 | LOC339875 | 0.06 |
| XM_088684 | LOC158830 | 0.06 |
| XM_377555 | LOC401934 | 0.06 |
| XM_374059 | LOC389163 | 0.06 |
| XM_373249 | LOC392217 | 0.06 |
| XM_370835 | LOC388078 | 0.06 |
| NM_001001674 | OR4F15 | 0.06 |
| NM_001726 | BRDT | 0.07 |
| NM_000677 | ADORA3 | 0.07 |
| NM_080818 | GPR80 | 0.07 |
| NM_015032 | APRIN | 0.07 |
| NM_001520 | GTF3C1 | 0.07 |
| NM_006167 | NKX3-1 | 0.07 |
| NM_000032 | ALAS2 | 0.07 |
| NM_004453 | ETFDH | 0.07 |
| NM_003273 | TM7SF2 | 0.07 |
| NM_002423 | MMP7 | 0.07 |
| NM_002828 | PTPN2 | 0.07 |
| NM_032603 | LOXL3 | 0.07 |
| NM_005215 | DCC | 0.07 |
| NM_004844 | SH3BP5 | 0.07 |
| NM_002957 | RXRA | 0.07 |
| NM_016526 | BET1L | 0.06 |
| NM_017956 | FLJ20772 | 0.06 |
| NM_017996 | DET1 | 0.06 |
| NM_018171 | DIP13B | 0.06 |
| NM_020178 | CA10 | 0.06 |
| NM_021831 | FLJ21839 | 0.06 |
| NM_024713 | FLJ22557 | 0.06 |
| NM_024870 | DEPDC2 | 0.06 |
| NM_024913 | FLJ21986 | 0.06 |
| NM_032024 | C10ORF11 | 0.06 |
| XM_038436 | KIAA1786 | 0.06 |
| NM_080650 | MGC14798 | 0.06 |
| NM_133261 | GIPC3 | 0.06 |
| NM_173494 | MGC35261 | 0.06 |
| NM_153355 | TCBA1 | 0.06 |
| XM_375358 | FLJ25339 | 0.06 |
| NM_145756 | ZNF396 | 0.06 |
| NM_182568 | FLJ36492 | 0.06 |
| NM_181614 | KRTAP19-7 | 0.06 |
| NM_198943 | MGC52000 | 0.06 |
| NM_207430 | FLJ46266 | 0.06 |
| NM_207442 | FLJ39779 | 0.06 |
| XM_292820 | LOC342979 | 0.06 |
| XM_097753 | LOC149913 | 0.06 |
| XM_209824 | LOC285929 | 0.06 |
| XM_211040 | LOC283440 | 0.06 |
| XM_374007 | LOC389029 | 0.06 |
| XM_374066 | LOC389184 | 0.06 |
| XM_372102 | LOC389742 | 0.06 |
| XM_373812 | LOC388568 | 0.06 |
| NM_001005237 | OR51G1 | 0.06 |
| NM_002754 | MAPK13 | 0.07 |
| NM_000706 | AVPR1A | 0.07 |
| NM_013937 | OR11A1 | 0.07 |
| NM_001746 | CANX | 0.07 |
| NM_004819 | SYMPK | 0.07 |
| NM_005919 | MEF2B | 0.07 |
| NM_002573 | PAFAH1B3 | 0.07 |
| NM_000364 | TNNT2 | 0.07 |
| NM_006660 | CLPX | 0.07 |
| XM_086650 | PPP4R1L | 0.07 |
| NM_018299 | FLJ11011 | 0.07 |
| NM_003979 | RAI3 | 0.07 |
| NM_005401 | PTPN14 | 0.07 |
| NM_006850 | IL24 | 0.07 |
| NM_003867 | FGF17 | 0.07 |

| | | | | | |
|---|---|---|---|---|---|
| NM_004140 | LLGL1 | 0.07 | NM_007234 | DCTN3 | 0.07 |
| NM_002268 | KPNA4 | 0.07 | NM_000112 | SLC26A2 | 0.07 |
| NM_003388 | CYLN2 | 0.07 | NM_001239 | CCNH | 0.07 |
| NM_015344 | LEPROTL1 | 0.07 | NM_173470 | LOC93380 | 0.07 |
| NM_002158 | HTLF | 0.07 | NM_005742 | TXNDC7 | 0.07 |
| NM_005831 | NDP52 | 0.07 | NM_018702 | ADARB2 | 0.07 |
| NM_001830 | CLCN4 | 0.07 | NM_000717 | CA4 | 0.07 |
| NM_004317 | ASNA1 | 0.07 | NM_020991 | CSH2 | 0.07 |
| NM_002620 | PF4V1 | 0.07 | NM_005083 | U2AF1L1 | 0.07 |
| NM_004886 | APBA3 | 0.07 | NM_003787 | NOL4 | 0.07 |
| NM_005463 | HNRPDL | 0.07 | NM_006040 | HS3ST4 | 0.07 |
| NM_006355 | TRIM38 | 0.07 | NM_007224 | NXPH4 | 0.07 |
| NM_012437 | SNAPAP | 0.07 | NM_015537 | NELF | 0.07 |
| NM_015441 | OLFML2B | 0.07 | XM_371960 | KIAA1277 | 0.07 |
| NM_012449 | STEAP | 0.07 | NM_015990 | KLHL5 | 0.07 |
| NM_013386 | SLC25A24 | 0.07 | NM_014591 | KCNIP2 | 0.07 |
| NM_014306 | HSPC117 | 0.07 | NM_018078 | FLJ10378 | 0.07 |
| NM_017712 | PGPEP1 | 0.07 | NM_018696 | ELAC1 | 0.07 |
| NM_019854 | HRMT1L4 | 0.07 | XM_049695 | VANGL2 | 0.07 |
| NM_020815 | PCDH10 | 0.07 | NM_020973 | GBA3 | 0.07 |
| NM_033051 | TSCOT | 0.07 | NM_022372 | GBL | 0.07 |
| NM_024067 | C7ORF26 | 0.07 | NM_024898 | FAM31C | 0.07 |
| NM_030802 | LOC81558 | 0.07 | NM_032164 | ZNF394 | 0.07 |
| NM_031484 | MRVLDC1 | 0.07 | NM_032273 | DKFZP586C1924 | 0.07 |
| NM_032728 | C9ORF67 | 0.07 | XM_030896 | LOC90321 | 0.07 |
| NM_145232 | LOC90353 | 0.07 | NM_052891 | PGLYRP3 | 0.07 |
| XM_376008 | LOC91464 | 0.07 | NM_138779 | LOC93081 | 0.07 |
| NM_139241 | FGD4 | 0.07 | NM_153217 | MGC13034 | 0.07 |
| NM_138571 | HINT3 | 0.07 | NM_032625 | C7ORF13 | 0.07 |
| NM_144629 | C2ORF11 | 0.07 | NM_182505 | C9ORF85 | 0.07 |
| NM_144717 | MGC34923 | 0.07 | NM_207326 | LOC149134 | 0.07 |
| NM_138482 | LOC151534 | 0.07 | NM_145284 | LOC159090 | 0.07 |
| NM_153364 | MGC39520 | 0.07 | XM_378866 | LOC199899 | 0.07 |
| XM_166227 | MPEG1 | 0.07 | NM_194299 | LOC221711 | 0.07 |
| NM_203422 | LOC221091 | 0.07 | NM_174936 | PCSK9 | 0.07 |
| XM_375516 | LOC284121 | 0.07 | XM_290817 | FLJ34907 | 0.07 |
| NM_182565 | MGC29814 | 0.07 | XM_378642 | LOC284009 | 0.07 |
| NM_173674 | DCBLD1 | 0.07 | NM_181607 | KRTAP19-1 | 0.07 |
| XM_379582 | LOC286177 | 0.07 | NM_182761 | LOC340069 | 0.07 |
| NM_198998 | AQP12 | 0.07 | NM_207383 | FLJ42289 | 0.07 |
| NM_203390 | LOC389677 | 0.07 | NM_207645 | LOC399947 | 0.07 |
| XM_117236 | LOC200475 | 0.07 | XM_058967 | LOC126037 | 0.07 |
| XM_173119 | LOC255130 | 0.07 | XM_097351 | LOC147975 | 0.07 |
| XM_067605 | LOC131909 | 0.07 | XM_092553 | LOC151320 | 0.07 |
| XM_171766 | LOC255519 | 0.07 | XM_208930 | LOC283953 | 0.07 |
| XM_293918 | LOC345643 | 0.07 | XM_210515 | LOC286561 | 0.07 |

| | | | | | |
|---|---|---|---|---|---|
| XM_371340 | LOC388734 | 0.07 | XM_373871 | LOC388709 | 0.07 |
| XM_378002 | LOC402322 | 0.07 | XM_373513 | LOC387810 | 0.07 |
| XM_378686 | LOC400606 | 0.07 | XM_378688 | LOC400607 | 0.07 |
| XM_294765 | LOC388154 | 0.07 | XM_378755 | LOC400657 | 0.07 |
| NM_001005518 | OR6C65 | 0.07 | NM_004119 | FLT3 | 0.08 |
| NM_002037 | FYN | 0.08 | NM_014030 | GIT1 | 0.08 |
| NM_001883 | CRHR2 | 0.08 | NM_004485 | GNG4 | 0.08 |
| NM_170776 | GPR97 | 0.08 | NM_000956 | PTGER2 | 0.08 |
| NM_000327 | ROM1 | 0.08 | NM_173857 | VN1R4 | 0.08 |
| NM_033056 | PCDH15 | 0.08 | NM_018905 | PCDHA2 | 0.08 |
| NM_002147 | HOXB5 | 0.08 | NM_016044 | CGI-105 | 0.08 |
| NM_001080 | ALDH5A1 | 0.08 | NM_001461 | FMO5 | 0.08 |
| NM_003310 | TSSC1 | 0.08 | NM_018943 | TUBA8 | 0.08 |
| NM_013342 | TFPT | 0.08 | NM_004618 | TOP3A | 0.08 |
| NM_016261 | TUBD1 | 0.08 | NM_005125 | CCS | 0.08 |
| NM_003144 | SSR1 | 0.08 | NM_016156 | MTMR2 | 0.08 |
| NM_144701 | IL23R | 0.08 | NM_006725 | CD6 | 0.08 |
| NM_002205 | ITGA5 | 0.08 | NM_003212 | TDGF1 | 0.08 |
| NM_004469 | FIGF | 0.08 | NM_006322 | TUBGCP3 | 0.08 |
| XM_044622 | COL14A1 | 0.08 | NM_001916 | CYC1 | 0.08 |
| NM_021105 | PLSCR1 | 0.08 | NM_000489 | ATRX | 0.08 |
| NM_015544 | DKFZP564K1964 | 0.08 | NM_032636 | DDA3 | 0.08 |
| NM_017418 | 37226 | 0.08 | NM_020482 | FHL5 | 0.08 |
| NM_005331 | HBQ1 | 0.08 | NM_005557 | KRT16 | 0.08 |
| NM_015368 | PANX1 | 0.08 | NM_001612 | ACRV1 | 0.08 |
| NM_005520 | HNRPH1 | 0.08 | NM_002783 | PSG7 | 0.08 |
| NM_138326 | ACMSD | 0.08 | NM_002937 | RNASE4 | 0.08 |
| NM_021104 | RPL41 | 0.08 | NM_004638 | BAT2 | 0.08 |
| NM_020981 | B3GALT1 | 0.08 | NM_003785 | GAGEB1 | 0.08 |
| NM_014716 | CENTB1 | 0.08 | NM_014258 | SYCP2 | 0.08 |
| NM_014741 | KIAA0652 | 0.08 | NM_015003 | GOLGIN-67 | 0.08 |
| NM_015219 | EXOC7 | 0.08 | NM_015147 | KIAA0582 | 0.08 |
| NM_014615 | KIAA0182 | 0.08 | NM_015475 | DKFZP564F0522 | 0.08 |
| NM_012446 | SSBP2 | 0.08 | NM_012243 | SLC35A3 | 0.08 |
| NM_015530 | GORASP2 | 0.08 | NM_017614 | BHMT2 | 0.08 |
| NM_015617 | PYGO1 | 0.08 | NM_012345 | NUFIP1 | 0.08 |
| NM_014051 | TMEM14A | 0.08 | NM_014463 | LSM3 | 0.08 |
| NM_018668 | VPS33B | 0.08 | NM_015953 | NOSIP | 0.08 |
| NM_018482 | DDEF1 | 0.08 | NM_013439 | PILRA | 0.08 |
| NM_015918 | POP5 | 0.08 | NM_024047 | NUDT9 | 0.08 |
| NM_018066 | FLJ10349 | 0.08 | NM_017693 | BIVM | 0.08 |
| NM_018460 | ARHGAP15 | 0.08 | XM_042301 | KIAA1546 | 0.08 |
| XM_031342 | ZSWIM4 | 0.08 | NM_024019 | NEUROG2 | 0.08 |
| NM_022824 | FBXL17 | 0.08 | NM_024071 | ZFYVE21 | 0.08 |
| NM_024754 | PTCD2 | 0.08 | NM_025159 | CXORF21 | 0.08 |
| NM_031925 | TMPIT | 0.08 | NM_031475 | ESPN | 0.08 |

| | | | | | |
|---|---|---|---|---|---|
| NM_032935 | MT4 | 0.08 | NM_032711 | MGC13090 | 0.08 |
| NM_032717 | MGC11324 | 0.08 | NM_080475 | SERPINB11 | 0.08 |
| NM_033195 | LDHL | 0.08 | XM_375665 | LOC116412 | 0.08 |
| XM_290850 | GRIN3B | 0.08 | NM_053048 | MGC16384 | 0.08 |
| NM_182496 | FLJ40089 | 0.08 | NM_152345 | FLJ25555 | 0.08 |
| NM_173479 | LOC126248 | 0.08 | NM_139281 | WDR36 | 0.08 |
| NM_144648 | FLJ32786 | 0.08 | NM_133455 | EMID1 | 0.08 |
| NM_144669 | FLJ31978 | 0.08 | XM_085836 | KIAA1956 | 0.08 |
| NM_153635 | LOC151835 | 0.08 | NM_145278 | LOC148823 | 0.08 |
| NM_182516 | FLJ32011 | 0.08 | NM_173526 | C14ORF54 | 0.08 |
| XM_372198 | MGC17403 | 0.08 | XM_090885 | C14ORF42 | 0.08 |
| NM_178505 | TMEM26 | 0.08 | NM_152735 | ZBTB9 | 0.08 |
| NM_178518 | FLJ36878 | 0.08 | NM_178548 | AP2E | 0.08 |
| NM_003514 | HIST1H2AM | 0.08 | XM_065026 | LOC126298 | 0.08 |
| XM_292046 | LOC341412 | 0.08 | XM_376062 | LOC400961 | 0.08 |
| XM_372923 | LOC391360 | 0.08 | XM_375714 | LOC400749 | 0.08 |
| XM_372985 | LOC391510 | 0.08 | XM_379501 | LOC401367 | 0.08 |
| XM_373180 | LOC392084 | 0.08 | XM_380040 | LOC402693 | 0.08 |
| XM_378460 | LOC400216 | 0.08 | XM_372496 | LOC390423 | 0.08 |
| XM_374890 | LOC399900 | 0.08 | XM_372544 | LOC390533 | 0.08 |
| NM_001004341 | FLJ16478 | 0.08 | NM_001005245 | OR5M11 | 0.08 |
| NM_001005334 | OR3A4 | 0.08 | NM_016282 | AK3L1 | 0.09 |
| NM_004418 | DUSP2 | 0.09 | NM_016281 | JIK | 0.09 |
| NM_003266 | TLR4 | 0.09 | NM_004101 | F2RL2 | 0.09 |
| NM_014683 | ULK2 | 0.09 | NM_005291 | GPR17 | 0.09 |
| NM_005899 | M17S2 | 0.09 | NM_006696 | BRD8 | 0.09 |
| NM_001519 | BRF1 | 0.09 | NM_012186 | FOXE3 | 0.09 |
| NM_003721 | RFXANK | 0.09 | NM_014112 | TRPS1 | 0.09 |
| NM_003659 | AGPS | 0.09 | NM_006368 | CREB3 | 0.09 |
| NM_002570 | PCSK6 | 0.09 | NM_021957 | GYS2 | 0.09 |
| NM_003558 | PIP5K1B | 0.09 | NM_005079 | TPD52 | 0.09 |
| NM_005412 | SHMT2 | 0.09 | NM_007019 | UBE2C | 0.09 |
| NM_002930 | RIT2 | 0.09 | NM_012300 | FBXW11 | 0.09 |
| NM_007205 | TREX2 | 0.09 | NM_013239 | PR48 | 0.09 |
| NM_002472 | MYH8 | 0.09 | NM_032559 | LOC84643 | 0.09 |
| NM_001558 | IL10RA | 0.09 | NM_003327 | TNFRSF4 | 0.09 |
| NM_002546 | TNFRSF11B | 0.09 | NM_002414 | CD99 | 0.09 |
| NM_004345 | CAMP | 0.09 | NM_007018 | CEP1 | 0.09 |
| NM_018202 | FLJ10747 | 0.09 | NM_013332 | HIG2 | 0.09 |
| NM_002284 | KRTHB6 | 0.09 | NM_016166 | PIAS1 | 0.09 |
| NM_001870 | CPA3 | 0.09 | NM_001900 | CST5 | 0.09 |
| NM_001512 | GSTA4 | 0.09 | NM_004135 | IDH3G | 0.09 |
| NM_000555 | DCX | 0.09 | NM_002774 | KLK6 | 0.09 |
| NM_004285 | H6PD | 0.09 | NM_004838 | HOMER3 | 0.09 |
| XM_375712 | SDC3 | 0.09 | NM_005725 | TSPAN-2 | 0.09 |
| NM_007021 | C10ORF10 | 0.09 | NM_015359 | SLC39A14 | 0.09 |

| | | | | | |
|---|---|---|---|---|---|
| NM_018413 | CHST11 | 0.09 | NM_013349 | SPUF | 0.09 |
| NM_016552 | ANKMY1 | 0.09 | NM_016233 | PADI3 | 0.09 |
| NM_019083 | FLJ10287 | 0.09 | NM_017867 | FLJ20534 | 0.09 |
| NM_018266 | FLJ10902 | 0.09 | NM_018555 | ZNF331 | 0.09 |
| NM_017926 | C14ORF118 | 0.09 | NM_018447 | LOC55831 | 0.09 |
| NM_020685 | HT021 | 0.09 | NM_023016 | C2ORF26 | 0.09 |
| NM_032156 | C1QDC1 | 0.09 | NM_030765 | B3GNT4 | 0.09 |
| NM_024644 | C14ORF169 | 0.09 | NM_024974 | FLJ11800 | 0.09 |
| NM_030776 | ZBP1 | 0.09 | NM_031216 | SEC13L | 0.09 |
| NM_032293 | GARNL3 | 0.09 | NM_032779 | FLJ14397 | 0.09 |
| NM_033058 | TRIM55 | 0.09 | NM_032445 | MEGF11 | 0.09 |
| NM_032815 | FLJ14639 | 0.09 | NM_032505 | TA-KRP | 0.09 |
| XM_379528 | LOC90520 | 0.09 | NM_138425 | GRCC10 | 0.09 |
| NM_033276 | KUB3 | 0.09 | NM_014021 | SSX2IP | 0.09 |
| NM_153340 | MGC46534 | 0.09 | XM_059368 | LOC129607 | 0.09 |
| NM_144651 | FLJ25471 | 0.09 | NM_080725 | C20ORF139 | 0.09 |
| NM_080829 | C20ORF175 | 0.09 | NM_153810 | C10ORF46 | 0.09 |
| NM_080390 | MY048 | 0.09 | NM_080618 | CTCFL | 0.09 |
| NM_148961 | OTOS | 0.09 | NM_152571 | FLJ36779 | 0.09 |
| NM_152638 | MGC26598 | 0.09 | NM_183373 | C6ORF145 | 0.09 |
| XM_376463 | MGC39372 | 0.09 | NM_152693 | MGC34827 | 0.09 |
| NM_152673 | MUC20 | 0.09 | NM_145313 | RASGEF1A | 0.09 |
| XM_378303 | LOC283214 | 0.09 | NM_198481 | UNQ3033 | 0.09 |
| NM_183062 | MPN2 | 0.09 | NM_181846 | HKR2 | 0.09 |
| NM_198571 | FLJ39237 | 0.09 | NM_198545 | LOC374946 | 0.09 |
| XM_293123 | LOC343578 | 0.09 | XM_295200 | LOC340286 | 0.09 |
| XM_098625 | LOC154872 | 0.09 | XM_378947 | LOC400813 | 0.09 |
| XM_372928 | LOC391373 | 0.09 | XM_371846 | LOC389428 | 0.09 |
| XM_377896 | LOC402214 | 0.09 | XM_373219 | LOC392152 | 0.09 |
| XM_374277 | LOC389683 | 0.09 | XM_376838 | LOC401507 | 0.09 |
| XM_372532 | LOC390502 | 0.09 | XM_378708 | LOC400623 | 0.09 |
| XM_370997 | LOC388308 | 0.09 | NM_001001524 | TM6SF2 | 0.09 |
| XM_377062 | LOC401620 | 0.09 | NM_001005274 | OR4A16 | 0.09 |
| NM_018167 | BTBD7 | 0.09 | NM_001005503 | OR11G2 | 0.09 |
| NM_004329 | BMPR1A | 0.1 | NM_004836 | EIF2AK3 | 0.1 |
| NM_002751 | MAPK11 | 0.1 | NM_002401 | MAP3K3 | 0.1 |
| NM_017412 | FZD3 | 0.1 | NM_007227 | GPR45 | 0.1 |
| NM_004061 | CDH12 | 0.1 | NM_003472 | DEK | 0.1 |
| NM_001421 | ELF4 | 0.1 | NM_002699 | POU3F1 | 0.1 |
| NM_005023 | PGGT1B | 0.1 | NM_004470 | FKBP2 | 0.1 |
| NM_001977 | ENPEP | 0.1 | NM_004194 | ADAM22 | 0.1 |
| NM_003072 | SMARCA4 | 0.1 | NM_022571 | GPR135 | 0.1 |
| NM_016362 | GHRL | 0.1 | NM_003395 | WNT9A | 0.1 |
| NM_002506 | NGFB | 0.1 | NM_004451 | ESRRA | 0.1 |
| NM_004732 | KCNAB3 | 0.1 | NM_000254 | MTR | 0.1 |
| NM_006860 | RABL4 | 0.1 | NM_005506 | SCARB2 | 0.1 |

| | | | | | |
|---|---|---|---|---|---|
| NM_001839 | CNN3 | 0.1 | NM_004129 | GUCY1B2 | 0.1 |
| NM_005003 | NDUFAB1 | 0.1 | NM_018674 | ACCN4 | 0.1 |
| NM_006279 | SIAT6 | 0.1 | NM_002818 | PSME2 | 0.1 |
| NM_004187 | JARID1C | 0.1 | NM_006961 | ZNF19 | 0.1 |
| NM_003825 | SNAP23 | 0.1 | XM_044155 | KIAA0446 | 0.1 |
| NM_005469 | PTE1 | 0.1 | NM_005687 | FARSLB | 0.1 |
| NM_006043 | HS3ST2 | 0.1 | NM_006848 | DIPA | 0.1 |
| XM_377338 | KIAA0323 | 0.1 | NM_015335 | THRAP2 | 0.1 |
| XM_171054 | KIAA0527 | 0.1 | NM_015650 | TRAF3IP1 | 0.1 |
| NM_014155 | HSPC063 | 0.1 | NM_016076 | PNAS-4 | 0.1 |
| NM_017628 | FLJ20032 | 0.1 | NM_017923 | FLJ20668 | 0.1 |
| NM_017931 | FLJ20699 | 0.1 | NM_017967 | FLJ20850 | 0.1 |
| NM_018389 | SLC35C1 | 0.1 | NM_018398 | CACNA2D3 | 0.1 |
| NM_183425 | RNPC1 | 0.1 | NM_020207 | C9ORF102 | 0.1 |
| NM_019108 | FLJ12886 | 0.1 | NM_020320 | RARSL | 0.1 |
| NM_023945 | MS4A5 | 0.1 | NM_024303 | ZNF495 | 0.1 |
| NM_030650 | KIAA1715 | 0.1 | NM_031310 | PLVAP | 0.1 |
| NM_032236 | USP48 | 0.1 | NM_032447 | FBN3 | 0.1 |
| NM_032495 | HOP | 0.1 | NM_032520 | GNPTAG | 0.1 |
| NM_052870 | SNAG1 | 0.1 | XM_028067 | MIDN | 0.1 |
| NM_198075 | DKFZP761L1518 | 0.1 | NM_001001410 | MGC24381 | 0.1 |
| XM_371577 | MGC23908 | 0.1 | NM_138698 | LOC91431 | 0.1 |
| NM_145038 | MGC16372 | 0.1 | NM_152349 | MGC45562 | 0.1 |
| NM_207325 | LOC147991 | 0.1 | NM_173797 | PAPD4 | 0.1 |
| XM_091914 | LOC162993 | 0.1 | NM_144970 | MGC39350 | 0.1 |
| NM_198460 | DKFZP686G0786 | 0.1 | XM_378346 | LOC196415 | 0.1 |
| NM_144990 | FLJ23878 | 0.1 | NM_173549 | FLJ39553 | 0.1 |
| XM_375833 | LOC284581 | 0.1 | XM_290712 | MGC46336 | 0.1 |
| NM_178351 | LCE1C | 0.1 | XM_211092 | LOC283583 | 0.1 |
| XM_291569 | LOC343413 | 0.1 | XM_292779 | LOC342918 | 0.1 |
| XM_293903 | LOC345630 | 0.1 | XM_372825 | LOC391194 | 0.1 |
| XM_372970 | LOC391481 | 0.1 | XM_379513 | LOC401396 | 0.1 |
| XM_374406 | LOC392636 | 0.1 | XM_378529 | LOC400400 | 0.1 |
| XM_372630 | LOC390712 | 0.1 | XM_373358 | LOC392509 | 0.1 |
| NM_014365 | HSPB8 | 0.11 | NM_002737 | PRKCA | 0.11 |
| NM_005160 | ADRBK2 | 0.11 | NM_016540 | GPR83 | 0.11 |
| NM_177437 | TAS2R60 | 0.11 | NM_001796 | CDH8 | 0.11 |
| NM_022162 | CARD15 | 0.11 | NM_004225 | MFHAS1 | 0.11 |
| NM_018911 | PCDHA8 | 0.11 | NM_006563 | KLF1 | 0.11 |
| NM_001518 | GTF2I | 0.11 | NM_003202 | TCF7 | 0.11 |
| NM_001360 | DHCR7 | 0.11 | NM_002028 | FNTB | 0.11 |
| NM_002035 | FVT1 | 0.11 | NM_000151 | G6PC | 0.11 |
| NM_003335 | UBE1L | 0.11 | NM_018037 | RALGPS2 | 0.11 |
| NM_000730 | CCKAR | 0.11 | NM_000758 | CSF2 | 0.11 |
| NM_002296 | LBR | 0.11 | NM_002345 | LUM | 0.11 |
| NM_021076 | NEFH | 0.11 | NM_001681 | ATP2A2 | 0.11 |

| | | |
|---|---|---|
| NM_000087 | CNGA1 | 0.11 |
| NM_015384 | IDN3 | 0.11 |
| NM_001183 | ATP6AP1 | 0.11 |
| NM_000775 | CYP2J2 | 0.11 |
| NM_002034 | FUT5 | 0.11 |
| NM_002260 | KLRC2 | 0.11 |
| NM_002784 | PSG9 | 0.11 |
| XM_376023 | ZBED4 | 0.11 |
| NM_006359 | SLC9A6 | 0.11 |
| NM_015100 | POGZ | 0.11 |
| NM_015393 | DKFZP564O0823 | 0.11 |
| NM_012436 | SPAG8 | 0.11 |
| NM_019070 | DDX49 | 0.11 |
| XM_371354 | FLJ10157 | 0.11 |
| NM_018719 | RAM2 | 0.11 |
| XM_087593 | KIAA1430 | 0.11 |
| NM_023015 | FLJ21919 | 0.11 |
| NM_024534 | FLJ12684 | 0.11 |
| NM_024061 | VIK | 0.11 |
| NM_032590 | FBXL10 | 0.11 |
| NM_032867 | FLJ14966 | 0.11 |
| NM_033257 | DGCR6L | 0.11 |
| NM_033418 | MGC9084 | 0.11 |
| NM_152433 | KBTBD3 | 0.11 |
| NM_172002 | HSC20 | 0.11 |
| NM_182518 | LOC149469 | 0.11 |
| NM_152602 | ZNF433 | 0.11 |
| XM_379334 | LOC257396 | 0.11 |
| XM_378430 | LOC283480 | 0.11 |
| NM_173832 | FLJ38705 | 0.11 |
| NM_178561 | MGC41943 | 0.11 |
| XM_292562 | LOC342460 | 0.11 |
| XM_211749 | LOC285047 | 0.11 |
| XM_068430 | LOC133609 | 0.11 |
| XM_379908 | LOC402579 | 0.11 |
| XM_374289 | LOC389702 | 0.11 |
| XM_373793 | LOC388504 | 0.11 |
| NM_001004063 | OR4K1 | 0.11 |
| NM_000298 | PKLR | 0.12 |
| NM_018979 | PRKWNK1 | 0.12 |
| NM_000291 | PGK1 | 0.12 |
| NM_001901 | CTGF | 0.12 |
| NM_001538 | HSF4 | 0.12 |
| NM_003181 | T | 0.12 |
| NM_006830 | UQCR | 0.12 |
| NM_004607 | TBCA | 0.12 |
| NM_058171 | ING2 | 0.11 |
| NM_004599 | SREBF2 | 0.11 |
| NM_005309 | GPT | 0.11 |
| NM_005323 | HIST1H1T | 0.11 |
| NM_001490 | GCNT1 | 0.11 |
| NM_019597 | HNRPH2 | 0.11 |
| NM_003133 | SRP9 | 0.11 |
| NM_006323 | SEC24B | 0.11 |
| NM_006857 | RY1 | 0.11 |
| NM_014925 | KIAA1002 | 0.11 |
| XM_166450 | BRPF3 | 0.11 |
| NM_199072 | HIC | 0.11 |
| NM_017704 | FGIF | 0.11 |
| NM_018328 | MBD5 | 0.11 |
| NM_020531 | C20ORF3 | 0.11 |
| XM_371664 | KIAA1257 | 0.11 |
| NM_024512 | LRRC2 | 0.11 |
| NM_024583 | SCRN3 | 0.11 |
| NM_030636 | KIAA1706 | 0.11 |
| NM_032334 | MGC14595 | 0.11 |
| NM_139279 | MCFD2 | 0.11 |
| NM_052888 | LOC114659 | 0.11 |
| NM_144772 | APOA1BP | 0.11 |
| NM_152507 | C21ORF128 | 0.11 |
| XM_086409 | KIAA2025 | 0.11 |
| NM_152597 | FSIP1 | 0.11 |
| NM_152782 | MGC33329 | 0.11 |
| NM_207341 | MGC87693 | 0.11 |
| NM_181608 | KRTAP19-2 | 0.11 |
| NM_198694 | KRTAP18-5 | 0.11 |
| NM_207460 | FLJ44313 | 0.11 |
| XM_098350 | LOC153297 | 0.11 |
| XM_117213 | LOC200292 | 0.11 |
| XM_372991 | LOC391540 | 0.11 |
| XM_374123 | LOC389295 | 0.11 |
| XM_373645 | LOC388169 | 0.11 |
| NM_001005234 | OR1L3 | 0.11 |
| NM_003607 | CDC42BPA | 0.12 |
| NM_005546 | ITK | 0.12 |
| NM_002760 | PRKY | 0.12 |
| NM_014879 | GPR105 | 0.12 |
| NM_005620 | S100A11 | 0.12 |
| NM_002501 | NFIX | 0.12 |
| NM_000918 | P4HB | 0.12 |
| NM_003645 | SLC27A2 | 0.12 |
| NM_001979 | EPHX2 | 0.12 |

| | | | | | |
|---|---|---|---|---|---|
| NM_020040 | TUBB4Q | 0.12 | NM_003300 | TRAF3 | 0.12 |
| NM_000867 | HTR2B | 0.12 | NM_001561 | TNFRSF9 | 0.12 |
| NM_014257 | CD209L | 0.12 | NM_006139 | CD28 | 0.12 |
| NM_004421 | DVL1 | 0.12 | NM_002995 | XCL1 | 0.12 |
| NM_000811 | GABRA6 | 0.12 | NM_000299 | PKP1 | 0.12 |
| AL022318 | APOBEC3D | 0.12 | NM_000253 | MTP | 0.12 |
| NM_006729 | DIAPH2 | 0.12 | NM_145246 | C10ORF4 | 0.12 |
| NM_003494 | DYSF | 0.12 | XM_370693 | POU6F1 | 0.12 |
| NM_014276 | RBPSUHL | 0.12 | NM_005840 | SPRY3 | 0.12 |
| NM_025220 | ADAM33 | 0.12 | NM_016615 | SLC6A13 | 0.12 |
| NM_001006607 | LOC474170 | 0.12 | NM_024498 | ZNF117 | 0.12 |
| NM_004782 | SNAP29 | 0.12 | NM_005447 | PAMCI | 0.12 |
| NM_014682 | ST18 | 0.12 | NM_006360 | GA17 | 0.12 |
| NM_006833 | COPS6 | 0.12 | XM_088315 | KIAA0870 | 0.12 |
| NM_014372 | RNF11 | 0.12 | NM_013322 | SNX10 | 0.12 |
| NM_016210 | LOC51161 | 0.12 | NM_013241 | FHOD1 | 0.12 |
| NM_018948 | MIG-6 | 0.12 | NM_017754 | C6ORF107 | 0.12 |
| NM_017903 | FLJ20618 | 0.12 | NM_017684 | VPS13C | 0.12 |
| NM_018172 | FLJ10661 | 0.12 | NM_019613 | LOC56270 | 0.12 |
| NM_022065 | THADA | 0.12 | NM_025000 | FLJ13096 | 0.12 |
| NM_024874 | PKD1-LIKE | 0.12 | NM_024887 | DHDDS | 0.12 |
| NM_031445 | MGC4268 | 0.12 | NM_032855 | HSH2D | 0.12 |
| XM_376018 | KIAA1644 | 0.12 | NM_173464 | L3MBTL4 | 0.12 |
| NM_138800 | TRIM43 | 0.12 | NM_080752 | ZSWIM3 | 0.12 |
| NM_173515 | MAGI1 | 0.12 | NM_173521 | C9ORF84 | 0.12 |
| NM_138295 | PKD1L1 | 0.12 | NM_173547 | LOC201292 | 0.12 |
| NM_173571 | LOC255313 | 0.12 | XM_048898 | HSPA12A | 0.12 |
| NM_178550 | MGC48998 | 0.12 | XM_292193 | DKFZP686J0811 | 0.12 |
| XM_294675 | LOC338667 | 0.12 | NM_182658 | C20ORF185 | 0.12 |
| NM_198539 | ZNF568 | 0.12 | NM_207458 | FLJ46026 | 0.12 |
| XM_060945 | LOC128360 | 0.12 | XM_088679 | LOC158812 | 0.12 |
| XM_061890 | LOC120146 | 0.12 | XM_089866 | LOC143506 | 0.12 |
| XM_372913 | LOC391347 | 0.12 | XM_374163 | LOC389372 | 0.12 |
| XM_374399 | LOC392630 | 0.12 | XM_373263 | LOC392262 | 0.12 |
| XM_378247 | LOC399806 | 0.12 | XM_372329 | LOC390007 | 0.12 |
| XM_373635 | LOC388139 | 0.12 | NM_001003693 | C6ORF21 | 0.12 |
| NM_001005482 | OR5H2 | 0.12 | NM_004717 | DGKI | 0.13 |
| NM_013330 | NME7 | 0.13 | NM_020126 | SPHK2 | 0.13 |
| NM_014476 | PDLIM3 | 0.13 | NM_004933 | CDH15 | 0.13 |
| NM_012147 | DUX2 | 0.13 | NM_004433 | ELF3 | 0.13 |
| NM_006454 | MXD4 | 0.13 | NM_005515 | HLXB9 | 0.13 |
| NM_000391 | CLN2 | 0.13 | NM_006412 | AGPAT2 | 0.13 |
| NM_003814 | ADAM20 | 0.13 | NM_002884 | RAP1A | 0.13 |
| NM_001990 | EYA3 | 0.13 | NM_016252 | BIRC6 | 0.13 |
| NM_001481 | GAS8 | 0.13 | NM_000962 | PTGS1 | 0.13 |
| NM_014479 | ADAMDEC1 | 0.13 | NM_004521 | KIF5B | 0.13 |

| | | | | | |
|---|---|---|---|---|---|
| NM_003856 | IL1RL1 | 0.13 | NM_002183 | IL3RA | 0.13 |
| NM_000809 | GABRA4 | 0.13 | NM_004557 | NOTCH4 | 0.13 |
| NM_133329 | KCNG3 | 0.13 | NM_003474 | ADAM12 | 0.13 |
| NM_017817 | RAB20 | 0.13 | NM_015417 | C20ORF28 | 0.13 |
| NM_003642 | HAT1 | 0.13 | NM_032832 | LRP11 | 0.13 |
| NM_015318 | ARHGEF18 | 0.13 | NM_003110 | SP2 | 0.13 |
| NM_006089 | SCML2 | 0.13 | NM_005277 | GPM6A | 0.13 |
| NM_002107 | H3F3A | 0.13 | NM_005512 | GARP | 0.13 |
| NM_018446 | AD-017 | 0.13 | NM_002813 | PSMD9 | 0.13 |
| NM_000582 | SPP1 | 0.13 | NM_003316 | TTC3 | 0.13 |
| NM_021968 | HIST1H4J | 0.13 | NM_019591 | ZNF26 | 0.13 |
| NM_015836 | WARS2 | 0.13 | XM_377355 | KIAA0602 | 0.13 |
| XM_031689 | MGA | 0.13 | NM_015144 | BDG29 | 0.13 |
| NM_012268 | PLD3 | 0.13 | NM_014579 | SLC39A2 | 0.13 |
| NM_013443 | SIAT7F | 0.13 | NM_016378 | VCX2 | 0.13 |
| NM_153203 | C21ORF74 | 0.13 | NM_017763 | FLJ20315 | 0.13 |
| NM_017917 | C14ORF10 | 0.13 | NM_018559 | KIAA1704 | 0.13 |
| NM_018400 | SCN3B | 0.13 | NM_031276 | TEX11 | 0.13 |
| NM_020873 | LRRN1 | 0.13 | NM_021824 | NIF3L1 | 0.13 |
| NM_020760 | HECW2 | 0.13 | NM_020764 | CASKIN1 | 0.13 |
| NM_133489 | SLC26A10 | 0.13 | NM_024726 | FLJ22527 | 0.13 |
| NM_030637 | DDHD1 | 0.13 | NM_030809 | C12ORF22 | 0.13 |
| NM_032018 | DKFZP547N043 | 0.13 | NM_032689 | ZNF607 | 0.13 |
| NM_032508 | FAM11A | 0.13 | NM_032898 | MGC14126 | 0.13 |
| NM_174887 | LOC90410 | 0.13 | NM_138434 | C7ORF29 | 0.13 |
| XM_043989 | LOC92270 | 0.13 | NM_145247 | C10ORF78 | 0.13 |
| XM_087804 | SYTL3 | 0.13 | NM_194283 | LOC134218 | 0.13 |
| NM_152436 | MGC39497 | 0.13 | XM_059384 | LOC129881 | 0.13 |
| NM_152516 | COMMD1 | 0.13 | XM_378876 | LOC149351 | 0.13 |
| NM_172037 | RDH10 | 0.13 | XM_376433 | SPINK5L3 | 0.13 |
| NM_153038 | FLJ32447 | 0.13 | NM_152684 | FLJ39653 | 0.13 |
| NM_145032 | FBXL13 | 0.13 | NM_174920 | LOC201191 | 0.13 |
| NM_152765 | MGC33510 | 0.13 | XM_208887 | LOC283871 | 0.13 |
| XM_378661 | LOC339263 | 0.13 | XM_293029 | CDKL4 | 0.13 |
| NM_138372 | LOC91661 | 0.13 | XM_375803 | LOC400785 | 0.13 |
| XM_371315 | LOC388701 | 0.13 | XM_373316 | LOC392391 | 0.13 |
| XM_378300 | LOC399920 | 0.13 | XM_378506 | LOC400350 | 0.13 |
| XM_373695 | LOC388300 | 0.13 | NM_001005275 | OR4A15 | 0.13 |
| NM_001715 | BLK | 0.14 | NM_174944 | C14ORF20 | 0.14 |
| NM_021158 | TRIB3 | 0.14 | NM_002821 | PTK7 | 0.14 |
| NM_000739 | CHRM2 | 0.14 | NM_005767 | P2RY5 | 0.14 |
| NM_018243 | 40787 | 0.14 | NM_176887 | TAS2R46 | 0.14 |
| NM_001942 | DSG1 | 0.14 | NM_001639 | APCS | 0.14 |
| NM_005346 | HSPA1B | 0.14 | NM_000596 | IGFBP1 | 0.14 |
| NM_000354 | SERPINA7 | 0.14 | NM_004083 | DDIT3 | 0.14 |
| NM_015995 | KLF13 | 0.14 | NM_005341 | HKR3 | 0.14 |

| | | | | | |
|---|---|---|---|---|---|
| NM_003106 | SOX2 | 0.14 | NM_006144 | GZMA | 0.14 |
| NM_003345 | UBE2I | 0.14 | NM_003385 | VSNL1 | 0.14 |
| NM_012400 | PLA2G2D | 0.14 | NM_018444 | PPM2C | 0.14 |
| XM_051093 | PPM1H | 0.14 | NM_002988 | CCL18 | 0.14 |
| NM_000732 | CD3D | 0.14 | NM_000760 | CSF3R | 0.14 |
| NM_000899 | KITLG | 0.14 | NM_006573 | TNFSF13B | 0.14 |
| NM_133263 | PPARGC1B | 0.14 | NM_000041 | APOE | 0.14 |
| NM_001045 | SLC6A4 | 0.14 | NM_001780 | CD63 | 0.14 |
| NM_016343 | CENPF | 0.14 | NM_000069 | CACNA1S | 0.14 |
| NM_022836 | DCLRE1B | 0.14 | NM_020958 | KIAA1622 | 0.14 |
| NM_004565 | PEX14 | 0.14 | NM_025151 | RCP | 0.14 |
| NR_001446 | ANXA2P3 | 0.14 | NM_004056 | CA8 | 0.14 |
| NM_002104 | GZMK | 0.14 | NM_004482 | GALNT3 | 0.14 |
| NM_001362 | DIO3 | 0.14 | NM_005389 | PCMT1 | 0.14 |
| NM_052957 | ACRC | 0.14 | NM_007149 | ZNF184 | 0.14 |
| NM_003518 | HIST1H2BG | 0.14 | NM_007126 | VCP | 0.14 |
| NM_003915 | CPNE1 | 0.14 | NM_003714 | STC2 | 0.14 |
| NM_020982 | CLDN9 | 0.14 | NM_005796 | NUTF2 | 0.14 |
| NM_006816 | LMAN2 | 0.14 | NM_007286 | SYNPO | 0.14 |
| NM_015110 | SMC5L1 | 0.14 | NM_014607 | UBXD2 | 0.14 |
| NM_014346 | C22ORF4 | 0.14 | NM_015342 | KIAA0073 | 0.14 |
| NM_030621 | DICER1 | 0.14 | XM_378078 | C9ORF4 | 0.14 |
| NM_015963 | THAP4 | 0.14 | NM_013399 | C16ORF5 | 0.14 |
| NM_015900 | PLA1A | 0.14 | NM_018982 | DJ167A19.1 | 0.14 |
| NM_016584 | IL23A | 0.14 | NM_017577 | DKFZP434C0328 | 0.14 |
| NM_017939 | FLJ20718 | 0.14 | NM_017678 | C11ORF33 | 0.14 |
| NM_017571 | LOC55580 | 0.14 | NM_020643 | C11ORF16 | 0.14 |
| XM_048721 | DKFZP762K222 | 0.14 | NM_020234 | MDS009 | 0.14 |
| NM_020119 | ZC3HAV1 | 0.14 | XM_029353 | KIAA1509 | 0.14 |
| NM_021203 | SRPRB | 0.14 | NM_022077 | MANBAL | 0.14 |
| NM_024918 | C20ORF172 | 0.14 | NM_030973 | ARC92 | 0.14 |
| NM_032259 | DKFZP434F054 | 0.14 | NM_032285 | MGC3207 | 0.14 |
| NM_032591 | SLC9A7 | 0.14 | NM_032598 | NYD-SP20 | 0.14 |
| XM_059730 | C6ORF159 | 0.14 | XM_376444 | LOC133491 | 0.14 |
| XM_380162 | LOC154822 | 0.14 | NM_152911 | PAOX | 0.14 |
| NM_181623 | KRTAP15-1 | 0.14 | XM_378356 | LOC283400 | 0.14 |
| NM_174942 | LOC283431 | 0.14 | XM_378969 | LOC284788 | 0.14 |
| NM_178516 | LOC283849 | 0.14 | NM_173648 | FLJ39502 | 0.14 |
| XM_379111 | LOC285033 | 0.14 | NM_175065 | HIST2H2AB | 0.14 |
| NM_182597 | FLJ39575 | 0.14 | NM_005110 | GFPT2 | 0.14 |
| XM_064062 | GPR139 | 0.14 | XM_060535 | LOC127545 | 0.14 |
| XM_211768 | LOC285110 | 0.14 | XM_087225 | LOC151507 | 0.14 |
| XM_379171 | LOC401048 | 0.14 | XM_377741 | LOC402072 | 0.14 |
| XM_380139 | LOC402587 | 0.14 | XM_376876 | LOC401531 | 0.14 |
| XM_379543 | LOC401442 | 0.14 | XM_372094 | LOC389730 | 0.14 |
| XM_372569 | LOC390577 | 0.14 | XM_375163 | LOC400320 | 0.14 |

| | | | | | |
|---|---|---|---|---|---|
| NM_001005326 | OR4F6 | 0.14 | NM_000604 | FGFR1 | 0.15 |
| NM_020421 | ADCK1 | 0.15 | NM_006259 | PRKG2 | 0.15 |
| NM_005873 | RGS19 | 0.15 | NM_005010 | NRCAM | 0.15 |
| NM_005013 | NUCB2 | 0.15 | NM_002580 | PAP | 0.15 |
| NM_014005 | PCDHA9 | 0.15 | NM_014012 | REM1 | 0.15 |
| NM_176822 | NALP14 | 0.15 | NM_002593 | PCOLCE | 0.15 |
| NM_001160 | APAF1 | 0.15 | NM_006286 | TFDP2 | 0.15 |
| NM_016371 | HSD17B7 | 0.15 | NM_002429 | MMP19 | 0.15 |
| NM_006690 | MMP24 | 0.15 | NM_138424 | KIF12 | 0.15 |
| NM_006845 | KIF2C | 0.15 | NM_003999 | OSMR | 0.15 |
| NM_002191 | INHA | 0.15 | NM_001430 | EPAS1 | 0.15 |
| NM_000268 | NF2 | 0.15 | NM_018209 | ARFGAP1 | 0.15 |
| NM_001764 | CD1B | 0.15 | NM_032830 | CIRH1A | 0.15 |
| NM_006400 | DCTN2 | 0.15 | NM_002004 | FDPS | 0.15 |
| NM_001442 | FABP4 | 0.15 | NM_006302 | GCS1 | 0.15 |
| NM_000283 | PDE6B | 0.15 | NM_014757 | MAML1 | 0.15 |
| NM_005039 | PRB1 | 0.15 | NM_014217 | KCNK2 | 0.15 |
| NM_000919 | PAM | 0.15 | NM_138726 | ABCC13 | 0.15 |
| NM_005615 | RNASE6 | 0.15 | NM_003276 | TMPO | 0.15 |
| NM_012074 | DPF3 | 0.15 | NM_012075 | C16ORF35 | 0.15 |
| NM_003462 | DNALI1 | 0.15 | NM_004921 | CLCA3 | 0.15 |
| NM_014659 | KIAA0377 | 0.15 | NM_007342 | NUPL2 | 0.15 |
| XM_375594 | ZNF507 | 0.15 | XM_048070 | ZNF292 | 0.15 |
| NM_015190 | DNAJC9 | 0.15 | NM_015679 | TRUB2 | 0.15 |
| NM_013300 | HSU79274 | 0.15 | NM_016953 | PDE11A | 0.15 |
| NM_015910 | LOC51057 | 0.15 | NM_018237 | CCAR1 | 0.15 |
| NM_020408 | C6ORF149 | 0.15 | XM_370756 | KIAA1305 | 0.15 |
| NM_178439 | GCL | 0.15 | NM_022135 | POPDC2 | 0.15 |
| NM_033030 | BOLL | 0.15 | NM_024086 | MGC3329 | 0.15 |
| NM_024576 | OGFRL1 | 0.15 | NM_024678 | FLJ23441 | 0.15 |
| NM_031435 | DKFZP564I0422 | 0.15 | NM_032300 | MGC10854 | 0.15 |
| XM_114418 | KIAA1729 | 0.15 | NM_080669 | MGC9564 | 0.15 |
| NM_152272 | MGC29816 | 0.15 | NM_152336 | FLJ32310 | 0.15 |
| NM_207314 | UNQ9373 | 0.15 | NM_173503 | FLJ25818 | 0.15 |
| NM_145054 | LOC146845 | 0.15 | NM_182829 | LOC158160 | 0.15 |
| NM_177455 | MIST1 | 0.15 | XM_376349 | LOC201725 | 0.15 |
| NM_152686 | MGC29463 | 0.15 | XM_378368 | LOC283392 | 0.15 |
| NM_173641 | EPHA10 | 0.15 | NM_182561 | FLJ36144 | 0.15 |
| NM_199350 | LOC375759 | 0.15 | NM_207501 | FLJ27255 | 0.15 |
| XM_292035 | LOC341392 | 0.15 | XM_373986 | LOC388952 | 0.15 |
| XM_379210 | LOC401085 | 0.15 | XM_373079 | LOC391769 | 0.15 |
| XM_371901 | LOC389490 | 0.15 | XM_380146 | LOC402596 | 0.15 |
| XM_370607 | LOC387750 | 0.15 | XM_377265 | LOC401720 | 0.15 |
| XM_377388 | LOC401819 | 0.15 | XM_373338 | LOC392425 | 0.15 |
| NM_001002761 | BPY2C | 0.15 | NM_004383 | CSK | 0.16 |
| NM_014550 | CARD10 | 0.16 | NM_139209 | GRK7 | 0.16 |

| | | | | | |
|---|---|---|---|---|---|
| NM_152230 | IMPK | 0.16 | NM_024594 | PANK3 | 0.16 |
| NM_014370 | STK23 | 0.16 | NM_005048 | PTHR2 | 0.16 |
| NM_002156 | HSPD1 | 0.16 | NM_002590 | PCDH8 | 0.16 |
| NM_002964 | S100A8 | 0.16 | NM_005249 | FOXG1B | 0.16 |
| NM_002449 | MSX2 | 0.16 | NM_005384 | NFIL3 | 0.16 |
| NM_006942 | SOX15 | 0.16 | NM_012476 | VAX2 | 0.16 |
| NM_000191 | HMGCL | 0.16 | NM_002686 | PNMT | 0.16 |
| NM_003257 | TJP1 | 0.16 | NM_033256 | PPP1R14A | 0.16 |
| NM_003219 | TERT | 0.16 | NM_002070 | GNAI2 | 0.16 |
| NM_006447 | USP16 | 0.16 | NM_006245 | PPP2R5D | 0.16 |
| NM_006613 | GRAP | 0.16 | NM_005854 | RAMP2 | 0.16 |
| NM_025216 | WNT10A | 0.16 | NM_000211 | ITGB2 | 0.16 |
| NM_002135 | NR4A1 | 0.16 | NM_020533 | MCOLN1 | 0.16 |
| NM_002644 | PIGR | 0.16 | Y10615 | ADAM3B | 0.16 |
| XM_170783 | LOC254571 | 0.16 | NM_000533 | PLP1 | 0.16 |
| NM_000876 | IGF2R | 0.16 | NM_001163 | APBA1 | 0.16 |
| NM_001925 | DEFA4 | 0.16 | NM_002519 | NPAT | 0.16 |
| NM_033226 | ABCC12 | 0.16 | XM_064152 | SRL | 0.16 |
| NM_021109 | TMSB4X | 0.16 | NM_007167 | ZNF258 | 0.16 |
| NM_006315 | RNF3 | 0.16 | XM_375697 | KIAA0459 | 0.16 |
| XM_370667 | KIAA1110 | 0.16 | NM_014611 | MDN1 | 0.16 |
| NM_032870 | C6ORF111 | 0.16 | NM_012330 | MYST4 | 0.16 |
| NM_014441 | SIGLEC9 | 0.16 | NM_014473 | HSA9761 | 0.16 |
| NM_020994 | CTAG2 | 0.16 | NM_015878 | OAZIN | 0.16 |
| NM_019075 | UGT1A10 | 0.16 | NM_019886 | CHST7 | 0.16 |
| NM_032546 | TRIM54 | 0.16 | NM_022052 | NXF3 | 0.16 |
| NM_020677 | HSCARG | 0.16 | NM_022744 | FLJ13868 | 0.16 |
| NM_022820 | CYP3A43 | 0.16 | NM_031481 | SLC25A18 | 0.16 |
| NM_032792 | ZNF499 | 0.16 | NM_052890 | PGLYRP2 | 0.16 |
| NM_145235 | FANK1 | 0.16 | XM_378828 | LOC115110 | 0.16 |
| NM_182501 | MGC61716 | 0.16 | NM_174983 | C19ORF28 | 0.16 |
| NM_152396 | MGC24132 | 0.16 | NM_175877 | MGC35023 | 0.16 |
| NM_152568 | FLJ25169 | 0.16 | NM_173529 | MGC33382 | 0.16 |
| XM_092342 | FLJ39061 | 0.16 | XM_114156 | LOC200213 | 0.16 |
| NM_173546 | MGC35097 | 0.16 | NM_199460 | LOC283439 | 0.16 |
| NM_173654 | MGC34132 | 0.16 | NM_199342 | LOC374969 | 0.16 |
| NM_182623 | FLJ36766 | 0.16 | NM_203347 | UNQ2541 | 0.16 |
| NM_207508 | FLJ45478 | 0.16 | NM_013440 | PILRB | 0.16 |
| NM_207453 | FLJ35934 | 0.16 | XM_060417 | LOC127295 | 0.16 |
| XM_291745 | LOC338611 | 0.16 | XM_373092 | LOC391807 | 0.16 |
| XM_374172 | LOC389403 | 0.16 | XM_374590 | LOC392850 | 0.16 |
| XM_374917 | LOC399937 | 0.16 | XM_373647 | LOC388171 | 0.16 |
| XM_378810 | LOC400706 | 0.16 | NM_133474 | KIAA1982 | 0.16 |
| NM_001893 | CSNK1D | 0.17 | NM_005316 | GTF2H1 | 0.17 |
| NM_006823 | PKIA | 0.17 | NM_021133 | RNASEL | 0.17 |
| NM_182691 | SRPK2 | 0.17 | NM_006281 | STK3 | 0.17 |

| | | | | | |
|---|---|---|---|---|---|
| NM_003553 | OR1E1 | 0.17 | NM_001051 | SSTR3 | 0.17 |
| NM_005284 | GPR6 | 0.17 | NM_005138 | SCO2 | 0.17 |
| NM_001078 | VCAM1 | 0.17 | NM_002718 | PPP2R3A | 0.17 |
| NM_024301 | FKRP | 0.17 | NM_022122 | MMP27 | 0.17 |
| NM_006811 | TDE1 | 0.17 | NM_016936 | UBN1 | 0.17 |
| NM_000463 | UGT1A1 | 0.17 | NM_006187 | OAS3 | 0.17 |
| NM_004901 | LYSAL1 | 0.17 | NM_006983 | MMP23A | 0.17 |
| NM_006292 | TSG101 | 0.17 | NM_001159 | AOX1 | 0.17 |
| NM_004423 | DVL3 | 0.17 | NM_006939 | SOS2 | 0.17 |
| NM_032557 | USP38 | 0.17 | NM_001783 | CD79A | 0.17 |
| NM_004798 | KIF3B | 0.17 | NM_054027 | ANKH | 0.17 |
| NM_015186 | VPS13A | 0.17 | NM_021097 | SLC8A1 | 0.17 |
| NM_014617 | CRYGA | 0.17 | NM_017700 | FLJ20184 | 0.17 |
| NM_001994 | F13B | 0.17 | NM_032242 | PLXNA1 | 0.17 |
| NM_012272 | HYPC | 0.17 | NM_001605 | AARS | 0.17 |
| NM_015285 | WDR7 | 0.17 | NM_002250 | KCNN4 | 0.17 |
| XM_039877 | MUC5B | 0.17 | NM_007105 | SLC22A1LS | 0.17 |
| NM_003445 | ZNF155 | 0.17 | XM_380170 | PDE4DIP | 0.17 |
| NM_003830 | SIGLEC5 | 0.17 | NM_005707 | PDCD7 | 0.17 |
| NM_021994 | ZNF277 | 0.17 | NM_007220 | CA5B | 0.17 |
| NM_015429 | TARSH | 0.17 | NM_014046 | MRPS18B | 0.17 |
| NM_014390 | SND1 | 0.17 | NM_016648 | HDCMA18P | 0.17 |
| NM_018927 | PCDHGB7 | 0.17 | XM_046600 | KIAA1272 | 0.17 |
| NM_022842 | CDCP1 | 0.17 | NM_024663 | NPEPL1 | 0.17 |
| NM_024783 | FLJ23598 | 0.17 | NM_024785 | FLJ22746 | 0.17 |
| NM_030573 | THAP7 | 0.17 | NM_025075 | NIF3L1BP1 | 0.17 |
| NM_031467 | SLC4A9 | 0.17 | NM_032353 | MGC10540 | 0.17 |
| NM_033082 | CIP29 | 0.17 | NM_052848 | MGC20255 | 0.17 |
| NM_033452 | TRIM47 | 0.17 | NM_182492 | DKFZP434O0213 | 0.17 |
| NM_173846 | C14ORF8 | 0.17 | NM_080746 | RPL10L | 0.17 |
| NM_024708 | ASB7 | 0.17 | XM_047734 | LOC146489 | 0.17 |
| NM_153291 | FAM10A5 | 0.17 | NM_152498 | FLJ32000 | 0.17 |
| NM_144963 | FLJ23790 | 0.17 | NM_145180 | C21ORF94 | 0.17 |
| XM_173036 | LOC255654 | 0.17 | NM_181718 | LOC253982 | 0.17 |
| XM_208778 | LOC283677 | 0.17 | NM_182564 | FLJ40319 | 0.17 |
| NM_181572 | RGSL1 | 0.17 | NM_198517 | FLJ00332 | 0.17 |
| XM_066752 | LOC139542 | 0.17 | XM_371755 | LOC401198 | 0.17 |
| XM_377943 | LOC402275 | 0.17 | XM_379515 | LOC401397 | 0.17 |
| XM_379517 | LOC401399 | 0.17 | XM_379619 | LOC401490 | 0.17 |
| XM_374637 | LOC392965 | 0.17 | XM_378228 | LOC399768 | 0.17 |
| XM_373519 | LOC387823 | 0.17 | XM_370729 | LOC387934 | 0.17 |
| XM_373789 | LOC388496 | 0.17 | XM_373359 | LOC392512 | 0.17 |
| XM_374343 | LOC389910 | 0.17 | NM_212556 | ASB18 | 0.17 |
| NM_138733 | PGK2 | 0.18 | NM_002089 | CXCL2 | 0.18 |
| NM_006727 | CDH10 | 0.18 | NM_020794 | KIAA1365 | 0.18 |
| NM_016374 | ARID4B | 0.18 | NM_020436 | SALL4 | 0.18 |

| | | | | | |
|---|---|---|---|---|---|
| NM_004405 | DLX2 | 0.18 | NM_005229 | ELK1 | 0.18 |
| NM_001775 | CD38 | 0.18 | NM_002800 | PSMB9 | 0.18 |
| NM_014235 | UBL4 | 0.18 | NM_004613 | TGM2 | 0.18 |
| NM_005045 | RELN | 0.18 | NM_002537 | OAZ2 | 0.18 |
| NM_000103 | CYP19A1 | 0.18 | NM_004871 | GOSR1 | 0.18 |
| NM_001790 | CDC25C | 0.18 | NM_003894 | PER2 | 0.18 |
| NM_005693 | NR1H3 | 0.18 | NM_004980 | KCND3 | 0.18 |
| NM_020675 | SPC25 | 0.18 | NM_005174 | ATP5C1 | 0.18 |
| NM_139286 | CDC26 | 0.18 | NM_001264 | CDSN | 0.18 |
| NM_002094 | GSPT1 | 0.18 | NM_016133 | INSIG2 | 0.18 |
| XM_375550 | SHC2 | 0.18 | NM_006607 | PTTG2 | 0.18 |
| NM_001816 | CEACAM8 | 0.18 | NM_006234 | POLR2J | 0.18 |
| XM_291723 | AKR1CL1 | 0.18 | NM_006750 | SNTB2 | 0.18 |
| NM_003155 | STC1 | 0.18 | NM_003715 | VDP | 0.18 |
| NM_006036 | KIAA0436 | 0.18 | NM_014639 | KIAA0372 | 0.18 |
| NM_006791 | MORF4L1 | 0.18 | NM_031456 | FBXW10 | 0.18 |
| NM_015503 | SH2B | 0.18 | NM_015584 | POLDIP2 | 0.18 |
| NM_014008 | JM1 | 0.18 | NM_014415 | ZBTB11 | 0.18 |
| NM_181836 | CGI-109 | 0.18 | NM_015946 | PELO | 0.18 |
| NM_019000 | FLJ20152 | 0.18 | NM_031274 | TEX13A | 0.18 |
| NM_020201 | NT5M | 0.18 | NM_020235 | BBX | 0.18 |
| NM_022058 | SLC4A10 | 0.18 | NM_020536 | CSRP2BP | 0.18 |
| NM_022659 | EBF2 | 0.18 | NM_031231 | APBA2BP | 0.18 |
| NM_023944 | CYP4F12 | 0.18 | NM_024336 | IRX3 | 0.18 |
| NM_153182 | MINA | 0.18 | NM_032368 | LZIC | 0.18 |
| NM_032735 | MGC13168 | 0.18 | NM_080664 | C14ORF126 | 0.18 |
| NM_033409 | C20ORF54 | 0.18 | NM_052913 | KIAA1913 | 0.18 |
| NM_144573 | NEXN | 0.18 | XM_062690 | ASCL4 | 0.18 |
| NM_144627 | SSTK-IP | 0.18 | NM_139172 | MDAC1 | 0.18 |
| NM_152523 | FLJ40432 | 0.18 | XM_168053 | C6ORF184 | 0.18 |
| XM_371057 | LOC201175 | 0.18 | NM_152914 | MGC33894 | 0.18 |
| XM_211736 | LOC285016 | 0.18 | XM_211345 | LOC284134 | 0.18 |
| XM_171892 | LOC253013 | 0.18 | XM_377778 | LOC402110 | 0.18 |
| XM_374046 | LOC389142 | 0.18 | XM_373852 | LOC388648 | 0.18 |
| XM_376532 | LOC401272 | 0.18 | XM_374175 | LOC389413 | 0.18 |
| XM_373214 | LOC392145 | 0.18 | XM_380140 | LOC402589 | 0.18 |
| XM_372423 | LOC390231 | 0.18 | XM_370837 | LOC388080 | 0.18 |
| XM_375575 | LOC400677 | 0.18 | XM_374323 | LOC389841 | 0.18 |
| NM_003688 | CASK | 0.19 | NM_020639 | ANKRD3 | 0.19 |
| XM_047355 | KIAA1765 | 0.19 | NM_018267 | H2AFJ | 0.19 |
| NM_006399 | BATF | 0.19 | NM_001888 | CRYM | 0.19 |
| NM_001909 | CTSD | 0.19 | NM_019094 | NUDT4 | 0.19 |
| NM_016237 | ANAPC5 | 0.19 | NM_004985 | KRAS2 | 0.19 |
| NM_017703 | FBXL12 | 0.19 | NM_012326 | MAPRE3 | 0.19 |
| NM_002470 | MYH3 | 0.19 | NM_006900 | IFNA13 | 0.19 |
| NM_005092 | TNFSF18 | 0.19 | NM_006687 | ACTL7A | 0.19 |

| | | |
|---|---|---|
| NM_022839 | MRPS11 | 0.19 |
| NM_014461 | CNTN6 | 0.19 |
| NM_153443 | KIR3DL3 | 0.19 |
| NR_001569 | ADAM3A | 0.19 |
| NM_005662 | VDAC3 | 0.19 |
| NM_003732 | EIF4EBP3 | 0.19 |
| NM_015127 | MCLC | 0.19 |
| NM_014612 | C9ORF10 | 0.19 |
| NM_015553 | PIP3-E | 0.19 |
| NM_022720 | DGCR8 | 0.19 |
| NM_018201 | TBC1D13 | 0.19 |
| NM_018022 | FLJ10199 | 0.19 |
| NM_017769 | KIAA1333 | 0.19 |
| NM_032579 | RETNLB | 0.19 |
| NM_032338 | MGC14817 | 0.19 |
| NM_138786 | LOC116441 | 0.19 |
| NM_153208 | MGC35048 | 0.19 |
| NM_213598 | ZNF543 | 0.19 |
| NM_175873 | LOC134548 | 0.19 |
| NM_181534 | KRT25A | 0.19 |
| NM_182521 | ZSWIM2 | 0.19 |
| NM_152591 | FLJ35843 | 0.19 |
| NM_194247 | HNRNPA3 | 0.19 |
| NM_182590 | FLJ33651 | 0.19 |
| NM_206831 | DESR1 | 0.19 |
| NM_199000 | LHFPL3 | 0.19 |
| XM_058931 | LOC125704 | 0.19 |
| XM_372764 | LOC391004 | 0.19 |
| XM_372047 | LOC389668 | 0.19 |
| XM_380045 | LOC402696 | 0.19 |
| XM_374902 | LOC399917 | 0.19 |
| XM_372695 | LOC390859 | 0.19 |
| XM_372274 | LOC389907 | 0.19 |
| NM_014183 | DNCL2A | 0.2 |
| NM_015477 | SIN3A | 0.2 |
| NM_005225 | E2F1 | 0.2 |
| NM_006492 | ALX3 | 0.2 |
| NM_016142 | HSD17B12 | 0.2 |
| XM_370630 | PPP1R14B | 0.2 |
| NM_003449 | TRIM26 | 0.2 |
| NM_004513 | IL16 | 0.2 |
| NM_000834 | GRIN2B | 0.2 |
| NM_000427 | LOR | 0.2 |
| NM_006922 | SCN3A | 0.2 |
| NM_005722 | ACTR2 | 0.2 |
| AK056774 | H19 | 0.2 |
| NM_004982 | KCNJ8 | 0.19 |
| NM_014417 | BBC3 | 0.19 |
| NM_002567 | PBP | 0.19 |
| NM_002925 | RGS10 | 0.19 |
| NM_003585 | DOC2B | 0.19 |
| NM_014700 | RAB11-FIP3 | 0.19 |
| NM_014939 | KIAA1012 | 0.19 |
| NM_015476 | C18ORF10 | 0.19 |
| NM_021093 | PYY2 | 0.19 |
| NM_016395 | HSPC121 | 0.19 |
| NM_017661 | SUHW4 | 0.19 |
| NM_018356 | FLJ11193 | 0.19 |
| NM_030798 | WBSCR16 | 0.19 |
| NM_032816 | FLJ14640 | 0.19 |
| NM_024586 | OSBPL9 | 0.19 |
| NM_053050 | MRPL53 | 0.19 |
| XM_371010 | MGC49942 | 0.19 |
| NM_152378 | FLJ31052 | 0.19 |
| NM_153339 | FLJ90811 | 0.19 |
| NM_152475 | MGC34079 | 0.19 |
| NM_173851 | SLC30A8 | 0.19 |
| NM_152635 | OIT3 | 0.19 |
| NM_173638 | MGC8902 | 0.19 |
| XM_376238 | LOC285331 | 0.19 |
| NM_198557 | FLJ45645 | 0.19 |
| NM_001004127 | LOC440138 | 0.19 |
| XM_060054 | LOC139793 | 0.19 |
| XM_380150 | LOC402603 | 0.19 |
| XM_379809 | LOC402481 | 0.19 |
| XM_373500 | LOC387771 | 0.19 |
| XM_373780 | LOC388481 | 0.19 |
| XM_378988 | LOC400849 | 0.19 |
| NM_002739 | PRKCG | 0.2 |
| NM_004459 | FALZ | 0.2 |
| NM_003084 | SNAPC3 | 0.2 |
| NM_001964 | EGR1 | 0.2 |
| NM_013351 | TBX21 | 0.2 |
| NM_014265 | ADAM28 | 0.2 |
| NM_006699 | MAN1A2 | 0.2 |
| NM_001718 | BMP6 | 0.2 |
| NM_002341 | LTB | 0.2 |
| NM_057088 | KRT3 | 0.2 |
| NM_003661 | APOL1 | 0.2 |
| NM_004974 | KCNA2 | 0.2 |
| NM_031942 | CDCA7 | 0.2 |
| NM_013374 | PDCD6IP | 0.2 |

| | | | | | |
|---|---|---|---|---|---|
| NM_152309 | PIK3AP1 | 0.2 | NM_005842 | SPRY2 | 0.2 |
| NM_005637 | SS18 | 0.2 | NM_005968 | HNRPM | 0.2 |
| NM_002364 | MAGEB2 | 0.2 | NM_004594 | SLC9A5 | 0.2 |
| XM_352937 | TBX15 | 0.2 | XM_032693 | KIAA0420 | 0.2 |
| NM_016940 | C21ORF6 | 0.2 | XM_375045 | C14ORF92 | 0.2 |
| NM_006426 | DPYSL4 | 0.2 | NM_015481 | ZNF385 | 0.2 |
| NM_016018 | CGI-72 | 0.2 | NM_015914 | LOC51061 | 0.2 |
| NM_019026 | LOC54499 | 0.2 | NM_016024 | RBMX2 | 0.2 |
| NM_017708 | FLJ20200 | 0.2 | NM_018352 | FLJ11184 | 0.2 |
| XM_290842 | LRFN1 | 0.2 | NM_021196 | SLC4A5 | 0.2 |
| NM_031420 | MRPL9 | 0.2 | NM_022129 | MAWBP | 0.2 |
| NM_024307 | MGC4171 | 0.2 | NM_030818 | MGC10471 | 0.2 |
| NM_032806 | FLJ14566 | 0.2 | NM_032524 | KRTAP4-4 | 0.2 |
| NM_033121 | ANKRD13 | 0.2 | NM_144584 | FLJ30525 | 0.2 |
| NM_052944 | SLC5A11 | 0.2 | NM_138453 | RAB3C | 0.2 |
| NM_145691 | ATPAF2 | 0.2 | NM_173475 | MGC48972 | 0.2 |
| NM_144591 | C10ORF32 | 0.2 | NM_144694 | ZNF570 | 0.2 |
| NM_153709 | MGC40168 | 0.2 | NM_153041 | FLJ32955 | 0.2 |
| XM_097580 | LOC149086 | 0.2 | NM_144962 | MGC22776 | 0.2 |
| XM_089747 | C10ORF80 | 0.2 | XM_378226 | C10ORF128 | 0.2 |
| NM_152612 | FLJ36046 | 0.2 | NM_153367 | C10ORF56 | 0.2 |
| NM_178836 | LOC201164 | 0.2 | NM_152726 | FLJ34588 | 0.2 |
| NM_152787 | TAB3 | 0.2 | NM_198278 | LOC255743 | 0.2 |
| NM_174962 | SSX9 | 0.2 | XM_378687 | LOC339210 | 0.2 |
| XM_290835 | ZNF181 | 0.2 | XM_290579 | LOC338797 | 0.2 |
| NM_182611 | GPR144 | 0.2 | NM_198519 | MGC12965 | 0.2 |
| XM_088578 | LOC375748 | 0.2 | NM_004193 | GBF1 | 0.2 |
| NM_138397 | LOC93082 | 0.2 | XM_291544 | LOC343384 | 0.2 |
| XM_208809 | LOC283726 | 0.2 | XM_209041 | LOC284158 | 0.2 |
| XM_096642 | LOC144631 | 0.2 | XM_371244 | LOC388624 | 0.2 |
| XM_379135 | LOC401005 | 0.2 | XM_379381 | LOC401225 | 0.2 |
| XM_371690 | LOC389202 | 0.2 | XM_374949 | LOC399979 | 0.2 |
| NM_001004345 | FLJ16124 | 0.2 | NM_005204 | MAP3K8 | 0.21 |
| NM_005884 | PAK4 | 0.21 | NM_002757 | MAP2K5 | 0.21 |
| NM_013939 | OR10H2 | 0.21 | NM_016568 | SALPR | 0.21 |
| NM_032551 | GPR54 | 0.21 | NM_003389 | CORO2A | 0.21 |
| NM_001232 | CASQ2 | 0.21 | NM_019071 | ING3 | 0.21 |
| NM_002471 | MYH6 | 0.21 | NM_004343 | CALR | 0.21 |
| NM_001198 | PRDM1 | 0.21 | NM_002229 | JUNB | 0.21 |
| NM_014269 | ADAM29 | 0.21 | NM_000263 | NAGLU | 0.21 |
| NM_024831 | NCOA6IP | 0.21 | NM_025003 | ADAMTS20 | 0.21 |
| XM_088476 | TXNDC4 | 0.21 | NM_000377 | WAS | 0.21 |
| NM_015601 | HERC4 | 0.21 | NM_012308 | FBXL11 | 0.21 |
| NM_000577 | IL1RN | 0.21 | NM_001146 | ANGPT1 | 0.21 |
| NM_002965 | S100A9 | 0.21 | NM_019101 | APOM | 0.21 |
| NM_002385 | MBP | 0.21 | NM_005436 | CCDC6 | 0.21 |

| | | | | | |
|---|---|---|---|---|---|
| NM_024744 | ALS2CR8 | 0.21 | NM_005075 | SLCO1A2 | 0.21 |
| NM_003478 | CUL5 | 0.21 | NM_003949 | HAP1 | 0.21 |
| NM_014641 | MDC1 | 0.21 | NM_145039 | MGC16385 | 0.21 |
| NM_002087 | GRN | 0.21 | NM_001384 | DPH2L2 | 0.21 |
| XM_031246 | ROBO2 | 0.21 | NM_005425 | TNP2 | 0.21 |
| NM_005082 | TRIM25 | 0.21 | NM_174910 | TCTE3 | 0.21 |
| NM_004839 | HOMER2 | 0.21 | NM_021064 | HIST1H2AG | 0.21 |
| XM_375032 | TBC1D4 | 0.21 | NM_021144 | PSIP1 | 0.21 |
| NM_007047 | BTN3A2 | 0.21 | NM_014954 | RPH3A | 0.21 |
| NM_015510 | DKFZP566O084 | 0.21 | NM_016590 | PART1 | 0.21 |
| NM_019040 | ELP4 | 0.21 | NM_023003 | TM6SF1 | 0.21 |
| NM_016200 | LSM8 | 0.21 | NM_017540 | GALNT10 | 0.21 |
| NM_020145 | SH3GLB2 | 0.21 | NM_021940 | SMAP1 | 0.21 |
| NM_032461 | SPANXB1 | 0.21 | NM_030899 | ZNF323 | 0.21 |
| NM_024511 | C4ORF15 | 0.21 | NM_024584 | FLJ13646 | 0.21 |
| NM_025031 | FLJ21075 | 0.21 | NM_203447 | DOCK8 | 0.21 |
| NM_032269 | DKFZP434I099 | 0.21 | NM_031289 | GSG1 | 0.21 |
| NM_032354 | MGC10744 | 0.21 | NM_032856 | FLJ14888 | 0.21 |
| NM_138771 | LOC90693 | 0.21 | XM_033370 | ZFHX2 | 0.21 |
| NM_080866 | SLC22A9 | 0.21 | NM_054024 | MIA2 | 0.21 |
| NM_152321 | FLJ32115 | 0.21 | NM_144577 | FLJ32926 | 0.21 |
| NM_058219 | EXOSC6 | 0.21 | NM_152389 | MGC35338 | 0.21 |
| NM_152395 | FLJ31265 | 0.21 | NM_144622 | FLJ32934 | 0.21 |
| NM_152440 | FLJ32549 | 0.21 | XM_167147 | ZNF390 | 0.21 |
| NM_152777 | C14ORF48 | 0.21 | NM_173647 | RNF149 | 0.21 |
| NM_173671 | FLJ37396 | 0.21 | NM_173672 | PPIL6 | 0.21 |
| NM_181807 | DCDC1 | 0.21 | NM_182608 | DKFZP686O1689 | 0.21 |
| NM_178353 | LCE1E | 0.21 | NM_198538 | UNQ698 | 0.21 |
| NM_207441 | FLJ42220 | 0.21 | XM_117239 | LOC200491 | 0.21 |
| XM_086725 | LOC149934 | 0.21 | XM_291757 | LOC340893 | 0.21 |
| XM_291885 | LOC341112 | 0.21 | XM_378874 | LOC400761 | 0.21 |
| XM_371783 | LOC389347 | 0.21 | XM_374192 | LOC389447 | 0.21 |
| XM_376486 | LOC401250 | 0.21 | XM_380136 | LOC402582 | 0.21 |
| XM_380151 | LOC402617 | 0.21 | XM_372078 | LOC389710 | 0.21 |
| XM_379643 | LOC401530 | 0.21 | XM_378223 | LOC399753 | 0.21 |
| XM_372305 | LOC389941 | 0.21 | XM_371053 | LOC388381 | 0.21 |
| XM_352159 | LOC388215 | 0.21 | NM_001004332 | FLJ41170 | 0.21 |
| XM_372254 | LOC389892 | 0.21 | NM_017988 | FLJ10074 | 0.22 |
| NM_002577 | PAK2 | 0.22 | NM_006258 | PRKG1 | 0.22 |
| NM_005298 | GPR25 | 0.22 | NM_001881 | CREM | 0.22 |
| NM_004492 | GTF2A2 | 0.22 | NM_020781 | ZNF398 | 0.22 |
| NM_004040 | RHOB | 0.22 | NM_000405 | GM2A | 0.22 |
| NM_002096 | GTF2F1 | 0.22 | NM_003733 | OASL | 0.22 |
| NM_007202 | AKAP10 | 0.22 | NM_004158 | PSPN | 0.22 |
| NM_000138 | FBN1 | 0.22 | NM_005481 | THRAP5 | 0.22 |
| NM_005268 | GJB5 | 0.22 | NM_006538 | BCL2L11 | 0.22 |

| | | |
|---|---|---|
| NM_005199 | CHRNG | 0.22 |
| NM_002114 | HIVEP1 | 0.22 |
| NM_002907 | RECQL | 0.22 |
| NM_002338 | LSAMP | 0.22 |
| NM_145698 | ACBD5 | 0.22 |
| XM_168302 | ZNF36 | 0.22 |
| NM_005832 | KCNMB2 | 0.22 |
| NM_005862 | STAG1 | 0.22 |
| NM_012255 | XRN2 | 0.22 |
| NM_014282 | HABP4 | 0.22 |
| NM_015649 | IRF2BP1 | 0.22 |
| NM_130459 | TOR2A | 0.22 |
| NM_019048 | NS3TP1 | 0.22 |
| NM_018366 | CNO | 0.22 |
| NM_018309 | FLJ11046 | 0.22 |
| NM_020398 | SPINLW1 | 0.22 |
| XM_370928 | KIAA1171 | 0.22 |
| XM_050644 | KIAA1623 | 0.22 |
| NM_022353 | OSGEPL1 | 0.22 |
| NM_024753 | FLJ11457 | 0.22 |
| NM_024806 | FLJ23554 | 0.22 |
| NM_032574 | LOC84661 | 0.22 |
| NM_032336 | MGC14799 | 0.22 |
| NM_032858 | FLJ14904 | 0.22 |
| NM_173468 | MOBKL1A | 0.22 |
| NM_201614 | IKIP | 0.22 |
| NM_194318 | B3GTL | 0.22 |
| NM_170610 | HIST1H2BA | 0.22 |
| NM_175900 | FLJ35681 | 0.22 |
| NM_198507 | UNQ1912 | 0.22 |
| NM_178012 | MGC8685 | 0.22 |
| XM_209569 | LOC285329 | 0.22 |
| XM_376074 | LOC400968 | 0.22 |
| XM_372311 | LOC389958 | 0.22 |
| XM_370905 | LOC388174 | 0.22 |
| NM_001001346 | CLDN20 | 0.22 |
| XM_377072 | LOC401623 | 0.22 |
| NM_001465 | FYB | 0.23 |
| NM_003029 | SHC1 | 0.23 |
| NM_014626 | GPR58 | 0.23 |
| NM_005982 | SIX1 | 0.23 |
| NM_001333 | CTSL2 | 0.23 |
| NM_002769 | PRSS1 | 0.23 |
| NM_016529 | ATP8A2 | 0.23 |
| NM_032622 | LNX | 0.23 |
| NM_001559 | IL12RB2 | 0.23 |
| NM_004446 | EPRS | 0.22 |
| NM_006851 | GLIPR1 | 0.22 |
| NM_004828 | NCR2 | 0.22 |
| NM_032432 | ABLIM2 | 0.22 |
| NM_013362 | ZNF225 | 0.22 |
| NM_014779 | KIAA0669 | 0.22 |
| XM_375848 | KIAA0792 | 0.22 |
| NM_006455 | SC65 | 0.22 |
| NM_015210 | KIAA0802 | 0.22 |
| NM_012456 | TIMM10 | 0.22 |
| NM_007052 | NOX1 | 0.22 |
| NM_016129 | COPS4 | 0.22 |
| NM_017905 | C13ORF11 | 0.22 |
| NM_018247 | TMEM30A | 0.22 |
| NM_020362 | HT014 | 0.22 |
| NM_199483 | C20ORF24 | 0.22 |
| XM_371576 | KIAA1189 | 0.22 |
| XM_370682 | KIAA1467 | 0.22 |
| NM_022918 | FLJ22104 | 0.22 |
| NM_024672 | THAP9 | 0.22 |
| NM_024911 | FLJ23091 | 0.22 |
| NM_032328 | MGC12458 | 0.22 |
| NM_032349 | SDOS | 0.22 |
| NM_139240 | LOC92346 | 0.22 |
| XM_058743 | LOC123876 | 0.22 |
| NM_138803 | LOC130940 | 0.22 |
| NM_152465 | MGC39650 | 0.22 |
| NM_198827 | GPR133 | 0.22 |
| XM_375446 | FLJ11822 | 0.22 |
| NM_207373 | C10ORF99 | 0.22 |
| NM_207419 | C1QTNF8 | 0.22 |
| XM_371313 | LOC388698 | 0.22 |
| XM_376048 | LOC400949 | 0.22 |
| XM_370758 | LOC387978 | 0.22 |
| XM_373692 | LOC388291 | 0.22 |
| XM_373945 | LOC388849 | 0.22 |
| NM_214461 | MGC50273 | 0.22 |
| NM_000115 | EDNRB | 0.23 |
| NM_005613 | RGS4 | 0.23 |
| NM_001235 | SERPINH1 | 0.23 |
| NM_003200 | TCF3 | 0.23 |
| NM_006229 | PNLIPRP1 | 0.23 |
| NM_014382 | ATP2C1 | 0.23 |
| NM_004620 | TRAF6 | 0.23 |
| NM_004577 | PSPH | 0.23 |
| NM_014211 | GABRP | 0.23 |

| | | | | | |
|---|---|---|---|---|---|
| NM_015102 | NPHP4 | 0.23 | NM_019604 | CRTAM | 0.23 |
| NM_033183 | CGB8 | 0.23 | NM_006726 | LRBA | 0.23 |
| NM_006986 | MAGED1 | 0.23 | NM_004077 | CS | 0.23 |
| NM_000779 | CYP4B1 | 0.23 | NM_000782 | CYP24A1 | 0.23 |
| NM_001404 | EEF1G | 0.23 | XM_495860 | MUC5AC | 0.23 |
| NM_031887 | PMCHL1 | 0.23 | NM_033087 | ALG2 | 0.23 |
| NM_002998 | SDC2 | 0.23 | NM_003008 | SEMG2 | 0.23 |
| NM_006762 | LAPTM5 | 0.23 | NM_145330 | MRPL33 | 0.23 |
| NM_004786 | TXNL1 | 0.23 | NM_004800 | TM9SF2 | 0.23 |
| NM_014642 | IQCB1 | 0.23 | NM_014908 | TMEM15 | 0.23 |
| NM_012405 | ICMT | 0.23 | NM_014187 | HSPC171 | 0.23 |
| NM_016479 | SCOTIN | 0.23 | NM_013379 | DPP7 | 0.23 |
| NM_019007 | FLJ20811 | 0.23 | NM_018407 | LAPTM4B | 0.23 |
| NM_017816 | LYAR | 0.23 | NM_018590 | GALNACT-2 | 0.23 |
| NM_030567 | MGC10772 | 0.23 | NM_025227 | BPIL1 | 0.23 |
| NM_033260 | FOXQ1 | 0.23 | XM_371838 | USP45 | 0.23 |
| NM_181705 | LOC90624 | 0.23 | NM_152365 | FLJ34633 | 0.23 |
| NM_080868 | ASB17 | 0.23 | NM_152415 | FLJ32642 | 0.23 |
| NM_144660 | SAMD8 | 0.23 | NM_144659 | TCP10L | 0.23 |
| NM_173516 | PNLDC1 | 0.23 | NM_144716 | MGC23918 | 0.23 |
| NM_173557 | RNF152 | 0.23 | NM_153274 | VMD2L2 | 0.23 |
| NM_173618 | FLJ90652 | 0.23 | NM_173665 | MGC34713 | 0.23 |
| XM_209490 | LOC285148 | 0.23 | NM_173637 | MGC34725 | 0.23 |
| AK091675 | LOC286087 | 0.23 | XM_113916 | LOC201181 | 0.23 |
| XM_211983 | LOC285694 | 0.23 | XM_167254 | LOC221875 | 0.23 |
| XM_374091 | LOC389233 | 0.23 | XM_374188 | LOC389440 | 0.23 |
| XM_372111 | LOC389752 | 0.23 | XM_379547 | LOC401445 | 0.23 |
| XM_374898 | LOC399909 | 0.23 | XM_373341 | LOC392439 | 0.23 |
| NM_001319 | CSNK1G2 | 0.24 | NM_024652 | LRRK1 | 0.24 |
| NM_001744 | CAMK4 | 0.24 | NM_004157 | PRKAR2A | 0.24 |
| NM_002447 | MST1R | 0.24 | NM_007197 | FZD10 | 0.24 |
| NM_005308 | GRK5 | 0.24 | NM_148962 | OXER1 | 0.24 |
| NM_001265 | CDX2 | 0.24 | NM_002478 | MYOD1 | 0.24 |
| NM_005643 | TAF11 | 0.24 | NM_001685 | ATP5J | 0.24 |
| NM_000270 | NP | 0.24 | NM_003260 | TLE2 | 0.24 |
| NM_000390 | CHM | 0.24 | NM_015089 | PARC | 0.24 |
| NM_003958 | RNF8 | 0.24 | NM_003031 | SIAH1 | 0.24 |
| XM_290527 | USP35 | 0.24 | NM_014053 | FLVCR | 0.24 |
| NM_181449 | IREM2 | 0.24 | NM_006419 | CXCL13 | 0.24 |
| NM_002608 | PDGFB | 0.24 | NM_005556 | KRT7 | 0.24 |
| NM_004048 | B2M | 0.24 | NM_004354 | CCNG2 | 0.24 |
| NM_018602 | DNAJA4 | 0.24 | NM_018649 | H2AFY2 | 0.24 |
| NM_152468 | EVER2 | 0.24 | XM_376683 | LCHN | 0.24 |
| NM_005534 | IFNGR2 | 0.24 | NM_004099 | STOM | 0.24 |
| XM_166300 | AIM1 | 0.24 | NM_001245 | SIGLEC6 | 0.24 |
| NM_031498 | GNGT2 | 0.24 | NM_023037 | 13CDNA73 | 0.24 |

| | | | | | |
|---|---|---|---|---|---|
| NM_012328 | DNAJB9 | 0.24 | NM_006671 | SLC1A7 | 0.24 |
| NM_007212 | RNF2 | 0.24 | NM_003099 | SNX1 | 0.24 |
| NM_020634 | GDF3 | 0.24 | NM_006702 | NTE | 0.24 |
| XM_037523 | KIAA1076 | 0.24 | NM_015061 | JMJD2C | 0.24 |
| NM_012319 | SLC39A6 | 0.24 | NM_015535 | DNAPTP6 | 0.24 |
| NM_014066 | COMMD5 | 0.24 | NM_016417 | C14ORF87 | 0.24 |
| NM_015982 | YBX2 | 0.24 | NM_013397 | C6ORF49 | 0.24 |
| NM_018215 | FLJ10781 | 0.24 | XM_370618 | FLJ20294 | 0.24 |
| XM_378914 | KIAA0492 | 0.24 | NM_024324 | MGC11256 | 0.24 |
| NM_025221 | KCNIP4 | 0.24 | NM_031442 | TM4SF10 | 0.24 |
| NM_032323 | MGC13102 | 0.24 | NM_032658 | MGC10701 | 0.24 |
| XM_055636 | KIAA1912 | 0.24 | NM_138463 | LOC116238 | 0.24 |
| NM_152479 | MGC33962 | 0.24 | NM_178868 | CKLFSF8 | 0.24 |
| NM_182828 | GDF7 | 0.24 | NM_173522 | FLJ36576 | 0.24 |
| XM_116497 | C6ORF163 | 0.24 | NM_152780 | FLJ14503 | 0.24 |
| XM_172968 | LOC253962 | 0.24 | NM_173642 | MGC47816 | 0.24 |
| XM_370699 | PTPRQ | 0.24 | NM_182628 | FLJ40083 | 0.24 |
| XM_291396 | LOC400735 | 0.24 | XM_379454 | LOC401284 | 0.24 |
| XM_377828 | LOC402160 | 0.24 | XM_379622 | LOC401491 | 0.24 |
| XM_373575 | LOC387946 | 0.24 | XM_370586 | LOC387723 | 0.24 |
| NM_004080 | DGKB | 0.25 | NM_001261 | CDK9 | 0.25 |
| NM_005906 | MAK | 0.25 | NM_005056 | JARID1A | 0.25 |
| NM_001701 | BAAT | 0.25 | NM_005700 | DPP3 | 0.25 |
| NM_012343 | NNT | 0.25 | NM_016272 | TOB2 | 0.25 |
| NM_021103 | TMSB10 | 0.25 | NM_004104 | FASN | 0.25 |
| NM_014861 | KIAA0703 | 0.25 | NM_006910 | RBBP6 | 0.25 |
| NM_007331 | WHSC1 | 0.25 | NM_002339 | LSP1 | 0.25 |
| NM_021077 | NMB | 0.25 | NM_000799 | EPO | 0.25 |
| NM_005720 | ARPC1B | 0.25 | NM_003356 | UCP3 | 0.25 |
| NM_004086 | COCH | 0.25 | NM_001196 | BID | 0.25 |
| NM_015675 | GADD45B | 0.25 | NM_000507 | FBP1 | 0.25 |
| X74144 | FOXG1C | 0.25 | NM_006667 | PGRMC1 | 0.25 |
| NM_014045 | LRP10 | 0.25 | NM_005944 | MOX2 | 0.25 |
| NM_005792 | MPHOSPH6 | 0.25 | NM_001351 | DAZL | 0.25 |
| NM_001374 | DNASE1L2 | 0.25 | NM_014384 | ACAD8 | 0.25 |
| NM_002803 | PSMC2 | 0.25 | NM_178559 | ABCB5 | 0.25 |
| NM_021194 | SLC30A1 | 0.25 | NM_003179 | SYP | 0.25 |
| NM_003548 | HIST2H4 | 0.25 | NM_003961 | RHBDL1 | 0.25 |
| NM_004854 | CHST10 | 0.25 | NM_007172 | NUP50 | 0.25 |
| NM_015331 | NCSTN | 0.25 | NM_014015 | DEXI | 0.25 |
| NM_016628 | WAC | 0.25 | NM_016267 | VGLL1 | 0.25 |
| NM_016599 | MYOZ2 | 0.25 | NM_017854 | FLJ20512 | 0.25 |
| NM_017632 | CARF | 0.25 | NM_018229 | C14ORF108 | 0.25 |
| NM_019612 | R30953_1 | 0.25 | XM_166140 | SFMBT2 | 0.25 |
| NM_031899 | GORASP1 | 0.25 | NM_032477 | MRPL41 | 0.25 |
| NM_022078 | FLJ12455 | 0.25 | NM_030813 | SKD3 | 0.25 |

| | | | | | |
|---|---|---|---|---|---|
| NM_032046 | MSP | 0.25 | NM_032802 | SPPL2A | 0.25 |
| NM_033125 | SLC22A16 | 0.25 | NM_138396 | 39873 | 0.25 |
| NM_080739 | C20ORF141 | 0.25 | NM_145268 | LOC136263 | 0.25 |
| NM_181709 | LOC144347 | 0.25 | NM_152500 | FLJ33084 | 0.25 |
| NM_153015 | FLJ30668 | 0.25 | NM_144723 | FLJ31121 | 0.25 |
| NM_152577 | FLJ25735 | 0.25 | NM_152730 | C6ORF170 | 0.25 |
| XM_166164 | LOC219854 | 0.25 | NM_178169 | RASSF3 | 0.25 |
| NM_153264 | FLJ35880 | 0.25 | XM_208993 | MGC34829 | 0.25 |
| NM_173614 | LOC283820 | 0.25 | XM_379510 | FLJ34048 | 0.25 |
| NM_181648 | LOC286161 | 0.25 | XM_209559 | LOC285303 | 0.25 |
| XM_373894 | LOC388752 | 0.25 | XM_377887 | LOC402210 | 0.25 |
| XM_373106 | LOC391859 | 0.25 | XM_376522 | LOC401270 | 0.25 |
| XM_372323 | LOC389988 | 0.25 | NM_001345 | DGKA | 0.26 |
| NM_004124 | GMFB | 0.26 | NM_014920 | ICK | 0.26 |
| NM_004586 | RPS6KA3 | 0.26 | NM_003876 | C17ORF35 | 0.26 |
| NM_002563 | P2RY1 | 0.26 | NM_023917 | TAS2R9 | 0.26 |
| NM_002474 | MYH11 | 0.26 | NM_006097 | MYL9 | 0.26 |
| NM_003280 | TNNC1 | 0.26 | NM_022066 | E2-230K | 0.26 |
| NM_006203 | PDE4D | 0.26 | NM_018837 | SULF2 | 0.26 |
| NM_173061 | CAST | 0.26 | NM_001167 | BIRC4 | 0.26 |
| NM_015017 | USP33 | 0.26 | NM_001200 | BMP2 | 0.26 |
| NM_021175 | HAMP | 0.26 | NM_002332 | LRP1 | 0.26 |
| NM_001438 | ESRRG | 0.26 | NM_007247 | AP1GBP1 | 0.26 |
| NM_032122 | DTNBP1 | 0.26 | NM_024774 | FLJ21924 | 0.26 |
| NM_001458 | FLNC | 0.26 | NM_001991 | EZH1 | 0.26 |
| NM_005385 | NKTR | 0.26 | NM_003101 | SOAT1 | 0.26 |
| NM_006542 | SPHAR | 0.26 | NM_004589 | SCO1 | 0.26 |
| NM_001635 | AMPH | 0.26 | NM_001387 | DPYSL3 | 0.26 |
| NM_005050 | ABCD4 | 0.26 | NM_020141 | AD-020 | 0.26 |
| NM_002782 | PSG6 | 0.26 | NM_006925 | SFRS5 | 0.26 |
| NM_021107 | MRPS12 | 0.26 | XM_040709 | PTGFRN | 0.26 |
| NM_001001502 | SNCB | 0.26 | NM_003089 | SNRP70 | 0.26 |
| NM_003440 | ZNF140 | 0.26 | NM_033177 | BAT4 | 0.26 |
| NM_014637 | CHPPR | 0.26 | NM_014713 | LAPTM4A | 0.26 |
| NM_006998 | SCGN | 0.26 | NM_015555 | ZNF451 | 0.26 |
| NM_016618 | LOC51315 | 0.26 | NM_018138 | FLJ10560 | 0.26 |
| NM_018224 | FLJ10803 | 0.26 | XM_375568 | HSZFP36 | 0.26 |
| NM_020215 | C14ORF132 | 0.26 | NM_021805 | SIGIRR | 0.26 |
| NM_022480 | FLJ12587 | 0.26 | NM_022167 | XYLT2 | 0.26 |
| NM_024537 | FLJ12118 | 0.26 | NM_024659 | GTDC1 | 0.26 |
| NM_053023 | ZFP91 | 0.26 | NM_025040 | ZNF614 | 0.26 |
| NM_032429 | LZTS2 | 0.26 | XM_373431 | LOC87769 | 0.26 |
| NM_032905 | RBM17 | 0.26 | NM_144575 | CAPN13 | 0.26 |
| XM_044166 | LOC92312 | 0.26 | NM_152303 | ZNF554 | 0.26 |
| NM_031946 | CENTG3 | 0.26 | NM_144638 | MGC29956 | 0.26 |
| NM_080625 | C20ORF160 | 0.26 | NM_178818 | CKLFSF4 | 0.26 |

| | | | | | |
|---|---|---|---|---|---|
| NM_152476 | ZNF560 | 0.26 | NM_173080 | SPRR4 | 0.26 |
| NM_182543 | NOPD1 | 0.26 | NM_173564 | FLJ37538 | 0.26 |
| NM_172139 | IL28B | 0.26 | NM_152785 | GCET2 | 0.26 |
| XM_379295 | LOC285441 | 0.26 | NM_152467 | KLHL10 | 0.26 |
| XM_294680 | LOC338694 | 0.26 | NM_173699 | MGC33889 | 0.26 |
| NM_021250 | LIR9 | 0.26 | NM_207434 | FLJ46363 | 0.26 |
| XM_294906 | LOC339306 | 0.26 | XM_097347 | LOC147941 | 0.26 |
| XM_210054 | LOC286453 | 0.26 | XM_293407 | LOC347541 | 0.26 |
| XM_066695 | LOC139431 | 0.26 | XM_372917 | LOC391353 | 0.26 |
| XM_372807 | LOC391144 | 0.26 | XM_379309 | LOC401171 | 0.26 |
| XM_371680 | LOC389177 | 0.26 | XM_373223 | LOC392159 | 0.26 |
| XM_374145 | LOC389338 | 0.26 | XM_374858 | LOC399844 | 0.26 |
| XM_373627 | LOC388117 | 0.26 | XM_372704 | LOC390876 | 0.26 |
| NM_001004347 | MGC39584 | 0.26 | XM_373967 | LOC388914 | 0.26 |
| NM_005246 | FER | 0.27 | NM_003488 | AKAP1 | 0.27 |
| NM_006201 | PCTK1 | 0.27 | NM_005813 | PRKCN | 0.27 |
| NM_000794 | DRD1 | 0.27 | NM_014264 | PLK4 | 0.27 |
| NM_005912 | MC4R | 0.27 | NM_018969 | SREB3 | 0.27 |
| NM_001795 | CDH5 | 0.27 | NM_000534 | PMS1 | 0.27 |
| NM_001486 | GCKR | 0.27 | NM_002360 | MAFK | 0.27 |
| NM_003878 | GGH | 0.27 | NM_000849 | GSTM3 | 0.27 |
| NM_006755 | TALDO1 | 0.27 | NM_032144 | RAB6C | 0.27 |
| NM_001441 | FAAH | 0.27 | NM_016621 | BHC80 | 0.27 |
| NM_012411 | PTPN22 | 0.27 | NM_004166 | CCL14 | 0.27 |
| NM_173728 | ARHGEF15 | 0.27 | NM_006367 | CAP1 | 0.27 |
| XM_113641 | PPAPDC1 | 0.27 | NM_001111 | ADAR | 0.27 |
| NM_001286 | CLCN6 | 0.27 | NM_006730 | DNASE1L1 | 0.27 |
| NM_002071 | GNAL | 0.27 | NM_002579 | PALM | 0.27 |
| NM_002406 | MGAT1 | 0.27 | NM_006946 | SPTBN2 | 0.27 |
| NM_194428 | DHX34 | 0.27 | NM_014810 | CAP350 | 0.27 |
| NM_005764 | MAP17 | 0.27 | NM_006476 | ATP5L | 0.27 |
| NM_012309 | SHANK2 | 0.27 | XM_039570 | SEC15L2 | 0.27 |
| XM_032571 | KIAA0888 | 0.27 | NM_014117 | PRO0149 | 0.27 |
| NM_016121 | KCTD3 | 0.27 | NM_017915 | FLJ20641 | 0.27 |
| NM_018465 | C9ORF46 | 0.27 | XM_376550 | KIAA1423 | 0.27 |
| NM_020832 | KIAA1441 | 0.27 | NM_023075 | MPPE1 | 0.27 |
| NM_022478 | CDH24 | 0.27 | NM_024333 | FSD1 | 0.27 |
| NM_024582 | FATJ | 0.27 | NM_025124 | FLJ21749 | 0.27 |
| NM_024929 | C6ORF59 | 0.27 | NM_031485 | GRWD1 | 0.27 |
| NM_032330 | CAPNS2 | 0.27 | NM_145041 | MGC20235 | 0.27 |
| NM_181307 | MRPL52 | 0.27 | NM_207322 | LOC145741 | 0.27 |
| NM_080832 | PABPC5 | 0.27 | NM_152544 | FLJ35725 | 0.27 |
| XM_171158 | MGC27348 | 0.27 | NM_152996 | SIAT7C | 0.27 |
| NM_174938 | FRMD3 | 0.27 | NM_173613 | FLJ35785 | 0.27 |
| NM_207426 | FLJ46831 | 0.27 | XM_171040 | LOC253820 | 0.27 |
| XM_209668 | LOC285588 | 0.27 | XM_371711 | LOC389228 | 0.27 |

| | | | | | |
|---|---|---|---|---|---|
| XM_374256 | LOC389638 | 0.27 | XM_378473 | LOC400238 | 0.27 |
| XM_373740 | LOC388406 | 0.27 | XM_378698 | LOC400617 | 0.27 |
| XM_372693 | LOC390846 | 0.27 | NM_001004305 | LOC284757 | 0.27 |
| XM_373925 | LOC388813 | 0.27 | NM_174922 | ADCK5 | 0.28 |
| NM_003885 | CDK5R1 | 0.28 | NM_005544 | IRS1 | 0.28 |
| NM_012290 | TLK1 | 0.28 | NM_003985 | TNK1 | 0.28 |
| NM_003068 | SNAI2 | 0.28 | NM_003454 | ZNF200 | 0.28 |
| NM_006235 | POU2AF1 | 0.28 | NM_005376 | MYCL1 | 0.28 |
| NM_018945 | PDE7B | 0.28 | NM_002194 | INPP1 | 0.28 |
| NM_007293 | C4A | 0.28 | NM_007024 | PL6 | 0.28 |
| NM_000318 | PXMP3 | 0.28 | NM_016323 | HERC5 | 0.28 |
| XM_371813 | KIFC1 | 0.28 | NM_181429 | HT2R55 | 0.28 |
| NM_002211 | ITGB1 | 0.28 | NM_020402 | CHRNA10 | 0.28 |
| NM_004133 | HNF4G | 0.28 | NM_000223 | KRT12 | 0.28 |
| NM_015365 | AMMECR1 | 0.28 | NM_005336 | HDLBP | 0.28 |
| NM_012429 | SEC14L2 | 0.28 | NM_006110 | CD2BP2 | 0.28 |
| NM_005677 | COLQ | 0.28 | NM_015576 | CAST | 0.28 |
| NM_144571 | CNOT6L | 0.28 | NM_001549 | IFIT4 | 0.28 |
| NM_014454 | SESN1 | 0.28 | NM_003487 | TAF15 | 0.28 |
| NM_003069 | SMARCA1 | 0.28 | NM_030979 | PABPC3 | 0.28 |
| NM_153028 | ZNF75A | 0.28 | NM_003704 | C4ORF8 | 0.28 |
| NM_005475 | LNK | 0.28 | NM_007055 | RPC155 | 0.28 |
| NM_012109 | C19ORF4 | 0.28 | NM_014577 | BRD1 | 0.28 |
| NM_014031 | SLC27A6 | 0.28 | NM_016302 | CRBN | 0.28 |
| NM_017799 | C14ORF101 | 0.28 | NM_018186 | FLJ10706 | 0.28 |
| NM_031416 | C18ORF2 | 0.28 | NM_024327 | ZNF576 | 0.28 |
| NM_024815 | FLJ22494 | 0.28 | NM_025149 | FLJ20920 | 0.28 |
| NM_025208 | PDGFD | 0.28 | NM_031293 | PMFBP1 | 0.28 |
| NM_031948 | MPN | 0.28 | NM_032178 | FLJ13291 | 0.28 |
| NM_031451 | MGC4766 | 0.28 | XM_376171 | KIAA1843 | 0.28 |
| NM_133491 | SAT2 | 0.28 | NM_052998 | ODC-P | 0.28 |
| NM_139159 | DPP9 | 0.28 | NM_138455 | CTHRC1 | 0.28 |
| NM_017731 | OSBPL7 | 0.28 | NM_052965 | C1ORF19 | 0.28 |
| NM_153708 | MGC35450 | 0.28 | XM_059832 | LOC136288 | 0.28 |
| NM_152371 | MGC26818 | 0.28 | NM_145051 | MGC4734 | 0.28 |
| NM_194320 | ZNF169 | 0.28 | XM_379433 | LOC285735 | 0.28 |
| NM_176891 | IFNT1 | 0.28 | NM_203405 | LOC388818 | 0.28 |
| XM_370669 | LOC360030 | 0.28 | NM_199341 | LOC374920 | 0.28 |
| NM_207473 | FLJ41733 | 0.28 | NM_017949 | CUEDC1 | 0.28 |
| XM_098030 | LOC151261 | 0.28 | XM_292785 | LOC342865 | 0.28 |
| XM_209505 | LOC285188 | 0.28 | XM_291726 | LOC340843 | 0.28 |
| XM_377783 | LOC402117 | 0.28 | XM_376243 | LOC401072 | 0.28 |
| XM_373864 | LOC388684 | 0.28 | XM_379194 | LOC401068 | 0.28 |
| XM_379149 | LOC401025 | 0.28 | XM_379368 | LOC401215 | 0.28 |
| XM_373491 | LOC387756 | 0.28 | XM_370856 | LOC388116 | 0.28 |
| XM_370968 | LOC388248 | 0.28 | NM_212559 | XKRX | 0.28 |

| | | |
|---|---|---|
| NM_153335 | LYK5 | 0.29 |
| NM_003663 | CGGBP1 | 0.29 |
| NM_002859 | PXN | 0.29 |
| NM_000552 | VWF | 0.29 |
| NM_012197 | RABGAP1 | 0.29 |
| NM_021959 | PPP1R11 | 0.29 |
| NM_001524 | HCRT | 0.29 |
| NM_052962 | IL22RA2 | 0.29 |
| NM_001767 | CD2 | 0.29 |
| NM_014071 | NCOA6 | 0.29 |
| NM_001885 | CRYAB | 0.29 |
| NM_006890 | CEACAM7 | 0.29 |
| NM_006835 | CCNI | 0.29 |
| NM_014287 | PM5 | 0.29 |
| NM_002819 | PTBP1 | 0.29 |
| NM_001357 | DHX9 | 0.29 |
| NM_000951 | PRRG2 | 0.29 |
| NM_005102 | FEZ2 | 0.29 |
| NM_006441 | MTHFS | 0.29 |
| NM_014902 | DLGAP4 | 0.29 |
| NM_014302 | SEC61G | 0.29 |
| NM_015597 | GPSM1 | 0.29 |
| NM_182476 | COQ6 | 0.29 |
| NM_015913 | TLP19 | 0.29 |
| NM_021827 | FLJ23514 | 0.29 |
| NM_025268 | MGC4659 | 0.29 |
| NM_031471 | URP2 | 0.29 |
| NM_032507 | PGBD1 | 0.29 |
| NM_153213 | ARHGEF19 | 0.29 |
| NM_144620 | MGC14816 | 0.29 |
| NM_173578 | FLJ90834 | 0.29 |
| XM_378379 | LOC283432 | 0.29 |
| XM_208766 | KIAA0284 | 0.29 |
| NM_182705 | MGC45871 | 0.29 |
| NM_198527 | MGC45386 | 0.29 |
| XM_293687 | LOC345079 | 0.29 |
| XM_373815 | LOC388573 | 0.29 |
| XM_374414 | LOC392647 | 0.29 |
| XM_371092 | LOC388440 | 0.29 |
| XM_370917 | LOC388189 | 0.29 |
| XM_374317 | LOC389831 | 0.29 |
| XM_380021 | LOC402677 | 0.29 |
| NM_001895 | CSNK2A1 | 0.3 |
| NM_001466 | FZD2 | 0.3 |
| NM_032594 | INSM2 | 0.3 |
| NM_002051 | GATA3 | 0.3 |
| NM_001369 | DNAH5 | 0.29 |
| NM_017523 | HSXIAPAF1 | 0.29 |
| NM_018932 | PCDHB12 | 0.29 |
| NM_003931 | WASF1 | 0.29 |
| NM_015713 | RRM2B | 0.29 |
| NM_003588 | CUL4B | 0.29 |
| NM_002387 | MCC | 0.29 |
| NM_001720 | BMP8B | 0.29 |
| NM_002632 | PGF | 0.29 |
| NM_005602 | CLDN11 | 0.29 |
| NM_001445 | FABP6 | 0.29 |
| NM_000750 | CHRNB4 | 0.29 |
| S73205 | INSAF | 0.29 |
| NM_005807 | PRG4 | 0.29 |
| NM_000773 | CYP2E1 | 0.29 |
| NM_000950 | PRRG1 | 0.29 |
| NM_006937 | SUMO2 | 0.29 |
| XM_290506 | SF3B2 | 0.29 |
| NM_016081 | KIAA0992 | 0.29 |
| XM_037759 | KIAA0376 | 0.29 |
| NM_018958 | C15ORF2 | 0.29 |
| NM_014364 | GAPDS | 0.29 |
| NM_015879 | SIAT8C | 0.29 |
| NM_021247 | PRM3 | 0.29 |
| NM_024791 | PDZK2 | 0.29 |
| NM_032167 | FLJ12363 | 0.29 |
| NM_032125 | DKFZP564D0478 | 0.29 |
| NM_152289 | ZNF561 | 0.29 |
| NM_182499 | DKFZP434M202 | 0.29 |
| NM_144965 | TTC16 | 0.29 |
| XM_173166 | C6ORF191 | 0.29 |
| NM_173658 | FLJ36870 | 0.29 |
| NM_178565 | MGC35555 | 0.29 |
| XM_370615 | DKFZP686O24166 | 0.29 |
| NM_003534 | HIST1H3G | 0.29 |
| XM_375841 | LOC400804 | 0.29 |
| XM_374001 | LOC389019 | 0.29 |
| XM_374763 | LOC393076 | 0.29 |
| XM_373769 | LOC388453 | 0.29 |
| NM_001004333 | MGC71993 | 0.29 |
| NM_001005494 | OR6C4 | 0.29 |
| NM_005713 | COL4A3BP | 0.3 |
| NM_002752 | MAPK9 | 0.3 |
| NM_003381 | VIP | 0.3 |
| NM_002956 | RSN | 0.3 |
| NM_007146 | ZNF161 | 0.3 |

| | | | | | |
|---|---|---|---|---|---|
| NM_000284 | PDHA1 | 0.3 | NM_006002 | UCHL3 | 0.3 |
| NM_003481 | USP5 | 0.3 | NM_001338 | CXADR | 0.3 |
| NM_000271 | NPC1 | 0.3 | NM_001560 | IL13RA1 | 0.3 |
| NM_002124 | HLA-DRB1 | 0.3 | NM_000877 | IL1R1 | 0.3 |
| NM_000514 | GDNF | 0.3 | NM_001904 | CTNNB1 | 0.3 |
| NM_002379 | MATN1 | 0.3 | NM_000482 | APOA4 | 0.3 |
| NM_014058 | DESC1 | 0.3 | NM_000853 | GSTT1 | 0.3 |
| XM_371146 | KIAA1683 | 0.3 | NM_022566 | MESDC1 | 0.3 |
| NM_017779 | DEPDC1 | 0.3 | NM_001906 | CTRB1 | 0.3 |
| NM_018658 | KCNJ16 | 0.3 | NM_006936 | SUMO3 | 0.3 |
| XM_376172 | ZNF142 | 0.3 | NM_005869 | SDCCAG10 | 0.3 |
| NM_006346 | C13ORF24 | 0.3 | NM_015179 | KIAA0690 | 0.3 |
| NM_014332 | SMPX | 0.3 | NM_012155 | EML2 | 0.3 |
| NM_015920 | RPS27L | 0.3 | NM_016257 | HPCAL4 | 0.3 |
| NM_019079 | FLJ10884 | 0.3 | XM_048774 | FBXO42 | 0.3 |
| NM_199043 | C14ORF102 | 0.3 | NM_020882 | KIAA1510 | 0.3 |
| NM_024665 | IRA1 | 0.3 | NM_024891 | FLJ11783 | 0.3 |
| NM_030941 | LOC81691 | 0.3 | NM_032326 | MGC4618 | 0.3 |
| NM_153751 | C21ORF82 | 0.3 | NM_145166 | KIAA1190 | 0.3 |
| NM_033194 | HSPB9 | 0.3 | NM_178467 | HMG4L | 0.3 |
| NM_153218 | FLJ38725 | 0.3 | XM_088567 | ZNF483 | 0.3 |
| NM_153229 | FLJ33318 | 0.3 | XM_173087 | LOC255798 | 0.3 |
| NM_198849 | LOC283514 | 0.3 | XM_291741 | DUPD1 | 0.3 |
| XM_371777 | LOC348938 | 0.3 | XM_208097 | LOC283523 | 0.3 |
| NM_207495 | DKFZP686I15217 | 0.3 | XM_293596 | LOC344797 | 0.3 |
| XM_087200 | LOC151443 | 0.3 | XM_294568 | LOC340501 | 0.3 |
| XM_293293 | LOC347359 | 0.3 | XM_066457 | LOC139067 | 0.3 |
| XM_371701 | LOC389217 | 0.3 | XM_373456 | LOC387662 | 0.3 |
| XM_378535 | LOC400411 | 0.3 | XM_372677 | LOC390805 | 0.3 |
| XM_373685 | LOC388276 | 0.3 | NM_001002921 | AK3L2 | 0.3 |
| NM_033550 | TP53RK | 0.31 | NM_012248 | SEPHS2 | 0.31 |
| NM_000908 | NPR3 | 0.31 | NM_000959 | PTGFR | 0.31 |
| NM_000130 | F5 | 0.31 | NM_031918 | KLF16 | 0.31 |
| NM_001426 | EN1 | 0.31 | NM_138691 | TMC1 | 0.31 |
| NM_001397 | ECE1 | 0.31 | NM_006983 | MMP23B | 0.31 |
| NM_002222 | ITPR1 | 0.31 | NM_000879 | IL5 | 0.31 |
| NM_000416 | IFNGR1 | 0.31 | NM_003376 | VEGF | 0.31 |
| NM_002001 | FCER1A | 0.31 | NM_000815 | GABRD | 0.31 |
| NM_001456 | FLNA | 0.31 | NM_005119 | THRAP3 | 0.31 |
| NM_004925 | AQP3 | 0.31 | NM_005562 | LAMC2 | 0.31 |
| NM_003982 | SLC7A7 | 0.31 | NM_005841 | SPRY1 | 0.31 |
| NM_002102 | GYPE | 0.31 | XM_290540 | MUC6 | 0.31 |
| XM_050041 | MYO1D | 0.31 | NM_015999 | ADIPOR1 | 0.31 |
| NM_005072 | SLC12A4 | 0.31 | NM_004735 | LRRFIP1 | 0.31 |
| NM_003414 | ZNF267 | 0.31 | NM_006073 | TRDN | 0.31 |
| NM_013390 | TMEM2 | 0.31 | NM_014400 | C4.4A | 0.31 |

| | | | | | |
|---|---|---|---|---|---|
| NM_014354 | C6ORF54 | 0.31 | NM_016094 | COMMD2 | 0.31 |
| NM_017897 | FLJ20604 | 0.31 | NM_018271 | FLJ10916 | 0.31 |
| NM_018380 | DDX28 | 0.31 | NM_020151 | STARD7 | 0.31 |
| NM_018924 | PCDHGB3 | 0.31 | NM_022915 | MRPL44 | 0.31 |
| NM_022778 | DKFZP434L0117 | 0.31 | NM_022488 | APG3 | 0.31 |
| NM_030625 | CXXC6 | 0.31 | NM_030801 | MAGED4 | 0.31 |
| NM_032152 | PRAM-1 | 0.31 | NM_078473 | BLP1 | 0.31 |
| NM_030933 | C1ORF14 | 0.31 | NM_032607 | CREB3L3 | 0.31 |
| NM_032721 | TA-NFKBH | 0.31 | XM_291202 | ZNF479 | 0.31 |
| NM_033210 | ZNF502 | 0.31 | NM_173474 | NTAN1 | 0.31 |
| NM_152344 | FLJ30656 | 0.31 | XM_061055 | C10ORF90 | 0.31 |
| NM_152448 | MGC33951 | 0.31 | NM_152489 | MGC35130 | 0.31 |
| XM_379554 | LOC157273 | 0.31 | NM_001001342 | BLOC1S2 | 0.31 |
| NM_152565 | ATP6V0D2 | 0.31 | NM_181719 | LOC255104 | 0.31 |
| NM_173805 | FLJ38723 | 0.31 | NM_178438 | LCE5A | 0.31 |
| NM_173652 | MGC34824 | 0.31 | NM_181537 | KRT25C | 0.31 |
| NM_198547 | FLJ46354 | 0.31 | NM_206832 | UNQ9372 | 0.31 |
| NM_198537 | FLJ44968 | 0.31 | XM_114067 | RP13-15M17.2 | 0.31 |
| XM_172889 | LOC256176 | 0.31 | XM_371299 | LOC388681 | 0.31 |
| XM_374124 | LOC389300 | 0.31 | XM_379485 | LOC401328 | 0.31 |
| XM_375090 | LOC400224 | 0.31 | XM_375955 | LOC400888 | 0.31 |
| NM_004614 | TK2 | 0.32 | NM_006572 | GNA13 | 0.32 |
| NM_181744 | OPN5 | 0.32 | NM_014373 | GPR160 | 0.32 |
| NM_006093 | PRG3 | 0.32 | NM_012278 | ITGB1BP2 | 0.32 |
| NM_006142 | SFN | 0.32 | NM_002134 | HMOX2 | 0.32 |
| NM_014255 | TMEM4 | 0.32 | NM_002592 | PCNA | 0.32 |
| NM_000275 | OCA2 | 0.32 | NM_020638 | FGF23 | 0.32 |
| NM_001711 | BGN | 0.32 | NM_005165 | ALDOC | 0.32 |
| NM_000014 | A2M | 0.32 | NM_003041 | SLC5A2 | 0.32 |
| NM_002214 | ITGB8 | 0.32 | NM_022782 | MPHOSPH9 | 0.32 |
| NM_000540 | RYR1 | 0.32 | NM_006868 | RAB31 | 0.32 |
| NM_000567 | CRP | 0.32 | NM_005325 | HIST1H1A | 0.32 |
| NM_002457 | MUC2 | 0.32 | NM_002346 | LY6E | 0.32 |
| NM_021130 | PPIA | 0.32 | NM_005004 | NDUFB8 | 0.32 |
| NM_002781 | PSG5 | 0.32 | NM_003434 | ZNF133 | 0.32 |
| NM_013256 | ZNF180 | 0.32 | NM_003783 | B3GALT2 | 0.32 |
| NM_004709 | CXORF1 | 0.32 | NM_018344 | SLC29A3 | 0.32 |
| NM_031280 | MRPS15 | 0.32 | NM_024081 | TMG4 | 0.32 |
| NM_024556 | FLJ21103 | 0.32 | NM_030923 | DKFZP566N034 | 0.32 |
| NM_032112 | MRPL43 | 0.32 | NM_032528 | ST6GALII | 0.32 |
| NM_207307 | LOC90288 | 0.32 | NM_020443 | NAV1 | 0.32 |
| NM_033114 | MADP-1 | 0.32 | XM_379540 | LOC157693 | 0.32 |
| NM_138706 | IMAGE:4907098 | 0.32 | NM_182531 | FLJ31875 | 0.32 |
| XM_379396 | LOC221710 | 0.32 | NM_207339 | MGC62094 | 0.32 |
| NM_173821 | FLJ33590 | 0.32 | XM_375527 | LOC339290 | 0.32 |
| NM_201550 | LRRC10 | 0.32 | NM_198404 | KCTD4 | 0.32 |

| | | |
|---|---|---|
| XM_086001 | LOC148003 | 0.32 |
| XM_371280 | LOC388662 | 0.32 |
| XM_353628 | LOC378125 | 0.32 |
| XM_376575 | LOC401307 | 0.32 |
| XM_372589 | LOC390637 | 0.32 |
| NM_020547 | AMHR2 | 0.33 |
| NM_178170 | NEK8 | 0.33 |
| NM_031414 | STK31 | 0.33 |
| NM_001350 | DAXX | 0.33 |
| NM_002487 | NDN | 0.33 |
| NM_006099 | PIAS3 | 0.33 |
| NM_007120 | UGT1A4 | 0.33 |
| XM_376201 | EDEM1 | 0.33 |
| NM_014241 | PTPLA | 0.33 |
| NM_005478 | INSL5 | 0.33 |
| NM_001659 | ARF3 | 0.33 |
| NM_001002905 | OR8G1P | 0.33 |
| NM_001712 | CEACAM1 | 0.33 |
| NM_000991 | RPL28 | 0.33 |
| NM_152701 | ABCA13 | 0.33 |
| NM_030757 | MKRN4 | 0.33 |
| NM_006844 | ILVBL | 0.33 |
| NM_007241 | EAP30 | 0.33 |
| NM_015084 | MRPS27 | 0.33 |
| NM_012094 | PRDX5 | 0.33 |
| NM_016078 | FAM18B | 0.33 |
| NM_017906 | PAK1IP1 | 0.33 |
| NM_017564 | STAB2 | 0.33 |
| NM_021221 | LY6G5B | 0.33 |
| NM_025107 | MYCT1 | 0.33 |
| NM_032601 | MCEE | 0.33 |
| NM_172365 | C14ORF50 | 0.33 |
| NM_153359 | MGC24975 | 0.33 |
| NM_152698 | FLJ38377 | 0.33 |
| XM_291725 | SIAT8F | 0.33 |
| NM_181337 | KAAG1 | 0.33 |
| XM_018432 | LOC146110 | 0.33 |
| XM_168585 | LOC219612 | 0.33 |
| XM_374711 | LOC392621 | 0.33 |
| NM_001012267 | RP11-19J3.3 | 0.33 |
| XM_372425 | LOC390234 | 0.33 |
| XM_373786 | LOC388489 | 0.33 |
| NM_032430 | KIAA1811 | 0.34 |
| NM_138995 | MYO3B | 0.34 |
| NM_006453 | TBL3 | 0.34 |
| NM_005848 | IRLB | 0.34 |
| XM_116384 | LOC202201 | 0.32 |
| XM_290501 | LOC338661 | 0.32 |
| XM_293387 | LOC340578 | 0.32 |
| XM_378064 | LOC402367 | 0.32 |
| NM_004448 | ERBB2 | 0.33 |
| NM_013392 | NRBP | 0.33 |
| NM_003965 | CCRL2 | 0.33 |
| NM_002511 | NMBR | 0.33 |
| NM_005537 | ING1 | 0.33 |
| NM_005933 | MLL | 0.33 |
| NM_006746 | SCML1 | 0.33 |
| NM_001093 | ACACB | 0.33 |
| NM_004999 | MYO6 | 0.33 |
| NM_178570 | RTN4RL2 | 0.33 |
| NM_001924 | GADD45A | 0.33 |
| NM_006589 | C1ORF2 | 0.33 |
| NM_003627 | SLC43A1 | 0.33 |
| NM_005553 | KRN1 | 0.33 |
| NM_032375 | AKT1S1 | 0.33 |
| NM_002934 | RNASE2 | 0.33 |
| NM_003901 | SGPL1 | 0.33 |
| NM_007221 | PMF1 | 0.33 |
| NM_015122 | FCHO1 | 0.33 |
| XM_048457 | KIAA0892 | 0.33 |
| XM_027236 | TTC9 | 0.33 |
| NM_017880 | FLJ20558 | 0.33 |
| NM_017935 | BANK1 | 0.33 |
| NM_018897 | DNAH7 | 0.33 |
| NM_020707 | KIAA1173 | 0.33 |
| NM_030974 | DKFZP434N1923 | 0.33 |
| NM_152409 | FLJ37562 | 0.33 |
| NM_152486 | MGC45873 | 0.33 |
| NM_152655 | ZNF585A | 0.33 |
| NM_181611 | KRTAP19-5 | 0.33 |
| NM_173691 | C9ORF75 | 0.33 |
| NM_198697 | KRTAP12-3 | 0.33 |
| XM_067228 | LOC131149 | 0.33 |
| XM_295178 | LOC340171 | 0.33 |
| XM_380100 | LOC402480 | 0.33 |
| XM_372109 | LOC389749 | 0.33 |
| XM_378362 | LOC400046 | 0.33 |
| NM_001005284 | OR9G4 | 0.33 |
| NM_145245 | LOC115704 | 0.34 |
| NM_007065 | CDC37 | 0.34 |
| NM_004629 | FANCG | 0.34 |
| NM_003624 | RANBP3 | 0.34 |

| | | |
|---|---|---|
| NM_000235 | LIPA | 0.34 |
| NM_020469 | ABO | 0.34 |
| NM_181672 | OGT | 0.34 |
| NM_000797 | DRD4 | 0.34 |
| NM_003824 | FADD | 0.34 |
| XM_370702 | FGD6 | 0.34 |
| NM_005022 | PFN1 | 0.34 |
| NM_004067 | CHN2 | 0.34 |
| NM_002258 | KLRB1 | 0.34 |
| NM_022899 | ACTR8 | 0.34 |
| NM_003529 | HIST1H3A | 0.34 |
| XM_290667 | KIAA0350 | 0.34 |
| NM_012336 | NARF | 0.34 |
| NM_016060 | CGI-125 | 0.34 |
| NM_017535 | DKFZP566H0824 | 0.34 |
| NM_018367 | PHCA | 0.34 |
| NM_024104 | MGC2747 | 0.34 |
| NM_024685 | FLJ23560 | 0.34 |
| XM_378655 | LOC96597 | 0.34 |
| NM_138962 | MSI2 | 0.34 |
| XM_059972 | C9ORF115 | 0.34 |
| NM_177966 | DKFZP667B1218 | 0.34 |
| NM_152990 | PXT1 | 0.34 |
| XM_379373 | LOC257358 | 0.34 |
| NM_207103 | UNQ5783 | 0.34 |
| XM_211079 | LOC283530 | 0.34 |
| XM_290558 | LOC338761 | 0.34 |
| XM_378917 | LOC400796 | 0.34 |
| XM_377849 | LOC402188 | 0.34 |
| NM_001004352 | FLJ16323 | 0.34 |
| NM_004075 | CRY1 | 0.35 |
| NM_016154 | RAB4B | 0.35 |
| NM_001485 | GBX2 | 0.35 |
| NM_018664 | SNFT | 0.35 |
| NM_003422 | ZNF42 | 0.35 |
| NM_016489 | NT5C3 | 0.35 |
| NM_016585 | THEG | 0.35 |
| NM_006477 | RRP22 | 0.35 |
| NM_005603 | ATP8B1 | 0.35 |
| NM_006654 | FRS2 | 0.35 |
| NM_002707 | PPM1G | 0.35 |
| NM_002527 | NTF3 | 0.35 |
| NM_000227 | LAMA3 | 0.35 |
| NM_001297 | CNGB1 | 0.35 |
| NM_006595 | API5 | 0.35 |
| NM_176811 | NALP8 | 0.35 |
| NM_006332 | IFI30 | 0.34 |
| NM_000789 | ACE | 0.34 |
| NM_052963 | TOP1MT | 0.34 |
| NM_004512 | IL11RA | 0.34 |
| NM_030877 | CTNNBL1 | 0.34 |
| NM_000184 | HBG2 | 0.34 |
| NM_014290 | TDRD7 | 0.34 |
| NM_001117 | ADCYAP1 | 0.34 |
| NM_002289 | LALBA | 0.34 |
| NM_152263 | TPM3 | 0.34 |
| NM_053274 | GLMN | 0.34 |
| NM_015173 | TBC1D1 | 0.34 |
| NM_014472 | C10ORF28 | 0.34 |
| NM_016641 | MIR16 | 0.34 |
| NM_017576 | KIF27 | 0.34 |
| NM_018683 | ZNF313 | 0.34 |
| NM_024642 | GALNT12 | 0.34 |
| NM_025027 | ZNF606 | 0.34 |
| NM_152275 | FLJ13946 | 0.34 |
| NM_138403 | MYLC2PL | 0.34 |
| NM_080877 | SLC34A3 | 0.34 |
| NM_181715 | TORC2 | 0.34 |
| NM_152755 | MGC40499 | 0.34 |
| NM_182575 | MGC34799 | 0.34 |
| NM_194270 | MOPT | 0.34 |
| NM_206895 | UNQ830 | 0.34 |
| XM_069612 | LOC135935 | 0.34 |
| XM_372805 | LOC391137 | 0.34 |
| XM_370584 | LOC387720 | 0.34 |
| XM_379894 | LOC402569 | 0.34 |
| NM_023940 | RASL11B | 0.35 |
| NM_002052 | GATA4 | 0.35 |
| NM_018951 | HOXA10 | 0.35 |
| NM_004000 | CHI3L2 | 0.35 |
| NM_001182 | ALDH7A1 | 0.35 |
| NM_014273 | ADAMTS6 | 0.35 |
| NM_000754 | COMT | 0.35 |
| NM_007110 | TEP1 | 0.35 |
| NM_021131 | PPP2R4 | 0.35 |
| NM_005894 | CD5L | 0.35 |
| NM_004962 | GDF10 | 0.35 |
| NM_001962 | EFNA5 | 0.35 |
| NM_001753 | CAV1 | 0.35 |
| NM_006950 | SYN1 | 0.35 |
| NM_003903 | CDC16 | 0.35 |
| NM_002895 | RBL1 | 0.35 |

| | | |
|---|---|---|
| NM_014210 | EVI2A | 0.35 |
| NM_033063 | MAP6 | 0.35 |
| NM_020772 | 182-FIP | 0.35 |
| NM_004598 | SPOCK | 0.35 |
| NM_017575 | C17ORF31 | 0.35 |
| XM_045421 | C20ORF194 | 0.35 |
| NM_019042 | FLJ20485 | 0.35 |
| NM_017908 | FLJ20626 | 0.35 |
| NM_022091 | DJ467N11.1 | 0.35 |
| NM_024060 | MGC5395 | 0.35 |
| NM_024074 | MGC3169 | 0.35 |
| NM_024701 | ASB13 | 0.35 |
| NM_025087 | FLJ21511 | 0.35 |
| NM_032153 | ZIC4 | 0.35 |
| NM_032846 | RAB2B | 0.35 |
| NM_052855 | MGC15396 | 0.35 |
| NM_174908 | C3ORF6 | 0.35 |
| NM_173556 | MGC34732 | 0.35 |
| NM_175895 | FLJ25590 | 0.35 |
| XM_290985 | LOC339692 | 0.35 |
| XM_291161 | LOC340168 | 0.35 |
| XM_375869 | LOC400818 | 0.35 |
| XM_371769 | LOC389321 | 0.35 |
| XM_372142 | LOC389791 | 0.35 |
| NM_001001873 | LOC283174 | 0.35 |
| NM_001982 | ERBB3 | 0.36 |
| NM_003010 | MAP2K4 | 0.36 |
| NM_003952 | RPS6KB2 | 0.36 |
| NM_001779 | CD58 | 0.36 |
| NM_002484 | NUBP1 | 0.36 |
| NM_004180 | TANK | 0.36 |
| NM_007233 | TP53AP1 | 0.36 |
| NM_017946 | FKBP14 | 0.36 |
| NM_004167 | CCL15 | 0.36 |
| NM_000756 | CRH | 0.36 |
| NM_000700 | ANXA1 | 0.36 |
| NM_004969 | IDE | 0.36 |
| NM_005032 | PLS3 | 0.36 |
| NM_006923 | SDF2 | 0.36 |
| NM_004803 | SLC22A14 | 0.36 |
| NM_014858 | HUCEP11 | 0.36 |
| NM_015106 | SRISNF2L | 0.36 |
| NM_016626 | RKHD2 | 0.36 |
| NM_021233 | DLAD | 0.36 |
| NM_024710 | FLJ23469 | 0.36 |
| NM_032886 | MGC15912 | 0.36 |
| NM_001449 | FHL1 | 0.35 |
| NM_005929 | MFI2 | 0.35 |
| NM_003042 | SLC6A1 | 0.35 |
| NM_021648 | TSPYL4 | 0.35 |
| XM_032278 | SIPA1L3 | 0.35 |
| NM_016539 | SIRT6 | 0.35 |
| NM_017646 | TRIT1 | 0.35 |
| NM_020651 | PELI1 | 0.35 |
| NM_022141 | PARVG | 0.35 |
| NM_024592 | FLJ13352 | 0.35 |
| NM_024766 | FLJ23451 | 0.35 |
| NM_025262 | LY6G5C | 0.35 |
| NM_030794 | TDRD3 | 0.35 |
| NM_032825 | ZNF382 | 0.35 |
| NM_138961 | ESAM | 0.35 |
| NM_144689 | FLJ32191 | 0.35 |
| XM_371586 | FLJ25415 | 0.35 |
| NM_198463 | FLJ42117 | 0.35 |
| XM_379477 | LOC285941 | 0.35 |
| NM_003349 | KUA-UEV | 0.35 |
| XM_375667 | LOC400723 | 0.35 |
| XM_373075 | LOC391763 | 0.35 |
| XM_379629 | LOC401500 | 0.35 |
| XM_377028 | LOC401606 | 0.35 |
| XM_371455 | LOC388882 | 0.35 |
| NM_002045 | GAP43 | 0.36 |
| NM_006510 | RFP | 0.36 |
| NM_175067 | TRAR4 | 0.36 |
| NM_004081 | DAZ | 0.36 |
| NM_006548 | IMP-2 | 0.36 |
| NM_002801 | PSMB10 | 0.36 |
| NM_003344 | UBE2H | 0.36 |
| NM_012176 | FBXO4 | 0.36 |
| NM_000887 | ITGAX | 0.36 |
| NM_020183 | ARNTL2 | 0.36 |
| NM_004811 | LPXN | 0.36 |
| NM_002482 | NASP | 0.36 |
| NM_173703 | MTATP8 | 0.36 |
| NM_003004 | SECTM1 | 0.36 |
| NM_003819 | PABPC4 | 0.36 |
| NM_006363 | SEC23B | 0.36 |
| XM_049037 | TNRC9 | 0.36 |
| NM_017870 | HSPA5BP1 | 0.36 |
| NM_021248 | CDH22 | 0.36 |
| NM_024293 | C2ORF17 | 0.36 |
| NM_052926 | PNMA5 | 0.36 |

| | | | | | |
|---|---|---|---|---|---|
| NM_144590 | ANKRD22 | 0.36 | NM_152447 | LRFN5 | 0.36 |
| NM_145274 | MGC21518 | 0.36 | XM_372592 | LOC145814 | 0.36 |
| NM_138703 | MAGEE2 | 0.36 | NM_198401 | LOC157567 | 0.36 |
| XM_371849 | C6ORF198 | 0.36 | NM_181783 | SMILE | 0.36 |
| NM_182546 | MGC33530 | 0.36 | NM_198215 | FAM13C1 | 0.36 |
| XM_378912 | LOC284688 | 0.36 | NM_178554 | FLJ33207 | 0.36 |
| NM_203304 | RKHD1 | 0.36 | NM_206923 | YY2 | 0.36 |
| XM_292700 | LOC342808 | 0.36 | XM_293984 | LOC345829 | 0.36 |
| XM_378009 | LOC402326 | 0.36 | XM_372616 | LOC390688 | 0.36 |
| NM_005308 | GRK5 | 0.37 | NM_020988 | GNAO1 | 0.37 |
| NM_015236 | LPHN3 | 0.37 | NM_001769 | CD9 | 0.37 |
| NM_173631 | ZNF547 | 0.37 | NM_006912 | RIT1 | 0.37 |
| NM_015394 | ZNF10 | 0.37 | NM_021572 | ENPP5 | 0.37 |
| NM_015907 | LAP3 | 0.37 | NM_012250 | RRAS2 | 0.37 |
| NM_003482 | MLL2 | 0.37 | NM_022822 | KLC2 | 0.37 |
| NM_002117 | HLA-C | 0.37 | NM_002941 | ROBO1 | 0.37 |
| NM_023004 | RTN4R | 0.37 | NM_024025 | MGC1136 | 0.37 |
| NM_005137 | DGCR2 | 0.37 | NM_004406 | DMBT1 | 0.37 |
| NM_003123 | SPN | 0.37 | NM_021101 | CLDN1 | 0.37 |
| NM_022121 | PERP | 0.37 | NM_170753 | PGBD3 | 0.37 |
| NM_014139 | SCN11A | 0.37 | NM_014781 | RB1CC1 | 0.37 |
| NM_001324 | CSTF1 | 0.37 | NM_003817 | ADAM7 | 0.37 |
| XM_377962 | PMS2L1 | 0.37 | NM_004159 | PSMB8 | 0.37 |
| NM_004596 | SNRPA | 0.37 | NM_003203 | C2ORF3 | 0.37 |
| NM_006862 | TDRKH | 0.37 | NM_015426 | DKFZP434C245 | 0.37 |
| NM_014469 | HNRNPG-T | 0.37 | NM_017573 | PCSK4 | 0.37 |
| NM_018132 | C6ORF139 | 0.37 | NM_018099 | MLSTD1 | 0.37 |
| NM_021181 | SLAMF7 | 0.37 | NM_023011 | UPF3A | 0.37 |
| NM_024508 | ZBED2 | 0.37 | NM_024833 | FLJ23506 | 0.37 |
| NM_024949 | BOMB | 0.37 | NM_032128 | DKFZP566M114 | 0.37 |
| NM_032485 | MCM8 | 0.37 | NM_032762 | MGC16121 | 0.37 |
| NM_138352 | LOC90378 | 0.37 | NM_138437 | GASP2 | 0.37 |
| NM_182522 | TAFA4 | 0.37 | NM_178821 | FLJ25955 | 0.37 |
| NM_153238 | MGC22001 | 0.37 | XM_168060 | C6ORF154 | 0.37 |
| NM_173853 | KRTCAP3 | 0.37 | NM_175904 | FLJ40121 | 0.37 |
| XM_209902 | ZNF252 | 0.37 | NM_178547 | ARCH | 0.37 |
| NM_207448 | FLJ45256 | 0.37 | XM_036942 | LOC150221 | 0.37 |
| XM_376423 | LOC401207 | 0.37 | XM_377883 | LOC402206 | 0.37 |
| XM_375302 | LOC400499 | 0.37 | NM_001001791 | C10ORF55 | 0.37 |
| NM_001004124 | OR4P4 | 0.37 | NM_004963 | GUCY2C | 0.38 |
| NM_016616 | TXNDC3 | 0.38 | NM_007271 | STK38 | 0.38 |
| NM_021205 | RHOU | 0.38 | NM_000243 | MEFV | 0.38 |
| NM_005016 | PCBP2 | 0.38 | NM_000548 | TSC2 | 0.38 |
| NM_005636 | SSX4 | 0.38 | NM_005375 | MYB | 0.38 |
| NM_033502 | TRERF1 | 0.38 | NM_001868 | CPA1 | 0.38 |
| NM_014324 | AMACR | 0.38 | NM_138809 | LOC134147 | 0.38 |

| | | | | | |
|---|---|---|---|---|---|
| NM_005540 | INPP5B | 0.38 | NM_003358 | UGCG | 0.38 |
| NM_001176 | ARHGDIG | 0.38 | NM_004827 | ABCG2 | 0.38 |
| NM_001993 | F3 | 0.38 | NM_004321 | KIF1A | 0.38 |
| NM_024888 | FLJ11535 | 0.38 | NM_000081 | CHS1 | 0.38 |
| NM_178831 | GATS | 0.38 | NM_003544 | HIST1H4B | 0.38 |
| NM_005007 | NFKBIL1 | 0.38 | NM_002865 | RAB2 | 0.38 |
| NM_130463 | ATP6V1G2 | 0.38 | NM_006232 | POLR2H | 0.38 |
| NM_013255 | MKLN1 | 0.38 | NM_005073 | SLC15A1 | 0.38 |
| NM_006289 | TLN1 | 0.38 | NM_014850 | SRGAP2 | 0.38 |
| NM_006336 | C9ORF60 | 0.38 | NM_006642 | SDCCAG8 | 0.38 |
| XM_351842 | SLC35D2 | 0.38 | NM_178424 | SOX30 | 0.38 |
| NM_006567 | FARS1 | 0.38 | NM_177424 | STX12 | 0.38 |
| NM_153681 | DSCR5 | 0.38 | NM_017432 | PTOV1 | 0.38 |
| XM_372879 | C21ORF57 | 0.38 | NM_016329 | SFMBT1 | 0.38 |
| NM_016532 | SKIP | 0.38 | NM_020309 | SLC17A7 | 0.38 |
| NM_018916 | PCDHGA3 | 0.38 | XM_029429 | KIAA1328 | 0.38 |
| NM_030651 | C6ORF31 | 0.38 | NM_025214 | SE57-1 | 0.38 |
| NM_052831 | C6ORF192 | 0.38 | NM_152342 | CDYL2 | 0.38 |
| NM_153018 | FLJ30726 | 0.38 | NM_173848 | LOC138046 | 0.38 |
| NM_080875 | LOC142678 | 0.38 | NM_207335 | FLJ46299 | 0.38 |
| NM_152623 | FLJ37927 | 0.38 | NM_152582 | MGC27005 | 0.38 |
| XM_114430 | LOC202051 | 0.38 | NM_145003 | FLJ31164 | 0.38 |
| NM_207395 | FLJ45850 | 0.38 | XM_293104 | LOC343574 | 0.38 |
| XM_379235 | LOC401103 | 0.38 | XM_373520 | LOC387824 | 0.38 |
| XM_373531 | LOC387854 | 0.38 | XM_372410 | LOC390192 | 0.38 |
| NM_004563 | PCK2 | 0.39 | NM_017433 | MYO3A | 0.39 |
| NM_000678 | ADRA1D | 0.39 | NM_016276 | SGK2 | 0.39 |
| NM_000844 | GRM7 | 0.39 | NM_175057 | TRAR3 | 0.39 |
| NM_153604 | MYOCD | 0.39 | NM_013232 | PDCD6 | 0.39 |
| NM_005825 | RASGRP2 | 0.39 | NM_000523 | HOXD13 | 0.39 |
| NM_003322 | TULP1 | 0.39 | NM_001464 | ADAM2 | 0.39 |
| NM_000072 | CD36 | 0.39 | NM_000074 | TNFSF5 | 0.39 |
| NM_002521 | NPPB | 0.39 | NM_002235 | KCNA6 | 0.39 |
| NM_000620 | NOS1 | 0.39 | NM_007366 | PLA2R1 | 0.39 |
| NM_000311 | PRNP | 0.39 | NM_002533 | NVL | 0.39 |
| XM_029438 | RUTBC1 | 0.39 | NM_001073 | UGT2B11 | 0.39 |
| NM_030913 | SEMA6C | 0.39 | NM_014994 | MAPKBP1 | 0.39 |
| NM_003419 | ZNF345 | 0.39 | NM_016192 | TMEFF2 | 0.39 |
| NM_015578 | C19ORF13 | 0.39 | XM_371074 | DKFZP564D166 | 0.39 |
| NM_016002 | CGI-49 | 0.39 | NM_018034 | FLJ10233 | 0.39 |
| NM_018502 | PRO1580 | 0.39 | NM_021191 | NEUROD4 | 0.39 |
| NM_024037 | MGC2603 | 0.39 | NM_022573 | TSPY2 | 0.39 |
| NM_024101 | MLPH | 0.39 | NM_024641 | MANEA | 0.39 |
| NM_025263 | PRR3 | 0.39 | NM_031926 | TTY7 | 0.39 |
| NM_032446 | MEGF10 | 0.39 | NM_032860 | C6ORF93 | 0.39 |
| NM_033514 | LIMS3 | 0.39 | NM_033412 | MCART1 | 0.39 |

| | | | | | |
|---|---|---|---|---|---|
| XM_117117 | FLJ13072 | 0.39 | NM_182497 | KA36 | 0.39 |
| XM_371468 | MGC40042 | 0.39 | NM_173355 | UPP2 | 0.39 |
| NM_145207 | SPATA5 | 0.39 | NM_173555 | TYSND1 | 0.39 |
| NM_153374 | MGC35274 | 0.39 | NM_173357 | SSX6 | 0.39 |
| NM_173624 | FLJ40504 | 0.39 | NM_173619 | MGC34761 | 0.39 |
| NM_205851 | FLJ35700 | 0.39 | NM_180990 | LGICZ | 0.39 |
| NM_182631 | LOC348840 | 0.39 | NM_004142 | MMPL1 | 0.39 |
| XM_371277 | LOC388659 | 0.39 | XM_087901 | LOC154288 | 0.39 |
| XM_379380 | LOC401222 | 0.39 | XM_373097 | LOC391840 | 0.39 |
| NM_014867 | KIAA0711 | 0.39 | XM_375608 | LOC400692 | 0.39 |
| XM_290923 | KIAA1639 | 0.4 | NM_002609 | PDGFRB | 0.4 |
| NM_015678 | NBEA | 0.4 | NM_004041 | ARRB1 | 0.4 |
| NM_007040 | HNRPUL1 | 0.4 | NM_005702 | ERAL1 | 0.4 |
| NM_005324 | H3F3B | 0.4 | NM_000179 | MSH6 | 0.4 |
| NM_006626 | ZNF482 | 0.4 | NM_000209 | IPF1 | 0.4 |
| NM_006354 | TADA3L | 0.4 | NM_000663 | ABAT | 0.4 |
| NM_015093 | MAP3K7IP2 | 0.4 | NM_000520 | HEXA | 0.4 |
| NM_012249 | RHOQ | 0.4 | NM_021652 | SMA4 | 0.4 |
| NM_003496 | TRRAP | 0.4 | NM_002710 | PPP1CC | 0.4 |
| NM_002688 | PNUTL1 | 0.4 | NM_004489 | GPS2 | 0.4 |
| NM_018659 | C17 | 0.4 | NM_000802 | FOLR1 | 0.4 |
| NM_005210 | CRYGB | 0.4 | NM_000477 | ALB | 0.4 |
| NM_020960 | GPR107 | 0.4 | NM_005332 | HBZ | 0.4 |
| NM_148959 | HUS1B | 0.4 | NM_001285 | CLCA1 | 0.4 |
| NM_006899 | IDH3B | 0.4 | NM_001930 | DHPS | 0.4 |
| NM_000824 | GLRB | 0.4 | NM_021965 | PGM5 | 0.4 |
| NM_003451 | ZNF177 | 0.4 | NM_007208 | MRPL3 | 0.4 |
| NM_015397 | WDR40A | 0.4 | XM_375171 | DKFZP434L187 | 0.4 |
| NM_014404 | CACNG5 | 0.4 | NM_016185 | HN1 | 0.4 |
| NM_058180 | C21ORF58 | 0.4 | NM_018404 | CENTA2 | 0.4 |
| NM_018059 | FLJ10324 | 0.4 | NM_018652 | GOLGA6 | 0.4 |
| NM_019893 | ASAH2 | 0.4 | NM_022483 | FLJ21657 | 0.4 |
| NM_024078 | MGC3162 | 0.4 | NM_024948 | C10ORF97 | 0.4 |
| NM_025225 | C22ORF20 | 0.4 | NM_030755 | TXNDC | 0.4 |
| XM_378186 | MGC15634 | 0.4 | NM_152281 | FLJ11752 | 0.4 |
| NM_181599 | KRTAP13-1 | 0.4 | NM_152657 | GGN | 0.4 |
| NM_153043 | FLJ37078 | 0.4 | XM_379322 | LOC340109 | 0.4 |
| NM_182894 | CHX10 | 0.4 | NM_198503 | SLICK | 0.4 |
| NM_207507 | FLJ45202 | 0.4 | XM_210400 | LOC285031 | 0.4 |
| XM_372911 | LOC391343 | 0.4 | XM_371491 | LOC388946 | 0.4 |
| XM_379534 | LOC401437 | 0.4 | XM_370834 | LOC388077 | 0.4 |
| XM_373756 | LOC388429 | 0.4 | XM_373771 | LOC388456 | 0.4 |
| XM_373397 | LOC392584 | 0.4 | NM_001005470 | OR4B1 | 0.4 |
| NM_001005491 | OR10AG1 | 0.4 | NM_004246 | GLP2R | 0.41 |
| NM_032503 | GPR145 | 0.41 | NM_004224 | GPR50 | 0.41 |
| NM_006176 | NRGN | 0.41 | NM_000185 | SERPIND1 | 0.41 |

| | | | | | |
|---|---|---|---|---|---|
| NM_004688 | NMI | 0.41 | NM_000755 | CRAT | 0.41 |
| NM_003937 | KYNU | 0.41 | NM_000487 | ARSA | 0.41 |
| NM_031295 | WBSCR21 | 0.41 | NM_003929 | RAB7L1 | 0.41 |
| NM_003812 | ADAM23 | 0.41 | NM_025245 | PBX4 | 0.41 |
| NM_006156 | NEDD8 | 0.41 | NM_005744 | ARIH1 | 0.41 |
| NM_001144 | AMFR | 0.41 | NM_005605 | PPP3CC | 0.41 |
| NM_021956 | GRIK2 | 0.41 | NM_006522 | WNT6 | 0.41 |
| NM_014880 | DCL-1 | 0.41 | NM_006529 | GLRA3 | 0.41 |
| NM_001459 | FLT3LG | 0.41 | NM_006085 | BPNT1 | 0.41 |
| NM_012317 | LDOC1 | 0.41 | NM_006898 | HOXD3 | 0.41 |
| NM_007042 | RPP14 | 0.41 | NM_175569 | XG | 0.41 |
| NM_004046 | ATP5A1 | 0.41 | NM_014289 | CAPN6 | 0.41 |
| NM_004950 | DSPG3 | 0.41 | NM_033025 | 7H3 | 0.41 |
| NM_033631 | LUZP1 | 0.41 | XM_035863 | ZNF37A | 0.41 |
| NM_003823 | TNFRSF6B | 0.41 | NM_006402 | HBXIP | 0.41 |
| NM_006680 | ME3 | 0.41 | NM_022375 | OCLM | 0.41 |
| NM_006577 | B3GNT1 | 0.41 | XM_374829 | PDCD11 | 0.41 |
| XM_059482 | FLJ00133 | 0.41 | NM_015529 | MOXD1 | 0.41 |
| NM_015545 | PTCD1 | 0.41 | NM_012199 | EIF2C1 | 0.41 |
| NM_015700 | HIRIP5 | 0.41 | NM_016548 | GOLPH2 | 0.41 |
| NM_015921 | C6ORF82 | 0.41 | NM_017443 | POLE3 | 0.41 |
| NM_018050 | MANSC1 | 0.41 | NM_018163 | FLJ10634 | 0.41 |
| NM_018000 | FLJ10116 | 0.41 | NM_020147 | THAP10 | 0.41 |
| NM_020652 | ZNF286 | 0.41 | NM_021948 | BCAN | 0.41 |
| NM_030783 | PTDSS2 | 0.41 | NM_033088 | FAM40A | 0.41 |
| NM_052954 | CYYR1 | 0.41 | NM_052960 | RBP7 | 0.41 |
| NM_153033 | KCTD7 | 0.41 | NM_152497 | FLJ32206 | 0.41 |
| NM_176791 | C20ORF65 | 0.41 | NM_182527 | CABP7 | 0.41 |
| NM_145013 | MGC35558 | 0.41 | NM_012184 | FOXD4L1 | 0.41 |
| NM_175061 | JAZF1 | 0.41 | NM_153613 | LOC254531 | 0.41 |
| XM_211305 | LOC284021 | 0.41 | NM_182759 | TAFA3 | 0.41 |
| XM_290777 | LOC339231 | 0.41 | NM_175923 | MGC42630 | 0.41 |
| NM_181808 | POLN | 0.41 | NM_198085 | RNF148 | 0.41 |
| XM_292850 | LOC343930 | 0.41 | XM_098008 | LOC151154 | 0.41 |
| XM_066443 | LOC139046 | 0.41 | XM_379527 | LOC401431 | 0.41 |
| XM_379827 | LOC402494 | 0.41 | XM_379986 | LOC402633 | 0.41 |
| XM_373233 | LOC392179 | 0.41 | XM_372471 | LOC390345 | 0.41 |
| XM_377259 | LOC401713 | 0.41 | NM_001004346 | MTHFD2L | 0.41 |
| XM_374498 | LOC392758 | 0.41 | XM_374501 | LOC392762 | 0.41 |
| NM_002378 | MATK | 0.42 | NM_003215 | TEC | 0.42 |
| NM_014621 | HOXD4 | 0.42 | NM_014383 | TZFP | 0.42 |
| NM_033285 | TP53INP1 | 0.42 | NM_002180 | IGHMBP2 | 0.42 |
| NM_002426 | MMP12 | 0.42 | NM_005038 | PPID | 0.42 |
| NM_006001 | TUBA2 | 0.42 | NM_002615 | SERPINF1 | 0.42 |
| NM_007013 | WWP1 | 0.42 | NM_004287 | GOSR2 | 0.42 |
| NM_012181 | FKBP8 | 0.42 | NM_004576 | PPP2R2B | 0.42 |

| | | | | | |
|---|---|---|---|---|---|
| NM_005448 | BMP15 | 0.42 | NM_000527 | LDLR | 0.42 |
| NM_004112 | FGF11 | 0.42 | NM_015099 | CAMTA2 | 0.42 |
| NM_005190 | CCNC | 0.42 | NM_153716 | HSFY2 | 0.42 |
| NM_004859 | CLTC | 0.42 | NM_021963 | NAP1L2 | 0.42 |
| NM_001001503 | NDUFV3 | 0.42 | NM_003100 | SNX2 | 0.42 |
| NM_003512 | HIST1H2AC | 0.42 | NM_004298 | NUP155 | 0.42 |
| NM_148920 | PIGQ | 0.42 | NM_021023 | FHR-3 | 0.42 |
| NM_013974 | DDAH2 | 0.42 | NM_015336 | HIP14 | 0.42 |
| NM_014311 | SMUG1 | 0.42 | NM_014337 | PPIL2 | 0.42 |
| NM_014067 | LRP16 | 0.42 | NM_014393 | STAU2 | 0.42 |
| NM_015717 | CD207 | 0.42 | NM_016220 | ZFD25 | 0.42 |
| NM_016249 | MAGEC2 | 0.42 | NM_018334 | LRRN3 | 0.42 |
| NM_018048 | FLJ10292 | 0.42 | NM_017841 | FLJ20487 | 0.42 |
| NM_017994 | FLJ10099 | 0.42 | NM_020237 | C8ORF17 | 0.42 |
| NM_020468 | SNX14 | 0.42 | NM_020120 | UGCGL1 | 0.42 |
| NM_020346 | SLC17A6 | 0.42 | NM_022374 | ARL6IP2 | 0.42 |
| NM_022482 | ZNF336 | 0.42 | NM_022829 | SLC13A3 | 0.42 |
| NM_022906 | FLJ13195 | 0.42 | NM_025143 | C21ORF96 | 0.42 |
| NM_030928 | CDT1 | 0.42 | NM_032287 | DKFZP761O17121 | 0.42 |
| NM_032873 | KIAA1959 | 0.42 | NM_033106 | GALP | 0.42 |
| XM_378848 | LOC85028 | 0.42 | XM_087672 | KIAA1935 | 0.42 |
| XM_057107 | KIAA1937 | 0.42 | NM_138389 | LOC92689 | 0.42 |
| NM_152315 | MGC34290 | 0.42 | NM_174891 | C14ORF79 | 0.42 |
| NM_138790 | LOC122618 | 0.42 | NM_182500 | FLJ25143 | 0.42 |
| NM_138794 | LYPLAL1 | 0.42 | NM_173850 | SERPINA12 | 0.42 |
| NM_152451 | GUP1 | 0.42 | NM_182506 | FLJ32965 | 0.42 |
| NM_152431 | PIWIL4 | 0.42 | NM_138287 | BBAP | 0.42 |
| NM_182528 | C1QL2 | 0.42 | NM_144979 | MGC27016 | 0.42 |
| XM_168055 | C6ORF185 | 0.42 | XM_378232 | LOC219690 | 0.42 |
| NM_152759 | MGC35140 | 0.42 | XM_373624 | LOC283697 | 0.42 |
| XM_209204 | MGC26694 | 0.42 | XM_379535 | LOC285889 | 0.42 |
| NM_198559 | MGC50811 | 0.42 | XM_062788 | LOC121792 | 0.42 |
| XM_211896 | LOC285435 | 0.42 | XM_065750 | LOC130500 | 0.42 |
| XM_117451 | LOC202775 | 0.42 | XM_377997 | LOC402318 | 0.42 |
| XM_370638 | LOC387790 | 0.42 | XM_372433 | LOC390259 | 0.42 |
| XM_373808 | LOC388549 | 0.42 | NM_001005486 | OR4K15 | 0.42 |
| NM_004336 | BUB1 | 0.43 | NM_000075 | CDK4 | 0.43 |
| NM_002253 | KDR | 0.43 | NM_001079 | ZAP70 | 0.43 |
| NM_004949 | DSC2 | 0.43 | NM_020248 | CTNNBIP1 | 0.43 |
| NM_018682 | MLL5 | 0.43 | NM_002979 | SCP2 | 0.43 |
| NM_001310 | CREBL2 | 0.43 | NM_006856 | ATF7 | 0.43 |
| NM_005644 | TAF12 | 0.43 | NM_000378 | WT1 | 0.43 |
| NM_017444 | CHRAC1 | 0.43 | NM_007204 | DDX20 | 0.43 |
| NM_021988 | UBE2V1 | 0.43 | NM_012414 | RAB3-GAP150 | 0.43 |
| NM_004205 | USP2 | 0.43 | NM_002721 | PPP6C | 0.43 |
| NM_003868 | FGF16 | 0.43 | NM_005202 | COL8A2 | 0.43 |

| | | |
|---|---|---|
| NM_133497 | KCNV2 | 0.43 |
| NM_002287 | LAIR1 | 0.43 |
| NM_001321 | CSRP2 | 0.43 |
| NM_005034 | POLR2K | 0.43 |
| NM_006508 | REGL | 0.43 |
| NM_003315 | DNAJC7 | 0.43 |
| NM_001075 | UGT2B10 | 0.43 |
| NM_014780 | CUL7 | 0.43 |
| NM_032632 | PAPOLA | 0.43 |
| NM_014309 | RBM9 | 0.43 |
| NM_019025 | SMOX | 0.43 |
| NM_016173 | HEMK | 0.43 |
| NM_053277 | CLIC6 | 0.43 |
| NM_018294 | CWF19L1 | 0.43 |
| NM_020818 | KIAA1409 | 0.43 |
| NM_022107 | GPSM3 | 0.43 |
| NM_024860 | FLJ21148 | 0.43 |
| NM_032773 | LRCH3 | 0.43 |
| XM_053966 | LOC113230 | 0.43 |
| NM_152313 | SLC36A4 | 0.43 |
| NM_152540 | SCFD2 | 0.43 |
| XM_208270 | BA395L14.13 | 0.43 |
| NM_198541 | UNQ644 | 0.43 |
| XM_372792 | LOC391092 | 0.43 |
| XM_374065 | LOC389183 | 0.43 |
| XM_374139 | LOC389330 | 0.43 |
| XM_376655 | LOC401391 | 0.43 |
| XM_375602 | LOC400687 | 0.43 |
| XM_378730 | LOC400638 | 0.43 |
| NM_001004467 | OR10J3 | 0.43 |
| NM_054106 | OR5AC2 | 0.43 |
| NM_006039 | MRC2 | 0.44 |
| NM_014322 | OPN3 | 0.44 |
| NM_001060 | TBXA2R | 0.44 |
| NM_021926 | ALX4 | 0.44 |
| NM_001919 | DCI | 0.44 |
| NM_178154 | FUT8 | 0.44 |
| NM_005173 | ATP2A3 | 0.44 |
| NM_018438 | FBXO6 | 0.44 |
| NM_007240 | DUSP12 | 0.44 |
| NM_004874 | BAG4 | 0.44 |
| NM_000696 | ALDH9A1 | 0.44 |
| NM_032304 | HAGHL | 0.44 |
| NM_014159 | HYPB | 0.44 |
| NM_014433 | RTDR1 | 0.44 |
| XM_371844 | TSPYL1 | 0.44 |
| NM_001807 | CEL | 0.43 |
| NM_006452 | PAICS | 0.43 |
| NM_001499 | GLE1L | 0.43 |
| NM_021038 | MBNL1 | 0.43 |
| NM_001005217 | FRG2 | 0.43 |
| NM_152653 | UBE2E2 | 0.43 |
| NM_017715 | ZNF3 | 0.43 |
| NM_006407 | ARL6IP5 | 0.43 |
| XM_374936 | KIAA1052 | 0.43 |
| NM_017901 | TPCN1 | 0.43 |
| NM_018962 | DSCR6 | 0.43 |
| NM_014164 | FXYD5 | 0.43 |
| NM_018062 | FANCL | 0.43 |
| NM_033049 | MUC13 | 0.43 |
| NM_021237 | SELK | 0.43 |
| NM_022466 | ZNFN1A5 | 0.43 |
| NM_031477 | MGC10500 | 0.43 |
| NM_032648 | MGC10820 | 0.43 |
| NM_138462 | ZMYND19 | 0.43 |
| NM_182513 | SPC24 | 0.43 |
| NM_175737 | LOC152831 | 0.43 |
| NM_207397 | UNQ6122 | 0.43 |
| XM_209936 | LOC286187 | 0.43 |
| XM_375821 | LOC400799 | 0.43 |
| XM_374196 | LOC389452 | 0.43 |
| XM_376469 | LOC401236 | 0.43 |
| XM_370709 | LOC387894 | 0.43 |
| XM_373659 | LOC388204 | 0.43 |
| XM_378753 | LOC400655 | 0.43 |
| XM_378124 | LOC402414 | 0.43 |
| NM_004734 | DCAMKL1 | 0.44 |
| NM_032553 | FKSG79 | 0.44 |
| NM_007203 | AKAP2 | 0.44 |
| NM_006389 | HYOU1 | 0.44 |
| NM_005642 | TAF7 | 0.44 |
| NM_004483 | GCSH | 0.44 |
| NM_005539 | INPP5A | 0.44 |
| NM_020886 | USP28 | 0.44 |
| NM_006404 | PROCR | 0.44 |
| NM_002351 | SH2D1A | 0.44 |
| NM_005903 | SMAD5 | 0.44 |
| NM_000334 | SCN4A | 0.44 |
| NM_016359 | NUSAP1 | 0.44 |
| NM_020229 | PRDM11 | 0.44 |
| NM_005625 | SDCBP | 0.44 |
| NM_004293 | GDA | 0.44 |

| | | | | | |
|---|---|---|---|---|---|
| NM_016138 | COQ7 | 0.44 | NM_007178 | STRAP | 0.44 |
| NM_006839 | IMMT | 0.44 | NM_006683 | FAM12A | 0.44 |
| NM_012088 | PGLS | 0.44 | NM_012413 | QPCT | 0.44 |
| XM_036708 | KIAA0368 | 0.44 | NM_015453 | THUMPD3 | 0.44 |
| NM_016608 | ARMCX1 | 0.44 | NM_016144 | COMMD10 | 0.44 |
| NM_017938 | FLJ20716 | 0.44 | NM_018165 | PB1 | 0.44 |
| NM_017544 | NRF | 0.44 | NM_018925 | PCDHGB5 | 0.44 |
| XM_044434 | KIAA1458 | 0.44 | NM_024711 | HIAN2 | 0.44 |
| NM_031938 | BCDO2 | 0.44 | NM_032299 | MGC2714 | 0.44 |
| NM_032641 | GRCC9 | 0.44 | NM_138355 | SCRN2 | 0.44 |
| NM_138433 | LOC113730 | 0.44 | NM_133496 | SLC30A7 | 0.44 |
| NM_152499 | MGC45441 | 0.44 | NM_152888 | COL22A1 | 0.44 |
| NM_144998 | STRA13 | 0.44 | NM_207344 | LOC283377 | 0.44 |
| NM_197941 | LOC345667 | 0.44 | NM_206827 | RASL11A | 0.44 |
| NM_198531 | ATP9B | 0.44 | NM_206834 | C6ORF201 | 0.44 |
| XM_291627 | LOC343531 | 0.44 | XM_086732 | LOC149950 | 0.44 |
| XM_085463 | LOC146439 | 0.44 | XM_374120 | LOC389288 | 0.44 |
| XM_379660 | LOC401545 | 0.44 | XM_379990 | LOC402637 | 0.44 |
| NM_000189 | HK2 | 0.45 | NM_001408 | CELSR2 | 0.45 |
| NM_015282 | CLASP1 | 0.45 | NM_003281 | TNNI1 | 0.45 |
| NM_006633 | IQGAP2 | 0.45 | NM_013434 | CSEN | 0.45 |
| NM_003097 | SNRPN | 0.45 | NM_006060 | ZNFN1A1 | 0.45 |
| NM_000497 | CYP11B1 | 0.45 | NM_001398 | ECH1 | 0.45 |
| NM_021139 | UGT2B4 | 0.45 | NM_002691 | POLD1 | 0.45 |
| NM_015294 | TRIM37 | 0.45 | NM_006247 | PPP5C | 0.45 |
| NM_000804 | FOLR3 | 0.45 | NM_052887 | TIRAP | 0.45 |
| NM_021068 | IFNA4 | 0.45 | NM_030753 | WNT3 | 0.45 |
| NM_000424 | KRT5 | 0.45 | NM_005330 | HBE1 | 0.45 |
| NM_172363 | HMG1L10 | 0.45 | NM_006308 | HSPB3 | 0.45 |
| AK090464 | IGHG3 | 0.45 | NM_001928 | DF | 0.45 |
| NM_002302 | LECT2 | 0.45 | NM_014234 | HSD17B8 | 0.45 |
| NM_181503 | EXOSC8 | 0.45 | NM_015286 | DMN | 0.45 |
| NM_015085 | GARNL4 | 0.45 | NM_012229 | NT5C2 | 0.45 |
| NM_014285 | EXOSC2 | 0.45 | NM_015608 | C10ORF137 | 0.45 |
| NM_016287 | HP1-BP74 | 0.45 | NM_016079 | VPS24 | 0.45 |
| NM_018086 | FIGN | 0.45 | NM_019849 | SLC7A10 | 0.45 |
| NM_019099 | LOC55924 | 0.45 | NM_019119 | PCDHB9 | 0.45 |
| NM_030785 | RSHL1 | 0.45 | NM_025078 | PQLC1 | 0.45 |
| NM_030800 | DKFZP564O1664 | 0.45 | XM_042978 | KIAA1817 | 0.45 |
| NM_080652 | MGC15397 | 0.45 | NM_207309 | UAP1L1 | 0.45 |
| NM_178452 | LOC123872 | 0.45 | NM_144635 | MGC21688 | 0.45 |
| NM_144644 | SPATA4 | 0.45 | NM_194287 | LOC145497 | 0.45 |
| NM_172004 | DCAL1 | 0.45 | NM_145080 | NSMCE1 | 0.45 |
| NM_152654 | FLJ38607 | 0.45 | NM_152688 | KHDRBS2 | 0.45 |
| XM_172929 | LOC255189 | 0.45 | NM_173827 | FLJ38991 | 0.45 |
| NM_198516 | MGC71806 | 0.45 | NM_198525 | LOC374654 | 0.45 |

| | | | | | |
|---|---|---|---|---|---|
| NM_005462 | MAGEC1 | 0.45 | XM_379214 | LOC339942 | 0.45 |
| XM_291169 | LOC340204 | 0.45 | XM_371728 | LOC389276 | 0.45 |
| XM_372180 | LOC389827 | 0.45 | XM_380104 | LOC402485 | 0.45 |
| XM_376986 | LOC401584 | 0.45 | NM_012288 | TRAM2 | 0.45 |
| NM_004721 | MAP3K13 | 0.46 | NM_004752 | GCM2 | 0.46 |
| NM_000177 | GSN | 0.46 | NM_006431 | CCT2 | 0.46 |
| NM_018848 | MKKS | 0.46 | NM_005093 | CBFA2T2 | 0.46 |
| NM_001730 | KLF5 | 0.46 | NM_018300 | ZNF83 | 0.46 |
| NM_005655 | TIEG | 0.46 | NM_000790 | DDC | 0.46 |
| NM_001218 | CA12 | 0.46 | NM_020184 | CNNM4 | 0.46 |
| NM_001228 | CASP8 | 0.46 | NM_003672 | CDC14A | 0.46 |
| NM_130771 | OSCAR | 0.46 | NM_000714 | BZRP | 0.46 |
| NM_021798 | IL21R | 0.46 | NM_020548 | DBI | 0.46 |
| NM_002517 | NPAS1 | 0.46 | NM_005122 | NR1I3 | 0.46 |
| NM_015166 | MLC1 | 0.46 | NM_005256 | GAS2 | 0.46 |
| NM_000902 | MME | 0.46 | NM_004584 | RAD9A | 0.46 |
| NM_000860 | HPGD | 0.46 | NM_007150 | ZNF185 | 0.46 |
| NM_004653 | JARID1D | 0.46 | NM_003963 | TM4SF5 | 0.46 |
| NM_014673 | KIAA0103 | 0.46 | NM_006321 | ARIH2 | 0.46 |
| NM_004263 | SEMA4F | 0.46 | XM_290471 | KIAA0261 | 0.46 |
| NM_015399 | BRMS1 | 0.46 | NM_013333 | EPN1 | 0.46 |
| NM_013433 | TNPO2 | 0.46 | NM_019028 | ZDHHC13 | 0.46 |
| NM_018997 | MRPS21 | 0.46 | NM_018479 | ECHDC1 | 0.46 |
| NM_020463 | KIAA1387 | 0.46 | NM_020957 | PCDHB16 | 0.46 |
| NM_031943 | IFP38 | 0.46 | NM_031952 | SPATA9 | 0.46 |
| NM_030960 | SPACA1 | 0.46 | NM_032882 | PNMA6A | 0.46 |
| NM_138414 | LOC112869 | 0.46 | NM_052924 | RHPN1 | 0.46 |
| NM_152301 | PP784 | 0.46 | NM_033647 | HELB | 0.46 |
| XM_059341 | LOC129293 | 0.46 | NM_153000 | APCDD1 | 0.46 |
| NM_144657 | FLJ30678 | 0.46 | NM_145052 | MGC23937 | 0.46 |
| NM_173509 | MGC16664 | 0.46 | NM_175881 | MGC48986 | 0.46 |
| NM_152760 | FLJ30934 | 0.46 | XM_373522 | LOC283314 | 0.46 |
| NM_147196 | TMIE | 0.46 | NM_173651 | FSIP2 | 0.46 |
| NM_178176 | MOGAT3 | 0.46 | XM_060880 | LOC128208 | 0.46 |
| NM_021092 | PPY2 | 0.46 | XM_291607 | LOC343505 | 0.46 |
| XM_379363 | LOC401212 | 0.46 | XM_372045 | LOC389663 | 0.46 |
| XM_377488 | LOC401891 | 0.46 | XM_374484 | LOC392742 | 0.46 |
| NM_001005405 | KRTAP5-11 | 0.46 | NM_003775 | EDG6 | 0.47 |
| NM_018492 | TOPK | 0.47 | NM_032575 | GLIS2 | 0.47 |
| NM_000107 | DDB2 | 0.47 | NM_005931 | MICB | 0.47 |
| NM_012421 | RLF | 0.47 | NM_033343 | LHX4 | 0.47 |
| NM_016571 | GLULD1 | 0.47 | NM_021033 | RAP2A | 0.47 |
| NM_004227 | PSCD3 | 0.47 | NM_013447 | EMR2 | 0.47 |
| NM_003014 | SFRP4 | 0.47 | NM_138410 | CKLFSF7 | 0.47 |
| NM_139163 | ALS2CR12 | 0.47 | NM_015431 | BIA2 | 0.47 |
| NM_003843 | SCEL | 0.47 | NM_004546 | NDUFB2 | 0.47 |

| | | | | | |
|---|---|---|---|---|---|
| NM_006944 | SPP2 | 0.47 | NM_005987 | SPRR1A | 0.47 |
| NM_012070 | ATRN | 0.47 | NM_003680 | YARS | 0.47 |
| NM_003695 | LY6D | 0.47 | NM_006333 | C1D | 0.47 |
| NM_015284 | KIAA0467 | 0.47 | NM_012450 | SLC13A4 | 0.47 |
| NM_013353 | TMOD4 | 0.47 | XM_372997 | OR5K1 | 0.47 |
| NM_015954 | CGI-26 | 0.47 | NM_013299 | HSU79266 | 0.47 |
| NM_017896 | C20ORF11 | 0.47 | XM_371822 | C6ORF110 | 0.47 |
| NM_018448 | TIP120A | 0.47 | NM_052839 | PANX2 | 0.47 |
| XM_047550 | ZNF492 | 0.47 | NM_021199 | SQRDL | 0.47 |
| NM_022095 | ZNF335 | 0.47 | NM_138415 | LOC112885 | 0.47 |
| NM_024907 | FBXO17 | 0.47 | NM_138390 | LOC92691 | 0.47 |
| NM_173476 | FLJ34512 | 0.47 | NM_181707 | LOC124773 | 0.47 |
| NM_152292 | RG9MTD2 | 0.47 | NM_153007 | ODF4 | 0.47 |
| XM_373742 | MGC40489 | 0.47 | NM_173518 | FLJ25692 | 0.47 |
| NM_181785 | LOC283537 | 0.47 | XM_376413 | DKFZP564C0469 | 0.47 |
| NM_181756 | ZNF233 | 0.47 | XM_059140 | LOC127391 | 0.47 |
| XM_172852 | LOC256676 | 0.47 | XM_377935 | LOC402253 | 0.47 |
| XM_379819 | LOC402489 | 0.47 | XM_372063 | LOC389697 | 0.47 |
| XM_372337 | LOC390020 | 0.47 | XM_372611 | LOC390680 | 0.47 |
| XM_372641 | LOC390736 | 0.47 | XM_373718 | LOC388348 | 0.47 |
| NM_001954 | DDR1 | 0.48 | NM_001315 | MAPK14 | 0.48 |
| NM_002416 | CXCL9 | 0.48 | NM_000623 | BDKRB2 | 0.48 |
| NM_018910 | PCDHA7 | 0.48 | NM_017852 | NALP2 | 0.48 |
| NM_004641 | MLLT10 | 0.48 | NM_001528 | HGFAC | 0.48 |
| NM_012410 | PSK-1 | 0.48 | NM_006153 | NCK1 | 0.48 |
| NM_003240 | EBAF | 0.48 | NM_000382 | ALDH3A2 | 0.48 |
| NM_005459 | GUCA1C | 0.48 | NM_020157 | OTOR | 0.48 |
| NM_001921 | DCTD | 0.48 | XM_038970 | SLC8A2 | 0.48 |
| NM_003262 | TLOC1 | 0.48 | NM_006524 | ZNF138 | 0.48 |
| NM_016324 | ZNF274 | 0.48 | NM_014923 | FNDC3 | 0.48 |
| XM_290559 | GRIP1 | 0.48 | NM_015420 | DKFZP564O0463 | 0.48 |
| NM_012367 | OR2B6 | 0.48 | NM_019063 | EML4 | 0.48 |
| NM_015952 | RWDD1 | 0.48 | NM_018221 | MOBK1B | 0.48 |
| NM_018060 | FLJ10326 | 0.48 | XM_290867 | RGL3 | 0.48 |
| NM_020427 | ARS | 0.48 | NM_020233 | MDS006 | 0.48 |
| NM_021571 | ICEBERG | 0.48 | NM_032656 | DHX37 | 0.48 |
| NM_024319 | C1ORF35 | 0.48 | NM_025248 | SNIP | 0.48 |
| NM_032206 | NOD27 | 0.48 | XM_050988 | MLR2 | 0.48 |
| NM_032852 | APG4C | 0.48 | NM_033127 | RGPR | 0.48 |
| NM_138376 | TTC5 | 0.48 | NM_134268 | CYGB | 0.48 |
| NM_144603 | NOXO1 | 0.48 | NM_138331 | RNASE8 | 0.48 |
| NM_173491 | LSM11 | 0.48 | NM_152402 | TRAM1L1 | 0.48 |
| NM_144653 | BTBD14A | 0.48 | NM_152487 | FLJ31842 | 0.48 |
| NM_153712 | TTL | 0.48 | NM_153230 | FBXO39 | 0.48 |
| XM_375754 | LOC163404 | 0.48 | XM_375187 | NUT | 0.48 |
| NM_175738 | RAB37 | 0.48 | XM_378923 | LOC339476 | 0.48 |

| | | |
|---|---|---|
| NM_207404 | FLJ45880 | 0.48 |
| XM_293570 | LOC344741 | 0.48 |
| XM_374131 | LOC389311 | 0.48 |
| XM_379977 | LOC402629 | 0.48 |
| XM_370849 | LOC388107 | 0.48 |
| XM_373370 | LOC392539 | 0.48 |
| NM_012145 | DTYMK | 0.49 |
| NM_006742 | PSKH1 | 0.49 |
| NM_003384 | VRK1 | 0.49 |
| NM_018934 | PCDHB14 | 0.49 |
| NM_022470 | WIG1 | 0.49 |
| NM_003677 | DENR | 0.49 |
| NM_000287 | PEX6 | 0.49 |
| NM_002585 | PBX1 | 0.49 |
| NM_003671 | CDC14B | 0.49 |
| NM_000220 | KCNJ1 | 0.49 |
| NM_173860 | HOXC12 | 0.49 |
| NM_173567 | ABHD7 | 0.49 |
| NM_005671 | D8S2298E | 0.49 |
| NM_032493 | AP1M1 | 0.49 |
| NM_016111 | KIAA0683 | 0.49 |
| NM_013377 | PDZRN4 | 0.49 |
| NM_017762 | FLJ20313 | 0.49 |
| NM_017612 | ZCCHC8 | 0.49 |
| NM_020751 | COG6 | 0.49 |
| NM_177441 | MGC3123 | 0.49 |
| NM_024780 | TMC5 | 0.49 |
| NM_133457 | EMID2 | 0.49 |
| XM_047499 | RNF187 | 0.49 |
| NM_173698 | FLJ37659 | 0.49 |
| XM_065124 | LOC126502 | 0.49 |
| XM_098317 | LOC153134 | 0.49 |
| XM_098828 | LOC157813 | 0.49 |
| XM_372302 | LOC389932 | 0.49 |
| NM_013355 | PKN3 | 0.5 |
| NM_000273 | GPR143 | 0.5 |
| NM_181882 | PRX | 0.5 |
| NM_004681 | EIF1AY | 0.5 |
| NM_005449 | TOSO | 0.5 |
| NM_000374 | UROD | 0.5 |
| NM_002660 | PLCG1 | 0.5 |
| NM_002706 | PPM1B | 0.5 |
| NM_004673 | ANGPTL1 | 0.5 |
| NM_000530 | MPZ | 0.5 |
| NM_006580 | CLDN16 | 0.5 |
| NM_000129 | F13A1 | 0.5 |
| NM_206998 | SCGB1D4 | 0.48 |
| XM_378859 | LOC400751 | 0.48 |
| XM_374141 | LOC389332 | 0.48 |
| XM_372375 | LOC390110 | 0.48 |
| XM_370722 | LOC387921 | 0.48 |
| NM_001787 | CDC2L1 | 0.49 |
| NM_001396 | DYRK1A | 0.49 |
| NM_018425 | PI4KII | 0.49 |
| NM_005201 | CCR8 | 0.49 |
| NM_002598 | PDCD2 | 0.49 |
| NM_014352 | POU2F3 | 0.49 |
| NM_001328 | CTBP1 | 0.49 |
| NM_012237 | SIRT2 | 0.49 |
| NM_022148 | CRLF2 | 0.49 |
| NM_000266 | NDP | 0.49 |
| NM_020307 | CCNL1 | 0.49 |
| NM_014576 | ACF | 0.49 |
| NM_006262 | PRPH | 0.49 |
| NM_003370 | VASP | 0.49 |
| NM_014799 | HEPH | 0.49 |
| NM_015726 | H326 | 0.49 |
| NM_016103 | SARA2 | 0.49 |
| NM_017727 | FLJ20254 | 0.49 |
| NM_019601 | SUSD2 | 0.49 |
| NM_021945 | C6ORF85 | 0.49 |
| NM_024552 | LASS4 | 0.49 |
| NM_024847 | TMC7 | 0.49 |
| NM_145269 | LOC137392 | 0.49 |
| NM_144969 | ZDHHC15 | 0.49 |
| NM_207485 | FLJ41327 | 0.49 |
| XM_293018 | LOC344332 | 0.49 |
| XM_292109 | LOC341511 | 0.49 |
| XM_373319 | LOC392395 | 0.49 |
| NM_001394 | DUSP4 | 0.5 |
| NM_002350 | LYN | 0.5 |
| NM_030903 | OR2W1 | 0.5 |
| NM_002198 | IRF1 | 0.5 |
| NM_153696 | PSMAL/GCP III | 0.5 |
| NM_003292 | TPR | 0.5 |
| NM_002883 | RANGAP1 | 0.5 |
| NM_003590 | CUL3 | 0.5 |
| NM_019846 | CCL28 | 0.5 |
| NM_003264 | TLR2 | 0.5 |
| NM_000559 | HBG1 | 0.5 |
| J05158 | CPN2 | 0.5 |
| NM_020386 | HRASLS | 0.5 |

| | | | | | |
|---|---|---|---|---|---|
| NM_007217 | PDCD10 | 0.5 | NM_002963 | S100A7 | 0.5 |
| NM_016448 | RAMP | 0.5 | NM_018117 | WDR11 | 0.5 |
| NM_021116 | ADCY1 | 0.5 | NM_001418 | EIF4G2 | 0.5 |
| NM_002532 | NUP88 | 0.5 | NM_005927 | MFAP3 | 0.5 |
| NM_005407 | SALL2 | 0.5 | NM_003539 | HIST1H4D | 0.5 |
| NM_005797 | EVA1 | 0.5 | XM_047214 | C22ORF9 | 0.5 |
| NM_144563 | RPIA | 0.5 | NM_016072 | CGI-141 | 0.5 |
| NM_016242 | EMCN | 0.5 | NM_017757 | ZNF407 | 0.5 |
| NM_018332 | FLJ11126 | 0.5 | NM_024345 | MGC10765 | 0.5 |
| NM_024835 | ZNF403 | 0.5 | NM_030780 | MFTC | 0.5 |
| XM_046911 | ZDHHC18 | 0.5 | NM_033132 | ZIC5 | 0.5 |
| NM_194277 | LOC90167 | 0.5 | NM_138450 | ARL11 | 0.5 |
| NM_178275 | DKFZP434B1231 | 0.5 | XM_371328 | DJ383J4.3 | 0.5 |
| NM_198923 | MRGPRD | 0.5 | NM_152318 | MGC40397 | 0.5 |
| NM_182502 | DKFZP686L1818 | 0.5 | XM_097736 | C20ORF82 | 0.5 |
| NM_152472 | ZNF578 | 0.5 | NM_178861 | ZNF183L1 | 0.5 |
| XM_371039 | MGC19764 | 0.5 | NM_183372 | LOC200030 | 0.5 |
| NM_175734 | LOC201243 | 0.5 | NM_174937 | TCERG1L | 0.5 |
| NM_173580 | FLJ39058 | 0.5 | NM_152772 | MGC40368 | 0.5 |
| NM_178033 | CYP4X1 | 0.5 | NM_207007 | CCL4L1 | 0.5 |
| NM_207471 | FLJ42200 | 0.5 | NM_203303 | LOC389874 | 0.5 |
| NM_207500 | FLJ44955 | 0.5 | XM_291392 | LOC343066 | 0.5 |
| XM_088797 | LOC163301 | 0.5 | XM_098117 | LOC151760 | 0.5 |
| XM_374053 | LOC389153 | 0.5 | XM_371591 | LOC389072 | 0.5 |
| XM_378453 | LOC400208 | 0.5 | NM_024619 | FN3KRP | 0.51 |
| NM_020526 | EPHA8 | 0.51 | NM_007181 | MAP4K1 | 0.51 |
| NM_001704 | BAI3 | 0.51 | NM_176881 | TAS2R39 | 0.51 |
| NM_001810 | CENPB | 0.51 | NM_002441 | MSH5 | 0.51 |
| NM_003024 | ITSN1 | 0.51 | NM_004332 | BPHL | 0.51 |
| NM_003577 | UTF1 | 0.51 | NM_000127 | EXT1 | 0.51 |
| NM_024308 | MGC4172 | 0.51 | NM_022662 | ANAPC1 | 0.51 |
| NM_012164 | FBXW2 | 0.51 | NM_001567 | INPPL1 | 0.51 |
| NM_004642 | CDK2AP1 | 0.51 | NM_002176 | IFNB1 | 0.51 |
| NM_003152 | STAT5A | 0.51 | NM_033031 | CCNB3 | 0.51 |
| XM_291057 | CLASP2 | 0.51 | NM_003025 | SH3GL1 | 0.51 |
| NM_002477 | MYL5 | 0.51 | NM_002967 | SAFB | 0.51 |
| NM_002773 | PRSS8 | 0.51 | NM_012339 | NET-7 | 0.51 |
| NM_012460 | TIMM9 | 0.51 | NM_015655 | ZNF337 | 0.51 |
| NM_013373 | ZDHHC8 | 0.51 | NM_017814 | FLJ20422 | 0.51 |
| NM_017848 | CXORF17 | 0.51 | NM_020122 | KCMF1 | 0.51 |
| NM_003776 | MRPL40 | 0.51 | NM_024623 | FLJ13491 | 0.51 |
| NM_024808 | FLJ22624 | 0.51 | NM_025160 | WDR26 | 0.51 |
| XM_054284 | H2-ALPHA | 0.51 | NM_080924 | LOC91219 | 0.51 |
| NM_133444 | ZNF526 | 0.51 | NM_152411 | DKFZP762I137 | 0.51 |
| XM_378507 | LOC145845 | 0.51 | NM_080430 | SELM | 0.51 |
| XM_378921 | LOC148756 | 0.51 | NM_207352 | CYP4V2 | 0.51 |

| | | |
|---|---|---|
| NM_207398 | FLJ38822 | 0.51 |
| XM_294802 | LOC339077 | 0.51 |
| XM_171171 | LOC255374 | 0.51 |
| XM_373080 | LOC391770 | 0.51 |
| XM_371429 | LOC388852 | 0.51 |
| NM_138293 | ATM | 0.52 |
| NM_173354 | SNF1LK | 0.52 |
| NM_020451 | SEPN1 | 0.52 |
| NM_000153 | GALC | 0.52 |
| NM_003614 | GALR3 | 0.52 |
| NM_003332 | TYROBP | 0.52 |
| NM_000901 | NR3C2 | 0.52 |
| NM_020364 | DAZ3 | 0.52 |
| NM_021176 | G6PC2 | 0.52 |
| NM_001414 | EIF2B1 | 0.52 |
| NM_004987 | LIMS1 | 0.52 |
| NM_015167 | PTDSR | 0.52 |
| NM_006160 | NEUROD2 | 0.52 |
| NM_002933 | RNASE1 | 0.52 |
| NM_005838 | GLYAT | 0.52 |
| NM_206995 | C10ORF1 | 0.52 |
| NM_014594 | ZNF354C | 0.52 |
| NM_024496 | C14ORF4 | 0.52 |
| NM_022133 | SNX16 | 0.52 |
| NM_024096 | XTP3TPA | 0.52 |
| NM_025048 | FLJ22684 | 0.52 |
| NM_178472 | C20ORF53 | 0.52 |
| NM_173512 | FLJ39822 | 0.52 |
| NM_173582 | PGM2L1 | 0.52 |
| XM_209363 | PTPNS1L3 | 0.52 |
| XM_293893 | LRG47 | 0.52 |
| NM_003171 | SUPV3L1 | 0.52 |
| XM_291154 | LOC340152 | 0.52 |
| XM_378033 | LOC402340 | 0.52 |
| XM_378567 | LOC400505 | 0.52 |
| NM_001005487 | OR13G1 | 0.52 |
| NM_020922 | PRKWNK3 | 0.53 |
| NM_000952 | PTAFR | 0.53 |
| NM_001812 | CENPC1 | 0.53 |
| NM_004058 | CAPS | 0.53 |
| NM_052838 | 37135 | 0.53 |
| NM_004079 | CTSS | 0.53 |
| NM_003252 | TIAL1 | 0.53 |
| NM_019116 | UBPH | 0.53 |
| NM_020903 | USP29 | 0.53 |
| NM_002224 | ITPR3 | 0.53 |
| NM_207412 | FLJ43582 | 0.51 |
| XM_291395 | LOC343069 | 0.51 |
| XM_301210 | LOC388775 | 0.51 |
| XM_372870 | LOC391257 | 0.51 |
| NM_003656 | CAMK1 | 0.52 |
| NM_004217 | AURKB | 0.52 |
| NM_006091 | CORO2B | 0.52 |
| NM_005957 | MTHFR | 0.52 |
| NM_001349 | DARS | 0.52 |
| NM_014875 | KIF14 | 0.52 |
| NM_003175 | XCL2 | 0.52 |
| NM_018662 | DISC1 | 0.52 |
| NM_022487 | DCLRE1C | 0.52 |
| NM_021907 | DTNB | 0.52 |
| NM_032020 | FUCA2 | 0.52 |
| NM_024747 | HPS6 | 0.52 |
| NM_002348 | LY9 | 0.52 |
| NM_014227 | SLC5A4 | 0.52 |
| XM_376917 | FUBP3 | 0.52 |
| NM_007357 | COG2 | 0.52 |
| NM_014035 | SNX24 | 0.52 |
| NM_019022 | FLJ20793 | 0.52 |
| NM_022733 | LOC64744 | 0.52 |
| NM_022894 | PAPOLG | 0.52 |
| NM_020858 | SEMA6D | 0.52 |
| NM_032937 | C9ORF37 | 0.52 |
| NM_152485 | FLJ25078 | 0.52 |
| NM_153707 | C9ORF138 | 0.52 |
| NM_153262 | SYT14 | 0.52 |
| NM_181622 | KRTAP13-3 | 0.52 |
| NM_180991 | SLCO4C1 | 0.52 |
| XM_211764 | LOC285095 | 0.52 |
| XM_212123 | LOC285995 | 0.52 |
| XM_380008 | LOC402665 | 0.52 |
| XM_373684 | LOC388274 | 0.52 |
| NM_001381 | DOK1 | 0.53 |
| NM_005027 | PIK3R2 | 0.53 |
| NM_014800 | ELMO1 | 0.53 |
| NM_012296 | GAB2 | 0.53 |
| NM_145173 | DIRAS1 | 0.53 |
| NM_005011 | NRF1 | 0.53 |
| NM_000500 | CYP21A2 | 0.53 |
| NM_003896 | SIAT9 | 0.53 |
| NM_001752 | CAT | 0.53 |
| NM_000252 | MTM1 | 0.53 |
| NM_030956 | TLR10 | 0.53 |

| | | |
|---|---|---|
| NM_018965 | TREM2 | 0.53 |
| NM_004961 | GABRE | 0.53 |
| NM_005422 | TECTA | 0.53 |
| NM_005880 | DNAJA2 | 0.53 |
| NM_014060 | MCTS1 | 0.53 |
| NM_004344 | CETN2 | 0.53 |
| NM_004544 | NDUFA10 | 0.53 |
| NM_004160 | PYY | 0.53 |
| NM_003838 | FPGT | 0.53 |
| NM_006828 | HELIC1 | 0.53 |
| NM_019049 | FLJ20054 | 0.53 |
| NM_017927 | MFN1 | 0.53 |
| NM_020356 | C20ORF32 | 0.53 |
| NM_030812 | LOC81569 | 0.53 |
| NM_032506 | KIAA1841 | 0.53 |
| NM_052897 | MBD6 | 0.53 |
| NM_152404 | FLJ34658 | 0.53 |
| NM_153337 | SLIC1 | 0.53 |
| NM_173794 | FUNDC1 | 0.53 |
| NM_139179 | LOC221955 | 0.53 |
| NM_178567 | MGC42638 | 0.53 |
| XM_294743 | LOC338918 | 0.53 |
| XM_290351 | LOC339843 | 0.53 |
| XM_373474 | LOC387710 | 0.53 |
| XM_377407 | LOC401827 | 0.53 |
| XM_373910 | LOC388791 | 0.53 |
| NM_014602 | PIK3R4 | 0.54 |
| NM_005756 | GPR64 | 0.54 |
| NM_006141 | DNCLI2 | 0.54 |
| NM_014739 | BCLAF1 | 0.54 |
| NM_004535 | MYT1 | 0.54 |
| NM_003198 | TCEB3 | 0.54 |
| NM_004893 | H2AFY | 0.54 |
| NM_003279 | TNNC2 | 0.54 |
| NM_002535 | OAS2 | 0.54 |
| NM_000429 | MAT1A | 0.54 |
| NM_004109 | FDX1 | 0.54 |
| NM_005090 | PLA2G4B | 0.54 |
| NM_017883 | WDR13 | 0.54 |
| NM_002725 | PRELP | 0.54 |
| NM_002861 | PCYT2 | 0.54 |
| NM_014878 | KIAA0020 | 0.54 |
| NM_005094 | SLC27A4 | 0.54 |
| NM_015666 | GTPBP5 | 0.54 |
| NM_015939 | CGI-09 | 0.54 |
| XM_290829 | INTERSEX | 0.54 |
| NM_000735 | CGA | 0.53 |
| NM_004237 | TRIP13 | 0.53 |
| NM_012097 | ARL5 | 0.53 |
| NM_000146 | FTL | 0.53 |
| NM_003002 | SDHD | 0.53 |
| NM_002347 | LY6H | 0.53 |
| NM_006226 | PLCL1 | 0.53 |
| NM_021089 | ZNF8 | 0.53 |
| NM_021067 | KIAA0186 | 0.53 |
| NM_014505 | KCNMB4 | 0.53 |
| XM_375684 | HES2 | 0.53 |
| NM_018712 | DKFZP547C176 | 0.53 |
| NM_021246 | LY6G6D | 0.53 |
| NM_031448 | C19ORF12 | 0.53 |
| XM_032678 | LOC90576 | 0.53 |
| NM_181435 | C1QTNF3 | 0.53 |
| NM_152288 | MGC13024 | 0.53 |
| NM_178833 | LOC133308 | 0.53 |
| NM_152731 | C6ORF65 | 0.53 |
| NM_177533 | NUDT14 | 0.53 |
| NM_207432 | FLJ45436 | 0.53 |
| XM_064879 | LOC125958 | 0.53 |
| XM_117030 | LOC197387 | 0.53 |
| XM_373527 | LOC387846 | 0.53 |
| XM_375357 | LOC400537 | 0.53 |
| NM_015148 | PASK | 0.54 |
| NM_004951 | EBI2 | 0.54 |
| NM_001777 | CD47 | 0.54 |
| NM_001944 | DSG3 | 0.54 |
| NM_001913 | CUTL1 | 0.54 |
| NM_080473 | GATA5 | 0.54 |
| NM_012252 | TFEC | 0.54 |
| NM_018441 | PECR | 0.54 |
| NM_000460 | THPO | 0.54 |
| NM_004259 | RECQL5 | 0.54 |
| NM_003636 | KCNAB2 | 0.54 |
| NM_004669 | CLIC3 | 0.54 |
| NM_006928 | SILV | 0.54 |
| NM_004436 | ENSA | 0.54 |
| NM_002523 | NPTX2 | 0.54 |
| NM_004256 | SLC22A13 | 0.54 |
| NM_006375 | COVA1 | 0.54 |
| NM_014613 | ETEA | 0.54 |
| NM_016948 | PARD6A | 0.54 |
| NM_018019 | MED25 | 0.54 |
| NM_018710 | DKFZP762O076 | 0.54 |

| | | | | | |
|---|---|---|---|---|---|
| NM_020373 | TMEM16B | 0.54 | NM_020770 | CGN | 0.54 |
| NM_022362 | MMS19L | 0.54 | NM_022786 | ARV1 | 0.54 |
| NM_024826 | FLJ21159 | 0.54 | NM_025153 | FLJ21477 | 0.54 |
| NM_030574 | STARD5 | 0.54 | NM_031431 | COG3 | 0.54 |
| NM_032756 | MGC15668 | 0.54 | NM_031911 | C1QTNF7 | 0.54 |
| NM_173478 | FLJ40137 | 0.54 | NM_152386 | SGPP2 | 0.54 |
| NM_024095 | ASB8 | 0.54 | NM_182519 | C20orf186 | 0.54 |
| NM_176814 | LOC168850 | 0.54 | NM_198989 | DLEU7 | 0.54 |
| XM_371369 | FLJ39207 | 0.54 | NM_198577 | FLJ46361 | 0.54 |
| NM_206997 | GPR152 | 0.54 | XM_293671 | LOC344988 | 0.54 |
| XM_061666 | LOC119764 | 0.54 | XM_293325 | LOC347411 | 0.54 |
| XM_208058 | LOC283247 | 0.54 | XM_352463 | LOC391408 | 0.54 |
| XM_374012 | LOC389043 | 0.54 | XM_371824 | LOC389395 | 0.54 |
| XM_372154 | LOC389803 | 0.54 | XM_372010 | LOC389607 | 0.54 |
| XM_373452 | LOC387649 | 0.54 | XM_370873 | LOC388135 | 0.54 |
| XM_370840 | LOC388085 | 0.54 | XM_372878 | LOC391282 | 0.54 |
| NM_001004067 | NOMO3 | 0.54 | NM_001654 | ARAF1 | 0.55 |
| NM_001400 | EDG1 | 0.55 | NM_000145 | FSHR | 0.55 |
| NM_005295 | GPR22 | 0.55 | NM_001257 | CDH13 | 0.55 |
| NM_001833 | CLTA | 0.55 | NM_032587 | CARD6 | 0.55 |
| NM_006149 | LGALS4 | 0.55 | NM_000192 | TBX5 | 0.55 |
| NM_003866 | INPP4B | 0.55 | NM_003366 | UQCRC2 | 0.55 |
| NM_006739 | MCM5 | 0.55 | NM_016334 | SH120 | 0.55 |
| NM_002063 | GLRA2 | 0.55 | NM_174975 | SEC14L3 | 0.55 |
| NM_000721 | CACNA1E | 0.55 | NM_000441 | SLC26A4 | 0.55 |
| NM_014937 | INPP5F | 0.55 | NM_021052 | HIST1H2AE | 0.55 |
| NM_004486 | GOLGA2 | 0.55 | NM_005561 | LAMP1 | 0.55 |
| NM_002553 | ORC5L | 0.55 | NM_173705 | MTCO2 | 0.55 |
| NM_006014 | DXS9879E | 0.55 | NM_003178 | SYN2 | 0.55 |
| NM_003598 | TEAD2 | 0.55 | NM_003654 | CHST1 | 0.55 |
| NM_005453 | ZNF297 | 0.55 | NM_004678 | BPY2 | 0.55 |
| NM_005773 | ZNF256 | 0.55 | XM_048592 | KIAA1045 | 0.55 |
| NM_013321 | SNX8 | 0.55 | NM_014595 | NT5C | 0.55 |
| NM_014863 | GALNAC4S-6ST | 0.55 | NM_018472 | HT011 | 0.55 |
| NM_020698 | KIAA1145 | 0.55 | NM_023010 | UPF3B | 0.55 |
| NM_032149 | DKFZP434G072 | 0.55 | NM_031947 | SLC25A2 | 0.55 |
| NM_138409 | C6ORF117 | 0.55 | NM_033315 | RASL10B | 0.55 |
| NM_054112 | DEFB118 | 0.55 | NM_173791 | PDZK8 | 0.55 |
| NM_152361 | FLJ38944 | 0.55 | XM_371312 | FLJ39117 | 0.55 |
| NM_131915 | LOC150236 | 0.55 | NM_194289 | LOC152195 | 0.55 |
| NM_152618 | FLJ35630 | 0.55 | NM_152621 | MGC26963 | 0.55 |
| NM_017629 | EIF2C4 | 0.55 | XM_113776 | LOC196913 | 0.55 |
| NM_170745 | HIST1H2AA | 0.55 | NM_199282 | LOC201176 | 0.55 |
| NM_198081 | SCML4 | 0.55 | NM_174939 | MGC39681 | 0.55 |
| XM_170736 | SLC25A30 | 0.55 | NM_198479 | FLJ40321 | 0.55 |
| XM_209163 | LOC284379 | 0.55 | NM_173620 | FLJ23825 | 0.55 |

| | | |
|---|---|---|
| XM_378371 | LOC338758 | 0.55 |
| XM_291170 | LOC340192 | 0.55 |
| XM_374045 | LOC389139 | 0.55 |
| XM_371694 | LOC389207 | 0.55 |
| XM_379995 | LOC402641 | 0.55 |
| XM_372443 | LOC390275 | 0.55 |
| XM_375148 | LOC400299 | 0.55 |
| NM_005228 | EGFR | 0.56 |
| NM_006449 | CDC42EP3 | 0.56 |
| NM_018046 | VG5Q | 0.56 |
| NM_013340 | PCDHB1 | 0.56 |
| NM_001987 | ETV6 | 0.56 |
| NM_014585 | SLC40A1 | 0.56 |
| NM_003107 | SOX4 | 0.56 |
| NM_138432 | SDSL | 0.56 |
| NM_001874 | CPM | 0.56 |
| NM_025210 | I-4 | 0.56 |
| NM_004393 | DAG1 | 0.56 |
| NM_000453 | SLC5A5 | 0.56 |
| NM_018456 | EAF2 | 0.56 |
| NM_006158 | NEFL | 0.56 |
| NM_015087 | SPG20 | 0.56 |
| NM_004569 | PIGH | 0.56 |
| NM_032501 | ACAS2L | 0.56 |
| NM_003537 | HIST1H3B | 0.56 |
| NM_006775 | QKI | 0.56 |
| NM_012266 | DNAJB5 | 0.56 |
| NM_016298 | FBXO40 | 0.56 |
| NM_024018 | BTN2A3 | 0.56 |
| NM_018369 | DEPDC1B | 0.56 |
| NM_022097 | LOC63928 | 0.56 |
| NM_024072 | DDX54 | 0.56 |
| XM_290972 | FLJ12747 | 0.56 |
| NM_053053 | STAF42 | 0.56 |
| XM_378763 | LOC284240 | 0.56 |
| XM_371132 | FLJ38144 | 0.56 |
| XM_294775 | LOC339022 | 0.56 |
| XM_208203 | LOC284428 | 0.56 |
| XM_114621 | LOC203076 | 0.56 |
| XM_376150 | LOC401016 | 0.56 |
| XM_374190 | LOC389442 | 0.56 |
| XM_373606 | LOC388011 | 0.56 |
| XM_373628 | LOC388123 | 0.56 |
| XM_372255 | LOC389895 | 0.56 |
| NM_002765 | PRPS2 | 0.57 |
| NM_003483 | HMGA2 | 0.57 |
| XM_211088 | LOC283604 | 0.55 |
| XM_294590 | LOC347265 | 0.55 |
| XM_373061 | LOC391749 | 0.55 |
| XM_374273 | LOC389669 | 0.55 |
| XM_372509 | LOC390445 | 0.55 |
| XM_373503 | LOC387777 | 0.55 |
| XM_379704 | LOC401588 | 0.55 |
| NM_002221 | ITPKB | 0.56 |
| NM_002149 | HPCAL1 | 0.56 |
| NM_018908 | PCDHA5 | 0.56 |
| NM_007115 | TNFAIP6 | 0.56 |
| NM_003643 | GCM1 | 0.56 |
| NM_000438 | PAX3 | 0.56 |
| NM_016243 | NQO3A2 | 0.56 |
| NM_001734 | C1S | 0.56 |
| NM_000929 | PLA2G5 | 0.56 |
| NM_021268 | IFNA17 | 0.56 |
| NM_015049 | ALS2CR3 | 0.56 |
| NM_016567 | BCCIP | 0.56 |
| NM_002038 | G1P3 | 0.56 |
| NM_018842 | LOC55971 | 0.56 |
| NM_000728 | CALCB | 0.56 |
| NM_002437 | MPV17 | 0.56 |
| NM_005061 | RPL3L | 0.56 |
| NM_004189 | SOX14 | 0.56 |
| NM_016106 | SCFD1 | 0.56 |
| NM_019051 | MRPL50 | 0.56 |
| NM_019087 | ARFRP2 | 0.56 |
| NM_018198 | DNAJC11 | 0.56 |
| NM_020631 | KIAA0720 | 0.56 |
| NM_024036 | LRFN4 | 0.56 |
| NM_024669 | FLJ11795 | 0.56 |
| NM_052972 | LRG1 | 0.56 |
| NM_178507 | NS5ATP13TP2 | 0.56 |
| NM_181686 | KRTAP12-1 | 0.56 |
| XM_211089 | LOC283586 | 0.56 |
| XM_173063 | LOC253662 | 0.56 |
| XM_071201 | LOC138972 | 0.56 |
| XM_114987 | LOC196120 | 0.56 |
| XM_379467 | LOC401296 | 0.56 |
| XM_380072 | LOC402716 | 0.56 |
| XM_373547 | LOC387885 | 0.56 |
| XM_378751 | LOC400654 | 0.56 |
| NM_001433 | ERN1 | 0.57 |
| NM_005279 | GPR1 | 0.57 |
| NM_031437 | RASSF5 | 0.57 |

| | | | | | |
|---|---|---|---|---|---|
| NM_002499 | NEO1 | 0.57 | NM_004723 | ARHGEF2 | 0.57 |
| NM_014409 | TAF5L | 0.57 | NM_001871 | CPB1 | 0.57 |
| NM_000204 | IF | 0.57 | NM_001308 | CPN1 | 0.57 |
| NM_002713 | PPP1R8 | 0.57 | NM_005474 | HDAC5 | 0.57 |
| NM_031457 | MS4A8B | 0.57 | NM_002616 | PER1 | 0.57 |
| NM_133445 | GRIN3A | 0.57 | NM_002204 | ITGA3 | 0.57 |
| NM_001562 | IL18 | 0.57 | NM_001858 | COL19A1 | 0.57 |
| NM_001738 | CA1 | 0.57 | NM_006208 | ENPP1 | 0.57 |
| NM_001550 | IFRD1 | 0.57 | NM_013376 | SERTAD1 | 0.57 |
| NM_012103 | AUP1 | 0.57 | NM_005382 | NEF3 | 0.57 |
| NM_031888 | PMCHL2 | 0.57 | NM_005089 | U2AF1L2 | 0.57 |
| NM_003566 | EEA1 | 0.57 | NM_005729 | PPIF | 0.57 |
| NM_005804 | DDX39 | 0.57 | NM_007067 | MYST2 | 0.57 |
| NM_007175 | C8ORF2 | 0.57 | NM_014300 | SPC18 | 0.57 |
| XM_085127 | PLEKHG3 | 0.57 | NM_020843 | ZNF291 | 0.57 |
| NM_016589 | C3ORF1 | 0.57 | NM_017699 | FLJ20174 | 0.57 |
| NM_018361 | LPAAT-E | 0.57 | NM_017864 | FLJ20530 | 0.57 |
| NM_018706 | DHTKD1 | 0.57 | NM_020338 | RAI17 | 0.57 |
| NM_198066 | GNPNAT1 | 0.57 | NM_025187 | LIN10 | 0.57 |
| NM_032194 | BXDC1 | 0.57 | NM_032031 | FKSG17 | 0.57 |
| NM_032411 | ECRG4 | 0.57 | NM_032606 | CAPS2 | 0.57 |
| NM_032829 | FLJ14721 | 0.57 | NM_032517 | LYZL1 | 0.57 |
| XM_370765 | PAPLN | 0.57 | NM_138375 | CABLES1 | 0.57 |
| NM_138401 | LOC93343 | 0.57 | XM_378780 | LOC126536 | 0.57 |
| NM_152454 | FLJ31461 | 0.57 | NM_198990 | NAPE-PLD | 0.57 |
| XM_171060 | ZNF620 | 0.57 | NM_173083 | TGS | 0.57 |
| NM_182609 | MGC48625 | 0.57 | NM_199175 | C21ORF123 | 0.57 |
| NM_207506 | FLJ39458 | 0.57 | XM_171490 | LOC256148 | 0.57 |
| XM_291770 | LOC340913 | 0.57 | XM_045705 | ATXN3L | 0.57 |
| XM_378898 | LOC400782 | 0.57 | XM_372953 | LOC391428 | 0.57 |
| XM_379306 | LOC401169 | 0.57 | XM_377296 | LOC401732 | 0.57 |
| XM_378795 | LOC400685 | 0.57 | NM_000681 | ADRA2A | 0.58 |
| NM_000863 | HTR1B | 0.58 | NM_001623 | AIF1 | 0.58 |
| NM_005570 | LMAN1 | 0.58 | NM_018952 | HOXB6 | 0.58 |
| NM_022569 | NDST4 | 0.58 | NM_002435 | MPI | 0.58 |
| NM_002617 | PEX10 | 0.58 | NM_000230 | LEP | 0.58 |
| NM_005121 | THRAP1 | 0.58 | NM_003289 | TPM2 | 0.58 |
| NM_001039 | SCNN1G | 0.58 | NM_004849 | APG5L | 0.58 |
| NM_005900 | SMAD1 | 0.58 | NM_032545 | CFC1 | 0.58 |
| NM_024318 | ILT8 | 0.58 | NM_152891 | EOS | 0.58 |
| NM_016534 | FLJ39616 | 0.58 | NM_000226 | KRT9 | 0.58 |
| XM_370880 | MESDC2 | 0.58 | NM_000862 | HSD3B1 | 0.58 |
| NM_014363 | SACS | 0.58 | NM_014483 | RBMS3 | 0.58 |
| NM_005172 | ATOH1 | 0.58 | NM_002113 | HFL1 | 0.58 |
| NM_174934 | SCN4B | 0.58 | NM_003741 | CHRD | 0.58 |
| XM_374983 | KIAA0748 | 0.58 | NM_006587 | CORIN | 0.58 |

| | | |
|---|---|---|
| NM_007225 | NXPH3 | 0.58 |
| XM_370931 | OR1F2P | 0.58 |
| NM_024707 | GEMIN7 | 0.58 |
| NM_024778 | RNF127 | 0.58 |
| NM_032800 | FLJ14525 | 0.58 |
| XM_376763 | COE2 | 0.58 |
| NM_153209 | FLJ37300 | 0.58 |
| NM_152482 | C19ORF25 | 0.58 |
| XM_378355 | LOC283332 | 0.58 |
| XM_088726 | LOC158957 | 0.58 |
| XM_372922 | LOC391359 | 0.58 |
| XM_378678 | LOC400600 | 0.58 |
| XM_372607 | LOC390667 | 0.58 |
| XM_371416 | LOC388828 | 0.58 |
| NM_005400 | PRKCE | 0.59 |
| NM_005064 | CCL23 | 0.59 |
| NM_138448 | ACYP2 | 0.59 |
| NM_014970 | KIFAP3 | 0.59 |
| NM_145295 | ZNF627 | 0.59 |
| NM_001417 | EIF4B | 0.59 |
| NM_005139 | ANXA3 | 0.59 |
| NM_003256 | TIMP4 | 0.59 |
| NM_004558 | NRTN | 0.59 |
| NM_003664 | AP3B1 | 0.59 |
| NM_030943 | AMN | 0.59 |
| NM_144672 | OTOA | 0.59 |
| NM_001130 | AES | 0.59 |
| NM_030893 | CD1E | 0.59 |
| NM_003438 | ZNF137 | 0.59 |
| NM_007156 | ZXDA | 0.59 |
| NM_007056 | SFRS16 | 0.59 |
| NM_013283 | MAT2B | 0.59 |
| XM_371590 | KIAA1571 | 0.59 |
| NM_024042 | C16ORF23 | 0.59 |
| NM_024803 | TUBAL3 | 0.59 |
| NM_032356 | MGC14151 | 0.59 |
| NM_138383 | LOC92154 | 0.59 |
| NM_153046 | TDRD9 | 0.59 |
| NM_152435 | MGC35366 | 0.59 |
| NM_139167 | SGCZ | 0.59 |
| NM_014261 | TRIF | 0.59 |
| NM_178823 | C6ORF165 | 0.59 |
| XM_054313 | LOC155100 | 0.59 |
| XM_376631 | WBSCR24 | 0.59 |
| XM_290597 | LOC283464 | 0.59 |
| XM_378791 | LOC339316 | 0.59 |
| NM_007028 | TRIM31 | 0.58 |
| NM_024705 | FLJ13639 | 0.58 |
| NM_024657 | ZCWCC2 | 0.58 |
| NM_024320 | MGC11242 | 0.58 |
| NM_032357 | MGC12981 | 0.58 |
| NM_080820 | HARS2 | 0.58 |
| XM_375838 | LOC128387 | 0.58 |
| NM_153695 | ZNF367 | 0.58 |
| NM_173820 | FLJ35487 | 0.58 |
| XM_066859 | LOC139735 | 0.58 |
| XM_373596 | LOC387994 | 0.58 |
| XM_375664 | LOC400721 | 0.58 |
| XM_373353 | LOC392486 | 0.58 |
| NM_001222 | CAMK2G | 0.59 |
| NM_024531 | FLJ11856 | 0.59 |
| NM_005306 | GPR43 | 0.59 |
| NM_005130 | HBP17 | 0.59 |
| NM_018936 | PCDHB2 | 0.59 |
| NM_003426 | ZNF74 | 0.59 |
| NM_006330 | LYPLA1 | 0.59 |
| NM_000492 | CFTR | 0.59 |
| NM_004715 | CTDP1 | 0.59 |
| NM_017934 | PHIP | 0.59 |
| NM_007163 | SLC14A2 | 0.59 |
| NM_002310 | LIFR | 0.59 |
| NM_006396 | SSSCA1 | 0.59 |
| NM_014205 | C11ORF5 | 0.59 |
| NM_005217 | DEFA3 | 0.59 |
| NM_003147 | SSX2 | 0.59 |
| NM_004733 | SLC33A1 | 0.59 |
| NM_007282 | RNF13 | 0.59 |
| NM_018478 | C20ORF35 | 0.59 |
| NM_021828 | HPSE2 | 0.59 |
| NM_024630 | ZDHHC14 | 0.59 |
| NM_031292 | DKFZP434G1415 | 0.59 |
| NM_033543 | R29124_1 | 0.59 |
| NM_153206 | AMICA | 0.59 |
| XM_070277 | OC90 | 0.59 |
| NM_080676 | C20ORF133 | 0.59 |
| NM_080828 | C20ORF173 | 0.59 |
| NM_152484 | ZNF569 | 0.59 |
| XM_379183 | LOC152024 | 0.59 |
| NM_152559 | WBSCR27 | 0.59 |
| NM_178495 | KIAA1754L | 0.59 |
| XM_209579 | LOC285346 | 0.59 |
| NM_203407 | LOC340602 | 0.59 |

| | | | | | |
|---|---|---|---|---|---|
| NM_181789 | COLM | 0.59 | NM_178536 | LCN12 | 0.59 |
| NM_198562 | FLJ43654 | 0.59 | NM_203393 | LOC389458 | 0.59 |
| NM_015680 | C2ORF24 | 0.59 | XM_292963 | LOC344178 | 0.59 |
| XM_291989 | LOC338756 | 0.59 | XM_373093 | LOC391809 | 0.59 |
| XM_372013 | LOC389611 | 0.59 | XM_378643 | LOC400568 | 0.59 |
| NM_001106 | ACVR2B | 0.6 | NM_001123 | ADK | 0.6 |
| NM_004304 | ALK | 0.6 | NM_003936 | CDK5R2 | 0.6 |
| NM_000858 | GUK1 | 0.6 | NM_024927 | FLJ21019 | 0.6 |
| NM_003736 | PCDHGB4 | 0.6 | NM_004684 | SPARCL1 | 0.6 |
| NM_005924 | MEOX2 | 0.6 | NM_000017 | ACADS | 0.6 |
| NM_003756 | EIF3S3 | 0.6 | NM_000411 | HLCS | 0.6 |
| NM_000498 | CYP11B2 | 0.6 | NM_002876 | RAD51C | 0.6 |
| NM_004088 | DNTT | 0.6 | NM_006506 | RASA2 | 0.6 |
| NM_005648 | TCEB1 | 0.6 | NM_000829 | GRIA4 | 0.6 |
| NM_004115 | FGF14 | 0.6 | NM_006536 | CLCA2 | 0.6 |
| NM_004556 | NFKBIE | 0.6 | NM_020403 | PCDH9 | 0.6 |
| NM_005058 | RBMY1A1 | 0.6 | NM_000613 | HPX | 0.6 |
| NM_002056 | GFPT1 | 0.6 | NM_006200 | PCSK5 | 0.6 |
| NM_138468 | ALS2CR15 | 0.6 | NM_003863 | DPM2 | 0.6 |
| NM_014701 | KIAA0256 | 0.6 | NM_013384 | LASS2 | 0.6 |
| NM_014283 | C1ORF9 | 0.6 | NM_018184 | ARL10C | 0.6 |
| NM_018199 | C14ORF114 | 0.6 | NM_018639 | WSB2 | 0.6 |
| NM_019596 | C21ORF62 | 0.6 | NM_018922 | PCDHGB1 | 0.6 |
| NM_021238 | C12ORF14 | 0.6 | NM_022837 | FLJ22833 | 0.6 |
| NM_024626 | B7-H4 | 0.6 | NM_024579 | FLJ23221 | 0.6 |
| NM_023938 | SARG | 0.6 | NM_030895 | FLJ14129 | 0.6 |
| NM_025235 | TNKS2 | 0.6 | XM_088376 | C8ORF7 | 0.6 |
| NM_032730 | RTN4IP1 | 0.6 | NM_033109 | PNPT1 | 0.6 |
| NM_138447 | LOC115509 | 0.6 | NM_138386 | LOC92345 | 0.6 |
| NM_199459 | C10ORF71 | 0.6 | NM_178451 | ZMYND17 | 0.6 |
| NM_144671 | FLJ32356 | 0.6 | NM_152443 | RDH12 | 0.6 |
| NM_144665 | SESN3 | 0.6 | NM_138819 | LOC159091 | 0.6 |
| NM_152890 | COL24A1 | 0.6 | NM_152773 | MGC33212 | 0.6 |
| XM_378455 | LOC283551 | 0.6 | XM_376727 | LOC285888 | 0.6 |
| XM_291339 | SATL1 | 0.6 | XM_291394 | LOC343068 | 0.6 |
| NM_198566 | FLJ32363 | 0.6 | XM_088677 | LOC158796 | 0.6 |
| XM_294353 | LOC340344 | 0.6 | XM_379112 | LOC400988 | 0.6 |
| XM_371697 | LOC389211 | 0.6 | XM_373224 | LOC392164 | 0.6 |
| XM_376602 | LOC401331 | 0.6 | XM_379395 | LOC401234 | 0.6 |
| XM_378653 | LOC400577 | 0.6 | NM_001004327 | FLJ42258 | 0.6 |
| XM_377033 | LOC401611 | 0.6 | NM_001005222 | OR52A4 | 0.6 |
| XM_037430 | DUSP7 | 0.61 | NM_003821 | RIPK2 | 0.61 |
| NM_002073 | GNAZ | 0.61 | NM_057163 | GNRHR2 | 0.61 |
| NM_000861 | HRH1 | 0.61 | NM_000870 | HTR4 | 0.61 |
| NM_012402 | ARFIP2 | 0.61 | NM_004281 | BAG3 | 0.61 |
| NM_004348 | RUNX2 | 0.61 | NM_016246 | DHRS10 | 0.61 |

| | | |
|---|---|---|
| NM_003372 | VBP1 | 0.61 |
| NM_032019 | HDAC10 | 0.61 |
| NM_000238 | KCNH2 | 0.61 |
| NM_000207 | INS | 0.61 |
| NM_005549 | KCNA10 | 0.61 |
| NM_033028 | BBS4 | 0.61 |
| NM_005267 | GJA8 | 0.61 |
| NM_019035 | PCDH18 | 0.61 |
| NM_005366 | MAGEA11 | 0.61 |
| NM_033046 | RTKN | 0.61 |
| XM_375469 | PROSAPIP2 | 0.61 |
| NM_007214 | SEC63 | 0.61 |
| NM_015484 | P29 | 0.61 |
| NM_014547 | TMOD3 | 0.61 |
| NM_016495 | TBC1D7 | 0.61 |
| NM_017755 | FLJ20303 | 0.61 |
| NM_018166 | FLJ10647 | 0.61 |
| NM_019103 | LOC55954 | 0.61 |
| NM_020134 | DPYSL5 | 0.61 |
| NM_024897 | PAQR6 | 0.61 |
| NM_052955 | TGM7 | 0.61 |
| NM_145255 | MRPL10 | 0.61 |
| NM_205545 | UNQ430 | 0.61 |
| NM_152719 | NURIT | 0.61 |
| NM_173664 | ARL10A | 0.61 |
| XM_378824 | LOC400729 | 0.61 |
| XM_376681 | LOC401409 | 0.61 |
| XM_375081 | LOC400214 | 0.61 |
| XM_372366 | LOC390078 | 0.61 |
| NM_001004325 | KRTAP5-2 | 0.61 |
| NM_006296 | VRK2 | 0.62 |
| NM_002929 | GRK1 | 0.62 |
| NM_020369 | FSCN3 | 0.62 |
| NM_003193 | TBCE | 0.62 |
| NM_017999 | RNF31 | 0.62 |
| NM_000589 | IL4 | 0.62 |
| NM_054020 | CATSPER2 | 0.62 |
| NM_000086 | CLN3 | 0.62 |
| NM_002496 | NDUFS8 | 0.62 |
| NM_001548 | IFIT1 | 0.62 |
| NM_013435 | RAX | 0.62 |
| NM_001212 | C1QBP | 0.62 |
| NM_004211 | SLC6A5 | 0.62 |
| NM_015113 | ZZEF1 | 0.62 |
| NM_015407 | DKFZP564O243 | 0.62 |
| NM_013369 | DNMT3L | 0.62 |
| NM_032525 | MGC4083 | 0.61 |
| NM_020816 | KIF17 | 0.61 |
| NM_020525 | IL22 | 0.61 |
| NM_001939 | DRP2 | 0.61 |
| NM_003759 | SLC4A4 | 0.61 |
| NM_004331 | BNIP3L | 0.61 |
| NM_006117 | PECI | 0.61 |
| NM_002962 | S100A5 | 0.61 |
| NM_002666 | PLIN | 0.61 |
| NM_021920 | SCT | 0.61 |
| NM_014759 | PHYHIP | 0.61 |
| NM_012216 | MID2 | 0.61 |
| NM_014044 | UNC50 | 0.61 |
| NM_022046 | KLK14 | 0.61 |
| NM_014601 | EHD2 | 0.61 |
| NM_018008 | FEZL | 0.61 |
| NM_018470 | C10ORF110 | 0.61 |
| NM_020424 | LOC57149 | 0.61 |
| NM_020860 | STIM2 | 0.61 |
| NM_031905 | SVH | 0.61 |
| NM_153697 | LOC91526 | 0.61 |
| NM_138805 | FAM3D | 0.61 |
| NM_183387 | EML5 | 0.61 |
| NM_153271 | MGC32065 | 0.61 |
| XM_291947 | LOC341208 | 0.61 |
| XM_373984 | LOC388947 | 0.61 |
| XM_379255 | LOC401119 | 0.61 |
| XM_374352 | LOC390367 | 0.61 |
| XM_370728 | LOC387933 | 0.61 |
| NM_030760 | EDG8 | 0.62 |
| NM_000579 | CCR5 | 0.62 |
| NM_004108 | FCN2 | 0.62 |
| NM_018200 | HMG20A | 0.62 |
| NM_032828 | ZNF587 | 0.62 |
| NM_020429 | SMURF1 | 0.62 |
| NM_000288 | PEX7 | 0.62 |
| NM_053054 | CATSPER1 | 0.62 |
| XM_370908 | MGC14386 | 0.62 |
| NM_003806 | HRK | 0.62 |
| NM_002868 | RAB5B | 0.62 |
| NM_007235 | XPOT | 0.62 |
| NM_020995 | HPR | 0.62 |
| NM_006461 | SPAG5 | 0.62 |
| NM_015528 | RNF167 | 0.62 |
| NM_015411 | SUMF2 | 0.62 |
| NM_016545 | IER5 | 0.62 |

| | | | | | |
|---|---|---|---|---|---|
| NM_018069 | FLJ10352 | 0.62 | NM_018077 | RBM28 | 0.62 |
| NM_017803 | FLJ20399 | 0.62 | NM_017682 | VMD2L1 | 0.62 |
| NM_020367 | PARP11 | 0.62 | NM_020836 | KIAA1446 | 0.62 |
| NM_024667 | FLJ12750 | 0.62 | NM_025009 | FLJ13621 | 0.62 |
| NM_052842 | BCL2L12 | 0.62 | NM_033069 | C6ORF114 | 0.62 |
| XM_370967 | LOC124411 | 0.62 | NM_152376 | UBXD3 | 0.62 |
| NM_203463 | LASS6 | 0.62 | XM_378756 | LOC284274 | 0.62 |
| NM_181655 | LOC284018 | 0.62 | NM_198483 | FLJ46536 | 0.62 |
| NM_173362 | LOC317671 | 0.62 | NM_178562 | MGC50844 | 0.62 |
| NM_153614 | TSARG6 | 0.62 | XM_098163 | LOC152118 | 0.62 |
| XM_372842 | LOC391211 | 0.62 | XM_372755 | LOC390988 | 0.62 |
| XM_373876 | LOC388716 | 0.62 | XM_376233 | LOC401067 | 0.62 |
| XM_374010 | LOC389033 | 0.62 | XM_373611 | LOC388019 | 0.62 |
| XM_372553 | LOC390547 | 0.62 | XM_373811 | LOC388563 | 0.62 |
| XM_372719 | LOC390906 | 0.62 | XM_378982 | LOC400847 | 0.62 |
| NM_001005277 | OR4F16 | 0.62 | NM_003900 | SQSTM1 | 0.63 |
| NM_001981 | EPS15 | 0.63 | NM_014321 | ORC6L | 0.63 |
| NM_020831 | MKL1 | 0.63 | NM_000249 | MLH1 | 0.63 |
| NM_021168 | RAB40C | 0.63 | NM_003881 | WISP2 | 0.63 |
| NM_006618 | JARID1B | 0.63 | NM_022357 | DPEP3 | 0.63 |
| NM_025098 | MOGAT2 | 0.63 | U25801 | TAX1BP2 | 0.63 |
| NM_003213 | TEAD4 | 0.63 | NM_002866 | RAB3A | 0.63 |
| NM_014495 | ANGPTL3 | 0.63 | NM_004591 | CCL20 | 0.63 |
| NM_000415 | IAPP | 0.63 | NM_002674 | PMCH | 0.63 |
| NM_001282 | AP2B1 | 0.63 | NM_004924 | ACTN4 | 0.63 |
| NM_000452 | SLC10A2 | 0.63 | NM_014379 | KCNV1 | 0.63 |
| NM_139056 | ADAMTS16 | 0.63 | NM_000039 | APOA1 | 0.63 |
| NM_002518 | NPAS2 | 0.63 | NM_021034 | IFITM3 | 0.63 |
| NM_003932 | ST13 | 0.63 | NM_004578 | RAB4A | 0.63 |
| NM_152323 | SPIC | 0.63 | NM_004342 | CALD1 | 0.63 |
| NM_006929 | SKIV2L | 0.63 | NM_013250 | ZNF215 | 0.63 |
| NM_006778 | TRIM10 | 0.63 | XM_027105 | KIAA0767 | 0.63 |
| XM_039828 | DNAJC13 | 0.63 | NM_016071 | MRPS33 | 0.63 |
| NM_024935 | KIAA1772 | 0.63 | NM_032865 | CTEN | 0.63 |
| NM_032374 | C14ORF153 | 0.63 | XM_030445 | C10ORF75 | 0.63 |
| NM_144641 | FLJ32332 | 0.63 | NM_152464 | MGC45714 | 0.63 |
| XM_379456 | LOC154222 | 0.63 | NM_152524 | SGOL2 | 0.63 |
| NM_152705 | MGC9850 | 0.63 | NM_145007 | NALP11 | 0.63 |
| NM_178161 | PTF1A | 0.63 | XM_375185 | FMN | 0.63 |
| NM_198584 | CA13 | 0.63 | NM_182621 | MGC52498 | 0.63 |
| XM_293177 | LOC343717 | 0.63 | XM_373850 | LOC388644 | 0.63 |
| XM_376072 | LOC400965 | 0.63 | XM_374038 | LOC389108 | 0.63 |
| XM_379141 | LOC401014 | 0.63 | XM_376888 | LOC401537 | 0.63 |
| XM_374766 | LOC399715 | 0.63 | XM_376010 | LOC400927 | 0.63 |
| NM_032037 | SSTK | 0.64 | NM_000907 | NPR2 | 0.64 |
| NM_000868 | HTR2C | 0.64 | NM_030959 | OR12D3 | 0.64 |

| | | | | | |
|---|---|---|---|---|---|
| NM_003888 | ALDH1A2 | 0.64 | NM_012398 | PIP5K1C | 0.64 |
| NM_002046 | GAPDH | 0.64 | NM_000133 | F9 | 0.64 |
| NM_015458 | MTMR9 | 0.64 | NM_015367 | BCL2L13 | 0.64 |
| NM_004590 | CCL16 | 0.64 | NM_006428 | MRPL28 | 0.64 |
| NM_000394 | CRYAA | 0.64 | NM_022458 | C7ORF2 | 0.64 |
| NM_004352 | CBLN1 | 0.64 | NM_019098 | CNGB3 | 0.64 |
| NM_024908 | FLJ12973 | 0.64 | NM_000117 | EMD | 0.64 |
| NM_004719 | SFRS2IP | 0.64 | NM_002412 | MGMT | 0.64 |
| NM_006275 | SFRS6 | 0.64 | NM_006766 | MYST3 | 0.64 |
| NM_013398 | ZNF224 | 0.64 | NM_004922 | SEC24C | 0.64 |
| NM_005836 | HRSP12 | 0.64 | NM_014837 | C1ORF16 | 0.64 |
| NM_014253 | ODZ1 | 0.64 | NM_006345 | SLC30A9 | 0.64 |
| NM_006649 | UTP14A | 0.64 | NM_145693 | LPIN1 | 0.64 |
| NM_015078 | MCF2L2 | 0.64 | NM_015391 | ANAPC13 | 0.64 |
| NM_015638 | TRPC4AP | 0.64 | NM_014405 | CACNG4 | 0.64 |
| XM_290482 | FLJ10824 | 0.64 | NM_019120 | PCDHB8 | 0.64 |
| NM_022351 | EFCBP1 | 0.64 | NM_022087 | GALNT11 | 0.64 |
| NM_023008 | FLJ12949 | 0.64 | NM_022166 | XYLT1 | 0.64 |
| NM_024751 | FLJ13273 | 0.64 | NM_024572 | GALNT14 | 0.64 |
| NM_025145 | C10ORF79 | 0.64 | NM_025204 | PP2447 | 0.64 |
| NM_030980 | FLJ12671 | 0.64 | NM_032298 | SYT3 | 0.64 |
| NM_033117 | RBM18 | 0.64 | NM_033318 | LOC91689 | 0.64 |
| NM_145256 | LRRC25 | 0.64 | NM_144628 | C20ORF140 | 0.64 |
| NM_139171 | STARD6 | 0.64 | NM_152605 | FLJ37549 | 0.64 |
| NM_145019 | FLJ30707 | 0.64 | NM_198097 | C7ORF28B | 0.64 |
| NM_178543 | ENPP7 | 0.64 | NM_203419 | LOC286016 | 0.64 |
| XM_045290 | LOC151579 | 0.64 | XM_059399 | LOC130106 | 0.64 |
| XM_212326 | LOC286478 | 0.64 | XM_371496 | LOC388955 | 0.64 |
| XM_379268 | LOC401135 | 0.64 | XM_378413 | LOC400128 | 0.64 |
| XM_371070 | LOC388403 | 0.64 | XM_371430 | LOC388853 | 0.64 |
| NM_025052 | FLJ23074 | 0.65 | NM_000269 | NME1 | 0.65 |
| NM_003804 | RIPK1 | 0.65 | NM_004066 | CETN1 | 0.65 |
| NM_052876 | BTBD14B | 0.65 | NM_001552 | IGFBP4 | 0.65 |
| NM_005197 | CHES1 | 0.65 | NM_003593 | FOXN1 | 0.65 |
| NM_023001 | ARID4A | 0.65 | NM_007255 | B4GALT7 | 0.65 |
| NM_001091 | ABP1 | 0.65 | NM_007005 | TLE4 | 0.65 |
| NM_000483 | APOC2 | 0.65 | NM_003253 | TIAM1 | 0.65 |
| NM_052932 | PORIMIN | 0.65 | NM_002558 | P2RX1 | 0.65 |
| XM_043106 | KCNS2 | 0.65 | NM_001849 | COL6A2 | 0.65 |
| NM_000386 | BLMH | 0.65 | NM_001831 | CLU | 0.65 |
| NM_018291 | FLJ10986 | 0.65 | NM_014997 | KIAA0265 | 0.65 |
| NM_004319 | ASTN | 0.65 | NM_005365 | MAGEA9 | 0.65 |
| NM_002785 | PSG11 | 0.65 | NM_014228 | SLC6A7 | 0.65 |
| NM_001008 | RPS4Y1 | 0.65 | NM_005051 | QARS | 0.65 |
| NM_001879 | MASP1 | 0.65 | NM_003098 | SNTA1 | 0.65 |
| NM_003747 | TNKS | 0.65 | NM_014819 | PJA2 | 0.65 |

| | | | | | |
|---|---|---|---|---|---|
| XM_048786 | FSTL4 | 0.65 | NM_012337 | NESG1 | 0.65 |
| NM_014450 | SIT | 0.65 | NM_025238 | BTBD1 | 0.65 |
| NM_017887 | FLJ20580 | 0.65 | NM_018143 | KLHL11 | 0.65 |
| NM_018290 | PGM2 | 0.65 | NM_021178 | CCNB1IP1 | 0.65 |
| NM_032547 | SCOC | 0.65 | NM_021939 | FKBP10 | 0.65 |
| NM_024819 | FLJ22955 | 0.65 | NM_024917 | FLJ12687 | 0.65 |
| NM_031491 | RBP5 | 0.65 | NM_032289 | DKFZP761B0514 | 0.65 |
| NM_194325 | ZNF30 | 0.65 | NM_080660 | MGC14289 | 0.65 |
| NM_144636 | CHCHD4 | 0.65 | NM_181533 | C14ORF29 | 0.65 |
| NM_144679 | FLJ31528 | 0.65 | NM_178835 | LOC152485 | 0.65 |
| NM_133267 | GSH-2 | 0.65 | NM_153711 | C6ORF188 | 0.65 |
| XM_378794 | LOC284402 | 0.65 | NM_181809 | BMP8A | 0.65 |
| NM_004313 | ARRB2 | 0.65 | XM_170777 | LOC255320 | 0.65 |
| XM_059492 | LOC131076 | 0.65 | XM_115100 | LOC196346 | 0.65 |
| XM_067076 | LOC140103 | 0.65 | XM_371738 | LOC389289 | 0.65 |
| XM_372163 | LOC389819 | 0.65 | XM_371949 | LOC389550 | 0.65 |
| XM_372432 | LOC390256 | 0.65 | NM_001001325 | SPINK5L2 | 0.65 |
| NM_001003807 | FLJ35429 | 0.65 | NM_000076 | CDKN1C | 0.66 |
| NM_030662 | MAP2K2 | 0.66 | NM_001727 | BRS3 | 0.66 |
| NM_000164 | GIPR | 0.66 | NM_004362 | CLGN | 0.66 |
| NM_001743 | CALM2 | 0.66 | NM_006570 | RRAGA | 0.66 |
| NM_004289 | NFE2L3 | 0.66 | NM_003187 | TAF9 | 0.66 |
| NM_002103 | GYS1 | 0.66 | NM_012214 | MGAT4A | 0.66 |
| NM_002772 | PRSS7 | 0.66 | NM_000123 | ERCC5 | 0.66 |
| NM_006380 | APPBP2 | 0.66 | NM_000264 | PTCH | 0.66 |
| NM_004931 | CD8B1 | 0.66 | NM_014251 | SLC25A13 | 0.66 |
| NM_001150 | ANPEP | 0.66 | NM_006516 | SLC2A1 | 0.66 |
| NM_005214 | CTLA4 | 0.66 | NM_006806 | BTG3 | 0.66 |
| NM_016247 | IMPG2 | 0.66 | NM_004508 | IDI1 | 0.66 |
| NM_000317 | PTS | 0.66 | NM_014633 | SH2BP1 | 0.66 |
| NM_006682 | FGL2 | 0.66 | NM_015066 | TRIM35 | 0.66 |
| NM_015172 | XTP2 | 0.66 | NM_173630 | RTTN | 0.66 |
| NM_012196 | GAGE8 | 0.66 | NM_021161 | KCNK10 | 0.66 |
| NM_016410 | C9ORF83 | 0.66 | NM_017866 | FLJ20533 | 0.66 |
| NM_018228 | C14ORF115 | 0.66 | NM_018399 | VNN3 | 0.66 |
| NM_020158 | EXOSC5 | 0.66 | NM_020359 | PLSCR2 | 0.66 |
| NM_020769 | KIAA1318 | 0.66 | NM_024618 | NOD9 | 0.66 |
| NM_024821 | FLJ22349 | 0.66 | NM_032120 | DKFZP564O0523 | 0.66 |
| NM_138350 | MGC33488 | 0.66 | NM_052846 | EMILIN3 | 0.66 |
| XM_371760 | LOC116068 | 0.66 | NM_138371 | MGC16044 | 0.66 |
| NM_133446 | CTGLF1 | 0.66 | NM_153346 | CXORF20 | 0.66 |
| NM_194285 | FLJ39441 | 0.66 | NM_145036 | MGC33887 | 0.66 |
| NM_152898 | FERD3L | 0.66 | NM_174932 | BPIL2 | 0.66 |
| XM_379438 | LOC285740 | 0.66 | XM_209509 | LOC285193 | 0.66 |
| NM_178542 | DKFZP762C2414 | 0.66 | NM_199290 | MGC71999 | 0.66 |
| NM_181506 | SLRN | 0.66 | NM_207480 | UNQ5830 | 0.66 |

| | | |
|---|---|---|
| XM_064003 | LOC124149 | 0.66 |
| XM_373886 | LOC388738 | 0.66 |
| XM_376488 | LOC401252 | 0.66 |
| XM_379516 | LOC401398 | 0.66 |
| XM_374741 | LOC392749 | 0.66 |
| NM_000064 | C3 | 0.67 |
| NM_005285 | GPR7 | 0.67 |
| NM_000615 | NCAM1 | 0.67 |
| NM_000380 | XPA | 0.67 |
| NM_006084 | ISGF3G | 0.67 |
| NM_022164 | LCN7 | 0.67 |
| NM_006670 | TPBG | 0.67 |
| NM_005993 | TBCD | 0.67 |
| NM_002939 | RNH | 0.67 |
| NM_080611 | DUSP15 | 0.67 |
| NM_000617 | SLC11A2 | 0.67 |
| NM_002297 | LCN1 | 0.67 |
| NM_015722 | DRD1IP | 0.67 |
| NM_007283 | MGLL | 0.67 |
| NM_001970 | EIF5A | 0.67 |
| NM_007103 | NDUFV1 | 0.67 |
| NM_003764 | STX11 | 0.67 |
| NM_004845 | PCYT1B | 0.67 |
| NM_005156 | ROD1 | 0.67 |
| NM_006566 | CD226 | 0.67 |
| NM_007347 | AP4E1 | 0.67 |
| NM_014600 | EHD3 | 0.67 |
| NM_019592 | RNF20 | 0.67 |
| NM_021167 | ODAG | 0.67 |
| NM_022140 | EPB41L4A | 0.67 |
| NM_024689 | FLJ14103 | 0.67 |
| NM_025045 | FLJ22582 | 0.67 |
| NM_025222 | PRO2730 | 0.67 |
| XM_290737 | ZNF594 | 0.67 |
| NM_138567 | SYT8 | 0.67 |
| NM_138783 | LOC115950 | 0.67 |
| NM_175056 | LOC131368 | 0.67 |
| NM_152398 | MGC45416 | 0.67 |
| NM_130388 | ASB12 | 0.67 |
| XM_379207 | LOC285194 | 0.67 |
| XM_379254 | LOC339988 | 0.67 |
| NM_182974 | GLTDC1 | 0.67 |
| XM_114301 | LOC200810 | 0.67 |
| XM_059047 | LOC126435 | 0.67 |
| XM_291763 | LOC340947 | 0.67 |
| XM_379493 | LOC401352 | 0.67 |
| XM_291139 | LOC340075 | 0.66 |
| XM_373873 | LOC388713 | 0.66 |
| XM_374222 | LOC389526 | 0.66 |
| XM_373470 | LOC387705 | 0.66 |
| NM_001346 | DGKG | 0.67 |
| NM_001784 | CD97 | 0.67 |
| NM_005716 | RGS19IP1 | 0.67 |
| NM_012294 | RAPGEF5 | 0.67 |
| NM_003670 | BHLHB2 | 0.67 |
| NM_007249 | KLF12 | 0.67 |
| NM_000305 | PON2 | 0.67 |
| NM_004665 | VNN2 | 0.67 |
| NM_000301 | PLG | 0.67 |
| NM_005393 | PLXNB3 | 0.67 |
| NM_020387 | RAB25 | 0.67 |
| NM_002237 | KCNG1 | 0.67 |
| NM_175069 | APTX | 0.67 |
| NM_017649 | CNNM2 | 0.67 |
| NM_004283 | RAB3D | 0.67 |
| NM_005014 | OMD | 0.67 |
| NM_003127 | SPTAN1 | 0.67 |
| NM_004265 | FADS2 | 0.67 |
| NM_003850 | SUCLA2 | 0.67 |
| NM_014834 | KIAA0563 | 0.67 |
| NM_152740 | HIBADH | 0.67 |
| NM_013327 | PARVB | 0.67 |
| NM_018009 | TAPBPL | 0.67 |
| NM_020812 | DOCK6 | 0.67 |
| NM_022123 | NPAS3 | 0.67 |
| NM_024653 | PRKRIP1 | 0.67 |
| NM_030571 | NDFIP1 | 0.67 |
| XM_040383 | KLHL15 | 0.67 |
| NM_032145 | FBXO30 | 0.67 |
| NM_178448 | C9ORF140 | 0.67 |
| NM_032160 | C18ORF4 | 0.67 |
| NM_138442 | LOC115098 | 0.67 |
| NM_173481 | C19ORF21 | 0.67 |
| XM_378487 | LOC145216 | 0.67 |
| NM_052867 | VGCNL1 | 0.67 |
| XM_291063 | LOC339903 | 0.67 |
| NM_198560 | LOC375323 | 0.67 |
| XM_113825 | LOC197135 | 0.67 |
| XM_065828 | LOC130678 | 0.67 |
| XM_084377 | LOC142910 | 0.67 |
| XM_373057 | LOC391745 | 0.67 |
| XM_374186 | LOC389438 | 0.67 |

| | | | | | |
|---|---|---|---|---|---|
| XM_370603 | LOC387745 | 0.67 | XM_373723 | LOC388365 | 0.67 |
| XM_378738 | LOC400643 | 0.67 | XM_371006 | LOC388323 | 0.67 |
| NM_001002923 | IGFL4 | 0.67 | NM_001005338 | OR5H1 | 0.67 |
| NM_023018 | FLJ13052 | 0.68 | NM_002733 | PRKAG1 | 0.68 |
| NM_001504 | CXCR3 | 0.68 | NM_015191 | SIK2 | 0.68 |
| NM_000172 | GNAT1 | 0.68 | NM_000866 | HTR1F | 0.68 |
| NM_004178 | TARBP2 | 0.68 | NM_000522 | HOXA13 | 0.68 |
| NM_000202 | IDS | 0.68 | NM_003711 | PPAP2A | 0.68 |
| NM_000667 | ADH1A | 0.68 | NM_006377 | UNC13B | 0.68 |
| XM_290793 | KSR | 0.68 | NM_002317 | LOX | 0.68 |
| NM_000360 | TH | 0.68 | NM_001175 | ARHGDIB | 0.68 |
| NM_003884 | PCAF | 0.68 | NM_003255 | TIMP2 | 0.68 |
| NM_000573 | CR1 | 0.68 | NM_004625 | WNT7A | 0.68 |
| NM_001242 | TNFRSF7 | 0.68 | NM_000814 | GABRB3 | 0.68 |
| NM_000361 | THBD | 0.68 | NM_020236 | MRPL1 | 0.68 |
| NM_000095 | COMP | 0.68 | NM_000713 | BLVRB | 0.68 |
| NM_012139 | DELGEF | 0.68 | NM_000457 | HNF4A | 0.68 |
| NM_000194 | HPRT1 | 0.68 | NM_001140 | ALOX15 | 0.68 |
| NM_002100 | GYPB | 0.68 | NM_005329 | HAS3 | 0.68 |
| NM_004499 | HNRPAB | 0.68 | NM_006918 | SC5DL | 0.68 |
| NM_006916 | RPE | 0.68 | NM_003532 | HIST1H3E | 0.68 |
| NM_006392 | NOL5A | 0.68 | NM_015238 | KIBRA | 0.68 |
| NM_016400 | HYPK | 0.68 | NM_012260 | HPCL2 | 0.68 |
| NM_016433 | GLTP | 0.68 | NM_016645 | NEUGRIN | 0.68 |
| NM_018067 | FLJ10350 | 0.68 | NM_018657 | MYNN | 0.68 |
| NM_018915 | PCDHGA2 | 0.68 | NM_022761 | C11ORF1 | 0.68 |
| NM_030935 | THG-1 | 0.68 | NM_018092 | NETO2 | 0.68 |
| NM_032389 | ZNF289 | 0.68 | NM_032775 | FLJ14360 | 0.68 |
| NM_033208 | TIGD7 | 0.68 | NM_152864 | C20ORF58 | 0.68 |
| NM_145808 | MTPN | 0.68 | XM_379215 | LOC132241 | 0.68 |
| NM_152401 | PDCL2 | 0.68 | NM_178493 | LOC147111 | 0.68 |
| NM_207324 | LOC147650 | 0.68 | NM_178497 | FLJ23657 | 0.68 |
| NM_178504 | FLJ40427 | 0.68 | XM_114611 | KIAA1833 | 0.68 |
| XM_292982 | LOC344227 | 0.68 | XM_084467 | LOC143244 | 0.68 |
| XM_375849 | LOC400809 | 0.68 | XM_379523 | LOC401407 | 0.68 |
| XM_374405 | LOC392634 | 0.68 | XM_374271 | LOC389665 | 0.68 |
| XM_370711 | LOC387904 | 0.68 | XM_370724 | LOC387923 | 0.68 |
| NM_003886 | AKAP4 | 0.69 | NM_006285 | TESK1 | 0.69 |
| NM_003485 | GPR68 | 0.69 | NM_005290 | GPR15 | 0.69 |
| NM_001058 | TACR1 | 0.69 | NM_130897 | DNCL2B | 0.69 |
| NM_006272 | S100B | 0.69 | NM_017481 | UBQLN3 | 0.69 |
| NM_006907 | PYCR1 | 0.69 | NM_000920 | PC | 0.69 |
| NM_018690 | APOB48R | 0.69 | NM_003828 | MTMR1 | 0.69 |
| NM_018113 | LIMR | 0.69 | NM_005398 | PPP1R3C | 0.69 |
| NM_001772 | CD33 | 0.69 | NM_002169 | IFNA5 | 0.69 |
| NM_016326 | CKLF | 0.69 | NM_006183 | NTS | 0.69 |

| | | | | | |
|---|---|---|---|---|---|
| NM_178231 | ALS2CR14 | 0.69 | NM_021137 | TNFAIP1 | 0.69 |
| NM_005688 | ABCC5 | 0.69 | NM_024632 | SAP30L | 0.69 |
| NM_002311 | LIG3 | 0.69 | NM_014348 | POM121L1 | 0.69 |
| NM_005001 | NDUFA7 | 0.69 | NM_006319 | CDIPT | 0.69 |
| NM_015199 | ANKRD28 | 0.69 | NM_014901 | RNF44 | 0.69 |
| NM_015289 | VPS39 | 0.69 | NM_015565 | ZNF294 | 0.69 |
| NM_013387 | HSPC051 | 0.69 | NM_016938 | EFEMP2 | 0.69 |
| NM_018457 | DKFZP564J157 | 0.69 | NM_018484 | SLC22A11 | 0.69 |
| XM_048747 | KIAA1223 | 0.69 | NM_024709 | FLJ14146 | 0.69 |
| NM_024743 | FLJ21934 | 0.69 | NM_024757 | EHMT1 | 0.69 |
| NM_024922 | FLJ21736 | 0.69 | NM_030815 | C20ORF126 | 0.69 |
| NM_030940 | HBLD2 | 0.69 | NM_032369 | MGC15619 | 0.69 |
| NM_144581 | C14ORF149 | 0.69 | NM_153713 | MGC46719 | 0.69 |
| NM_153447 | NALP5 | 0.69 | NM_173484 | FLJ40160 | 0.69 |
| NM_144639 | FLJ31300 | 0.69 | NM_173493 | LOC139135 | 0.69 |
| NM_153360 | FLJ90166 | 0.69 | NM_181773 | ARP10 | 0.69 |
| NM_173576 | C10ORF48 | 0.69 | NM_198185 | OVTN | 0.69 |
| NM_198511 | FLJ42925 | 0.69 | XM_294894 | LOC339281 | 0.69 |
| XM_067994 | LOC132706 | 0.69 | XM_086879 | LOC150371 | 0.69 |
| XM_373881 | LOC388727 | 0.69 | XM_377925 | LOC402244 | 0.69 |
| XM_370899 | LOC388167 | 0.69 | XM_375147 | LOC400298 | 0.69 |
| XM_379722 | LOC401616 | 0.69 | XM_374318 | LOC389834 | 0.69 |
| NM_003954 | MAP3K14 | 0.7 | NM_005296 | GPR23 | 0.7 |
| NM_030774 | OR51E2 | 0.7 | NM_001455 | FOXO3A | 0.7 |
| NM_006004 | UQCRH | 0.7 | NM_001661 | ARF4L | 0.7 |
| NM_003799 | RNMT | 0.7 | NM_015316 | PPP1R13B | 0.7 |
| NM_016848 | SHC3 | 0.7 | NM_013278 | IL17C | 0.7 |
| NM_001081 | CUBN | 0.7 | NM_002313 | ABLIM1 | 0.7 |
| NM_180989 | ITR | 0.7 | XM_374502 | MUC3A | 0.7 |
| NM_002212 | ITGB4BP | 0.7 | NM_002125 | HLA-DRB5 | 0.7 |
| NM_002443 | MSMB | 0.7 | NM_003270 | TM4SF6 | 0.7 |
| NM_003517 | HIST2H2AC | 0.7 | NM_005769 | CHST4 | 0.7 |
| NM_006553 | C18ORF43 | 0.7 | NM_007270 | FKBP9 | 0.7 |
| NM_015252 | NACSIN | 0.7 | NM_015057 | MYCBP2 | 0.7 |
| NM_012095 | AP3M1 | 0.7 | XM_372387 | OR5L2 | 0.7 |
| NM_017831 | RNF125 | 0.7 | NM_017711 | GDPD2 | 0.7 |
| NM_017625 | ITLN1 | 0.7 | NM_020150 | SARA1 | 0.7 |
| NM_024702 | FLJ13841 | 0.7 | NM_024686 | FLJ23033 | 0.7 |
| NM_025141 | BLP2 | 0.7 | XM_374922 | KIAA1731 | 0.7 |
| NM_138349 | LOC90313 | 0.7 | NM_153336 | C10ORF89 | 0.7 |
| NM_139243 | TENR | 0.7 | NM_152382 | FLJ37953 | 0.7 |
| XM_166532 | KIAA1950 | 0.7 | XM_375917 | LOC149692 | 0.7 |
| NM_198459 | FLJ37099 | 0.7 | NM_145314 | C10ORF49 | 0.7 |
| XM_210562 | LOC285335 | 0.7 | NM_173666 | FLJ33977 | 0.7 |
| XM_371320 | FLJ00193 | 0.7 | NM_198353 | KCTD8 | 0.7 |
| NM_206895 | UNQ830 | 0.7 | XM_061880 | LOC120126 | 0.7 |

| | | |
|---|---|---|
| XM_378842 | LOC400742 | 0.7 |
| XM_372352 | LOC390054 | 0.7 |
| XM_378367 | LOC400053 | 0.7 |
| NM_018170 | P15RS | 0.71 |
| NM_020778 | MIDORI | 0.71 |
| NM_016453 | NCKIPSD | 0.71 |
| NM_012153 | EHF | 0.71 |
| NM_145004 | ADAM32 | 0.71 |
| NM_002340 | LSS | 0.71 |
| NM_178500 | PHOSPHO1 | 0.71 |
| NM_021628 | ALOXE3 | 0.71 |
| NM_001656 | TRIM23 | 0.71 |
| NM_000888 | ITGB6 | 0.71 |
| NM_021833 | UCP1 | 0.71 |
| NM_006340 | BAIAP2 | 0.71 |
| NM_006342 | TACC3 | 0.71 |
| NM_002483 | CEACAM6 | 0.71 |
| NM_006471 | MRCL3 | 0.71 |
| NM_015015 | JMJD2B | 0.71 |
| NM_014317 | TPRT | 0.71 |
| NM_080671 | KCNE4 | 0.71 |
| XM_210581 | CLDN22 | 0.71 |
| NM_020376 | TTS-2.2 | 0.71 |
| NM_021632 | ZNF350 | 0.71 |
| NM_032130 | DKFZP434J0113 | 0.71 |
| NM_182495 | FLJ25224 | 0.71 |
| NM_152320 | FLJ31295 | 0.71 |
| NM_145913 | SLC5A8 | 0.71 |
| NM_175918 | FLJ34443 | 0.71 |
| NM_175066 | DDX51 | 0.71 |
| XM_212238 | LOC286235 | 0.71 |
| XM_210168 | LOC283092 | 0.71 |
| XM_379201 | LOC401079 | 0.71 |
| XM_380113 | LOC402516 | 0.71 |
| NM_001001704 | FLJ44796 | 0.71 |
| NM_012193 | FZD4 | 0.72 |
| NM_181791 | GPR141 | 0.72 |
| NM_001453 | FOXC1 | 0.72 |
| NM_002583 | PAWR | 0.72 |
| NM_000672 | ADH6 | 0.72 |
| XM_370662 | OR8G2 | 0.72 |
| NM_002300 | LDHB | 0.72 |
| NM_153487 | MDGA1 | 0.72 |
| NM_001127 | AP1B1 | 0.72 |
| NM_004377 | CPT1B | 0.72 |
| NM_015239 | AGTPBP1 | 0.72 |
| XM_371841 | LOC389419 | 0.7 |
| XM_377185 | LOC401679 | 0.7 |
| XM_372866 | LOC391248 | 0.7 |
| NM_006457 | LIM | 0.71 |
| NM_017572 | MKNK2 | 0.71 |
| NM_004833 | AIM2 | 0.71 |
| NM_000019 | ACAT1 | 0.71 |
| NM_001818 | AKR1C4 | 0.71 |
| NM_006849 | PDIP | 0.71 |
| NM_025191 | C1ORF22 | 0.71 |
| NM_003730 | RNASET2 | 0.71 |
| NM_007079 | PTP4A3 | 0.71 |
| NM_006889 | CD86 | 0.71 |
| NM_003305 | TRPC3 | 0.71 |
| NM_006214 | PHYH | 0.71 |
| NM_004036 | ADCY3 | 0.71 |
| NM_006927 | SIAT4B | 0.71 |
| NM_015198 | COBL | 0.71 |
| NM_015134 | M-RIP | 0.71 |
| NM_014297 | ETHE1 | 0.71 |
| NM_014487 | ZNF330 | 0.71 |
| NM_016390 | C9ORF114 | 0.71 |
| NM_020678 | HT017 | 0.71 |
| NM_031419 | MAIL | 0.71 |
| NM_033549 | TRIM41 | 0.71 |
| NM_153022 | FLJ31166 | 0.71 |
| NM_182766 | FLJ32940 | 0.71 |
| NM_145654 | RAD52B | 0.71 |
| XM_378599 | LOC283854 | 0.71 |
| XM_295263 | LOC340508 | 0.71 |
| XM_292968 | LOC344191 | 0.71 |
| XM_371344 | LOC388743 | 0.71 |
| XM_372076 | LOC389705 | 0.71 |
| XM_370674 | LOC387832 | 0.71 |
| NM_007064 | KALRN | 0.72 |
| NM_005990 | STK10 | 0.72 |
| NM_003020 | SGNE1 | 0.72 |
| NM_006166 | NFYB | 0.72 |
| NM_004462 | FDFT1 | 0.72 |
| NM_004320 | ATP2A1 | 0.72 |
| NM_016382 | CD244 | 0.72 |
| NM_007031 | HSF2BP | 0.72 |
| NM_018255 | STATIP1 | 0.72 |
| NM_001217 | CA11 | 0.72 |
| NM_012207 | HNRPH3 | 0.72 |
| NM_006973 | ZNF32 | 0.72 |

| | | | | | |
|---|---|---|---|---|---|
| NM_003729 | RTCD1 | 0.72 | NM_015107 | PHF8 | 0.72 |
| NM_013267 | GLS2 | 0.72 | NM_015621 | DKFZP434C171 | 0.72 |
| NM_013450 | BAZ2B | 0.72 | NM_017547 | H17 | 0.72 |
| NM_020190 | OLFML3 | 0.72 | NM_022134 | GAL3ST2 | 0.72 |
| NM_022341 | PDF | 0.72 | NM_024745 | SHCBP1 | 0.72 |
| NM_024563 | FLJ14054 | 0.72 | NM_032803 | SLC7A3 | 0.72 |
| NM_031854 | KRTAP4-12 | 0.72 | NM_032901 | MGC14288 | 0.72 |
| NM_207316 | UNQ846 | 0.72 | NM_152429 | C10ORF13 | 0.72 |
| XM_086046 | ZNF599 | 0.72 | NM_139173 | LOC150159 | 0.72 |
| NM_152588 | DKFZP762A217 | 0.72 | NM_152687 | FLJ33641 | 0.72 |
| NM_181841 | TMC3 | 0.72 | NM_182619 | LOC348174 | 0.72 |
| XM_290342 | LOC339746 | 0.72 | XM_069734 | LOC136143 | 0.72 |
| XM_371207 | LOC388579 | 0.72 | XM_379494 | LOC401353 | 0.72 |
| XM_374200 | LOC389457 | 0.72 | XM_371885 | LOC389473 | 0.72 |
| XM_371731 | LOC389281 | 0.72 | XM_379452 | LOC401282 | 0.72 |
| XM_376783 | LOC401466 | 0.72 | XM_378956 | LOC400831 | 0.72 |
| NM_001004328 | FLJ16353 | 0.72 | NM_001004698 | OR2W5 | 0.72 |
| NM_004441 | EPHB1 | 0.73 | NM_014776 | GIT2 | 0.73 |
| NM_022437 | ABCG8 | 0.73 | NM_004860 | FXR2 | 0.73 |
| NM_020239 | SPEC1 | 0.73 | NM_000148 | FUT1 | 0.73 |
| NM_006033 | LIPG | 0.73 | NM_005759 | ABI2 | 0.73 |
| NM_003064 | SLPI | 0.73 | NM_006537 | USP3 | 0.73 |
| NM_014634 | PPM1F | 0.73 | NM_004747 | DLG5 | 0.73 |
| NM_006783 | GJB6 | 0.73 | NM_006066 | AKR1A1 | 0.73 |
| NM_004975 | KCNB1 | 0.73 | NM_005712 | HHLA1 | 0.73 |
| NM_004473 | FOXE1 | 0.73 | NM_052902 | STK11IP | 0.73 |
| NM_002882 | RANBP1 | 0.73 | NM_013265 | C11ORF2 | 0.73 |
| NM_001978 | EPB49 | 0.73 | NM_021134 | MRPL23 | 0.73 |
| NM_005619 | RTN2 | 0.73 | NM_001074 | UGT2B7 | 0.73 |
| NM_003245 | TGM3 | 0.73 | NM_005789 | PSME3 | 0.73 |
| NM_014773 | KIAA0141 | 0.73 | NM_006675 | NET-5 | 0.73 |
| NM_015341 | BRRN1 | 0.73 | NM_012241 | SIRT5 | 0.73 |
| NM_012154 | EIF2C2 | 0.73 | NM_152354 | ZNF285 | 0.73 |
| NM_015705 | RUTBC3 | 0.73 | NM_016069 | MAGMAS | 0.73 |
| NM_033048 | CPXCR1 | 0.73 | NM_018043 | TMEM16A | 0.73 |
| NM_017832 | FLJ20457 | 0.73 | NM_021226 | ARHGAP22 | 0.73 |
| XM_042661 | KIAA1530 | 0.73 | NM_024050 | PCIA1 | 0.73 |
| NM_024035 | MGC3113 | 0.73 | NM_024945 | C9ORF76 | 0.73 |
| NM_031290 | DKFZP434K1172 | 0.73 | NM_032333 | C10ORF58 | 0.73 |
| NM_032536 | NTNG2 | 0.73 | XM_379680 | LOC90120 | 0.73 |
| NM_145645 | WBSCR20B | 0.73 | XM_050219 | SYNPO2 | 0.73 |
| NM_153241 | MGC42157 | 0.73 | NM_152743 | C7ORF27 | 0.73 |
| XM_167072 | BZRPL1 | 0.73 | NM_181723 | LOC286097 | 0.73 |
| NM_205854 | UNQ541 | 0.73 | XM_063287 | LOC122706 | 0.73 |
| XM_374055 | LOC389159 | 0.73 | XM_380114 | LOC402523 | 0.73 |
| NM_212555 | UNQ3112 | 0.73 | NM_007207 | DUSP10 | 0.74 |

| | | | | | |
|---|---|---|---|---|---|
| NM_005076 | CNTN2 | 0.74 | NM_005901 | SMAD2 | 0.74 |
| NM_007361 | NID2 | 0.74 | NM_003342 | UBE2G1 | 0.74 |
| NM_005077 | TLE1 | 0.74 | NM_000277 | PAH | 0.74 |
| NM_000924 | PDE1B | 0.74 | NM_005941 | MMP16 | 0.74 |
| NM_000096 | CP | 0.74 | XM_165973 | USP24 | 0.74 |
| NM_006840 | LILRB5 | 0.74 | NM_002856 | PVRL2 | 0.74 |
| NM_013431 | KLRC4 | 0.74 | NM_000347 | SPTB | 0.74 |
| NM_004866 | SCAMP1 | 0.74 | NM_000297 | PKD2 | 0.74 |
| NM_003874 | CD84 | 0.74 | NM_138578 | BCL2L1 | 0.74 |
| NM_014266 | HCST | 0.74 | NM_015898 | ZBTB7 | 0.74 |
| NM_021954 | GJA3 | 0.74 | NM_002166 | ID2 | 0.74 |
| NM_020640 | RP42 | 0.74 | NM_033033 | KRTHB2 | 0.74 |
| NM_002635 | SLC25A3 | 0.74 | NM_014782 | ARMCX2 | 0.74 |
| NM_006661 | PDE10A | 0.74 | NM_007196 | KLK8 | 0.74 |
| NM_007268 | Z39IG | 0.74 | NM_015395 | DKFZP434B0335 | 0.74 |
| NM_013313 | YPEL1 | 0.74 | NM_016148 | SHANK1 | 0.74 |
| NM_016588 | NRN1 | 0.74 | NM_015622 | C7ORF28A | 0.74 |
| NM_018474 | C20ORF19 | 0.74 | NM_020405 | PLXDC1 | 0.74 |
| XM_043653 | BEXL1 | 0.74 | XM_166707 | OR13A1 | 0.74 |
| NM_138435 | LOC113828 | 0.74 | NM_058165 | MOGAT1 | 0.74 |
| NM_152285 | ARRDC1 | 0.74 | NM_033328 | CAPZA3 | 0.74 |
| NM_152219 | GJC1 | 0.74 | NM_152607 | FLJ40201 | 0.74 |
| NM_173081 | ARMC3 | 0.74 | NM_173574 | MGC33414 | 0.74 |
| NM_199135 | FOXD4L2 | 0.74 | NM_182606 | LOC339967 | 0.74 |
| NM_207509 | FLJ46836 | 0.74 | XM_059608 | LOC132870 | 0.74 |
| XM_098980 | LOC158730 | 0.74 | XM_374022 | LOC389070 | 0.74 |
| XM_377942 | LOC402273 | 0.74 | XM_371141 | LOC388508 | 0.74 |
| NM_001005468 | OR8B2 | 0.74 | NM_002743 | PRKCSH | 0.75 |
| NM_003265 | TLR3 | 0.75 | NM_018909 | PCDHA6 | 0.75 |
| NM_005978 | S100A2 | 0.75 | NM_020549 | CHAT | 0.75 |
| NM_004609 | TCF15 | 0.75 | NM_003689 | AKR7A2 | 0.75 |
| NM_001334 | CTSO | 0.75 | NM_017726 | PPP1R14D | 0.75 |
| NM_003811 | TNFSF9 | 0.75 | NM_004428 | EFNA1 | 0.75 |
| NM_004106 | FCER1G | 0.75 | NM_006540 | NCOA2 | 0.75 |
| NM_018234 | TSAP6 | 0.75 | NM_012120 | CD2AP | 0.75 |
| NM_002150 | HPD | 0.75 | NM_001689 | ATP5G3 | 0.75 |
| NM_031900 | AGXT2 | 0.75 | NM_003436 | ZNF135 | 0.75 |
| NM_003375 | VDAC2 | 0.75 | XM_496278 | ZNF516 | 0.75 |
| NM_015208 | ANKRD12 | 0.75 | NM_015908 | ARS2 | 0.75 |
| NM_016019 | LUC7L2 | 0.75 | NM_019055 | ROBO4 | 0.75 |
| XM_035953 | C9ORF11 | 0.75 | NM_018312 | C11ORF23 | 0.75 |
| NM_138704 | NDNL2 | 0.75 | XM_045271 | NGL-1 | 0.75 |
| NM_022061 | MRPL17 | 0.75 | NM_022102 | C6ORF79 | 0.75 |
| NM_030577 | MGC10993 | 0.75 | NM_024872 | FLJ22570 | 0.75 |
| NM_031297 | DKFZP761H1710 | 0.75 | NM_032243 | TXNDC2 | 0.75 |
| NM_033123 | PLCZ1 | 0.75 | NM_174890 | ANUBL1 | 0.75 |

| | | | | | |
|---|---|---|---|---|---|
| XM_379154 | LOC151300 | 0.75 | NM_144966 | C9ORF154 | 0.75 |
| NM_145715 | TIGD2 | 0.75 | NM_173558 | FGD2 | 0.75 |
| NM_152747 | DKFZP586I1420 | 0.75 | XM_379340 | LOC285713 | 0.75 |
| NM_181654 | CPLX4 | 0.75 | NM_182633 | FLJ39963 | 0.75 |
| XM_166160 | LOC221017 | 0.75 | XM_373868 | LOC388697 | 0.75 |
| XM_374033 | LOC389095 | 0.75 | XM_373031 | LOC391692 | 0.75 |
| XM_377974 | LOC402300 | 0.75 | XM_372123 | LOC389766 | 0.75 |
| XM_378437 | LOC400164 | 0.75 | XM_373790 | LOC388497 | 0.75 |
| NM_001005479 | OR5H6 | 0.75 | NM_002747 | MAPK4 | 0.76 |
| NM_002759 | PRKR | 0.76 | NM_016542 | MST4 | 0.76 |
| NM_000913 | OPRL1 | 0.76 | NM_004381 | CREBL1 | 0.76 |
| NM_000027 | AGA | 0.76 | NM_012079 | DGAT1 | 0.76 |
| NM_000791 | DHFR | 0.76 | NM_003238 | TGFB2 | 0.76 |
| NM_001469 | G22P1 | 0.76 | XM_056455 | D2S448 | 0.76 |
| NM_138806 | CD200R | 0.76 | NM_002193 | INHBB | 0.76 |
| NM_000612 | IGF2 | 0.76 | NM_002374 | MAP2 | 0.76 |
| XM_046570 | KIAA1679 | 0.76 | NM_006023 | C10ORF7 | 0.76 |
| NM_018963 | C21ORF107 | 0.76 | NM_016124 | RHD | 0.76 |
| NM_014715 | RICS | 0.76 | NM_003184 | TAF2 | 0.76 |
| NM_005057 | RBBP5 | 0.76 | NR_001275 | CELP | 0.76 |
| NM_005630 | SLCO2A1 | 0.76 | NM_006512 | SAA4 | 0.76 |
| NM_006470 | TRIM16 | 0.76 | NM_015125 | CIC | 0.76 |
| NM_014935 | PEPP3 | 0.76 | NM_014351 | SULT4A1 | 0.76 |
| NM_033160 | DKFZP572C163 | 0.76 | NM_016499 | MGC:13379 | 0.76 |
| NM_015522 | D2LIC | 0.76 | NM_018164 | FLJ10637 | 0.76 |
| NM_018349 | FLJ11175 | 0.76 | XM_048675 | KIAA1238 | 0.76 |
| NM_025202 | EFHD1 | 0.76 | NM_152302 | C20ORF158 | 0.76 |
| NM_033401 | CNTNAP4 | 0.76 | NM_138384 | SPRN | 0.76 |
| NM_153369 | KIAA1919 | 0.76 | NM_152455 | FLJ35867 | 0.76 |
| NM_182832 | PLAC4 | 0.76 | NM_194324 | MGC39900 | 0.76 |
| NM_207437 | FLJ43486 | 0.76 | NM_203400 | LOC388394 | 0.76 |
| NM_001995 | ACSL1 | 0.76 | XM_373884 | LOC388732 | 0.76 |
| XM_376784 | LOC401467 | 0.76 | XM_377278 | LOC401725 | 0.76 |
| XM_377538 | LOC401923 | 0.76 | XM_374341 | LOC389908 | 0.76 |
| NM_001992 | F2R | 0.77 | NM_002386 | MC1R | 0.77 |
| NM_013388 | PREB | 0.77 | NM_001189 | BAPX1 | 0.77 |
| NM_004865 | TBPL1 | 0.77 | NM_003437 | ZNF136 | 0.77 |
| NM_007162 | TFEB | 0.77 | NM_004617 | TM4SF4 | 0.77 |
| NM_000785 | CYP27B1 | 0.77 | NM_014470 | RND1 | 0.77 |
| NM_012397 | SERPINB13 | 0.77 | NM_000362 | TIMP3 | 0.77 |
| NM_000551 | VHL | 0.77 | NM_004707 | APG12L | 0.77 |
| NM_032515 | BOK | 0.77 | NM_014710 | GASP | 0.77 |
| NM_002306 | LGALS3 | 0.77 | NM_005368 | MB | 0.77 |
| NM_002394 | SLC3A2 | 0.77 | NM_002852 | PTX3 | 0.77 |
| NM_152392 | AHSA2 | 0.77 | NM_004731 | SLC16A7 | 0.77 |
| NM_014972 | KIAA1049 | 0.77 | XM_377912 | KIAA0194 | 0.77 |

| | | |
|---|---|---|
| XM_371617 | TIP120B | 0.77 |
| NM_014375 | FETUB | 0.77 |
| NM_024506 | GLB1L | 0.77 |
| NM_025147 | FLJ13448 | 0.77 |
| NM_138368 | DKFZP761E198 | 0.77 |
| NM_139280 | ORMDL3 | 0.77 |
| NM_152403 | FLJ39155 | 0.77 |
| NM_138815 | LOC151871 | 0.77 |
| NM_152615 | FLJ40597 | 0.77 |
| XM_095965 | LOC169834 | 0.77 |
| NM_153692 | FLJ90724 | 0.77 |
| XM_292184 | LOC341720 | 0.77 |
| XM_379264 | LOC401128 | 0.77 |
| XM_373907 | LOC388787 | 0.77 |
| NM_212554 | LOC399818 | 0.77 |
| NM_018650 | MARK1 | 0.78 |
| NM_012395 | PFTK1 | 0.78 |
| NM_032554 | GPR81 | 0.78 |
| NM_004932 | CDH6 | 0.78 |
| NM_005902 | SMAD3 | 0.78 |
| NM_000691 | ALDH3A1 | 0.78 |
| NM_021147 | UNG2 | 0.78 |
| NM_000506 | F2 | 0.78 |
| NM_002827 | PTPN1 | 0.78 |
| NM_176817 | TAS2R38 | 0.78 |
| NM_001980 | EPIM | 0.78 |
| NM_030779 | KCNH6 | 0.78 |
| NM_005231 | CTTN | 0.78 |
| NM_003023 | SH3BP2 | 0.78 |
| NM_001355 | DDT | 0.78 |
| NM_006752 | SURF5 | 0.78 |
| NM_006864 | LILRB3 | 0.78 |
| NM_013435 | RAX | 0.78 |
| NM_016356 | DCDC2 | 0.78 |
| NM_017806 | FLJ20406 | 0.78 |
| NM_032885 | APG4D | 0.78 |
| NM_052958 | VEST1 | 0.78 |
| NM_153224 | MGC34034 | 0.78 |
| XM_375563 | OR7D2 | 0.78 |
| NM_181721 | FOXR1 | 0.78 |
| XM_379270 | CSN1S2A | 0.78 |
| XM_173164 | LOC256096 | 0.78 |
| XM_379078 | LOC400945 | 0.78 |
| XM_373726 | LOC388375 | 0.78 |
| NM_001005240 | OR4F17 | 0.78 |
| XM_291107 | NEK1 | 0.79 |
| XM_037557 | KIAA0984 | 0.77 |
| NM_015996 | CGI-40 | 0.77 |
| NM_025139 | FLJ12584 | 0.77 |
| NM_133483 | GEFT | 0.77 |
| NM_152290 | MGC35194 | 0.77 |
| NM_152356 | ZNF491 | 0.77 |
| NM_207320 | HSHIN6 | 0.77 |
| NM_144719 | FLJ25467 | 0.77 |
| NM_152625 | ZNF366 | 0.77 |
| NM_152679 | SLC10A4 | 0.77 |
| NM_147198 | WFDC9 | 0.77 |
| XM_374112 | LOC389270 | 0.77 |
| XM_373631 | LOC388129 | 0.77 |
| NM_001001317 | TRY1 | 0.77 |
| NM_002093 | GSK3B | 0.78 |
| NM_032387 | PRKWNK4 | 0.78 |
| XM_291859 | OR5F1 | 0.78 |
| NM_138327 | TRAR1 | 0.78 |
| NM_005739 | RASGRP1 | 0.78 |
| NM_016954 | TBX22 | 0.78 |
| NM_017811 | UBE2R2 | 0.78 |
| NM_004251 | RAB9A | 0.78 |
| NM_003589 | CUL4A | 0.78 |
| NM_030764 | SPAP1 | 0.78 |
| NM_001036 | RYR3 | 0.78 |
| NM_003277 | CLDN5 | 0.78 |
| NM_004947 | DOCK3 | 0.78 |
| NM_007076 | HYPE | 0.78 |
| NM_001288 | CLIC1 | 0.78 |
| NM_024758 | AGMAT | 0.78 |
| NM_006063 | KBTBD10 | 0.78 |
| XM_166571 | KIAA0363 | 0.78 |
| NM_015899 | LOC51054 | 0.78 |
| NM_017798 | YTHDF1 | 0.78 |
| NM_017713 | FLJ20211 | 0.78 |
| NM_152270 | FLJ34922 | 0.78 |
| NM_152353 | MGC33839 | 0.78 |
| NM_153014 | FLJ30634 | 0.78 |
| NM_181714 | C6ORF152 | 0.78 |
| NM_173625 | FLJ39647 | 0.78 |
| XM_089307 | LOC164036 | 0.78 |
| XM_209741 | LOC285741 | 0.78 |
| XM_374248 | LOC389624 | 0.78 |
| XM_372698 | LOC390865 | 0.78 |
| NM_004384 | CSNK1G3 | 0.79 |
| NM_001275 | CHGA | 0.79 |

| | | |
|---|---|---|
| NM_022443 | MLF1 | 0.79 |
| NM_002700 | POU4F3 | 0.79 |
| NM_031313 | ALPPL2 | 0.79 |
| NM_001728 | BSG | 0.79 |
| NM_000210 | ITGA6 | 0.79 |
| NM_013354 | CNOT7 | 0.79 |
| NM_006765 | TUSC3 | 0.79 |
| NM_004573 | PLCB2 | 0.79 |
| NM_005666 | HFL3 | 0.79 |
| NM_004288 | PSCDBP | 0.79 |
| NM_006591 | POLD3 | 0.79 |
| NM_014963 | KIAA0963 | 0.79 |
| NM_018944 | C21ORF45 | 0.79 |
| NM_021222 | HTCD37 | 0.79 |
| NM_022479 | WBSCR17 | 0.79 |
| NM_032822 | FLJ14668 | 0.79 |
| NM_198077 | GM117 | 0.79 |
| XM_375430 | LOC201229 | 0.79 |
| NM_145658 | SPESP1 | 0.79 |
| NM_153243 | MGC26143 | 0.79 |
| XM_374877 | DKFZP779M0652 | 0.79 |
| XM_376454 | LOC401224 | 0.79 |
| XM_374713 | LOC402455 | 0.79 |
| NM_001616 | ACVR2 | 0.8 |
| NM_001118 | ADCYAP1R1 | 0.8 |
| NM_052996 | PRDM7 | 0.8 |
| NM_001713 | BHMT | 0.8 |
| NM_007375 | TARDBP | 0.8 |
| NM_000764 | CYP2A7 | 0.8 |
| NM_002711 | PPP1R3A | 0.8 |
| NM_000217 | KCNA1 | 0.8 |
| NM_014312 | CTXL | 0.8 |
| NM_005176 | ATP5G2 | 0.8 |
| NM_003053 | SLC18A1 | 0.8 |
| NM_003135 | SRP19 | 0.8 |
| NM_014868 | RNF10 | 0.8 |
| XM_036988 | KIAA1000 | 0.8 |
| NM_012197 | RABGAP1 | 0.8 |
| NM_014179 | HSPC157 | 0.8 |
| NM_016478 | NIPA | 0.8 |
| NM_022771 | TBC1D15 | 0.8 |
| NM_025246 | TMEM22 | 0.8 |
| XM_370653 | KIAA1826 | 0.8 |
| XM_291077 | LOC90113 | 0.8 |
| XM_056282 | KIAA1904 | 0.8 |
| NM_033209 | LOC94105 | 0.8 |
| NM_002913 | RFC1 | 0.79 |
| NM_001224 | CASP2 | 0.79 |
| NM_024505 | NOX5 | 0.79 |
| NM_174892 | TREM5 | 0.79 |
| NM_000965 | RARB | 0.79 |
| NM_006320 | PGRMC2 | 0.79 |
| NM_005694 | COX17 | 0.79 |
| NM_006134 | C21ORF4 | 0.79 |
| NM_024015 | HOXB4 | 0.79 |
| NM_021195 | CLDN6 | 0.79 |
| NM_007365 | PADI2 | 0.79 |
| NM_019032 | TSRC1 | 0.79 |
| NM_016399 | HSPC132 | 0.79 |
| NM_020771 | HACE1 | 0.79 |
| NM_024691 | FLJ23233 | 0.79 |
| NM_176812 | C20ORF178 | 0.79 |
| NM_144968 | FLJ32783 | 0.79 |
| NM_152781 | FLJ32830 | 0.79 |
| NM_175893 | LOC283241 | 0.79 |
| NM_173688 | FLJ39630 | 0.79 |
| XM_380175 | MGC22265 | 0.79 |
| XM_374203 | LOC389469 | 0.79 |
| XM_373607 | LOC388014 | 0.79 |
| NM_000906 | NPR1 | 0.8 |
| NM_020993 | BCL7A | 0.8 |
| NM_033512 | TSPYL5 | 0.8 |
| NM_033068 | ACPT | 0.8 |
| NM_001876 | CPT1A | 0.8 |
| NM_020230 | PPAN | 0.8 |
| XM_377774 | KIF5C | 0.8 |
| NM_006111 | ACAA2 | 0.8 |
| NM_007349 | PAXIP1L | 0.8 |
| NM_014224 | PGA5 | 0.8 |
| NM_182762 | 7A5 | 0.8 |
| NM_003533 | HIST1H3I | 0.8 |
| XM_375485 | HELZ | 0.8 |
| NM_015162 | BG1 | 0.8 |
| NM_014478 | RCP9 | 0.8 |
| NM_058184 | C21ORF42 | 0.8 |
| NM_020245 | TULP4 | 0.8 |
| NM_021819 | LMAN1L | 0.8 |
| NM_024963 | FBXL18 | 0.8 |
| NM_032838 | ZNF566 | 0.8 |
| NM_153704 | MGC26979 | 0.8 |
| NM_198844 | ZPBP2 | 0.8 |
| NM_152503 | C20ORF132 | 0.8 |

| | | | | | |
|---|---|---|---|---|---|
| NM_181535 | KRT25D | 0.8 | NM_145024 | FLJ31547 | 0.8 |
| XM_373580 | LOC254028 | 0.8 | NM_178434 | LCE3C | 0.8 |
| NM_178571 | MGC51025 | 0.8 | NM_021066 | HIST1H2AJ | 0.8 |
| NM_003524 | HIST1H2BH | 0.8 | XM_291099 | LOC339977 | 0.8 |
| XM_209180 | LOC284397 | 0.8 | XM_208361 | LOC285900 | 0.8 |
| XM_372966 | LOC391462 | 0.8 | XM_375738 | LOC400757 | 0.8 |
| XM_374113 | LOC389271 | 0.8 | NM_001005513 | OR4C45 | 0.8 |
| NM_007023 | RAPGEF4 | 0.81 | NM_002436 | MPP1 | 0.81 |
| NM_012073 | CCT5 | 0.81 | NM_014079 | KLF15 | 0.81 |
| NM_006434 | SORBS1 | 0.81 | NM_022114 | PRDM16 | 0.81 |
| NM_014587 | SOX8 | 0.81 | NM_018310 | BRF2 | 0.81 |
| NM_018324 | THEDC1 | 0.81 | NM_003362 | UNG | 0.81 |
| NM_016109 | ANGPTL4 | 0.81 | NM_176875 | CCKBR | 0.81 |
| NM_005514 | HLA-B | 0.81 | NM_017761 | PNRC2 | 0.81 |
| NM_012201 | GLG1 | 0.81 | NM_016179 | TRPC4 | 0.81 |
| NM_000619 | IFNG | 0.81 | NM_005567 | LGALS3BP | 0.81 |
| NM_004774 | PPARBP | 0.81 | NM_005909 | MAP1B | 0.81 |
| NM_001670 | ARVCF | 0.81 | NM_031436 | AKR1CL2 | 0.81 |
| NM_006391 | IPO7 | 0.81 | NM_006071 | PKDREJ | 0.81 |
| NM_004065 | CDR1 | 0.81 | NM_001725 | BPI | 0.81 |
| NM_000085 | CLCNKB | 0.81 | NM_004628 | XPC | 0.81 |
| NM_004388 | CTBS | 0.81 | NM_001460 | FMO2 | 0.81 |
| NM_002407 | SCGB2A1 | 0.81 | NM_020133 | AGPAT4 | 0.81 |
| NM_020745 | AARSL | 0.81 | NM_003047 | SLC9A1 | 0.81 |
| NM_004627 | WRB | 0.81 | NM_005782 | THOC4 | 0.81 |
| NM_014888 | FAM3C | 0.81 | NM_006581 | FUT9 | 0.81 |
| NM_006809 | TOMM34 | 0.81 | NM_006657 | FTCD | 0.81 |
| NM_015558 | SS18L1 | 0.81 | NM_015596 | KLK13 | 0.81 |
| NM_014455 | ZNF364 | 0.81 | NM_022034 | CUZD1 | 0.81 |
| NM_020208 | SLC6A20 | 0.81 | NM_018064 | C6ORF166 | 0.81 |
| NM_018388 | MBNL3 | 0.81 | NM_020062 | SLC2A4RG | 0.81 |
| NM_020859 | SHRML | 0.81 | NM_031921 | ATAD3B | 0.81 |
| NM_032731 | TXNL5 | 0.81 | NM_032888 | COL27A1 | 0.81 |
| XM_036936 | KIAA1666 | 0.81 | NM_052858 | MRVLDC3 | 0.81 |
| XM_057040 | KIAA1922 | 0.81 | NM_138333 | C9ORF42 | 0.81 |
| XM_372578 | LOC123346 | 0.81 | XM_085261 | MESP2 | 0.81 |
| NM_152539 | FLJ32859 | 0.81 | NM_170725 | PGBD2 | 0.81 |
| XM_211805 | LOC285205 | 0.81 | NM_175907 | ZADH2 | 0.81 |
| NM_178148 | SLC35B2 | 0.81 | XM_208403 | LOC286310 | 0.81 |
| XM_379935 | LOC402601 | 0.81 | XM_380044 | LOC402695 | 0.81 |
| NM_001258 | CDK3 | 0.82 | NM_004670 | PAPSS2 | 0.82 |
| NM_003691 | STK16 | 0.82 | NM_014246 | CELSR1 | 0.82 |
| NM_002564 | P2RY2 | 0.82 | NM_173077 | CPO | 0.82 |
| NM_001910 | CTSE | 0.82 | NM_018449 | UBAP2 | 0.82 |
| XM_291241 | IPLA2(GAMMA) | 0.82 | NM_014248 | RBX1 | 0.82 |
| NM_021003 | PPM1A | 0.82 | NM_004257 | TGFBRAP1 | 0.82 |

| | | |
|---|---|---|
| NM_001496 | GFRA3 | 0.82 |
| NM_007045 | FGFR1OP | 0.82 |
| NM_017675 | PC-LKC | 0.82 |
| NM_006717 | SPIN | 0.82 |
| NM_003048 | SLC9A2 | 0.82 |
| NM_005868 | BET1 | 0.82 |
| NM_007215 | POLG2 | 0.82 |
| NM_001001560 | GGA1 | 0.82 |
| NM_014039 | PTD012 | 0.82 |
| NM_173160 | FXYD4 | 0.82 |
| XM_373170 | LOC54103 | 0.82 |
| NM_022094 | CIDE-3 | 0.82 |
| NM_053285 | TEKT1 | 0.82 |
| NM_032539 | SLITRK2 | 0.82 |
| NM_033261 | IDI2 | 0.82 |
| NM_152592 | C14ORF49 | 0.82 |
| NM_152662 | FLJ23867 | 0.82 |
| XM_292093 | LST-3 | 0.82 |
| XM_173160 | LOC255187 | 0.82 |
| NM_020397 | CAMK1D | 0.83 |
| NM_030883 | OR2H1 | 0.83 |
| NM_016932 | SIX2 | 0.83 |
| NM_003713 | PPAP2B | 0.83 |
| NM_002086 | GRB2 | 0.83 |
| NM_000393 | COL5A2 | 0.83 |
| NM_000655 | SELL | 0.83 |
| NM_001915 | CYB561 | 0.83 |
| XM_371116 | MYO5B | 0.83 |
| XM_376519 | ANKRD6 | 0.83 |
| NM_015973 | GAL | 0.83 |
| NM_018661 | DEFB103A | 0.83 |
| NM_020662 | MRS2L | 0.83 |
| NM_080670 | SLC35A4 | 0.83 |
| NM_054110 | GALNTL2 | 0.83 |
| XM_091331 | LOC162073 | 0.83 |
| NM_207385 | FLJ26184 | 0.83 |
| NM_173793 | LOC128977 | 0.83 |
| XM_373846 | LOC388639 | 0.83 |
| XM_375424 | LOC400586 | 0.83 |
| NM_017607 | PPP1R12C | 0.84 |
| NM_003682 | MADD | 0.84 |
| NM_052931 | SLAMF6 | 0.84 |
| NM_005628 | SLC1A5 | 0.84 |
| XM_375593 | ZNF536 | 0.84 |
| NM_012208 | HARSL | 0.84 |
| NM_013352 | SART2 | 0.84 |
| NM_001124 | ADM | 0.82 |
| NM_006495 | EVI2B | 0.82 |
| NM_014059 | RGC32 | 0.82 |
| NM_001281 | CKAP1 | 0.82 |
| NM_003054 | SLC18A2 | 0.82 |
| NM_014765 | TOMM20 | 0.82 |
| NM_014929 | KIAA0971 | 0.82 |
| NM_014037 | SLC6A16 | 0.82 |
| NM_016563 | RASL12 | 0.82 |
| NM_019038 | TDRD4 | 0.82 |
| NM_020212 | LOC56964 | 0.82 |
| NM_024313 | MGC3731 | 0.82 |
| NM_032258 | TBC1D3 | 0.82 |
| NM_052844 | WDR34 | 0.82 |
| NM_138817 | SLC7A13 | 0.82 |
| XM_378628 | MGC23284 | 0.82 |
| NM_153020 | RBM24 | 0.82 |
| XM_208312 | LOC285296 | 0.82 |
| XM_371825 | LOC389396 | 0.82 |
| NM_003816 | ADAM9 | 0.83 |
| NM_145805 | ISL2 | 0.83 |
| NM_005670 | EPM2A | 0.83 |
| NM_018402 | IL26 | 0.83 |
| NM_003373 | VCL | 0.83 |
| NM_004050 | BCL2L2 | 0.83 |
| NM_001311 | CRIP1 | 0.83 |
| NM_004468 | FHL3 | 0.83 |
| NM_004710 | SYNGR2 | 0.83 |
| NM_014573 | MAC30 | 0.83 |
| NM_017877 | C2ORF18 | 0.83 |
| NM_020456 | C13ORF1 | 0.83 |
| XM_098368 | KCTD16 | 0.83 |
| NM_080661 | MGC15937 | 0.83 |
| NM_080747 | K6IRS2 | 0.83 |
| XM_373810 | LOC199800 | 0.83 |
| NM_206838 | LOC390511 | 0.83 |
| XM_373855 | LOC388655 | 0.83 |
| XM_378203 | LOC399708 | 0.83 |
| NM_004051 | BDH | 0.84 |
| NM_000553 | WRN | 0.84 |
| XM_371358 | OR2M4 | 0.84 |
| NM_002493 | NDUFB6 | 0.84 |
| NM_003917 | AP1G2 | 0.84 |
| NM_006838 | METAP2 | 0.84 |
| NM_134264 | WSB1 | 0.84 |
| XM_041116 | C14ORF171 | 0.84 |

| | | |
|---|---|---|
| NM_020689 | SLC24A3 | 0.84 |
| NM_052840 | BRUNOL6 | 0.84 |
| NM_022818 | MAP1LC3B | 0.84 |
| NM_032814 | FLJ14627 | 0.84 |
| NM_033445 | HIST3H2A | 0.84 |
| NM_152330 | C14ORF31 | 0.84 |
| XM_117174 | SLC5A9 | 0.84 |
| XM_209111 | LOC284307 | 0.84 |
| NM_012315 | KLK9 | 0.84 |
| NM_173653 | SLC9A9 | 0.84 |
| NM_201548 | RP26 | 0.84 |
| XM_379298 | LOC401164 | 0.84 |
| XM_373498 | LOC387764 | 0.84 |
| NM_006251 | PRKAA1 | 0.85 |
| NM_012424 | RPS6KC1 | 0.85 |
| NM_000842 | GRM5 | 0.85 |
| NM_017884 | PINX1 | 0.85 |
| NM_016361 | ACP6 | 0.85 |
| NM_001510 | GRID2 | 0.85 |
| NM_017823 | DUSP23 | 0.85 |
| NM_024317 | ILT10 | 0.85 |
| NM_003055 | SLC18A3 | 0.85 |
| NM_003458 | BSN | 0.85 |
| NM_006812 | OS-9 | 0.85 |
| NM_013275 | ANKRD11 | 0.85 |
| NM_016401 | HSPC138 | 0.85 |
| NM_020375 | C12ORF5 | 0.85 |
| NM_024613 | PLEKHF2 | 0.85 |
| NM_033035 | TSLP | 0.85 |
| XM_290865 | LOC163233 | 0.85 |
| NM_152682 | MGC10198 | 0.85 |
| XM_373603 | C14ORF86 | 0.85 |
| NM_198496 | AMACO | 0.85 |
| XM_210334 | LOC284064 | 0.85 |
| NM_005975 | PTK6 | 0.86 |
| NM_003554 | OR1E2 | 0.86 |
| NM_021951 | DMRT1 | 0.86 |
| NM_001068 | TOP2B | 0.86 |
| NM_021096 | CACNA1I | 0.86 |
| NM_001197 | BIK | 0.86 |
| NM_006432 | NPC2 | 0.86 |
| NM_002217 | ITIH3 | 0.86 |
| NM_079420 | MYL1 | 0.86 |
| NM_002670 | PLS1 | 0.86 |
| NM_001054 | SULT1A2 | 0.86 |
| NM_012240 | SIRT4 | 0.86 |
| NM_020936 | DDX55 | 0.84 |
| NM_024536 | CHPF | 0.84 |
| NM_032246 | RKHD3 | 0.84 |
| NM_138348 | LOC90268 | 0.84 |
| NM_016311 | ATPIF1 | 0.84 |
| NM_145176 | SLC2A12 | 0.84 |
| NM_198991 | KCTD1 | 0.84 |
| NM_174952 | MGC46496 | 0.84 |
| NM_182586 | FLJ33534 | 0.84 |
| XM_294993 | LOC339529 | 0.84 |
| NM_203451 | LOC400120 | 0.84 |
| XM_378054 | LOC402360 | 0.84 |
| NM_005476 | GNE | 0.85 |
| NM_002227 | JAK1 | 0.85 |
| NM_000647 | CCR2 | 0.85 |
| NM_005314 | GRPR | 0.85 |
| NM_002735 | PRKAR1B | 0.85 |
| NM_003194 | TBP | 0.85 |
| NM_002118 | HLA-DMB | 0.85 |
| NM_000077 | CDKN2A | 0.85 |
| NM_053276 | VIT | 0.85 |
| NM_003619 | PRSS12 | 0.85 |
| NM_014711 | CP110 | 0.85 |
| NM_015091 | KIAA0423 | 0.85 |
| NM_013357 | PURG | 0.85 |
| NM_017781 | FLJ20359 | 0.85 |
| NM_032512 | PDZK4 | 0.85 |
| NM_024528 | NKAP | 0.85 |
| XM_098238 | DKFZP434D0215 | 0.85 |
| NM_144988 | MGC19780 | 0.85 |
| NM_139319 | SLC17A8 | 0.85 |
| NM_181786 | HKR1 | 0.85 |
| XM_498131 | LOC442247 | 0.85 |
| XM_378564 | LOC400500 | 0.85 |
| NM_000621 | HTR2A | 0.86 |
| NM_014522 | PCDH11X | 0.86 |
| NM_006236 | POU3F3 | 0.86 |
| NM_013315 | TPTE | 0.86 |
| NM_001630 | ANXA8 | 0.86 |
| NM_002294 | LAMP2 | 0.86 |
| NM_021019 | MYL6 | 0.86 |
| NM_006151 | LPO | 0.86 |
| NM_145804 | ABTB2 | 0.86 |
| NM_006978 | ZNF183 | 0.86 |
| NM_014745 | FAM38A | 0.86 |
| NM_015444 | RIS1 | 0.86 |

| | | |
|---|---|---|
| NM_015698 | GPKOW | 0.86 |
| NM_016379 | VCX3 | 0.86 |
| NM_017845 | COMMD8 | 0.86 |
| NM_020412 | CHMP1.5 | 0.86 |
| NM_032221 | CHD6 | 0.86 |
| NM_032857 | LACTB | 0.86 |
| NM_178834 | LOC143903 | 0.86 |
| NM_152620 | TRIM60 | 0.86 |
| NM_201546 | LOC200008 | 0.86 |
| NM_172138 | IL28A | 0.86 |
| NM_006382 | CDRT1 | 0.86 |
| XM_373839 | LOC388622 | 0.86 |
| XM_373303 | LOC392360 | 0.86 |
| XM_373904 | LOC388780 | 0.86 |
| NM_022726 | ELOVL4 | 0.87 |
| NM_004296 | RGS6 | 0.87 |
| NM_005611 | RBL2 | 0.87 |
| NM_014620 | HOXC4 | 0.87 |
| NM_001546 | ID4 | 0.87 |
| NM_006022 | TGFB1I4 | 0.87 |
| NM_005763 | AASS | 0.87 |
| NM_015052 | NEDL1 | 0.87 |
| NM_021258 | IL22RA1 | 0.87 |
| NM_000358 | TGFBI | 0.87 |
| NM_001886 | CRYBA4 | 0.87 |
| NM_020366 | RPGRIP1 | 0.87 |
| NM_004587 | RRBP1 | 0.87 |
| NM_005752 | CLECSF1 | 0.87 |
| NM_015358 | ZCWCC3 | 0.87 |
| NM_014442 | SIGLEC8 | 0.87 |
| NM_017743 | DPP8 | 0.87 |
| NM_018263 | ASXL2 | 0.87 |
| NM_020944 | GBA2 | 0.87 |
| NM_024861 | FLJ22671 | 0.87 |
| NM_080651 | THRAP6 | 0.87 |
| NM_152905 | NEDD1 | 0.87 |
| XM_375353 | LOC146481 | 0.87 |
| NM_138499 | PWWP2 | 0.87 |
| NM_032895 | MGC14376 | 0.87 |
| XM_293924 | LOC345651 | 0.87 |
| XM_374300 | LOC389777 | 0.87 |
| XM_373914 | LOC388796 | 0.87 |
| XM_373953 | LOC388890 | 0.87 |
| NM_001005465 | OR10G3 | 0.87 |
| NM_004439 | EPHA5 | 0.88 |
| NM_003453 | ZNF198 | 0.88 |
| NM_015720 | PODLX2 | 0.86 |
| NM_017515 | SLC35F2 | 0.86 |
| NM_018183 | SBNO1 | 0.86 |
| NM_022757 | FLJ12892 | 0.86 |
| NM_033548 | MGC12518 | 0.86 |
| NM_015230 | CENTD1 | 0.86 |
| XM_114000 | ANKRD24 | 0.86 |
| NM_173544 | BCNP1 | 0.86 |
| NM_153610 | CMYA5 | 0.86 |
| NM_175898 | LOC283687 | 0.86 |
| XM_066534 | LOC139189 | 0.86 |
| XM_373027 | LOC391656 | 0.86 |
| XM_378421 | LOC400144 | 0.86 |
| NM_002750 | MAPK8 | 0.87 |
| NM_006018 | GPR109B | 0.87 |
| NM_001419 | ELAVL1 | 0.87 |
| NM_000330 | RS1 | 0.87 |
| NM_004506 | HSF2 | 0.87 |
| NM_003140 | SRY | 0.87 |
| NM_003244 | TGIF | 0.87 |
| NM_006810 | PDIR | 0.87 |
| NM_005167 | PPP2CZ | 0.87 |
| NM_020530 | OSM | 0.87 |
| NM_020897 | HCN3 | 0.87 |
| NM_000143 | FH | 0.87 |
| NM_005956 | MTHFD1 | 0.87 |
| NM_003976 | ARTN | 0.87 |
| NM_033178 | DUX4 | 0.87 |
| NM_013451 | FER1L3 | 0.87 |
| NM_019092 | KIAA1164 | 0.87 |
| NM_018122 | FLJ10514 | 0.87 |
| XM_374080 | KIAA0114 | 0.87 |
| NM_021257 | NGB | 0.87 |
| NM_030787 | FHR5 | 0.87 |
| NM_170710 | WDR17 | 0.87 |
| NM_145261 | TIM14 | 0.87 |
| NM_144697 | LOC148523 | 0.87 |
| NM_152718 | FLJ32009 | 0.87 |
| XM_208990 | LOC284067 | 0.87 |
| XM_377820 | LOC402152 | 0.87 |
| XM_374842 | FLJ37035 | 0.87 |
| XM_375963 | LOC400891 | 0.87 |
| XM_373347 | LOC392465 | 0.87 |
| NM_024838 | THNSL1 | 0.88 |
| NM_002958 | RYK | 0.88 |
| NM_030791 | SGPP1 | 0.88 |

| | | |
|---|---|---|
| NM_006003 | UQCRFS1 | 0.88 |
| NM_004162 | RAB5A | 0.88 |
| NM_014271 | IL1RAPL1 | 0.88 |
| NM_001621 | AHR | 0.88 |
| NM_004787 | SLIT2 | 0.88 |
| NM_000219 | KCNE1 | 0.88 |
| NM_000852 | GSTP1 | 0.88 |
| NM_001815 | CEACAM3 | 0.88 |
| NM_004769 | ACCN3 | 0.88 |
| NM_004172 | SLC1A3 | 0.88 |
| NM_002776 | KLK10 | 0.88 |
| NM_014679 | KIAA0092 | 0.88 |
| NM_007195 | POLI | 0.88 |
| NM_012081 | ELL2 | 0.88 |
| NM_022003 | FXYD6 | 0.88 |
| NM_018687 | LOC55908 | 0.88 |
| NM_024341 | ZNF557 | 0.88 |
| XM_291315 | KIAA1815 | 0.88 |
| NM_032376 | MGC4251 | 0.88 |
| NM_138476 | MGC5987 | 0.88 |
| NM_152666 | FLJ40773 | 0.88 |
| NM_198508 | FLJ44186 | 0.88 |
| XM_114166 | LOC200230 | 0.88 |
| XM_379102 | LOC400960 | 0.88 |
| XM_374111 | LOC389269 | 0.88 |
| XM_378631 | LOC400558 | 0.88 |
| NM_006206 | PDGFRA | 0.89 |
| NM_002648 | PIM1 | 0.89 |
| NM_000329 | RPE65 | 0.89 |
| NM_000308 | PPGB | 0.89 |
| NM_022336 | EDAR | 0.89 |
| NM_003810 | TNFSF10 | 0.89 |
| NM_001765 | CD1C | 0.89 |
| NM_004221 | NK4 | 0.89 |
| NM_198270 | NHS | 0.89 |
| NM_003108 | SOX11 | 0.89 |
| NM_004801 | NRXN1 | 0.89 |
| NM_014705 | DOCK4 | 0.89 |
| NM_014714 | KIAA0590 | 0.89 |
| NM_013244 | HGNT-IV-H | 0.89 |
| XM_039495 | D15WSU75E | 0.89 |
| NM_017735 | FLJ20272 | 0.89 |
| NM_031309 | SCRT1 | 0.89 |
| NM_182491 | LOC90637 | 0.89 |
| NM_080821 | C20ORF108 | 0.89 |
| XM_096688 | LOC144920 | 0.89 |
| NM_006270 | RRAS | 0.88 |
| NM_014606 | HERC3 | 0.88 |
| NM_014339 | IL17R | 0.88 |
| NM_002280 | KRTHA5 | 0.88 |
| NM_004572 | PKP2 | 0.88 |
| NM_181334 | ARHGAP8 | 0.88 |
| NM_003550 | MAD1L1 | 0.88 |
| NM_001109 | ADAM8 | 0.88 |
| NM_006796 | AFG3L2 | 0.88 |
| NM_152328 | ADSSL1 | 0.88 |
| NM_014646 | LPIN2 | 0.88 |
| NM_014724 | ZNF305 | 0.88 |
| NM_006684 | FHR-4 | 0.88 |
| NM_015942 | CGI-12 | 0.88 |
| XM_085929 | MEIS3 | 0.88 |
| NM_007008 | RTN4 | 0.88 |
| NM_024828 | C9ORF82 | 0.88 |
| NM_032872 | SYTL1 | 0.88 |
| NM_138700 | TRIM40 | 0.88 |
| NM_138492 | MGC21644 | 0.88 |
| NM_152766 | MGC40107 | 0.88 |
| XM_060104 | LOC126637 | 0.88 |
| XM_117514 | LOC203235 | 0.88 |
| XM_372941 | LOC391405 | 0.88 |
| XM_377230 | LOC401703 | 0.88 |
| NM_001005238 | OR51G2 | 0.88 |
| NM_004690 | LATS1 | 0.89 |
| NM_152430 | MGC24137 | 0.89 |
| NM_013266 | CTNNA3 | 0.89 |
| NM_016188 | ACTL6 | 0.89 |
| NM_006249 | PRB3 | 0.89 |
| NM_016929 | CLIC5 | 0.89 |
| NM_032839 | DIRC2 | 0.89 |
| NM_003150 | STAT3 | 0.89 |
| NM_004593 | SFRS10 | 0.89 |
| NM_004875 | POLR1C | 0.89 |
| NM_014649 | SAFB2 | 0.89 |
| XM_049380 | KIAA0339 | 0.89 |
| NM_014610 | GANAB | 0.89 |
| NM_014180 | MRPL22 | 0.89 |
| NM_016404 | HSPC152 | 0.89 |
| NM_021942 | FLJ12716 | 0.89 |
| NM_152903 | KBTBD6 | 0.89 |
| XM_056680 | LOC115749 | 0.89 |
| NM_138792 | LOC123169 | 0.89 |
| NM_080749 | NEURL2 | 0.89 |

| | | | | | |
|---|---|---|---|---|---|
| NM_138573 | LOC145957 | 0.89 | NM_152491 | FLJ32569 | 0.89 |
| XM_371267 | LOC164045 | 0.89 | NM_173528 | FLJ38615 | 0.89 |
| NM_152695 | FLJ23614 | 0.89 | NM_173677 | FLJ40852 | 0.89 |
| XM_047554 | LOC148198 | 0.89 | XM_166820 | LOC219464 | 0.89 |
| XM_376257 | LOC401082 | 0.89 | XM_375746 | LOC400759 | 0.89 |
| XM_374161 | LOC389369 | 0.89 | XM_372466 | LOC390335 | 0.89 |
| XM_377475 | LOC401883 | 0.89 | NM_004438 | EPHA4 | 0.9 |
| NM_032422 | GPR123 | 0.9 | NM_002032 | FTH1 | 0.9 |
| NM_012225 | NUBP2 | 0.9 | NM_004992 | MECP2 | 0.9 |
| NM_000303 | PMM2 | 0.9 | NM_018677 | ACAS2 | 0.9 |
| NM_005264 | GFRA1 | 0.9 | NM_005856 | RAMP3 | 0.9 |
| NM_130785 | TPTE2 | 0.9 | NM_014440 | IL1F6 | 0.9 |
| NM_001252 | TNFSF7 | 0.9 | NM_000350 | ABCA4 | 0.9 |
| NM_012142 | CCNDBP1 | 0.9 | NM_004791 | ITGBL1 | 0.9 |
| NM_014845 | KIAA0274 | 0.9 | NM_005257 | GATA6 | 0.9 |
| NM_012083 | FRAT2 | 0.9 | NM_000715 | C4BPA | 0.9 |
| NM_005181 | CA3 | 0.9 | NM_005599 | NHLH2 | 0.9 |
| NM_006933 | SLC5A3 | 0.9 | NM_006532 | ELL | 0.9 |
| NM_005667 | RNF103 | 0.9 | NM_014260 | HKE2 | 0.9 |
| NM_015959 | TMX2 | 0.9 | NM_016033 | CGI-90 | 0.9 |
| NM_017545 | HAO1 | 0.9 | NM_018375 | SLC39A9 | 0.9 |
| NM_207306 | KIAA0495 | 0.9 | NM_020415 | RETN | 0.9 |
| XM_378197 | FLJ14464 | 0.9 | NM_080672 | PHACTR3 | 0.9 |
| NM_152352 | C18ORF19 | 0.9 | NM_181522 | WFDC3 | 0.9 |
| NM_138818 | C9ORF65 | 0.9 | NM_198464 | UNQ9391 | 0.9 |
| XM_294751 | LOC338949 | 0.9 | XM_291346 | LOC340591 | 0.9 |
| XM_379200 | LOC401078 | 0.9 | XM_371714 | LOC389239 | 0.9 |
| XM_379858 | LOC402539 | 0.9 | XM_373671 | LOC388231 | 0.9 |
| NM_001052 | SSTR4 | 0.91 | NM_023921 | TAS2R10 | 0.91 |
| NM_021979 | HSPA2 | 0.91 | NM_000328 | RPGR | 0.91 |
| NM_003655 | CBX4 | 0.91 | NM_005606 | LGMN | 0.91 |
| NM_058176 | HDAC9 | 0.91 | NM_004290 | RNF14 | 0.91 |
| NM_006378 | SEMA4D | 0.91 | NM_003307 | TRPM2 | 0.91 |
| NM_000033 | ABCD1 | 0.91 | NM_014350 | TNFAIP8 | 0.91 |
| NM_153257 | GIOT-1 | 0.91 | NM_000897 | LTC4S | 0.91 |
| NM_001293 | CLNS1A | 0.91 | NM_174930 | PMS2L5 | 0.91 |
| XM_290670 | LOC23117 | 0.91 | XM_371761 | KIAA0825 | 0.91 |
| NM_015180 | SYNE2 | 0.91 | NM_018149 | FLJ10587 | 0.91 |
| NM_017664 | ANKRD10 | 0.91 | NM_032146 | ARL6 | 0.91 |
| NM_032211 | LOXL4 | 0.91 | NM_032874 | KIAA1984 | 0.91 |
| NM_138773 | LOC91137 | 0.91 | NM_153013 | FLJ30596 | 0.91 |
| NM_173167 | CMYA4 | 0.91 | XM_212581 | ZNF311 | 0.91 |
| XM_379288 | LOC340017 | 0.91 | NM_207399 | FLJ36116 | 0.91 |
| XM_291017 | LOC339793 | 0.91 | XM_291716 | LOC340784 | 0.91 |
| XM_296117 | LOC341333 | 0.91 | XM_374058 | LOC389162 | 0.91 |
| XM_372002 | LOC389599 | 0.91 | XM_373573 | LOC387942 | 0.91 |

| | | | | | |
|---|---|---|---|---|---|
| XM_372576 | LOC390598 | 0.91 | XM_377445 | LOC401860 | 0.91 |
| XM_372689 | LOC390828 | 0.91 | NM_005109 | OSR1 | 0.92 |
| NM_006785 | MALT1 | 0.92 | NM_003468 | FZD5 | 0.92 |
| NM_005683 | GPR55 | 0.92 | NM_015355 | JJAZ1 | 0.92 |
| NM_003371 | VAV2 | 0.92 | NM_003078 | SMARCD3 | 0.92 |
| NM_002027 | FNTA | 0.92 | NM_003873 | NRP1 | 0.92 |
| NM_002910 | RENBP | 0.92 | NM_006092 | CARD4 | 0.92 |
| NM_002120 | HLA-DOB | 0.92 | NM_002851 | PTPRZ1 | 0.92 |
| NM_002192 | INHBA | 0.92 | NM_001855 | COL15A1 | 0.92 |
| NM_020939 | CPNE5 | 0.92 | NM_052845 | MMAB | 0.92 |
| NM_000432 | MYL2 | 0.92 | NM_003324 | TULP3 | 0.92 |
| NM_004889 | ATP5J2 | 0.92 | NM_007185 | TNRC4 | 0.92 |
| NM_152280 | SYT11 | 0.92 | NM_017741 | FLJ20280 | 0.92 |
| NM_018651 | ZNF167 | 0.92 | NM_022911 | SLC26A6 | 0.92 |
| NM_032496 | ARHGAP9 | 0.92 | NM_024656 | FLJ22329 | 0.92 |
| NM_024661 | FLJ12436 | 0.92 | NM_025026 | FLJ14107 | 0.92 |
| NM_032434 | ZNF512 | 0.92 | NM_052905 | FMNL2 | 0.92 |
| NM_152390 | MGC33926 | 0.92 | NM_153347 | FLJ90119 | 0.92 |
| NM_153027 | FLJ31659 | 0.92 | NM_153607 | LOC153222 | 0.92 |
| NM_138575 | MGC5352 | 0.92 | NM_178276 | C5ORF12 | 0.92 |
| NM_174943 | FLJ25976 | 0.92 | XM_212170 | LOC286094 | 0.92 |
| NM_198722 | AMIGO3 | 0.92 | XM_166971 | LOC220416 | 0.92 |
| XM_017374 | LOC150580 | 0.92 | XM_293354 | LOC347442 | 0.92 |
| XM_377630 | LOC401983 | 0.92 | XM_372161 | LOC389816 | 0.92 |
| XM_378511 | LOC400368 | 0.92 | XM_379041 | LOC400920 | 0.92 |
| XM_496820 | LOC441165 | 0.92 | NM_002612 | PDK4 | 0.93 |
| NM_000823 | GHRHR | 0.93 | NM_022124 | CDH23 | 0.93 |
| NM_052949 | RASGRP4 | 0.93 | NM_030751 | TCF8 | 0.93 |
| NM_003343 | UBE2G2 | 0.93 | NM_021197 | WFDC1 | 0.93 |
| NM_004308 | ARHGAP1 | 0.93 | NM_006611 | KLRA1 | 0.93 |
| NM_014812 | KAB | 0.93 | NM_015322 | FEM1B | 0.93 |
| NM_002026 | FN1 | 0.93 | NM_001845 | COL4A1 | 0.93 |
| NM_000698 | ALOX5 | 0.93 | NM_014386 | PKD2L2 | 0.93 |
| NM_018173 | FLJ10665 | 0.93 | NM_001540 | HSPB1 | 0.93 |
| NM_004174 | SLC9A3 | 0.93 | NM_153332 | 3'HEXO | 0.93 |
| XM_091895 | ZNF17 | 0.93 | NM_003943 | GENX-3414 | 0.93 |
| NM_014280 | DNAJC8 | 0.93 | NM_012123 | MTO1 | 0.93 |
| XM_166479 | KIAA0240 | 0.93 | NM_014955 | CGI-01 | 0.93 |
| NM_020162 | DHX33 | 0.93 | NM_020198 | GK001 | 0.93 |
| NM_020123 | SMBP | 0.93 | NM_021190 | PTBP2 | 0.93 |
| NM_024023 | UNKL | 0.93 | NM_024902 | FLJ13236 | 0.93 |
| NM_031209 | QTRT1 | 0.93 | NM_032047 | B3GNT5 | 0.93 |
| NM_032565 | EBPL | 0.93 | NM_033105 | DNAJC5B | 0.93 |
| XM_030958 | LOC90333 | 0.93 | NM_080429 | AQP10 | 0.93 |
| NM_052904 | KIAA1900 | 0.93 | NM_052953 | LRP15 | 0.93 |
| NM_178826 | TMEM16D | 0.93 | NM_175575 | WFIKKNRP | 0.93 |

| | | | | | |
|---|---|---|---|---|---|
| NM_133265 | AMOT | 0.93 | NM_173560 | RFXDC1 | 0.93 |
| NM_178815 | ARL8 | 0.93 | NM_153261 | FLJ38101 | 0.93 |
| NM_173644 | FLJ33860 | 0.93 | NM_175619 | ZAR1 | 0.93 |
| NM_203494 | USP50 | 0.93 | NM_199349 | LOC375616 | 0.93 |
| XM_376068 | LOC400962 | 0.93 | XM_379173 | LOC401050 | 0.93 |
| XM_371486 | LOC388937 | 0.93 | XM_377933 | LOC402251 | 0.93 |
| XM_380077 | LOC402720 | 0.93 | XM_372122 | LOC389765 | 0.93 |
| XM_379539 | LOC401440 | 0.93 | NM_181657 | LTB4R | 0.94 |
| NM_000906 | NPR1 | 0.94 | NM_000872 | HTR7 | 0.94 |
| NM_001882 | CRHBP | 0.94 | NM_002920 | RFX4 | 0.94 |
| NM_020974 | SCUBE2 | 0.94 | NM_013320 | HCFC2 | 0.94 |
| NM_014500 | HTATSF1 | 0.94 | NM_000771 | CYP2C9 | 0.94 |
| NM_019093 | UGT1A3 | 0.94 | NM_005802 | TOPORS | 0.94 |
| NM_003644 | GAS7 | 0.94 | NM_033553 | GUCA2A | 0.94 |
| NM_022169 | ABCG4 | 0.94 | NM_013380 | ZNF228 | 0.94 |
| NM_003784 | SERPINB7 | 0.94 | XM_375074 | KIAA0391 | 0.94 |
| NM_017586 | C9ORF7 | 0.94 | NM_015465 | GEMIN5 | 0.94 |
| NM_014077 | FAM32A | 0.94 | NM_016025 | DREV1 | 0.94 |
| XM_166138 | ANKRD16 | 0.94 | NM_017830 | OCIA | 0.94 |
| NM_023036 | DNAI2 | 0.94 | NM_031207 | HT036 | 0.94 |
| NM_032589 | DSCR8 | 0.94 | NM_033201 | BC008967 | 0.94 |
| NM_145283 | C9ORF121 | 0.94 | XM_113696 | LOC196337 | 0.94 |
| XM_090844 | TMEM30B | 0.94 | NM_173609 | C15ORF21 | 0.94 |
| NM_207475 | FLJ90680 | 0.94 | XM_091830 | LOC162835 | 0.94 |
| XM_372762 | LOC391002 | 0.94 | XM_374067 | LOC389185 | 0.94 |
| XM_380148 | LOC402598 | 0.94 | XM_376774 | LOC401460 | 0.94 |
| XM_380121 | LOC402538 | 0.94 | XM_374880 | LOC399888 | 0.94 |
| NM_001005515 | OR5H15 | 0.94 | NM_004938 | DAPK1 | 0.95 |
| NM_006871 | RIPK3 | 0.95 | NM_153834 | GPR112 | 0.95 |
| NM_024012 | HTR5A | 0.95 | NM_022436 | ABCG5 | 0.95 |
| NM_022131 | CLSTN2 | 0.95 | NM_002155 | HSPA6 | 0.95 |
| NM_005098 | MSC | 0.95 | NM_002022 | FMO4 | 0.95 |
| NM_004190 | LIPF | 0.95 | NM_004663 | RAB11A | 0.95 |
| NM_153012 | TNFSF12 | 0.95 | NM_000939 | POMC | 0.95 |
| NM_005679 | TAF1C | 0.95 | NM_001966 | EHHADH | 0.95 |
| NM_004738 | VAPB | 0.95 | NM_004280 | EEF1E1 | 0.95 |
| NM_003826 | NAPG | 0.95 | XM_045712 | PDZK10 | 0.95 |
| NM_007261 | CMRF-35H | 0.95 | XM_046808 | NFASC | 0.95 |
| XM_041964 | KIAA0523 | 0.95 | NM_014296 | CAPN7 | 0.95 |
| NM_014476 | PDLIM3 | 0.95 | NM_019023 | PRMT7 | 0.95 |
| NM_017802 | FLJ20397 | 0.95 | NM_017974 | APG16L | 0.95 |
| NM_017740 | ZDHHC7 | 0.95 | NM_018914 | PCDHGA11 | 0.95 |
| NM_024605 | FLJ20896 | 0.95 | NM_032132 | NOHMA | 0.95 |
| NM_032355 | MGC13272 | 0.95 | NM_178124 | LOC91966 | 0.95 |
| NM_152269 | FLJ38663 | 0.95 | NM_182511 | CBLN2 | 0.95 |
| NM_130387 | ASB14 | 0.95 | XM_086761 | LOC150084 | 0.95 |

| | | | | | |
|---|---|---|---|---|---|
| NM_153358 | FLJ90396 | 0.95 | NM_144973 | MGC24039 | 0.95 |
| NM_181720 | LOC257106 | 0.95 | NM_178514 | LOC283487 | 0.95 |
| XM_379011 | C21ORF130 | 0.95 | NM_173681 | NOS3AS | 0.95 |
| XM_371540 | UNQ2430 | 0.95 | NM_198520 | FLJ44112 | 0.95 |
| XM_208080 | LOC283368 | 0.95 | XM_376056 | LOC400954 | 0.95 |
| XM_379174 | LOC401051 | 0.95 | XM_372396 | LOC390158 | 0.95 |
| NM_003836 | DLK1 | 0.96 | NM_018660 | ZNF395 | 0.96 |
| NM_000598 | IGFBP3 | 0.96 | NM_003019 | SFTPD | 0.96 |
| NM_002658 | PLAU | 0.96 | NM_017582 | UBE2Q | 0.96 |
| NM_014555 | TRPM5 | 0.96 | NM_002834 | PTPN11 | 0.96 |
| NM_080679 | COL11A2 | 0.96 | NM_001156 | ANXA7 | 0.96 |
| NM_006891 | CRYGD | 0.96 | NM_015895 | GMNN | 0.96 |
| NM_005113 | GOLGA5 | 0.96 | NM_012365 | OR2A5 | 0.96 |
| NM_001899 | CST4 | 0.96 | NM_002241 | KCNJ10 | 0.96 |
| NM_006122 | MAN2A2 | 0.96 | NM_005930 | MGEA6 | 0.96 |
| NM_004602 | STAU | 0.96 | NM_005660 | SLC35A2 | 0.96 |
| NM_003562 | SLC25A11 | 0.96 | NM_004808 | NMT2 | 0.96 |
| NM_003803 | MYOM1 | 0.96 | NM_005875 | GC20 | 0.96 |
| NM_007057 | ZWINT | 0.96 | NM_007190 | SEC23IP | 0.96 |
| NM_015047 | KIAA0090 | 0.96 | NM_015433 | DKFZP586D0919 | 0.96 |
| NM_016494 | LOC51255 | 0.96 | NM_019065 | EFCBP2 | 0.96 |
| NM_017578 | ROPN1 | 0.96 | NM_018003 | UACA | 0.96 |
| NM_198271 | LMOD3 | 0.96 | NM_018845 | LOC55974 | 0.96 |
| NM_022467 | CHST8 | 0.96 | NM_015513 | CRELD1 | 0.96 |
| NM_022143 | LRRC4 | 0.96 | NM_025261 | LY6G6C | 0.96 |
| NM_024493 | ZNF306 | 0.96 | NM_031427 | C14ORF168 | 0.96 |
| NM_031461 | LOC83690 | 0.96 | NM_033185 | KRTAP3-3 | 0.96 |
| XM_376186 | LOC93463 | 0.96 | NM_153344 | C6ORF141 | 0.96 |
| NM_153225 | RPESP | 0.96 | NM_145287 | ZNF519 | 0.96 |
| NM_198403 | PAQR10 | 0.96 | NM_173617 | FLJ36701 | 0.96 |
| NM_173649 | FLJ40172 | 0.96 | XM_293745 | LOC345222 | 0.96 |
| XM_117548 | LOC203413 | 0.96 | XM_290185 | LOC338598 | 0.96 |
| XM_372958 | LOC391445 | 0.96 | XM_372371 | LOC390084 | 0.96 |
| XM_372591 | LOC390638 | 0.96 | NM_001006604 | LOC387646 | 0.96 |
| NM_000623 | BDKRB2 | 0.97 | NM_000188 | HK1 | 0.97 |
| NM_031866 | FZD8 | 0.97 | NM_000933 | PLCB4 | 0.97 |
| NM_013274 | POLL | 0.97 | NM_003141 | SSA1 | 0.97 |
| NM_015074 | KIF1B | 0.97 | NM_002127 | HLA-G | 0.97 |
| NM_000926 | PGR | 0.97 | NM_014685 | HERPUD1 | 0.97 |
| NM_014808 | FARP2 | 0.97 | NM_000231 | SGCG | 0.97 |
| NM_000711 | BGLAP | 0.97 | NM_001143 | AMELY | 0.97 |
| NM_000992 | RPL29 | 0.97 | NM_024855 | ACTR5 | 0.97 |
| NM_032604 | ABHD1 | 0.97 | NM_014797 | ZBTB24 | 0.97 |
| NM_005768 | C3F | 0.97 | NM_014343 | CLDN15 | 0.97 |
| NM_017770 | ELOVL2 | 0.97 | NM_018382 | FLJ11292 | 0.97 |
| NM_018355 | ZNF415 | 0.97 | XM_166320 | KIAA1553 | 0.97 |

| | | |
|---|---|---|
| NM_021803 | IL21 | 0.97 |
| NM_030934 | C1ORF25 | 0.97 |
| NM_152304 | MGC45806 | 0.97 |
| NM_172364 | CACNA2D4 | 0.97 |
| NM_014068 | PSORS1C1 | 0.97 |
| NM_182580 | CYB561D1 | 0.97 |
| NM_199343 | FLJ90637 | 0.97 |
| XM_375747 | LOC400760 | 0.97 |
| XM_379656 | LOC401543 | 0.97 |
| NM_018890 | RAC1 | 0.98 |
| NM_004821 | HAND1 | 0.98 |
| NM_002770 | PRSS2 | 0.98 |
| NM_005751 | AKAP9 | 0.98 |
| NM_018647 | TNFRSF19 | 0.98 |
| NM_001631 | ALPI | 0.98 |
| NM_003018 | SFTPC | 0.98 |
| NM_004171 | SLC1A2 | 0.98 |
| NM_014870 | KIAA0478 | 0.98 |
| NM_014959 | CARD8 | 0.98 |
| NM_016099 | GOLGA7 | 0.98 |
| NM_017520 | HSMPP8 | 0.98 |
| NM_020401 | NUP107 | 0.98 |
| NM_144582 | TEX261 | 0.98 |
| NM_138285 | LOC129401 | 0.98 |
| NM_194304 | LOC283031 | 0.98 |
| NM_207505 | FLJ45248 | 0.98 |
| XM_062300 | LOC120824 | 0.98 |
| XM_376576 | LOC401308 | 0.98 |
| NM_032165 | LRRIQ1 | 0.98 |
| NM_005318 | H1F0 | 0.99 |
| NM_005953 | MT2A | 0.99 |
| NM_006147 | IRF6 | 0.99 |
| NM_006095 | ATP8A1 | 0.99 |
| NM_000796 | DRD3 | 0.99 |
| XM_374879 | LOC114971 | 0.99 |
| NM_000874 | IFNAR2 | 0.99 |
| NM_003820 | TNFRSF14 | 0.99 |
| NM_024013 | IFNA1 | 0.99 |
| NM_002889 | RARRES2 | 0.99 |
| NM_001284 | AP3S1 | 0.99 |
| NM_006061 | CRISP3 | 0.99 |
| NM_014989 | RIMS1 | 0.99 |
| NM_012390 | PROL5 | 0.99 |
| NM_018384 | IAN4L1 | 0.99 |
| XM_370660 | KIAA1201 | 0.99 |
| NM_022765 | NICAL | 0.99 |

| | | |
|---|---|---|
| NM_024525 | TTC13 | 0.97 |
| NM_138428 | LOC113444 | 0.97 |
| NM_183376 | ARRDC4 | 0.97 |
| NM_145280 | LOC151194 | 0.97 |
| NM_182578 | FLJ37964 | 0.97 |
| XM_209719 | LOC285679 | 0.97 |
| XM_210543 | LOC285253 | 0.97 |
| XM_373039 | LOC391714 | 0.97 |
| NM_001004476 | OR10K2 | 0.97 |
| NM_005508 | CCR4 | 0.98 |
| NM_003960 | NAT8 | 0.98 |
| NM_006624 | ZMYND11 | 0.98 |
| NM_006914 | RORB | 0.98 |
| NM_005559 | LAMA1 | 0.98 |
| NM_018004 | FLJ10134 | 0.98 |
| NM_002003 | FCN1 | 0.98 |
| NM_004745 | DLGAP2 | 0.98 |
| NM_006446 | SLCO1B1 | 0.98 |
| NM_022163 | MRPL46 | 0.98 |
| NM_017447 | C21ORF91 | 0.98 |
| NM_017843 | BCAS4 | 0.98 |
| NM_024906 | SCD4 | 0.98 |
| NM_145266 | LOC134492 | 0.98 |
| XM_015334 | FAM10A3 | 0.98 |
| NM_178553 | MGC44505 | 0.98 |
| NM_207446 | LOC400451 | 0.98 |
| XM_061864 | LOC120105 | 0.98 |
| NM_001005239 | OR11H1 | 0.98 |
| NM_015076 | CDK11 | 0.99 |
| XM_084482 | ARID5B | 0.99 |
| NM_003409 | ZFP161 | 0.99 |
| NM_002524 | NRAS | 0.99 |
| NM_153615 | RGR | 0.99 |
| NM_017683 | C20ORF23 | 0.99 |
| NM_001243 | TNFRSF8 | 0.99 |
| NM_000176 | NR3C1 | 0.99 |
| NM_002315 | LMO1 | 0.99 |
| NM_005399 | PRKAB2 | 0.99 |
| NM_005080 | XBP1 | 0.99 |
| NM_000847 | GSTA3 | 0.99 |
| NM_006571 | DCTN6 | 0.99 |
| NM_014165 | C6ORF66 | 0.99 |
| NM_017694 | FLJ20160 | 0.99 |
| NM_020674 | CYP20A1 | 0.99 |
| NM_021821 | MRPS35 | 0.99 |
| NM_024077 | SECISBP2 | 0.99 |

| | | | | | |
|---|---|---|---|---|---|
| NM_152310 | ELOVL3 | 0.99 | NM_080474 | SERPINB12 | 0.99 |
| NM_152271 | FLJ23749 | 0.99 | XM_376436 | LOC134466 | 0.99 |
| NM_144615 | MGC23244 | 0.99 | NM_181842 | ZBTB12 | 0.99 |
| NM_181512 | MRPL21 | 0.99 | XM_291335 | LOC340542 | 0.99 |
| XM_379060 | LOC339685 | 0.99 | NM_178011 | LRRTM3 | 0.99 |
| NM_198581 | ZC3HDC6 | 0.99 | XM_374150 | LOC389345 | 0.99 |
| XM_373700 | LOC388305 | 0.99 | XM_371170 | LOC388545 | 0.99 |
| XM_373775 | LOC388467 | 0.99 | XM_373669 | LOC388220 | 0.99 |
| NM_002637 | PHKA1 | 1 | NM_002029 | FPR1 | 1 |
| NM_032787 | GPR128 | 1 | NM_130760 | MADCAM1 | 1 |
| NM_001564 | ING1L | 1 | NM_000450 | SELE | 1 |
| NM_000071 | CBS | 1 | NM_014881 | DCLRE1A | 1 |
| NM_014899 | RHOBTB3 | 1 | NM_004264 | SURB7 | 1 |
| NM_002559 | P2RX3 | 1 | NM_001169 | AQP8 | 1 |
| NM_001270 | CHD1 | 1 | NM_000060 | BTD | 1 |
| AJ277481 | ILK-2 | 1 | NM_005614 | RHEB | 1 |
| NM_000606 | C8G | 1 | NM_033423 | GZMH | 1 |
| NM_002949 | MRPL12 | 1 | NM_012128 | CLCA4 | 1 |
| NM_016310 | POLR3K | 1 | NM_018293 | FLJ10997 | 1 |
| NM_018919 | PCDHGA6 | 1 | NM_020130 | C8ORF4 | 1 |
| XM_031104 | GALNTL1 | 1 | NM_021820 | C6ORF75 | 1 |
| NM_024043 | MGC3101 | 1 | NM_032808 | LRRN6A | 1 |
| NM_052950 | WDFY2 | 1 | XM_058997 | LOC126167 | 1 |
| NM_144663 | 37196 | 1 | NM_014406 | CESK1 | 1 |
| NM_152494 | DCST1 | 1 | NM_182755 | LOC220929 | 1 |
| NM_178527 | MGC43026 | 1 | NM_182579 | FLJ40343 | 1 |
| NM_199183 | TESSP5 | 1 | XM_370726 | LOC387927 | 1 |
| XM_209616 | LOC285453 | 1 | XM_292717 | LOC342850 | 1 |
| XM_371208 | LOC388581 | 1 | XM_373844 | LOC388635 | 1 |
| XM_370686 | LOC387849 | 1 | XM_373926 | LOC388814 | 1 |
| NM_002578 | PAK3 | 1.01 | NM_005377 | MYCL2 | 1.01 |
| NM_005653 | TFCP2 | 1.01 | NM_000113 | DYT1 | 1.01 |
| NM_030955 | ADAMTS12 | 1.01 | NM_005800 | C13ORF22 | 1.01 |
| NM_003122 | SPINK1 | 1.01 | NM_005070 | SLC4A3 | 1.01 |
| NM_003311 | PHLDA2 | 1.01 | NM_002444 | MSN | 1.01 |
| NM_012140 | SLC25A10 | 1.01 | NM_002255 | KIR2DL4 | 1.01 |
| NM_006952 | UPK1B | 1.01 | NM_182490 | ZNF227 | 1.01 |
| NM_004896 | VPS26 | 1.01 | NM_004272 | HOMER1 | 1.01 |
| NM_015435 | RNF19 | 1.01 | NM_015567 | SLITRK5 | 1.01 |
| NM_024335 | IRX6 | 1.01 | NM_024674 | LIN28 | 1.01 |
| NM_031958 | KRTAP3-1 | 1.01 | XM_030300 | UNC5A | 1.01 |
| NM_130783 | LOC90139 | 1.01 | XM_376280 | LOC152078 | 1.01 |
| NM_152519 | FLJ23861 | 1.01 | NM_207496 | C6ORF214 | 1.01 |
| XM_292023 | LOC341356 | 1.01 | XM_373461 | LOC387683 | 1.01 |
| XM_373521 | LOC387826 | 1.01 | XM_380025 | LOC402681 | 1.01 |
| XM_496631 | FLJ35107 | 1.02 | NM_002749 | MAPK7 | 1.02 |

| | | |
|---|---|---|
| NM_003629 | PIK3R3 | 1.02 |
| NM_003805 | CRADD | 1.02 |
| NM_006756 | TCEA1 | 1.02 |
| NM_006415 | SPTLC1 | 1.02 |
| NM_022909 | CENPH | 1.02 |
| NM_014221 | MTCP1 | 1.02 |
| NM_004910 | PITPNM1 | 1.02 |
| NM_006393 | NEBL | 1.02 |
| NM_018987 | SEMA5B | 1.02 |
| NM_018192 | LEPREL1 | 1.02 |
| NM_031488 | L3MBTL2 | 1.02 |
| NM_178838 | LOC90768 | 1.02 |
| NM_014770 | CENTG1 | 1.02 |
| NM_152554 | C6ORF195 | 1.02 |
| NM_133328 | DEDD2 | 1.02 |
| NM_174940 | LOC283232 | 1.02 |
| NM_175857 | KRTAP8-1 | 1.02 |
| NM_207407 | FLJ16046 | 1.02 |
| XM_059689 | LOC134111 | 1.02 |
| XM_376872 | LOC401529 | 1.02 |
| XM_374333 | LOC389878 | 1.02 |
| NM_005009 | NME4 | 1.03 |
| NM_005239 | ETS2 | 1.03 |
| NM_006768 | BRAP | 1.03 |
| NM_000485 | APRT | 1.03 |
| NM_013326 | C18ORF8 | 1.03 |
| NM_000942 | PPIB | 1.03 |
| NM_006190 | ORC2L | 1.03 |
| NM_005808 | CTDSPL | 1.03 |
| NM_014462 | LSM1 | 1.03 |
| NM_020217 | DKFZP547I014 | 1.03 |
| NM_017963 | KIAA1295 | 1.03 |
| NM_032219 | FLJ22269 | 1.03 |
| NM_138343 | KNSL8 | 1.03 |
| NM_198450 | UNQ8193 | 1.03 |
| XM_370690 | ARID2 | 1.03 |
| XM_209408 | LOC284901 | 1.03 |
| XM_379939 | LOC402604 | 1.03 |
| XM_373714 | LOC388338 | 1.03 |
| XM_038150 | MAST3 | 1.04 |
| NM_004328 | BCS1L | 1.04 |
| NM_003083 | SNAPC2 | 1.04 |
| NM_001948 | DUT | 1.04 |
| NM_006681 | NMU | 1.04 |
| NM_012076 | CRB1 | 1.04 |
| AF073924 | OR7E35P | 1.04 |
| NM_004934 | CDH18 | 1.02 |
| NM_021622 | PLEKHA1 | 1.02 |
| NM_024407 | NDUFS7 | 1.02 |
| NM_017677 | MTMR8 | 1.02 |
| NM_024608 | NEIL1 | 1.02 |
| NM_000608 | ORM2 | 1.02 |
| NM_004695 | SLC16A5 | 1.02 |
| NM_013284 | POLM | 1.02 |
| NM_019009 | TOLLIP | 1.02 |
| NM_021639 | SP192 | 1.02 |
| NM_032229 | SLITRK6 | 1.02 |
| NM_145648 | SLC15A4 | 1.02 |
| NM_033296 | PGR1 | 1.02 |
| NM_152574 | C9ORF52 | 1.02 |
| XM_377060 | LOC203547 | 1.02 |
| NM_174981 | ANKRD21 | 1.02 |
| NM_173684 | FLJ35721 | 1.02 |
| NM_032583 | ABCC11 | 1.02 |
| XM_294077 | LOC346157 | 1.02 |
| XM_372367 | LOC390079 | 1.02 |
| NM_001004354 | MGC61598 | 1.02 |
| NM_014397 | NEK6 | 1.03 |
| NM_003223 | TFAP4 | 1.03 |
| NM_052939 | FCRH3 | 1.03 |
| NM_173859 | BASE | 1.03 |
| NM_006133 | C11ORF11 | 1.03 |
| NM_000943 | PPIC | 1.03 |
| NM_006223 | PIN4 | 1.03 |
| XM_374779 | ANKRD26 | 1.03 |
| NM_015718 | NOX3 | 1.03 |
| NM_020909 | EPB41L5 | 1.03 |
| NM_018153 | ANTXR1 | 1.03 |
| NM_032117 | GAJ | 1.03 |
| NM_173079 | RUNDC1 | 1.03 |
| XM_379705 | LOC158572 | 1.03 |
| NM_001001664 | LOC339745 | 1.03 |
| XM_374100 | LOC389248 | 1.03 |
| XM_372753 | LOC390980 | 1.03 |
| NM_001823 | CKB | 1.04 |
| NM_001778 | CD48 | 1.04 |
| NM_006494 | ERF | 1.04 |
| NM_006012 | CLPP | 1.04 |
| XM_050846 | IHH | 1.04 |
| NM_002906 | RDX | 1.04 |
| NM_000047 | ARSE | 1.04 |
| NM_000280 | PAX6 | 1.04 |

| | | | | | |
|---|---|---|---|---|---|
| NM_000345 | SNCA | 1.04 | NM_173075 | APBB2 | 1.04 |
| NM_001539 | DNAJA1 | 1.04 | NM_002404 | MFAP4 | 1.04 |
| NM_015423 | AASDHPPT | 1.04 | NM_022735 | ACBD3 | 1.04 |
| NM_006753 | SURF6 | 1.04 | NM_014821 | KIAA0317 | 1.04 |
| NM_007210 | GALNT6 | 1.04 | NM_015607 | DKFZP547E1010 | 1.04 |
| NM_012453 | TBL2 | 1.04 | NM_016321 | RHCG | 1.04 |
| NM_016470 | C20ORF111 | 1.04 | NM_017948 | NOL8 | 1.04 |
| NM_017920 | URG4 | 1.04 | NM_020647 | JPH1 | 1.04 |
| NM_021817 | HAPLN2 | 1.04 | XM_029084 | FLJ21438 | 1.04 |
| NM_024854 | FLJ22028 | 1.04 | NM_025030 | FLJ20972 | 1.04 |
| NM_025073 | FLJ21168 | 1.04 | NM_144576 | FLJ32452 | 1.04 |
| NM_152363 | FLJ39369 | 1.04 | NM_144643 | FLJ30655 | 1.04 |
| NM_198466 | FLJ37183 | 1.04 | NM_152793 | ELLS1 | 1.04 |
| NM_182592 | FLJ39576 | 1.04 | XM_067176 | LOC131055 | 1.04 |
| XM_372042 | LOC389660 | 1.04 | XM_372469 | LOC390342 | 1.04 |
| XM_379720 | LOC401613 | 1.04 | NM_000586 | IL2 | 1.05 |
| NM_170699 | GPBAR1 | 1.05 | NM_006763 | BTG2 | 1.05 |
| NM_033329 | SIGLECL1 | 1.05 | NM_016123 | IRAK4 | 1.05 |
| NM_002303 | LEPR | 1.05 | NM_000629 | IFNAR1 | 1.05 |
| XM_094581 | SEC24A | 1.05 | NM_012403 | ANP32C | 1.05 |
| NM_005564 | LCN2 | 1.05 | NM_147127 | EVC2 | 1.05 |
| NM_001177 | ARL1 | 1.05 | NM_022444 | SLC13A1 | 1.05 |
| NM_005454 | CER1 | 1.05 | NM_012297 | G3BP2 | 1.05 |
| NM_006371 | CRTAP | 1.05 | NM_152943 | ZNF268 | 1.05 |
| NM_013336 | SEC61A1 | 1.05 | NM_016498 | HSPC242 | 1.05 |
| NM_018112 | C9ORF87 | 1.05 | NM_018001 | FLJ10120 | 1.05 |
| NM_018206 | VPS35 | 1.05 | XM_290799 | ARHGAP23 | 1.05 |
| NM_022068 | FAM38B | 1.05 | NM_152456 | MGC34647 | 1.05 |
| NM_152595 | PGBD4 | 1.05 | XM_378734 | LOC284214 | 1.05 |
| XM_379085 | LOC285043 | 1.05 | XM_208545 | LOC283155 | 1.05 |
| XM_377766 | LOC402103 | 1.05 | XM_380141 | LOC402590 | 1.05 |
| XM_372418 | LOC390213 | 1.05 | XM_370844 | LOC388094 | 1.05 |
| NM_001001437 | MGC12815 | 1.05 | NM_003618 | MAP4K3 | 1.06 |
| NM_000685 | AGTR1 | 1.06 | NM_000838 | GRM1 | 1.06 |
| NM_004276 | CABP1 | 1.06 | NM_013375 | ABT1 | 1.06 |
| NM_207305 | FOXD4 | 1.06 | NM_000941 | POR | 1.06 |
| NM_006601 | TEBP | 1.06 | NM_030773 | TUBB1 | 1.06 |
| NM_016561 | BFAR | 1.06 | NM_003592 | CUL1 | 1.06 |
| NM_052938 | FCRH1 | 1.06 | NM_000825 | GNRH1 | 1.06 |
| NM_001766 | CD1D | 1.06 | NM_021975 | RELA | 1.06 |
| NM_022112 | P53AIP1 | 1.06 | NM_004078 | CSRP1 | 1.06 |
| NM_152992 | POMZP3 | 1.06 | NM_001862 | COX5B | 1.06 |
| NM_000723 | CACNB1 | 1.06 | NM_001475 | GAGE5 | 1.06 |
| NM_207288 | AAA1 | 1.06 | NM_005999 | TSNAX | 1.06 |
| NM_004753 | DHRS3 | 1.06 | NM_005491 | CXORF6 | 1.06 |
| NM_005714 | KCNK7 | 1.06 | NM_006610 | MASP2 | 1.06 |

| | | | | | |
|---|---|---|---|---|---|
| NM_006541 | TXNL2 | 1.06 | NM_014160 | MKRN2 | 1.06 |
| NM_012463 | ATP6V0A2 | 1.06 | NM_013358 | PADI1 | 1.06 |
| NM_017971 | MRPL20 | 1.06 | NM_018148 | WINS1 | 1.06 |
| NM_018289 | FLJ10979 | 1.06 | NM_018442 | PC326 | 1.06 |
| NM_020803 | KLHL8 | 1.06 | NM_020901 | KIAA1542 | 1.06 |
| NM_022831 | FLJ12806 | 1.06 | NM_022901 | LRRC19 | 1.06 |
| NM_025184 | FLJ22843 | 1.06 | NM_025049 | C15ORF20 | 1.06 |
| XM_290462 | LRRC27 | 1.06 | NM_032884 | MGC15882 | 1.06 |
| NM_080928 | ASB15 | 1.06 | NM_152474 | C19ORF18 | 1.06 |
| NM_197956 | C9ORF90 | 1.06 | XM_372289 | CYORF15A | 1.06 |
| NM_207378 | SERPINA13 | 1.06 | NM_207009 | LOC404636 | 1.06 |
| XM_087671 | LOC153441 | 1.06 | XM_379234 | LOC401101 | 1.06 |
| XM_374267 | LOC389654 | 1.06 | XM_372573 | LOC390594 | 1.06 |
| XM_370826 | LOC388065 | 1.06 | NM_000144 | FRDA | 1.07 |
| NM_004873 | BAG5 | 1.07 | NM_005402 | RALA | 1.07 |
| NM_007145 | ZNF146 | 1.07 | NM_005590 | MRE11A | 1.07 |
| NM_016000 | TRNT1 | 1.07 | NM_004330 | BNIP2 | 1.07 |
| NM_001199 | BMP1 | 1.07 | NM_000880 | IL7 | 1.07 |
| NM_001142 | AMELX | 1.07 | NM_000670 | ADH4 | 1.07 |
| NM_004454 | ETV5 | 1.07 | NM_014947 | FOXJ3 | 1.07 |
| NM_014465 | SULT1B1 | 1.07 | NM_001263 | CDS1 | 1.07 |
| NM_001389 | DSCAM | 1.07 | NM_012393 | PFAS | 1.07 |
| NM_023928 | AACS | 1.07 | NM_004775 | B4GALT6 | 1.07 |
| NM_007219 | RNF24 | 1.07 | NM_014900 | COBLL1 | 1.07 |
| NM_152268 | DKFZP727A071 | 1.07 | NM_013231 | FLRT2 | 1.07 |
| NM_013235 | RNASE3L | 1.07 | NM_020312 | DKFZP434K046 | 1.07 |
| NM_022451 | C10ORF117 | 1.07 | NM_024955 | FLJ23322 | 1.07 |
| NM_030578 | MGC4093 | 1.07 | NM_025080 | ASRGL1 | 1.07 |
| NM_032350 | MGC11257 | 1.07 | NM_032268 | ZNRF1 | 1.07 |
| NM_033271 | BTBD6 | 1.07 | NM_018538 | ERMAP | 1.07 |
| XM_058961 | TRAPPC5 | 1.07 | NM_138705 | CAGLP | 1.07 |
| NM_145312 | ZNF485 | 1.07 | NM_148676 | DSCR10 | 1.07 |
| NM_182581 | LOC284680 | 1.07 | NM_198828 | LOC375449 | 1.07 |
| NM_199046 | TEPP | 1.07 | NM_198585 | UNQ2492 | 1.07 |
| XM_293225 | LOC343854 | 1.07 | XM_084672 | LOC143941 | 1.07 |
| XM_373845 | LOC388638 | 1.07 | XM_374110 | LOC389268 | 1.07 |
| XM_374146 | LOC389339 | 1.07 | XM_378080 | LOC402377 | 1.07 |
| XM_376800 | LOC401483 | 1.07 | XM_373344 | LOC392459 | 1.07 |
| NM_006772 | SYNGAP1 | 1.07 | NM_002953 | RPS6KA1 | 1.08 |
| NM_020371 | AVEN | 1.08 | NM_020806 | GPHN | 1.08 |
| NM_003071 | SMARCA3 | 1.08 | NM_004520 | KIF2 | 1.08 |
| NM_018984 | SSH1 | 1.08 | NM_004370 | COL12A1 | 1.08 |
| NM_145058 | MGC7036 | 1.08 | NM_198141 | GANC | 1.08 |
| NM_003918 | GYG2 | 1.08 | NM_003774 | GALNT4 | 1.08 |
| NM_014846 | KIAA0196 | 1.08 | NM_148674 | SMC1L2 | 1.08 |
| NM_018724 | IL20 | 1.08 | NM_022338 | C11ORF24 | 1.08 |

| | | |
|---|---|---|
| NM_017810 | ZNF434 | 1.08 |
| NM_024616 | FLJ23186 | 1.08 |
| NM_024928 | OBFC1 | 1.08 |
| NM_052935 | MGC20781 | 1.08 |
| NM_147202 | C9ORF25 | 1.08 |
| XM_378516 | LOC255177 | 1.08 |
| NM_173629 | C18ORF26 | 1.08 |
| XM_061930 | LOC120237 | 1.08 |
| XM_373277 | LOC392288 | 1.08 |
| NM_153024 | FLJ31393 | 1.09 |
| NM_005533 | IFI35 | 1.09 |
| NM_000367 | TPMT | 1.09 |
| NM_001551 | IGBP1 | 1.09 |
| NM_138694 | PKHD1 | 1.09 |
| AL117555 | DKFZP564I1171 | 1.09 |
| NM_012396 | PHLDA3 | 1.09 |
| NM_006947 | SRP72 | 1.09 |
| NM_004479 | FUT7 | 1.09 |
| NM_006520 | TCTE1L | 1.09 |
| NM_005816 | CD96 | 1.09 |
| NM_007244 | PRR4 | 1.09 |
| NM_022064 | RNF123 | 1.09 |
| NM_030790 | CDA08 | 1.09 |
| NM_030799 | SMAP-5 | 1.09 |
| NM_147189 | MGC39325 | 1.09 |
| NM_145806 | ZNF511 | 1.09 |
| NM_152416 | MGC40214 | 1.09 |
| NM_152606 | ZNF540 | 1.09 |
| NM_173834 | MGC21416 | 1.09 |
| NM_207440 | FLJ26443 | 1.09 |
| XM_059438 | LOC130502 | 1.09 |
| XM_374694 | LOC393062 | 1.09 |
| XM_372428 | LOC390243 | 1.09 |
| XM_372730 | LOC390937 | 1.09 |
| NM_001005216 | OR2J3 | 1.09 |
| NM_032030 | FKSG83 | 1.1 |
| NM_000474 | TWIST1 | 1.1 |
| NM_004832 | GSTO1 | 1.1 |
| NM_000267 | NF1 | 1.1 |
| NM_004128 | GTF2F2 | 1.1 |
| NM_021950 | MS4A1 | 1.1 |
| NM_001250 | TNFRSF5 | 1.1 |
| NM_007029 | STMN2 | 1.1 |
| NM_014371 | AKAP8L | 1.1 |
| NM_015383 | DJ328E19.C1.1 | 1.1 |
| NM_015949 | C7ORF20 | 1.1 |
| XM_040527 | TNN | 1.08 |
| NM_024768 | FLJ12057 | 1.08 |
| NM_032267 | RGSL2 | 1.08 |
| NM_080622 | C20ORF135 | 1.08 |
| NM_173804 | MGC30208 | 1.08 |
| XM_166090 | PLAC9 | 1.08 |
| XM_064903 | LOC126017 | 1.08 |
| XM_375845 | LOC400807 | 1.08 |
| NM_031464 | RPS6KL1 | 1.09 |
| NM_054031 | MRGX3 | 1.09 |
| NM_015001 | SHARP | 1.09 |
| NM_003341 | UBE2E1 | 1.09 |
| NM_001305 | CLDN4 | 1.09 |
| XM_053074 | TIMM50 | 1.09 |
| NM_007267 | EVER1 | 1.09 |
| NM_177453 | PAQR3 | 1.09 |
| NM_021141 | XRCC5 | 1.09 |
| NM_007245 | A2LP | 1.09 |
| NM_004703 | RABEP1 | 1.09 |
| NM_007009 | ZPBP | 1.09 |
| NM_014396 | VPS41 | 1.09 |
| NM_022151 | MOAP1 | 1.09 |
| NM_025114 | FLJ13615 | 1.09 |
| NM_032812 | PLXDC2 | 1.09 |
| NM_133375 | MGC4562 | 1.09 |
| NM_182494 | FAM26A | 1.09 |
| NM_152457 | ZNF597 | 1.09 |
| XM_373847 | LOC339468 | 1.09 |
| XM_291623 | LOC343521 | 1.09 |
| XM_208234 | LOC284672 | 1.09 |
| XM_166805 | LOC219417 | 1.09 |
| XM_379820 | LOC402490 | 1.09 |
| XM_375152 | LOC400304 | 1.09 |
| XM_371106 | LOC388458 | 1.09 |
| NM_001001912 | OR4E2 | 1.09 |
| NM_005517 | HMGN2 | 1.1 |
| NM_080385 | CPA5 | 1.1 |
| NM_004236 | TRIP15 | 1.1 |
| NM_012204 | GTF3C4 | 1.1 |
| NM_015255 | C6ORF133 | 1.1 |
| NM_002986 | CCL11 | 1.1 |
| NM_004060 | CCNG1 | 1.1 |
| NM_001483 | GBAS | 1.1 |
| NM_015472 | TAZ | 1.1 |
| NM_015577 | RAI14 | 1.1 |
| NM_017566 | DKFZP434G0522 | 1.1 |

| | | |
|---|---|---|
| NM_017914 | C19ORF24 | 1.1 |
| XM_166372 | LRFN2 | 1.1 |
| NM_032623 | OSAP | 1.1 |
| NM_058173 | LOC118430 | 1.1 |
| NM_173501 | LOC146174 | 1.1 |
| NM_205849 | FAM9B | 1.1 |
| NM_173538 | FLJ35802 | 1.1 |
| XM_379044 | LOC284898 | 1.1 |
| NM_020880 | ZNF530 | 1.1 |
| XM_378798 | LOC400690 | 1.1 |
| NM_002650 | PIK4CA | 1.11 |
| NM_003506 | FZD6 | 1.11 |
| NM_001572 | IRF7 | 1.11 |
| NM_006521 | TFE3 | 1.11 |
| NM_015328 | KIAA0828 | 1.11 |
| NM_020345 | NKIRAS1 | 1.11 |
| NM_002185 | IL7R | 1.11 |
| NM_000747 | CHRNB1 | 1.11 |
| NM_004518 | KCNQ2 | 1.11 |
| NM_003542 | HIST1H4C | 1.11 |
| NM_022070 | ABC1 | 1.11 |
| NM_005814 | GPA33 | 1.11 |
| NM_007253 | CYP4F8 | 1.11 |
| XM_050561 | SIN3B | 1.11 |
| NM_016062 | CGI-128 | 1.11 |
| NM_018103 | LRRC5 | 1.11 |
| XM_371832 | KIAA1411 | 1.11 |
| NM_022734 | FLJ20859 | 1.11 |
| NM_030769 | NPL | 1.11 |
| NM_138801 | GALM | 1.11 |
| NM_182699 | DDX53 | 1.11 |
| NM_194439 | LOC285498 | 1.11 |
| XM_371304 | LOC388687 | 1.11 |
| XM_373078 | LOC391767 | 1.11 |
| NM_001001700 | FLJ45966 | 1.11 |
| NM_001570 | IRAK2 | 1.12 |
| NM_004850 | ROCK2 | 1.12 |
| NM_014371 | AKAP8L | 1.12 |
| NM_003423 | ZNF43 | 1.12 |
| NM_005134 | PPP4R1 | 1.12 |
| NM_007183 | PKP3 | 1.12 |
| NM_032383 | HPS3 | 1.12 |
| NM_006138 | MS4A3 | 1.12 |
| NM_015263 | RC3 | 1.12 |
| XM_046264 | DKFZP434B172 | 1.12 |
| NM_019005 | FLJ20323 | 1.12 |
| NM_016205 | PDGFC | 1.1 |
| NM_032799 | ZDHHC12 | 1.1 |
| NM_033253 | NT5C1B | 1.1 |
| NM_152385 | FLJ31438 | 1.1 |
| NM_012364 | OR1Q1 | 1.1 |
| NM_144972 | MGC23940 | 1.1 |
| NM_145297 | ZNF626 | 1.1 |
| XM_291020 | LOC339809 | 1.1 |
| NM_207488 | FLJ42393 | 1.1 |
| NM_030911 | CDADC1 | 1.11 |
| NM_001702 | BAI1 | 1.11 |
| NM_001508 | GPR39 | 1.11 |
| NM_004608 | TBX6 | 1.11 |
| NM_016286 | DCXR | 1.11 |
| NM_014554 | SENP1 | 1.11 |
| NM_004788 | UBE4A | 1.11 |
| NM_052918 | SORCS1 | 1.11 |
| NM_002292 | LAMB2 | 1.11 |
| NM_005698 | SCAMP3 | 1.11 |
| NM_005320 | HIST1H1D | 1.11 |
| XM_092995 | ZNF21 | 1.11 |
| NM_007049 | BTN2A1 | 1.11 |
| XM_085028 | ATP11A | 1.11 |
| NM_015430 | DKFZP586H2123 | 1.11 |
| NM_016366 | CABP2 | 1.11 |
| NM_017828 | COMMD4 | 1.11 |
| NM_021179 | LOC57821 | 1.11 |
| NM_022843 | PCDH20 | 1.11 |
| XM_291262 | ZNF251 | 1.11 |
| XM_087137 | LOC151242 | 1.11 |
| NM_152912 | MTIF3 | 1.11 |
| XM_292674 | LOC342669 | 1.11 |
| XM_376278 | LOC401095 | 1.11 |
| XM_378981 | LOC400846 | 1.11 |
| XM_373950 | LOC388887 | 1.11 |
| NM_002742 | PRKCM | 1.12 |
| NM_173858 | VN1R5 | 1.12 |
| NM_005598 | NHLH1 | 1.12 |
| NM_001132 | AFG3L1 | 1.12 |
| NM_002990 | CCL22 | 1.12 |
| XM_088463 | AEGP | 1.12 |
| NR_000039 | RAB9P1 | 1.12 |
| NM_006390 | IPO8 | 1.12 |
| NM_015516 | E2IG4 | 1.12 |
| NM_018976 | SLC38A2 | 1.12 |
| NM_023948 | MOSPD3 | 1.12 |

| | | | | | |
|---|---|---|---|---|---|
| XM_051200 | FTO | 1.12 | NM_024532 | PF20 | 1.12 |
| NM_024761 | MOBKL2B | 1.12 | NM_030920 | ANP32E | 1.12 |
| NM_032129 | DKFZP434H2010 | 1.12 | XM_059987 | ANKRD19 | 1.12 |
| NM_033267 | IRX2 | 1.12 | NM_182541 | TMEM31 | 1.12 |
| NM_173581 | FLJ90231 | 1.12 | NM_207413 | UNQ9433 | 1.12 |
| XM_086308 | LOC148766 | 1.12 | XM_374191 | LOC389443 | 1.12 |
| XM_378028 | LOC402336 | 1.12 | XM_379997 | LOC402643 | 1.12 |
| XM_373915 | LOC388798 | 1.12 | NM_000222 | KIT | 1.13 |
| NM_002556 | OSBP | 1.13 | NM_001674 | ATF3 | 1.13 |
| NM_014212 | HOXC11 | 1.13 | NM_014588 | VSX1 | 1.13 |
| NM_006411 | AGPAT1 | 1.13 | NM_016434 | RTEL1 | 1.13 |
| NM_016577 | RAB6B | 1.13 | NM_004411 | DNCI1 | 1.13 |
| NM_005154 | USP8 | 1.13 | NM_005060 | RORC | 1.13 |
| NM_000088 | COL1A1 | 1.13 | NM_003246 | THBS1 | 1.13 |
| NM_020363 | DAZ2 | 1.13 | NM_006016 | CD164 | 1.13 |
| NM_002006 | FGF2 | 1.13 | NM_014556 | EVC | 1.13 |
| NM_138731 | MIPOL1 | 1.13 | NM_002242 | KCNJ13 | 1.13 |
| NM_014563 | SEDL | 1.13 | NM_014628 | MAD2L1BP | 1.13 |
| NM_005887 | DLEU1 | 1.13 | NM_199069 | DKFZP564J0123 | 1.13 |
| NM_024510 | MGC4368 | 1.13 | NM_024840 | ZNF613 | 1.13 |
| NM_033034 | TRIM5 | 1.13 | NM_152351 | SLC5A10 | 1.13 |
| XM_378312 | LOC283143 | 1.13 | NM_203305 | MGC50853 | 1.13 |
| XM_210035 | LOC286408 | 1.13 | XM_379118 | LOC400992 | 1.13 |
| XM_374061 | LOC389167 | 1.13 | XM_370536 | LOC387642 | 1.13 |
| XM_370664 | LOC387816 | 1.13 | NM_004635 | MAPKAPK3 | 1.14 |
| NM_006218 | PIK3CA | 1.14 | NM_001401 | EDG2 | 1.14 |
| NM_012375 | OR52A1 | 1.14 | NM_013381 | TRHDE | 1.14 |
| NM_000369 | TSHR | 1.14 | NM_022041 | GAN | 1.14 |
| NM_014516 | CNOT3 | 1.14 | NM_007107 | SSR3 | 1.14 |
| NM_002502 | NFKB2 | 1.14 | NM_000026 | ADSL | 1.14 |
| NM_002225 | IVD | 1.14 | NM_000496 | CRYBB2 | 1.14 |
| NM_006136 | CAPZA2 | 1.14 | NM_005755 | EBI3 | 1.14 |
| NM_001094 | ACCN1 | 1.14 | NM_004529 | MLLT3 | 1.14 |
| NM_002679 | PMS2L2 | 1.14 | NM_005396 | PNLIPRP2 | 1.14 |
| NM_021143 | ZNF20 | 1.14 | XM_085507 | ZNF500 | 1.14 |
| NM_017918 | FLJ20647 | 1.14 | NM_018370 | FLJ11259 | 1.14 |
| NM_018242 | FLJ10847 | 1.14 | NM_033326 | SOX6 | 1.14 |
| NM_024548 | FLJ23047 | 1.14 | NM_025057 | C14ORF45 | 1.14 |
| NM_031941 | USHBP1 | 1.14 | NM_147174 | HS6ST2 | 1.14 |
| NM_178829 | C7ORF34 | 1.14 | NM_152994 | LOC129285 | 1.14 |
| NM_144666 | FLJ32752 | 1.14 | NM_153714 | C10ORF67 | 1.14 |
| NM_175858 | KRTAP11-1 | 1.14 | NM_207484 | FLJ40712 | 1.14 |
| NM_003519 | HIST1H2BL | 1.14 | XM_292889 | LOC344022 | 1.14 |
| XM_374060 | LOC389166 | 1.14 | XM_374168 | LOC389392 | 1.14 |
| XM_372614 | LOC390683 | 1.14 | XM_371014 | LOC388333 | 1.14 |
| NM_001005466 | OR10G2 | 1.14 | NM_031303 | KATNAL2 | 1.14 |

| | | | | | |
|---|---|---|---|---|---|
| NM_005160 | ADRBK2 | 1.15 | NM_000167 | GK | 1.15 |
| NM_015715 | PLA2G3 | 1.15 | NM_006627 | POP4 | 1.15 |
| NM_016147 | PME-1 | 1.15 | NM_001494 | GDI2 | 1.15 |
| NM_002002 | FCER2 | 1.15 | NM_145298 | APOBEC3F | 1.15 |
| NM_002270 | TNPO1 | 1.15 | NM_000491 | C1QB | 1.15 |
| NM_005342 | HMGB3 | 1.15 | NM_005271 | GLUD1 | 1.15 |
| NM_000246 | MHC2TA | 1.15 | NM_006145 | DNAJB1 | 1.15 |
| NM_002728 | PRG2 | 1.15 | NM_006926 | SFTPA2 | 1.15 |
| NM_024551 | ADIPOR2 | 1.15 | NM_003052 | SLC34A1 | 1.15 |
| NM_003928 | CXX1 | 1.15 | NM_014940 | HSRG1 | 1.15 |
| NM_016612 | MSCP | 1.15 | NM_024021 | MS4A4A | 1.15 |
| NM_015888 | HOOK1 | 1.15 | NM_018087 | FLJ10407 | 1.15 |
| NM_020648 | TWSG1 | 1.15 | NM_022728 | NEUROD6 | 1.15 |
| NM_024848 | FLJ13941 | 1.15 | NM_024850 | BTNL8 | 1.15 |
| NM_033503 | BMF | 1.15 | NM_145170 | TTC18 | 1.15 |
| NM_181708 | LOC144233 | 1.15 | NM_173539 | ZNF596 | 1.15 |
| NM_152744 | SDK1 | 1.15 | NM_207343 | DKFZP547C195 | 1.15 |
| NM_205850 | SLC24A5 | 1.15 | XM_373109 | LOC340156 | 1.15 |
| XM_372117 | PTAR1 | 1.15 | NM_182635 | LOC349236 | 1.15 |
| XM_371626 | LOC389107 | 1.15 | XM_376049 | LOC400950 | 1.15 |
| XM_377305 | LOC401740 | 1.15 | NM_016083 | CNR1 | 1.16 |
| NM_002432 | MNDA | 1.16 | NM_005997 | TCFL1 | 1.16 |
| NM_002662 | PLD1 | 1.16 | NM_000403 | GALE | 1.16 |
| NM_001304 | CPD | 1.16 | NM_001083 | PDE5A | 1.16 |
| NM_003102 | SOD3 | 1.16 | NM_020241 | SEMA6B | 1.16 |
| NM_000216 | KAL1 | 1.16 | XM_371850 | C6orf103 | 1.16 |
| NM_020381 | C6ORF210 | 1.16 | NM_002879 | RAD52 | 1.16 |
| NM_012286 | MORF4L2 | 1.16 | NM_016512 | SPAG11 | 1.16 |
| NM_006456 | SIAT7B | 1.16 | NM_015246 | MGRN1 | 1.16 |
| NM_022157 | RRAGC | 1.16 | NM_024622 | FLJ21901 | 1.16 |
| NM_030788 | DCSTAMP | 1.16 | NM_030971 | BA108L7.2 | 1.16 |
| NM_032302 | MGC10911 | 1.16 | NM_032558 | FLJ14753 | 1.16 |
| NM_052862 | MK2S4 | 1.16 | NM_198149 | C1ORF40 | 1.16 |
| NM_173514 | FLJ90709 | 1.16 | NM_152604 | ZNF383 | 1.16 |
| NM_183242 | BTBD8 | 1.16 | XM_211908 | LOC285479 | 1.16 |
| XM_062285 | LOC120775 | 1.16 | XM_378349 | LOC400019 | 1.16 |
| XM_372705 | LOC390877 | 1.16 | NM_001004344 | MGC44328 | 1.16 |
| XM_372248 | LOC389888 | 1.16 | XM_379702 | LOC401585 | 1.16 |
| NM_014240 | LIMD1 | 1.17 | NM_005946 | MT1A | 1.17 |
| NM_006410 | HTATIP2 | 1.17 | NM_000821 | GGCX | 1.17 |
| NM_015350 | TA-LRRP | 1.17 | NM_004399 | DDX11 | 1.17 |
| NM_006406 | PRDX4 | 1.17 | NM_020416 | PPP2R2C | 1.17 |
| NM_007026 | DUSP14 | 1.17 | NM_016651 | DACT1 | 1.17 |
| NM_005164 | ABCD2 | 1.17 | NM_016428 | ABI3 | 1.17 |
| NM_021082 | SLC15A2 | 1.17 | NM_003447 | ZNF165 | 1.17 |
| NM_021996 | GBGT1 | 1.17 | NM_014583 | LMCD1 | 1.17 |

| | | | | | |
|---|---|---|---|---|---|
| NM_017766 | FLJ20321 | 1.17 | NM_018316 | FLJ11078 | 1.17 |
| NM_022730 | COPS7B | 1.17 | NM_024952 | C14ORF159 | 1.17 |
| NM_030961 | TRIM56 | 1.17 | NM_031215 | CABLES2 | 1.17 |
| NM_032861 | SERAC1 | 1.17 | NM_033414 | ZNF622 | 1.17 |
| NM_144565 | NIP | 1.17 | NM_033184 | KRTAP2-4 | 1.17 |
| NM_178453 | MGC52282 | 1.17 | NM_033259 | CAM-KIIN | 1.17 |
| NM_001008274 | LOC493829 | 1.17 | XM_209700 | LOC285647 | 1.17 |
| XM_378879 | LOC400763 | 1.17 | XM_375604 | LOC400689 | 1.17 |
| XM_378758 | LOC400660 | 1.17 | NM_001005224 | OR4F3 | 1.17 |
| NM_001005496 | OR5D16 | 1.17 | NM_022049 | GPR88 | 1.18 |
| NM_000038 | APC | 1.18 | NM_005985 | SNAI1 | 1.18 |
| NM_005220 | DLX3 | 1.18 | NM_005240 | ETV3 | 1.18 |
| NM_001754 | RUNX1 | 1.18 | NM_002337 | LRPAP1 | 1.18 |
| NM_021724 | NR1D1 | 1.18 | NM_014427 | CPNE7 | 1.18 |
| NM_017913 | CDC37L1 | 1.18 | NM_016203 | PRKAG2 | 1.18 |
| NM_004528 | MGST3 | 1.18 | NM_001082 | CYP4F2 | 1.18 |
| NM_024419 | PGS1 | 1.18 | NM_007263 | COPE | 1.18 |
| XM_028522 | MYR8 | 1.18 | NM_012287 | CENTB2 | 1.18 |
| NM_015933 | HSPC016 | 1.18 | NM_016322 | RAB14 | 1.18 |
| NM_017728 | FLJ20255 | 1.18 | NM_019104 | F25965 | 1.18 |
| NM_032227 | FLJ22679 | 1.18 | NM_030969 | TMEM14B | 1.18 |
| NM_053043 | MGC20460 | 1.18 | NM_176884 | TAS2R43 | 1.18 |
| NM_173682 | FLJ40288 | 1.18 | NM_014096 | SLC43A3 | 1.18 |
| XM_292210 | LOC338870 | 1.18 | XM_293029 | CDKL4 | 1.18 |
| XM_292678 | LOC339291 | 1.18 | XM_372040 | LOC389652 | 1.18 |
| XM_376003 | LOC400924 | 1.18 | NM_005286 | GPR8 | 1.19 |
| NM_006980 | MTERF | 1.19 | NM_020249 | ADAMTS9 | 1.19 |
| XM_290898 | DUSTYPK | 1.19 | XM_496642 | USP19 | 1.19 |
| NM_004810 | GRAP2 | 1.19 | NM_001294 | CLPTM1 | 1.19 |
| NM_002490 | NDUFA6 | 1.19 | NM_014657 | KIAA0406 | 1.19 |
| NM_014434 | NDOR1 | 1.19 | NM_018340 | FLJ11151 | 1.19 |
| NM_020219 | CEAL1 | 1.19 | NM_024730 | FLJ22655 | 1.19 |
| NM_024770 | FLJ13984 | 1.19 | NM_024863 | FLJ21174 | 1.19 |
| NM_175875 | SIX5 | 1.19 | NM_144714 | FLJ25449 | 1.19 |
| NM_144967 | FLJ30058 | 1.19 | NM_173798 | LOC170261 | 1.19 |
| NM_145665 | SPANXE | 1.19 | NM_139285 | GAR17 | 1.19 |
| NM_173572 | C10ORF93 | 1.19 | NM_207489 | FLJ35816 | 1.19 |
| NM_004312 | ARR3 | 1.19 | XM_373826 | LOC388593 | 1.19 |
| XM_372761 | LOC391001 | 1.19 | XM_380117 | LOC402532 | 1.19 |
| XM_370715 | LOC387911 | 1.19 | XM_370949 | LOC388229 | 1.19 |
| NM_004579 | MAP4K2 | 1.2 | NM_006055 | LANCL1 | 1.2 |
| NM_001908 | CTSB | 1.2 | NM_012213 | MLYCD | 1.2 |
| NM_016155 | MMP17 | 1.2 | NM_173536 | GABRG1 | 1.2 |
| NM_005069 | SIM2 | 1.2 | NM_001038 | SCNN1A | 1.2 |
| NM_005566 | LDHA | 1.2 | NM_001678 | ATP1B2 | 1.2 |
| NM_005273 | GNB2 | 1.2 | NM_022568 | ALDH8A1 | 1.2 |

| | | | | | |
|---|---|---|---|---|---|
| NM_002971 | SATB1 | 1.2 | NM_024923 | NUP210 | 1.2 |
| NM_020850 | RANBP10 | 1.2 | NM_024621 | FLJ12604 | 1.2 |
| NM_032714 | C14orf151 | 1.2 | NM_138379 | LOC91937 | 1.2 |
| NM_198441 | FLJ40296 | 1.2 | NM_153006 | NAGS | 1.2 |
| NM_178230 | COAS2 | 1.2 | NM_153756 | FNDC5 | 1.2 |
| NM_173808 | NEGR1 | 1.2 | NM_173812 | FLJ32949 | 1.2 |
| NM_182563 | MGC21830 | 1.2 | NM_172311 | SALF | 1.2 |
| XM_371670 | LOC389158 | 1.2 | XM_374372 | LOC392238 | 1.2 |
| XM_374286 | LOC389695 | 1.2 | XM_370648 | LOC387804 | 1.2 |
| XM_377000 | LOC401589 | 1.2 | NM_005443 | PAPSS1 | 1.21 |
| NM_001505 | GPR30 | 1.21 | NM_016945 | TAS2R16 | 1.21 |
| NM_031891 | CDH20 | 1.21 | NM_138960 | TGIF2LX | 1.21 |
| NM_015369 | TP53TG3 | 1.21 | NM_133368 | KIAA1972 | 1.21 |
| NM_014671 | UBE3C | 1.21 | NM_004449 | ERG | 1.21 |
| NM_022725 | FANCF | 1.21 | NM_005578 | LPP | 1.21 |
| NM_003596 | TPST1 | 1.21 | NM_016341 | PLCE1 | 1.21 |
| NM_017846 | SECP43 | 1.21 | NM_018670 | MESP1 | 1.21 |
| NM_032111 | MRPL14 | 1.21 | NM_024310 | PLEKHF1 | 1.21 |
| NM_031964 | KRTAP17-1 | 1.21 | NM_032373 | RNF159 | 1.21 |
| XM_028810 | KIAA1755 | 1.21 | NM_175871 | FLJ35119 | 1.21 |
| NM_138286 | LOC148213 | 1.21 | XM_378823 | LOC148413 | 1.21 |
| NM_152742 | GPC2 | 1.21 | NM_213601 | LOC283578 | 1.21 |
| XM_370838 | LOC339005 | 1.21 | XM_293580 | GPR149 | 1.21 |
| XM_292027 | LOC341370 | 1.21 | NM_003681 | PDXK | 1.22 |
| NM_001048 | SST | 1.22 | NM_002858 | ABCD3 | 1.22 |
| NM_005524 | HES1 | 1.22 | NM_007265 | HSGT1 | 1.22 |
| NM_012086 | GTF3C3 | 1.22 | NM_001361 | DHODH | 1.22 |
| NM_002575 | SERPINB2 | 1.22 | NM_019074 | DLL4 | 1.22 |
| NM_014141 | CNTNAP2 | 1.22 | NM_016174 | CEECAM1 | 1.22 |
| NM_032701 | MGC2705 | 1.22 | NM_001836 | CMA1 | 1.22 |
| NM_001864 | COX7A1 | 1.22 | NM_007216 | HPS5 | 1.22 |
| NM_015693 | PDZK6 | 1.22 | XM_168590 | ZRF1 | 1.22 |
| NM_014162 | HSPC072 | 1.22 | NM_153240 | NPHP3 | 1.22 |
| NM_013291 | CPSF1 | 1.22 | NM_016516 | VPS54 | 1.22 |
| NM_018372 | RIF1 | 1.22 | NM_020417 | TBX20 | 1.22 |
| NM_016050 | MRPL11 | 1.22 | NM_022760 | C20ORF81 | 1.22 |
| NM_022474 | MPP5 | 1.22 | NM_023939 | MGC2752 | 1.22 |
| NM_030634 | ZNF436 | 1.22 | NM_025065 | RPF1 | 1.22 |
| NM_133448 | KIAA1944 | 1.22 | NM_138795 | ARL10B | 1.22 |
| NM_080388 | S100A16 | 1.22 | NM_152458 | FLJ32130 | 1.22 |
| NM_152676 | FBXO15 | 1.22 | NM_198499 | FLJ46156 | 1.22 |
| NM_016206 | FLJ38507 | 1.22 | NM_207477 | FLJ27365 | 1.22 |
| XM_087500 | LOC152667 | 1.22 | XM_372566 | LOC390570 | 1.22 |
| NM_006648 | PRKWNK2 | 1.23 | NM_020680 | SCYL1 | 1.23 |
| NM_178471 | GPR119 | 1.23 | NM_032119 | MASS1 | 1.23 |
| NM_004155 | SERPINB9 | 1.23 | NM_007108 | TCEB2 | 1.23 |

| | | | | | |
|---|---|---|---|---|---|
| NM_022476 | FTS | 1.23 | NM_005525 | HSD11B1 | 1.23 |
| NM_004675 | ARHI | 1.23 | NM_014330 | PPP1R15A | 1.23 |
| NM_006157 | NELL1 | 1.23 | NM_003601 | SMARCA5 | 1.23 |
| NM_130466 | UBE3B | 1.23 | NM_000626 | CD79B | 1.23 |
| XM_044727 | MTMR7 | 1.23 | NM_006669 | LILRB1 | 1.23 |
| NM_017541 | CRYGS | 1.23 | NM_016131 | RAB10 | 1.23 |
| NM_000935 | PLOD2 | 1.23 | NM_003045 | SLC7A1 | 1.23 |
| NM_014678 | KIAA0685 | 1.23 | NM_007246 | KLHL2 | 1.23 |
| NM_015064 | ELKS | 1.23 | NM_018051 | FLJ10300 | 1.23 |
| NM_017610 | RNF111 | 1.23 | NM_018131 | C10ORF3 | 1.23 |
| NM_018464 | C10ORF70 | 1.23 | NM_022454 | SOX17 | 1.23 |
| NM_024645 | FLJ13842 | 1.23 | NM_052886 | MAL2 | 1.23 |
| NM_178830 | FLJ36888 | 1.23 | NM_144654 | C9ORF116 | 1.23 |
| NM_002936 | RNASEH1 | 1.23 | XM_378946 | LOC400812 | 1.23 |
| XM_371681 | LOC389178 | 1.23 | XM_373548 | LOC387886 | 1.23 |
| XM_373496 | LOC387762 | 1.23 | XM_378562 | LOC400496 | 1.23 |
| XM_352913 | RAB26 | 1.24 | NM_018930 | PCDHB10 | 1.24 |
| NM_005593 | MYF5 | 1.24 | NM_002955 | RREB1 | 1.24 |
| NM_080738 | EDARADD | 1.24 | NM_003789 | TRADD | 1.24 |
| NM_003383 | VLDLR | 1.24 | NM_003980 | MAP7 | 1.24 |
| NM_006185 | NUMA1 | 1.24 | NM_058241 | CCNT2 | 1.24 |
| NM_000152 | GAA | 1.24 | NM_006737 | KIR3DL2 | 1.24 |
| NM_005468 | NAALADL1 | 1.24 | NM_006779 | CDC42EP2 | 1.24 |
| XM_290546 | KIAA0830 | 1.24 | XM_376895 | ZNF510 | 1.24 |
| NM_017812 | CHCHD3 | 1.24 | XM_035946 | KIAA1613 | 1.24 |
| NM_020753 | CASKIN2 | 1.24 | NM_022490 | PAF53 | 1.24 |
| NM_024038 | MGC2803 | 1.24 | NM_032567 | NYD-TSP1 | 1.24 |
| NM_053000 | TIGA1 | 1.24 | XM_059377 | FLJ40298 | 1.24 |
| NM_178004 | PRIMA1 | 1.24 | XM_379434 | LOC154092 | 1.24 |
| NM_144698 | FLJ25124 | 1.24 | XM_372144 | PHYHD1 | 1.24 |
| NM_182562 | FLJ39743 | 1.24 | NM_181600 | KRTAP13-4 | 1.24 |
| XM_379386 | LOC285768 | 1.24 | XM_371202 | FLJ00038 | 1.24 |
| XM_060301 | LOC127059 | 1.24 | XM_290949 | LOC339553 | 1.24 |
| XM_211557 | LOC284623 | 1.24 | XM_171078 | LOC255324 | 1.24 |
| XM_375018 | LOC400121 | 1.24 | NM_001001966 | OR5AT1 | 1.24 |
| NM_003188 | MAP3K7 | 1.25 | NM_024779 | PIP5K2C | 1.25 |
| NM_001637 | AOAH | 1.25 | NM_058167 | UBE2J2 | 1.25 |
| NM_018217 | C20ORF31 | 1.25 | NM_000078 | CETP | 1.25 |
| NM_005754 | G3BP | 1.25 | NM_001613 | ACTA2 | 1.25 |
| NM_005047 | PSMD5 | 1.25 | NM_003716 | CADPS | 1.25 |
| NM_015509 | DKFZP566B183 | 1.25 | NM_018109 | PAPD1 | 1.25 |
| XM_035371 | ZFYVE28 | 1.25 | NM_025106 | SSB1 | 1.25 |
| NM_031433 | MFRP | 1.25 | NM_032343 | CHCHD6 | 1.25 |
| NM_032709 | C10ORF33 | 1.25 | NM_183238 | ZNF605 | 1.25 |
| NM_144626 | MGC17299 | 1.25 | NM_152505 | C21ORF13 | 1.25 |
| XM_376013 | LOC200321 | 1.25 | NM_198996 | LOC375108 | 1.25 |

| | | | | | |
|---|---|---|---|---|---|
| XM_210755 | LOC286365 | 1.25 | XM_379667 | LOC401550 | 1.25 |
| XM_372121 | LOC389764 | 1.25 | XM_378279 | LOC399875 | 1.25 |
| XM_372900 | LOC391322 | 1.25 | XM_373968 | LOC388915 | 1.25 |
| NM_173492 | PIP5KL1 | 1.26 | NM_002377 | MAS1 | 1.26 |
| NM_024342 | GRLF1 | 1.26 | NM_000692 | ALDH1B1 | 1.26 |
| NM_006207 | PDGFRL | 1.26 | NM_012215 | MGEA5 | 1.26 |
| NM_022366 | TFB2M | 1.26 | NM_003686 | EXO1 | 1.26 |
| NM_007054 | KIF3A | 1.26 | NM_021969 | NR0B2 | 1.26 |
| NM_004693 | K6HF | 1.26 | NM_001846 | COL4A2 | 1.26 |
| NM_006417 | IFI44 | 1.26 | NM_000701 | ATP1A1 | 1.26 |
| NM_000765 | CYP3A7 | 1.26 | NM_002164 | INDO | 1.26 |
| NM_004173 | SLC7A4 | 1.26 | NM_003166 | SULT1A3 | 1.26 |
| NM_014969 | KIAA0893 | 1.26 | NM_014451 | B1 | 1.26 |
| NM_018940 | PCDHB7 | 1.26 | XM_166451 | KIAA1586 | 1.26 |
| NM_024919 | FRMD1 | 1.26 | NM_152904 | HCMOGT-1 | 1.26 |
| NM_080842 | LOC129026 | 1.26 | NM_173660 | FLJ33718 | 1.26 |
| XM_116980 | LOC197049 | 1.26 | XM_060328 | LOC127099 | 1.26 |
| XM_087762 | LOC153778 | 1.26 | XM_372732 | LOC390940 | 1.26 |
| XM_380019 | LOC402675 | 1.26 | NM_000116 | TAZ | 1.27 |
| NM_002427 | MMP13 | 1.27 | NM_003119 | SPG7 | 1.27 |
| NM_002869 | RAB6A | 1.27 | NM_018557 | LRP1B | 1.27 |
| NM_000342 | SLC4A1 | 1.27 | NM_005002 | NDUFA9 | 1.27 |
| NM_014003 | DHX38 | 1.27 | NM_015200 | KIAA0648 | 1.27 |
| NM_017993 | FLJ10094 | 1.27 | NM_017720 | STAP2 | 1.27 |
| NM_019609 | CPXM | 1.27 | NM_020870 | SH3MD2 | 1.27 |
| NM_024300 | CHCHD7 | 1.27 | NM_153025 | FLJ31606 | 1.27 |
| NM_145206 | VTI1A | 1.27 | XM_172341 | FLJ35036 | 1.27 |
| XM_371250 | LOC388630 | 1.27 | XM_379562 | LOC401456 | 1.27 |
| XM_372527 | LOC390488 | 1.27 | XM_373373 | LOC392549 | 1.27 |
| NM_001005217 | FRG2 | 1.27 | NM_001005278 | OR6N2 | 1.27 |
| NM_014840 | ARK5 | 1.28 | NM_004311 | ARL3 | 1.28 |
| NM_080819 | GPR78 | 1.28 | NM_017790 | RGS3 | 1.28 |
| NM_018699 | PRDM5 | 1.28 | NM_004219 | PTTG1 | 1.28 |
| NM_016292 | TRAP1 | 1.28 | NM_005711 | EDIL3 | 1.28 |
| NM_000748 | CHRNB2 | 1.28 | NM_015927 | TGFB1I1 | 1.28 |
| NM_000313 | PROS1 | 1.28 | NM_021083 | XK | 1.28 |
| XM_352906 | ACYP1 | 1.28 | NM_004368 | CNN2 | 1.28 |
| NM_001474 | GAGE4 | 1.28 | NM_021638 | AFAP | 1.28 |
| NM_004232 | SOCS6 | 1.28 | NM_014876 | KIAA0063 | 1.28 |
| NM_014153 | ZC3HDC7 | 1.28 | NM_016558 | SCAND1 | 1.28 |
| NM_018038 | FLJ10246 | 1.28 | NM_022152 | PP1201 | 1.28 |
| NM_030627 | CPEB4 | 1.28 | NM_032016 | STARD3NL | 1.28 |
| NM_032039 | DKFZP761D0211 | 1.28 | NM_014903 | NAV3 | 1.28 |
| NM_138811 | C7ORF31 | 1.28 | NM_177454 | KIAA1946 | 1.28 |
| NM_152631 | FLJ35782 | 1.28 | XM_296315 | LOC341604 | 1.28 |
| XM_068903 | LOC134541 | 1.28 | XM_169258 | LOC219638 | 1.28 |

| | | | | | |
|---|---|---|---|---|---|
| XM_373849 | LOC388643 | 1.28 | XM_371630 | LOC389112 | 1.28 |
| XM_379248 | LOC401112 | 1.28 | NM_001004319 | VHLL | 1.28 |
| NM_015669 | PCDHB5 | 1.29 | NM_005437 | NCOA4 | 1.29 |
| NM_004952 | EFNA3 | 1.29 | NM_015902 | DD5 | 1.29 |
| NM_012130 | CLDN14 | 1.29 | NM_006790 | TTID | 1.29 |
| NM_004794 | RAB33A | 1.29 | NM_001844 | COL2A1 | 1.29 |
| NM_004102 | FABP3 | 1.29 | NM_005981 | SAS | 1.29 |
| NM_002665 | PLGL | 1.29 | NM_020963 | MOV10 | 1.29 |
| NM_003272 | TM7SF1 | 1.29 | NM_004909 | TRAG3 | 1.29 |
| NM_012472 | TSLRP | 1.29 | NM_016617 | UFM1 | 1.29 |
| NM_018057 | SLC6A15 | 1.29 | NM_018107 | RBM23 | 1.29 |
| NM_017984 | ZCWPW1 | 1.29 | NM_015392 | NPDC1 | 1.29 |
| NM_022350 | LRAP | 1.29 | NM_024771 | FLJ13848 | 1.29 |
| NM_032121 | DKFZP564K142 | 1.29 | NM_033062 | KRTAP4-2 | 1.29 |
| NM_032523 | OSBPL6 | 1.29 | NM_144596 | TTC8 | 1.29 |
| NM_152493 | FLJ25476 | 1.29 | XM_376981 | LOC401581 | 1.29 |
| NM_001522 | GUCY2F | 1.3 | XM_055866 | LMTK3 | 1.3 |
| NM_003696 | OR6A2 | 1.3 | NM_000561 | GSTM1 | 1.3 |
| NM_001076 | UGT2B15 | 1.3 | NM_001733 | C1R | 1.3 |
| NM_003891 | PROZ | 1.3 | NM_004412 | DNMT2 | 1.3 |
| NM_000702 | ATP1A2 | 1.3 | NM_012347 | FBXO9 | 1.3 |
| NM_006504 | PTPRE | 1.3 | NM_004437 | EPB41 | 1.3 |
| NM_000048 | ASL | 1.3 | XM_376567 | KIAA1856 | 1.3 |
| NM_002257 | KLK1 | 1.3 | NM_006041 | HS3ST3B1 | 1.3 |
| NM_006615 | CAPN9 | 1.3 | NM_015070 | KIAA0853 | 1.3 |
| NM_012106 | ARL2BP | 1.3 | NM_014161 | MRPL18 | 1.3 |
| NM_018129 | PNPO | 1.3 | NM_017666 | SUHW3 | 1.3 |
| NM_021047 | ZNF253 | 1.3 | NM_031462 | CD99L2 | 1.3 |
| NM_032312 | MGC11061 | 1.3 | XM_376684 | LOC93432 | 1.3 |
| NM_145202 | PRAP1 | 1.3 | NM_182498 | MGC51082 | 1.3 |
| NM_152477 | ZNF565 | 1.3 | XM_379378 | LOC401220 | 1.3 |
| XM_379359 | LOC401210 | 1.3 | XM_370992 | LOC388298 | 1.3 |
| XM_371078 | LOC388419 | 1.3 | NM_005104 | BRD2 | 1.31 |
| NM_024876 | ADCK4 | 1.31 | NM_145001 | STK32A | 1.31 |
| NM_002841 | PTPRG | 1.31 | NM_001762 | CCT6A | 1.31 |
| NM_007358 | M96 | 1.31 | NM_006184 | NUCB1 | 1.31 |
| NM_001269 | CHC1 | 1.31 | NM_001710 | BF | 1.31 |
| NM_000293 | PHKB | 1.31 | NM_000368 | TSC1 | 1.31 |
| NM_007106 | UBL3 | 1.31 | NM_002836 | PTPRA | 1.31 |
| NM_021073 | BMP5 | 1.31 | NM_000043 | TNFRSF6 | 1.31 |
| NM_001877 | CR2 | 1.31 | NM_002236 | KCNF1 | 1.31 |
| NM_031157 | HNRPA1 | 1.31 | NM_003706 | PLA2G4C | 1.31 |
| XM_291001 | MYO7B | 1.31 | NM_032834 | ALG10 | 1.31 |
| NM_015156 | RCOR1 | 1.31 | NM_013242 | GTL3 | 1.31 |
| XM_098762 | CHD7 | 1.31 | NM_020898 | KIAA1536 | 1.31 |
| NM_024331 | C20ORF121 | 1.31 | NM_024693 | ECHDC3 | 1.31 |

| | | | | | |
|---|---|---|---|---|---|
| NM_024881 | SLC35E1 | 1.31 | NM_031426 | C9ORF58 | 1.31 |
| NM_032930 | MGC13040 | 1.31 | NM_054108 | HRLP5 | 1.31 |
| NM_080608 | C20ORF165 | 1.31 | NM_173497 | HECTD2 | 1.31 |
| NM_182523 | MGC61571 | 1.31 | NM_173530 | ZNF610 | 1.31 |
| NM_145305 | LOC203427 | 1.31 | NM_152761 | FLJ25444 | 1.31 |
| NM_173626 | SLC26A11 | 1.31 | NM_001005272 | OR4A5 | 1.31 |
| NM_133494 | NEK7 | 1.32 | NM_002566 | P2RY11 | 1.32 |
| NM_000911 | OPRD1 | 1.32 | NM_015568 | PPP1R16B | 1.32 |
| NM_014233 | UBTF | 1.32 | NM_001814 | CTSC | 1.32 |
| NM_022355 | DPEP2 | 1.32 | NM_004911 | ERP70 | 1.32 |
| NM_001071 | TYMS | 1.32 | NM_012228 | MSRB | 1.32 |
| NM_016195 | MPHOSPH1 | 1.32 | NM_033131 | WNT3A | 1.32 |
| NM_002010 | FGF9 | 1.32 | NM_004195 | TNFRSF18 | 1.32 |
| NM_004519 | KCNQ3 | 1.32 | NM_018158 | SLC4A1AP | 1.32 |
| NM_052830 | GGTL3 | 1.32 | NM_013361 | ZNF223 | 1.32 |
| NM_003225 | TFF1 | 1.32 | NM_007177 | TU3A | 1.32 |
| NM_015221 | DNMBP | 1.32 | NM_016303 | WBP5 | 1.32 |
| NM_019064 | SDK2 | 1.32 | NM_017558 | HYDIN | 1.32 |
| NM_018686 | CMAS | 1.32 | NM_024090 | ELOVL6 | 1.32 |
| NM_138364 | LOC90826 | 1.32 | NM_178134 | CYP4Z1 | 1.32 |
| XM_294139 | C6ORF143 | 1.32 | XM_376179 | LOC401034 | 1.32 |
| XM_371374 | LOC388774 | 1.32 | XM_371034 | LOC388361 | 1.32 |
| NM_001005287 | OR2A1 | 1.32 | NM_144624 | KIS | 1.33 |
| NM_020240 | SPEC2 | 1.33 | NM_001057 | TACR2 | 1.33 |
| NM_004892 | SEC22L1 | 1.33 | NM_005252 | FOS | 1.33 |
| NM_003859 | DPM1 | 1.33 | NM_016015 | LCMT1 | 1.33 |
| NM_003026 | SH3GL2 | 1.33 | NM_005681 | TAF1A | 1.33 |
| NM_012222 | MUTYH | 1.33 | NM_005626 | SFRS4 | 1.33 |
| NM_004894 | C14ORF2 | 1.33 | NM_017893 | SEMA4G | 1.33 |
| NM_138775 | LOC91801 | 1.33 | XM_071061 | ARID3C | 1.33 |
| XM_370918 | DKFZP434P162 | 1.33 | NM_173663 | NY-REN-7 | 1.33 |
| XM_379597 | FLJ10489 | 1.33 | XM_060278 | LOC126987 | 1.33 |
| XM_292301 | LOC341912 | 1.33 | XM_293656 | LOC339951 | 1.33 |
| XM_371603 | LOC389085 | 1.33 | NM_022048 | CSNK1G1 | 1.34 |
| NM_000953 | PTGDR | 1.34 | NM_018904 | PCDHA13 | 1.34 |
| NM_006497 | HIC1 | 1.34 | NM_001329 | CTBP2 | 1.34 |
| NM_005427 | TP73 | 1.34 | NM_002586 | PBX2 | 1.34 |
| NM_001530 | HIF1A | 1.34 | NM_016614 | TTRAP | 1.34 |
| NM_005103 | FEZ1 | 1.34 | XM_496845 | LFNG | 1.34 |
| NM_001116 | ADCY9 | 1.34 | NM_003603 | ARGBP2 | 1.34 |
| NM_005794 | DHRS2 | 1.34 | NM_007020 | U1SNRNPBP | 1.34 |
| NM_014927 | CNKSR2 | 1.34 | NM_020172 | SPPL2B | 1.34 |
| NM_020191 | MRPS22 | 1.34 | XM_208522 | KIAA1394 | 1.34 |
| NM_020825 | CRAMP1L | 1.34 | NM_032576 | CYORF15B | 1.34 |
| NM_198076 | FAM36A | 1.34 | XM_372038 | FLJ32731 | 1.34 |
| NM_145055 | C18ORF25 | 1.34 | XM_378980 | LOC339568 | 1.34 |

| | | |
|---|---|---|
| XM_371691 | LOC389203 | 1.34 |
| XM_371230 | LOC388605 | 1.34 |
| XM_370704 | LOC387880 | 1.34 |
| NM_006180 | NTRK2 | 1.35 |
| NM_012102 | RERE | 1.35 |
| NM_001165 | BIRC3 | 1.35 |
| NM_002846 | PTPRN | 1.35 |
| NM_001672 | ASIP | 1.35 |
| NM_017900 | AKIP | 1.35 |
| NM_000699 | AMY2A | 1.35 |
| NM_004255 | COX5A | 1.35 |
| NM_015192 | PLCB1 | 1.35 |
| NM_014475 | DHDH | 1.35 |
| NM_018445 | SELS | 1.35 |
| XM_209234 | DKFZP434E1410 | 1.35 |
| NM_014413 | HRI | 1.36 |
| NM_012340 | NFATC2 | 1.36 |
| NM_003221 | TFAP2B | 1.36 |
| NM_004359 | CDC34 | 1.36 |
| NM_000705 | ATP4B | 1.36 |
| NM_005964 | MYH10 | 1.36 |
| NM_031279 | AGXT2L1 | 1.36 |
| NM_005046 | KLK7 | 1.36 |
| NM_014887 | PFAAP5 | 1.36 |
| NM_015634 | KIAA1279 | 1.36 |
| NM_018928 | PCDHGC4 | 1.36 |
| NM_024546 | C13ORF7 | 1.36 |
| NM_177964 | LOC130576 | 1.36 |
| NM_152545 | RASGEF1B | 1.36 |
| NM_003582 | DYRK3 | 1.37 |
| NM_015725 | RDH8 | 1.37 |
| NM_016638 | ARL6IP4 | 1.37 |
| NM_005610 | RBBP4 | 1.37 |
| NM_014792 | KIAA0125 | 1.37 |
| NM_020742 | NLGN4X | 1.37 |
| NM_172251 | MRPL54 | 1.37 |
| NM_145017 | FLJ32771 | 1.37 |
| NM_178537 | BETA4GALNAC-T4 | 1.37 |
| XM_211871 | LOC285382 | 1.37 |
| XM_208438 | LOC286495 | 1.37 |
| XM_371655 | LOC389137 | 1.37 |
| NM_015353 | KCTD2 | 1.37 |
| NM_004720 | EDG4 | 1.38 |
| NM_000446 | PON1 | 1.38 |
| NM_003771 | KRTHA6 | 1.38 |
| NM_000392 | ABCC2 | 1.38 |
| XM_372969 | LOC391475 | 1.34 |
| XM_379623 | LOC401492 | 1.34 |
| XM_373646 | LOC388170 | 1.34 |
| NM_012117 | CBX5 | 1.35 |
| NM_003254 | TIMP1 | 1.35 |
| NM_000259 | MYO5A | 1.35 |
| NM_000885 | ITGA4 | 1.35 |
| NM_001955 | EDN1 | 1.35 |
| NM_015629 | PRPF31 | 1.35 |
| NM_003162 | STRN | 1.35 |
| NM_014962 | BTBD3 | 1.35 |
| NM_014329 | RCD-8 | 1.35 |
| NM_017493 | HSHIN1 | 1.35 |
| NM_032345 | PYM | 1.35 |
| NM_194072 | HILS1 | 1.35 |
| NM_004103 | PTK2B | 1.36 |
| NM_005269 | GLI | 1.36 |
| NM_001055 | SULT1A1 | 1.36 |
| NM_012292 | HA-1 | 1.36 |
| NM_001382 | DPAGT1 | 1.36 |
| NM_003021 | SGTA | 1.36 |
| NM_002771 | PRSS3 | 1.36 |
| NM_014798 | PLEKHM1 | 1.36 |
| NM_014953 | KIAA1008 | 1.36 |
| NM_021253 | TRIM39 | 1.36 |
| NM_020851 | KIAA1465 | 1.36 |
| NM_198086 | JUB | 1.36 |
| NM_148960 | CLDN19 | 1.36 |
| XM_373973 | LOC388923 | 1.36 |
| NM_004834 | MAP4K4 | 1.37 |
| NM_012157 | FBXL2 | 1.37 |
| NM_000290 | PGAM2 | 1.37 |
| XM_376007 | DEPDC5 | 1.37 |
| XM_051017 | KIAA0657 | 1.37 |
| NM_032181 | FLJ13391 | 1.37 |
| NM_198462 | FLJ46154 | 1.37 |
| NM_194298 | SLC16A9 | 1.37 |
| XM_376845 | FLJ30435 | 1.37 |
| XM_208859 | LOC283816 | 1.37 |
| XM_372777 | LOC391030 | 1.37 |
| NM_001001417 | LOC414059 | 1.37 |
| NM_005923 | MAP3K5 | 1.38 |
| NM_015872 | ZFP67 | 1.38 |
| NM_012428 | SDFR1 | 1.38 |
| NM_000084 | CLCN5 | 1.38 |
| NM_001618 | PARP1 | 1.38 |

| | | | | | |
|---|---|---|---|---|---|
| XM_370635 | KIAA0280 | 1.38 | NM_018386 | FLJ11305 | 1.38 |
| NM_023039 | ANKRA2 | 1.38 | NM_032361 | THOC3 | 1.38 |
| NM_152460 | FLJ31882 | 1.38 | NM_194292 | DKFZP761A078 | 1.38 |
| NM_207418 | MGC57827 | 1.38 | XM_117100 | LOC201484 | 1.38 |
| XM_377756 | LOC402095 | 1.38 | XM_374056 | LOC389160 | 1.38 |
| XM_374140 | LOC389331 | 1.38 | XM_371718 | LOC389246 | 1.38 |
| XM_379628 | LOC401499 | 1.38 | NM_213608 | UNQ6411 | 1.38 |
| NM_005858 | AKAP8 | 1.39 | NM_145259 | ACVR1C | 1.39 |
| NM_002062 | GLP1R | 1.39 | NM_007129 | ZIC2 | 1.39 |
| NM_030756 | TCF7L2 | 1.39 | NM_002601 | PDE6D | 1.39 |
| NM_020935 | USP37 | 1.39 | NM_025209 | EPC1 | 1.39 |
| NM_000592 | C4B | 1.39 | NM_024629 | KLIP1 | 1.39 |
| NM_007345 | ZNF236 | 1.39 | NM_005770 | SERF2 | 1.39 |
| NM_014856 | KIAA0476 | 1.39 | NM_019088 | PD2 | 1.39 |
| NM_024717 | FLJ22344 | 1.39 | NM_032359 | MGC4308 | 1.39 |
| NM_178857 | RP1L1 | 1.39 | NM_138421 | LOC113174 | 1.39 |
| XM_168583 | MUC17 | 1.39 | NM_152522 | MGC33864 | 1.39 |
| NM_173554 | C10ORF107 | 1.39 | NM_207186 | OR10A4 | 1.39 |
| XM_211028 | LOC283403 | 1.39 | NM_178340 | APRG1 | 1.39 |
| XM_291268 | GRINA | 1.39 | XM_062025 | LOC120364 | 1.39 |
| XM_066585 | LOC139263 | 1.39 | NM_006639 | CYSLTR1 | 1.4 |
| NM_000315 | PTH | 1.4 | NM_000912 | OPRK1 | 1.4 |
| NM_004214 | FIBP | 1.4 | NM_181844 | BCL6B | 1.4 |
| NM_023078 | PYCRL | 1.4 | NM_000546 | TP53 | 1.4 |
| NM_000557 | GDF5 | 1.4 | NM_005036 | PPARA | 1.4 |
| NM_007371 | BRD3 | 1.4 | NM_002307 | LGALS7 | 1.4 |
| NM_033262 | SLC8A3 | 1.4 | NM_005074 | SLC17A1 | 1.4 |
| NM_004649 | C21ORF33 | 1.4 | NM_001001434 | STX16 | 1.4 |
| NM_018219 | FLJ10786 | 1.4 | NM_024093 | MGC5509 | 1.4 |
| NM_016647 | LOC51337 | 1.4 | XM_173140 | LOC253254 | 1.4 |
| NM_001625 | AK2 | 1.41 | NM_021624 | HRH4 | 1.41 |
| NM_018907 | PCDHA4 | 1.41 | NM_003220 | TFAP2A | 1.41 |
| NM_006120 | HLA-DMA | 1.41 | NM_004912 | CCM1 | 1.41 |
| NM_139178 | DEPC-1 | 1.41 | NM_000835 | GRIN2C | 1.41 |
| NM_020197 | SMYD2 | 1.41 | NM_001468 | GAGE1 | 1.41 |
| NM_004588 | SCN2B | 1.41 | NM_012133 | COPG2 | 1.41 |
| NM_017776 | FLJ20344 | 1.41 | NM_018921 | PCDHGA9 | 1.41 |
| NM_052851 | STARD13 | 1.41 | NM_052937 | LOC115294 | 1.41 |
| NM_144684 | MGC32104 | 1.41 | NM_173854 | SLC41A1 | 1.41 |
| NM_207427 | LOC399851 | 1.41 | XM_374655 | LOC392997 | 1.41 |
| XM_373778 | LOC388477 | 1.41 | NM_001001418 | MGC44903 | 1.41 |
| NM_000958 | PTGER4 | 1.42 | NM_030908 | OR2A4 | 1.42 |
| NM_004095 | EIF4EBP1 | 1.42 | NM_014786 | ARHGEF17 | 1.42 |
| NM_001457 | FLNB | 1.42 | NM_018937 | PCDHB3 | 1.42 |
| NM_018667 | SMPD3 | 1.42 | NM_001064 | TKT | 1.42 |
| NM_001173 | ARHGAP5 | 1.42 | NM_006010 | ARMET | 1.42 |

| | | | | | |
|---|---|---|---|---|---|
| NM_025252 | RAPH1 | 1.42 | NM_003043 | SLC6A6 | 1.42 |
| NM_006757 | TNNT3 | 1.42 | NM_006344 | CLECSF14 | 1.42 |
| NM_015101 | C1ORF17 | 1.42 | NM_145200 | CABP4 | 1.42 |
| NM_020918 | GPAM | 1.42 | NM_022773 | FLJ12681 | 1.42 |
| NM_031476 | DKFZP434B044 | 1.42 | NM_032527 | KIAA1847 | 1.42 |
| NM_144774 | SMCR5 | 1.42 | NM_152569 | C9ORF66 | 1.42 |
| XM_374600 | LOC392862 | 1.42 | XM_370639 | LOC387791 | 1.42 |
| XM_370991 | LOC388297 | 1.42 | NM_001005289 | OR52H1 | 1.42 |
| NM_001005492 | OR5J2 | 1.42 | NM_001798 | CDK2 | 1.43 |
| NM_024504 | PRDM14 | 1.43 | NM_004497 | FOXA3 | 1.43 |
| NM_002384 | MBD1 | 1.43 | NM_000855 | GUCY1A2 | 1.43 |
| NM_004402 | DFFB | 1.43 | NM_001256 | CDC27 | 1.43 |
| NM_002571 | PAEP | 1.43 | NM_014736 | KIAA0101 | 1.43 |
| NM_006576 | AVIL | 1.43 | NM_015033 | FNBP1 | 1.43 |
| XM_371891 | KIAA0877 | 1.43 | NM_014173 | HSPC142 | 1.43 |
| AF120323 | TIAM2 | 1.43 | NM_016274 | CKIP-1 | 1.43 |
| NM_018995 | MOV10L1 | 1.43 | NM_017924 | C14ORF119 | 1.43 |
| NM_017944 | USP47 | 1.43 | NM_023935 | C20ORF116 | 1.43 |
| NM_032452 | JPH4 | 1.43 | NM_032819 | ZNF341 | 1.43 |
| NM_032845 | FLJ14816 | 1.43 | XM_044062 | DKFZP761O2018 | 1.43 |
| NM_178160 | OTOP2 | 1.43 | NM_152670 | FLJ25369 | 1.43 |
| XM_211460 | LOC284434 | 1.43 | XM_170909 | LOC257177 | 1.43 |
| XM_117268 | LOC200731 | 1.43 | XM_373561 | LOC387915 | 1.43 |
| NM_001006607 | LOC474170 | 1.43 | NM_020439 | CAMK1G | 1.44 |
| NM_001221 | CAMK2D | 1.44 | NM_018323 | PI4K2B | 1.44 |
| NM_033101 | LGALS12 | 1.44 | NM_003317 | TITF1 | 1.44 |
| NM_003032 | SIAT1 | 1.44 | NM_014220 | TM4SF1 | 1.44 |
| NM_004530 | MMP2 | 1.44 | NM_177417 | KLC2L | 1.44 |
| NM_022832 | USP46 | 1.44 | NM_199336 | DKFZP434N062 | 1.44 |
| NM_001927 | DES | 1.44 | NM_019013 | FAM64A | 1.44 |
| NM_001425 | EMP3 | 1.44 | NM_003156 | STIM1 | 1.44 |
| NM_004890 | SPAG7 | 1.44 | NM_005107 | ENDOGL1 | 1.44 |
| NM_019006 | AWP1 | 1.44 | NM_017602 | DKFZP761A052 | 1.44 |
| NM_018180 | DHX32 | 1.44 | NM_020318 | PLAC3 | 1.44 |
| NM_021215 | C20ORF77 | 1.44 | NM_024727 | FLJ23259 | 1.44 |
| NM_024315 | C7ORF23 | 1.44 | NM_031305 | ARHGAP24 | 1.44 |
| NM_052874 | STX1B2 | 1.44 | NM_144623 | FLJ32784 | 1.44 |
| NM_145028 | C6ORF81 | 1.44 | NM_178128 | LOC283985 | 1.44 |
| NM_206912 | C6ORF216 | 1.44 | NM_203452 | MGC39545 | 1.44 |
| XM_373035 | LOC391705 | 1.44 | XM_372473 | LOC390352 | 1.44 |
| XM_378685 | LOC400605 | 1.44 | NM_001841 | CNR2 | 1.45 |
| NM_014946 | SPG4 | 1.45 | NM_005270 | GLI2 | 1.45 |
| NM_001439 | EXTL2 | 1.45 | NM_016262 | TUBE1 | 1.45 |
| NM_145809 | LOC220594 | 1.45 | NM_000395 | CSF2RB | 1.45 |
| NM_001945 | DTR | 1.45 | NM_000171 | GLRA1 | 1.45 |
| NM_004324 | BAX | 1.45 | NM_004291 | CART | 1.45 |

| | | | | | |
|---|---|---|---|---|---|
| NM_004895 | CIAS1 | 1.45 | NM_014622 | LOH11CR2A | 1.45 |
| NM_002078 | GOLGA4 | 1.45 | XM_371474 | PLXNB2 | 1.45 |
| NM_016306 | DNAJB11 | 1.45 | NM_144736 | PRO1853 | 1.45 |
| NM_018452 | C6ORF35 | 1.45 | NM_033518 | SLC38A5 | 1.45 |
| NM_153010 | C18ORF16 | 1.45 | XM_376440 | LOC285629 | 1.45 |
| XM_371181 | NANOS2 | 1.45 | XM_372349 | LOC390038 | 1.45 |
| NM_004935 | CDK5 | 1.46 | XM_375726 | KIAA0494 | 1.46 |
| NM_003815 | ADAM15 | 1.46 | NM_021252 | RAB18 | 1.46 |
| NM_004762 | PSCD1 | 1.46 | NM_002171 | IFNA10 | 1.46 |
| NM_005486 | TOM1L1 | 1.46 | NM_005821 | NBR2 | 1.46 |
| NM_000198 | HSD3B2 | 1.46 | NM_005727 | TSPAN-1 | 1.46 |
| NM_006370 | VTI1B | 1.46 | NM_015436 | RCHY1 | 1.46 |
| NM_015896 | ZMYND10 | 1.46 | NM_017423 | GALNT7 | 1.46 |
| NM_020357 | PCNP | 1.46 | NM_022774 | FLJ21144 | 1.46 |
| NM_024671 | FLJ23436 | 1.46 | NM_025258 | C6ORF27 | 1.46 |
| NM_177532 | RASSF6 | 1.46 | XM_294794 | LOC339065 | 1.46 |
| XM_371829 | LOC389405 | 1.46 | XM_044630 | DKFZP434C131 | 1.47 |
| NM_021813 | BACH2 | 1.47 | NM_020310 | MNT | 1.47 |
| NM_000635 | RFX2 | 1.47 | NM_015216 | KIAA0433 | 1.47 |
| NM_000236 | LIPC | 1.47 | NM_031407 | HUWE1 | 1.47 |
| NM_014584 | ERO1L | 1.47 | NM_005574 | LMO2 | 1.47 |
| NM_002863 | PYGL | 1.47 | NM_000896 | CYP4F3 | 1.47 |
| NM_004192 | ASMTL | 1.47 | NM_005886 | KATNB1 | 1.47 |
| NM_014982 | PCNX | 1.47 | NM_024624 | SMC6L1 | 1.47 |
| NM_024122 | MGC4825 | 1.47 | NM_033200 | BC002942 | 1.47 |
| NM_033254 | BOC | 1.47 | NM_174926 | MGC17839 | 1.47 |
| NM_178509 | STXBP4 | 1.47 | XM_209155 | LOC284371 | 1.47 |
| NM_207474 | FLJ42953 | 1.47 | XM_374144 | LOC389335 | 1.47 |
| NM_004760 | STK17A | 1.48 | NM_001706 | BCL6 | 1.48 |
| NM_021964 | ZNF148 | 1.48 | NM_000302 | PLOD | 1.48 |
| NM_003283 | TNNT1 | 1.48 | NM_006568 | CGRRF1 | 1.48 |
| NM_012310 | KIF4A | 1.48 | NM_014992 | DAAM1 | 1.48 |
| NM_001866 | COX7B | 1.48 | NM_033161 | SURF4 | 1.48 |
| NM_003634 | NIPSNAP1 | 1.48 | NM_003720 | DSCR2 | 1.48 |
| NM_015046 | KIAA0625 | 1.48 | NM_014403 | SIAT7D | 1.48 |
| NM_016048 | CGI-111 | 1.48 | NM_138332 | C21ORF41 | 1.48 |
| NM_024056 | MGC5576 | 1.48 | NM_152307 | FLJ40452 | 1.48 |
| XM_350780 | FAM21C | 1.48 | XM_370866 | FLJ25756 | 1.48 |
| XM_379474 | LOC401310 | 1.48 | XM_380142 | LOC402591 | 1.48 |
| NM_002960 | S100A3 | 1.49 | NM_005521 | TLX1 | 1.49 |
| NM_006088 | TUBB2 | 1.49 | NM_003217 | TEGT | 1.49 |
| NM_020166 | MCCC1 | 1.49 | NM_024079 | ALG8 | 1.49 |
| NM_003495 | HIST1H4I | 1.49 | NM_004868 | GPSN2 | 1.49 |
| NM_021148 | ZNF273 | 1.49 | NM_024734 | CLMN | 1.49 |
| NM_024868 | FLJ14124 | 1.49 | NM_030642 | APOL5 | 1.49 |
| NM_145647 | MGC21654 | 1.49 | NM_194291 | LOC157378 | 1.49 |

| | | |
|---|---|---|
| NM_182542 | FLJ32682 | 1.49 |
| XM_371384 | LOC388776 | 1.49 |
| XM_375590 | LOC400682 | 1.49 |
| NM_005158 | ABL2 | 1.5 |
| NM_005958 | MTNR1A | 1.5 |
| NM_002144 | HOXB1 | 1.5 |
| NM_004215 | EBAG9 | 1.5 |
| NM_017617 | NOTCH1 | 1.5 |
| NM_014636 | RALGPS1A | 1.5 |
| NM_021079 | NMT1 | 1.5 |
| NM_001056 | SULT1C1 | 1.5 |
| NM_018233 | FLJ10826 | 1.5 |
| NM_022071 | SH2D4A | 1.5 |
| XM_371257 | KIAA1799 | 1.5 |
| XM_058681 | C14ORF66 | 1.5 |
| NM_173524 | C10ORF64 | 1.5 |
| XM_064265 | LOC124685 | 1.5 |
| XM_373574 | LOC387943 | 1.5 |
| NM_017672 | TRPM7 | 1.51 |
| NM_004540 | NCAM2 | 1.51 |
| NM_003290 | TPM4 | 1.51 |
| NM_001332 | CTNND2 | 1.51 |
| NM_001878 | CRABP2 | 1.51 |
| NM_006468 | POLR3C | 1.51 |
| XM_374927 | KIAA1377 | 1.51 |
| NM_032231 | FLJ22875 | 1.51 |
| NM_152624 | DCP2 | 1.51 |
| XM_212061 | LOC285872 | 1.51 |
| NM_212550 | BLOC1S3 | 1.51 |
| NM_003667 | GPR49 | 1.52 |
| NM_012238 | SIRT1 | 1.52 |
| NM_016639 | TNFRSF12A | 1.52 |
| NM_005668 | SIAT8D | 1.52 |
| XM_375851 | KIAA0133 | 1.52 |
| XM_032945 | C21ORF25 | 1.52 |
| XM_031744 | STARD9 | 1.52 |
| NM_152683 | FLJ33167 | 1.52 |
| XM_167044 | SLC35F1 | 1.52 |
| XM_374035 | LOC389100 | 1.52 |
| XM_378329 | LOC399983 | 1.52 |
| NM_212558 | LOC401498 | 1.52 |
| NM_000118 | ENG | 1.53 |
| NM_002393 | MDM4 | 1.53 |
| NM_018725 | IL17RB | 1.53 |
| NM_002077 | GOLGA1 | 1.53 |
| NM_080282 | ABCA10 | 1.53 |
| XM_209597 | LOC285407 | 1.49 |
| XM_373490 | LOC387754 | 1.49 |
| NM_001004195 | OR4F4 | 1.49 |
| NM_002645 | PIK3C2A | 1.5 |
| NM_003278 | TNA | 1.5 |
| NM_014754 | PTDSS1 | 1.5 |
| NM_004685 | MTMR6 | 1.5 |
| NM_001152 | SLC25A5 | 1.5 |
| NM_002492 | NDUFB5 | 1.5 |
| NM_022496 | ACTR6 | 1.5 |
| XM_041162 | NDFIP2 | 1.5 |
| NM_019607 | FLJ11267 | 1.5 |
| NM_030781 | COLEC12 | 1.5 |
| NM_032843 | FIBCD1 | 1.5 |
| XM_291111 | GPR125 | 1.5 |
| NM_176823 | S100A15 | 1.5 |
| XM_294567 | LOC347252 | 1.5 |
| NM_012407 | PRKCABP | 1.51 |
| NM_006644 | HSPH1 | 1.51 |
| NM_001972 | ELA2 | 1.51 |
| NM_020445 | ARP3BETA | 1.51 |
| NM_001161 | NUDT2 | 1.51 |
| NM_003769 | SFRS9 | 1.51 |
| NM_020394 | SBZF3 | 1.51 |
| NM_025063 | FLJ23550 | 1.51 |
| NM_153811 | SLC38A6 | 1.51 |
| NM_203381 | MGC71805 | 1.51 |
| XM_376725 | LOC401433 | 1.51 |
| NM_033214 | GK2 | 1.52 |
| NM_005523 | HOXA11 | 1.52 |
| NM_000759 | CSF3 | 1.52 |
| NM_006818 | AF1Q | 1.52 |
| NM_014847 | NICE-4 | 1.52 |
| NM_006604 | RFPL3 | 1.52 |
| NM_017774 | CDKAL1 | 1.52 |
| NM_032321 | MGC13057 | 1.52 |
| NM_145304 | C7ORF33 | 1.52 |
| NM_153265 | FLJ35827 | 1.52 |
| XM_379975 | LOC402625 | 1.52 |
| XM_373567 | LOC387932 | 1.52 |
| NM_001932 | MPP3 | 1.53 |
| NM_000917 | P4HA1 | 1.53 |
| NM_002122 | HLA-DQA1 | 1.53 |
| NM_002266 | KPNA2 | 1.53 |
| NM_006893 | LGTN | 1.53 |
| NM_024722 | ACBD4 | 1.53 |

| | | | | | |
|---|---|---|---|---|---|
| NM_014670 | BZW1 | 1.53 | NM_016255 | FAM8A1 | 1.53 |
| NM_016482 | C9ORF78 | 1.53 | NM_018011 | FLJ10154 | 1.53 |
| NM_018303 | SEC5L1 | 1.53 | NM_020932 | KIAA1587 | 1.53 |
| NM_032230 | FLJ22789 | 1.53 | NM_139168 | SFRS12 | 1.53 |
| NM_152901 | PYC1 | 1.53 | XM_370946 | LOC388226 | 1.53 |
| XM_060943 | LOC128322 | 1.53 | NM_002036 | FY | 1.54 |
| NM_000535 | PMS2 | 1.54 | NM_020992 | PDLIM1 | 1.54 |
| NM_002495 | NDUFS4 | 1.54 | NM_000741 | CHRM4 | 1.54 |
| NM_000585 | IL15 | 1.54 | NM_000256 | MYBPC3 | 1.54 |
| NM_005208 | CRYBA1 | 1.54 | NM_001960 | EEF1D | 1.54 |
| NM_017829 | CECR5 | 1.54 | NM_016564 | BM88 | 1.54 |
| NM_020406 | PRV1 | 1.54 | NM_022477 | NDRG3 | 1.54 |
| NM_032602 | CX62 | 1.54 | NM_032497 | ZNF559 | 1.54 |
| NM_198448 | UNQ429 | 1.54 | NM_173541 | C10ORF91 | 1.54 |
| NM_199047 | TBPL2 | 1.54 | XM_379741 | LOC401701 | 1.54 |
| XM_379716 | LOC401599 | 1.54 | NM_013941 | OR10C1 | 1.54 |
| NM_152327 | AK7 | 1.55 | NM_006915 | RP2 | 1.55 |
| NM_004310 | RHOH | 1.55 | NM_001534 | TLX2 | 1.55 |
| NM_006741 | PPP1R1A | 1.55 | NM_005434 | BENE | 1.55 |
| NM_000882 | IL12A | 1.55 | NM_006544 | SEC10L1 | 1.55 |
| NM_001195 | BFSP1 | 1.55 | NM_001542 | IGSF3 | 1.55 |
| NM_003796 | C19ORF2 | 1.55 | NM_016611 | KCNK4 | 1.55 |
| NM_017548 | H41 | 1.55 | NM_020189 | E(Y)2 | 1.55 |
| NM_152377 | MGC34837 | 1.55 | NM_198152 | URP | 1.55 |
| XM_378178 | MGC9913 | 1.55 | XM_093087 | LOC170067 | 1.55 |
| NM_001005499 | OR6C70 | 1.55 | NM_012093 | AK5 | 1.56 |
| NM_002753 | MAPK10 | 1.56 | NM_005087 | FXR1 | 1.56 |
| NM_000344 | SMN1 | 1.56 | NM_000572 | IL10 | 1.56 |
| NM_018244 | C20ORF44 | 1.56 | NM_006017 | PROM1 | 1.56 |
| NM_005444 | RQCD1 | 1.56 | NM_007032 | HRIHFB2122 | 1.56 |
| NM_007049 | BTN2A1 | 1.56 | NM_018835 | MNAB | 1.56 |
| NM_031275 | TEX12 | 1.56 | NM_024680 | FLJ23311 | 1.56 |
| NM_198148 | CPXM2 | 1.56 | NM_152792 | FLJ25084 | 1.56 |
| XM_371138 | LOC284390 | 1.56 | XM_373931 | LOC388823 | 1.56 |
| XM_372908 | LOC391340 | 1.56 | NM_014002 | IKBKE | 1.57 |
| NM_001943 | DSG2 | 1.57 | NM_004820 | CYP7B1 | 1.57 |
| NM_005588 | MEP1A | 1.57 | NM_003347 | UBE2L3 | 1.57 |
| NM_007315 | STAT1 | 1.57 | NM_004070 | CLCNKA | 1.57 |
| NM_004937 | CTNS | 1.57 | NR_001562 | ANXA2P1 | 1.57 |
| XM_290821 | EGFL4 | 1.57 | NM_013338 | ALG5 | 1.57 |
| NM_012327 | PIGN | 1.57 | NM_019036 | HMGCLL1 | 1.57 |
| NM_020156 | C1GALT1 | 1.57 | NM_020845 | PITPNM2 | 1.57 |
| NM_020779 | WDR35 | 1.57 | NM_024738 | FLJ21415 | 1.57 |
| XM_378549 | LOC91948 | 1.57 | NM_144589 | COMTD1 | 1.57 |
| NM_152546 | FLJ25286 | 1.57 | XM_371190 | LOC162967 | 1.57 |
| NM_213603 | LOC285989 | 1.57 | XM_376207 | LOC401054 | 1.57 |

| | | |
|---|---|---|
| XM_375603 | LOC400688 | 1.57 |
| NM_014326 | DAPK2 | 1.58 |
| NM_020948 | MI-ER1 | 1.58 |
| NM_000502 | EPX | 1.58 |
| NM_001096 | ACLY | 1.58 |
| NM_014411 | NSG-X | 1.58 |
| NM_032683 | MGC12972 | 1.58 |
| XM_114618 | LOC203069 | 1.58 |
| NM_198284 | LOC349114 | 1.58 |
| NM_023038 | ADAM19 | 1.59 |
| NM_017621 | FLJ20013 | 1.59 |
| NM_178463 | C20ORF166 | 1.59 |
| NM_152611 | C20ORF75 | 1.59 |
| NM_002090 | CXCL3 | 1.6 |
| NM_005487 | HMG2L1 | 1.6 |
| NM_001230 | CASP10 | 1.6 |
| NM_000079 | CHRNA1 | 1.6 |
| NM_004887 | CXCL14 | 1.6 |
| NM_014421 | DKK2 | 1.6 |
| XM_166132 | KIAA1462 | 1.6 |
| NM_052936 | APG4A | 1.6 |
| XM_092778 | LOC164395 | 1.6 |
| XM_210042 | LOC286423 | 1.6 |
| XM_375631 | LOC400707 | 1.6 |
| NM_006035 | CDC42BPB | 1.61 |
| NM_005194 | CEBPB | 1.61 |
| NM_005263 | GFI1 | 1.61 |
| NM_000022 | ADA | 1.61 |
| NM_003205 | TCF12 | 1.61 |
| NM_003339 | UBE2D2 | 1.61 |
| NM_020384 | CLDN2 | 1.61 |
| NM_012430 | SEC22L2 | 1.61 |
| NM_031896 | CACNG7 | 1.61 |
| NM_138959 | VANGL1 | 1.61 |
| NM_033421 | C20ORF161 | 1.61 |
| XM_376309 | LOC285540 | 1.61 |
| NM_006712 | FASTK | 1.62 |
| NM_015149 | RGL1 | 1.62 |
| NM_003374 | VDAC1 | 1.62 |
| NM_016055 | MRPL48 | 1.62 |
| NM_024903 | FLJ14297 | 1.62 |
| NM_054029 | C8ORF14 | 1.62 |
| NM_001364 | DLG2 | 1.63 |
| NM_004646 | NPHS1 | 1.63 |
| NM_006600 | NUDC | 1.63 |
| NM_005834 | TIMM17B | 1.63 |
| XM_371140 | LOC388507 | 1.57 |
| NM_033450 | ABCC10 | 1.58 |
| NM_002703 | PPAT | 1.58 |
| BC007782 | IGLJ3 | 1.58 |
| NM_014668 | GREB1 | 1.58 |
| NM_030594 | CPEB1 | 1.58 |
| NM_152407 | GRPEL2 | 1.58 |
| NM_178828 | C9ORF79 | 1.58 |
| XM_171150 | LOC254027 | 1.58 |
| NM_016487 | C6ORF203 | 1.59 |
| NM_182503 | DEADC1 | 1.59 |
| NM_153183 | NUDT10 | 1.59 |
| NM_152997 | C4ORF7 | 1.59 |
| NM_018654 | GPRC5D | 1.6 |
| NM_002572 | PAFAH1B2 | 1.6 |
| NM_012173 | FBXO25 | 1.6 |
| NM_002274 | KRT13 | 1.6 |
| NM_017411 | SMN2 | 1.6 |
| NM_018641 | CHST12 | 1.6 |
| NM_022830 | RBM21 | 1.6 |
| NM_174905 | LOC147965 | 1.6 |
| XM_379586 | LOC286186 | 1.6 |
| XM_372376 | LOC390113 | 1.6 |
| XM_378970 | LOC400839 | 1.6 |
| NM_003507 | FZD7 | 1.61 |
| XM_039762 | MYT1L | 1.61 |
| NM_002397 | MEF2C | 1.61 |
| NM_003425 | ZNF45 | 1.61 |
| NM_002133 | HMOX1 | 1.61 |
| NM_021642 | FCGR2A | 1.61 |
| NM_001393 | ECM2 | 1.61 |
| NM_014358 | CLECSF9 | 1.61 |
| NM_024698 | SLC25A22 | 1.61 |
| NM_032494 | ZC3HDC8 | 1.61 |
| NM_019016 | KRT24 | 1.61 |
| XM_291269 | KIAA1875 | 1.61 |
| NM_002587 | PCDH1 | 1.62 |
| NM_004467 | FGL1 | 1.62 |
| XM_042635 | KIAA1069 | 1.62 |
| NM_025130 | FLJ22761 | 1.62 |
| NM_019014 | POLR1B | 1.62 |
| NM_173700 | FLJ39821 | 1.62 |
| NM_003319 | TTN | 1.63 |
| NM_000603 | NOS3 | 1.63 |
| NM_022658 | HOXC8 | 1.63 |
| NM_006451 | PAIP1 | 1.63 |

| | | | | | |
|---|---|---|---|---|---|
| NM_016360 | LOC51204 | 1.63 | NM_194294 | LOC169355 | 1.63 |
| XM_097622 | LOC149297 | 1.63 | XM_089281 | LOC149281 | 1.63 |
| XM_068632 | LOC133993 | 1.63 | XM_374079 | LOC389204 | 1.63 |
| XM_375165 | LOC390535 | 1.63 | XM_374227 | LOC389534 | 1.63 |
| NM_175620 | MTM | 1.63 | NM_001291 | CLK2 | 1.64 |
| NM_004473 | FOXE1 | 1.64 | NM_003222 | TFAP2C | 1.64 |
| NM_006847 | LILRB4 | 1.64 | NM_020462 | KIAA1181 | 1.64 |
| NM_001340 | CYLC2 | 1.64 | NM_005035 | POLRMT | 1.64 |
| NM_003904 | ZNF259 | 1.64 | NM_015853 | LOC51035 | 1.64 |
| NM_016483 | PHF7 | 1.64 | NM_153646 | SLC24A4 | 1.64 |
| NM_152425 | FLJ40249 | 1.64 | NM_173639 | FLJ35976 | 1.64 |
| XM_209656 | LOC285550 | 1.64 | XM_379495 | LOC401358 | 1.64 |
| NM_001006120 | RBMY1D | 1.64 | NM_000459 | TEK | 1.65 |
| NM_000406 | GNRHR | 1.65 | NM_000333 | SCA7 | 1.65 |
| NM_003602 | FKBP6 | 1.65 | NM_006113 | VAV3 | 1.65 |
| NM_030775 | WNT5B | 1.65 | NM_021008 | DEAF1 | 1.65 |
| NM_172341 | PEN2 | 1.65 | NM_152374 | FLJ38984 | 1.65 |
| XM_371722 | LOC389257 | 1.65 | XM_376861 | LOC401520 | 1.65 |
| NM_033126 | PSKH2 | 1.66 | NM_013296 | GPSM2 | 1.66 |
| NM_000921 | PDE3A | 1.66 | NM_004241 | JMJD1C | 1.66 |
| NM_006365 | CROC4 | 1.66 | NM_004993 | MJD | 1.66 |
| NM_006801 | KDELR1 | 1.66 | NM_024114 | TRIM48 | 1.66 |
| NM_032847 | FLJ14825 | 1.66 | NM_152334 | FLJ25005 | 1.66 |
| XM_371285 | LOC128102 | 1.66 | NM_139165 | RAET1E | 1.66 |
| NM_153341 | IBRDC3 | 1.66 | XM_378786 | LOC148145 | 1.66 |
| XM_088691 | DGAT2L3 | 1.66 | XM_378983 | LOC284749 | 1.66 |
| NM_030630 | C17ORF28 | 1.66 | NM_183379 | TESSP1 | 1.66 |
| XM_172389 | LOC256085 | 1.66 | XM_374973 | LOC400025 | 1.66 |
| NM_006193 | PAX4 | 1.67 | NM_005649 | ZNF354A | 1.67 |
| NM_006244 | PPP2R5B | 1.67 | NM_002415 | MIF | 1.67 |
| NM_014766 | SCRN1 | 1.67 | NM_020728 | KIAA1228 | 1.67 |
| NM_023080 | FLJ20989 | 1.67 | NM_024839 | RPP21 | 1.67 |
| NM_138781 | LOC113386 | 1.67 | NM_053049 | UCN3 | 1.67 |
| NM_173675 | FLJ33708 | 1.67 | NM_173831 | LOC286075 | 1.67 |
| NM_198282 | LOC340061 | 1.67 | XM_170708 | LOC255411 | 1.67 |
| XM_210908 | LOC283157 | 1.67 | XM_379262 | LOC401124 | 1.67 |
| NM_002843 | PTPRJ | 1.68 | NM_000955 | PTGER1 | 1.68 |
| NM_002550 | OR3A1 | 1.68 | NM_005319 | HIST1H1C | 1.68 |
| NM_001563 | IMPG1 | 1.68 | NM_000304 | PMP22 | 1.68 |
| NM_004337 | C8ORF1 | 1.68 | NM_181532 | ERAS | 1.68 |
| NM_032264 | AE2 | 1.68 | NM_003540 | HIST1H4F | 1.68 |
| NM_016644 | LOC51334 | 1.68 | NM_019056 | P17.3 | 1.68 |
| NM_138283 | CSTL1 | 1.68 | NM_173599 | FLJ40126 | 1.68 |
| XM_379476 | LOC401312 | 1.68 | XM_376472 | LOC401239 | 1.68 |
| NM_032427 | MAML2 | 1.69 | NM_002545 | OPCML | 1.69 |
| NM_014362 | HIBCH | 1.69 | NM_005101 | G1P2 | 1.69 |

| | | | | | |
|---|---|---|---|---|---|
| NM_002344 | LTK | 1.7 | NM_006500 | MCAM | 1.7 |
| NM_005989 | AKR1D1 | 1.7 | NM_003182 | TAC1 | 1.7 |
| NM_003243 | TGFBR3 | 1.7 | NM_173708 | MTND1 | 1.7 |
| NM_003056 | SLC19A1 | 1.7 | NM_018185 | C13ORF17 | 1.7 |
| NM_024926 | FLJ12571 | 1.7 | NM_181704 | BAGE4 | 1.7 |
| NM_145755 | STI2 | 1.7 | NM_199136 | MGC72075 | 1.7 |
| XM_375067 | LOC400197 | 1.7 | XM_372420 | LOC390217 | 1.7 |
| NM_002597 | PDC | 1.71 | NM_001773 | CD34 | 1.71 |
| NM_014977 | ACIN1 | 1.71 | NM_178568 | RTN4RL1 | 1.71 |
| NM_020041 | SLC2A9 | 1.71 | NM_007373 | SHOC2 | 1.71 |
| NM_024553 | FLJ20097 | 1.71 | NM_022663 | CTAGE1 | 1.71 |
| NM_173670 | RGMB | 1.71 | NM_203395 | DEHAL1 | 1.71 |
| XM_371302 | LOC388686 | 1.71 | NM_031219 | C9ORF158 | 1.72 |
| NM_002647 | PIK3C3 | 1.72 | NM_003172 | SURF1 | 1.72 |
| NM_194449 | PLEKHE1 | 1.72 | NM_031281 | IRTA2 | 1.72 |
| NM_002365 | MAGEB3 | 1.72 | NM_005741 | ZNF263 | 1.72 |
| NM_015485 | RWDD3 | 1.72 | NM_016350 | NIN | 1.72 |
| NM_032305 | MGC3200 | 1.72 | NM_138769 | RHOT2 | 1.72 |
| NM_138570 | MGC15523 | 1.72 | XM_085578 | FLJ46675 | 1.72 |
| XM_290714 | RAB43 | 1.72 | XM_374255 | LOC389637 | 1.72 |
| XM_379002 | LOC400861 | 1.72 | NM_001005514 | OR5H14 | 1.72 |
| NM_007043 | HRB2 | 1.73 | NM_003285 | TNR | 1.73 |
| NM_014263 | YME1L1 | 1.73 | BC038293 | PTENP1 | 1.73 |
| NM_016087 | WNT16 | 1.73 | NM_004085 | TIMM8A | 1.73 |
| NM_019845 | REPRIMO | 1.73 | NM_014938 | MONDOA | 1.73 |
| NM_018031 | WDR6 | 1.73 | NM_001185 | AZGP1 | 1.73 |
| NM_004640 | BAT1 | 1.73 | NM_005885 | 38777 | 1.73 |
| NM_005799 | INADL | 1.73 | NM_016107 | ZFR | 1.73 |
| NM_021255 | PELI2 | 1.73 | XM_047325 | THOC2 | 1.73 |
| NM_024031 | MGC3121 | 1.73 | NM_032263 | DKFZP434B227 | 1.73 |
| NM_138341 | LOC89894 | 1.73 | NM_152262 | ZNF439 | 1.73 |
| XM_374781 | LOC220906 | 1.73 | XM_373549 | LOC387887 | 1.73 |
| XM_370975 | LOC388259 | 1.73 | NM_001347 | DGKQ | 1.74 |
| NM_004350 | RUNX3 | 1.74 | NM_000528 | MAN2B1 | 1.74 |
| NM_016011 | CGI-63 | 1.74 | NM_001953 | ECGF1 | 1.74 |
| NM_005874 | LILRB2 | 1.74 | NM_006238 | PPARD | 1.74 |
| NM_144699 | ATP1A4 | 1.74 | NM_004089 | DSIPI | 1.74 |
| NM_030661 | HOXA3 | 1.74 | NM_005969 | NAP1L4 | 1.74 |
| NM_005651 | TDO2 | 1.74 | NM_015035 | ZHX3 | 1.74 |
| NM_016074 | CGI-143 | 1.74 | NM_015964 | CGI-38 | 1.74 |
| NM_016289 | MO25 | 1.74 | NM_016551 | TM7SF3 | 1.74 |
| NM_030572 | MGC10946 | 1.74 | XM_210184 | LOC283117 | 1.74 |
| XM_379639 | LOC401522 | 1.74 | NM_004936 | CDKN2B | 1.75 |
| NM_005226 | EDG3 | 1.75 | NM_003662 | PIR | 1.75 |
| NM_014491 | FOXP2 | 1.75 | NM_000314 | PTEN | 1.75 |
| NM_006705 | GADD45G | 1.75 | NM_021244 | RRAGD | 1.75 |

| | | | | | |
|---|---|---|---|---|---|
| NM_012466 | TM4-B | 1.75 | NM_152453 | MGC35118 | 1.75 |
| XM_379179 | LOC152274 | 1.75 | NM_174911 | NSE2 | 1.75 |
| NM_181716 | P30 | 1.75 | NM_182576 | MGC39821 | 1.75 |
| XM_290671 | LOC339047 | 1.75 | NM_207396 | FLJ46380 | 1.75 |
| XM_372824 | LOC391192 | 1.75 | XM_373098 | LOC391844 | 1.75 |
| XM_374137 | LOC389328 | 1.75 | XM_377514 | LOC401898 | 1.75 |
| NM_153361 | MGC42105 | 1.76 | NM_014063 | HIP-55 | 1.76 |
| NM_003864 | SAP30 | 1.76 | NM_001090 | ABCF1 | 1.76 |
| NM_001128 | AP1G1 | 1.76 | NM_004407 | DMP1 | 1.76 |
| NM_013444 | UBQLN2 | 1.76 | NM_032804 | C10ORF22 | 1.76 |
| XM_059318 | RUTBC2 | 1.76 | XM_290755 | FLJ35848 | 1.76 |
| XM_379231 | LOC401098 | 1.76 | XM_372500 | LOC390429 | 1.76 |
| XM_375224 | LOC400410 | 1.76 | XM_372638 | LOC390729 | 1.76 |
| NM_002031 | FRK | 1.77 | NM_013247 | PRSS25 | 1.77 |
| NM_006984 | CLDN10 | 1.77 | NM_004552 | NDUFS5 | 1.77 |
| NM_015670 | SENP3 | 1.77 | NM_024729 | MYH14 | 1.77 |
| NM_152769 | C19ORF26 | 1.77 | XM_376206 | LOC285375 | 1.77 |
| NM_018843 | MCFP | 1.77 | XM_371842 | LOC389421 | 1.77 |
| NM_004780 | TCEAL1 | 1.78 | NM_012212 | LTB4DH | 1.78 |
| NM_002187 | IL12B | 1.78 | NM_016150 | ASB2 | 1.78 |
| AF126749 | KLHL1AS | 1.78 | NM_017657 | AFTIPHILIN | 1.78 |
| NM_004756 | NUMBL | 1.78 | NM_003773 | HYAL2 | 1.78 |
| NM_182831 | TNT | 1.78 | NM_175901 | LOC283932 | 1.78 |
| XM_370657 | C11ORF34 | 1.78 | XM_374134 | LOC389319 | 1.78 |
| XM_373687 | LOC388278 | 1.78 | NM_006956 | ZNF12 | 1.78 |
| NM_005159 | ACTC | 1.79 | NM_002099 | GYPA | 1.79 |
| NM_001372 | DNAH9 | 1.79 | NM_018950 | HLA-F | 1.79 |
| NM_004722 | AP4M1 | 1.79 | NM_006656 | NEU3 | 1.79 |
| NM_014345 | ZNF318 | 1.79 | NM_024959 | SLC24A6 | 1.79 |
| NM_025161 | FLJ22175 | 1.79 | NM_031914 | SYT14L | 1.79 |
| XM_096317 | C10ORF73 | 1.79 | XM_351193 | SYCN | 1.79 |
| NM_194314 | FRBZ1 | 1.79 | XM_372122 | LOC389765 | 1.79 |
| XM_209429 | LOC284988 | 1.79 | XM_373734 | LOC388388 | 1.79 |
| NM_002141 | HOXA4 | 1.8 | NM_000550 | TYRP1 | 1.8 |
| NM_018315 | FBXW7 | 1.8 | NM_057178 | LOC117584 | 1.8 |
| NM_018941 | CLN8 | 1.8 | NM_005397 | PODXL | 1.8 |
| NM_006995 | BTN2A2 | 1.8 | NM_006472 | TXNIP | 1.8 |
| NM_012338 | TM4SF12 | 1.8 | NM_018420 | SLC22A15 | 1.8 |
| XM_378189 | MGC15705 | 1.8 | XM_373594 | LOC387992 | 1.8 |
| XM_370560 | LOC387684 | 1.8 | XM_373958 | LOC388898 | 1.8 |
| NM_000914 | OPRM1 | 1.81 | NM_002833 | PTPN9 | 1.81 |
| NM_005629 | SLC6A8 | 1.81 | NM_000276 | OCRL | 1.81 |
| NM_013398 | ZNF224 | 1.81 | NM_006630 | ZNF234 | 1.81 |
| NM_012069 | ATP1B4 | 1.81 | NM_018268 | FLJ10904 | 1.81 |
| NM_024490 | ATP10A | 1.81 | NM_022789 | IL17E | 1.81 |
| NM_032850 | ZFYVE19 | 1.81 | NM_033229 | TRIM15 | 1.81 |

| | | | | | |
|---|---|---|---|---|---|
| NM_138458 | LOC116143 | 1.81 | NM_138393 | C19ORF32 | 1.81 |
| XM_208908 | LOC283922 | 1.81 | NM_173830 | C6ORF182 | 1.81 |
| NM_173685 | FLJ32440 | 1.81 | NM_003737 | PCDH16 | 1.82 |
| NM_007044 | KATNA1 | 1.82 | NM_016208 | VPS28 | 1.82 |
| NM_018261 | SEC3L1 | 1.82 | NM_019891 | ERO1LB | 1.82 |
| NM_024039 | MIS12 | 1.82 | NM_199044 | MGC22960 | 1.82 |
| NM_002240 | KCNJ6 | 1.83 | NM_016279 | CDH9 | 1.83 |
| NM_001806 | CEBPG | 1.83 | NM_000636 | SOD2 | 1.83 |
| NM_004465 | FGF10 | 1.83 | NM_000149 | FUT3 | 1.83 |
| NM_173704 | MTCO1 | 1.83 | NM_014696 | KIAA0514 | 1.83 |
| NM_012381 | ORC3L | 1.83 | NM_016145 | PTD008 | 1.83 |
| NM_018977 | NLGN3 | 1.83 | NM_018317 | FLJ11082 | 1.83 |
| NM_032143 | DKFZP434B1727 | 1.83 | NM_145260 | ODD | 1.83 |
| XM_376728 | LOC155435 | 1.83 | NM_000909 | NPY1R | 1.84 |
| NM_005938 | MLLT7 | 1.84 | NM_004235 | KLF4 | 1.84 |
| NM_000055 | BCHE | 1.84 | NM_021080 | DAB1 | 1.84 |
| NM_018669 | WDR4 | 1.84 | NM_002279 | KRTHA3B | 1.84 |
| NM_005676 | RBM10 | 1.84 | NM_025010 | KIAA0795 | 1.84 |
| NM_032706 | MGC12966 | 1.84 | NM_207365 | MGC72001 | 1.84 |
| XM_292085 | LOC341457 | 1.84 | NM_020524 | PBXIP1 | 1.85 |
| NM_000049 | ASPA | 1.85 | NM_005363 | MAGEA6 | 1.85 |
| NM_005095 | ZNF262 | 1.85 | NM_006103 | WFDC2 | 1.85 |
| NM_021637 | FLJ14084 | 1.85 | NM_031458 | BAL | 1.85 |
| NM_138394 | LOC92906 | 1.85 | XM_379498 | LOC401363 | 1.85 |
| XM_375589 | LOC400681 | 1.85 | NM_003687 | PDLIM4 | 1.86 |
| NM_003660 | PPFIA3 | 1.86 | NM_000851 | GSTM5 | 1.86 |
| NM_033554 | HLA-DPA1 | 1.86 | NM_015483 | KBTBD2 | 1.86 |
| NM_015702 | C2ORF25 | 1.86 | NM_016510 | SCLY | 1.86 |
| XM_371399 | C20ORF142 | 1.86 | NM_152637 | MGC17301 | 1.86 |
| NM_178822 | IGSF10 | 1.86 | NM_001669 | ARSD | 1.86 |
| NM_003576 | STK24 | 1.87 | NM_178471 | GPR119 | 1.87 |
| NM_018450 | ARID1A | 1.87 | NM_002108 | HAL | 1.87 |
| NM_001861 | COX4I1 | 1.87 | NM_018358 | ABCF3 | 1.87 |
| NM_006694 | JTB | 1.87 | NM_020195 | C14ORF124 | 1.87 |
| XM_064190 | FLJ40311 | 1.87 | NM_172070 | ZNF650 | 1.87 |
| NM_178820 | FBXO27 | 1.87 | NM_175876 | EXOC8 | 1.87 |
| NM_153486 | LDHD | 1.87 | XM_373691 | LOC388283 | 1.87 |
| NM_000549 | TSHB | 1.88 | NM_001491 | GCNT2 | 1.88 |
| NM_020726 | NLN | 1.88 | NM_000036 | AMPD1 | 1.88 |
| NM_000409 | GUCA1A | 1.88 | NM_012318 | LETM1 | 1.88 |
| NM_144640 | IL17RE | 1.88 | XM_372159 | LOC389813 | 1.88 |
| NM_015978 | TNNI3K | 1.89 | NM_004087 | DLG1 | 1.89 |
| NM_003431 | ZNF124 | 1.89 | NM_002584 | PAX7 | 1.89 |
| NM_004918 | TCL1B | 1.89 | NM_000751 | CHRND | 1.89 |
| NM_022731 | NUCKS | 1.89 | NM_003038 | SLC1A4 | 1.89 |
| NM_016474 | LOC51244 | 1.89 | NM_017747 | ANKHD1 | 1.89 |

| | | | | | |
|---|---|---|---|---|---|
| NM_022770 | FLJ13912 | 1.89 | XM_171094 | LOC253314 | 1.89 |
| XM_373541 | LOC387873 | 1.89 | NM_214711 | LOC401137 | 1.89 |
| NM_002154 | HSPA4 | 1.9 | NM_014488 | RAB30 | 1.9 |
| NM_032147 | USP44 | 1.9 | NM_004355 | CD74 | 1.9 |
| NM_013248 | NXT1 | 1.9 | NM_024083 | ASPSCR1 | 1.9 |
| NM_004374 | COX6C | 1.9 | NM_018056 | FLJ10315 | 1.9 |
| NM_025182 | KIAA1539 | 1.9 | XM_371174 | ZNF283 | 1.9 |
| XM_370541 | FLJ44037 | 1.9 | NM_001059 | TACR3 | 1.91 |
| NM_003350 | UBE2V2 | 1.91 | NM_032288 | DKFZP761B1514 | 1.91 |
| NM_058192 | C16ORF40 | 1.91 | XM_376148 | LOC401015 | 1.91 |
| NM_005327 | HADHSC | 1.92 | NM_020919 | ALS2 | 1.92 |
| NM_001223 | CASP1 | 1.92 | NM_014438 | IL1F8 | 1.92 |
| XM_051264 | TXNRD3 | 1.92 | XM_048825 | KIAA1026 | 1.92 |
| NM_002298 | LCP1 | 1.92 | NM_003635 | NDST2 | 1.92 |
| NM_020411 | GAGED2 | 1.92 | NM_016118 | NYREN18 | 1.92 |
| XM_378184 | KIAA1383 | 1.92 | NM_018281 | FLJ10948 | 1.92 |
| XM_045907 | FAM40B | 1.92 | NM_023923 | PHACTR4 | 1.92 |
| NM_144568 | C14ORF9 | 1.92 | NM_178127 | ANGPTL5 | 1.92 |
| NM_207359 | LOC339896 | 1.92 | NM_199344 | LOC375035 | 1.92 |
| XM_293514 | LOC344620 | 1.92 | NM_213723 | C13ORF25 | 1.92 |
| NM_001248 | ENTPD3 | 1.93 | NM_021159 | RAP1GDS1 | 1.93 |
| NM_002837 | PTPRB | 1.93 | NM_016579 | 8D6A | 1.93 |
| NM_007058 | CAPN11 | 1.93 | NM_013372 | GREM1 | 1.93 |
| NM_017975 | FLJ10036 | 1.93 | NM_173510 | FLJ33814 | 1.93 |
| NM_002715 | PPP2CA | 1.94 | NM_022825 | PORCN | 1.94 |
| NM_020786 | PDP2 | 1.94 | NM_020673 | RAB22A | 1.94 |
| NM_014477 | C20ORF10 | 1.94 | NM_015957 | MMRP19 | 1.94 |
| NM_017791 | C14ORF58 | 1.94 | NM_022471 | GMCL1L | 1.94 |
| NM_024103 | SLC25A23 | 1.94 | NM_178517 | PIGW | 1.94 |
| NM_203371 | LOC387758 | 1.94 | XM_211694 | LOC284931 | 1.94 |
| NM_014693 | ECE2 | 1.95 | NM_006873 | SBLF | 1.95 |
| NM_052903 | TUBGCP5 | 1.95 | XM_377949 | LOC402282 | 1.95 |
| XM_375619 | LOC400705 | 1.95 | XM_371097 | LOC388444 | 1.95 |
| NM_002543 | OLR1 | 1.96 | NM_000749 | CHRNB3 | 1.96 |
| NM_020546 | ADCY2 | 1.96 | NM_006539 | CACNG3 | 1.96 |
| XM_032996 | KIAA0819 | 1.96 | NM_019086 | FLJ20674 | 1.96 |
| NM_194252 | C9ORF20 | 1.96 | NM_152672 | OSTALPHA | 1.96 |
| NM_172241 | CTAGE-2 | 1.96 | XM_372921 | LOC391358 | 1.96 |
| XM_376841 | LOC401509 | 1.96 | NM_153835 | GPR113 | 1.97 |
| NM_001667 | ARL2 | 1.97 | NM_006078 | CACNG2 | 1.97 |
| NM_017435 | SLCO1C1 | 1.97 | NM_021180 | SOM | 1.97 |
| NM_152317 | DEPDC4 | 1.97 | NM_153362 | C6ORF158 | 1.97 |
| NM_139248 | LIPH | 1.97 | XM_293802 | LOC345378 | 1.97 |
| XM_294456 | LOC346910 | 1.97 | NM_012280 | FTSJ1 | 1.98 |
| NM_002390 | ADAM11 | 1.98 | NM_173709 | MTND2 | 1.98 |
| XM_050478 | KIAA1202 | 1.98 | NM_024057 | NUP37 | 1.98 |

| | | |
|---|---|---|
| NM_058188 | C21ORF67 | 1.98 |
| NM_152311 | MGC32871 | 1.98 |
| NM_080743 | SRRP35 | 1.98 |
| NM_003159 | CDKL5 | 1.99 |
| NM_001001349 | NKIRAS2 | 1.99 |
| NM_006306 | SMC1L1 | 1.99 |
| NM_015065 | SLAC2-B | 1.99 |
| XM_085347 | LOC146167 | 1.99 |
| NM_003926 | MBD3 | 2 |
| XM_376044 | STAF65(GAMMA) | 2 |
| NM_018269 | SIPL | 2 |
| NM_000376 | VDR | 2.01 |
| NM_016271 | RNF138 | 2.01 |
| XM_371195 | MGC35045 | 2.01 |
| NM_004375 | COX11 | 2.02 |
| NM_007351 | MMRN1 | 2.02 |
| NM_054017 | C8ORF12 | 2.02 |
| NM_004300 | ACP1 | 2.03 |
| NM_016146 | TRAPPC4 | 2.03 |
| XM_070233 | LOC137107 | 2.03 |
| NM_001278 | CHUK | 2.04 |
| NM_016139 | CHCHD2 | 2.04 |
| NM_024894 | FLJ14075 | 2.04 |
| NM_173683 | C8ORF21 | 2.04 |
| NM_001005243 | OR9K2 | 2.04 |
| NM_003151 | STAT4 | 2.05 |
| NM_173039 | AQP11 | 2.05 |
| NM_001001731 | HHCM | 2.05 |
| NM_005526 | HSF1 | 2.06 |
| NM_032848 | FLJ14827 | 2.06 |
| NM_033107 | LOC85865 | 2.06 |
| XM_373639 | LOC388144 | 2.06 |
| NM_024885 | TAF7L | 2.07 |
| NM_002430 | MN1 | 2.07 |
| NM_015926 | ZSIG11 | 2.07 |
| NM_004306 | ANXA13 | 2.08 |
| XM_371086 | LOC388432 | 2.08 |
| NM_006531 | TTC10 | 2.09 |
| NM_018443 | ZNF302 | 2.09 |
| NM_213604 | THSD6 | 2.09 |
| XM_371948 | LOC389549 | 2.09 |
| NM_006560 | CUGBP1 | 2.1 |
| NM_016027 | LACTB2 | 2.1 |
| NM_024549 | FLJ21127 | 2.1 |
| NM_205848 | SYT6 | 2.1 |
| XM_370848 | LOC388104 | 2.1 |

| | | |
|---|---|---|
| NM_145167 | PIGM | 1.98 |
| NM_145046 | CALR3 | 1.98 |
| XM_294521 | FLJ43950 | 1.98 |
| NM_014423 | AF5Q31 | 1.99 |
| NM_003430 | ZNF91 | 1.99 |
| NM_003779 | B4GALT3 | 1.99 |
| XM_378372 | LOC256021 | 1.99 |
| NM_003845 | DYRK4 | 2 |
| NM_005810 | KLRG1 | 2 |
| NM_016598 | ZDHHC3 | 2 |
| NM_006770 | MARCO | 2.01 |
| NM_005415 | SLC20A1 | 2.01 |
| NM_032131 | ARMC2 | 2.01 |
| XM_370982 | LOC388275 | 2.01 |
| NM_000628 | IL10RB | 2.02 |
| NM_003615 | SLC4A7 | 2.02 |
| NM_053001 | OSR2 | 2.02 |
| NM_001399 | ED1 | 2.03 |
| XM_372747 | FLJ00060 | 2.03 |
| XM_370629 | LOC387775 | 2.03 |
| NM_006886 | ATP5E | 2.04 |
| NM_018359 | FLJ11200 | 2.04 |
| NM_144609 | FLJ31795 | 2.04 |
| XM_379671 | LOC401553 | 2.04 |
| NM_005766 | FARP1 | 2.05 |
| NM_013449 | BAZ2A | 2.05 |
| NM_178171 | GSDM | 2.05 |
| NM_007050 | PTPRT | 2.06 |
| NM_017659 | FLJ20084 | 2.06 |
| NM_032881 | LSM10 | 2.06 |
| XM_059104 | LOC127003 | 2.06 |
| XM_377696 | LOC402036 | 2.06 |
| NM_000490 | AVP | 2.07 |
| NM_006943 | SOX12 | 2.07 |
| XM_379145 | LOC401020 | 2.07 |
| XM_371917 | LOC389517 | 2.08 |
| NM_001771 | CD22 | 2.09 |
| NM_018297 | NGLY1 | 2.09 |
| NM_144688 | FLJ32658 | 2.09 |
| XM_294249 | LOC340265 | 2.09 |
| NM_001005323 | OR5AK2 | 2.09 |
| NM_002232 | KCNA3 | 2.1 |
| NM_018695 | ERBB2IP | 2.1 |
| NM_033317 | ZD52F10 | 2.1 |
| NM_139016 | LOC128439 | 2.1 |
| XM_377034 | LOC401612 | 2.1 |

| | | |
|---|---|---|
| NM_002405 | MFNG | 2.11 |
| XM_058404 | PNLIPRP3 | 2.11 |
| XM_210787 | LOC284433 | 2.11 |
| XM_380159 | LOC402632 | 2.11 |
| NM_004554 | NFATC4 | 2.12 |
| NM_012285 | KCNH4 | 2.12 |
| NM_145012 | C10ORF9 | 2.12 |
| NM_032928 | MGC14141 | 2.12 |
| NM_000738 | CHRM1 | 2.14 |
| NM_002832 | PTPN7 | 2.14 |
| XM_371717 | ODZ3 | 2.14 |
| NM_001003799 | TARP | 2.14 |
| NM_018036 | C14ORF103 | 2.15 |
| NM_198841 | C9ORF10OS | 2.15 |
| XM_291028 | DKFZP434A128 | 2.15 |
| NM_018174 | BPY2IP1 | 2.16 |
| NM_032193 | AYP1 | 2.16 |
| NM_007085 | FSTL1 | 2.17 |
| NM_003192 | TBCC | 2.17 |
| NM_052885 | SLC2A13 | 2.17 |
| NM_153442 | GPR26 | 2.18 |
| NM_016505 | PS1D | 2.18 |
| NM_021784 | FOXA2 | 2.19 |
| NM_022062 | PKNOX2 | 2.19 |
| NM_004945 | DNM2 | 2.19 |
| NM_018342 | FLJ11155 | 2.19 |
| NM_001002029 | C4B | 2.19 |
| NM_017721 | FLJ20241 | 2.2 |
| XM_061871 | FAT3 | 2.2 |
| XM_290722 | LOC339123 | 2.2 |
| XM_376965 | LOC401570 | 2.2 |
| NM_018339 | RFK | 2.21 |
| NM_012087 | GTF3C5 | 2.21 |
| NM_080751 | TMC2 | 2.21 |
| XM_291120 | SLC6A19 | 2.21 |
| NM_003386 | ZAN | 2.22 |
| NM_006517 | SLC16A2 | 2.22 |
| NM_020552 | TCL6 | 2.22 |
| XM_376899 | LOC401546 | 2.22 |
| NM_000895 | LTA4H | 2.23 |
| NM_032687 | MGC13010 | 2.23 |
| XM_379025 | LOC400877 | 2.23 |
| NM_000761 | CYP1A2 | 2.24 |
| NM_006603 | STAG2 | 2.24 |
| NM_030919 | C20ORF129 | 2.24 |
| NM_014562 | OTX1 | 2.25 |
| NM_007073 | BVES | 2.11 |
| NM_181806 | NRPS998 | 2.11 |
| XM_373082 | LOC391784 | 2.11 |
| XM_378436 | LOC400163 | 2.11 |
| NM_016437 | TUBG2 | 2.12 |
| NM_000445 | PLEC1 | 2.12 |
| XM_375065 | ZNF409 | 2.12 |
| XM_209607 | LOC285429 | 2.12 |
| NM_000455 | STK11 | 2.14 |
| NM_002538 | OCLN | 2.14 |
| NM_014399 | TM4SF13 | 2.14 |
| XM_166453 | TTBK1 | 2.15 |
| NM_033204 | ZNF101 | 2.15 |
| NM_181788 | LOC341567 | 2.15 |
| XM_065445 | LOC129870 | 2.15 |
| NM_030777 | SLC2A10 | 2.16 |
| NM_144598 | MGC24976 | 2.16 |
| NM_003387 | WASPIP | 2.17 |
| XM_047707 | SLC39A10 | 2.17 |
| NM_144715 | FLJ25200 | 2.17 |
| NM_003143 | SSBP1 | 2.18 |
| NM_152481 | FLJ25660 | 2.18 |
| AF113887 | IGKC | 2.19 |
| NM_004957 | FPGS | 2.19 |
| NM_017750 | FLJ20296 | 2.19 |
| NM_175058 | LOC144100 | 2.19 |
| NM_000831 | GRIK3 | 2.2 |
| NM_018241 | FLJ10846 | 2.2 |
| NM_173553 | FLJ25801 | 2.2 |
| XM_372046 | LOC389667 | 2.2 |
| XM_374490 | LOC392748 | 2.2 |
| NM_012415 | RAD54B | 2.21 |
| NM_017643 | MBTD1 | 2.21 |
| NM_178832 | C10ORF83 | 2.21 |
| NM_133646 | ZAK | 2.22 |
| NM_002844 | PTPRK | 2.22 |
| NM_007041 | ATE1 | 2.22 |
| XM_375809 | LOC126669 | 2.22 |
| NM_001005484 | OR4F5 | 2.22 |
| NM_016627 | LOC51321 | 2.23 |
| NM_144574 | WDR20 | 2.23 |
| NM_002115 | HK3 | 2.24 |
| NM_007125 | UTY | 2.24 |
| NM_015938 | CGI-07 | 2.24 |
| NM_199345 | LOC375133 | 2.24 |
| NM_139055 | ADAMTS15 | 2.25 |

| | | | | | |
|---|---|---|---|---|---|
| NM_013341 | PTD004 | 2.25 | XM_375456 | DKFZP761G2113 | 2.25 |
| NM_024941 | FLJ13611 | 2.25 | XM_292504 | LOC342357 | 2.25 |
| NM_005111 | CRYZL1 | 2.26 | XM_376905 | EGFL5 | 2.26 |
| NM_032274 | DKFZP547F072 | 2.26 | XM_378787 | LOC284395 | 2.26 |
| NM_012188 | FOXI1 | 2.27 | NM_015002 | FBXO21 | 2.27 |
| NM_013316 | CNOT4 | 2.27 | NM_001444 | FABP5 | 2.27 |
| NM_014509 | DJ222E13.1 | 2.27 | NM_006650 | CPLX2 | 2.27 |
| NM_021808 | GALNT9 | 2.27 | NM_018054 | ARHGAP17 | 2.27 |
| XM_091156 | LOC161823 | 2.27 | XM_372626 | LOC390705 | 2.27 |
| NM_012219 | MRAS | 2.28 | NM_006037 | HDAC4 | 2.28 |
| NM_152517 | FLJ30990 | 2.28 | NM_207502 | C6ORF122 | 2.28 |
| XM_047770 | LOC144245 | 2.28 | NM_176818 | 15E1.2 | 2.28 |
| NM_006268 | DPF2 | 2.29 | NM_001647 | APOD | 2.29 |
| XM_370654 | KIAA1726 | 2.29 | NM_138417 | MGC20419 | 2.29 |
| NM_207372 | SH2D4B | 2.29 | NM_016530 | RAB8B | 2.3 |
| NM_018222 | PARVA | 2.31 | NM_003308 | TSPY1 | 2.31 |
| NM_016622 | MRPL35 | 2.31 | NM_006243 | PPP2R5A | 2.32 |
| NM_144602 | MGC33367 | 2.32 | NM_203300 | LOC147727 | 2.32 |
| XM_117014 | LOC197317 | 2.32 | NM_004580 | RAB27A | 2.33 |
| NM_000338 | SLC12A1 | 2.33 | NM_002109 | HARS | 2.33 |
| NM_018168 | C14ORF105 | 2.33 | NM_018207 | FLJ10759 | 2.33 |
| XM_370616 | LOC338645 | 2.33 | NM_004049 | BCL2A1 | 2.34 |
| NM_006584 | CCT6B | 2.34 | NM_152251 | DEFB106 | 2.34 |
| XM_378949 | LOC400817 | 2.34 | NM_002677 | PMP2 | 2.35 |
| NM_002903 | RCV1 | 2.35 | NM_013389 | NPC1L1 | 2.35 |
| NM_022117 | SE20-4 | 2.35 | NM_032947 | NID67 | 2.35 |
| NM_012206 | HAVCR1 | 2.36 | NM_004209 | SYNGR3 | 2.36 |
| NM_016447 | MPP6 | 2.36 | NM_152834 | TMEM18 | 2.36 |
| XM_293581 | LOC344760 | 2.36 | XM_375914 | LOC400842 | 2.36 |
| NM_001292 | CLK3 | 2.37 | NM_000389 | CDKN1A | 2.37 |
| NM_020792 | KIAA1363 | 2.37 | NM_030882 | APOL2 | 2.37 |
| NM_152613 | MGC26816 | 2.37 | XM_370980 | LOC388271 | 2.37 |
| XM_371009 | LOC388327 | 2.37 | NM_153251 | FLJ25952 | 2.38 |
| NM_017947 | MOCOS | 2.38 | NM_024760 | FLJ14009 | 2.38 |
| NM_006509 | RELB | 2.39 | NM_178519 | FLJ39421 | 2.39 |
| NM_005777 | RBM6 | 2.4 | NM_001933 | DLST | 2.4 |
| NM_000857 | GUCY1B3 | 2.4 | NM_005535 | IL12RB1 | 2.4 |
| XM_171536 | MRGPRE | 2.4 | NM_052967 | MAS1L | 2.41 |
| NM_007136 | ZNF80 | 2.41 | XM_041191 | KIAA0931 | 2.41 |
| NM_020695 | TCEB3BP1 | 2.41 | NM_032487 | ARPM1 | 2.42 |
| NM_176815 | LOC200895 | 2.42 | NM_023012 | FLJ11021 | 2.43 |
| BC020240 | IGHM | 2.44 | NM_003538 | HIST1H4A | 2.44 |
| NM_023073 | FLJ13231 | 2.44 | NM_018571 | ALS2CR2 | 2.45 |
| NM_005604 | POU3F2 | 2.45 | NM_005627 | SGK | 2.46 |
| NM_003028 | SHB | 2.46 | NM_016004 | C20ORF9 | 2.46 |
| NM_058168 | GDEP | 2.46 | NM_005379 | MYO1A | 2.47 |

| | | | | | |
|---|---|---|---|---|---|
| NM_015339 | ADNP | 2.47 | NM_022495 | C14ORF135 | 2.47 |
| NM_152762 | FLJ32880 | 2.47 | XM_371476 | LOC388922 | 2.47 |
| NM_004995 | MMP14 | 2.48 | NM_003778 | B4GALT4 | 2.48 |
| NM_002515 | NOVA1 | 2.49 | NM_021071 | DO | 2.49 |
| NM_020347 | LZTFL1 | 2.49 | XM_375599 | FLJ21369 | 2.49 |
| NM_145201 | PP3856 | 2.5 | XM_034819 | ZNF629 | 2.51 |
| NM_018974 | UNC93A | 2.51 | NM_144678 | TOM1L2 | 2.51 |
| XM_113796 | LOC196996 | 2.51 | NM_022447 | PAPD5 | 2.52 |
| NM_006646 | WASF3 | 2.53 | NM_012253 | TKTL1 | 2.53 |
| NM_001782 | CD72 | 2.53 | NM_032364 | DNAJC14 | 2.53 |
| NM_144647 | MGC26610 | 2.53 | NM_019020 | TBC1D16 | 2.53 |
| NM_199352 | LOC387601 | 2.53 | NM_001952 | E2F6 | 2.55 |
| NM_021818 | SAV1 | 2.55 | XM_291344 | FLJ12649 | 2.55 |
| XM_380091 | LOC402466 | 2.55 | XM_370580 | LOC387716 | 2.55 |
| NM_012119 | CCRK | 2.56 | NM_006948 | STCH | 2.56 |
| NM_003218 | TERF1 | 2.56 | XM_084578 | PPFIBP2 | 2.56 |
| NM_018622 | PSARL | 2.56 | NM_007193 | ANXA10 | 2.57 |
| AF120323 | TIAM2 | 2.57 | XM_292895 | LOC344065 | 2.57 |
| NM_007194 | CHEK2 | 2.58 | NM_014245 | RNF7 | 2.58 |
| NM_138791 | C14ORF148 | 2.58 | NM_005735 | ACTR1B | 2.59 |
| NM_012159 | FBXL3P | 2.59 | NM_002039 | GAB1 | 2.59 |
| NM_018846 | KLHL7 | 2.59 | NM_018970 | GPR85 | 2.6 |
| NM_000082 | CKN1 | 2.6 | XM_376556 | C6ORF70 | 2.6 |
| NM_145175 | NSE1 | 2.6 | XM_371939 | LOC389541 | 2.6 |
| NM_014497 | NP220 | 2.61 | NM_000841 | GRM4 | 2.62 |
| NM_006941 | SOX10 | 2.62 | NM_032750 | MGC15429 | 2.62 |
| XM_062872 | LOC121952 | 2.62 | NM_000365 | TPI1 | 2.63 |
| NM_033281 | MRPS36 | 2.63 | NM_000676 | ADORA2B | 2.64 |
| NM_006911 | RLN1 | 2.64 | XM_375080 | KIAA0831 | 2.64 |
| XM_027162 | DMRTA2 | 2.64 | NM_005171 | ATF1 | 2.66 |
| NM_001236 | CBR3 | 2.66 | NM_016406 | UFC1 | 2.66 |
| NM_199001 | MGC59937 | 2.66 | NM_000864 | HTR1D | 2.67 |
| XM_166526 | USP42 | 2.67 | NM_002831 | PTPN6 | 2.68 |
| NM_178864 | NXF | 2.68 | NM_134444 | NALP4 | 2.68 |
| NM_022491 | SDS3 | 2.69 | XM_371823 | LOC389394 | 2.69 |
| NM_000697 | ALOX12 | 2.7 | NM_173483 | FLJ39501 | 2.71 |
| XM_377444 | LOC401859 | 2.71 | NM_015356 | SCRIB | 2.72 |
| NM_004626 | WNT11 | 2.73 | NM_153813 | ZFPM1 | 2.73 |
| XM_060597 | LOC127665 | 2.74 | XM_371469 | LOC388906 | 2.75 |
| NM_002516 | NOVA2 | 2.76 | NM_013348 | KCNJ14 | 2.77 |
| NM_178468 | C20ORF128 | 2.77 | NM_203423 | LOC389199 | 2.78 |
| NM_016030 | TTC15 | 2.79 | NM_024591 | FLJ11749 | 2.79 |
| NM_004654 | USP9Y | 2.81 | NM_033057 | OR2B2 | 2.83 |
| NM_032192 | PPP1R1B | 2.84 | NM_005934 | MLLT1 | 2.84 |
| XM_373499 | LOC387765 | 2.86 | NM_052928 | SMYD4 | 2.88 |
| NM_005568 | LHX1 | 2.92 | NM_152250 | DEFB105 | 2.92 |

| | | | | | |
|---|---|---|---|---|---|
| NM_005207 | CRKL | 2.94 | NM_007166 | PICALM | 2.94 |
| NM_017594 | DIRAS2 | 2.94 | NM_175571 | HIAN6 | 2.94 |
| NM_017771 | PXK | 2.95 | NM_054030 | MRGX2 | 2.95 |
| NM_207371 | FLJ45187 | 2.95 | NM_144726 | FLJ31951 | 2.96 |
| NM_002061 | GCLM | 2.97 | NM_015416 | HCCR1 | 2.98 |
| NM_175709 | CBX7 | 2.98 | NM_006583 | RRH | 2.99 |
| NM_183240 | PR1 | 2.99 | NM_139175 | RNF133 | 3 |
| NM_203403 | C9ORF150 | 3 | NM_006982 | CART1 | 3.05 |
| NM_181620 | KRTAP22-1 | 3.05 | NM_023936 | MRPS34 | 3.1 |
| NM_139076 | FLJ13614 | 3.1 | NM_182640 | MRPS9 | 3.13 |
| NM_000244 | MEN1 | 3.14 | NM_022146 | GPR147 | 3.15 |
| NM_015683 | ARRDC2 | 3.15 | NM_018376 | NIPSNAP3B | 3.17 |
| NM_004527 | MEOX1 | 3.18 | NM_023914 | GPR86 | 3.2 |
| NM_032823 | C9ORF3 | 3.21 | NM_171999 | SALL3 | 3.22 |
| NM_014063 | HIP-55 | 3.23 | NM_002976 | SCN7A | 3.28 |
| NM_002552 | ORC4L | 3.28 | NM_015345 | DAAM2 | 3.38 |
| NM_032311 | POLDIP3 | 3.44 | NM_005439 | MLF2 | 3.48 |
| NM_002897 | RBMS1 | 3.5 | XM_058999 | LOC126208 | 3.52 |
| NM_013323 | SNX11 | 3.53 | NM_016217 | HECA | 3.54 |
| NM_130775 | XAGE-5 | 3.6 | NM_001001693 | FLJ46257 | 3.69 |
| XM_374517 | LOC392779 | 3.73 | NM_004206 | SEC22L3 | 3.83 |
| NM_016508 | CDKL3 | 3.87 | NM_000436 | OXCT1 | 4.05 |
| NM_004380 | CREBBP | 4.05 | XM_372741 | LOC390956 | 4.08 |
| NM_012443 | SPAG6 | 4.17 | NM_032810 | ATAD1 | 4.18 |
| NM_002285 | LAF4 | 4.28 | NM_016264 | ZNF44 | 4.28 |
| NM_006874 | ELF2 | 4.36 | NM_003887 | DDEF2 | 4.62 |
| NM_152558 | KIAA1023 | 4.76 | NM_173537 | GTF2IRD2 | 5.08 |
| NM_020155 | C11ORF4 | 5.32 | | | |
