# Supplementary material for: Genome-wide RNA interference analysis of renal carcinoma survival regulators identifies MCT4 as a Warburg effect metabolic target
Source: J Pathol. 2012 Apr 18;227(2):146–56. doi: 10.1002/path.4006 (PMC3504091; doi:10.1002/path.4006)
Supplement: Supplementary file 2 [file path0227-0146-SD1.pdf]

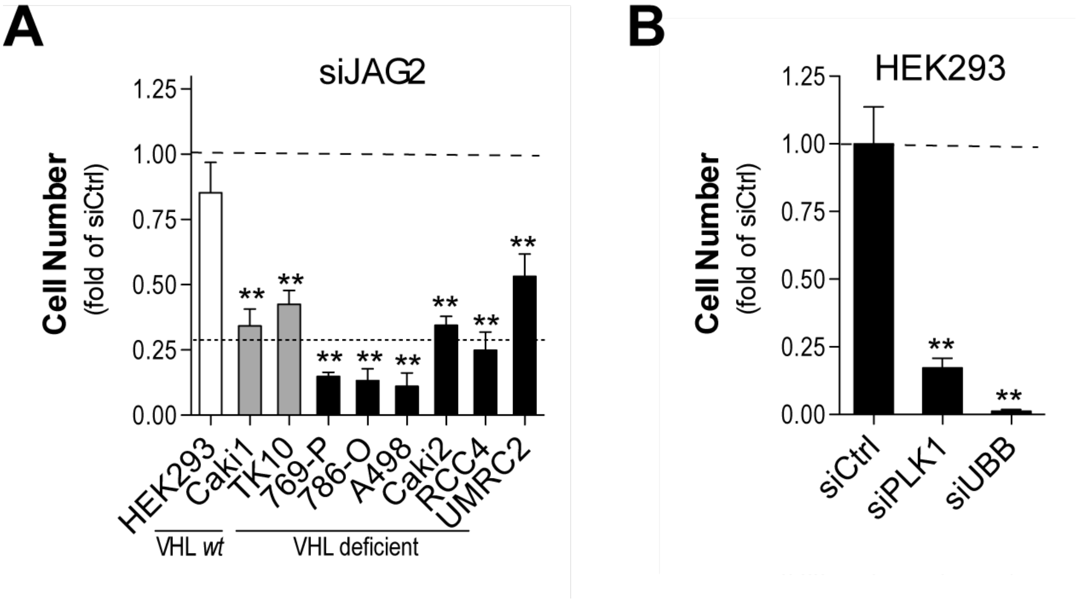

Figure S1

A) A panel of renal cell carcinoma cell lines is highly sensitive to silencing of JAG2. Four days after transfection with JAG2 siRNAs cell numbers were determined and normalised to non-targeting siRNAs. The embryonic kidney cell line HEK293 was used as a control. B) Silencing of PLK1 and UBB efficiently reduce cell numbers in HEK293 four days after transfection. Cell numbers were normalised to that of non-targeting siRNA. Graphs shows mean ± SD (n=6). ** $p < 0.001$
