# Supplementary material for: Genome-wide RNA interference analysis of renal carcinoma survival regulators identifies MCT4 as a Warburg effect metabolic target
Source: J Pathol. 2012 Apr 18;227(2):146–56. doi: 10.1002/path.4006 (PMC3504091; doi:10.1002/path.4006)
Supplement: Supplementary file 3 [file path0227-0146-SD2.pdf]

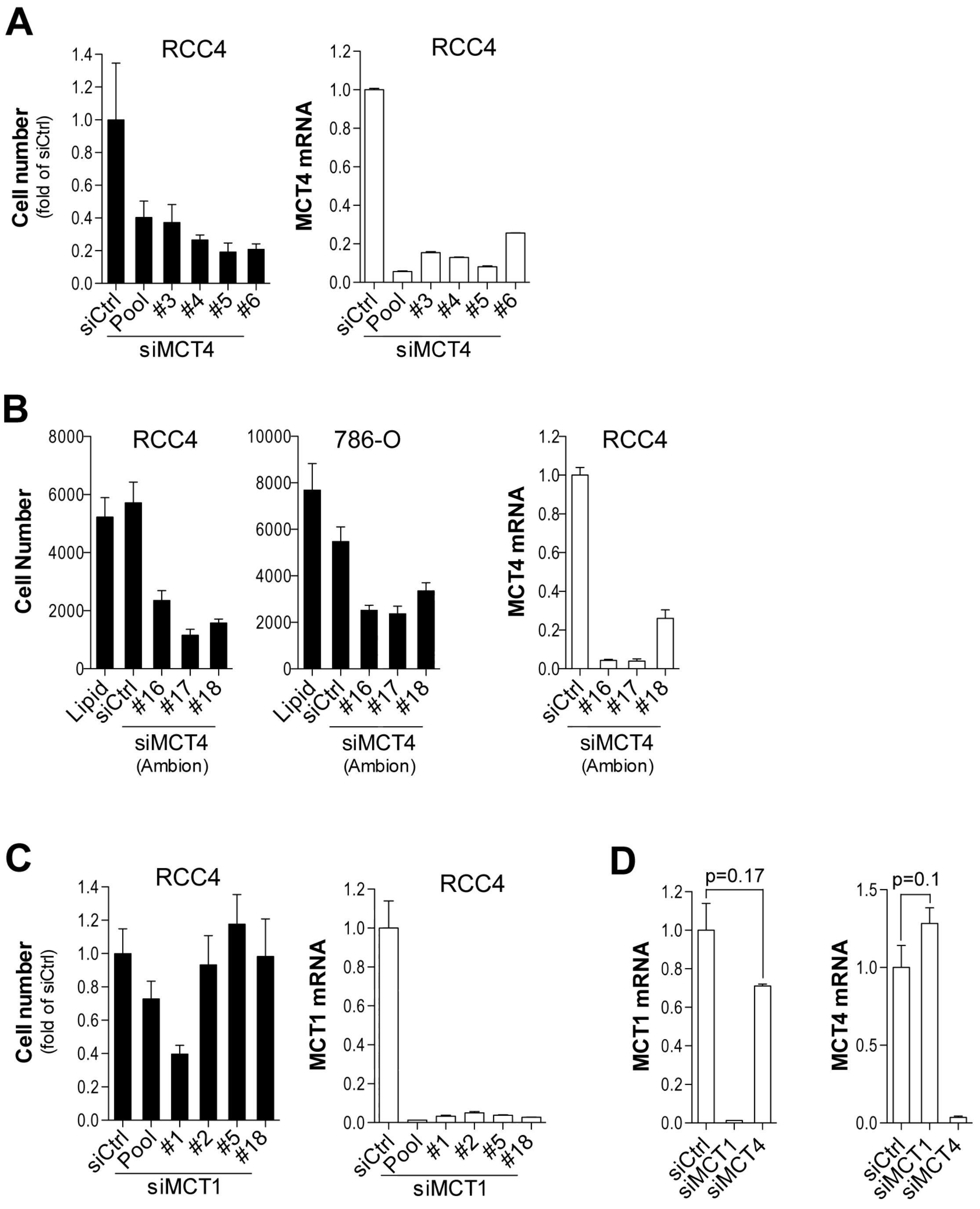

Figure S2

A) Effect of MCT4 silencing on cell number correlates well with silencing efficiency. RCC4 were transfected in parallel for cell number (after 4 days) and mRNA level determination (after 2 days). MCT4 expression levels were determined by Q-PCR and normalised to B2M expression. Graphs show mean ± SD (n=3).

B) MCT4 silencing phenotype is also observed using different siRNA sequences obtained from another manufacturer. RCC4 and 786-O were transfected in parallel for cell number (after 4 days) and mRNA level determination in parallel (after 2 days, RCC4 only). Graphs show mean ± SD (n=6 for cell number; n=3 for mRNA expression).

C) The effect of silencing MCT1 on cell number is not significant despite efficient silencing. RCC4 were transfected in parallel for cell number (after 4 days) and mRNA level determination (after 2 days). MCT4 expression levels were determined by Q-PCR and normalised with B2M expression. Graphs show mean ± SD (n=6 for cell number; n=3 for mRNA expression).

D) MCT1 and MCT4 silencing is specific. MCT1 and MCT4 expression levels were determined 2 days after transfection by Q-PCR and normalised with B2M expression. Graphs show mean ± SD (n=3).
