# Supplementary material for: Genome-wide RNA interference analysis of renal carcinoma survival regulators identifies MCT4 as a Warburg effect metabolic target
Source: J Pathol. 2012 Apr 18;227(2):146–56. doi: 10.1002/path.4006 (PMC3504091; doi:10.1002/path.4006)
Supplement: Supplementary file 4 [file path0227-0146-SD3.pdf]

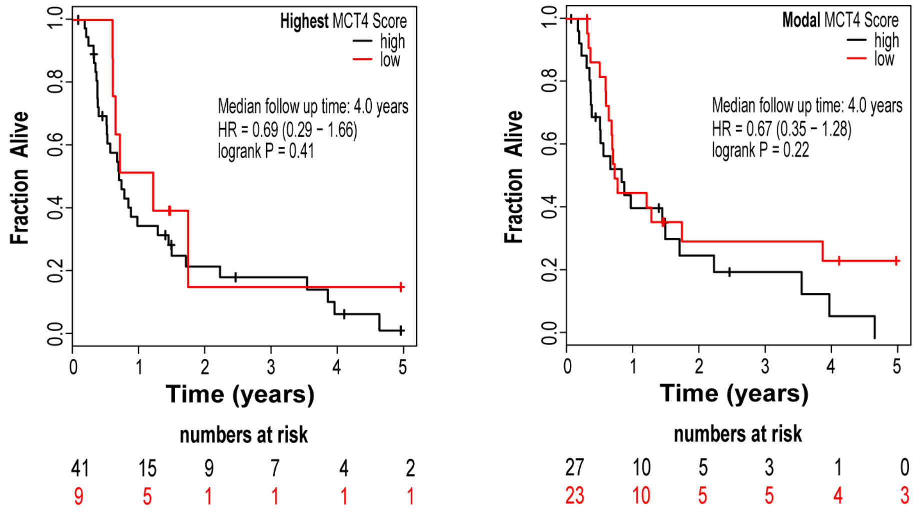

Figure S3

Overall survival (OS) by highest and modal MCT4 staining intensity in tumour specimens of patients with metastatic disease treated with a palliative nephrectomy (low = MCT4 staining intensities 1 or 2, high = MCT4 staining intensities 3 or 4).
