# Supplementary material for: Genome-wide RNA interference analysis of renal carcinoma survival regulators identifies MCT4 as a Warburg effect metabolic target
Source: J Pathol. 2012 Apr 18;227(2):146–56. doi: 10.1002/path.4006 (PMC3504091; doi:10.1002/path.4006)
Supplement: Supplementary file 5 [file path0227-0146-SD4.pdf]

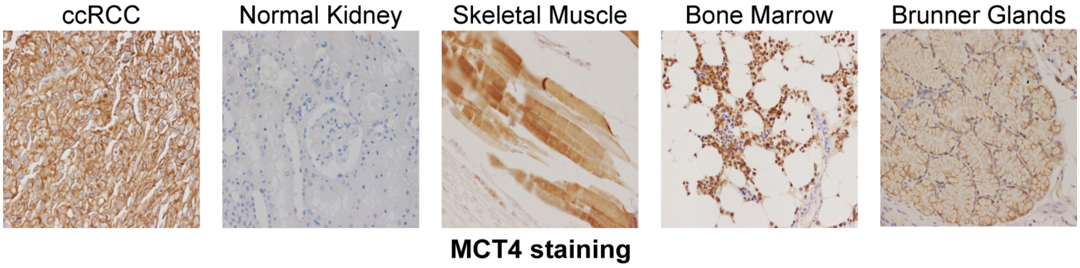

Figure S4

MCT4 expression in normal tissues (20x). The following normal tissues were analysed: bone marrow (trephine), cardiac muscle, cortical brain, duodenum, kidney cortex, liver, lung, skeletal muscle, smooth muscle and skin. Expression was found only in skeletal muscle, bone marrow and the duodenal Brunner glands. ccRCC tissue is shown for comparison.
