# Supplementary material for: Genome-wide RNA interference analysis of renal carcinoma survival regulators identifies MCT4 as a Warburg effect metabolic target
Source: J Pathol. 2012 Apr 18;227(2):146–56. doi: 10.1002/path.4006 (PMC3504091; doi:10.1002/path.4006)
Supplement: Supplementary file 6 [file path0227-0146-SD5.pdf]

Supplementary Materials and Methods

Materials

siRNAs, including the genome-wide library, were from Dharmacon (siGENOME). An an additional set of MCT4 siRNAS was purchased from Ambion (#16, 17 and 18). DAPI (4',6-diamidino-2-phenylindole) was from Roche. All other chemicals were from Sigma.

Cell culture

CcRCC cell lines and HEK293 cells were obtained from the LRI's cell services and cultured in DMEM or RPMI supplemented with 10% FCS as recommended by ATCC.

Genome-wide siRNA screen

RCC4 cells were reverse-transfected in triplicate in 384 well plates with Dharmafect 3 (Dharmacon) and 20 nM of siRNA. After 4 days cells were fixed in 80% ice-cold ethanol, washed with PBS and stained with 1 μg/mL DAPI. Nuclei/cells per well were counted using an Acumen Explorer eX3 (TTP LabTech). A robust Z-score calculation was applied to the data: the median value of samples per plate was subtracted from each well and each well value was divided by the median absolute deviation (MAD) of all sample wells on that plate. In order to account for edge effects a smoothing calculation was applied, based on the median and MAD calculated when comparing the distribution of Z-scores at each well position across all plates within the screen.

Genome-wide siRNA screens in HCT116, PC9, HT1080 and MCF10A cell lines were performed and analysed similarly.

mRNA expression analysis

For the identification of siRNA screen hits overexpressed in ccRCC a normalised expression dataset (GSE14994, published in [1]) was downloaded from GEO. Each column was labelled according to its ccRCC/Normal status and each genes' group averages was determined using a linear model. Standard errors were calculated and moderated using the empirical Bayes method within Bioconductor's [2] limma [3] package. A moderated t-test was performed. Genes were selected by imposing a two-fold threshold on those whose differential expression was statistically significant when controlling for a false discovery rate of 1%. They were mapped to the RCC4 siRNA screen data via their RefSeq identifiers.

The meta-analysis of genes overexpressed in ccRCC was performed on Oncomine™ (Compendia Bioscience, Ann Arbor, MI). All datasets generated with the Affymetrix HT HGU133A array and including both normal renal tissue and ccRCC samples [1, 4-7] were included. Oncomine™ was also used to compare expression data of MCT4 in normal tissue, ccRCC primary tumors and metastases [5].

The Cancer Genome Project cell line mRNA expression dataset (http://www.sanger.ac.uk/genetics/CGP) was used to compare MCT1 and MCT4 expression in ccRCC and non-ccRCC tumour entities. Samples which failed the quality check criteria and from tumour types with less than 20 samples were excluded from the analysis. The raw expression data of the remaining 541 cell lines (including 20 ccRCC cell lines) was RMA normalised with the R-package *affy*. Expression

values of all Affymetrix probes corresponding to the MCT4 or the MCT1 gene were averaged for each tumour type.

Western blot analysis

Cells were lysed with Cell Lysis Buffer (Cell Signaling) or in 1% Triton X-100, 0.25% IPEGAL and 0.25 % Sodium Deoxycholate buffer for CD147 detection, all supplemented with protease inhibitor cocktail (Roche). Antibodies against MCT1 and MCT4 were from Santa Cruz and CD147 from Novus Biologicals. The HRP-conjugated anti-Actin antibody was from Sigma and the HRP-conjugated secondary antibodies from Dako.

Analysis of mRNA expression by Q-PCR

MCT1, 2, 3 and 4 and Beta-2 microglobulin Q-PCR primers and TaqMan PCR master mix were from Applied Biosystems and used according to the manufacturer's instructions in an ABI 7500 FAST machine.

Patient samples

Surgical FFPE specimens from primary ccRCCs and corresponding pathological information (RCC subtype, Fuhrman grade) were obtained from the Royal Marsden Hospital (UK) and the Centre Hospitalier Universitaire de Rennes (France). The Royal Marsden Hospital (UK) contributed samples from 86 patients treated with a curative or palliative nephrectomy. Retrospectively collected RFS and/or OS data were available for these patients. The Centre Hospitalier Universitaire de Rennes

contributed 41 specimens from patients with early stage ccRCC treated in curative intent.

All French patients provided written informed consent for the use of surplus tissues for research purposes. All UK patients treated surgically after 01/09/ 2006 provided written informed consent for the use of surplus tissue samples. The institutional ethics board had waived this for patients treated before that date. The use of these surplus tissues was approved by the institutional ethics board (ethics approval number 09/H0801/4).

<u>MCT4 immunohistochemistry</u>

4 µm sections were cut from FFPE blocks, dewaxed in xylene and cleared in ethanol to water. Sections were microwaved (900W) in 0.1M sodium citrate pH 6 for 15 min. Endogenous peroxidase activity was blocked in 0.3% H_2O_2 for 10 min. Sections were incubated in 10% normal goat serum and then in anti-MCT4 antibody (Santa Cruz, 1:350 dilution) for 1 hour. After incubation in biotinylated Goat anti-Rabbit IgG (Vector) and in Avidin Biotin Complex (Vector) sections were developed with DAB, counterstained in haematoxylin, dehydrated and mounted in DPX.

References

1. Beroukhim R, Brunet JP, Di Napoli A*, et al.* Patterns of gene expression and copy-number alterations in von-hippel lindau disease-associated and sporadic clear cell carcinoma of the kidney. *Cancer Res* 2009; **69**: 4674-4681.
2. Gentleman RC, Carey VJ, Bates DM*, et al.* Bioconductor: open software development for computational biology and bioinformatics. *Genome Biol* 2004; **5**: R80.
3. Smyth GK. Limma: linear models for microarray data. In: Robert Gentleman VC, Wolfgang Huber, Rafael Irizarry and Sandrine Dudoit, ed. *Bioinformatics and Computational Biology Solutions using R and Bioconductor*. Springer: New York 2005:397-420.
4. Gumz ML, Zou H, Kreinest PA*, et al.* Secreted frizzled-related protein 1 loss contributes to tumor phenotype of clear cell renal cell carcinoma. *Clin Cancer Res* 2007; **13**: 4740-4749.
5. Jones J, Otu H, Spentzos D*, et al.* Gene signatures of progression and metastasis in renal cell cancer. *Clin Cancer Res* 2005; **11**: 5730-5739.
6. Lenburg ME, Liou LS, Gerry NP*, et al.* Previously unidentified changes in renal cell carcinoma gene expression identified by parametric analysis of microarray data. *BMC Cancer* 2003; **3**: 31.
7. Yusenko MV, Kuiper RP, Boethe T*, et al.* High-resolution DNA copy number and gene expression analyses distinguish chromophobe renal cell carcinomas and renal oncocytomas. *BMC Cancer* 2009; **9**: 152.
